# PROTAC‐Mediated HDAC7 Protein Degradation Unveils Its Deacetylase‐Independent Proinflammatory Function in Macrophages

**DOI:** 10.1002/advs.202309459

**Published:** 2024-07-25

**Authors:** Kailibinuer Kadier, Tian Niu, Baoli Ding, Boya Chen, Xuxin Qi, Danni Chen, Xirui Cheng, Yizheng Fang, Jiahao Zhou, Wenyi Zhao, Zeqi Liu, Yi Yuan, Zhan Zhou, Xiaowu Dong, Bo Yang, Qiaojun He, Ji Cao, Li Jiang, Cheng‐Liang Zhu

**Affiliations:** ^1^ Institute of Pharmacology & Toxicology Zhejiang Province Key Laboratory of Anti‐Cancer Drug Research College of Pharmaceutical Sciences Zhejiang University Hangzhou 310058 P. R. China; ^2^ Innovation Institute for Artificial Intelligence in Medicine Zhejiang University Hangzhou 310018 P. R. China; ^3^ Engineering Research Center of Innovative Anticancer Drugs Ministry of Education Hangzhou 310058 P. R. China; ^4^ Hangzhou Institute of Innovative Medicine Zhejiang University Hangzhou 310018 P. R. China; ^5^ Cancer Center Zhejiang University Hangzhou 310058 P. R. China; ^6^ Center for Medical Research and Innovation in Digestive System Tumors Ministry of Education Hangzhou 310058 P. R. China; ^7^ School of Medicine Hangzhou City University Hangzhou 310015 P. R. China; ^8^ Center for Drug Safety Evaluation and Research of Zhejiang University Hangzhou 310058 P. R. China

**Keywords:** anti‐inflammatory drug development, HDAC7, proinflammatory cytokines, PROTAC, TLR4 signaling

## Abstract

Class IIa histone deacetylases (Class IIa HDACs) play critical roles in regulating essential cellular metabolism and inflammatory pathways. However, dissecting the specific roles of each class IIa HDAC isoform is hindered by the pan‐inhibitory effect of current inhibitors and a lack of tools to probe their functions beyond epigenetic regulation. In this study, a novel PROTAC‐based compound **B4 is** developed, which selectively targets and degrades HDAC7, resulting in the effective attenuation of a specific set of proinflammatory cytokines in both lipopolysaccharide (LPS)‐stimulated macrophages and a mouse model. By employing **B4** as a molecular probe, evidence is found for a previously explored role of HDAC7 that surpasses its deacetylase function, suggesting broader implications in inflammatory processes. Mechanistic investigations reveal the critical involvement of HDAC7 in the Toll‐like receptor 4 (TLR4) signaling pathway by directly interacting with the TNF receptor‐associated factor 6 and TGFβ‐activated kinase 1 (TRAF6‐TAK1) complex, thereby initiating the activation of the downstream mitogen‐activated protein kinase/nuclear factor‐κB (MAPK/NF‐κB) signaling cascade and subsequent gene transcription. This study expands the insight into HDAC7's role within intricate inflammatory networks and highlights its therapeutic potential as a novel target for anti‐inflammatory treatments.

## Graphical abstract

The research reveals a novel, deacetylase‐independent proinflammatory function of HDAC7 in macrophages using isoform‐selective protein degraders developed for this study. These findings underscore HDAC7's nonenzymatic function within the TLR4 signaling pathway, particularly its involvement in maintaining the TRAF6‐TAK1 complex, and highlight the therapeutic promise of targeting HDAC7 as a novel approach for the treatment of cytokine‐mediated autoinflammatory diseases.

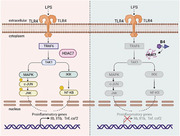

## 1 Introduction

Cytokines are essential chemical messengers secreted by immune cells that orchestrate normal immune responses. By interacting with their cellular receptors, cytokines activate downstream signaling cascades, triggering immune reactions that protect the body from inflammation or infection.^[^ ^1^ ^]^ Therefore, maintaining a dynamic balance of proinflammatory and anti‐inflammatory cytokines is crucial for immune homeostasis. Conversely, dysregulated cytokine production can disrupt this balance, leading to immune disorders such as autoimmune diseases characterized by the immune system mistakenly attacking the body's own tissues.^[^ ^2^ ^]^ Numerous studies have shown that autoimmune diseases are often accompanied by excessive macrophage activation and proinflammatory cytokine release. Thus, a dysregulated cytokine network has been considered a principal factor driving these diseases.^[^ ^3^ ^]^ However, understanding the pathogenesis of autoimmune diseases remains challenging due to the intricate immune networks involving multiple signaling pathways and proteins that are difficult to tackle.^[^ ^4^ ^]^ Furthermore, while targeting cytokines and their receptors offers an approach to treating immune‐mediated inflammatory diseases, inhibiting these targets can lead to undesirable side effects because many cytokines and receptors have pleiotropic effects.^[^ ^5^ ^]^ Therefore, there is an urgent need to develop novel therapeutic strategies that target key molecular nodes in the cytokine signaling network. One promising approach is to target epigenetic regulators that control cytokine gene expression. Epigenetic modifications, such as histone acetylation and deacetylation, are critical in regulating gene transcription. By modulating the activity of epigenetic enzymes, it may be possible to fine‐tune cytokine production and restore immune balance.^[^ ^6^ ^]^

Histone deacetylases (HDACs) function as epigenetic enzymes, catalyzing the hydrolysis of *N*‐ε‐acetyl modifications from lysine residues on both histone and nonhistone proteins. Through this deacetylation activity, HDACs can regulate the gene transcription that is critical for immune cell differentiation, activation, and metabolism.^[^ ^7^ ^]^ To date, eleven zinc‐dependent HDACs have been identified and categorized into four subclasses: class I (HDAC1, 2, 3, 8), class IIa (HDAC4, 5, 7, 9), class IIb (HDAC6, 10), and class IV (HDAC11). Literature and our previous studies have shown that HDACs are crucial regulators of inflammation and immunity.^[^ ^8^ ^]^ Class IIa HDACs are notable for their substantially weak deacetylase activity, attributed to a mutation of the active tyrosine to histidine within their catalytic domain.^[^ ^9^ ^]^ Unlike class I HDACs, which are primarily localized in the nucleus, class IIa HDACs shuttle between the nucleus and cytoplasm, engaging in various cellular activities in addition to chromatin modification. Specifically, class IIa HDACs have large noncatalytic regions and may act as epigenetic readers or pseudo enzymes to interact with diverse cellular proteins.^[^ ^10^ ^]^ Dysregulation or overexpression of class IIa HDACs has been implicated in many diseases, including metabolic disorders, inflammation, and cancer.^[^ ^11^ ^]^ Despite significant advancements, the study of specific class IIa HDAC isoforms faces challenges due to the pan‐inhibitory effects of current inhibitors and the limited probes for investigating their biological functions beyond deacetylation.^[^ ^12^ ^]^ For instance, HDAC7, a class IIa HDAC, is upregulated in inflammatory macrophages and plays critical roles in cell metabolism and inflammation. However, its deacetylase‐independent functions in regulating inflammatory responses remain poorly understood.

Recently, proteolysis targeting chimera (PROTAC) has emerged as a novel approach for disrupting protein functions by harnessing the cell's endogenous proteasomal degradation machinery.^[^ ^13^ ^]^ Compared to traditional enzyme inhibition, PROTAC‐mediated protein degradation holds several attractive properties, including durable efficacy, enhanced selectivity, and the ability to target both enzymatic and nonenzymatic activities. As a result, the PROTAC approach has shown considerable promise in studying those previously challenging targets.^[^ ^14^ ^]^ Fischer and colleagues have further advanced this field by using a chemoproteomics approach to explore HDAC degradability, providing insightful guidance for developing PROTACs targeting class IIa HDACs.^[^ ^15^ ^]^

In this study, we uncovered a previously unexplored, deacetylase‐independent proinflammatory role of HDAC7 and reported the first in vivo efficacious HDAC7‐specific degrader **B4**. The PROTAC compound **B4** effectively induces HDAC7 degradation and significantly reduces the secretion of multiple proinflammatory cytokines in both lipopolysaccharide (LPS)‐stimulated macrophages and a mouse model. Using **B4** as a molecular probe, we dissected the nonenzymatic function of HDAC7 in maintaining the TNF receptor‐associated factor 6 and TGFβ‐activated kinase 1 (TRAF6‐TAK1) complex and regulating cytokine gene transcription. Our research highlights HDAC7's essential role in the Toll‐like receptor 4 (TLR4) signaling pathway and proposes targeting HDAC7 with isoform‐selective degraders as a potential therapeutic approach for treating cytokine‐mediated autoinflammatory diseases.

## 2 Result

### 2.1 HDAC7 Knockdown Significantly Attenuates Proinflammatory Cytokine Production in LPS‐stimulated Macrophages

**Figure 1:**
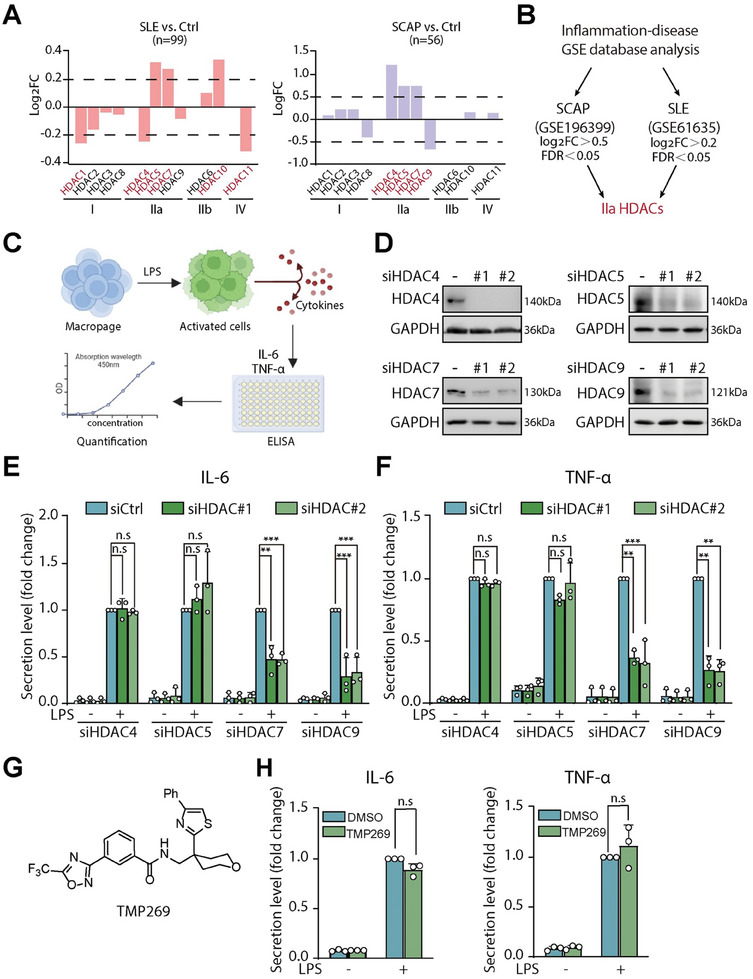
HDAC7 knockdown significantly attenuates proinflammatory cytokine production in LPS‐stimulated macrophages. A) Differential expression of the HDAC gene family in association with SLE (left) and SCAP (right). Transcriptome data for SLE (GSE616352, *n* = 99) and SCAP (GSE1963993, *n* = 56) were obtained from the Gene Expression Omnibus (GEO) database, applying a significance threshold of *p* < 0.05 and an absolute log fold change (|log_2_FC|) greater than 0.2 for the SLE dataset and 0.5 for the SCAP dataset. B) Comparative analysis of HDAC gene expression across the two datasets indicates that significantly changed genes belong to class IIa HDACs. C) Illustration depicting the secretion of cytokines (TNF‐α, IL‐6) by macrophages following LPS stimulation and subsequent measurement via ELISA. D) The effective knockdown of class IIa HDAC isoforms in RAW264.7 cells was validated by immunoblotting. E,F) Measurement and analysis of IL‐6 (E) and TNF‐α (F) levels in RAW264.7 cells after class IIa HDAC knockdown, using an ELISA assay. G) Chemical structure of the class IIa HDAC inhibitor **TMP269**. H) Quantification of IL‐6 and TNF‐α secretion levels in RAW264.7 cells following **TMP269** treatment, determined using ELISA kits. All experimental results are based on at least three biological replicates, with statistical significances denoted as not significant (n.s.), ^**^ for *p* < 0.01, and ^***^ for *p* < 0.001.

Differential gene expression analysis is widely used to investigate how gene expression changes correlate with a disease.^[^ ^16^ ^]^ To identify the key members involved in elevating proinflammatory cytokines, we analyzed the differential expression of the HDAC family in systemic lupus erythematosus (SLE) and severe community‐acquired pneumonia (SCAP), two common autoimmune diseases accompanied by a hyperactive immune system and excessive proinflammatory cytokine secretion.^[^ ^17^ ^]^ As shown in **Figure** **1**A, in the SLE dataset (GSE61635), with the cutoff at False Discovery Rate (FDR) < 0.05 and |log_2_(FC)| > 0.2, HDACs 1, 4, and 11 were found down‐regulated, while HDACs 5, 7 and 10 were significantly upregulated. Meanwhile, in the SCAP dataset (GSE196399), HDACs 4, 5, and 7 were found to be upregulated, while HDAC9 was downregulated by using cutoffs at FDR <0.05 and |log_2_(FC)| > 0.5. Notably, the genes most significantly changed in both datasets belong to the class IIa subfamily, highlighting their potential roles in promoting autoimmune diseases (Figure 1B).

Building on these observations, we investigated the direct influence of class IIa HDACs on cytokine production using the small interfering RNA (siRNA) silencing approach in a well‐characterized LPS‐induced macrophage activation model.^[^ ^18^ ^]^ We individually knocked down each isoform of class IIa HDACs with two specific siRNAs (#1 and #2) in the RAW264.7 cells and quantified tumor necrosis factor‐alpha (TNF‐α) and interleukin‐6 (IL‐6), two potent proinflammatory cytokines, via enzyme‐linked immunosorbent assays （ELISA） (Figure 1C,D). Our results demonstrated that the HDAC4 or HDAC5 silencing had minimal effect on the cytokine levels, whereas the knockdown of HDAC7 and HDAC9 markedly attenuated both IL‐6 and TNF‐α production (Figure 1E,F). This observation implicates these specific isoforms in proinflammatory pathways. Further exploration assessed the ability of a highly potent pan‐class IIa HDAC inhibitor **TMP269** (Figure 1G) to modulate cytokine secretion in LPS‐stimulated macrophages.^[^ ^19^ ^]^ Despite demonstrating strong inhibitory activity in vitro (Figure S2, Supporting Information), the effect of **TMP269** on IL‐6 and TNF‐α levels was minimal (Figure 1H). This discrepancy observed between siRNA silencing and enzymatic inhibition suggests that class IIa HDACs such as HDAC7 and HDAC9 might regulate cytokine production through mechanisms independent of their deacetylase activity, underscoring the necessity of developing novel tools to dissect these functions. Considering HDAC9's essential physiological functions and its involvement in tumor progression and cardiac hypertrophy,^[^ ^20^ ^]^ specifically targeting HDAC7 without adversely impacting HDAC9's beneficial physiological functions presents a more favorable therapeutic approach for alleviating inflammation associated with excessive IL‐6 and TNF‐α production.

Unlike traditional inhibitors that block the active site to inhibit enzymatic actions, PROTAC‐based degraders utilize a unique mechanism that removes a target protein's enzymatic and nonenzymatic functions. Moreover, studies have demonstrated that PROTACs with promiscuous warheads are capable of targeting a specific isoform over others with remarkable specificity.^[^ ^21^ ^]^ Thus, developing HDAC7 selective PROTACs could provide valuable tools for investigating HDAC7's involvement in cytokine secretion pathways and addressing its proinflammatory activities with a focus on both efficacy and safety.

### 2.2 Rational Design and Development of HDAC7 Selective Protein Degraders

**Figure 2:**
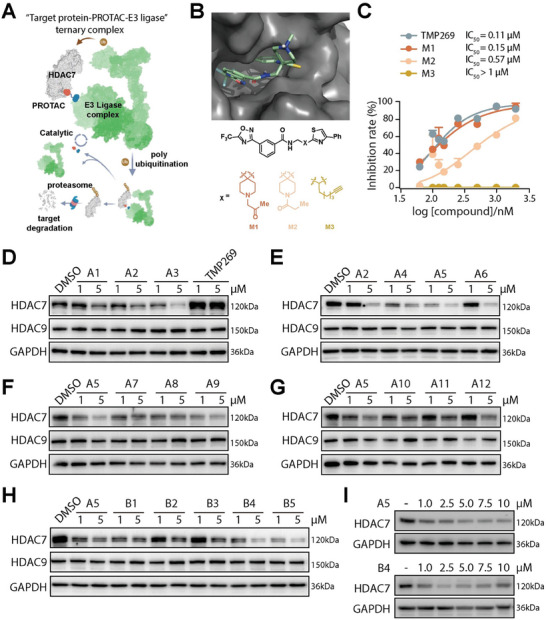
Design and optimization of selective HDAC7 degraders. A) Schematic representation of the mechanism underlying PROTAC‐induced protein degradation. B) Computational modeling displaying the binding mode of **TMP269** to the crystal structure of HDAC7 (PDB code: 3ZNS) using PyMOL software and the chemical structures of **M1**, **M2**, and **M3**. C) Assessment of in vitro HDAC7 enzymatic activity inhibition when treating with tested compounds at various concentrations and using Ac‐Leu‐Gly‐Lys(TFAc)‐AMC (TFAc: trifluoroacetyl; AMC: 7‐amino‐4‐methylcoumarin) as a substrate. D‐H) Immunoblot analysis of HDAC7 and HDAC9 in 293T cells upon treatment with different PROTAC compounds and **TMP269** (1 and 5 µm, 24 h). I) Immunoblotting results illustrating the impact on HDAC7 levels in 293T cells treated with **A5** (top) and **B4** (bottom) at varying concentrations for 12 h.

PROTAC molecules structurally consist of a warhead that interacts with the protein of interest (POI) and an E3 ligase ligand linked together by a linker. This heterobifunctional structure enables the formation of a ternary complex involving the POI, the PROTAC, and the E3 ligase, leading to the protein's polyubiquitination and subsequent proteasomal degradation (**Figure** **2**A). In our PROTAC design, we utilized the core structure of **TMP269** as the warhead, known for its high‐affinity interaction with HDAC7. Molecular docking‐based structural analysis reveals that the tetrahydropyran moiety is solvent‐exposed and amenable to linker attachment (Figure 2B).^[^ ^19^ ^]^ Replacing the tetrahydropyran in **TMP269** by a linker‐incorporated piperidine generated two model compounds **M1** and **M2**. Both compounds demonstrated notable in vitro inhibition of HDAC7 (IC_50_s = 0.15 and 0.57 µm), similar to **TMP269** (IC_50_ = 0.11 µm), confirming the viability of incorporating a piperidine‐based linker for effective binding. Conversely, substitution with a more flexible n‐hexynyl linker resulted in a decreased inhibitory performance (**M3**, IC_50_ > 1.0 µm), underscoring the importance of structural rigidity for enhancing its binding affinity to HDAC7. **M1** emerged more potent than **M2**, likely due to increased flexibility at the linker attachment point, allowing for optimal linker orientation. The structure‐activity relationship (SAR) study of the model compounds emphasizes the critical balance between flexibility and rigidity of the solvent‐exposed linkage moiety to ensure effective target interaction.

Previous studies have demonstrated that the efficacy of PROTACs in degrading target protein depends more on their ability to facilitate the formation of the ternary complex than on their affinity for the target itself. Therefore, achieving specificity in degrading HDAC7 over other class IIa HDACs is possible through careful selection of linkers and E3 ligase‐recruiting ligands.^[^ ^22^ ^]^ Choosing **M1** as our warhead, we explored how different E3 ligases and linkers affect degradation selectivity toward HDAC7. In this study, we employed reported ligands of Cereblon (CRBN) (**E1‐E4**) and von Hippel‐Lindau disease tumor suppressor (VHL) (**E5**), combined with commonly used chemical linkers, to create a focused library of PROTACs (**Table** **1**). Our teams have recently developed a synthetic method to synthesize pre‐assembled terminal azide‐labeled linker‐E3 ligand conjugates known as “preTACs,” serving as ready‐to‐assemble building blocks for PROTAC synthesis.^[^ ^23^ ^]^ By coupling the alkyne‐modified **M1** to a variety of 4‐substituted thalidomide‐based preTACs via click chemistry, we quickly generated a series of triazole‐containing PROTACs **A1**‐**A6** (Table 1), which were then assessed in 293T cells through immunoblot analysis. Compared with **A1** and **A3**, PROTAC **A5** stood out for its superior degradation efficiency and selectivity toward HDAC7, underscoring the impact of linker length on the PROTACs’ effectiveness (Figure 2D,E). Furthermore, we observed notable differences in degradation efficiency with variations in the triazole placement on the linkers (**A5** vs **A2**, **A4**, and **A6**) and the linker composition (**A5** vs **A7**, **A8**, and **A9**), emphasizing the critical role of the triazole moiety in enhancing **A5**’s cellular activity (Figure 2E,F). Exploring alternative CRBN ligands, including the C‐5 modified thalidomide (**E2**), lenalidomide (**E**3), and phenyl‐glutarimide (**E4**), led to the synthesis of PROTACs **A10**, **A11**, and **A12**. Despite these modifications, none outperformed **A5** in degrading HDAC7, confirming it as the most effective CRBN‐based HDAC7 degrader synthesized in this study (Figure 2G).

**Table 1:** PROTAC compounds synthesized in this study.

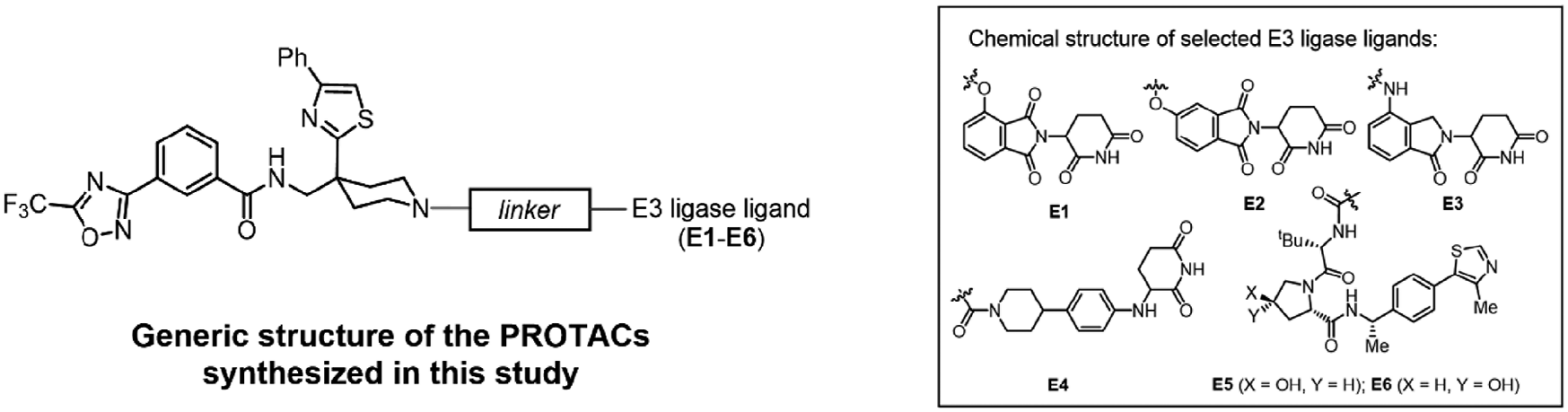					
PROTAC	Structure of linker	E3 ligase ligand	PROTAC	Structure of linker	E3 ligase ligand
A1	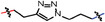	E1	A10	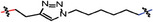	E2
A2	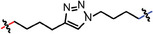	E1	A11	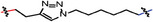	E3
A3	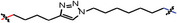	E1	A12	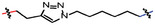	E4
A4	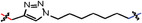	E1	B1	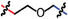	E5
A5	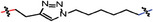	E1	B2	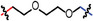	E5
A6	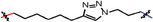	E1	B3	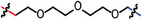	E5
A7	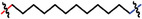	E1	B4	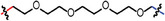	E5
A8	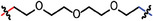	E1	B5	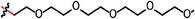	E5
A9	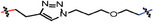	E1	B6	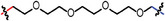	E6

Synthetic procedures and characterization data are provided in Supporting Information.

To assess the influence of E3 ligases on PROTAC activity and specificity, VHL‐recruiting PROTACs **B1**‐**B5** were synthesized by linking a warhead with the VHL ligand **E5** using various polyethylene glycol linkers (Table 1). Additionally, PROTAC **B6** served as a negative control, designed to block VHL recruitment through the epimerization of a chiral hydroxyl group in the E3 ligand. Subsequent degradation activity assessments established a clear structure‐activity relationship, identifying **B4** and **B5** as potent and selective degraders of HDAC7 (Figure 2H). Further exploration of SARs through the PROTACs’ in vitro inhibition against the class IIa HDAC family revealed that PROTACs’ degradation potency did not correlate with their enzymatic activity, emphasizing the nuanced relationship between inhibitory and degradation mechanisms (Figure S3, Supporting Information). Notably, while **B4** showed no toxicity up to 20 µm, **B5** was cytotoxic in RAW264.7 cells at concentrations as low as 1 µm (Figure S4, Supporting Information). While the exact mechanism remains unclear, the cytotoxicity linked to **B5** restricts its use in immune cell studies. Moreover, dose‐dependent degradation experiments with **A5** and **B4** reinforced their potential for exploring HDAC7‐specific functions in future studies (Figure 2I).

### 2.3 A5 and B4 Induce Isoform‐Selective, UPS‐Dependent Degradation of HDAC7

**Figure 3:**
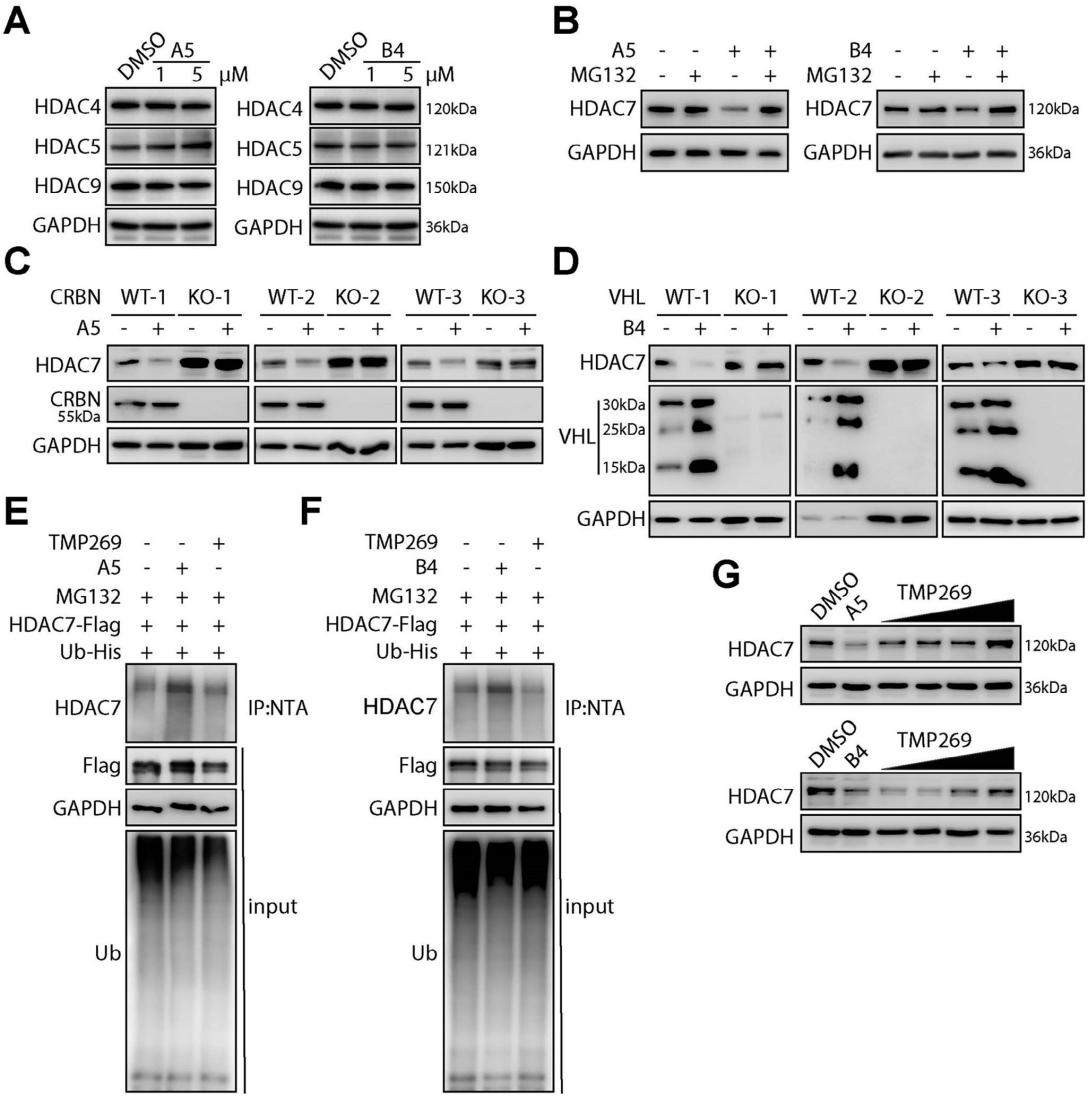
**A5** and **B4** induce isoform‐selective, UPS‐dependent HDAC7 degradation. A) Assessment of HDAC4, HDAC5, and HDAC9 protein levels in 293T cells following treatment with **A5** (left) and **B4** (right) at concentrations of 1 and 5 µm for 12 h. B) Immunoblot analysis of HDAC7 levels in 293T cells treated with **MG132** (10 µm) along with either **A5** (5 µm, top) or **B4** (5 µm, bottom) for 12 h. C) Analysis of HDAC7 protein levels in *CRBN*‐WT and *CRBN*‐KO cells treated with **A5** (5 µm) for 12 h. D) Analysis of HDAC7 protein levels in *VHL*‐WT and *VHL*‐KO cells treated with **B4** (5 µm) for 12 h. E, F) Validation of **A5** or **B**4‐induced HDAC7 ubiquitination. Transient co‐transfection of 293T cells with HDAC7‐Flag and Ub‐His plasmids (1:1 ratio) followed by treatment with **A5**/**B4** (5 µm), **MG132** (10 µm), and **TMP269** (5 µm) for 12 h. Co‐immunoprecipitation using anti‐NTA beads performed post‐transfection and treatment. G) Immunoblot analysis of HDAC7 in 293T cells after co‐treatment with various concentrations of **TMP269** (25, 50, 100, and 150 µM) and **A5** (5 µM, top) or **B4** (5 µm, bottom) for 12 h.

Upon identifying **A5** and **B4** as effective HDAC7 degraders, we proceeded to assess their selectivity among various HDAC isoforms. First, both compounds consistently showed a preference for targeting HDAC7 over any other class IIa HDACs (**Figure** **3**A), as well as the broader HDAC family (Figure S5A, Supporting Information), underscoring their excellent specificity. Furthermore, we observed that **A5** and **B4** could reduce HDAC7 protein levels without affecting its mRNA expression (Figure 3B; Figure S5B, Supporting Information). To verify the underlying mechanism of this observed protein degradation, we conducted a series of control experiments in 293T cells. First, the proteasome inhibitor **MG132** effectively blocked the degradation of HDAC7 by **A5** and **B4** (Figure 3B). Second, to confirm the dependency of the degradation on specific E3 ligases, we utilized the CRISPR‐Cas9 technique to generate *CRBN‐KO* and *VHL‐KO* cell lines. The removal of CRBN or VHL significantly reduced the degradation efficacy of **A5** and **B4** (Figure 3C,D), whereas the reintroduction of CRBN or VHL restored the degrading activity of both PROTACs (Figure S6, Supporting Information). Additionally, the co‐treatment of **A5** or **B4** with **MG132** led to an increase in ubiquitinated HDAC7 levels, while treatment with **TMP269** and **MG132** did not produce the same effect, ruling out degradation through exclusively warhead‐protein interaction (Figure 3E,F). Increasing doses of **TMP269** counteracted the HDAC7 reduction induced by PROTACs, suggesting that the PROTAC's activity is partially attributed to direct engagement with HDAC7 (Figure 3G). These mechanistic findings collectively verify that **A5** and **B4** induce selective HDAC7 degradation by orchestrating an interaction between HDAC7 and an E3 ligase via the ubiquitin‐proteasome system (UPS)‐dependent pathway, thus highlighting their potential utility in studying and manipulating HDAC7 function in cellular contexts.

### 2.4 HDAC7 Degraders Efficiently Reduce Proinflammatory Cytokine Release in LPS‐stimulated Macrophages

**Figure 4:**
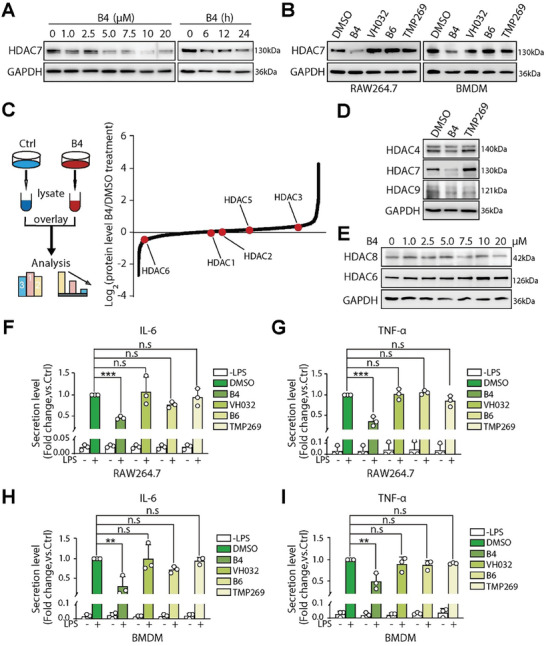
**B4**‐mediated HDAC7 degradation reduces inflammatory cytokine levels in LPS‐stimulated macrophages. A) Immunoblot analysis of HDAC7 protein levels in RAW264.7 cells after treatment with varying concentrations of **B4** for 12 h (left) and with 5 µm **B4** at different time points (right). B) Immunoblot assessment of HDAC7 protein levels in RAW264.7 and BMDM cells following treatment with **B4** (5 µm), **VH032** (5 µm), **B6** (5 µm), and **TMP269** (5 µm). C) Overview of the proteomic analysis workflow (left), and the analysis results illustrating changes in HDAC protein abundances in RAW264.7 cells after **B4** treatment displayed on a log_2_FC scale (right). HDAC7 was undetected in this experiment, likely due to its low expression levels. D) Immunoblot analysis of HDAC4, HDAC7, and HDAC9 in RAW264.7 cells after treatment with **B4** (5 µm) and **TMP269** (5 µm). E) Immunoblot analysis of HDAC6 and HDAC8 in RAW264.7 cells after treatment with various concentrations of **B4** up to 20 µm. F‐I) ELISA analysis of IL‐6 and TNF‐α secretion levels in RAW264.7 and BMDM cells. Cells were treated with tested compounds (5 µm) for 12 h, followed by LPS stimulation at 10 ng mL^−1^ for 24 h. All data presented are based on a minimum of three replicates, with statistical significance denoted as follows: not significant (n.s.), ** for *p* < 0.01, and ^***^for *p* < 0.001.

We next examined the impact of PROTAC‐mediated HDAC7 degradation on murine macrophage RAW264.7 cells and primary bone marrow‐derived macrophages (BMDM), specifically its effect on the inflammatory response within these immune cells. Immunoblot analyses showed that **B4** induced a dose‐ and time‐dependent degradation of HDAC7 in RAW264.7 cells, with a half‐maximal degradation concentration (DC_50_) of ≈5 µm (**Figure** **4**A). When comparing HDAC7 expression in RAW264.7 and BMDM cells after treatment with various compounds, including **B4**, the VHL ligand (**VH032**), a negative control PROTAC (**B6**), or an enzymatic inhibitor (**TMP269**), we found that only **B4** substantially decreased HDAC7 protein levels in both types of macrophages (Figure 4B). Additionally, quantitative proteomics and immunoblot analyses of **B4**‐treated RAW264.7 cells confirmed the excellent selectivity of **B4** toward HDAC7 within the HDAC family (Figure 4C,D; Tables S6 and S7, Supporting Information). Given that **TMP269** has been reported to effectively inhibit HDAC8 (class I) and HDAC6 (class IIb) with in vitro IC_50_ values below 10 µm,^[^ ^19^ ^]^ we further evaluated the specificity of **B4** at elevated concentrations. The results depicted in Figure 4E indicate that treatment with **B4** does not significantly impact HDAC6 protein levels at any tested concentrations. However, only a modest reduction in HDAC8 levels was observed at the highest concentration of 20 µm. Considering the DC_50_ of **B4** against HDAC7 is 5 µm, the relatively weak inhibition of HDAC8 by **B4** should not impede its application as an HDAC7‐selective probe in the subsequent studies.

We then assessed the effects of **B4** and **A5** on crucial proinflammatory cytokines, such as TNF‐α and IL‐6, in LPS‐stimulated RAW264.7 and BMDM cells by measuring their concentrations via ELISA assays. As demonstrated in Figure 4F–I and Figure S7 (Supporting Information), **B4** and **A5** significantly reduced the production of both cytokines following LPS stimulation, whereas treatments with **TMP269**, **B6**, or **VH032** did not exhibit the same inhibitory effect. These results suggest that the observed decrease in cytokine levels is primarily linked to PROTAC‐mediated HDAC7 degradation, proposing that HDAC7 may participate in cytokine production via a deacetylase‐independent mechanism.

Given that CRBN ligands have been identified to cause non‐specific protein degradation through a “molecular glue” mechanism,^[^ ^24^ ^]^ and have been shown to decrease TNF‐α levels in activated human monocytes.^[^ ^25^ ^]^ To circumvent the possible off‐target effects associated with these ligands, we chose **B4** to further explore the roles of HDAC7 in modulating inflammatory responses in macrophages.

### 2.5 B4 Specifically Suppresses the Transcription of Certain Key Proinflammatory Cytokines

**Figure 5:**
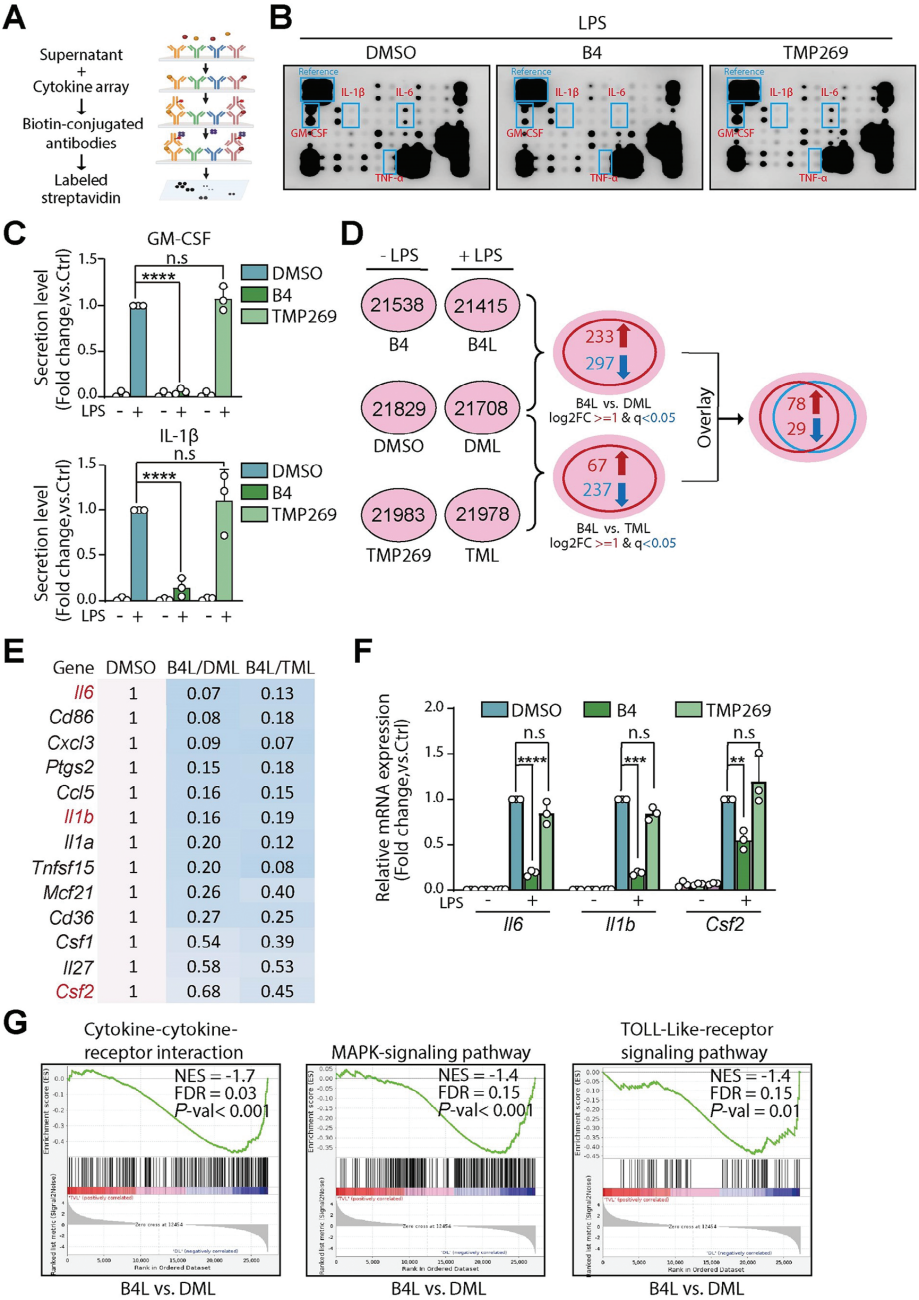
**B4** selectively suppresses the transcription of a group of proinflammatory cytokines. A) Workflow illustrating the Antibody‐array method for detecting inflammatory cytokines and chemokines. B) Analysis of inflammatory cytokines and chemokines utilizing the Mouse Inflammation Antibody Array‐Membrane Kit. RAW264.7 cells were treated with **B4** (5 µm) and **TMP269** (5 µm) for 12 h, followed by exposure to LPS (10 ng mL^−1^) for an additional 24 h. C) ELISA measurements of GM‐CSF (top) and IL‐1β (bottom) secretion levels. RAW264.7 cells were treated with **B4** (5 µm) and **TMP269** (5 µm) for 12 h, followed by LPS stimulation at 10 ng mL^−1^ for another 24 h. D) Venn diagram depicting differentially expressed genes in RAW264.7 cells. Cells were treated with DMSO, **B4**, and **TMP269** for 12 h, followed by LPS stimulation at 10 ng mL^−1^ for 24 h. Differential gene identification criteria were set at FC ≥ 2 or FC ≤0.5 (equivalent to |log_2_(FC)| ≥ 1) with a *p*‐value < 0.05 for screening differential genes. E) Analysis of inflammation‐related genes in the **B4L** group compared to **DML** and **TML** groups. F) qRT‐PCR analysis of *Il6*, *Il1b*, and *Csf2* mRNA expression levels. Data were obtained from three independent experiments. Statistical significance is indicated as follows: not significant (n.s.), ^**^ for *p* < 0.01, ^***^ for *p* < 0.001, and ^****^ for *p* < 0.0001. G) GSEA algorithm analysis of inflammation response processes in RAW264.7 cells.

Building on our prior findings, we sought to identify the specific cytokines regulated by HDAC7 and to differentiate the biological effects arising from HDAC7 degradation versus its inhibition in macrophages. To this end, RAW264.7 cells were treated with DMSO, **TMP269**, and **B4**, followed by LPS stimulation, and the cytokine secretion profile was then assessed using a semi‐quantitative Mouse Inflammation Antibody Array‐Membrane Kit, designed to detect 40 inflammatory markers (**Figure** **5**A). The results, illustrated in Figure 5B, highlighted a notable reduction in several key proinflammatory cytokines, including TNF‐α, GM‐CSF, IL‐1β, and IL‐6, specifically following **B4** treatment, in contrast to those observed with DMSO and **TMP269** treatments. This significant reduction was further confirmed through ELISA for IL‐1β and GM‐CSF in LPS‐stimulated RAW264.7 cells, highlighting **B4**’s superior efficacy compared to **TMP269** (Figure 5C).

To delve deeper into the effects of **B4**‐induced HDAC7 depletion on cellular transcriptional activity, transcriptome sequencing was conducted on RAW264.7 cells. Differential gene analysis was applied with criteria set at a fold change threshold of ≥2 or ≤0.5 and a *p*‐value < 0.05 across six experimental groups. The comparative analysis identified 233 genes upregulated and 297 downregulated in the group treated with **B4** and stimulated with LPS (referred to as **B4L**) compared to the group treated with DMSO and similarly stimulated with LPS (**DML**). A closer comparison between **B4L** and the **TMP269**‐treated, LPS‐stimulated group (**TML**) identified 67 upregulated and 237 downregulated genes. An overlay analysis spotlighted 107 genes, with 78 showing upregulation and 29 downregulation (Figure 5D). Notably, the genes downregulated in **B4L** group, including *Il6*, *Il1b*, and *Csf2*, are linked to proinflammatory signaling, which aligns with the cytokine profiling results (Figure 5E). This was further corroborated by real‐time PCR (RT‐qPCR) analysis, confirming decreased mRNA levels of *Il6*, *Il1b*, and *Csf2* following **B4** treatment, thereby demonstrating its superior capability to suppress the expression of proinflammatory cytokines over the inhibitor **TMP269** (Figure 5F).

Furthermore, we conducted Gene Set Enrichment Analysis (GSEA) to uncover the biological pathways affected by genes altered due to HDAC7 degradation. According to Figure 5G, **B4** significantly reduced the expression levels of genes implicated in critical inflammatory pathways, such as cytokine‐cytokine receptor interactions, the mitogen‐activated protein kinase (MAPK) signaling pathway, and TOLL‐like receptor (TLR) signaling. These findings further highlight HDAC7's crucial role in immune response regulation, specifically through the transcriptional control of proinflammatory cytokines via a mechanism that had not been fully elucidated before.

### 2.6 HDAC7‐Modulated Cytokine Gene Transcription is Independent of Chromatin Opening

**Figure 6:**
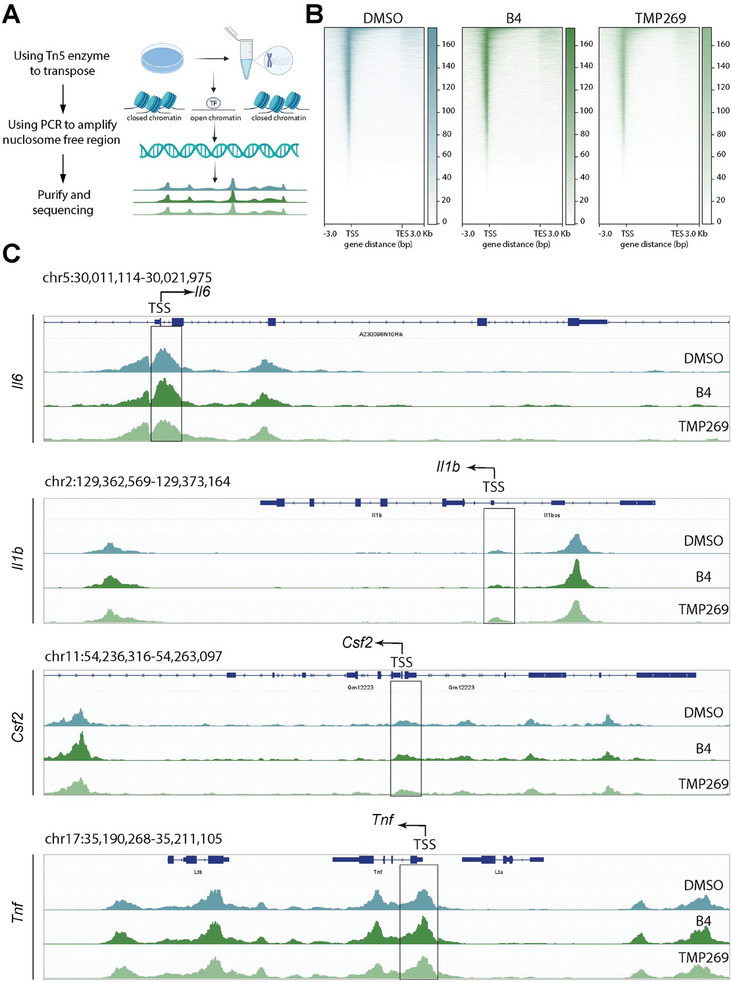
The regulatory role of HDAC7 in macrophage cytokine transcription is independent of chromatin opening. A) Graphic representation of the ATAC‐seq process. B) Enrichment of ATAC‐seq signals around TSSs after treatment with various compounds in RAW264.7 cells for 12 h. C) ATAC‐seq signal profiles in representative regions displaying consistent accessibility status across three groups. The chromatin coordinates of each region are indicated at the top left of each plot, with the TSS locations and transcription directions of target genes (*Il6*, *Il1b*, *Csf2*, and *Tnf*) denoted by black arrows.

Additional studies were undertaken to understand how HDAC7 affects cytokine gene transcription in macrophages, specifically examining whether this process is linked to changes in chromatin accessibility. HDAC7 has been implicated in regulating gene expression as an acetyl lysine reader, hinting at a potential mechanism for repressing cytokine gene transcription.^[^ ^10b^ ^]^ To investigate this, we employed a high‐throughput Assay for Transposase‐Accessible Chromatin using sequencing (ATAC‐seq) to perform a genome‐wide analysis of chromatin accessibility in RAW264.7 cells treated with **B4** and **TMP269** (**Figure** **6**A). Quality assessment of the ATAC‐seq data through FastQC confirmed its high quality, with mapping rates exceeding 99% for samples treated with DMSO, **B4**, and **TMP269**, laying a solid foundation for further examination. The distribution of reads was consistent across all treatment conditions, showing a pronounced accumulation of ATAC‐seq reads near the transcription start sites (TSS) of genes (Figure 6B). By referencing existing Chromatin Immunoprecipitation sequencing (ChIP‐seq) data, we were able to identify the TSS positions of crucial cytokine genes (*Il6*, *Il1b*, *Csf2*, and *Tnf*) and subsequently assess chromatin accessibility in these areas following treatments with **B4** or **TMP269**. Intriguingly, our comparative analysis did not indicate significant alterations in chromatin accessibility at the TSS regions of these cytokines after treatment (Figure 6C). This observation suggests that HDAC7's modulatory effect on the transcription of cytokine genes may proceed through a pathway that does not involve altering chromatin accessibility.

### 2.7 HDAC7 Regulates Proinflammatory Cytokine Transcription via The TLR4‐TRAF6‐TAK1 Signaling Pathway

**Figure 7:**
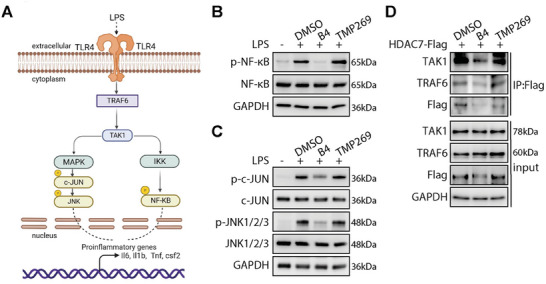
HDAC7 mediates proinflammatory cytokines transcription via the TLR4‐TRAF6‐TAK1 signaling pathway. A) Simplified illustration of the LPS‐induced TLR4 signaling pathway. B) Immunoblot analysis depicting protein levels of p‐NF‐κB(S536), NF‐κB, and GAPDH in RAW264.7 cells. Cells were pretreated with **B4** (5 µm) or **TMP269** (5 µm) for 12 h, followed by LPS stimulation for 2 h. C) Immunoblot analysis illustrating protein levels of p‐c‐JUN, c‐JUN, p‐JNK1/2/3, JNK1/2/3, and GAPDH in RAW264.7 cells. For p‐c‐JUN and c‐JUN, cells were pre‐treated with **B4** (5 µm) or **TMP269** (5 µm) for 12 h, then stimulated by LPS for 2 h; for p‐JNK1/2/3 and JNK1/2/3, cells were pretreated with **B4** (5 µm) or **TMP269** (5 µm) for 12 h, followed by LPS stimulation for 0.5 h. D) 293T cells were transfected with HDAC7‐Flag, then treated with **B4** (5 µm) or **TMP269** (5 µm) for 12 h. Cell lysates prepared using RIPA lysis buffer underwent immunoprecipitation with Flag beads, followed by immunoblotting with anti‐Flag, anti‐TAK1, and anti‐TRAF6 antibodies. Representative immunoblot data were presented.

TLR4 is known for sensing LPS and triggering a series of signaling events that lead to inflammatory responses.^[^ ^26^ ^]^ Building upon our prior GSEA results, which showed **B4**‐mediated downregulation of TLR‐related genes (Figure 5G), we next focused on the MAPK and nuclear factor‐κB (NF‐κB) pathways. These two pivotal inflammatory signaling cascades are closely associated with TLR4 signaling and are crucial in understanding HDAC7's role in inflammation. It is well established that the activation of MAPK and NF‐κB pathways, through phosphorylation processes, can drive the transcription of proinflammatory cytokines and induce inflammatory responses (**Figure** **7**A)^[^ ^27^ ^]^ To gain insights into the mechanism at play, we examined the phosphorylation status of NF‐κB and key downstream targets within the MAPK pathway following the **B4**‐induced HDAC7 depletion. Remarkably, treatment with **B4** led to a significant reduction in the levels of phosphorylated NF‐κB in RAW264.7 cells, a change not observed with **TMP269** treatment. Additionally, **B4** was found to suppress the phosphorylation of c‐JUN and JNK (c‐Jun N‐terminal kinase) effectively, whereas **TMP269** showed limited effects (Figure 7B,C).^[^ ^28^ ^]^ Delving deeper into the mechanism, we overexpressed HDAC7 in 293T cells to explore its interaction with TRAF6 and TAK1, two essential components of the TLR4 signaling pathway. The results, shown in Figure 7D, unveiled a direct interaction between HDAC7 and both TRAF6 and TAK1, which was disrupted following **B4** treatment.

These findings underscore HDAC7's critical function within the TLR4 signaling axis, specifically its role in maintaining the essential TRAF6‐TAK1 complex. This, in turn, facilitates the activation of downstream NF‐κB and MAPK pathways in LPS‐stimulated macrophages, illuminating HDAC7's involvement in the regulation of proinflammatory cytokine transcription.

### 2.8 The HDAC7 Degrader Effectively Reduced Inflammatory Cytokine Levels In Vivo

**Figure 8:**
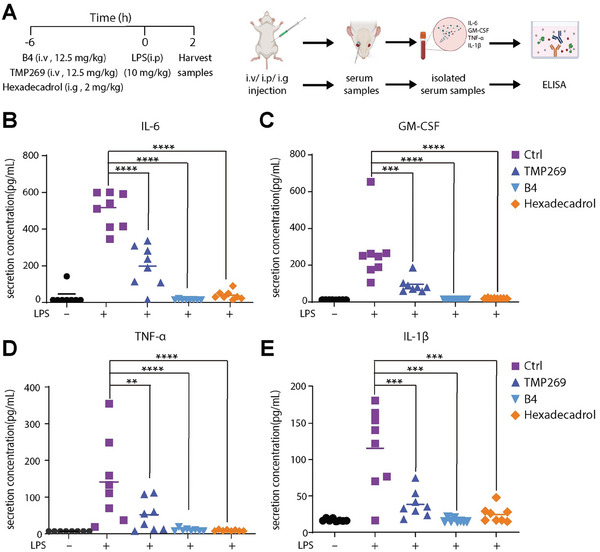
HDAC7 degrader **B4** effectively reduces inflammatory cytokine levels in vivo. A) Outline depicting the experimental procedures conducted in mice. Eight‐week‐old ICR mice in each group (*n* = 8) received pretreatment with **B4** (i.v., 12.5 mg kg^−1^), **TMP269** (i.v., 12.5 mg kg^−1^), or Hexadecadrol (i.g., 2.0 mg kg^−1^) for 6 h before LPS administration (i.p., 10 mg kg^−1^). Serum samples were collected from each group 2 h post‐LPS exposure. Note: the administrated dosages were determined through preliminary studies designed to ascertain non‐toxicity levels (Table S8, Supporting Information). B–E) Measurement of IL‐6, GM‐CSF, TNF‐α, and IL‐1β secretion levels was performed using the ELISA assay.

To evaluate the in vivo efficacy of **B4** for cytokine suppression, Institute of Cancer Research (ICR) mice were pre‐treated with an intravenous injection of **B4** (i.v., 12.5 mg kg^−1^), **TMP269** (i.v., 12.5 mg kg^−1^), or received an intragastric administration of hexadecadrol (also known as Dexamethasone, i.g., 2.0 mg kg^−1^), which served as an FDA (U.S. Food and Drug Administration)‐approved anti‐inflammatory benchmark for this research.^[^ ^3b^ ^]^ Six hours following these treatments, acute inflammation was triggered through an intraperitoneal injection of LPS (i.p., 10 mg kg^−1^), as depicted in **Figure** **8**A. Serum samples collected 2 h after LPS administration were analyzed for cytokine levels using ELISA. The comparative assessment of the effects of these compounds on key proinflammatory cytokines (IL‐6, GM‐CSF, TNF‐α, and IL‐1β) revealed that **B4** significantly reduced the levels of these cytokines by more than 95%, demonstrating anti‐inflammatory efficacy comparable to that of Dexamethasone (Figure 8B–E). Given the comparable in vitro HDAC7 inhibition exhibited by **B4** and its structural analog **B6** (Figure S3, Supporting Information), it is intriguing to further compare their in vivo bioactivities. As shown in Figure S8 (Supporting Information), compared to the active PROTAC **B4**, the degradation‐inactive **B6** demonstrates lower potency in suppressing the secretion of various proinflammatory cytokines, reinforcing our conclusion that the anti‐inflammatory activity of **B4** predominantly depends on VHL‐mediated HDAC7 degradation rather than the inhibition induced by the warhead. These results highlight **B4**’s potential not just as a potent research tool but also as a promising lead compound for developing new therapeutic agents for cytokine‐driven diseases. Nonetheless, further medicinal chemistry efforts are necessary to enhance **B4**’s bioavailability and overall efficacy in vivo, thereby advancing its therapeutic potential.

## 3 Discussion

Recent studies have identified HDAC7 as a key regulator in cellular processes related to metabolism and inflammation.^[^ ^29^ ^]^ For instance, research by Sweet et al. pointed out the crucial role of HDAC7 in macrophages, particularly in producing TLR‐inducible inflammatory mediators in response to submaximal LPS concentrations.^[^ ^30^ ^]^ In their investigation, the pan‐class IIa HDAC inhibitor **TMP195** was shown to reduce IL‐1β levels in LPS‐stimulated macrophages. However, our study suggests that the reduction in proinflammatory cytokines, including IL‐1β, results from HDAC7 degradation rather than inhibition. These divergent observations might be due to the different LPS doses and stimulation durations used in the various studies. Furthermore, growing evidence in support of HDAC7's deacetylase‐independent functions aligns with some aspects of our findings.^[^ ^10^, ^29^ ^]^

Despite a prior report claiming the absence of HDAC7 expression at both mRNA and protein levels in RAW264.7 cells,^[^ ^31^ ^]^ our current study clearly demonstrates HDAC7 expression via immunoblotting, albeit at lower levels. This discrepancy could be due to the diversity of experimental approaches across studies, including the choice of antibodies for HDAC7 detection and the control cell lines used. Notably, our immunoblot results reveal a significant reduction in HDAC7 protein levels following the application of two specific siRNAs (Figure 1D). This evidence, along with the HDAC7 degradation induced by **A5** and **B4**, confirms HDAC7's presence in RAW264.7 cells, which is also supported and explored by other research groups in various publications.^[^ ^8^, ^32^ ^]^

**B4** emerged as the most potent HDAC7 degrader in this study, possibly because of its unique linker configuration that facilitates stable ternary complex formation. To provide direct evidence of **B4**‐triggered ternary complex formation, we conducted pull‐down assays that confirmed the interaction between HDAC7 and VHL to form a ternary complex in the presence of **B4**. In contrast, no HDAC9‐**B4**‐VHL complex formation was observed under any concentration of VHL and **B4** used in our experiments (Figure S9, Supporting Information). These results not only corroborate the existence of the ternary complex but also shed light on the mechanism supporting **B4**’s selectivity toward HDAC7.

The excessive production of proinflammatory cytokines can disrupt immune homeostasis, leading to autoimmune diseases such as rheumatoid arthritis (RA) and SLE, with TNF‐α and IL‐6 being prime targets for drug development.^[^ ^33^ ^]^ While clinical applications of monoclonal antibodies targeting these cytokines or their receptors have been successful, concerns about side effects drive the search for new therapeutic targets and strategies.^[^ ^5^, ^34^ ^]^ Our findings highlight the pivotal role of HDAC7 in mediating LPS‐induced inflammatory responses by activating downstream MAPK/NF‐κB pathways.

The PROTAC compounds we developed selectively degrade HDAC7 and significantly diminish cytokine production both in vitro and in vivo. It is noteworthy that while **TMP269** does not reduce cytokine levels in vitro, it possesses a moderate inhibitory effect in vivo. This discrepancy could be due to the complex nature of immune systems in living organisms, which comprise various immune cell types. Additionally, potential drug metabolism in vivo might lead to the hydrolysis of the key 5‐(trifluoromethyl)−1,2,4‐oxadiazole group in **TMP269**, resulting in a loss of selectivity against class IIa HDACs and production of metabolites that contribute to its in vivo activity. Given the limitations and side effects associated with pan‐class IIa HDAC inhibitors, such a learning and memory impairments related to HDAC4 inhibition and cardiotoxicity risks from HDAC5 inhibition, targeting HDAC7 with isoform‐selective degraders presents a promising path for both efficacy and safety.^[^ ^35^ ^]^

Furthermore, other HDACs have been identified in immune regulation, not relying on their deacetylase activity.^[^ ^7^, ^36^ ^]^ For instance, Dekker et al. developed an effective HDAC3 degrader **P7**, which promotes anti‐inflammatory cytokine secretion in THP‐1‐derived macrophages.^[^ ^37^ ^]^ These findings together with ours provide valuable insights into the nonenzymatic roles of HDACs in immunoregulation and encourage further exploration of their functions using isoform‐selective degraders.

In conclusion, our current work indicates that an HDAC7‐targeting PROTAC exhibits anti‐inflammatory capacity, which can be mechanistically explained by the findings presented here. Specifically, HDAC7 interacts with the TRAF6‐TAK1 complex, thereby initiating the activation of NF‐κB/MAPK pathways and promoting the transcription of proinflammatory cytokine genes. Beyond identifying an in vivo efficacious molecular probe, our findings expand the current understanding of HDAC7 as an epigenetic regulator and underscore the potential of developing isoform‐selective HDAC7 degraders as therapeutic agents for treating inflammation‐related diseases.

## 4 Experimental Section

### Chemical Synthesis

Detailed synthetic procedures and full characterization of final compounds are provided in the Supporting Information.

### Bioinformatics Analysis

Transcriptome datasets for two autoimmune diseases (SLE, GSE616352 and SCAP, GSE1963993) were downloaded from the Gene Expression Omnibus (GEO) database. Gene differential expression analyses were performed using the Limma software package, grouped by disease and control, and the data were normalized using the built‐in function normalize between arrays.

### Western Blotting Analysis

Cells and murine macrophages (RAW264.7, BMDMs) with a density of 2 × 10^5^ cells mL^−1^ were seeded in 24‐well plates and stabilized for 12 h. Then, the cells were treated with compounds for 12 h. At the endpoint of the experiment, the cells were first washed with cold phosphate‐buffered saline (PBS) and then lysed with 100 µL loading buffer containing Tris (20 mm, pH 6.8), SDS (4% w/v), glycerol (16% v/v), DTT (3% w/v), and bromophenol blue (0.02% w/v). Subsequently, the lysate was incubated at 95 °C for 30 min. The cell lysate containing equal amounts of proteins was separated by sodium dodecylsulfate‐polyacrylamide gel electrophoresis (SDS‐PAGE) and the proteins were transferred to PVDF membranes. The membranes were blocked using 5% skim milk for 1 h at room temperature and incubated with primary antibody overnight at 4 °C. After washing three times with PBS‐T, the membranes were incubated with secondary antibodies (from Ford Biotechnology) at room temperature for 1 h. The immunoreactive bands were then visualized using ECL and analyzed using the Amersham ImageQuant 800 system (cytiva, 29399481). The primary antibody used in this study is shown in Table S1 (Supporting Information).

### HDAC7 In Vitro and In Cells Kinase Assay

Full‐length human HDAC7 fused with C‐terminal His tag were recombinantly expressed in Sf9 insect cells and then were purified by His Trap HP and using ÄKTA pure system. The in vitro kinase assay was performed in assay buffer (1 m Tris‐HCl, 4 m NaCl) at 30 °C using 0.1 µm purified HDAC7 enzyme and 50 µm Ac‐ Leu‐Gly‐Lys (TFAc)‐AMC substrate. For the in‐cell kinase assay, RAW264.7 cells were treated with 5 µm **TMP269** for 12 h and lysed with RIPA buffer (25 mm Tris‐HCl, 150 mm NaCl, 1% NP‐40, 1% sodium deoxycholate, 0.1% SDS, pH 7.4). Subsequently, 5 µm **TMP269** was added to the cell lysate during the enzyme reaction. The kinase activity of HDAC7 was represented by the fluorescence of 7‐amino‐4‐methylcoumarin released by 0.5 µg mL^−1^ Trypsin.

### Real‐Time Quantitative PCR

Total mRNA was extracted using the PureLink RNA extraction kit (Thermo Fisher, 12183018A). A total of 1 µg of mRNA was then reverse‐transcribed into cDNA using the cDNA synthesis kit (Transgen, AT311‐03). The quantitative Real‐Time PCR Analysis System (Roche) was used for qRT‐PCR, utilizing the iTaq Universal SYBR Green qPCR supermixes (Bio‐Rad, L001752B). The relative expression values for each gene of interest were determined by normalizing them to *GAPDH* mRNA expression. The specific primer pairs for each gene are listed in Table S2 (Supporting Information).

### ELISA Assay for Cytokine Quantification

RAW264.7 and BMDM cells were seeded in 24‐well plates at a density of 2 × 10^5^ cells mL^−1^ and incubated for 12 h. After being treated and stimulated with the tested compounds and LPS, as indicated in individual figure legends, the supernatant was collected for measuring the level of cytokines by using commercial ELISA kits (BIOKER). First, the ELISA plate is coated with capture antibody in a Coating Buffer and sealed. It is then incubated overnight at 4 °C. Next, the wells were blocked using 200 µL of ELISA/ELISPOT Diluent and incubated at room temperature for 2 h. After incubation, set up the standard curve and add 100 µL well^−1^ of samples. Incubate them for 2 h at room temperature or overnight at 4 °C. Next, add the diluted Detection Antibody to each well and incubate the plate at room temperature for 1 h. After that, Streptavidin‐HRP was added at a volume of 100 µL well^−1^, and the plate was incubated at room temperature for 30 min. Then, the plate was incubated with TMB solution at room temperature for 15 min. Finally, the reaction was stopped by adding 1 m H_2_SO_4_. The absorbance was measured at 450 nm (reference wavelength: 570 nm) using a microplate reader (TECAN, 2102004761).

### Cytokines Array Analysis

The cytokines secreted by RAW264.7 cells were analyzed using the Mouse Inflammation Antibody Array‐Membrane Kit (Abcam, ab133999) according to the manufacturer's instructions. In brief, block the Antibody‐Array membranes by incubating them with 2 mL Blocking buffer at room temperature for 30 min. Then, incubate the membranes with the cell culture supernatant‐antibody samples overnight at 4 °C. The array membranes were washed with wash buffer I and wash buffer II provided in the kit and then incubated with Biotin‐Conjugated Anti‐Cytokines for 2 h at room temperature. Next, pipette HRP‐Conjugated into each well and incubate for 2 h at room temperature. Finally, detect chemiluminescence using the Amersham ImageQuant 800 system (cytiva, 29399481) after reaching the dot blots with detection buffers. The Inflammation cytokines lists are shown in Table S3 (Supporting Information).

### Pulldown of Total Ubiquitinylated Proteins with Ni‐NTA Beads

293T cells (2 × 10^6^ cells dish^−1^) were co‐transfected with the indicated plasmids (HDAC7‐Flag and His‐ubiquitin) using Jetprime transfection reagents. At 24 h post‐transfection, the media was changed, and cells were co‐treated with PROTACs or **TMP269** for 12 h. In addition, 20 µm of **MG132** was added 8 h before collecting the cells. Cells were washed with PBS and lysed with 8 m urea buffer (10 mm Tris‐HCl pH 8.0; 100 mm NaH_2_PO_4_; 8 m urea) containing 10 mm imidazole. The cell lysate was incubated with Ni‐NTA Beads (Smart‐Lifesciences, SA004005) overnight at 4 °C. The beads were then washed five times with wash buffer (10 mm Tris‐HCl pH 6.3, 100 mm NaH_2_PO_4_, 8 m urea) containing 20 mm imidazole. Protein elution was performed using 2× loading buffer (20 mm Tris‐base, 4% (w/v) SDS, 16% (w/v) glycerol, 3% (w/v) DTT, and 0.02% (w/v) bromophenol blue, pH 6.8) at 95 °C for 10 min. Finally, 20 µg of proteins were subjected to Western blotting analysis.

### Generation of CRBN/VHL‐Knockout Cell Lines Using CRISPR Cas9 Gene Editing Technology

The *CRBN*‐knockout (KO) and *VHL*‐knockout cells were generated using CRISPR‐cas9 technology. CRISPR gRNAs were designed by http://crispor.tefor.net/. The annealed sgRNA targeting *CRBN* or *VHL* were inserted into Bbs1‐digested pSpCas9(BB)−2A‐GFP (PX458) plasmid (Addgene plasmid #48138) to generate PX458‐CRBN gRNAs and PX458‐VHL gRNAs. The sgRNA sequences can be found in Table S4 (Supporting Information). To generate CRBN/VHL KO 293T cells, the wild‐type 293T cells were transfected gRNAs targeting *CRBN* or *VHL* with Jetprime transfection reagents. The flow cytometry technique was used to isolate the single cell, which was then seeded in 96‐well plates. The genomic DNA of each individual cell clone was extracted using the TIANamp Genomic DNA kit (TIANGEN Biotech, DP304‐02) and identified through gene sequencing.

### siHDACs Assay

RAW264.7 cells were seeded with a density of 5 × 10^3^ cells mL^−1^ in a 24‐well plate and cultivated for 12 h. Mix siRNA against HDAC4, 5, 7, 9 or scrambled control oligonucleotides and lipo3000 in transfection reagent (Thermo Fisher Scientific) and then transfect them to RAW264.7 cells. Cells were incubated with siRNA over a period of 48 h before incubation with LPS for another 24 h. The sequence of siRNA is shown in Table S5 (Supporting Information).

### Global Proteomics Analysis

The RAW264.7 and 293T cells were individually treated with B4 or DMSO before being rapidly frozen by liquid nitrogen. Quantitative proteomics analysis was performed by Jingjie PTM‐Biolab company (Hangzhou, China). The quantitative mass spectrometry‐based proteomics analysis for HDACs is summarized in Tables S6 and S7 (Supporting Information).

### RNA‐Sequencing Analysis

Total RNA was isolated and purified using TRIzol reagent (Invitrogen, Carlsbad, CA, USA) following the manufacturer's procedure. RNA sequencing was completed by Hangzhou Lianchuan Biotechnology Ltd. The RNA amount and purity of each sample were quantified using NanoDrop ND‐1000 (NanoDrop, Wilmington, DE, USA). The RNA integrity was assessed by Bioanalyzer 2100 (Agilent, CA, USA) with RIN number >7.0, and confirmed by electrophoresis with denaturing agarose gel. Poly (A) RNA was purified from 1 µg total RNA using Dynabeads Oligo (dT) 25–61005 (Thermo Fisher, CA, USA) through two rounds of purification. The purified poly (A) RNA was then fragmented into small pieces under 94 °C for 5–7 min using the Magnesium RNA Fragmentation Module (NEB, cat. e6150, USA). Next, the cleaved RNA fragments were reverse‐transcribed by SuperScript II Reverse Transcriptase (Invitrogen, cat. 1896649, USA) to generate cDNA. The cDNA was subsequently used to synthesize U‐labeled second‐stranded DNAs with the assistance of E. coli DNA polymerase I (NEB, cat.m0209, USA), RNase H (NEB, cat.m0297, USA), and dUTP Solution (Thermo Fisher, cat. R0133, USA). Preparing them for ligation to the indexed adapters, an A‐base is added to the blunt ends of each strand. The adapters, which contain a T‐base overhang, are then ligated to the A‐tailed fragmented DNA. Following this, the fragments are ligated to single‐ or dual‐index adapters. Size selection is performed with AMPureXP beads. The second‐stranded DNAs labeled with U are first treated with the heat‐labile UDG enzyme (NEB, cat.m0280, USA). Following this treatment, the ligated products undergo PCR amplification. The PCR conditions consist of an initial denaturation at 95 °C for 3 min. This is followed by eight cycles of denaturation at 98 °C for 15 s, annealing at 60 °C for 15 s, and extension at 72 °C for 30 s. Finally, a final extension is carried out at 72 °C for 5 min. The resulting cDNA library has an average insert size of 300 ± 50 bp. To complete the sequencing process, 2×150 bp paired‐end sequencing (PE150) on an Illumina Novaseq6000 (LC‐Biotechnology CO, Ltd., Hangzhou, China) was performed. This sequencing was conducted in accordance with the vendor's recommended protocol.

### ATAC‐Sequencing Analysis

The ATAC‐seq was conducted following the ATAC‐seq protocol by Shanghai Jiayin Biotechnology Ltd. Briefly, cells were harvested from cell culture and lysed using a lysis buffer. The transposition step was performed using the Nextera DNA Library Preparation Kit (Illumina), following the manufacturer's instructions. A total of 50 000 nuclei were pelleted and then resuspended with transposase at 37 °C for 30 min. The resulting transposed DNA fragments were promptly purified using a MinElute PCR Purification Kit (Qiagen). Samples were PCR‐amplified using NEBNext High‐Fidelity PCR Master Mix (New England Biolabs, MA). The amplified libraries were purified with the MinElute PCR Purification Kit (Qiagen) and sequenced using PE150 on the Illumina Novaseq 6000.

Raw data (raw reads) of fastq format underwent initial processing using in‐house perl scripts. In this step, reads containing adapter sequences, reads containing ploy‐N, and low‐quality reads were removed from the raw data to obtain clean data (clean reads). Simultaneously, Q20, Q30, and the GC content of the clean data were calculated. Subsequently, all subsequent analyses were conducted using high‐quality clean data. Pair‐end reads were mapped after obtaining clean reads by removing adaptor sequences from the raw reads. The clean reads were aligned to reference genome sequences using the BWA program. Visualization of the ATAC‐seq data was analyzed by Integrative Genomics Viewers (IGV).

### Co‐Immunoprecipitation

pCDH‐HDAC7‐Flag was transfected into 293T cells using Jetprime transfection reagents. After 24 h transfection, the cells were treated with PROTACs or **TMP269** for 12 h. The transfected cells were washed with ice‐cold PBS twice and lysed using RIPA lysis buffer containing (50 mm Tris‐base, 150 mm NaCl, 5 mm EDTA, 0.1% SDS, 1% TritonX‐100, 0.25% Sodium deoxycholate, pH 7.4), then centrifuged at 13 000 g for 30 min. The cell lysate was incubated with Flag beads overnight at 4 °C. The complex was washed with T‐PBS five times and suspended in the loading buffer followed by western blot analysis.

### Ternary Complex Formation Assay

The 293T cells (2 × 106 cells dish^−1^) were co‐transfected with the indicated plasmids (HDAC7‐Flag or HDAC9‐Flag) using Jetprime transfection reagents (Polyplus, 101000046). The 24 h after transfection, the cells were collected and lysed using 1% NP40 buffer (25 mm Tris‐HCl pH 8.0, 150 mm NaCl, 10% Glycerol). Subsequently, the cell lysate was either treated with concentration gradients of compounds of **B4** (1, 5 and, 10 µm) and incubated with the fixed concentration of VHL recombinant protein (1 µm) or treated with the fixed concentration of **B4** (5 µm) and incubated with concentration gradients of VHL recombinant protein (0.1, 1 and, 3 µm) for 10 min at room temperature. Then, the mixture was incubated with GST Beads (Smart‐Lifesciences, SM002005) for immunoprecipitation overnight at 4 °C. The beads were then washed five times with wash buffer (25 mm Tris‐HCl pH 8.0, 150 mm NaCl, 10% Glycerol). The proteins were eluted with 2× loading buffer (20 mm Tris‐base, 4% (w/v) SDS, 16% (w/v) glycerol, 3% (w/v) DTT, and 0.02% (w/v) bromophenol blue, pH 6.8), followed by immunoblotting with anti‐HDAC7, anti‐HDAC9, and anti‐VHL antibodies.

### VHL Recombinant Protein Expression and Purification

The pACYC‐Duet1‐Elongin B/C (#110274) and VHL‐pGex2TK (#20790) plasmids were purchased from Addgene. The two plasmids were then co‐transformed into *E. coli* BL21(DE3)‐RIPL, and positive clones were screened by LB agar plates (1% NaCl, 1% Tryptone, 0.5% Yeast Extract, 1.5% agar pH 7.0) containing 50 µg mL^−1^ Kanamycin, 100 µg mL^−1^ Carbenicillin, and 30 µg mL^−1^ Chloramphenicol antibiotics. The *E. coli* was growing at 37 °C in LB broth until the OD600 reached 0.4–0.6. Isopropyl‐β‐D‐thiogalactoside (IPTG, 0.4 mm) (V900917, Sigma‐Aldrich) was then added, and bacteria were further incubated at 16 °C for 16 h. After centrifugation at 3000 rcf for 10 min at 4 °C, the bacteria were resuspended in lysis buffer (30 mm Tris‐HCl pH 8.0, 200 mm NaCl, 5 mm DTT, 5% glycerol, 1× EDTA‐free Protease Inhibitor Cocktail). The bacteria were homogenized using a high‐pressure cell crusher (Union Bio‐tech, UX450) and centrifuged at 20 000 g for 1 h at 4 °C. After filtration with the 0.22 µm microporous filter membrane, the supernatant was loaded onto the GST affinity column (cytiva, 17528101). The protein was eluted with elution buffer (50 mm Tris‐HCl pH 8.0, 200 mm NaCl, 1 mm DTT, 10 mm glutathione). The purified protein was dialyzed with PBS (pH 8.0) buffer containing 20% glycerinum.

### In Vivo Studies

It was confirmed that the in vivo experiment in this study complies with all relevant ethical regulations. Maintenance and experimental procedures for the mice studies were approved by The Innovation Institute for Artificial Intelligence in Medicine, Zhejiang University's IACUC (DW202210131929). Preliminary experiments designed to ascertain non‐toxicity levels suggested an administration of 12.5 mg kg^−1^ of **B4** did not result in any animal death, suggesting a safe dosage for the in vivo model. In the mice study, ICR mice (8 weeks, Charles River Animal Resource, Beijing, China) were kept in ventilated cages and provided with sterilized food, water, and bedding. The mice were maintained within a temperature range of 21 to 26 °C and a humidity range of 50–70%. The mice were randomly assigned and separately administered with **B4** (i.v, 12.5 mg kg^−1^), **TMP269** (i.v, 12.5 mg kg^−1^), Hexadecadrol (i.g, 2 mg kg^−1^) for 6 h, then the mice were intraperitoneal injection with 10 mg kg^−1^ LPS to induce acute inflammation. The 2 h later, the serum samples were collected and allowed to stand at room temperature for 4 h until separation occurred. Subsequently, the samples were centrifuged at 4 000 rpm for 30 min, and the supernatant (100–300 µL) was collected. Then detect the levels of proinflammatory cytokines using commercially accessible ELISA kits (BIOKER).

### Statistical Analysis

All statistical evaluation was performed with Graphpad Prism 8.4.3 (Graphpad Software), and data were analyzed by one‐way analysis of univariate variance (ANOVA) or unpaired Student's *t*‐test. For all numerical tests, a probable value (^**^, *p *< 0.01, ^***^, *p *< 0.001, ^****^, *p *< 0.0001, ns: not significant) was considered to be statistically significant. All data are shown as the mean ± standard deviation (SD) from at least three biologically independent experiments.

## Conflict of Interest

The authors declare no conflict of interest.

## Author Contributions

K.K., T.N., and B.D. contributed equally to this work. K.K., B.C., X.Q., X.C., Y.F., and J.Z. carried out the biological experiments and analyzed the data. T.N., B.D., Z.L., and Y.Y. designed and synthesized the PROTACs. W.Z. performed the bioinformatics analysis. D.C. performed computational analysis of the ternary complex; Z.Z., X.D., B.Y., and Q.H. supervised the project. J.C., L.J., and C.‐L.Z. conceived this study, supervised experiments, analyzed the data, and wrote the manuscript with assistance from K.K.

## Supporting information

Supporting Information

Supplemental Tables 6‐7

## Data Availability

The data that support the findings of this study are available in the supplementary material of this article.
