# Supplementary material for: PROTAC‐Mediated HDAC7 Protein Degradation Unveils Its Deacetylase‐Independent Proinflammatory Function in Macrophages
Source: Adv Sci (Weinh). 2024 Jul 25;11(36):2309459. doi: 10.1002/advs.202309459 (PMC11423193; doi:10.1002/advs.202309459)
Supplement: Supplementary file 1 — Supporting Information [file ADVS-11-2309459-s001.docx]

**Supporting Information**

**PROTAC-mediated HDAC7 Protein Degradation Unveils Its Deacetylase-independent Proinflammatory Function in Macrophages**

Kailibinuer Kadier, Tian Niu, Baoli Ding, Boya Chen, Xuxin Qi, Danni Chen, Xirui Cheng, Yizheng Fang, Jiahao Zhou, Wenyi Zhao, Zeqi Liu, Yi Yuan, Zhan Zhou, Xiaowu Dong, Bo Yang, Qiaojun He, Ji Cao,* Li Jiang,* and Cheng-Liang Zhu*

K. Kadier, T. Niu, B. Ding, B. Chen, X. Qi, D. Chen, X. Cheng, Y. Fang, J. Zhou, W. Zhao, Z. Liu, Y. Yuan, Z. Zhou, X. Dong, B. Yang, Q. He, J. Cao, L. Jiang, C.-L. Zhu

Institute of Pharmacology & Toxicology, Zhejiang Province Key Laboratory of Anti-Cancer Drug Research, College of Pharmaceutical Sciences, Zhejiang University

Hangzhou 310058, P.R. China

E-mail: caoji88@zju.edu.cn; jiangli49@zju.edu.cn; chengliangzhu@zju.edu.cn

Z. Zhou, X. Dong, B. Yang, Q. He, J. Cao, L. Jiang, C.-L. Zhu

Innovation Institute for Artificial Intelligence in Medicine, Zhejiang University

Hangzhou 310018, P.R. China

Q. He, C.-L. Zhu

Center for Drug Safety Evaluation and Research of Zhejiang University

Hangzhou 310058, P.R. China

X. Dong, B. Yang, Q. He, J. Cao

Engineering Research Center of Innovative Anticancer Drugs, Ministry of Education

Hangzhou 310058, P.R. China

B. Yang, Q. He, J. Cao

Center for Medical Research and Innovation in Digestive System Tumors, Ministry of Education

Hangzhou 310058, P.R. China

X. Dong, B. Yang, Q. He, J. Cao, C.-L. Zhu

Hangzhou Institute of Innovative Medicine, Zhejiang University

Hangzhou 310018, P.R. China

X. Dong, B. Yang, Q. He, J. Cao

Cancer Center, Zhejiang University

Hangzhou 310058, P.R. China

B. Yang

School of Medicine, Hangzhou City University

Hangzhou 310015, P.R. China

1. **General Information**
2. **Synthetic Procedures and Characterization of Model Compounds and PROTACs**
3. **Methods and Protocols for Biological Studies**
4. **Supplementary Figures**
5. **NMR Spectra**
6. **HPLC and HRMS Analysis Reports**
7. **References**

**A. General Information**

**Reagents** Commercially accessible chemicals were purchased from LeYan.com, Energy-chemical, Sigma-Aldrich, TCI Chemicals, and MedChemExpress. Unless otherwise noted, all reagents were used without further purification. The reagents used in biological experiment were purchased from ChinaNational Pharmaceutical Group Corporation, Biofox, Biorad, Thermofisher, Cell signaling technology, Bethyl, Absin and Sigma-Aldrich, and all reagents were used without further purification.

**Instrumentation** Reactions were monitored via LC/MS analysis and thin layer chromatography (TLC). LC/MS was carried out on an Agilent 1290 Infinity II machine and a C18 reversed-phase column (Agilent Poroshell 120 EC-C18, 2.1 × 50 mm, 2.7 μm), with a flow rate of 0.5 mL/min, monitoring at 254 nm with positive mode for detection of mass-to-charge ratio of ions. Solvents for LC-MS were water with 0.1% formic acid (solvent A) and acetonitrile (solvent B) at a flow rate of 0.5 mL/min. TLC plates were purchased from NUOTAI Chemicals (TLC Silica gel 60 F254, 250 mm thickness). Flash column chromatography was performed over Silica gel 60 (particle size 0.04-0.063 mm) from NUOTAI Chemicals.

Proton nuclear magnetic resonance (^1^H NMR) spectra and carbon nuclear magnetic resonance (^13^C NMR) spectra were recorded on JEOL400 (400 MHz). Chemical shifts for protons are reported in parts per million downfield from tetramethylsilane and are referenced to the NMR solvent residual peak (CHCl_3_ δ 7.26, DMSO δ 2.50). Chemical shifts for carbons are reported in parts per million downfield from tetramethylsilane and are referenced to the carbon resonances of the NMR solvent (CDCl_3_ δ 77.0, DMSO δ 39.52). Data are represented as follows: chemical shift, multiplicity (br = broad, s = singlet, d = doublet, t = triplet, q = quartet, m = multiplet), coupling constants in Hertz (Hz), and integration.

Analytical HPLC analysis was carried out on an Agilent 1260 Infinity II (LC03) machine and a C18 reversed-phase column (Agilent Eclipse XDB-C18, 4.6 × 250 mm, 5 μm), with a flow rate of 1.0 mL/min, the detection by UV absorbance at a wavelength of 254 nm. Solvents for analytical HPLC were water with 0.1% formic acid (solvent A) and methanol (solvent B) at a flow rate of 1.0 mL/min (Gradient method: 0 min, 90% / 10% solvent A / solvent B; 15 min, 10% / 90% solvent A / solvent B; 25 min, 90% / 10% solvent A / solvent B)

**Supplemental Tables-data generated for the quantitative mass spectrometry-based proteomes analyses (in a separate file):** **Table S6** The quantitative mass spectrometry-based proteomes analyses for HDACs of RAW264.7 cells treated with **B4**; **Table S7** The quantitative mass spectrometry-based proteomes analyses for HDACs of 293T cells treated with **B4**.

**Abbreviations Used** MeOH–methanol, EtOH–ethanol, EtOAc–ethyl acetate, THF–tetrahydrofuran, Et_2_O–diethyl ether, CH_2_Cl_2_–dichloromethane, TEA–triethylamine, MeCN–acetonitrile, TFAA–Trifluoroacetic anhydride, TLC–thin layer chromatography, LiAlH_4_–lithium aluminum hydride, TsOH–*p*-toluenesulfonic acid, TsCl–tosyl chloride, KI–potassium iodide, DMP–Dess–Martin periodinane, DMF–*N*, *N*-dimethylformamide, EDCI–1-(3-dimethylaminopropyl)-3-ethylcarbodiimide hydrochloride, HOBt–1-Hydroxybenzotriazole hydrate, DIPEA–*N*, *N*-diisopropylethylamine, Et_3_N−triethylamine, DMAP−4-dimethylaminopyridine, *t*BuOH–*tert-*butanol, VitCNa–L-ascorbic acid sodium salt.

**B．Synthetic Procedures and Characterization of Model Compounds and PROTACs**

1. **Synthesis of the key intermediate S7**

PROTAC compounds in the manuscript were synthesized from a key intermediate **S7**, which was synthesized by referring to the published procedures with modifications (Scheme S1).^1^

**Scheme S1.** The synthetic route of **S7**

8-Hydroxyquinoline (24.65 mg, 0.17 mmol, 0.003 equiv) was added to the solution of 3-cyanobenzoic acid (5.0 g, 34 mmol, 1.0 equiv) in 100 mL ethanol. To the reaction mixture was added hydroxylamine hydrochloride (4.76 g, 68 mmol, 2.1 equiv) solution in water (30 mL), followed by sodium carbonate (5.8 g, 54.4 mmol, 1.6 equiv) in water (31 mL). The mixture was heated to reflux for 4 h. After removal of ethanol under reduced pressure, the residue was diluted with water (100 mL), and the aqueous solution was acidified with 1*N* HCl solution to pH~3. The resulting white precipitate was filtered, washed with water and acetone, and then dried under reduced pressure to afford compound **S1** as a white solid (5.13 g, 84 % yield).

**(Z)-3-(N’-hydroxycarbamimidoyl)benzoic acid** (**S1**): ^1^H NMR (400 MHz, CDCl_3_) δ 13.03 (br s, 1H), 9.76 (s, 1H), 8.27-8.26 (m, 1H), 7.95-7.89 (m, 2H), 7.53 (t, *J* = 7.8 Hz, 1H), 5.94 (br s, 2H). LC-MS (ESI, m/z): calcd for C_8_H_9_N_2_O_3_^+^, [M + H]^+^ 181.05, found 180.9. Compound **S1** was reported before, and its characterization matched the published data. ^1^

The solution of compound **S1** (5.13 g, 28.3 mmol, 1.0 equiv) in anhydrous pyridine (50 mL) was cooled down to 0 °C and then trifluoroacetic anhydride (11.8 mL, 85 mmol, 3.0 equiv) was added dropwise. The reaction mixture was warmed slowly to room temperature and further stirred at 50 °C for 3 h. The reaction mixture was poured into ice water and adjusted to pH~4 with 1.5 *N* HCl solution and extracted with ethyl acetate (60 mL 🞩 3). The combined organic layers were washed with brine (50 mL), dried over anhydrous Na_2_SO_4_ and concentrated under vacuum, the residue was subsequently purified through column chromatography (hexanes/EtOAc: from 100:0 to 10:1) to afford the product **S2** as a white solid (2.12 g, 29 % yield).

**3-(5-(Trifluoromethyl)-1,2,4-oxadiazol-3-yl)benzoic acid (S2):** ^1^H NMR (400 MHz, CDCl_3_) δ 13.44 (s, 1H), 8.56 (s, 1H), 8.30 (d, *J* = 7.9 Hz, 1H), 8.21 (d, *J* = 7.9 Hz, 1H), 7.78 (t, *J* = 7.8 Hz, 1H). LC-MS (ESI, m/z): calcd for C_10_H_4_F_3_N_2_O_3_^-^, [M - H]^-^ 257.03, found 257.0. Compound **S2** was reported before, and its characterization matched the published data. ^1^

A mixture of 2-bromoacetophenone (5.0 g, 25.13 mmol, 1.0 equiv) and 2-cyanothioacetamide (2.5 g, 25.13 mmol, 1.0 equiv) in ethanol (75 mL) was stirred at 80 °C for 4 h. The reaction mixture was cooled down to room temperature and poured into an aqueous ammonia solution (final pH was >7). The mixture was then extracted with ethyl acetate (30 mL 🞩 3). The combined organic layers were washed with brine (30 mL), dried over anhydrous Na_2_SO_4_ and concentrated under vacuum, the residue was purified by flash column chromatography (hexanes/EtOAc: from 100:0 to 10:1) to afford the target compound **S3** as a yellow solid (3.9 g, 78 % yield).

**2-(4-Phenylthiazol-2-yl)acetonitrile** (**S3**): ^1^H NMR (400 MHz, CDCl_3_) δ 7.88 (dd, *J* = 5.3, 3.3 Hz, 2H), 7.48 (d, *J* = 4.2 Hz, 1H), 7.27-7.48 (m, 3H), 4.18 (d, *J* = 4.9 Hz, 2H); LC-MS (ESI, m/z): calcd for C_11_H_9_N_2_S^+^, [M + H]^+^ 201.04, found 201.0. Compound **S3** was reported before, and its characterization matched the published data. ^1^

To a flame-dried flask equipped with a magnetic stir bar was added compound **S3** (3.9 g, 19.5 mmol, 1.0 equiv). After the flask was evacuated and backfilled with N_2_ twice, anhydrous DMF (10 mL) was added via syringes. NaH (60% in mineral oil, 2.4 g, 58.5 mmol, 3.0 equiv) was added to the reaction at 0 °C in three portions over 20 minutes. The mixture was then stirred at room temperature for 30 minutes. *tert*-Butyl bis(2-chloroethyl)carbamate (9.4 g, 39 mmol, 2.0 equiv) in DMF (10 mL) was added slowly and the mixture was stirred at 60 °C for 3 h. The reaction was quenched with water (80 mL), and EtOAc (40 mL) was added. The organic phase was separated from the aqueous one, and the aqueous phase was extracted with EtOAc (30 mL 🞩 3). The combined organic layers were dried over anhydrous Na_2_SO_4_ and concentrated *in vacuo*. The residue was further purified through a silica gel flash column (hexanes/EtOAc: from 100:1 to 10:1) to afford the target compound **S4** as yellow oil (3.29 g, 46 % yield).

***tert*-Butyl 4-cyano-4-(4-phenylthiazol-2-yl)piperidine-1-carboxylate** (**S4**): ^1^H NMR (400 MHz, CDCl_3_) δ 7.95 – 7.83 (m, 2H), 7.50 (s, 1H), 7.47 – 7.40 (m, 2H), 7.39 – 7.30 (m, 1H), 4.23 (s, 2H), 3.27 (s, 2H), 2.37 (d, *J* = 13.1 Hz, 2H), 2.29 – 2.18 (m, 2H), 1.49 (s, 9H). LC-MS (ESI, m/z): calcd for C_20_H_24_N_3_O_2_S^+^, [M + H]^+^ 370.15, found 370.2. Compound **S4** was reported before, and its characterization matched the reported data. ^1^

A LiAlH_4_ (1.0 M) solution in THF (3.03 mL, 3.03 mmol, 1.5 equiv) was chilled to 0 °C. Compound **S4** (746 mg, 2.02 mmol, 1.0 equiv) was dissolved in THF (10 mL) and added to the LiAlH_4_ solution over 5 min. The reaction was stirred for 1 h and allowed to warm to room temperature. After stirring for an additional hour, the reaction was carefully quenched with water and diluted to 40 mL with EtOAc. The organic phase was washed with saturated sodium bicarbonate (3 🞩 10 mL), dried with magnesium sulfate, and concentrated to brown oil. The residue was subsequently purified through column chromatography (CH_2_Cl_2_ /MeOH from 99:1 to 9:1) to afford the product **S5** as colorless oil (292 mg, 39 % yield).

***tert*-Butyl 4-(aminomethyl)-4-(4-phenylthiazol-2-yl)piperidine-1-carboxylate** (**S5**): ^1^H NMR (400 MHz, CDCl_3_) δ 7.92 – 7.81 (m, 2H), 7.51 (s, 1H), 7.45 – 7.40 (m, 2H), 7.39 – 7.32 (m, 1H), 4.21 (s, 2H), 3.27 (s, 2H), 3.22 (td, *J* = 6.3, 0.8 Hz, 2H), 3.12 – 3.05 (m, 2H), 2.35 (d, *J* = 13.1 Hz, 2H), 2.27 – 2.16 (m, 2H), 1.45 (s, 9H). LC-MS (ESI, m/z): calcd for C_20_H_28_N_3_O_2_S^+^, [M + H]^+^ 374.18, found 374.2. Compound **S5** was reported before, and its characterization matched the published data.^1^

To a flame-dried flask equipped with a stir bar was added compound **S2** (1.2 g, 4.62 mmol, 1.1 equiv) and compound **S5** (1.56 g, 4.2 mmol, 1.0 equiv). After this flask was evacuated and backfilled with N_2_ twice, anhydrous CH_2_Cl_2_ (10 mL) was added via syringes. EDCI (887 mg, 4.62 mmol, 1.1 equiv), HOBt (771 mg, 5.04 mmol, 1.1 equiv) and DIPEA (2.2 mL, 12.6 mmol, 3.0 equiv) were added to the reaction at 0 °C and the mixture was stirred at room temperature for 8 h. The reaction was poured into water and CH_2_Cl_2_ was added. The organic phase was separated from the aqueous one, and the aqueous phase was extracted with CH_2_Cl_2_ 🞩 3. The combined organic layers were dried over anhydrous Na_2_SO_4_ and concentrated *in vacuo*. After concentration, the residue was purified through column chromatography to afford **S6** as a white solid (1.84 g, 71 % yield).

***tert*-Butyl 4-(4-phenylthiazol-2-yl)-4-((3-(5-(trifluoromethyl)-1,2,4-oxadiazol-3-yl)benzamido)methyl)piperidine-1-carboxylate** (**S6**)**:** ^1^H-NMR (400 MHz, DMSO-*d*_6_) δ 8.79 (t, *J* = 6.4 Hz, 1H), 8.43 (s, 1H), 8.18 (d, *J* = 7.8 Hz, 1H), 8.08 (s, 1H), 8.05 (d, *J* = 7.7 Hz, 1H), 7.93 (d, *J* = 7.3 Hz, 2H), 7.68 (t, *J* = 7.8 Hz, 1H), 7.38 (t, *J* = 7.6 Hz, 2H), 7.29 (t, *J* = 7.3 Hz, 1H), 3.83 (d, *J* = 13.5 Hz, 2H), 3.56 (d, *J* = 6.3 Hz, 2H), 2.94 (s, 2H), 2.26 (d, *J* = 14.2 Hz, 2H), 1.92 – 1.80 (m, 2H), 1.37 (s, 9H). LC-MS (ESI, m/z): calcd for C_30_H_31_F_3_N_5_O_4_S^+^, [M + H]^+^ 614.20, found 614.2. Compound **S6** was reported before, and its characterization matched the published data.^1^

Compound **S6** was further deprotected with hydrogen chloride/EtOAc solution to afford compound **S7**, which was used in next step without further purification. LC-MS (ESI, m/z): calcd for C_25_H_23_F_3_N_5_O_2_S^+^, [M + H]^+^ 514.14, found 514.1.

**TMP269** was prepared through a known literature procedure. And its characterization matched the reported data. ^1^

***N*-((4-(4-phenylthiazol-2-yl)tetrahydro-2H-pyran-4-yl)methyl)-3-(5-(trifluoromethyl)-1,2,4-oxadiazol-3-yl)benzamide** (**TMP269**)**:** ^1^H NMR (400 MHz, CDCl_3_) δ 8.49 (td, *J* = 1.8, 0.6 Hz, 1H), 8.21 (ddd, *J* = 7.8, 1.7, 1.2 Hz, 1H), 7.98 (ddd, *J* = 7.8, 1.8, 1.2 Hz, 1H), 7.89 – 7.85 (m, 2H), 7.61 – 7.51 (m, 3H), 7.38 – 7.28 (m, 3H), 3.95 (ddd, *J* = 12.0, 6.7, 3.6 Hz, 2H), 3.88 (d, *J* = 5.8 Hz, 2H), 3.74 (ddd, *J* = 11.6, 7.5, 3.4 Hz, 2H), 2.37 – 2.25 (m, 2H), 2.04 (ddd, *J* = 13.8, 7.6, 3.6 Hz, 2H). LC-MS (ESI, m/z): calcd for C_25_H_22_F_3_N_4_O_3_S^+^, [M + H]^+^ 515.13, found 515.1.

**Ac-Leu-Gly-Lys(TFAc)-AMC**, the enzymatic substrate of Class IIa HDACs, was prepared through a known literature procedure. And its characterization matched the reported data. ^2^

1. **Synthesis of the model compounds**

Model compounds **M1**, **M2**, **M3** were synthesized according to the following procedures and characterized with MS, ^1^H NMR, ^13^C NMR and HPLC.

To a stirred suspension of 4-methylbenzenesulfonic acid hydrate (1.9 g, 10 mmol, 2.0 equiv) and Dess–Martin periodinane (3.2 g, 7.5 mmol, 1.5 equiv) in MeCN (15 mL) was added the propanone (367 μL, 5 mmol, 1.0 equiv) in one portion and the reaction mixture was refluxed until complete consumption of starting material (monitored by TLC). After completion of reaction MeCN was removed under reduced pressure. The residue obtained was diluted by adding CH_2_Cl_2_ (50 mL) and washed with sat. solutions of NaHCO_3_ (2 🞩 30 mL) and brine (50 mL). The organic layer was dried over anhydrous Na_2_SO_4_ and concentrated under reduced pressure to give the crude product. Pure product was obtained after column chromatography (EtOAc/hexane: from 10:90 to 20:80) as yellow liquid (382 mg, 34 % yield).

**2-Oxopropyl 4-methylbenzenesulfonate** (**S8**): ^1^H NMR (400 MHz, CDCl_3_) δ 7.81 (d, *J* = 8.3 Hz, 2H), 7.37 (d, *J* = 7.8 Hz, 2H), 4.48 (s, 2H), 2.46 (s, 3H), 2.21 (s, 3H). LC-MS (ESI, m/z): calcd for C_10_H_13_O_4_S^+^, [M + H]^+^229.05, found 229.0.

To a flask equipped with a stir bar were added **S7** (30 mg, 0.058 mmol, 1.0 equiv), K_2_CO_3_ (48.4 mg, 0.351mmol, 6.0 equiv). After this flask was evacuated and backfilled with N_2_ twice, anhydrous DMF (0.5 mL) was added via syringes. **S8** (13.2 mg, 0.058 mmol, 1.0 equiv) was added dropwise, and the mixture was stirred at room temperature for 8 h until the starting material was fully consumed (monitored by TLC). The reaction was poured into water and EtOAc (5 mL) was added. The organic layer was separated from the aqueous one, and the aqueous phase was extracted with EtOAc (3 🞩 5 mL). The combined organic layers were dried over anhydrous Na_2_SO_4_ and concentrated *in vacuo*. The residue was further purified through a silica gel flash column to afford the desired product **M1** as a yellow solid (14 mg, 43 % yield).

***N*-((1-(2-oxopropyl)-4-(4-phenylthiazol-2-yl)piperidin-4-yl)methyl)-3-(5-(trifluoromethyl)-1,2,4-oxadiazol-3-yl)benzamide** (**M1**): ^1^H NMR (400 MHz, CDCl_3_) δ 8.51 (t, *J* = 1.7 Hz, 1H), 8.21 (dt, *J* = 7.8, 1.4 Hz, 1H), 7.99 (dt, *J* = 7.9, 1.4 Hz, 1H), 7.88 – 7.85 (m, 2H), 7.78 (s, 1H), 7.54 (t, *J* = 7.8 Hz, 1H), 7.49 (s, 1H), 7.37 – 7.30 (m, 3H), 3.87 (d, *J* = 5.7 Hz, 2H), 3.25 (s, 2H), 2.74 – 2.67 (m, 2H), 2.65 – 2.58 (m, 2H), 2.40 – 2.32 (m, 2H), 2.15 (s, 3H), 2.13 – 2.07 (m, 2H). ^13^C NMR (100 MHz, CDCl_3_) δ 168.5, 166.1, 155.1, 135.8, 134.0, 130.6, 130.4, 129.5, 128.7, 128.3, 126.3, 126.2, 125.5, 112.5, 67.9, 49.8, 42.7, 34.2, 27.8. LC-MS (ESI, m/z): calcd for C_28_H_27_F_3_N_5_O_3_S^+^, [M + H]^+^570.17, found 570.2.

To a flame-dried flask equipped with a stir bar was added **S7** (30 mg, 0.058 mmol, 1.0 equiv) and propionic acid (5 μL, 0.070 mmol, 1.2 equiv). After this flask was evacuated and backfilled with N_2_ twice, anhydrous DMF (0.5 mL) was added via syringes. EDCI (12.4 mg, 0.064 mmol, 1.1 equiv), HOBt (10.7 mg, 0.070 mmol, 1.2 equiv) and DIPEA (82 μL, 0.468 mmol, 8.0 equiv) were added to the reaction at 0 °C and the mixture was stirred at room temperature for 8 h. The reaction was poured into water and EtOAc was added. The organic phase was separated from the aqueous one, and the aqueous phase was extracted with EtOAc 🞩 3. The combined organic layers were dried over anhydrous Na_2_SO_4_ and concentrated *in vacuo*. After concentration *in vacuo*, the residue was purified through column chromatography to afford **M2** as a white solid (30 mg, 90 % yield).

***N*-((4-(4-phenylthiazol-2-yl)-1-propionylpiperidin-4-yl)methyl)-3-(5-(trifluoromethyl)-1,2,4-oxadiazol-3-yl)benzamide** (**M2**): ^1^H NMR (400 MHz, DMSO-*d*_6_) δ 8.79 (t, *J* = 6.3 Hz, 1H), 8.43 (t, *J* = 1.8 Hz, 1H), 8.18 (dt, *J* = 7.8, 1.4 Hz, 1H), 8.08 (s, 1H), 8.05 (dt, *J* = 7.8, 1.4 Hz, 1H), 7.95 – 7.91 (m, 2H), 7.68 (t, *J* = 7.8 Hz, 1H), 7.41 – 7.36 (m, 2H), 7.32 – 7.27 (m, 1H), 4.22 – 4.15 (m, 1H), 3.86 – 3.78 (m, 1H), 3.58 (d, *J* = 6.1 Hz, 2H), 3.19 – 3.10 (m, 1H), 2.88 – 2.79 (m, 1H), 2.36 – 2.23 (m, 4H), 1.95 – 1.79 (m, 2H), 0.96 (t, *J* = 7.4 Hz, 3H). ^13^C NMR (100 MHz, DMSO-*d*_6_) δ 173.8, 171.4, 168.3, 166.0, 165.1, 154.0, 135.9, 134.5, 131.5, 130.1, 129.8, 128.8, 128.0, 126.5, 126.2, 124.8, 117.3, 114.7, 49.5, 45.3, 41.8, 38.0, 34.2, 33.6, 25.7, 9.6. LC-MS (ESI, m/z): calcd for C_28_H_27_F_3_N_5_O_3_S^+^, [M + H]^+^570.17, found 570.2.

Et_3_N (16 mL, 116.4 mmol, 3.0 equiv) and DMAP (473 mg, 3.88 mmol, 0.1 equiv) were added to a solution of the hex-5-yn-1-ol (3.8 g, 38.8 mmol, 1.0 equiv) in CH_2_Cl_2_ (20 mL). The flask was cooled to 0 °C on ice bath, and a solution of TsCl (8.9 g, 46.5 mmol, 1.2 equiv) in CH_2_Cl_2_ (20 mL) was added dropwise. After stirring for overnight at room temperature, the reaction was poured into water and CH_2_Cl_2_ was added. The organic layer was separated from the aqueous one, and the aqueous phase was extracted with CH_2_Cl_2_ 🞩 3. The combined organic layers were dried over anhydrous Na_2_SO_4_ and concentrated *in vacuo*. Purification by flash column chromatography afforded a colorless product **S9** (7.4 g, 76 % yield).

**Hex-5-yn-1-yl 4-methylbenzenesulfonate** (**S9**): ^1^H NMR (400 MHz, DMSO-*d*_6_) δ 7.78 (d, *J* = 8.4 Hz, 2H), 7.48 (d, *J* = 8.6 Hz, 2H), 4.03 (t, *J* = 6.3 Hz, 2H), 2.76 (q, *J* = 2.5 Hz, 1H), 2.42 (s, 3H), 2.10 (td, *J* = 7.0, 2.7 Hz, 2H), 1.68 – 1.60 (m, 2H), 1.44 – 1.36 (m, 2H). LC-MS (ESI, m/z): calcd for C_13_H_17_O_3_S^+^, [M + H]^+^253.08, found 253.1.

To a flame-dried flask equipped with a stir bar was added compound **S3** (2.6 g, 13.0 mmol, 1.0 equiv). After the flask was evacuated and backfilled with N_2_ twice, anhydrous DMF (20 mL) was added via syringes. NaH (60% in mineral oil, 572 mg, 14.3 mmol, 1.1 equiv) was added to the reaction at 0 °C in three portions over 20 minutes. The mixture was then stirred at room temperature for 30 minutes. **S9** (3.6 g, 14.3 mmol, 1.1 equiv) in DMF (16 mL) was added slowly and the mixture was stirred at room temperature for 8 h. The reaction was quenched with water (30 mL), and EtOAc (60 mL) was added. The organic phase was separated from the aqueous one, and the aqueous phase was extracted with EtOAc (30 mL 🞩 3). The combined organic layers were dried over anhydrous Na_2_SO_4_ and concentrated *in vacuo*. The residue was further purified through a silica gel flash column (hexanes/EtOAc: from 100:0 to 98:2) to afford the target compound **S10** as yellow oil (1.0 g, 28 % yield).

**2-(4-Phenylthiazol-2-yl)oct-7-ynenitrile** (**S10**): ^1^H NMR (400 MHz, CDCl_3_) δ 7.90 – 7.87 (m, 2H), 7.48 (s, 1H), 7.46 – 7.41 (m, 2H), 7.38 – 7.36 (m, 1H), 4.34 – 4.29 (m, 1H), 2.24 (td, *J* = 6.8, 2.6 Hz, 2H), 2.19 – 2.14 (m, 2H), 1.96 (t, *J* = 2.6 Hz, 1H), 1.64 – 1.52 (m, 4H). LC-MS (ESI, m/z): calcd for C_17_H_17_N_2_S^+^, [M + H]^+^281.10, found 281.1.

Under nitrogen protection, lithium aluminum hydride (272 mg, 7.14 mmol, 4.0 equiv) was dissolved in anhydrous THF, and anhydrous THF solution (5 mL) of **S10** (500 mg, 1.79 mmol, 1.0 equiv) was added at 0 ℃. The reaction was stirred for 1 h and allowed to warm to room temperature. After stirring for an additional hour, the TLC monitoring reaction was completed. The reaction was carefully quenched with 0.3 mL H_2_O, 0.3 mL 15 % NaOH solution and 0.9 mL H_2_O in sequence at 0 ℃. After fully stirring for 10 min and filtration through celite, the filtrate was extracted three times with EtOAc (10 mL), dried with magnesium sulfate, and concentrated under reduced pressure to obtain 70 mg of crude product (**S11**). The product was used in next step without further purification.

To a flame-dried flask equipped with a stir bar was added compound **S2** (76.3 mg, 0.296 mmol, 1.2 equiv) and compound **S11** (70 mg, 0.246 mmol, 1.0 equiv). After this flask was evacuated and backfilled with N_2_ twice, anhydrous CH_2_Cl_2_ (10 mL) was added via syringes. EDCI (52 mg, 0.271 mmol, 1.1 equiv), HOBt (45.3 mg, 0.296 mmol, 1.2 equiv) and DIPEA (154 μL, 0.888 mmol, 3.0 equiv) were added to the reaction at 0 °C and the mixture was stirred at room temperature for 8 h. The reaction was poured into water and CH_2_Cl_2_ was added. The organic phase was separated from the aqueous one, and the aqueous phase was extracted with CH_2_Cl_2_ 🞩 3. The combined organic layers were dried over anhydrous Na_2_SO_4_ and concentrated *in vacuo*. After concentration *in vacuo*, the residue was purified through column chromatography to afford **M3** as a yellow solid (61 mg, 47 % yield).

***N*-(2-(4-phenylthiazol-2-yl)oct-7-yn-1-yl)-3-(5-(trifluoromethyl)-1,2,4-oxadiazol-3-yl)benzamide** (**M3**): ^1^H NMR (400 MHz, CDCl_3_) δ 8.51 (t, *J* = 1.8 Hz, 1H), 8.22 (dt, *J* = 7.9, 1.4 Hz, 1H), 8.02 – 8.00 (m, 1H), 7.88 – 7.85 (m, 2H), 7.74 – 7.69 (m, 1H), 7.57 (t, *J* = 7.7 Hz, 1H), 7.41 (s, 1H), 7.37 – 7.30 (m, 3H), 3.98 – 3.92 (m, 1H), 3.88 – 3.81 (m, 1H), 3.46 – 3.39 (m, 1H), 2.23 – 2.19 (m, 2H), 1.98 – 1.87 (m, 3H), 1.64 – 1.56 (m, 4H). ^13^C NMR (100 MHz, CDCl_3_) δ 173.0, 168.6, 166.2, 155.3, 135.9, 134.1, 130.8, 130.4, 129.5, 128.7, 128.2, 126.2, 126.2, 125.4, 112.0, 84.1, 68.6, 43.4, 42.9, 32.8, 28.2, 26.3, 18.2. LC-MS (ESI, m/z): calcd for C_27_H_24_F_3_N_4_O_2_S ^+^, [M + H]^+^ 525.15, found 525.2.

1. **Synthetic procedures of the** **CRBN-recruiting PROTACs (A1-A12)**

**Scheme S2.** The CRBN-recruiting PROTACs generated with CuAAC reaction

*Synthesis of the Class IIa HDACs binding ligand with an alkyne handle* *(****S12-S15****):*

**General Procedure A.** To a flask equipped with a stir bar were added **S7** (1.0 equiv), K_2_CO_3_ (3.0 equiv). After this flask was evacuated and backfilled with N_2_ twice, anhydrous DMF was added via syringes. Aliphatic alkyne **Ln** (1.1 equiv) was added dropwise, and the mixture was stirred at 60 °C for 8 h until the starting material was fully consumed (monitored by TLC). The reaction was poured into water and EtOAc was added. The organic layer was separated from the aqueous one, and the aqueous phase was extracted with EtOAc 🞩 3. The combined organic layers were dried over anhydrous Na_2_SO_4_ and concentrated *in vacuo*. The residue was further purified through a silica gel flash column to afford the desired alkyne product **S12-S15** (60-70 % yield) as colorless oil.

**L1** (3-bromoprop-1-yne), **L2** (but-3-yn-1-yl 4-methylbenzenesulfonate), **L3** (6-Chlorohex-1-yne), were purchased from LeYan.com and used directly without further purification. **L4** (oct-7-yn-1-yl 4-methylbenzenesulfonate) was prepared as below.

Et_3_N (3.3 mL, 23.79 mmol) and DMAP (97 mg, 0.79 mmol) were added to a solution of oct-7-yn-1-ol (1.0 g, 7.93 mmol) in CH_2_Cl_2_ (10 mL). The flask was cooled to 0 °C on ice bath, and a solution of TsCl (2.27 g, 11.9 mmol) in CH_2_Cl_2_ (10 mL) was added dropwise. After stirring for overnight at room temperature, the reaction was poured into water and CH_2_Cl_2_ was added. The organic layer was separated from the aqueous one, and the aqueous phase was extracted with CH_2_Cl_2_ 🞩 3. The combined organic layers were dried over anhydrous Na_2_SO_4_ and concentrated *in vacuo*. Purification by flash column chromatography afforded **L4** as a colorless solid (1.99 g, 90 % yield).

**Oct-7-yn-1-yl 4-methylbenzenesulfonate (L4):** ^1^H NMR (400 MHz, CDCl_3_) δ 7.78 (d, *J* = 8.3 Hz, 2H), 7.34 (d, *J* = 8.0 Hz, 2H), 4.01 (t, *J* = 6.4 Hz, 2H), 2.44 (s, 3H), 2.14 (td, *J* = 7.0, 2.6 Hz, 2H), 1.92 (t, *J* = 2.7 Hz, 1H), 1.70 – 1.59 (m, 2H), 1.46 (dq, *J* = 9.4, 6.3 Hz, 2H), 1.38 – 1.27 (m, 4H). LC-MS (ESI, m/z): calcd for C_15_H_21_O_3_S^+^, [M + H]^+^ 281.11, found 281.1.

***N*-((4-(4-phenylthiazol-2-yl)-1-(prop-2-yn-1-yl)piperidin-4-yl)methyl)-3-(5-(trifluoromethyl)-1,2,4-oxadiazol-3-yl)benzamide (S12):** ^1^H NMR (400 MHz, CDCl_3_) δ 8.51 (s, 1H), 8.21 (d, *J* = 7.8 Hz, 1H), 7.98 (d, *J* = 7.8 Hz, 1H), 7.87 (dd, *J* = 7.7, 1.6 Hz, 2H), 7.81 (s, 1H), 7.54 (td, *J* = 7.8, 1.5 Hz, 1H), 7.49 (s, 1H), 7.38 – 7.27 (m, 3H), 3.86 (d, *J* = 5.6 Hz, 2H), 3.32 (t, *J* = 2.0 Hz, 2H), 2.81 (ddd, *J* = 11.8, 8.2, 3.3 Hz, 2H), 2.73 – 2.63 (m, 2H), 2.35 (ddd, *J* = 12.8, 8.1, 3.5 Hz, 2H), 2.26 (t, *J* = 2.1 Hz, 1H), 2.11 (ddd, *J* = 13.6, 7.1, 3.5 Hz, 2H). LC-MS (ESI, m/z): calcd for C_28_H_25_F_3_N_5_O_2_S^+^, [M + H]^+^ 552.16, found 552.2.

***N*-((1-(but-3-yn-1-yl)-4-(4-phenylthiazol-2-yl)piperidin-4-yl)methyl)-3-(5-(trifluoromethyl)-1,2,4-oxadiazol-3-yl)benzamide (S13):** ^1^H NMR (400 MHz, DMSO-*d*_6_) ^1^H NMR (400 MHz, DMSO- *d*_6_) δ 8.81 (s, 1H), 8.44 (s, 1H), 8.19 (d, *J* = 7.7 Hz, 1H), 8.08 (s, 2H), 7.93 (d, *J* = 7.6 Hz, 2H), 7.81 – 7.60 (m, 1H), 7.44 – 7.36 (m, 2H), 7.35 – 7.27 (m, 1H), 3.54 (s, 2H), 3.03 – 2.63 (m, 4H), 2.43 – 2.16 (m, 5H), 2.26 – 1.91 (m, 4H). LC-MS (ESI, m/z): calcd for C_29_H_27_F_3_N_5_O_2_S^+^, [M + H]^+^ 566.18, found 566.2.

***N*-((1-(hex-5-yn-1-yl)-4-(4-phenylthiazol-2-yl)piperidin-4-yl)methyl)-3-(5-(trifluoromethyl)-1,2,4-oxadiazol-3-yl)benzamide (S14):** ^1^H NMR (400 MHz, DMSO-*d*_6_) δ 8.90 (s, 1H), 8.45 (s, 1H), 8.19 (d, *J* = 7.8 Hz, 1H), 8.11 (d, *J* = 7.3 Hz, 2H), 7.94 (d, *J* = 7.0 Hz, 1H), 7.69 (t, *J* = 7.8 Hz, 1H), 7.39 (t, *J* = 7.6 Hz, 2H), 7.30 (t, *J* = 7.3 Hz, 1H), 3.56 (s, 2H), 3.73 – 3.12 (m, 4H), 2.94 – 2.58 (m, 3H), 2.45 – 2.40 (m, 2H), 2.30 – 2.26 (m, 2H), 2.16 (q, *J* = 4.5 Hz, 2H), 1.69 – 1.64 (m, 2H), 1.42 (t, *J* = 7.7 Hz, 2H). LC-MS (ESI, m/z): calcd for C_31_H_31_F_3_N_5_O_2_S^+^, [M + H]^+^ 594.21, found 594.2.

***N*-((1-(oct-7-yn-1-yl)-4-(4-phenylthiazol-2-yl)piperidin-4-yl)methyl)-3-(5-(trifluoromethyl)-1,2,4-oxadiazol-3-yl)benzamide (S15):** ^1^H NMR (400 MHz, CDCl_3_) δ 8.52 (t, *J* = 1.8 Hz, 1H), 8.21 (dt, *J* = 7.9, 1.4 Hz, 1H), 7.99 (dt, *J* = 7.9, 1.5 Hz, 1H), 7.91 – 7.83 (m, 3H), 7.54 (t, *J* = 7.8 Hz, 1H), 7.48 (s, 1H), 7.37 – 7.29 (m, 3H), 3.86 (d, *J* = 5.6 Hz, 2H), 2.67 (d, *J* = 9.4 Hz, 2H), 2.59 (s, 2H), 2.39 (dd, *J* = 9.2, 6.3 Hz, 2H), 2.30 (q, *J* = 9.9 Hz, 2H), 2.18 (td, *J* = 7.0, 2.6 Hz, 2H), 2.13 – 2.05 (m, 2H), 1.94 (t, *J* = 2.6 Hz, 1H), 1.59 – 1.47 (m, 4H), 1.46 – 1.37 (m, 4H). LC-MS (ESI, m/z): calcd for C_33_H_35_F_3_N_5_O_2_S^+^, [M + H]^+^ 622.24, found 622.2.

*Synthesis of PROTAC compounds from Class IIa HDACs binding ligand with an alkyne handle and a collection of prepacked Terminal Azide-labeled linker-E3 ligand Conjugates (preTACs) through CuAAC reactions* *(****A1-A6, A9-A12****):*

**General Procedure B.** To a flask equipped with a stir bar were added the **preTACs** (1.0 equiv), TMP-alkyne **S12-S15** (1.0 equiv) and VitCNa (0.6 equiv). CH_2_Cl_2_ and *^t^*BuOH were added via syringes, and CuSO_4_·5H_2_O (0.2 equiv) dissolved in water was added dropwise. The mixture was stirred vigorously at room temperature for 3 h until the starting material was fully consumed (monitored by TLC). The reaction was poured into water and CH_2_Cl_2_ was added. The organic layer was separated from the aqueous one, and the aqueous phase was extracted with CH_2_Cl_2_ 🞩 3. The combined organic layers were dried over anhydrous Na_2_SO_4_ and concentrated *in vacuo*. The residue was further purified through a silica gel flash column to afford the desired triazole-based PROTACs.

All PROTACs were characterized by ^1^H NMR, ^13^C NMR and LC-MS with HPLC purity over 90 %.

*Synthesis of the* ***preTACs****:*

**Figure S1.** The chemical structures of **preTACs** used in this study

The **preTACs** were prepared through a known literature procedure except **preTAC 8**,^3^ which was prepared by the procedure below.

To a flask equipped with a stir bar were added the **S16** (867 mg, 3.14 mmol, 1.2 equiv), DIPEA (1.64 mL, 9.42 mmol, 3.0 equiv) and 3-bromopiperidine-2,6-dione (500 mg, 2.62 mmol, 1.0 equiv). After this flask was evacuated and backfilled with N_2_ twice, anhydrous DMF (13 mL) was added via syringes. And the mixture was stirred at 60 °C for 8 h until the starting material was fully consumed (monitored by TLC). The reaction was poured into water (30 mL) and EtOAc (20 mL) was added. The organic layer was separated from the aqueous one, and the aqueous phase was extracted with EtOAc (20 mL 🞩 3). The combined organic layers were dried over anhydrous Na_2_SO_4_ and concentrated *in vacuo*. The residue was further purified through a silica gel flash column to afford the desired alkyne product **S17** as a white solid (254 mg, 25 % yield).

***tert*-Butyl 4-(4-((2,6-dioxopiperidin-3-yl)amino)phenyl)piperidine-1-carboxylate** (**S17**): ^1^H-NMR (400 MHz, CDCl_3_) δ 8.11 (s, 1H), 7.06 (d, *J* = 8.5 Hz, 2H), 6.64 (d, *J* = 8.5 Hz, 2H), 4.68 (d, *J* = 3.6 Hz, 1H), 4.39 – 4.13 (m, 2H), 4.05 (dt, *J* = 12.4, 4.2 Hz, 1H), 3.01 – 2.69 (m, 4H), 2.63 – 2.49 (m, 2H), 1.90 (qd, *J* = 13.3, 4.7 Hz, 1H), 1.82 – 1.72 (m, 2H), 1.62 – 1.49 (m, 2H), 1.48 (s, 9H); LC-MS (ESI, m/z): calcd for C_21_H_30_N_3_O_4_^+^, [M + H]^+^ 388.22, found 388.2.

Compound **S17** was further deprotected with hydrogen chloride/EtOAc solution to afford compound **S18**, which was used in next step without further purification. LC-MS (ESI, m/z): calcd for C_16_H_22_N_3_O_2_S^+^, [M + H]^+^ 288.16, found 288.2.

To a flame-dried flask equipped with a stir bar was added the 7-bromoheptanoic acid (500 mg, 2.4 mmol, 1.0 equiv). After this flask was evacuated and backfilled with N_2_ twice, anhydrous DMF (0.5 M) was added via syringes. NaN_3_ (6.0 equiv) was added to the reaction and the mixture was stirred at 80 °C for 8 h until the alkenyl bromide was fully consumed (monitored by TLC). The reaction was quenched with water and EtOAc was added. The organic phase was separated from the aqueous one, and the organic phase was washed with brine 🞩 3 and dried over anhydrous Na_2_SO_4_. After concentration *in vacuo*, the residue was purified through column chromatography to afford the desired **S19** as colorless oil (309 mg, 75 % yield).

**7-Azidoheptanoic acid** (**S19**): ^1^H NMR (400 MHz, DMSO-*d*_6_) δ 11.99 (s, 1H), 3.31 (t, *J* = 6.9 Hz, 2H), 2.19 (t, *J* = 7.3 Hz, 2H), 1.50 (ddt, *J* = 14.3, 11.9, 7.1 Hz, 4H), 1.31 (dp, *J* = 11.0, 3.5 Hz, 4H). LC-MS (ESI, m/z): calcd for C_7_H_12_N_3_O_2_^-^, [M - H]^-^ 170.10, found 170.1.

To a flame-dried flask equipped with a stir bar was added **S19** (90 mg, 0.523 mmol, 1.0 equiv) and **S18** (150 mg, 0.523 mmol, 1.0 equiv). After this flask was evacuated and backfilled with N_2_ twice, anhydrous CH_2_Cl_2_ (4 mL) was added via syringes. EDCI (97 mg, 0.628 mmol, 1.2 equiv), HOBt (104 mg, 0.68 mmol, 1.3 equiv) and DIPEA (0.55 mL, 3.14 mmol, 6.0 equiv) were added to the reaction at 0 °C and the mixture was stirred at room temperature for 8 h until **S18** was fully consumed (monitored by TLC). The reaction was poured into water and CH_2_Cl_2_ was added. The organic phase was separated from the aqueous one, and the aqueous phase was extracted with CH_2_Cl_2_ 🞨 3. The combined organic layers were dried over anhydrous Na_2_SO_4_ and concentrated *in vacuo*. After concentration *in vacuo*, the residue was purified through column chromatography to afford **preTAC 8** as white foam (130 mg, 56 % yield).

**3-((4-(1-(7-Azidoheptanoyl)piperidin-4-yl)phenyl)amino)piperidine-2,6-dione** (**preTAC 8**): ^1^H NMR (400 MHz, DMSO-*d*_6_) δ 10.78 (s, 1H), 6.94 (d, *J* = 8.5 Hz, 2H), 6.61 (d, *J* = 8.5 Hz, 2H), 5.68 (d, *J* = 7.3 Hz, 1H), 4.61 – 4.47 (m, 1H), 4.26 (dt, *J* = 11.2, 5.5 Hz, 1H), 3.94 (d, *J* = 13.4 Hz, 1H), 3.42 – 3.25 (m, 2H), 3.04 (td, *J* = 13.1, 2.5 Hz, 1H), 2.80 – 2.68 (m, 1H), 2.57 (dq, *J* = 16.8, 3.8 Hz, 3H), 2.31 (t, *J* = 7.4 Hz, 2H), 2.09 (dq, *J* = 13.6, 4.8 Hz, 1H), 1.92 – 1.79 (m, 1H), 1.78 – 1.65 (m, 2H), 1.51 (dq, *J* = 9.1, 6.1 Hz, 5H), 1.33 (ddd, *J* = 11.6, 7.7, 4.1 Hz, 5H). LC-MS (ESI, m/z): calcd for C_23_H_33_N_6_O_3_^+^, [M + H]^+^ 441.25, found 441.3.

**A1** was prepared from **S13** and **preTAC 1** on a 0.106 mmol scale by following the **General Procedure B**. It was purified through column chromatography (hexanes/EtOAc: from 4:1 to 1:100, then EtOAc/MeOH: from 100:1 to 30:1) as a white solid (50 mg, 51 % yield).

***N*-((1-(2-(1-(3-((2-(2,6-dioxopiperidin-3-yl)-1,3-dioxoisoindolin-4-yl)oxy)propyl)-1H-1,2,3-triazol-4-yl)ethyl)-4-(4-phenylthiazol-2-yl)piperidin-4-yl)methyl)-3-(5-(trifluoromethyl)-1,2,4-oxadiazol-3-yl)benzamide (A1):** ^1^H NMR (400 MHz, DMSO-*d*_6_) δ 11.13 (s, 1H), 8.76 (t, *J* = 6.4 Hz, 1H), 8.44 (t, *J* = 1.8 Hz, 1H), 8.18 (dt, *J* = 7.7, 1.5 Hz, 1H), 8.08 – 8.03 (m, 2H), 7.95 – 7.91 (m, 2H), 7.88 (s, 1H), 7.79 (dd, *J* = 8.5, 7.2 Hz, 1H), 7.68 (t, *J* = 7.8 Hz, 1H), 7.46 (dd, *J* = 7.9, 5.8 Hz, 2H), 7.38 (t, *J* = 7.6 Hz, 2H), 7.31 – 7.26 (m, 1H), 5.10 (dd, *J* = 12.9, 5.4 Hz, 1H), 4.52 (t, *J* = 6.8 Hz, 2H), 4.16 (t, *J* = 6.0 Hz, 2H), 3.55 (d, *J* = 6.3 Hz, 2H), 2.95 – 2.81 (m, 3H), 2.79 – 2.72 (m, 2H), 2.62 – 2.50 (m, 4H), 2.35 – 2.25 (m, 4H), 2.24 – 2.11 (m, 2H), 2.07 – 1.95 (m, 3H). ^13^C NMR (100 MHz, DMSO-*d*_6_) δ 174.6, 173.0, 170.2, 168.3, 167.0, 165.9, 165.6, 165.1, 155.8, 153.9, 145.3, 137.3, 136.0, 134.6, 133.4, 131.5, 130.1, 129.8, 128.8, 128.0, 126.5, 126.2, 124.8, 122.5, 120.1, 116.7, 115.7, 114.5, 65.9, 57.5, 55.1, 49.7, 49.0, 46.1, 44.8, 33.7, 31.2, 30.0, 29.4, 29.2, 22.2. LC-MS (ESI, m/z): calcd for C_45_H_42_F_3_N_10_O_7_S^+^, [M + H]^+^ 923.28, found 923.3.

**A2** was prepared from **S14** and **preTAC 2** on a 0.106 mmol scale by following the **General Procedure B**. It was purified through column chromatography (hexanes/EtOAc: from 4:1 to 1:100, then EtOAc/MeOH: from 100:1 to 30:1) as a white solid (49 mg, 51 % yield).

***N*-((1-(4-(1-(4-((2-(2,6-dioxopiperidin-3-yl)-1,3-dioxoisoindolin-4-yl)oxy)butyl)-1H-1,2,3-triazol-4-yl)butyl)-4-(4-phenylthiazol-2-yl)piperidin-4-yl)methyl)-3-(5-(trifluoromethyl)-1,2,4-oxadiazol-3-yl)benzamide (A2):** ^1^H NMR (400 MHz, DMSO-*d*_6_) δ 11.12 (s, 1H), 8.79 (s, 1H), 8.44 (s, 1H), 8.18 (dt, *J* = 7.9, 1.4 Hz, 1H), 8.06 (d, *J* = 7.3 Hz, 2H), 7.92 (d, *J* = 7.3 Hz, 2H), 7.85 (s, 1H), 7.80 (dd, *J* = 8.5, 7.3 Hz, 1H), 7.68 (t, *J* = 7.8 Hz, 1H), 7.46 (dd, *J* = 14.3, 7.9 Hz, 2H), 7.38 (t, *J* = 7.6 Hz, 2H), 7.29 (t, *J* = 7.3 Hz, 1H), 5.08 (dd, *J* = 12.8, 5.4 Hz, 1H), 4.41 (t, *J* = 6.9 Hz, 2H), 4.21 (t, *J* = 6.2 Hz, 2H), 3.54 (s, 2H), 2.92 – 2.83 (m, 2H), 2.62 – 2.52 (m, 5H), 2.42 – 2.26 (m, 3H), 2.07 – 1.95 (m, 5H), 1.74 – 1.68 (m, 2H), 1.61 – 1.44 (m, 5H), 1.36 – 1.28 (m, 2H). ^13^C NMR (100 MHz, DMSO-*d*_6_) δ 173.0, 170.2, 168.3, 167.0, 165.9, 165.6, 165.2, 156.0, 154.0, 146.8, 137.3, 135.9, 134.5, 133.4, 131.5, 130.1, 129.8, 128.8, 128.0, 126.5, 126.2, 124.8, 122.0, 119.9, 117.3, 116.5, 115.5, 114.7, 68.4, 55.1, 49.4, 49.0, 48.9, 44.6, 31.5, 31.3, 31.2, 29.2, 28.9, 26.8, 26.6, 25.6, 24.9, 22.3, 22.2. LC-MS (ESI, m/z): calcd for C_48_H_48_F_3_N_10_O_7_S^+^, [M + H]^+^ 965.33, found 965.3.

**A3** was prepared from **S14** and **preTAC 4** on a 0.106 mmol scale by following the **General Procedure B**. It was purified through column chromatography (hexanes/EtOAc: from 4:1 to 1:100, then EtOAc/MeOH: from 100:1 to 30:1) as a white solid (36 mg, 34 % yield).

***N*-((1-(4-(1-(7-((2-(2,6-dioxopiperidin-3-yl)-1,3-dioxoisoindolin-4-yl)oxy)heptyl)-1H-1,2,3-triazol-4-yl)butyl)-4-(4-phenylthiazol-2-yl)piperidin-4-yl)methyl)-3-(5-(trifluoromethyl)-1,2,4-oxadiazol-3-yl)benzamide (A3):** ^1^H NMR (400 MHz, DMSO-*d*_6_) δ 11.12 (s, 1H), 8.81 (t, *J* = 6.4 Hz, 1H), 8.44 (t, *J* = 1.9 Hz, 1H), 8.18 (dt, *J* = 7.7, 1.4 Hz, 1H), 8.09 – 8.06 (m, 2H), 7.94 – 7.91 (m, 2H), 7.82 (s, 1H), 7.78 (dd, *J* = 8.5, 7.2 Hz, 1H), 7.68 (t, *J* = 7.8 Hz, 1H), 7.47 (d, *J* = 8.6 Hz, 1H), 7.42 (d, *J* = 7.2 Hz, 1H), 7.40 – 7.36 (m, 2H), 7.31 – 7.27 (m, 1H), 5.08 (dd, *J* = 12.8, 5.4 Hz, 1H), 4.27 (t, *J* = 7.0 Hz, 2H), 4.16 (t, *J* = 6.4 Hz, 2H), 3.55 (d, *J* = 6.3 Hz, 2H), 3.07 – 2.97 (m, 2H), 2.92 – 2.84 (m, 1H), 2.63 – 2.51 (m, 5H), 2.37 (d, *J* = 10.2 Hz, 4H), 2.16 – 1.95 (m, 4H), 1.79 – 1.68 (m, 4H), 1.58 – 1.50 (m, 4H), 1.48 – 1.30 (m, 6H). ^13^C NMR (100 MHz, DMSO-*d*_6_) δ 173.0, 170.2, 168.3, 167.1, 166.0, 165.6, 165.6, 156.2, 154.0, 146.7, 137.2, 135.9, 134.5, 133.4, 131.5, 129.8, 128.8, 128.0, 126.6, 126.2, 124.8, 122.0, 119.9, 116.4, 115.4, 114.8, 68.9, 56.9, 49.4, 49.3, 48.9, 48.8, 44.6, 32.7, 31.2, 29.8, 29.2, 28.5, 28.2, 26.8, 26.0, 25.3, 24.9, 22.2. LC-MS (ESI, m/z): calcd for C_51_H_54_F_3_N_10_O_7_S^+^, [M + H]^+^ 1007.38, found 1007.4.

**A4** was prepared from **S12** and **preTAC 4** on a 0.091 mmol scale by following the **General Procedure B**. It was purified through column chromatography (hexanes/EtOAc: from 4:1 to 1:100, then EtOAc/MeOH: from 100:1 to 30:1) as a white solid (44 mg, 50 % yield).

***N*-((1-((1-(7-((2-(2,6-dioxopiperidin-3-yl)-1,3-dioxoisoindolin-4-yl)oxy)heptyl)-1H-1,2,3-triazol-4-yl)methyl)-4-(4-phenylthiazol-2-yl)piperidin-4-yl)methyl)-3-(5-(trifluoromethyl)-1,2,4-oxadiazol-3-yl)benzamide (A4):** ^1^H NMR (400 MHz, DMSO-*d*_6_) δ 11.12 (s, 1H), 8.75 (s, 1H), 8.43 (t, *J* = 1.8 Hz, 1H), 8.17 (dt, *J* = 7.9, 1.5 Hz, 1H), 8.07 – 8.02 (m, 2H), 7.96 (s, 1H), 7.93 – 7.90 (m, 2H), 7.78 (dd, *J* = 8.5, 7.2 Hz, 1H), 7.67 (t, *J* = 7.8 Hz, 1H), 7.48 (d, *J* = 8.5 Hz, 1H), 7.42 (d, *J* = 7.2 Hz, 1H), 7.40 – 7.35 (m, 2H), 7.31 – 7.26 (m, 1H), 5.08 (dd, *J* = 12.9, 5.4 Hz, 1H), 4.29 (t, *J* = 6.9 Hz, 2H), 4.16 (t, *J* = 6.3 Hz, 2H), 3.53 (d, *J* = 6.2 Hz, 4H), 2.94 – 2.68 (m, 3H), 2.63 – 2.50 (m, 3H), 2.30 (d, *J* = 13.6 Hz, 2H), 2.21 – 2.12 (m, 1H), 2.05 – 1.94 (m, 3H), 1.80 – 1.68 (m, 4H), 1.46 – 1.26 (m, 6H). ^13^C NMR (100 MHz, DMSO-*d*_6_) δ 173.0, 170.2, 168.3, 167.1, 165.8, 165.5, 165.1, 156.2, 153.9, 137.2, 135.9, 134.6, 130.0, 129.8, 128.8, 128.0, 126.5, 126.2, 119.9, 117.3, 116.4, 115.3, 114.5, 68.9, 55.1, 49.4, 48.9, 44.7, 31.2, 29.7, 29.2, 28.9, 28.4, 28.1, 25.8, 25.3, 22.2. LC-MS (ESI, m/z): calcd for C_48_H_48_F_3_N_10_O_7_S^+^, [M + H]^+^ 965.33, found 965.3.

**A5** was prepared from **S13** and **preTAC 3** on a 0.177 mmol scale by following the **General Procedure B**. It was purified through column chromatography (hexanes/EtOAc: from 4:1 to 1:100, then EtOAc/MeOH: from 100:1 to 30:1) as a white solid (55.8 mg, 33 % yield).

***N*-((1-(2-(1-(6-((2-(2,6-dioxopiperidin-3-yl)-1,3-dioxoisoindolin-4-yl)oxy)hexyl)-1H-1,2,3-triazol-4-yl)ethyl)-4-(4-phenylthiazol-2-yl)piperidin-4-yl)methyl)-3-(5-(trifluoromethyl)-1,2,4-oxadiazol-3-yl)benzamide (A5):** ^1^H NMR (400 MHz, DMSO-*d*_6_) δ 11.12 (s, 1H), 8.74 (t, *J* = 6.4 Hz, 1H), 8.43 (t, *J* = 1.8 Hz, 1H), 8.18 (dt, *J* = 7.8, 1.4 Hz, 1H), 8.07 – 8.03 (m, 2H), 7.94 – 7.91 (m, 2H), 7.82 (s, 1H), 7.77 (dd, *J* = 8.5, 7.3 Hz, 1H), 7.68 (t, *J* = 7.8 Hz, 1H), 7.47 (d, *J* = 8.6 Hz, 1H), 7.42 (d, *J* = 7.3 Hz, 1H), 7.39 – 7.35 (m, 2H), 7.31 – 7.26 (m, 1H), 5.07 (dd, *J* = 12.9, 5.4 Hz, 1H), 4.27 (t, *J* = 7.0 Hz, 2H), 4.15 (t, *J* = 6.3 Hz, 2H), 3.55 (d, *J* = 5.9 Hz, 2H), 2.93 – 2.77 (m, 3H), 2.76 – 2.69 (m, 2H), 2.62 – 2.52 (m, 2H), 2.48 – 2.43 (m, 1H), 2.31 (d, *J* = 12.2 Hz, 2H), 2.23 – 2.05 (m, 2H), 2.03 – 1.93 (m, 3H), 1.81 – 1.69 (m, 4H), 1.49 – 1.42 (m, 2H), 1.30 – 1.22 (m, 3H). ^13^C NMR (100 MHz, DMSO-*d*_6_) δ 173.0, 170.2, 168.3, 167.1, 165.8, 165.5, 156.2, 153.9, 137.2, 136.0, 134.6, 133.4, 131.5, 130.0, 129.8, 128.8, 128.0, 126.5, 126.2, 124.8, 122.3, 119.9, 117.3, 116.4, 115.4, 114.6, 114.8, 68.8, 57.7, 55.1, 49.7, 49.2, 48.9, 44.9, 33.8, 31.2, 29.8, 29.2, 28.4, 25.6, 24.9, 23.4, 22.2. HR-MS (ESI, m/z): calcd for C_48_H_48_F_3_N_10_O_7_S^+^, [M + H]^+^ 965.3312, found 965.3319.

**A6** was prepared from **S15** and **preTAC 1** on a 0.097 mmol scale by following the **General Procedure B**. It was purified through column chromatography (hexanes/EtOAc: from 4:1 to 1:100, then EtOAc/MeOH: from 100:1 to 30:1) as a white solid (35 mg, 37 % yield).

***N*-((1-(6-(1-(3-((2-(2,6-dioxopiperidin-3-yl)-1,3-dioxoisoindolin-4-yl)oxy)propyl)-1H-1,2,3-triazol-4-yl)hexyl)-4-(4-phenylthiazol-2-yl)piperidin-4-yl)methyl)-3-(5-(trifluoromethyl)-1,2,4-oxadiazol-3-yl)benzamide (A6):** ^1^H NMR (400 MHz, DMSO-*d*_6_) δ 11.13 (s, 1H), 8.82 (s, 1H), 8.44 (t, *J* = 1.7 Hz, 1H), 8.19 (d, *J* = 6.7 Hz, 1H), 8.07 (d, *J* = 6.5 Hz, 2H), 7.94 – 7.91 (m, 2H), 7.86 (s, 1H), 7.80 (dd, *J* = 8.5, 7.2 Hz, 1H), 7.68 (t, *J* = 7.7 Hz, 1H), 7.47 (dd, *J* = 7.9, 5.7 Hz, 2H), 7.40 – 7.36 (m, 2H), 7.31 – 7.27 (m, 1H), 5.10 (dd, *J* = 12.9, 5.4 Hz, 1H), 4.52 (t, *J* = 6.8 Hz, 2H), 4.18 (d, *J* = 6.0 Hz, 2H), 3.56 (d, *J* = 6.0 Hz, 2H), 3.15 – 2.96 (m, 2H), 2.95 – 2.83 (m, 2H), 2.63 – 2.51 (m, 6H), 2.41 – 2.26 (m, 6H), 2.19 – 2.02 (m, 4H), 1.59 – 1.42 (m, 6H). ^13^C NMR (100 MHz, DMSO-*d*_6_) δ 173.0, 170.2, 168.3, 167.0, 165.9, 165.6, 165.1, 155.8, 154.0, 147.1, 137.3, 135.9, 134.5, 133.4, 131.5, 130.1, 129.8, 128.8, 128.0, 126.2, 124.8, 122.1, 120.1, 117.3, 116.7, 115.7, 114.6, 65.9, 60.0, 49.5, 49.0, 46.1, 34.7, 31.3, 30.0, 29.4, 29.2, 29.0, 28.5, 26.6, 25.1, 22.2. LC-MS (ESI, m/z): calcd for C_49_H_50_F_3_N_10_O_7_S^+^, [M + H]^+^ 979.35, found 979.4.

**A9** was prepared from **S13** and **preTAC 5** on a 0.088 mmol scale by following the **General Procedure B**. It was purified through column chromatography (hexanes/EtOAc: from 4:1 to 1:100, then EtOAc/MeOH: from 100:1 to 30:1) as a white solid (32 mg, 38 % yield).

***N*-((1-(2-(1-(3-(2-((2-(2,6-dioxopiperidin-3-yl)-1,3-dioxoisoindolin-4-yl)oxy)ethoxy)propyl)-1H-1,2,3-triazol-4-yl)ethyl)-4-(4-phenylthiazol-2-yl)piperidin-4-yl)methyl)-3-(5-(trifluoromethyl)-1,2,4-oxadiazol-3-yl)benzamide (A9):** ^1^H NMR (400 MHz, DMSO-*d*_6_) δ 11.13 (s, 1H), 8.76 (t, *J* = 6.2 Hz, 1H), 8.44 (t, *J* = 1.8 Hz, 1H), 8.18 (d, *J* = 8.0 Hz, 1H), 8.07 – 8.04 (m, 2H), 7.94 – 7.91 (m, 2H), 7.81 (s, 1H), 7.78 (dd, *J* = 8.5, 7.3 Hz, 1H), 7.68 (t, *J* = 7.8 Hz, 1H), 7.51 (d, *J* = 8.6 Hz, 1H), 7.44 (d, *J* = 7.3 Hz, 1H), 7.37 (t, *J* = 7.5 Hz, 2H), 7.31 – 7.26 (m, 1H), 5.07 (dd, *J* = 12.8, 5.4 Hz, 1H), 4.36 – 4.31 (m, 4H), 3.75 – 3.72 (m, 2H), 3.54 (d, *J* = 6.3 Hz, 2H), 3.43 (t, *J* = 6.0 Hz, 2H), 2.92 – 2.80 (m, 3H), 2.76 – 2.70 (m, 2H), 2.61 – 2.51 (m, 3H), 2.32 (d, *J* = 12.6 Hz, 2H), 2.26 – 2.09 (m, 2H), 2.07 – 1.93 (m, 6H). ^13^C NMR (100 MHz, DMSO-*d*_6_) δ 173.0, 170.2, 168.3, 167.0, 165.9, 165.6, 165.5, 165.1, 156.0, 153.9, 145.0, 137.2, 136.0, 134.6, 133.4, 131.5, 129.8, 128.8, 128.0, 126.5, 126.2, 122.6, 120.2, 117.3, 116.5, 115.6, 114.5, 68.8, 68.6, 67.2, 57.6, 49.6, 48.9, 46.5, 44.8, 33.7, 31.1, 30.1, 29.2, 23.2, 22.2. LC-MS (ESI, m/z): calcd for C_47_H_46_F_3_N_10_O_8_S^+^, [M + H]^+^ 967.31, found 967.3.

**A10** was prepared from **S13** and **preTAC 6** on a 0.067 mmol scale by following the **General Procedure B**. It was purified through column chromatography (hexanes/EtOAc: from 4:1 to 1:100, then EtOAc/MeOH: from 100:1 to 30:1) as a white solid (37 mg, 57 % yield).

***N*-((1-(2-(1-(6-((2-(2,6-dioxopiperidin-3-yl)-1,3-dioxoisoindolin-5-yl)oxy)hexyl)-1H-1,2,3-triazol-4-yl)ethyl)-4-(4-phenylthiazol-2-yl)piperidin-4-yl)methyl)-3-(5-(trifluoromethyl)-1,2,4-oxadiazol-3-yl)benzamide (A10):** ^1^H NMR (400 MHz, DMSO-*d*_6_) δ 11.13 (s, 1H), 8.78 (s, 1H), 8.44 (t, *J* = 1.8 Hz, 1H), 8.18 (dt, *J* = 7.8, 1.4 Hz, 1H), 8.09 – 8.05 (m, 2H), 7.94 – 7.91 (m, 2H), 7.85 (s, 1H), 7.81 (d, *J* = 8.3 Hz, 1H), 7.68 (t, *J* = 7.8 Hz, 1H), 7.40 – 7.35 (m, 3H), 7.32 – 7.26 (m, 2H), 5.12 (dd, *J* = 12.9, 5.4 Hz, 1H), 4.28 (t, *J* = 6.9 Hz, 2H), 4.12 (t, *J* = 6.4 Hz, 2H), 3.56 (d, *J* = 5.9 Hz, 2H), 3.02 – 2.74 (m, 5H), 2.63 – 2.51 (m, 3H), 2.35 (d, *J* = 12.8 Hz, 2H), 2.23 – 1.93 (m, 5H), 1.81 – 1.68 (m, 4H), 1.47 – 1.28 (m, 5H). ^13^C NMR (100 MHz, DMSO-*d*_6_) δ 173.0, 170.2, 168.3, 167.1, 167.0, 165.9, 165.6, 165.1, 164.2, 154.0, 135.9, 134.6, 134.1, 131.5, 130.1, 129.8, 128.8, 128.0, 126.5, 126.2, 125.5, 124.8, 123.1, 122.3, 120.9, 117.3, 114.6, 109.0, 68.8, 55.1, 49.5, 49.3, 49.2, 44.7, 33.9, 31.2, 29.8, 29.2, 28.9, 28.4, 25.7, 25.0, 22.3. LC-MS (ESI, m/z): calcd for C_48_H_48_F_3_N_10_O_7_S^+^, [M + H]^+^ 965.33, found 965.3.

**A11** was prepared from **S13** and **preTAC 7** on a 0.088 mmol scale by following the **General Procedure B**. It was purified through column chromatography (hexanes/EtOAc: from 4:1 to 1:100, then EtOAc/MeOH: from 100:1 to 30:1) as a white solid (30 mg, 36 % yield).

***N*-((1-(2-(1-(6-((2-(2,6-dioxopiperidin-3-yl)-1-oxoisoindolin-4-yl)amino)hexyl)-1H-1,2,3-triazol-4-yl)ethyl)-4-(4-phenylthiazol-2-yl)piperidin-4-yl)methyl)-3-(5-(trifluoromethyl)-1,2,4-oxadiazol-3-yl)benzamide (A11):** ^1^H NMR (400 MHz, DMSO-*d*_6_) δ 11.02 (s, 1H), 8.87 (s, 1H), 8.44 (t, *J* = 1.8 Hz, 1H), 8.18 (dt, *J* = 7.8, 1.5 Hz, 1H), 8.15 – 8.05 (m, 2H), 7.95 – 7.91 (m, 2H), 7.88 (s, 1H), 7.68 (t, *J* = 7.8 Hz, 1H), 7.38 (t, *J* = 7.5 Hz, 2H), 7.32 – 7.23 (m, 2H), 6.91 (d, *J* = 7.4 Hz, 1H), 6.70 (d, *J* = 8.0 Hz, 1H), 5.57 (t, *J* = 5.5 Hz, 1H), 5.11 (dd, *J* = 13.3, 5.1 Hz, 1H), 4.28 (t, *J* = 7.0 Hz, 2H), 4.22 (d, *J* = 17.2 Hz, 1H), 4.11 (d, *J* = 17.2 Hz, 1H), 3.66 – 3.40 (m, 6H), 3.12 – 3.02 (m, 3H), 2.99 – 2.85 (m, 3H), 2.68 – 2.51 (m, 5H), 2.48 – 2.34 (m, 2H), 2.35 – 2.22 (m, 2H), 2.05 – 1.99 (m, 1H), 1.82 – 1.72 (m, 2H), 1.57 – 1.50 (m, 2H), 1.39 – 1.33 (m, 2H). ^13^C NMR (100 MHz, DMSO-*d*_6_) δ 173.1, 171.5, 169.1, 168.3, 166.0, 165.6, 165.1, 154.0, 144.0, 135.9, 134.5, 132.2, 131.6, 130.1, 129.8, 129.4, 128.8, 128.0, 126.7, 126.6, 126.6, 126.2, 124.8, 122.5, 117.3, 111.9, 110.1, 51.7, 49.4, 46.0, 42.8, 42.7, 31.4, 29.9, 29.2, 28.5, 26.2, 25. 9, 23.0. LC-MS (ESI, m/z): calcd for C_48_H_51_F_3_N_11_O_5_S^+^, [M + H]^+^ 950.37, found 950.4.

**A12** was prepared from **S13** and **preTAC 8** on a 0.078 mmol scale by following the **General Procedure B**. It was purified through column chromatography (hexanes/EtOAc: from 4:1 to 1:100, then EtOAc/MeOH: from 100:1 to 30:1) as a white solid (33 mg, 42 % yield).

***N*-((1-(2-(1-(7-(4-(4-((2,6-dioxopiperidin-3-yl)amino)phenyl)piperidin-1-yl)-7-oxoheptyl)-1H-1,2,3-triazol-4-yl)ethyl)-4-(4-phenylthiazol-2-yl)piperidin-4-yl)methyl)-3-(5-(trifluoromethyl)-1,2,4-oxadiazol-3-yl)benzamide (A12):** ^1^H NMR (400 MHz, DMSO-*d*_6_) δ 10.79 (s, 1H), 8.80 (s, 1H), 8.45 (t, *J* = 1.8 Hz, 1H), 8.19 (dt, *J* = 7.8, 1.4 Hz, 1H), 8.09 – 8.03 (m, 2H), 7.95 – 7.90 (m, 2H), 7.85 (s, 1H), 7.68 (t, *J* = 7.8 Hz, 1H), 7.38 (t, *J* = 7.6 Hz, 2H), 7.32 – 7.26 (m, 1H), 6.93 (d, *J* = 8.6 Hz, 2H), 6.60 (d, *J* = 8.3 Hz, 2H), 5.68 (d, *J* = 7.5 Hz, 1H), 4.50 (d, *J* = 12.7 Hz, 1H), 4.26 (t, *J* = 6.7 Hz, 3H), 3.90 (d, *J* = 12.6 Hz, 1H), 3.56 (d, *J* = 4.6 Hz, 2H), 3.01 (t, *J* = 12.0 Hz, 2H), 2.87 – 2.67 (m, 4H), 2.62 – 2.51 (m, 5H), 2.39 – 2.32 (m, 2H), 2.27 (t, *J* = 7.4 Hz, 2H), 2.16 – 1.92 (m, 4H), 1.93 – 1.55 (m, 6H), 1.51 – 1.37 (m, 4H), 1.37 – 1.26 (m, 4H). ^13^C NMR (100 MHz, DMSO-*d*_6_) δ 174.0, 173.9, 173.3, 173.2, 170.4, 168.3, 165.9, 154.0, 146.4, 146.3, 135.9, 134.6, 133.8, 131.5, 130.1, 129.8, 128.8, 128.0, 127.2, 126.5, 126.2, 124. 8, 122.3, 114.6, 112.9, 55.1, 52.8, 49.5, 49.3, 45.8, 41.8, 41.2, 34.1, 33.3, 32.4, 31.5, 30.9, 29.8, 29.2, 28.9, 28.3, 25.9, 24.9, 22.3. LC-MS (ESI, m/z): calcd for C_52_H_59_F_3_N_11_O_5_S^+^, [M + H]^+^ 1006.43, found 1006.4.

PROTACs **A7** and **A8** were synthesized according to the procedures below.

A solution of TsCl (945 mg, 4.95 mmol, 1.0 equiv) in anhydrous CH_2_Cl_2_ (30 mL) was slowly added to a solution of dodecane-1,12-diol (1 g, 4.95 mmol, 1.0 equiv) in 30 mL of anhydrous CH_2_Cl_2_ at 0 ℃. Triethylamine (1.03 mL, 7.42 mmol, 1.5 equiv) and DMAP (60.4 mg, 0.495 mmol, 0.1 equiv) were added. After stirring at room temperature for 8 h, the reaction was quenched with water (50 mL) and the organic phase was separated, dried with anhydrous Na_2_SO_4_ and filtered. The solvent was removed under reduced pressure and the residue was purified by column chromatography to afford **S20** as a white solid (634 mg, 36 % yield).

**12-Hydroxydodecyl 4-methylbenzenesulfonate (S20):** ^1^H NMR (400 MHz, CDCl_3_) δ 7.78 (d, J = 8.3 Hz, 2H), 7.34 (d, J = 7.7 Hz, 2H), 4.01 (t, J = 6.5 Hz, 2H), 3.63 (t, J = 6.6 Hz, 2H), 2.44 (s, 3H), 1.66 – 1.51 (m, 8H), 1.38 – 1.22 (m, 12H); LC-MS (ESI, m/z): calcd for C_19_H_33_O_4_S^+^, [M + H]^+^ 357.20, found 357.2.

2-(2,6-dioxopiperidin-3-yl)-4-hydroxyisoindoline-1,3-dione (250 mg, 0.91 mmol, 1.0 equiv), **S20** (356 mg, 1.0 mmol, 1.1 equiv), potassium iodide (151 mg, 0.91 mmol, 1.0 equiv), NaHCO_3_ (153 mg, 1.82 mmol, 2.0 equiv) were dissolved in DMF (3 mL) and stirred for 24 h at 70 °C. Concentrated, diluted with EtOAc (10 mL), washed with saturated sodium bicarbonate solution (10 mL) four times, the organic layers were combined and dried over anhydrous Na_2_SO_4_. Concentrated under reduced pressure to obtain 329 mg of crude product **S21**. The product was used in next step without further purification.

Et_3_N (0.3 mL, 2.16 mmol, 3.0 equiv) and DMAP (8.8 mg, 0.07 mmol, 0.1 equiv) were added to a solution of **S21** (329 mg, 0.72 mmol, 1.0 equiv) in CH_2_Cl_2_ (2 mL). The flask was cooled to 0 °C on ice bath, and a solution of TsCl (206 mg, 1.08 mmol, 1.5 equiv) in CH_2_Cl_2_ (2 mL) was added dropwise. After stirring for overnight at room temperature, the reaction was poured into water and CH_2_Cl_2_ was added. The organic layer was separated from the aqueous one, and the aqueous phase was extracted with CH_2_Cl_2_ 🞩 3. The combined organic layers were dried over anhydrous Na_2_SO_4_ and concentrated *in vacuo*. Purification by flash column chromatography (hexanes/EtOAc: from 80:20 to 70:30 afforded a white solid **S22** (147 mg, 33 % yield).

**12-((2-(2,6-Dioxopiperidin-3-yl)-1,3-dioxoisoindolin-4-yl)oxy)dodecyl 4-methylbenzenesulfonate (S22):** ^1^H NMR (400 MHz, CDCl_3_) δ 8.10 (s, 1H), 7.78 (d, *J* = 8.3 Hz, 2H), 7.66 (dd, *J* = 8.5, 7.3 Hz, 1H), 7.44 (d, *J* = 7.2 Hz, 1H), 7.34 (d, *J* = 8.1 Hz, 2H), 7.21 (d, *J* = 8.4 Hz, 1H), 4.95 (dd, *J* = 12.3, 5.3 Hz, 1H), 4.16 (t, *J* = 6.6 Hz, 2H), 4.01 (t, *J* = 6.5 Hz, 2H), 2.91 – 2.80 (m, 2H), 2.78 – 2.72 (m, 1H), 2.44 (s, 3H), 2.14 – 2.09 (m, 1H), 1.91 – 1.84 (m, 2H), 1.63 – 1.58 (m, 2H), 1.53 – 1.46 (m, 2H), 1.36 – 1.22 (m, 14H). LC-MS (ESI, m/z): calcd for C_32_H_41_N_2_O_8_S^+^, [M + H]^+^ 613.25, found 613.3.

To a flask equipped with a stir bar were added **S7** (60 mg, 0.109 mmol, 1.0 equiv), DIPEA (95 μL, 5.0 equiv) and potassium iodide (21.7 mg, 0.131 mmol, 1.2 equiv). After this flask was evacuated and backfilled with N_2_ twice, anhydrous DMF (1 mL) was added via syringes. **S22** (80 mg, 0.131 mmol, 1.2 equiv) was dissolved in anhydrous DMF (1 mL) and then added dropwise to the reaction, and the mixture was stirred for overnight at room temperature until the starting material was fully consumed (monitored by TLC). The reaction was poured into water and EtOAc was added. The organic layer was separated from the aqueous one, and the aqueous phase was extracted with EtOAc 🞩 3. The combined organic layers were dried over anhydrous Na_2_SO_4_ and concentrated *in vacuo*. The residue was further purified through a silica gel flash column (CH_2_Cl_2_/EtOAc: from 30:70 to 10:90) to afford the desired product **A7** (29 mg, 28 % yield) as a yellow solid.

***N*-((1-(12-((2-(2,6-dioxopiperidin-3-yl)-1,3-dioxoisoindolin-4-yl)oxy)dodecyl)-4-(4-phenylthiazol-2-yl)piperidin-4-yl)methyl)-3-(5-(trifluoromethyl)-1,2,4-oxadiazol-3-yl)benzamide (A7):** ^1^H NMR (400 MHz, CDCl_3_) δ 8.51 (t, *J* = 1.8 Hz, 1H), 8.21 (dt, *J* = 7.8, 1.4 Hz, 1H), 7.99 (dt, *J* = 7.8, 1.5 Hz, 1H), 7.90 – 7.83 (m, 2H), 7.65 (dd, *J* = 8.5, 7.3 Hz, 1H), 7.54 (t, *J* = 7.8 Hz, 1H), 7.48 (s, 1H), 7.43 (d, *J* = 7.3 Hz, 1H), 7.38 – 7.25 (m, 3H), 7.20 (d, *J* = 8.5 Hz, 1H), 4.94 (dd, *J* = 12.4, 5.1 Hz, 1H), 4.16 (t, *J* = 6.4 Hz, 2H), 3.86 (d, *J* = 5.6 Hz, 2H), 2.92 – 2.64 (m, 7H), 2.50 – 2.28 (m, 4H), 2.15 – 2.06 (m, 3H), 1.86 (dt, *J* = 14.6, 6.7 Hz, 2H), 1.55 – 1.45 (m, 4H), 1.44 – 1.22 (m, 14H). ^13^C NMR (100 MHz, CDCl_3_) δ 171.4, 168.5, 168.4, 167.1, 166.1, 165.6, 156.7, 155.0, 136.4, 135.8, 134.1, 133.8, 130.4, 129.4, 128.7, 128.2, 126.3, 126.2, 125.4, 118.8, 117.0, 115.6, 112.5, 69.4, 58.6, 49.4, 49.1, 43.0, 34.0, 31.5, 29.6, 29.4, 29.3, 29.3, 29.2, 29.1, 28.8, 27.5, 26.4, 25.6, 22.6. LC-MS (ESI, m/z): calcd for C_50_H_55_F_3_N_7_O_7_S^+^, [M + H]^+^ 954.38, found 954.4.

Compounds **A8** was synthesized by following procedures similar to **A7**.

**2-(2-(2-(2-Hydroxyethoxy)ethoxy)ethoxy)ethyl 4-methylbenzenesulfonate (S23):** ^1^H NMR (400 MHz, CDCl_3_) δ 7.79 (d, J = 8.3 Hz, 2H), 7.34 (d, J = 7.9 Hz, 2H), 4.17 – 4.14 (m, 2H), 3.72 – 3.58 (m, 15H), 2.44 (s, 3H). LC-MS (ESI, m/z): calcd for C_15_H_24_O_7_S^+^, [M + H]^+^ 349.12, found 349.1.

**2-(2-(2-(2-((2-(2,6-Dioxopiperidin-3-yl)-1,3-dioxoisoindolin-4-yl)oxy)ethoxy)ethoxy)ethoxy)ethyl 4-methylbenzenesulfonate (S25):** ^1^H NMR (400 MHz, CDCl_3_) δ 8.16 (s, 1H), 7.78 (d, *J* = 8.3 Hz, 2H), 7.67 (dd, *J* = 8.5, 7.3 Hz, 1H), 7.46 (d, *J* = 7.3 Hz, 1H), 7.33 (d, *J* = 8.0 Hz, 2H), 7.27 (d, *J* = 8.8 Hz, 1H), 4.95 (dd, *J* = 12.2, 5.3 Hz, 1H), 4.36 – 4.33 (m, 2H), 4.16 – 4.13 (m, 2H), 3.95 – 3.92 (m, 2H), 3.78 – 3.75 (m, 2H), 3.69 – 3.66 (m, 2H), 3.65 – 3.62 (m, 2H), 3.59 – 3.56 (m, 4H), 2.90 – 2.72 (m, 3H), 2.43 (s, 3H), 2.15 – 2.09 (m, 1H). LC-MS (ESI, m/z): calcd for C_28_H_33_N_2_O_11_S^+^, [M + H]^+^ 605.17, found 605.2.

***N*-((1-(2-(2-(2-(2-((2-(2,6-dioxopiperidin-3-yl)-1,3-dioxoisoindolin-4-yl)oxy)ethoxy)ethoxy)ethoxy)ethyl)-4-(4-phenylthiazol-2-yl)piperidin-4-yl)methyl)-3-(5-(trifluoromethyl)-1,2,4-oxadiazol-3-yl)benzamide (A8):** ^1^H NMR (400 MHz, CDCl_3_) δ 8.50 (t, *J* = 1.8 Hz, 1H), 8.20 (dt, *J* = 7.8, 1.4 Hz, 1H), 8.01 (d, *J* = 7.9 Hz, 1H), 7.87 – 7.83 (m, 2H), 7.65 (dd, *J* = 8.5, 7.3 Hz, 1H), 7.55 (t, *J* = 7.8 Hz, 1H), 7.49 (s, 1H), 7.44 (d, *J* = 7.3 Hz, 1H), 7.37 – 7.30 (m, 3H), 7.24 (d, *J* = 8.3 Hz, 1H), 4.94 (dd, *J* = 12.0, 5.4 Hz, 1H), 4.32 (t, *J* = 5.1 Hz, 2H), 3.92 (t, *J* = 4.6 Hz, 2H), 3.91 – 3.82 (m, 2H), 3.81 – 3.73 (m, 4H), 3.68 – 3.60 (m, 6H), 3.21 – 2.98 (m, 2H), 2.92 – 2.70 (m, 6H), 2.55 – 2.41 (m, 2H), 2.36 – 2.16 (m, 3H), 2.13 – 2.09 (m, 1H). ^13^C NMR (100 MHz, CDCl_3_) δ 171.4, 168.6, 168.5, 167.0, 166.4, 165.6, 156.3, 155.0, 136.5, 135.5, 134.0, 133.7, 130.8, 130.5, 129.5, 128.7, 128.3, 126.3, 126.2, 125.4, 119.2, 117.2, 116.1, 112.9, 71.2, 70.5, 70.3, 70.3, 69.3, 69.2, 57.1, 49.8, 49.1, 42.6, 32.7, 31.4, 29.7, 22.6. LC-MS (ESI, m/z): calcd for C_46_H_47_F_3_N_7_O_10_S^+^, [M + H]^+^ 946.30, found 946.3.

1. **Synthetic procedures of the VHL-recruiting PROTACs (B1-B6)**

**Scheme S3.** The synthetic route of **B1-B6**

**General Procedure C.** To a flask equipped with a stir bar were added **S7** (1.0 equiv), K_2_CO_3_ (3.0 equiv). After this flask was evacuated and backfilled with N_2_ twice, anhydrous DMF was added via syringes. **L5-L9** (1.1 equiv) was added dropwise, and the mixture was stirred at 60 °C for 8 h until the starting material was fully consumed (monitored by TLC). The reaction was poured into water and EtOAc was added. The organic layer was separated from the aqueous one, and the aqueous phase was extracted with EtOAc 🞩 3. The combined organic layers were dried over anhydrous Na_2_SO_4_ and concentrated *in vacuo*. The residue was further purified through a silica gel flash column to afford the desired alkyne product **S26-S30** (60-70 % yield) as a colorless oil.

**General Procedure D.** **S26-S30** was treated with 30% TFA/CH_2_Cl_2_ solution at room temperature for 8 h to afford **S26-S30** acid. The mixture was concentrated *in vacuo* and mixed with the **E5** or **E6** (1.0 equiv), EDCI (1.1 equiv), HOBt (1.2 equiv) and DIPEA (8.0 equiv) in a flame-dried round bottom flask equipped with a stir bar. After the flask was evacuated and backfilled with N_2_ twice, anhydrous DMF (0.2 M) was added via syringes. The resulting mixture was stirred at 25 °C for 8 h. H_2_O was added and the mixture was extracted with EtOAc (15 mL) three times. The combined organic layers were dried over anhydrous Na_2_SO_4_, concentrated *in vacuo* and the residue was further purified through a silica gel flash column to afford the **B1-B5** (40-62 % yield) as white solids.

The VHL ligand-HCl **E5** ((2S,4R)-1-((S)-2-amino-3,3-dimethylbutanoyl)-4-hydroxy-N-((S)-1-(4-(4-methylthiazol-5-yl)phenyl)ethyl)pyrrolidine-2-carboxamide hydrochloride) was prepared through a known literature procedure.^4^

The negative VHL ligand-HCl **E6** ((2S,4S)-1-((S)-2-amino-3,3-dimethylbutanoyl)-4-hydroxy-N-((S)-1-(4-(4-methylthiazol-5-yl)phenyl)ethyl)pyrrolidine-2-carboxamide hydrochloride) was purchased from LeYan.com.

**L5**-**L9** were prepared by following procedures from **LS5**-**LS9**, which are commercially available.

**General Procedure E.** To a flame-dried flask equipped with a stir bar was added **LS5-LS9** (1.0 equiv), Et_3_N (3.0 equiv), DMAP (0.1 equiv) and CH_2_Cl_2_. The flask was cooled to 0 °C on ice bath, and a solution of TsCl (1.5 equiv) in CH_2_Cl_2_ was added dropwise. After stirring for overnight at room temperature, the reaction was poured into water and CH_2_Cl_2_ was added. The organic layer was separated from the aqueous one, and the aqueous phase was extracted with CH_2_Cl_2_ 🞩 3. The combined organic layers were dried over anhydrous Na_2_SO_4_ and concentrated *in vacuo*. Purification by flash column chromatography afforded a colorless product (80-90 % yield).

***tert*-Butyl 2-(2-(tosyloxy)ethoxy)acetate (L5):** ^1^H NMR (400 MHz, DMSO-*d*_6_) δ 7.82 – 7.76 (m, 2H), 7.51 – 7.45 (m, 2H), 4.13 (t, *J* = 6.0 Hz, 2H), 3.93 (s, 2H), 3.65 (t, *J* = 6.1 Hz, 2H), 2.42 (s, 3H), 1.40 (s, 9H). LC-MS (ESI, m/z): calcd for C_15_H_23_O_6_S^+^, [M + H]^+^ 331.11, found 331.1.

***tert*-Butyl 2-(2-(2-(tosyloxy)ethoxy)ethoxy)acetate (L6):** ^1^H NMR (400 MHz, CDCl_3_) δ 7.80 (d, *J* = 8.3 Hz, 2H), 7.34 (d, *J* = 8.7 Hz, 2H), 4.19 – 4.14 (m, 2H), 3.97 (s, 2H), 3.72 – 3.67 (m, 2H), 3.66 – 3.59 (m, 4H), 2.44 (s, 3H), 1.47 (s, 9H). LC-MS (ESI, m/z): calcd for C_17_H_27_O_7_S^+^, [M + H]^+^ 375.14, found [M + H]^+^ 375.1.

***tert*-Butyl 2-(2-(2-(2-(tosyloxy)ethoxy)ethoxy)ethoxy)acetate (L7):** ^1^H NMR (400 MHz, CDCl_3_) δ 7.79 (d, *J* = 8.3 Hz, 2H), 7.34 (d, *J* = 8.1 Hz, 2H), 4.17 – 4.13 (m, 2H), 4.00 (s, 2H), 3.70 – 3.63 (m, 6H), 3.60 – 3.57 (m, 4H), 2.44 (s, 3H), 1.46 (s, 9H). LC-MS (ESI, m/z): calcd for C_19_H_31_O_8_S^+^, [M + H]^+^ 419.17, found 419.2.

***tert*-Butyl 14-(tosyloxy)-3,6,9,12-tetraoxatetradecanoate (L8):** ^1^H NMR (400 MHz, CDCl_3_) δ 7.81 – 7.74 (m, 2H), 7.35 – 7.29 (m, 2H), 4.13 (t, *J* = 6.0 Hz, 2H), 3.99 (s, 2H), 3.71 – 3.58 (m, 11H), 3.56 (s, 3H), 2.42 (s, 3H), 1.45 (s, 9H). LC-MS (ESI, m/z): calcd for C_21_H_35_O_9_S^+^, [M + H]^+^ 463.19, found 463.2.

***tert*-Butyl 17-(tosyloxy)-3,6,9,12,15-pentaoxaheptadecanoate (L9):** ^1^H NMR (400 MHz, DMSO-*d*_6_) δ 7.81 – 7.76 (m, 2H), 7.52 – 7.45 (m, 2H), 4.11 (t, *J* = 6.0 Hz, 2H), 3.97 (s, 2H), 3.58 – 3.54 (m, 4H), 3.53 – 3.47 (m, 10H), 3.44 (s, 4H), 2.42 (s, 3H), 1.41 (s, 9H). LC-MS (ESI, m/z): calcd for C_23_H_39_O_10_S^+^, [M + H]^+^ 507.22, found 507.2.

***tert*-Butyl 2-(2-(4-(4-phenylthiazol-2-yl)-4-((3-(5-(trifluoromethyl)-1,2,4-oxadiazol-3-yl)benzamido)methyl)piperidin-1-yl)ethoxy)acetate (S26):** ^1^H NMR (400 MHz, CDCl_3_) δ 8.50 (t, *J* = 1.9 Hz, 1H), 8.23 – 8.16 (m, 1H), 8.02 – 7.94 (m, 1H), 7.88 – 7.84 (m, 2H), 7.81 (s, 1H), 7.55 – 7.50 (m, 1H), 7.48 (s, 1H), 7.38 – 7.27 (m, 3H), 4.02 (s, 2H), 3.82 (d, *J* = 5.6 Hz, 2H), 3.71 – 3.62 (m, 2H), 2.74 (t, *J* = 8.7 Hz, 2H), 2.65 (t, *J* = 5.8 Hz, 4H), 2.32 (s, 2H), 2.25 – 2.15 (m, 2H), 1.48 (s, 9H). LC-MS (ESI, m/z): calcd for C_33_H_37_F_3_N_5_O_5_S^+^, [M + H]^+^ 672.24, found 672.2.

***tert*-Butyl 2-(2-(2-(4-(4-phenylthiazol-2-yl)-4-((3-(5-(trifluoromethyl)-1,2,4-oxadiazol-3-yl)benzamido)methyl)piperidin-1-yl)ethoxy)ethoxy)acetate (S27):** ^1^H NMR (400 MHz, CDCl_3_) δ 8.50 (t, *J* = 1.8 Hz, 1H), 8.20 (dt, *J* = 7.9, 1.3 Hz, 1H), 8.02 – 7.95 (m, 1H), 7.90 – 7.83 (m, 2H), 7.80 (s, 1H), 7.53 (t, *J* = 7.8 Hz, 1H), 7.48 (s, 1H), 7.37 – 7.28 (m, 3H), 4.01 (s, 2H), 3.84 (d, *J* = 5.6 Hz, 2H), 3.73 – 3.62 (m, 6H), 2.79 (t, *J* = 9.3 Hz, 2H), 2.67 (t, *J* = 5.8 Hz, 4H), 2.33 (d, *J* = 8.3 Hz, 2H), 2.15 – 2.05 (m, 2H), 1.46 (s, 9H). LC-MS (ESI, m/z): calcd for C_35_H_41_F_3_N_5_O_6_S^+^, [M + H]^+^ 716.27, found 716.3.

***tert*-Butyl 2-(2-(2-(2-(4-(4-phenylthiazol-2-yl)-4-((3-(5-(trifluoromethyl)-1,2,4-oxadiazol-3-yl)benzamido)methyl)piperidin-1-yl)ethoxy)ethoxy)ethoxy)acetate (S28):** ^1^H NMR (400 MHz, DMSO-*d*_6_) δ 8.81 (s, 1H), 8.45 (s, 1H), 8.19 (d, *J* = 7.8 Hz, 1H), 8.07 (d, *J* = 8.3 Hz, 2H), 7.93 (d, *J* = 7.1 Hz, 2H), 7.69 (t, *J* = 7.8 Hz, 1H), 7.38 (t, *J* = 7.5 Hz, 2H), 7.30 (t, *J* = 7.3 Hz, 1H), 3.96 (s, 2H), 3.57 – 3.47 (m, 14H), 3.39 – 3.33 (m, 6H), 2.42 – 2.27 (m, 2H), 2.20 – 1.97 (m, 2H), 1.40 (s, 9H). LC-MS (ESI, m/z): calcd for C_37_H_45_F_3_N_5_O_7_S^+^, [M + H]^+^ 760.29, found 760.3.

***tert*-Butyl 2-(2-(2-(2-(4-(4-phenylthiazol-2-yl)-4-((3-(5-(trifluoromethyl)-1,2,4-oxadiazol-3-yl)benzamido)methyl)piperidin-1-yl)ethoxy)ethoxy)ethoxy)acetate (S29):** ^1^H NMR (400 MHz, CDCl_3_) δ 8.50 (t, *J* = 1.9 Hz, 1H), 8.23 – 8.16 (m, 1H), 8.02 – 7.94 (m, 1H), 7.88 – 7.84 (m, 2H), 7.81 (s, 1H), 7.55 – 7.50 (m, 1H), 7.48 (s, 1H), 7.37 – 7.27 (m, 3H), 4.00 (s, 2H), 3.84 (d, *J* = 5.6 Hz, 2H), 3.71 – 3.59 (m, 14H), 2.74 (td, *J* = 8.7, 4.4 Hz, 2H), 2.63 (t, *J* = 5.8 Hz, 4H), 2.32 (s, 2H), 2.22 (s, 2H), 1.46 (s, 9H). LC-MS (ESI, m/z): calcd for C_39_H_49_F_3_N_5_O_8_S^+^, [M + H]^+^ 803.32, found 803.3.

***tert*-Butyl 17-(4-(4-phenylthiazol-2-yl)-4-((3-(5-(trifluoromethyl)-1,2,4-oxadiazol-3-yl)benzamido)methyl)piperidin-1-yl)-3,6,9,12,15-pentaoxaheptadecanoate (S30):** ^1^H NMR (400 MHz, DMSO-*d*_6_) δ 8.75 (t, *J* = 6.4 Hz, 1H), 8.44 (t, *J* = 2.0 Hz, 1H), 8.18 (dt, *J* = 7.8, 1.0 Hz, 1H), 8.08 – 8.02 (m, 2H), 7.92 (dq, *J* = 7.7, 1.6 Hz, 2H), 7.68 (t, *J* = 7.8 Hz, 1H), 7.41 – 7.33 (m, 2H), 7.32 – 7.25 (m, 1H), 3.96 (s, 2H), 3.57 – 3.42 (m, 20H), 2.82 (d, *J* = 11.2 Hz, 2H), 2.42 (s, 2H), 2.30 (d, *J* = 13.4 Hz, 2H), 2.18 (d, *J* = 10.1 Hz, 2H), 2.03 – 1.92 (m, 2H), 1.40 (q, *J* = 1.8 Hz, 9H). LC-MS (ESI, m/z): calcd for C_41_H_53_F_3_N_5_O_9_S^+^, [M + H]^+^ 848.34, found 848.3.

**B1** was prepared from **S26** and **E5** on a 0.041 mmol scale by following the **General Procedure D**. It was purified through column chromatography (hexanes/EtOAc: from 4:1 to 1:100, then EtOAc/MeOH: from 100:1 to 30:1) as a white solid (31 mg, 72 % yield).

**(2S,4R)-1-((S)-3,3-dimethyl-2-(2-(2-(4-(4-phenylthiazol-2-yl)-4-((3-(5-(trifluoromethyl)-1,2,4-oxadiazol-3-yl)benzamido)methyl)piperidin-1-yl)ethoxy)acetamido)butanoyl)-4-hydroxy-N-((S)-1-(4-(4-methylthiazol-5-yl)phenyl)ethyl)pyrrolidine-2-carboxamide (B1):** ^1^H NMR (400 MHz, DMSO-*d*_6_) δ 8.97 (s, 1H), 8.75 (t, *J* = 6.3 Hz, 1H), 8.49 – 8.39 (m, 2H), 8.18 (dt, *J* = 7.8, 1.4 Hz, 1H), 8.07 – 8.03 (m, 2H), 7.94 – 7.90 (m, 2H), 7.72 – 7.65 (m, 2H), 7.44 – 7.41 (m, 2H), 7.39 – 7.33 (m, 5H), 7.30 – 7.26 (m, 1H), 4.90 (p, *J* = 6.9 Hz, 1H), 4.53 (d, *J* = 9.6 Hz, 1H), 4.45 (t, *J* = 8.2 Hz, 1H), 4.32 – 4.25 (m, 1H), 3.97 – 3.89 (m, 2H), 3.60 – 3.53 (m, 6H), 2.95 – 2.78 (m, 2H), 2.49 – 2.45 (m, 2H), 2.45 (s, 3H), 2.31 (d, *J* = 12.5 Hz, 2H), 2.17 (t, *J* = 7.4 Hz, 2H), 2.07 – 1.97 (m, 3H), 1.82 – 1.72 (m, 1H), 1.35 (d, *J* = 7.0 Hz, 3H), 0.93 (s, 9H). ^13^C NMR (100 MHz, DMSO-*d*_6_) δ 170.7, 169.2, 168.7, 168.3, 167.2, 165.9, 154.0, 151.7, 148.0, 144.9, 136.0, 134.6, 131.9, 131.8, 131.5, 131.3, 130.0, 129.9, 129.8, 129.0, 128.9, 128.8, 128.0, 126.5, 126.2, 124.8, 114.5, 69.7, 69.0, 67.6, 58.8, 57.4, 56.7, 55.8, 50.4, 50.3, 48.8, 48.0, 44.8, 38.3, 36.0, 33.8, 29.2, 26.4, 22.6, 16.2. LC-MS (ESI, m/z): calcd for C_52_H_59_F_3_N_9_O_7_S_2_^+^, [M + H]^+^ 1042.39, found 1042.4.

**B2** was prepared from **S27** and **E5** on a 0.0516 mmol scale by following the **General Procedure D**. It was purified through column chromatography (hexanes/EtOAc: from 4:1 to 1:100, then EtOAc/MeOH: from 100:1 to 30:1) as a white solid (37 mg, 66 % yield).

**(2S,4R)-1-((S)-3,3-dimethyl-2-(2-(2-(2-(4-(4-phenylthiazol-2-yl)-4-((3-(5-(trifluoromethyl)-1,2,4-oxadiazol-3-yl)benzamido)methyl)piperidin-1-yl)ethoxy)ethoxy)acetamido)butanoyl)-4-hydroxy-N-((S)-1-(4-(4-methylthiazol-5-yl)phenyl)ethyl)pyrrolidine-2-carboxamide (B2):** ^1^H NMR (400 MHz, DMSO-*d*_6_) δ 8.99 (s, 1H), 8.96 (s, 1H), 8.50 – 8.44 (m, 2H), 8.19 (dt, *J* = 7.8, 1.5 Hz, 1H), 8.15 – 8.09 (m, 2H), 7.95 – 7.92 (m, 2H), 7.69 (t, *J* = 7.8 Hz, 1H), 7.43 – 7.30 (m, 9H), 4.88 (p, *J* = 6.9 Hz, 1H), 4.54 (d, *J* = 9.6 Hz, 1H), 4.44 (t, *J* = 8.3 Hz, 1H), 4.30 – 4.25 (m, 1H), 3.95 (s, 2H), 3.78 – 3.72 (m, 2H), 3.65 – 3.52 (m, 10H), 3.32 – 3.06 (m, 3H), 2.98 – 2.74 (m, 2H), 2.44 (s, 3H), 2.42 – 2.25 (m, 3H), 2.10 – 2.03 (m, 1H), 1.79 – 1.72 (m, 1H), 1.35 (d, *J* = 7.0 Hz, 3H), 0.91 (s, 9H). ^13^C NMR (100 MHz, DMSO-*d*_6_) δ 170.6, 169.2, 168.7, 168.3, 166.0, 151.7, 148.0, 144.9, 135.7, 134.4, 131.6, 130.2, 129.9, 129.9, 129.0, 128.8, 128.1, 126.5, 126.5, 126.2, 124.8, 114.6, 70.3, 69.7, 69.6, 69.0, 58.8, 56.8, 55.9, 48.0, 38.0, 36.0, 31.2, 28.7, 26.4, 22.7, 16.2. LC-MS (ESI, m/z): calcd for C_54_H_63_F_3_N_9_O_8_S_2_^+^, [M + H]^+^ 1086.41, found 1086.4.

**B3** was prepared from **S28** and **E5** on a 0.0354 mmol scale by following the **General Procedure D**. It was purified through column chromatography (hexanes/EtOAc: from 4:1 to 1:100, then EtOAc/MeOH: from 100:1 to 30:1) as a white solid (28 mg, 70 % yield).

**(2S,4R)-1-((S)-2-(*tert*-butyl)-4-oxo-14-(4-(4-phenylthiazol-2-yl)-4-((3-(5-(trifluoromethyl)-1,2,4-oxadiazol-3-yl)benzamido)methyl)piperidin-1-yl)-6,9,12-trioxa-3-azatetradecanoyl)-4-hydroxy-N-((S)-1-(4-(4-methylthiazol-5-yl)phenyl)ethyl)pyrrolidine-2-carboxamide (B3):** ^1^H NMR (400 MHz, DMSO-*d*_6_) δ 8.98 (s, 1H), 8.93 (s, 1H), 8.52 – 8.42 (m, 2H), 8.22 – 8.09 (m, 3H), 7.95 (d, *J* = 7.5 Hz, 2H), 7.70 (t, *J* = 7.7 Hz, 1H), 7.45 – 7.28 (m, 9H), 4.89 (p, *J* = 7.2 Hz, 1H), 4.54 (d, *J* = 9.4 Hz, 1H), 4.44 (t, *J* = 8.1 Hz, 1H), 4.32 – 4.23 (m, 1H), 3.96 (s, 2H), 3.78 – 3.69 (m, 2H), 3.62 – 3.49 (m, 14H), 3.31 – 3.09 (m, 3H), 3.06 – 2.77 (m, 2H), 2.45 (s, 3H), 2.42 – 2.28 (m, 3H), 2.10 – 2.02 (m, 1H), 1.81 – 1.71 (m, 1H), 1.36 (d, *J* = 7.0 Hz, 3H), 0.93 (s, 9H). ^13^C NMR (100 MHz, DMSO-*d*_6_) δ 170.7, 169.2, 168.7, 168.3, 166.1, 151.7, 148.0, 144.9, 135.7, 134.4, 131.6, 131.3, 130.2, 129.9, 129.0, 128.9, 128.2, 126.5, 126.5, 126.2, 124.8, 70.6, 69.8, 69.0, 58.8, 56.8, 55.9, 55.1, 48.0, 38.0, 36.0, 29.2, 26.4, 22.7, 16.2. LC-MS (ESI, m/z): calcd for C_56_H_67_F_3_N_9_O_9_S_2_^+^, [M + H]^+^ 1130.44, found 1130.4.

**B4** was prepared from **S29** and **E5** on a 0.063 mmol scale by following the **General Procedure D**. It was purified through column chromatography (hexanes/EtOAc: from 4:1 to 1:100, then EtOAc/MeOH: from 100:1 to 30:1) as a white solid (51 mg, 69 % yield).

**(2S,4R)-1-((S)-2-(*tert*-butyl)-4-oxo-17-(4-(4-phenylthiazol-2-yl)-4-((3-(5-(trifluoromethyl)-1,2,4-oxadiazol-3-yl)benzamido)methyl)piperidin-1-yl)-6,9,12,15-tetraoxa-3-azaheptadecanoyl)-4-hydroxy-N-((S)-1-(4-(4-methylthiazol-5-yl)phenyl)ethyl)pyrrolidine-2-carboxamide (B4):** ^1^H NMR (400 MHz, DMSO-*d*_6_) δ 8.98 (s, 1H), 8.79 – 8.73 (m, 1H), 8.47 – 8.42 (m, 2H), 8.20 – 8.15 (m, 2H), 8.07 – 8.04 (m, 2H), 7.94 – 7.90 (m, 2H), 7.68 (t, *J* = 7.8 Hz, 1H), 7.44 – 7.41 (m, 2H), 7.39 – 7.34 (m, 5H), 7.30 – 7.26 (m, 1H), 4.91 (p, *J* = 7.1 Hz, 1H), 4.54 (d, *J* = 9.6 Hz, 1H), 4.44 (t, *J* = 8.1 Hz, 1H), 4.30 – 4.26 (m, 1H), 3.94 (s, 2H), 3.60 – 3.47 (m, 18H), 2.87 – 2.81 (m, 2H), 2.45 (s, 5H), 2.30 (d, *J* = 12.3 Hz, 2H), 2.18 (t, *J* = 10.8 Hz, 2H), 2.07 – 1.94 (m, 3H), 1.81 – 1.73 (m, 1H), 1.36 (d, *J* = 7.0 Hz, 3H), 0.93 (s, 9H). ^13^C NMR (100 MHz, DMSO-*d*_6_) δ 170.7, 169.2, 168.7, 168.3, 167.2, 165.9, 154.0, 151.7, 148.0, 144.9, 135.9, 134.5, 131.9, 131.8, 131.5, 131.3, 130.1, 129.9, 129.8, 129.0, 128.9, 128.8, 128.0, 126.5, 126.2, 124.8, 69.6, 69.0, 67.6, 58.8, 56.7, 55.9, 47.9, 38.3, 37.9, 35.9, 33.8, 31.5, 30.0, 29.2, 28.9, 28.6, 26.4, 23.4, 22.7, 22.6, 22.3, 16.2. HR-MS (ESI, m/z): calcd for C_58_H_71_F_3_N_9_O_10_S_2_^+^, [M + H]^+^ 1174.4644, found 1174.4670.

**B5** was prepared from **S30** and **E5** on a 0.0424 mmol scale by following the **General Procedure D**. It was purified through column chromatography (hexanes/EtOAc: from 4:1 to 1:100, then EtOAc/MeOH: from 100:1 to 30:1) as a white solid (32 mg, 62 % yield).

**(2S,4R)-1-((S)-2-(*tert*-butyl)-4-oxo-20-(4-(4-phenylthiazol-2-yl)-4-((3-(5-(trifluoromethyl)-1,2,4-oxadiazol-3-yl)benzamido)methyl)piperidin-1-yl)-6,9,12,15,18-pentaoxa-3-azaicosanoyl)-4-hydroxy-N-((S)-1-(4-(4-methylthiazol-5-yl)phenyl)ethyl)pyrrolidine-2-carboxamide (B5):** ^1^H NMR (400 MHz, DMSO-*d*_6_) δ 8.98 (s, 1H), 8.85 (s, 1H), 8.48 – 8.43 (m, 2H), 8.20 (d, *J* = 7.8 Hz, 1H), 8.13 – 8.06 (m, 2H), 7.94 (d, *J* = 6.9 Hz, 2H), 7.69 (t, *J* = 7.8 Hz, 1H), 7.46 – 7.27 (m, 9H), 4.90 (p, *J* = 7.1 Hz, 1H), 4.54 (d, *J* = 9.5 Hz, 1H), 4.44 (t, *J* = 8.1 Hz, 1H), 4.30 – 4.26 (m, 1H), 3.95 (s, 2H), 3.61 – 3.48 (m, 22H), 2.87 (d, *J* = 15.1 Hz, 1H), 2.72 (d, *J* = 15.3 Hz, 1H), 2.45 (s, 3H), 2.42 – 2.31 (m, 2H), 2.24 – 2.09 (m, 2H), 2.08 – 2.03 (m, 1H), 1.81 – 1.73 (m, 1H), 1.59 – 1.45 (m, 4H), 1.37 (d, *J* = 7.1 Hz, 3H), 0.93 (s, 9H). ^13^C NMR (100 MHz, DMSO-*d*_6_) δ 170.7, 169.5, 169.2, 168.7, 168.3, 166.0, 154.0, 151.7, 148.0, 144.9, 135.8, 134.5, 131.5, 131.3, 130.2, 129.9, 129.9, 129.0, 128.8, 128.1, 126.5, 126.2, 124.8, 73.1, 70.6, 70.1, 70.0, 70.0, 69.9, 69.8, 69.0, 64.7, 64.0, 58.8, 56.7, 55.9, 55.1, 48.0, 43.3, 38.0, 36.0, 30.3, 30.2, 26.4, 22.7, 18.8, 16.2. LC-MS (ESI, m/z): calcd for C_60_H_75_F_3_N_9_O_11_S_2_^+^, [M + H]^+^ 1218.49, found 1218.5.

**B6** was prepared from **S29** and **E6** on a 0.0288 mmol scale by following the **General Procedure D**. It was purified through column chromatography (hexanes/EtOAc: from 4:1 to 1:100, then EtOAc/MeOH: from 100:1 to 30:1) as a white solid (25 mg, 65 % yield).

**(2S,4S)-1-((S)-2-(*tert*-butyl)-4-oxo-17-(4-(4-phenylthiazol-2-yl)-4-((3-(5-(trifluoromethyl)-1,2,4-oxadiazol-3-yl)benzamido)methyl)piperidin-1-yl)-6,9,12,15-tetraoxa-3-azaheptadecanoyl)-4-hydroxy-N-((S)-1-(4-(4-methylthiazol-5-yl)phenyl)ethyl)pyrrolidine-2-carboxamide (B6):** ^1^H NMR (400 MHz, DMSO-*d*_6_) δ 8.98 (s, 1H), 8.76 (t, *J* = 6.5 Hz, 1H), 8.44 (d, *J* = 7.6 Hz, 2H), 8.20 – 8.16 (m, 2H), 8.06 (d, *J* = 5.9 Hz, 2H), 7.92 (d, *J* = 7.0 Hz, 2H), 7.68 (t, *J* = 7.8 Hz, 1H), 7.44 – 7.41 (m, 2H), 7.39 – 7.35 (m, 5H), 7.30 – 7.26 (m, 1H), 4.90 (t, *J* = 7.2 Hz, 1H), 4.54 (d, *J* = 9.6 Hz, 1H), 4.44 (t, *J* = 8.1 Hz, 1H), 4.28 (s, 1H), 3.94 (s, 2H), 3.61 – 3.48 (m, 18H), 2.84 (d, *J* = 12.4 Hz, 2H), 2.46 – 2.43 (m, 5H), 2.30 (d, *J* = 14.5 Hz, 2H), 2.23 – 2.16 (m, 2H), 2.05 – 1.94 (m, 3H), 1.76 (ddd, *J* = 13.0, 8.9, 4.5 Hz, 1H), 1.36 (d, *J* = 7.0 Hz, 3H), 0.93 (s, 9H). LC-MS (ESI, m/z): calcd for C_58_H_71_F_3_N_9_O_10_S_2_^+^, [M + H]^+^ 1174.46, found 1174.5.

**C．Methods and Protocols** **for Biological Studies**

**(1) Bioinformatics analysis**

Transcriptome datasets for two autoimmune diseases (SLE, GSE616352 and SCAP, GSE1963993) were downloaded from the Gene Expression Omnibus (GEO) database. Gene differential expression analyses were performed using the Limma software package, grouped by disease and control, and the data were normalized using the built-in function normalizeBetweenArrays.

**(2) Cell lines and cell culture**

293T and RAW264.7 cell lines purchased from the Shanghai Institute of Biochemistry and Cell Biology (Shanghai, China) and cultured in DMEM supplemented with 10% FBS. These two cell lines were authenticated by short tandem repeat (STR) profiling every 6 months. Bone marrow-derived macrophages (BMDM) were obtained from 7-8 weeks old mice bone marrow and treated with macrophage colony-stimulating factor (M-CSF) at a concentration of 40 ng/mL for 5 days. After 5 days induction, the adhered cells (BMDMs) were resuspended and seeded in plate and treated with different compounds. All cell lines were maintained at 37 ℃ in a 5% CO_2_ incubator and monitored for mycoplasma contamination every month.

**(3) Western blotting analysis**

Cells and murine macrophages (RAW264.7, BMDMs) with a density of 2×10^5^ cells/mL were seeded in 24-well plates and stabilized for 12 h. Then, the cells were treated with compounds for 12 h. At the end point of experiment, the cells were first washed with cold phosphate-buffered saline (PBS) and then lysed with 100 μL loading buffer containing Tris (20 mM, pH 6.8), SDS (4% w/v), glycerol (16% v/v), DTT (3% w/v), and bromophenol blue (0.02% w/v). Subsequently, the lysate was incubated at 95 ℃ for 30 min. The cell lysate containing equal amounts of proteins were separated by sodium dodecylsulfate-polyacrylamide gel electrophoresis (SDS-PAGE) and the proteins were transferred to PVDF membranes. The membranes were blocked using 5% skim milk for 1 h at room temperature and incubated with primary antibody overnight at 4 ℃. After washing three times with PBS-T, the membranes were incubated with secondary antibodies (from Ford Biotechnology) at room temperature for 1 h. The immunoreactive bands were then visualized using ECL and analyzed using the Amersham ImageQuant 800 system (cytiva, 29399481). The primary antibody used in this study are showed in **Table S1** below.

**Table S1.** Primary antibody used in this study

| **Primary antibody** | **Manufacturer** | **Cat.#** |
| --- | --- | --- |
| Anti-HDAC4 Antibody (for HEK293T) | HUABIO | ET1612-51 |
| Anti-HDAC4 Antibody (for RAW264.7) | ABclonal | A0239 |
| Anti-HDAC5 Antibody (for HEK293T) | ABclonal | A7189 |
| Anti-HDAC5 Antibody (for RAW264.7) | Cell signaling technology | 98329 |
| Anti-HDAC7 Antibody | Cell signaling technology | 33418 |
| Anti-HDAC9 Antibody (for HEK293T) | HUABIO | ET1706-36 |
| Anti-HDAC9 Antibody (for RAW264.7) | HUABIO | ER63671 |
| Anti-HDAC8 Antibody | Diagbio | db11765 |
| Anti-CRBN Antibody | Cell signaling technology | 71810 |
| Anti-VHL Antibody | Cell signaling technology | 68547 |
| Anti-c-Jun Antibody | HUABIO | ET1608-3 |
| Anti-Phospho-c-Jun(S63) Antibody | HUABIO | ET1608-4 |
| Anti-JNK1/2/3 Antibody | HUABIO | ET1601-28 |
| Anti-Phospho-JNK1/JNK2/JNK3 Antibody | Diagbio | db3253 |
| Anti-NF-kB p65 Antibody | Diagbio | db3141 |
| Anti-Phospho-NF-kB p65 (Ser536) Antibody | Diagbio | db7996 |
| Anti-TRAF6 Antibody | Diagbio | db12997 |
| Anti-TAK1 Antibody | Diagbio | db12332 |
| Anti-Ubiquitin Antibody | Diagbio | db935 |
| Anti-GAPDH Antibody | Diagbio | db106 |
| Anti-Flag Antibody | AbHO | HOA012FL01 |
| Anti-Acetylated-Lysine Antibody | Cell signaling technology | 9441 |

**(4) HDAC7 *in vitro* and in cells assays**

Full-length human HDAC7 fused with C-terminal His tag were recombinantly expressed in Sf9 insect cells and then were purified by His Trap^TM^ HP and using ӒKTA pure^TM^ system. The *in-vitro* kinase assay was performed in assay buffer (1M Tris-HCl, 4 M NaCl) at 30 ℃ using 0.1 μM purified HDAC7 enzyme and 50 μM Ac- Leu-Gly-Lys (TFAc)-AMC substrate. For the in-cell kinase assay, RAW264.7 cells were treated with 5 μM **TMP269** for 12 h and lysed with RIPA buffer (25 mM Tris-HCl, 150 mM NaCl, 1% NP-40, 1% sodium deoxycholate, 0.1% SDS, pH 7.4). Subsequently, 5 μM **TMP269** was added to the cell lysate during the enzyme reaction. The kinase activity of HDAC7 was represented by the fluorescence of 7-amino-4-methylcoumarin released by 0.5 μg/mL Trypsin.

**(5) Real-time quantitative PCR**

Total mRNA was extracted using the PureLink RNA extraction kit (Thermo Fisher, 12183018A). A total of 1 μg of mRNA was then reverse transcribed into cDNA using the cDNA synthesis kit (Transgen, AT311-03). The quantitative Real-Time PCR Analysis System (Roche) was used for qRT-PCR, utilizing the iTaq Universal SYBR Green qPCR supermixes (Bio-Rad, L001752B). The relative expression values for each gene of interest were determined by normalizing them to *GAPDH* mRNA expression. The specific primer pairs for each gene are listed in **Table S2** below.

**Table S2.** Gene specific primer pairs

| **genes** | **Forward primers** | **Reverse primers** |
| --- | --- | --- |
| *Il6* | CAATTCCAGAAACCGCT | GCAAGTGCATCATCGT |
| *Il1b* | TTCAGGCAGGCAGTATCACTC | GAAGGTCCACGGGAAAGACAC |
| *Csf2* | TCGTCTCTAACGAGTTCTCCTT | CGTAGACCCTGCTCGAATATCT |
| *Gapdh* | AAGAAGGTGGTGAAGCAGGCATC | CGCGATCGAAGGTGGAAGAGTG |

**(5)** **ELISA assays for cytokine quantification**

RAW264.7 and BMDM cells were seeded in 24-well plates at a density of 2×10^5^ cells/mL and incubated for 12 h. After treated and stimulated with the tested compounds and LPS, as indicated in individual figure legends, the supernatant was collected for measuring the level of cytokines by using commercial ELISA kits (BIOKER). Firstly, the ELISA plate is coated with capture antibody in a Coating Buffer and sealed. It is then incubated overnight at 4 °C. Next, the wells were blocked using 200 µL of ELISA/ELISPOT Diluent and incubated at room temperature for 2 h. After incubation, set up the standard curve and add 100 µL/well of samples. Incubate them for 2 h at room temperature or overnight at 4 °C. Next, add the diluted Detection Antibody to each well and incubate the plate at room temperature for 1 h. After that, Streptavidin-HRP was added at a volume of 100 µL/well, and the plate was incubated at room temperature for 30 min. Then, the plate was incubated with TMB solution at room temperature for 15 min. Finally, the reaction was stopped by adding 1 M H_2_SO_4_. The absorbance was measured at 450 nm (reference wavelength: 570 nm) using a microplate reader (TECAN, 2102004761).

**(6)** **Cytokines array analysis**

The cytokines secreted by RAW264.7 cells were analyzed using the Mouse Inflammation Antibody Array-Membrane Kit (Abcam, ab133999) according to the manufacturer's instructions. In brief, block the Antibody-Array membranes by incubating them with 2 mL Blocking buffer at room temperature for 30 min. Then, incubate the membranes with the cell culture supernatant-antibody samples overnight at 4 ℃. The array membranes were washed with wash buffer I and wash buffer II provided in the kit and then incubated with Biotin-Conjugated Anti-Cytokines for 2 h at room temperature. Next, pipette HRP-Conjugated into each well and incubate for 2 h at room temperature. Finally, detect chemiluminescence using the Amersham ImageQuant 800 system (cytiva, 29399481) after reaching the dot blots with detection buffers. The inflammation cytokines lists are showed in **Table S3** below.

**Table S3.** Lists of inflammation cytokines

|  | 1 2 | 3 4 | 5 6 | 7 8 |
| --- | --- | --- | --- | --- |
| A | Pos | GM-CSF | IL-13 | RANTES |
| B | Pos | IFN-γ | IL-17 | SDF-1 |
| C | Neg | IL-1α | I-TAC | TCA-3 |
| D | Neg | IL-1β | KC | TECK |
| E | Blank | IL-2 | Leptin | TIMP-1 |
| F | BCL | IL-7 | LIX | TIMP-2 |
| G | CD30L | IL-4 | Lymphotactin | TNF-α |
| H | Eotaxin | IL-6 | MCP-1 | sTNF RI |
| I | Eotaxin-2 | IL-9 | MCSF | sTNF RII |
| J | Fas-ligand | IL-10 | MIG | Blank |
| K | Fractalkine | IL-12 p40/70 | MIP-1α | Blank |
| L | GCSF | IL-12 p70 | MIP-1γ | Pos |

**(7)** **Pulldown of total ubiquitinylated proteins with Ni-NTA Beads**

293T cells (2×10^6^ cells/dish) were co-transfected with the indicated plasmids (HDAC7-Flag and His-ubiquitin) using Jetprime transfection reagents. At 24 h post-transfection, the media was changed, and cells were co-treated with PROTACs or **TMP269** for 12 h. In addition, 20µM MG132 was added 8 h before collecting the cells. Cells were washed with PBS and lysed with 8 M urea buffer (10 mM Tris-HCl pH 8.0; 100 mM NaH_2_PO_4_; 8 M urea) containing 10 mM imidazole. The cell lysate was incubated with Ni-NTA Beads (Smart-Lifesciences, SA004005) overnight at 4 ℃. The beads were then washed five times with wash buffer (10 mM Tris-HCl pH 6.3, 100 mM NaH_2_PO_4_, 8 M urea) containing 20 mM imidazole. Protein elution was performed using 2× loading buffer (20 mM Tris-base, 4% (w/v) SDS, 16% (w/v) glycerol, 3% (w/v) DTT, and 0.02% (w/v) bromophenol blue, pH 6.8) at 95 ℃ for 10 min. Finally, 20 μg of proteins were subjected to Western blotting analysis.

**(8)** **Generation of CRBN/VHL-knockout cell lines using CRISPR Cas9 gene editing technology**

The *CRBN*-knockout (KO) and *VHL*-knockout cells were generated using CRISPR-cas9 technology. CRISPR gRNAs were designed by http://crispor.tefor.net/. The annealed sgRNA targeting *CRBN* or *VHL* were inserted into Bbs1-digested pSpCas9(BB)−2A-GFP (PX458) plasmid (Addgene plasmid #48138) to generate PX458-*CRBN* gRNAs and PX458-*VHL* gRNAs. The sgRNA sequences could be found in **Table S4** below. To generate *CRBN*/*VHL* KO 293T cells, the wild-type 293T cells was transfected gRNAs targeting *CRBN* or *VHL* with Jetprime transfection reagents. The flow cytometry technique was used to isolate the single cell, which was then seeded in 96-well plates. The genomic DNA of each individual cell clone was extracted using the TIANamp Genomic DNA kit (TIANGEN Biotech, DP304-02) and identified through gene sequencing.

**Table S4.** sgRNA sequences

| **SgRNA** | **Forward primers** | **Reverse primers** |
| --- | --- | --- |
| sgCRBN #1 | CACCGACCAATGTTCATATAAATGG | AAACCCATTTATATGAACATTGGTC |
| sgCRBN #2 | CACCGCTGACTGTGTTCTTAGCTCA | AAACTGAGCTAAGAACACAGTCAGC |
| sgCRBN #3 | CACCGTTCTAATTGAACTGCAGACA | AAACTGTCTGCAGTTCAATTAGAAC |
| sgVHL #1 | CACCGGAGATGCAGGGACACACGAT | AAACATCGTGTGTCCCTGCATCTCC |
| sgVHL #2 | CACCGCGCCGCATCCACAGCTACCG | AAACCGGTAGCTGTGGATGCGGCGC |
| sgVHL #3 | CACCGCCCGTATGGCTCAACTTCGA | AAACTCGAAGTTGAGCCATACGGGC |

**(9)** **siHDACs assay**

RAW264.7 cells were seeded with a density of 5×10^3^ cells/mL in a 24-well plate and cultivated for 12 h. Mix siRNA against HDAC4, 5, 7, 9 or scrambled control oligonucleotides and lipo3000 in transfection reagent (Thermo Fisher Scientific) and then transfect them to RAW264.7 cells. Cells were incubated with siRNA over a period of 48 h before incubation with LPS for another 24 h. The sequence of siRNA is showed in **Table S5** below.

**Table S5.** siRNA sequences

| **Gene** | **Sequences** |
| --- | --- |
| siHDAC4#1 | GCAGTTCCTGGAGAAGCACAAGCAA |
| siHDAC4#2 | TCCTGGAGAAGCACAAGCAACAGTT |
| siHDAC5#1 | GACGCCTCCCTCCTACAAATT |
| siHDAC5#2 | CATCGCTGAGAACGGCTTTAC |
| siHDAC7#1 | CTTCGGCAACTTCTCAATAAA |
| siHDAC7#2 | GCTGAAGTGATCCTGAAGAAA |
| siHDAC9#1 | GCTCCAGGATTTGTAATTAAA |
| siHDAC9#2 | GAAAGAATTTCACCAGGCATT |

**(10)** **Global Proteomics Analysis**

The RAW264.7 and 293T cells were individually treated with B4 or DMSO before being rapidly frozen by liquid nitrogen. Quantitative proteomics analysis was performed by Jingjie PTM-Biolab company (Hangzhou, China). The quantitative mass spectrometry-based proteomics analysis for HDACs is summarized in **Table S6** and **Table S7** in a separate file named as **Supplemental Tables**.

**(11)** **RNA-sequencing analysis**

Total RNA was isolated and purified using TRIzol reagent (Invitrogen, Carlsbad, CA, USA) following the manufacturer's procedure. RNA-sequencing was completed by Hangzhou Lianchuan Biotechnology Ltd. The RNA amount and purity of each sample was quantified using NanoDrop ND-1000 (NanoDrop, Wilmington, DE, USA). The RNA integrity was assessed by Bioanalyzer 2100 (Agilent, CA, USA) with RIN number >7.0, and confirmed by electrophoresis with denaturing agarose gel. Poly (A) RNA was purified from 1 μg total RNA using Dynabeads Oligo (dT) 25-61005 (Thermo Fisher, CA, USA) through two rounds of purification. The purified poly (A) RNA was then fragmented into small pieces under 94 ℃ for 5-7 min using the Magnesium RNA Fragmentation Module (NEB, cat. e6150, USA). Next, the cleaved RNA fragments were reverse-transcribed by SuperScript™ II Reverse Transcriptase (Invitrogen, cat. 1896649, USA) to generate cDNA. The cDNA was subsequently used to synthesize U-labeled second-stranded DNAs with the assistance of E. coli DNA polymerase I (NEB, cat.m0209, USA), RNase H (NEB, cat.m0297, USA), and dUTP Solution (Thermo Fisher, cat. R0133, USA). Preparing them for ligation to the indexed adapters, an A-base is added to the blunt ends of each strand. The adapters, which contain a T-base overhang, are then ligated to the A-tailed fragmented DNA. Following this, the fragments are ligated to single- or dual-index adapters. Size selection is performed with AMPureXP beads. The second-stranded DNAs labeled with U are first treated with the heat-labile UDG enzyme (NEB, cat.m0280, USA). Following this treatment, the ligated products undergo PCR amplification. The PCR conditions consist of an initial denaturation at 95 ℃ for 3 min. This is followed by 8 cycles of denaturation at 98 ℃ for 15 second, annealing at 60 ℃ for 15 second, and extension at 72 ℃ for 30 second. Finally, a final extension is carried out at 72 ℃ for 5 min. The resulting cDNA library has an average insert size of 300 ± 50 bp. To complete the sequencing process, we performed 2×150 bp paired-end sequencing (PE150) on an Illumina Novaseq™6000 (LC-Biotechnology CO, Ltd., Hangzhou, China). This sequencing was conducted in accordance with the vendor’s recommended protocol.

**(12)** **ATAC-sequencing analysis**

ATAC-seq was conducted following the ATAC-seq protocol by Shanghai Jiayin Biotechnology Ltd. Briefly, cells were harvested from cell culture and lysed using a lysis buffer. The transposition step was performed using the Nextera DNA Library Preparation Kit (Illumina), following the manufacturer's instructions. A total of 50,000 nuclei were pelleted and then resuspended with transposase at 37 ℃ for 30 min. The resulting transposed DNA fragments were promptly purified using a MinElute PCR Purification Kit (Qiagen). Samples were PCR-amplified using NEBNext High-Fidelity PCR Master Mix (New England Biolabs, MA). The amplified libraries were purified with the MinElute PCR Purification Kit (Qiagen) and sequenced using PE150 on the Illumina Novaseq 6000.

Raw data (raw reads) of fastq format underwent initial processing using in-house perl scripts. In this step, reads containing adapter sequences, reads containing ploy-N, and low-quality reads were removed from the raw data to obtain clean data (clean reads). Simultaneously, Q20, Q30, and the GC content of the clean data were calculated. Subsequently, all subsequent analyses were conducted using the high-quality clean data. Pair-end reads were mapped after obtaining clean reads by removing adaptor sequences from the raw reads. The clean reads were aligned to reference genome sequences using the bwa program. Visualization of the ATAC-seq data were analyzed by Integrative Genomics Viewers (IGV).

**(13)** **Co-immunoprecipitation**

pCDH-HDAC7-Flag was transfected into 293T cells using Jetprime transfection reagents. After 24 h transfection, the cells were treated with PROTAC or TMP269 for 12 h. The transfected cells were washed with ice-cold PBS twice and lysed using RIPA lysis buffer containing (50 mM Tris-base, 150 mM Nacl, 5 mM EDTA, 0.1% SDS, 1% TritonX-100, 0.25% Sodium deoxycholate, pH 7.4), then centrifuged at 13,000 g for 30 min. The cell lysate was incubated with Flag beads overnight at 4 ℃. The complex was washed with T-PBS five times and suspended in the loading buffer and followed by western blot analysis.

1. **VHL recombinant protein expression and purification**

The pACYC-Duet1-Elongin B/C (#110274) and VHL-pGex2TK (#20790) plasmids were purchased from Addgene. The two plasmids were then co-transformed into *E. coli* BL21(DE3)-RIPL, and positive clones were screened by LB agar plates (1% NaCl, 1% Tryptone, 0.5% Yeast Extract, 1.5% agar pH 7.0) containing 50 µg/mL Kanamycin, 100 µg/mL Carbenicillin, and 30 µg/mL Chloramphenicol antibiotics. The *E. coli* was growing at 37 °C in LB broth until the OD600 reached 0.4–0.6. Isopropyl-β-D-thiogalactoside (IPTG, 0.4 mM) (V900917, Sigma-Aldrich) was then added, and bacteria were further incubated at 16 °C for 16 h. After centrifugation at 3,000 rcf for 10 min at 4 °C, the bacteria were resuspended in lysis buffer (30 mM Tris-HCl pH 8.0, 200 mM NaCl, 5 mM DTT, 5% glycerol, 1× EDTA-free Protease Inhibitor Cocktail). The bacteria were homogenized using a high-pressure cell crusher (Union Bio-tech, UX450) and centrifuged at 20,000g for 1 h at 4 °C. After filtration with the 0.22 μm microporous filter membrane, the supernatant was loaded onto the GST affinity column (cytiva, 17528101). The protein was eluted with elution buffer (50 mM Tris-HCl pH 8.0, 200 mM NaCl, 1 mM DTT, 10 mM glutathione). The purified protein was dialyzed with PBS (pH 8.0) buffer containing 20% glycerinum.

1. **Ternary complex formation assay**

293T cells (2×10^6^ cells/dish) were co-transfected with the indicated plasmids (HDAC7-Flag or HDAC9-Flag) using Jetprime transfection reagents (Polyplus, 101000046). 24 h after transfection, the cells were collected and lysed using 1% NP40 buffer (25 mM Tris-HCl pH 8.0, 150 mM NaCl, 10% Glycerol). Subsequently, the cell lysate was either treated with concentration gradients of compounds of **B4** (1 µM, 5 µM, 10 µM) and incubated with the fixed concentration of VHL recombinant protein (1 µM) or treated with the fixed concentration of **B4** (5 µM) and incubated with concentration gradients of VHL recombinant protein (0.1 µM, 1 µM, 3 µM) for 10 min at room temperature. Then, the mixture was incubated with GST Beads (Smart-Lifesciences, SM002005) for immunoprecipitation overnight at 4 °C. The beads were then washed five times with wash buffer (25 mM Tris-HCl pH 8.0, 150 mM NaCl, 10% Glycerol). The proteins were eluted with 2× loading buffer (20 mM Tris-base, 4% (w/v) SDS, 16% (w/v) glycerol, 3% (w/v) DTT, and 0.02% (w/v) bromophenol blue, pH 6.8), followed by immunoblotting with anti-HDAC7, anti-HDAC9 and anti-VHL antibodies. Results are presented in Figure S9.

**(16)** ***in vivo* Studies**

We confirm that the *in vivo* experiment in this study complies with all relevant ethical regulations. Maintenance and experimental procedures for the mice studies were approved by The Innovation Institute for Artificial Intelligence in Medicine, Zhejiang University’s IACUC (DW202210131929). Preliminary experiments designed to ascertain non-toxicity levels suggested an administration of 12.5 mg/kg of **B4** did not result in any animal death, suggesting its suitability as a safe dosage for the *in vivo* model (Table S8).

**Table S8.** **Preliminary studies to define the compounds’ effective dosage range**


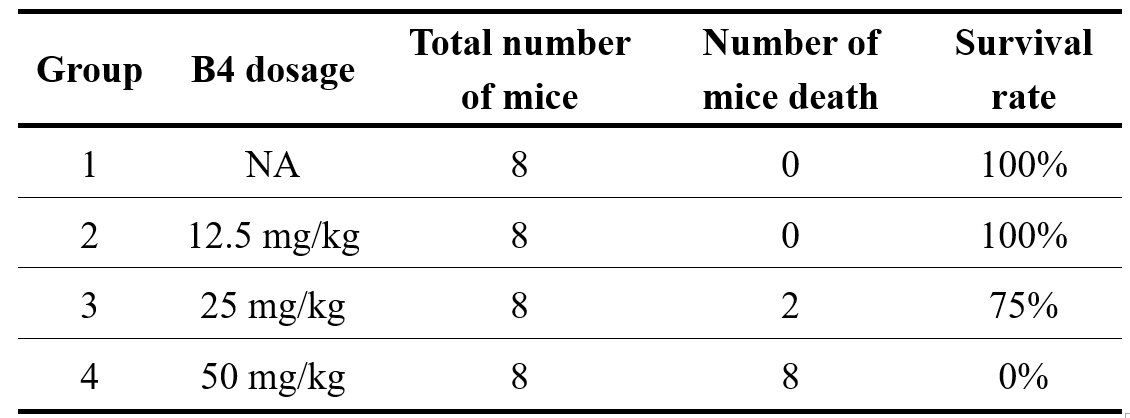


In the mice study, ICR mice (8 weeks, Charles River Animal Resource, Beijing, China) were kept in ventilated cages and provided with sterilized food, water, and bedding. The mice were maintained within a temperature range of 21 to 26 °C and a humidity range of 50% to 70%. The mice were randomly assigned and separately administered with **B4** (i.v, 12.5 mg/kg), **B6** (i.v, 12.5 mg/kg), **TMP269** (i.v, 12.5 mg/kg), Hexadecadrol (i.g, 2 mg/kg) for 6 h, then the mice were intraperitoneal injection with 10 mg/kg LPS to induce acute inflammation. Two hours later, the serum samples were collected and allowed to stand at room temperature for 4 h until separation occurred. Subsequently, the samples were centrifuged at 4,000 rpm for 30 min, and the supernatant (100-300 μL) was collected. Then detect the levels of proinflammatory cytokines using commercially accessible ELISA kits (BIOKER). Results are presented in Figure 8 and Figure S8.

**(17)** **Statistical analysis**

All statistical evaluation was performed with Graphpad Prism 8.4.3 (Graphpad Software), data were analyzed by one-way analysis of univariate variance (ANOVA) or unpaired Student's T‑test. For all numerical tests, a probably value (**, *P*＜0.01, ***, *P*＜0.001, ****, *P*＜0.0001, ns: not significant) was considered to be statistical significant. All data are shown as the mean ± standard deviation (SD) from at least three biological independent experiments.

**D. Supplementary Figures of Biological Evaluation**

**Figure S2**: **TMP269 inhibits enzyme activity of HDAC7 in RAW264.7.** RAW264.7 cells were pre-treated with 5 μM **TMP269** for 12 h. After the treatment, cells were collected and lysed by RIPA. The kinase assay was performed using **TMP269** treated cell lysis and **Ac-Leu-Gly-Lys(TFAc)-AMC** substrate. The assay was conducted at 30 ℃. The fluorescence of 7-amino-4-methylcoumarin released by 0.5 μg/mL Trypsin represented the kinase activity of HDAC7. Additionally, 5 μM **TMP269** was treated in cell lysis to ensure accurate measurement of HDAC7’s *in vitro* kinase activity inhibitory assay.


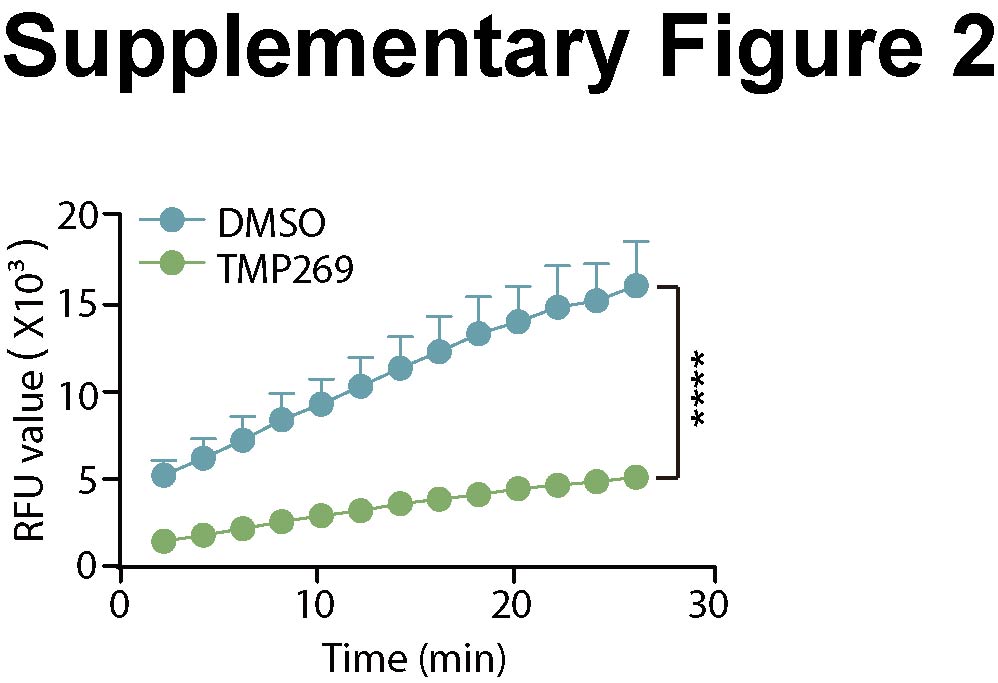


**Figure S3**: **Evaluation of the *in vitro* inhibitory activity of all VHL-based PROTACs that varied in linker length against class IIa HDACs** (the parental inhibitor **TMP269** was utilized as a positive control).


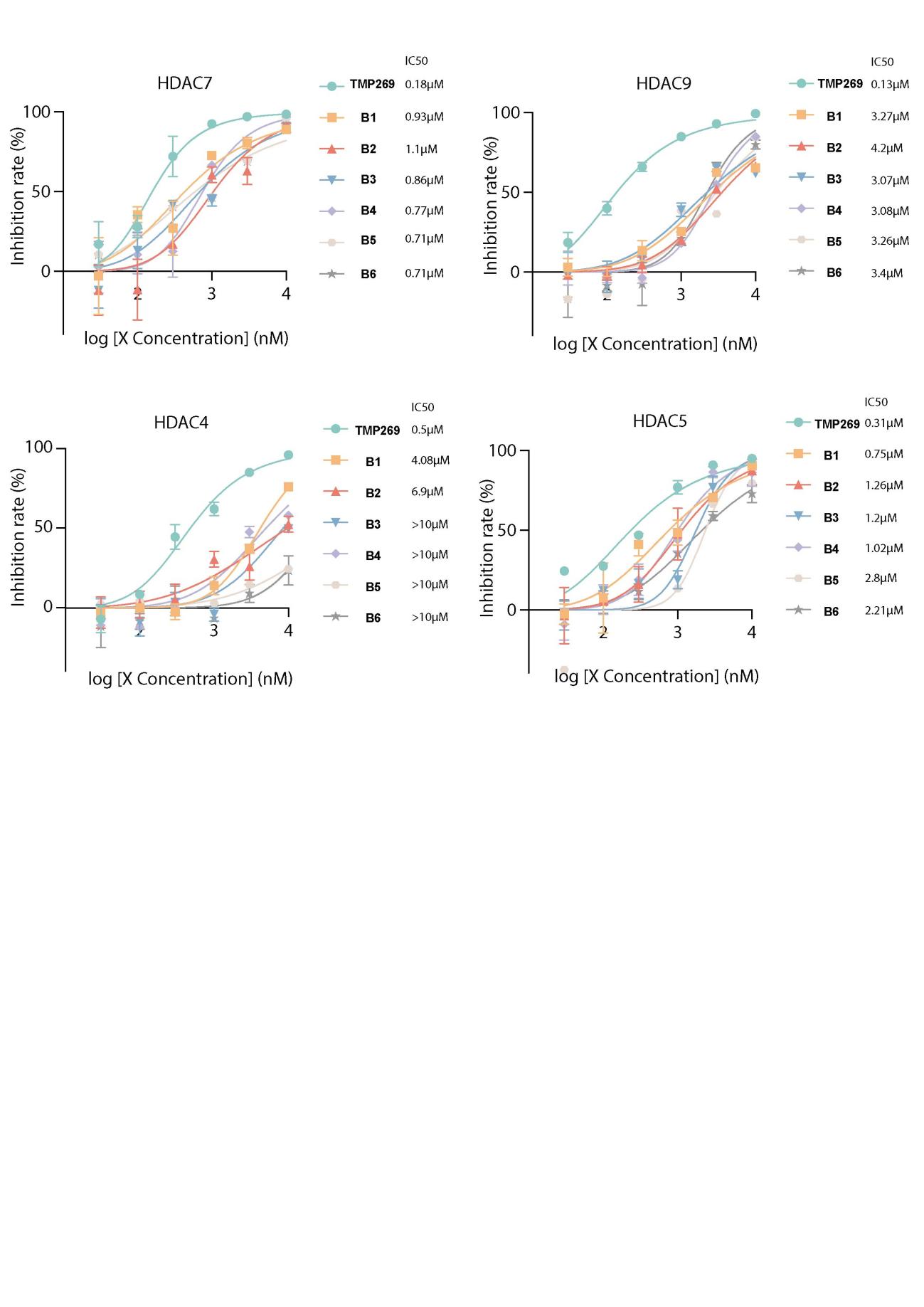


**Figure S4: Evaluation of the dose-dependent cytotoxicity of B4 and B5 in RAW264.7 cells**


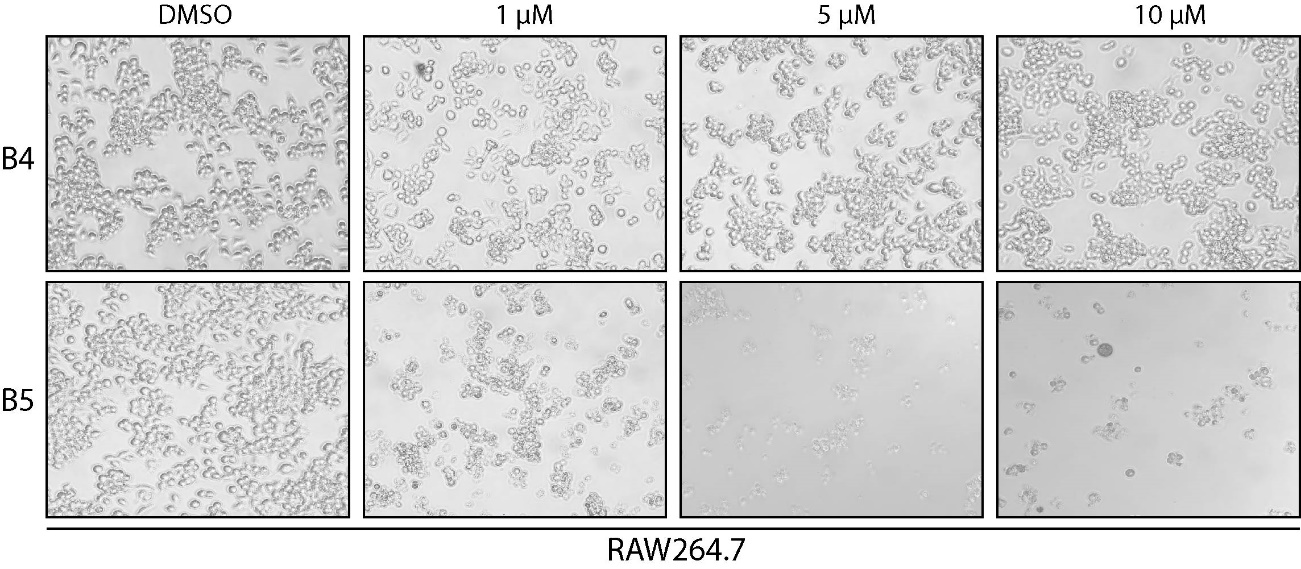


**Figure S5**: **B4 induces isoform-selective degradation of HDAC7.** **(A)** The proteomic analysis of protein expression changes in 293T cells after treated with **B4** (5 μM), measured on a log_2_FC scale. Note: HDAC7 was not detected in this experiment probably due to its low abundance. **(B)** The mRNA expression analysis of *HDAC7* using qRT-PCR. Graphical data were derived from three independent experiments. Statistical significance levels indicated as follow: not significant (n.s.).


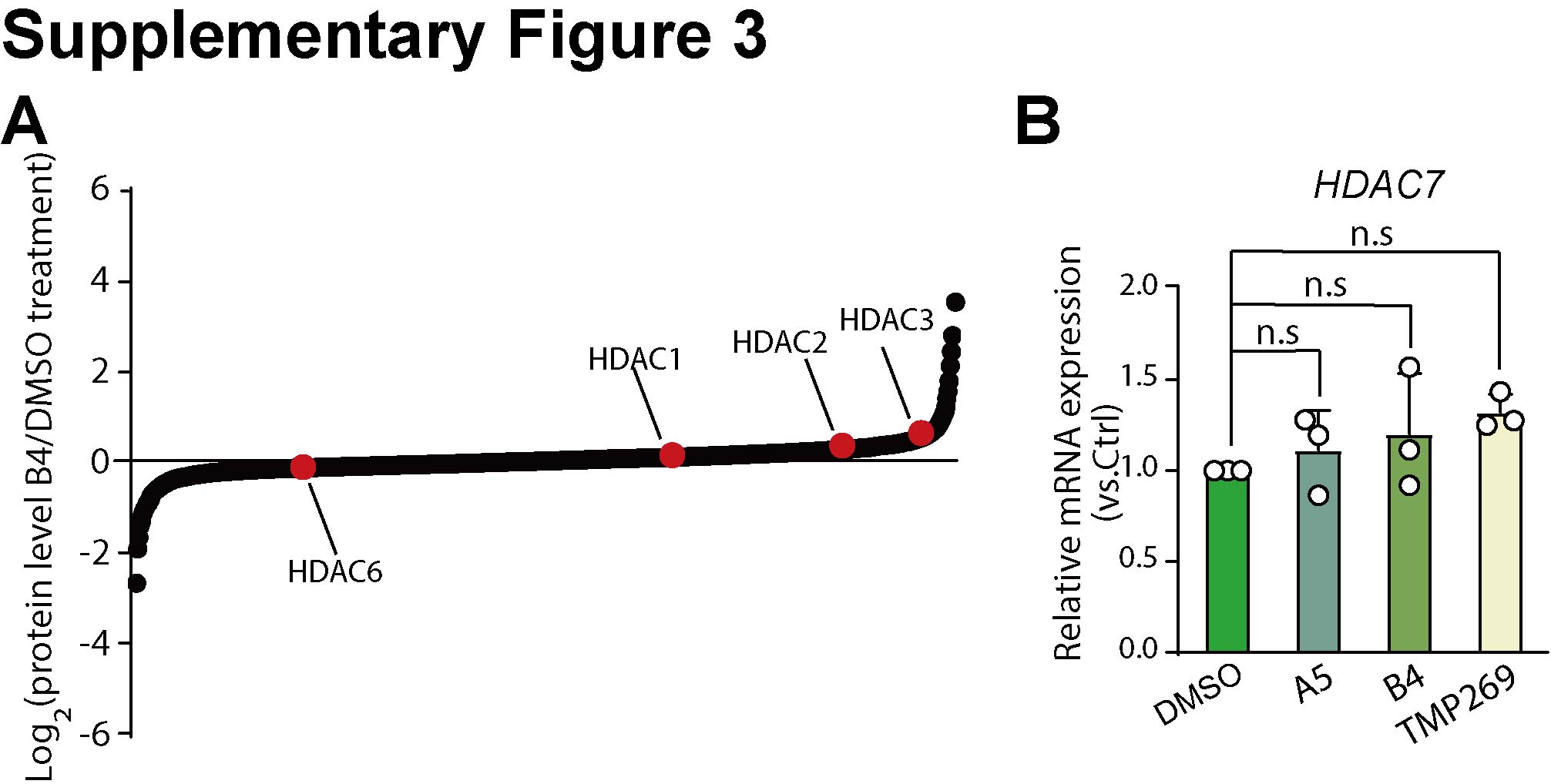


**Figure S6**: **Left: analysis of HDAC7 protein levels in *CRBN*-KO cells and *CRBN* re-expressed 293T cells treated with A5 (5 μM) for 12 hours; Right: analysis of HDAC7 protein levels in *VHL*-KO cells and *VHL* re-expressed 293T cells (left) treated with B4 (5 μM) for 12 hours.**


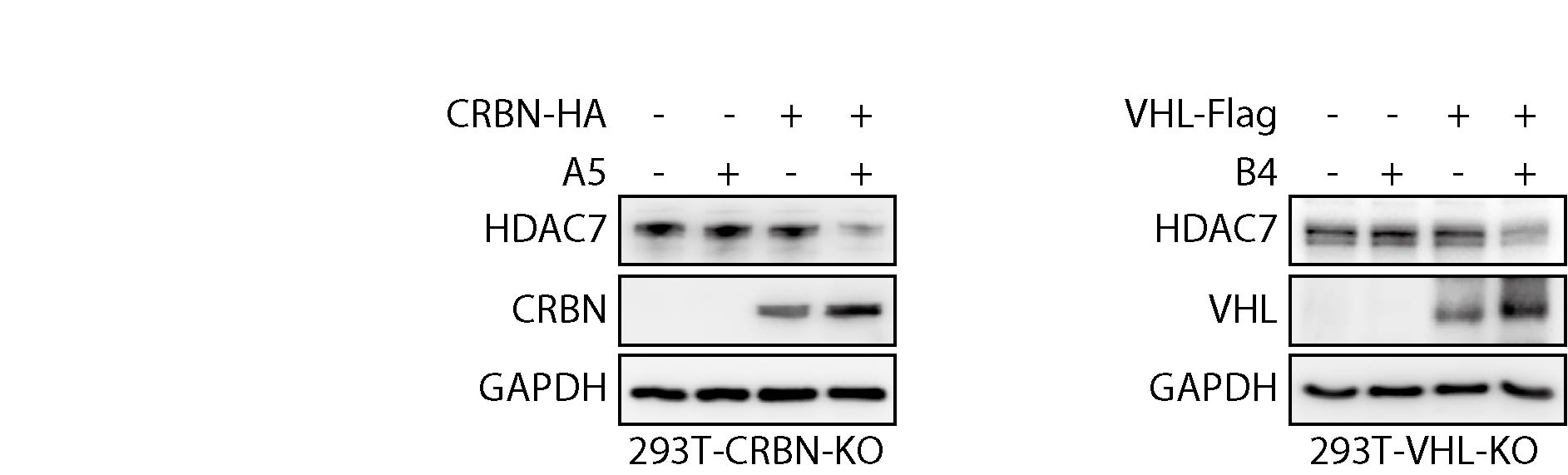


**Figure S7**: **A5-mediated HDAC7 degradation reduces inflammatory cytokine levels in both RAW264.7 and BMDM cell lines.** **(A)** Immunoblotting analysis of HDAC7 protein levels after treatment with various concentrations of **A5** for 12 h (left) and after treatment with **A5** (5 μM) at different time points (right) in RAW264.7 cells. **(B)** Immunoblotting analysis of HDAC7 protein levels after treatment with **A5** (5 μM) and **TMP269** (5 μM) in RAW264.7 and BMDM cells. **(C)** Protein levels of HDAC4, HDAC7, HDAC8, and HDAC9 in RAW264.7 cells analyzed by immunoblotting after treatment with **A5** (5 μM) and **TMP269** (5 μM). **(D-G)** ELISA analysis of the secretion levels of IL-6 and TNF-α in both RAW264.7 and BMDM cells. Cells were incubated with **A5** (5 μM) and **TMP269** (5 μM) for 12 h, followed by treatment with 10 ng/mL LPS for 24 h. All data represent a minimum of three replicates. Statistical significance levels indicated as follows: not significant (n.s.), **, *P* < 0.01 and ***, *P* < 0.001.

**
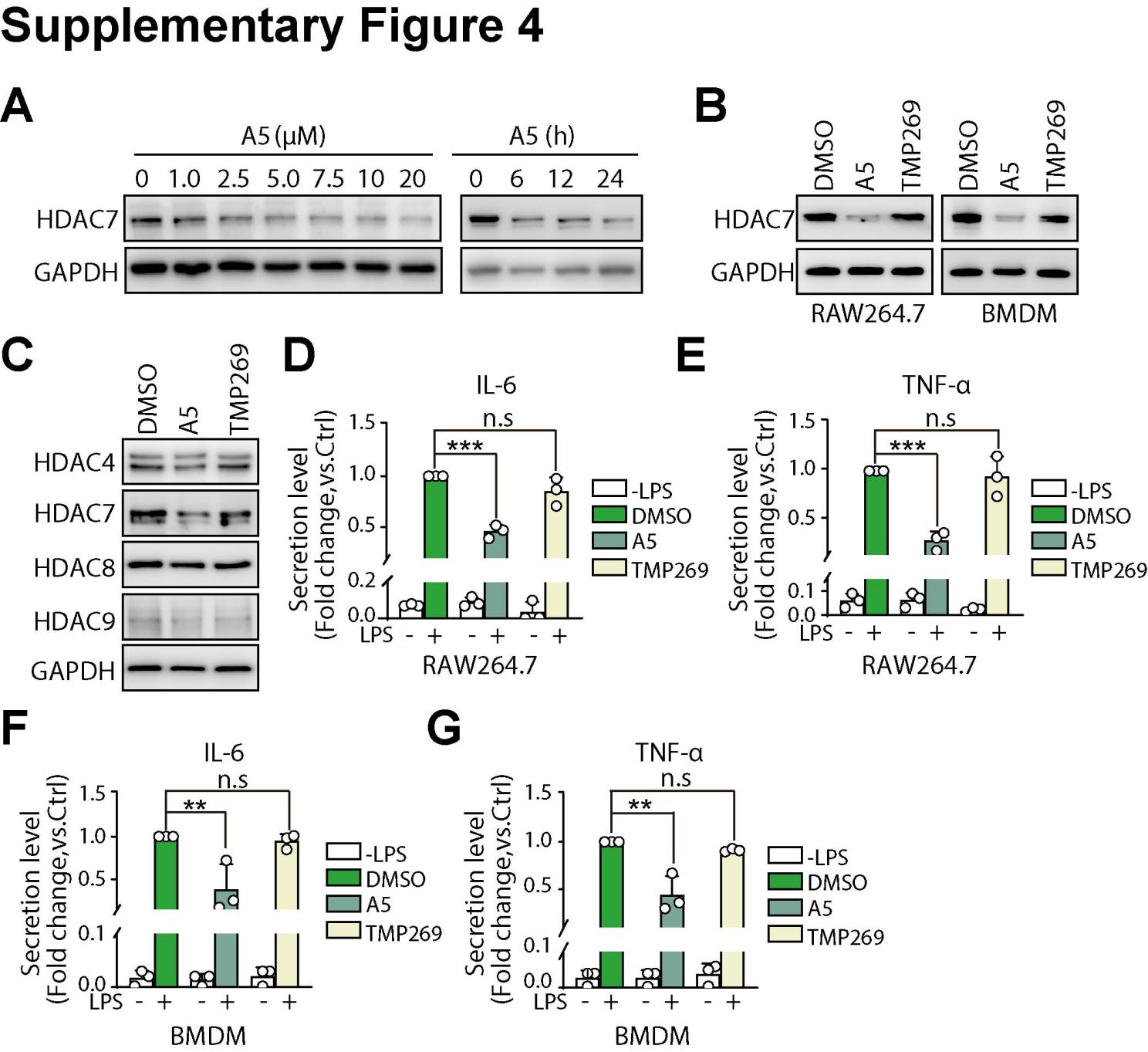
**

**Figure S8**: Evaluation of the *in vivo* biological effects of various compounds (**TMP269**, **B4**, **B6** and Hexadecadrol) on inflammatory cytokine levels. Eight-week-old ICR mice in each group (n=5) received pre-treatment with **TMP269** (i.v., 12.5 mg/kg), **B4** (i.v., 12.5 mg/kg), **B6** (i.v., 12.5 mg/kg) or Hexadecadrol (i.g., 2.0 mg/kg) for 6 hours before LPS administration (i.p., 10 mg/kg). Serum samples were collected from each group 2 hours post-LPS exposure. A-D) Measurement of IL-6, TNF-α, IL-1β and GM-CSF secretion levels was performed using the ELISA assay.

**
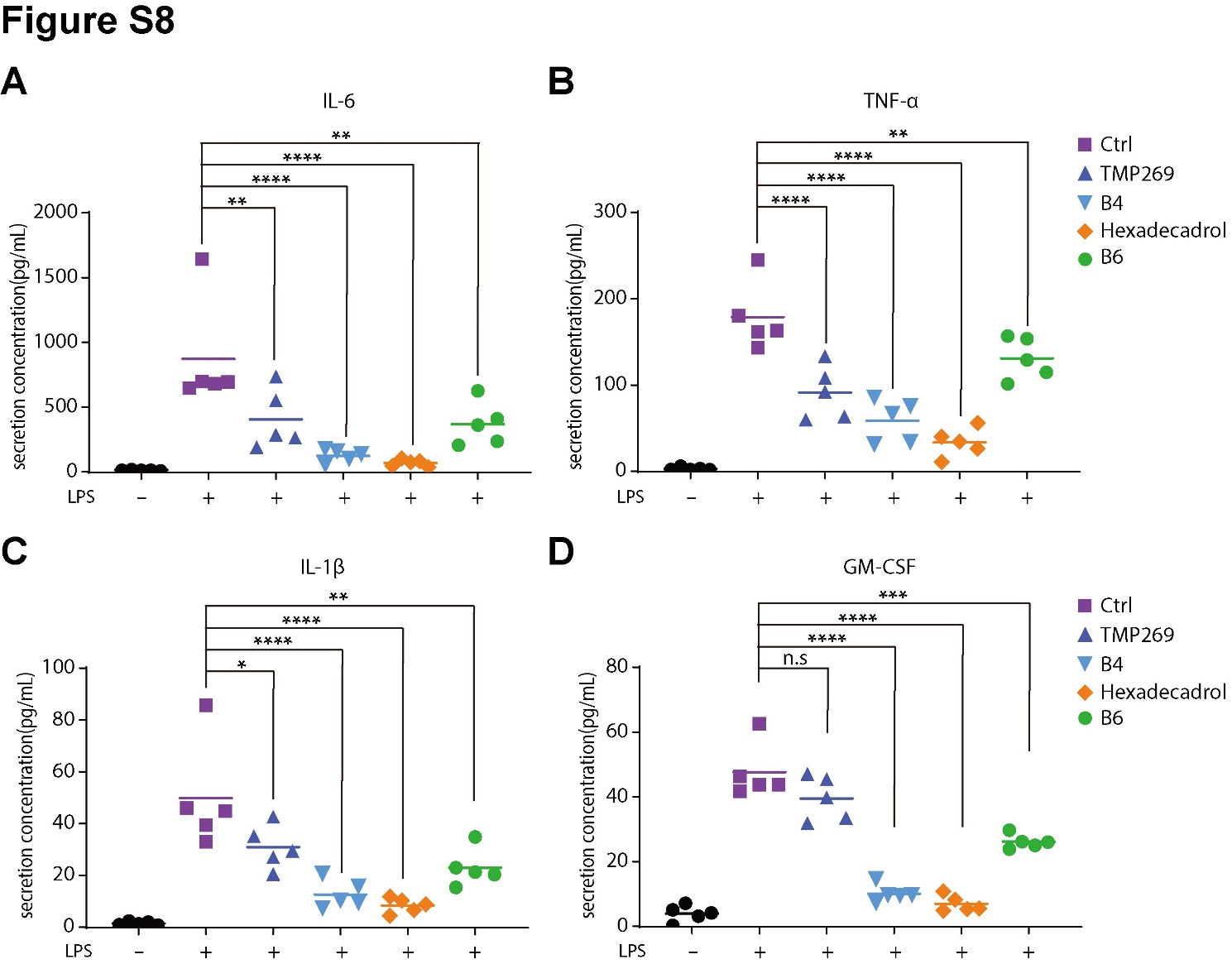
**

**Figure S9**: **Pull down assay of HDAC7/9-B4-VHL ternary complex.** 293T cells were co-transfected with the indicated plasmids (HDAC7-Flag or HDAC9-Flag) for 24 h and lysed using 1% NP40 buffer. (A-B) The HDAC7-**B4**-VHL ternary complex was detected by GST pull down assay. The cell lysate was treated with concentration gradients of compounds of **B4** (1 µM, 5 µM, 10 µM) and incubated with the fixed concentration of VHL recombinant protein (1 µM, **Figure S9A**) or treated with the fixed concentration of **B4** (5 µM) and incubated with concentration gradients of VHL recombinant protein (0.1 µM, 1 µM, 3 µM, **Figure S9B**). Then, the mixture was incubated with GST beads for immunoprecipitation, followed by immunoblotting with anti-HDAC7, and anti-VHL antibodies. (C-D) The HDAC9-**B4**-VHL ternary complex was detected by GST pull down assay. The cell lysate was treated with concentration gradients of compounds of **B4** (1 µM, 5 µM, 10 µM) and incubated with the fixed concentration of VHL recombinant protein (1 µM, **Figure S9C**) or treated with the fixed concentration of **B4** (5 µM) and incubated with concentration gradients of VHL recombinant protein (0.1 µM, 1 µM, 3 µM, **Figure S9D**). Then, the mixture was incubated with GST beads for immunoprecipitation, followed by immunoblotting with anti-HDAC9, and anti-VHL antibodies.


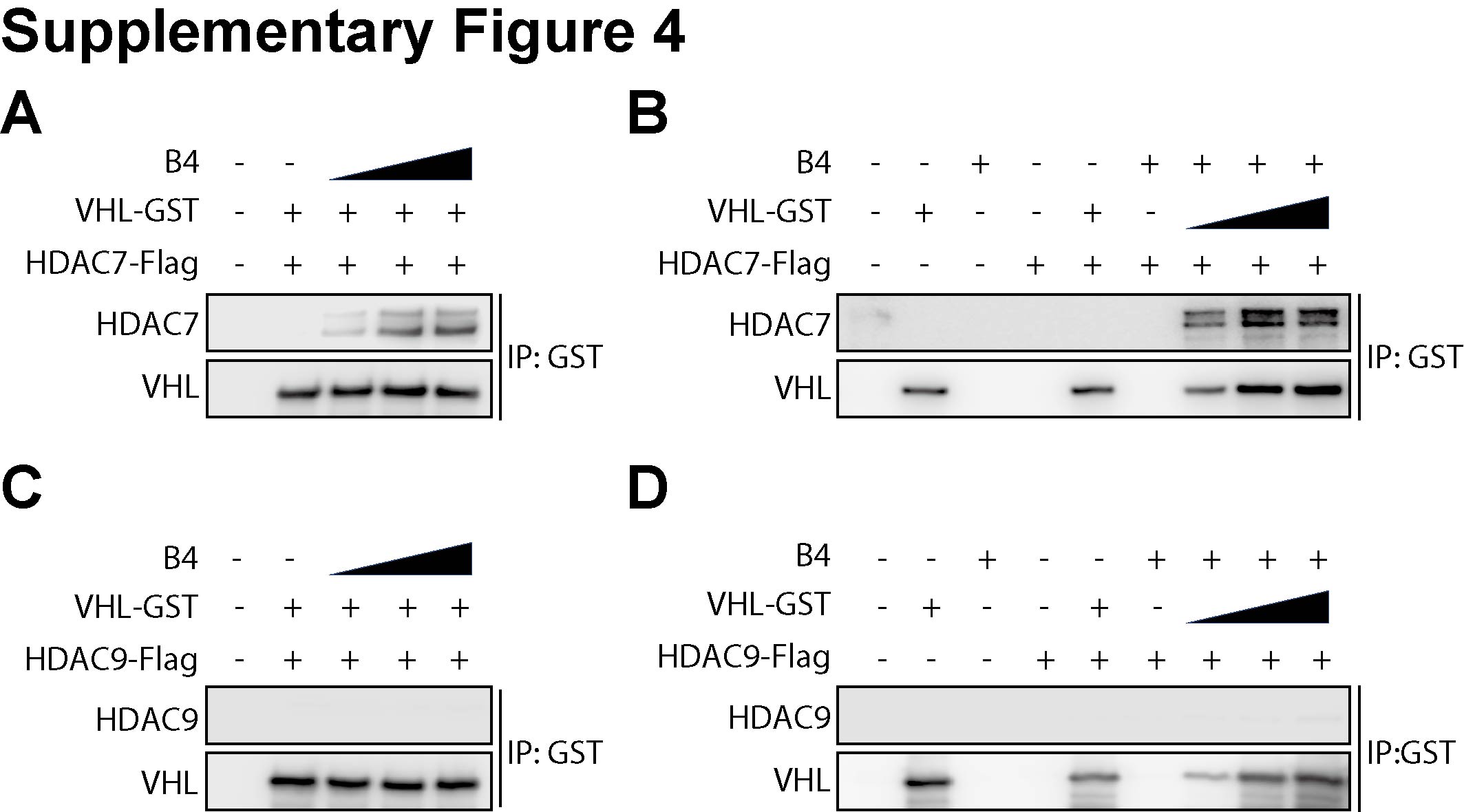


**E. NMR Spectra**

**F. HPLC and HRMS Analysis Reports**


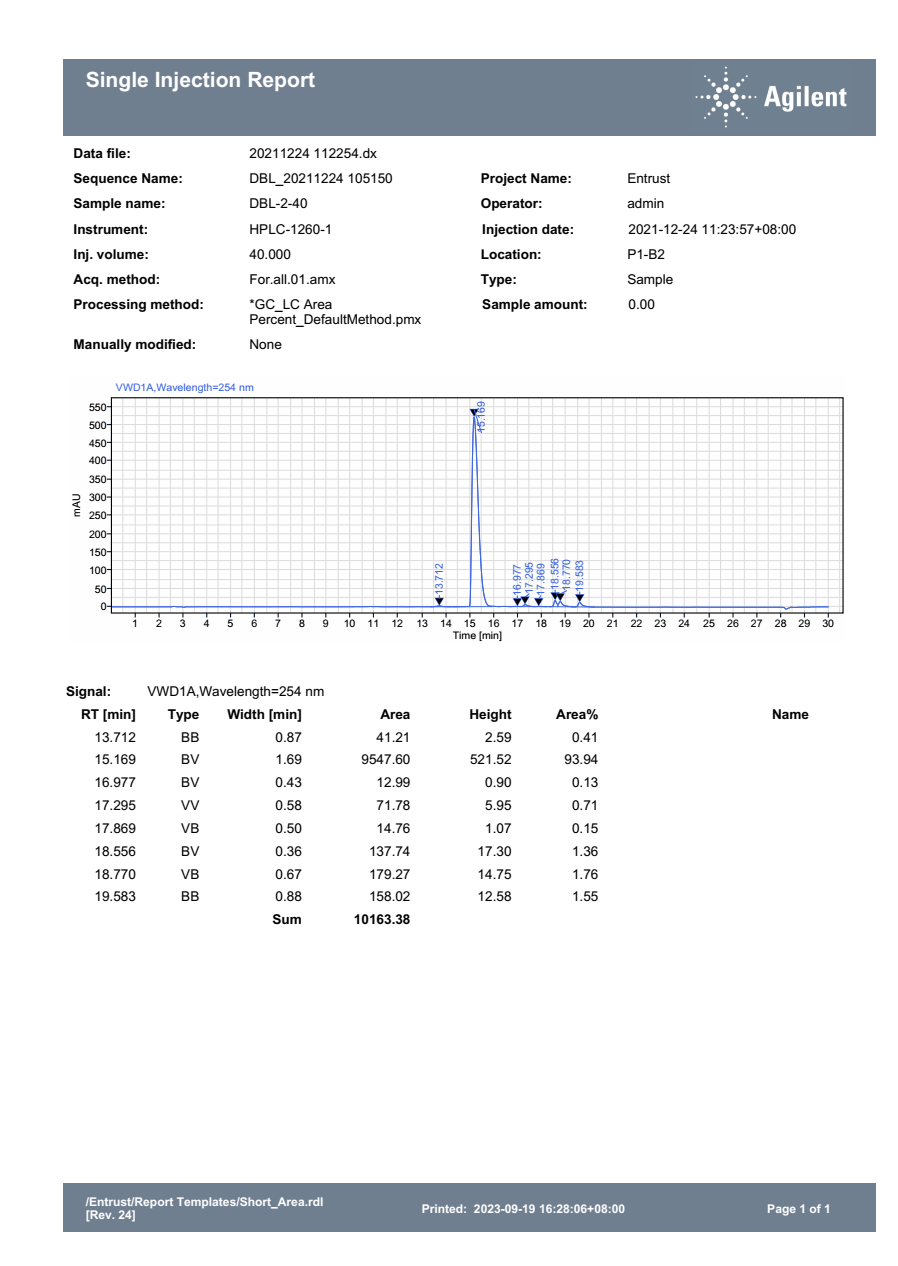


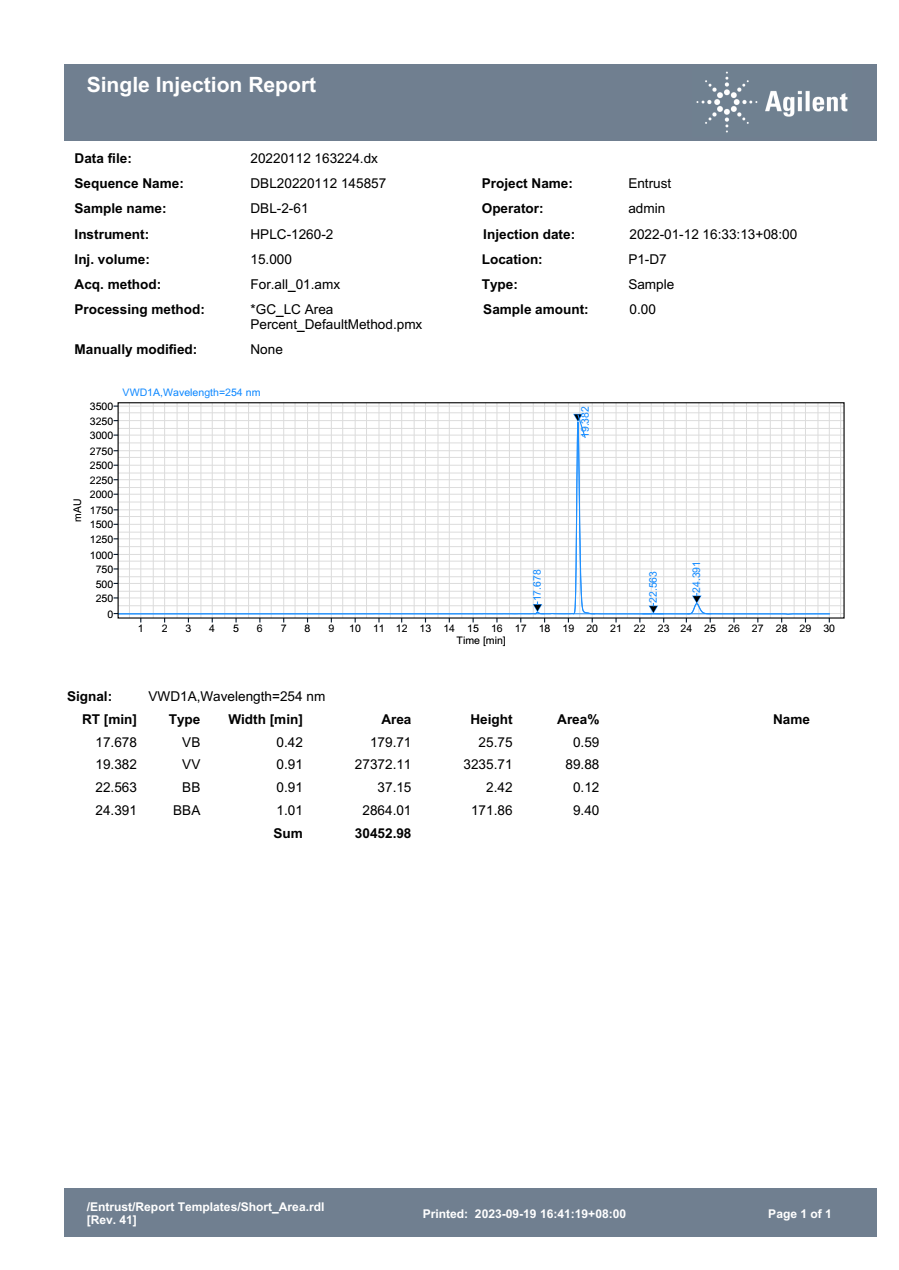


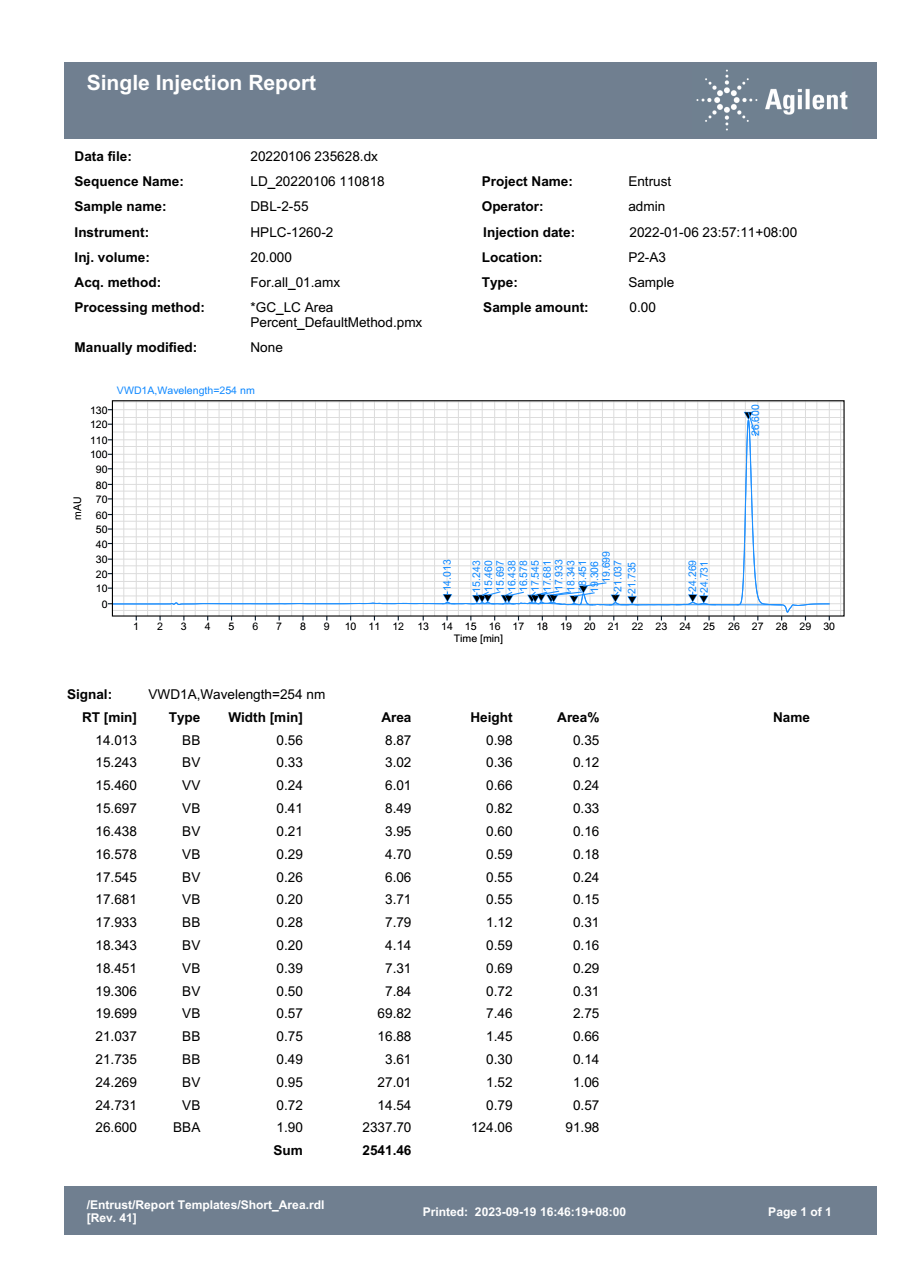


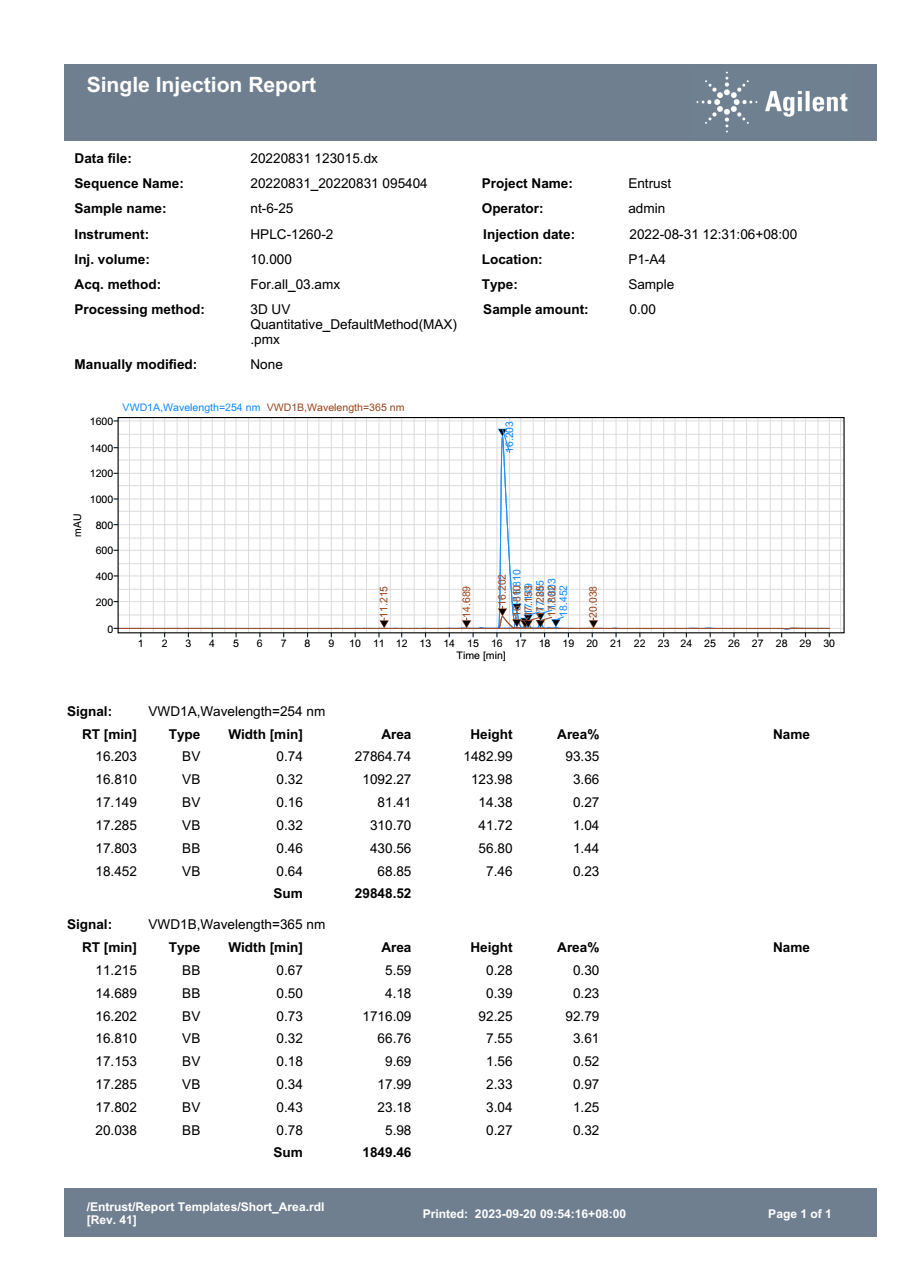


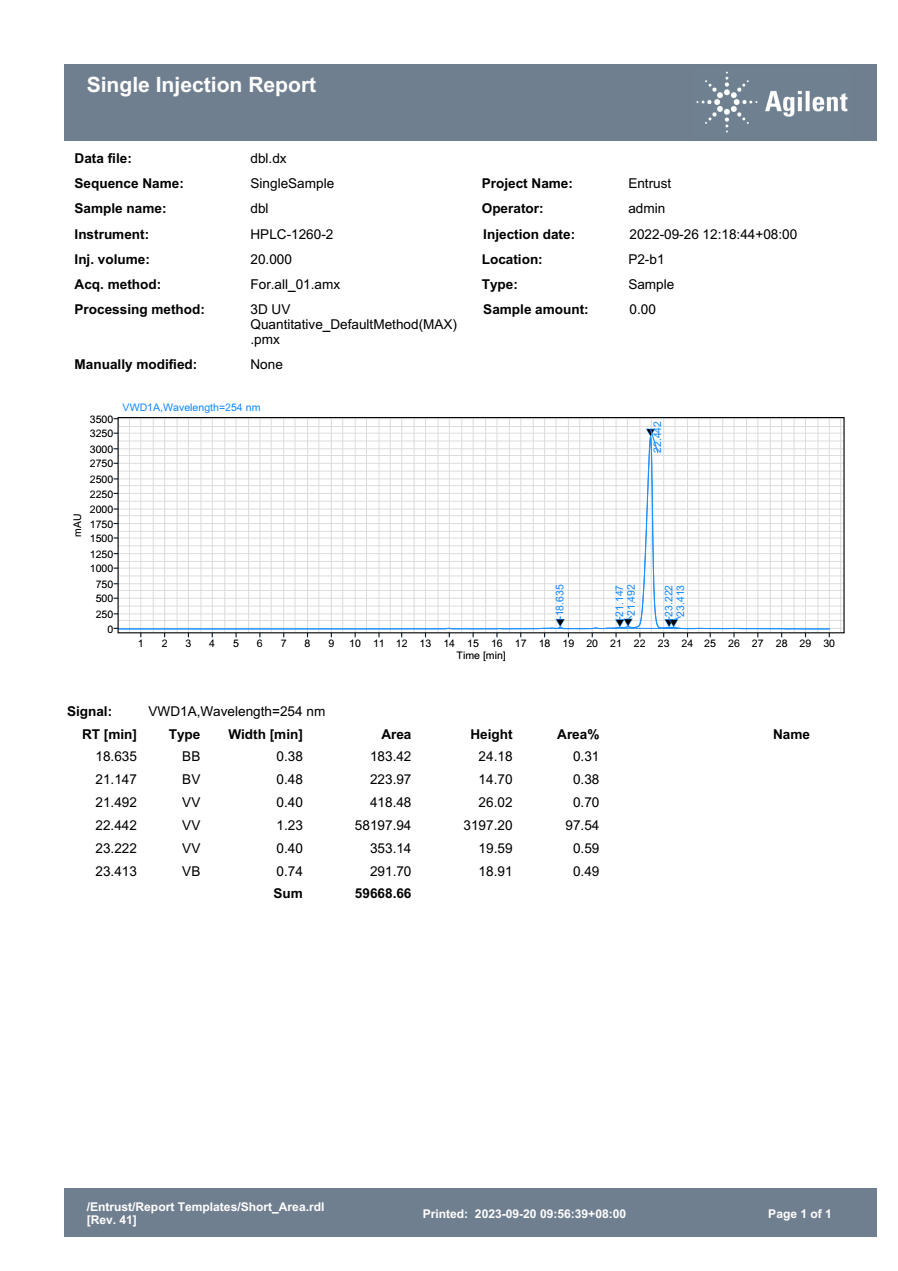


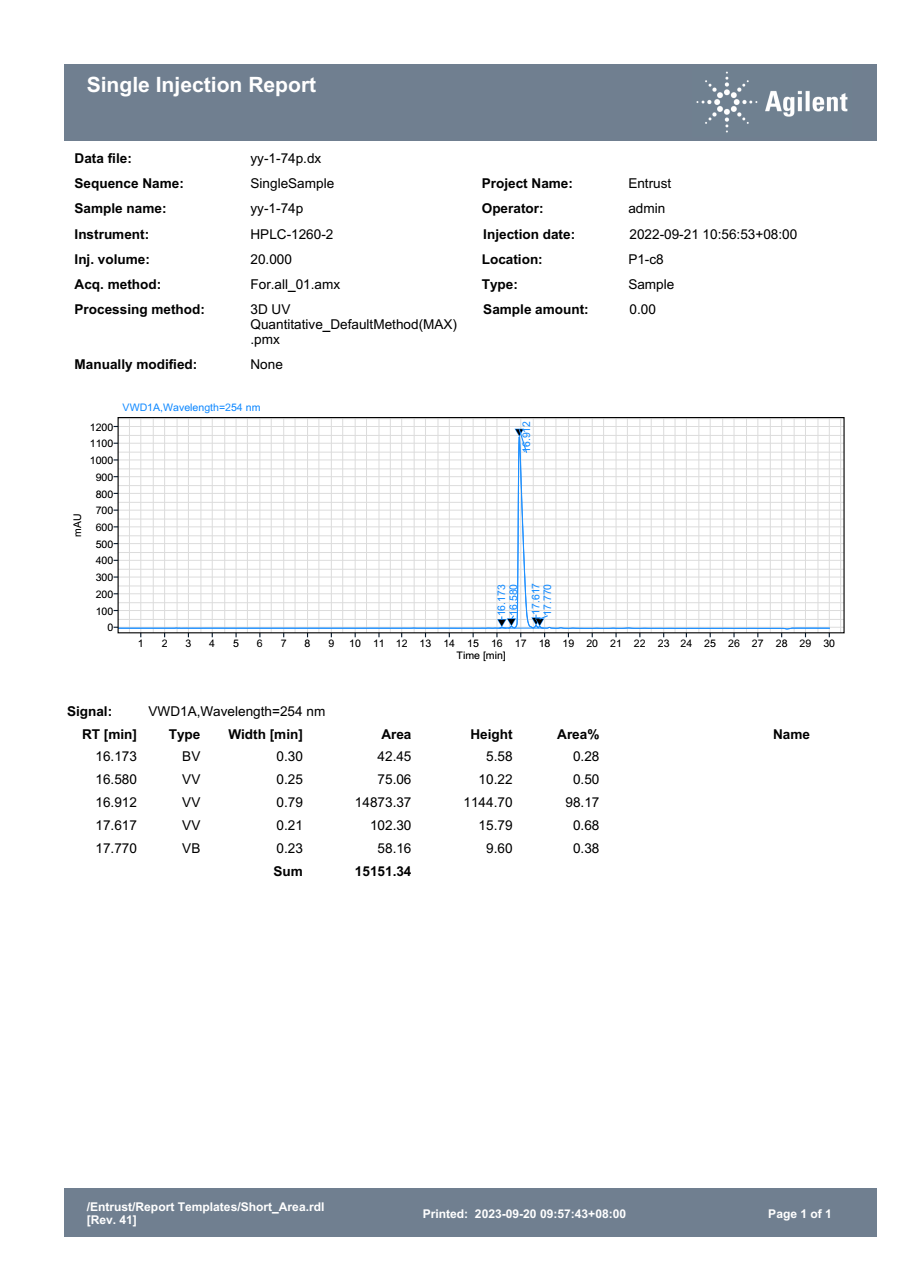


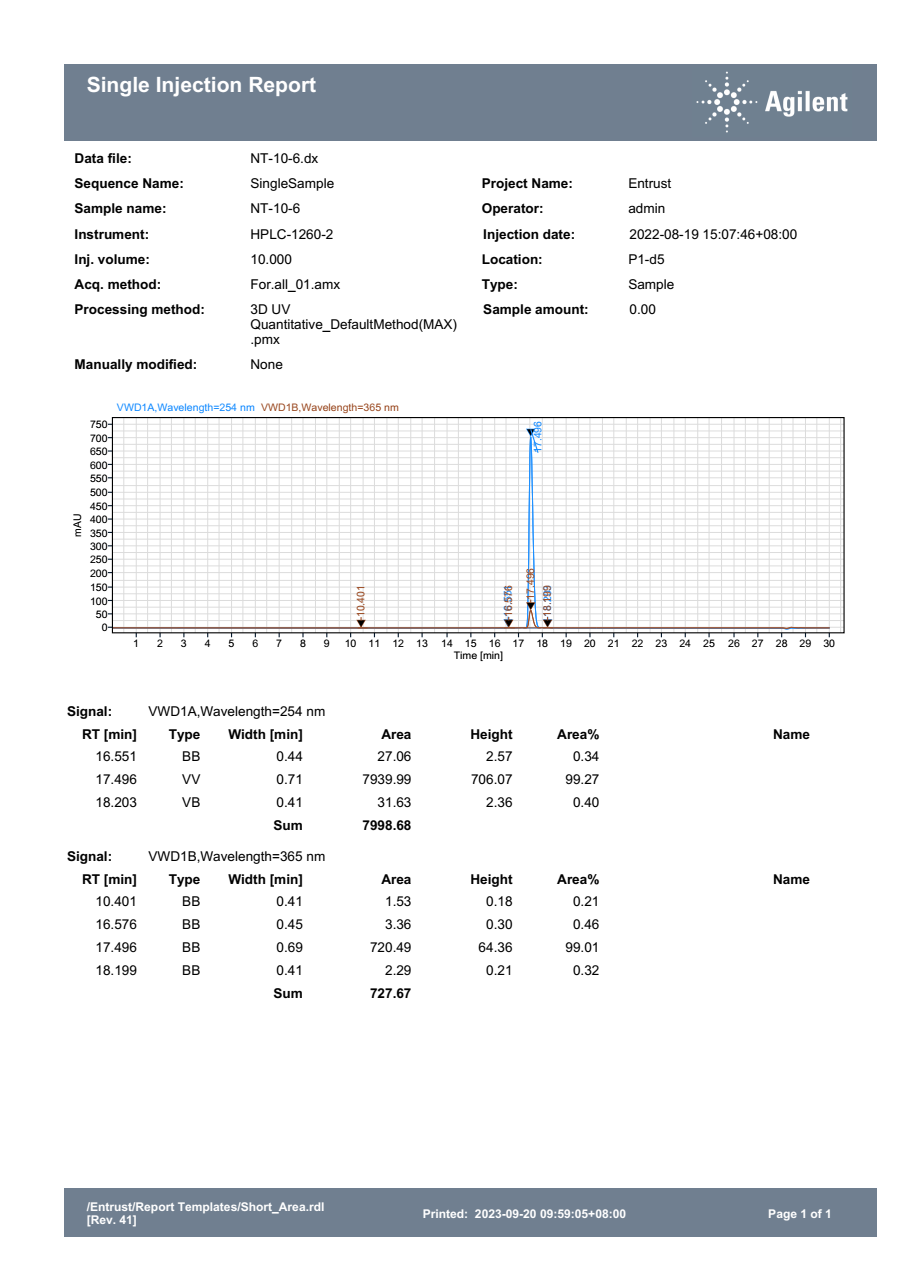


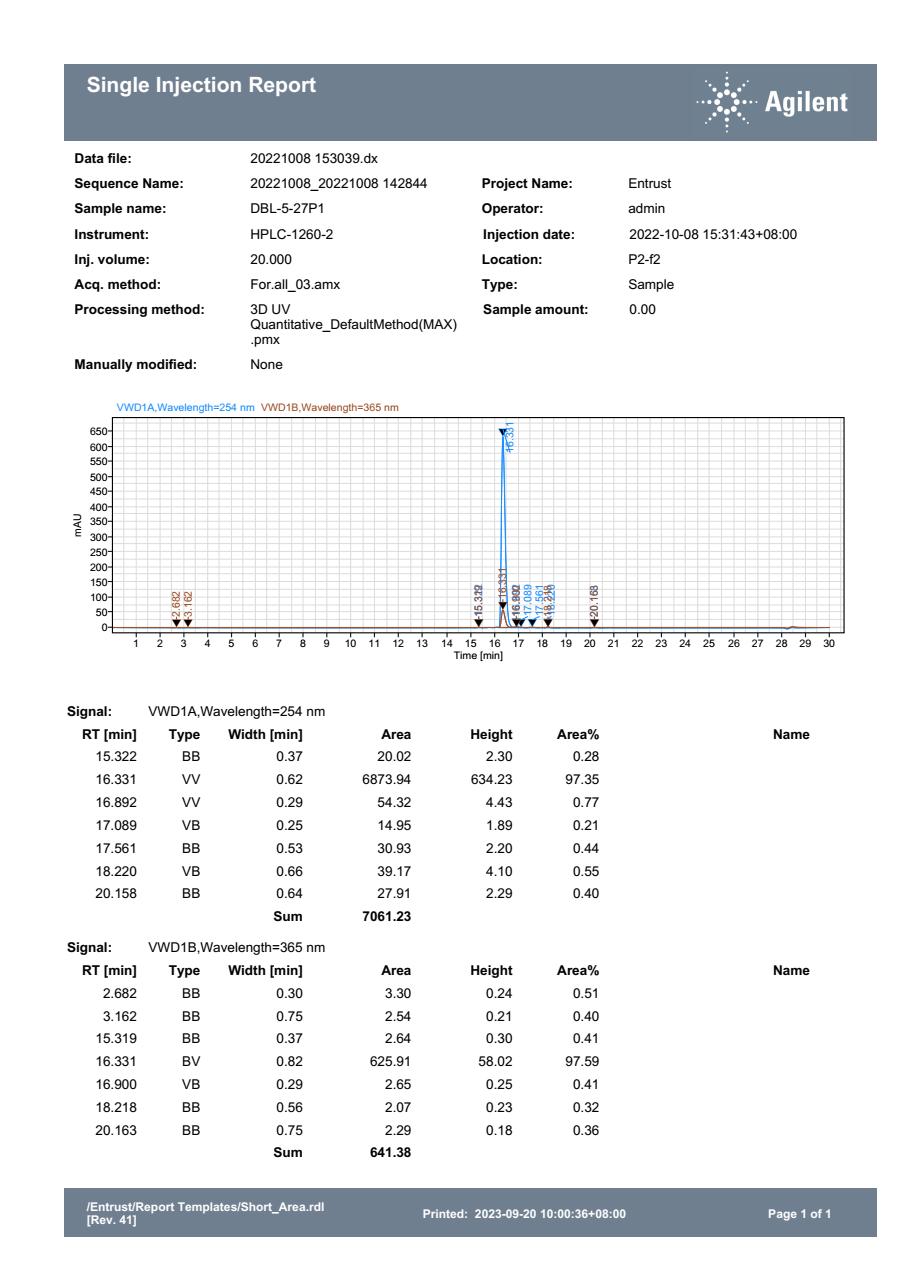


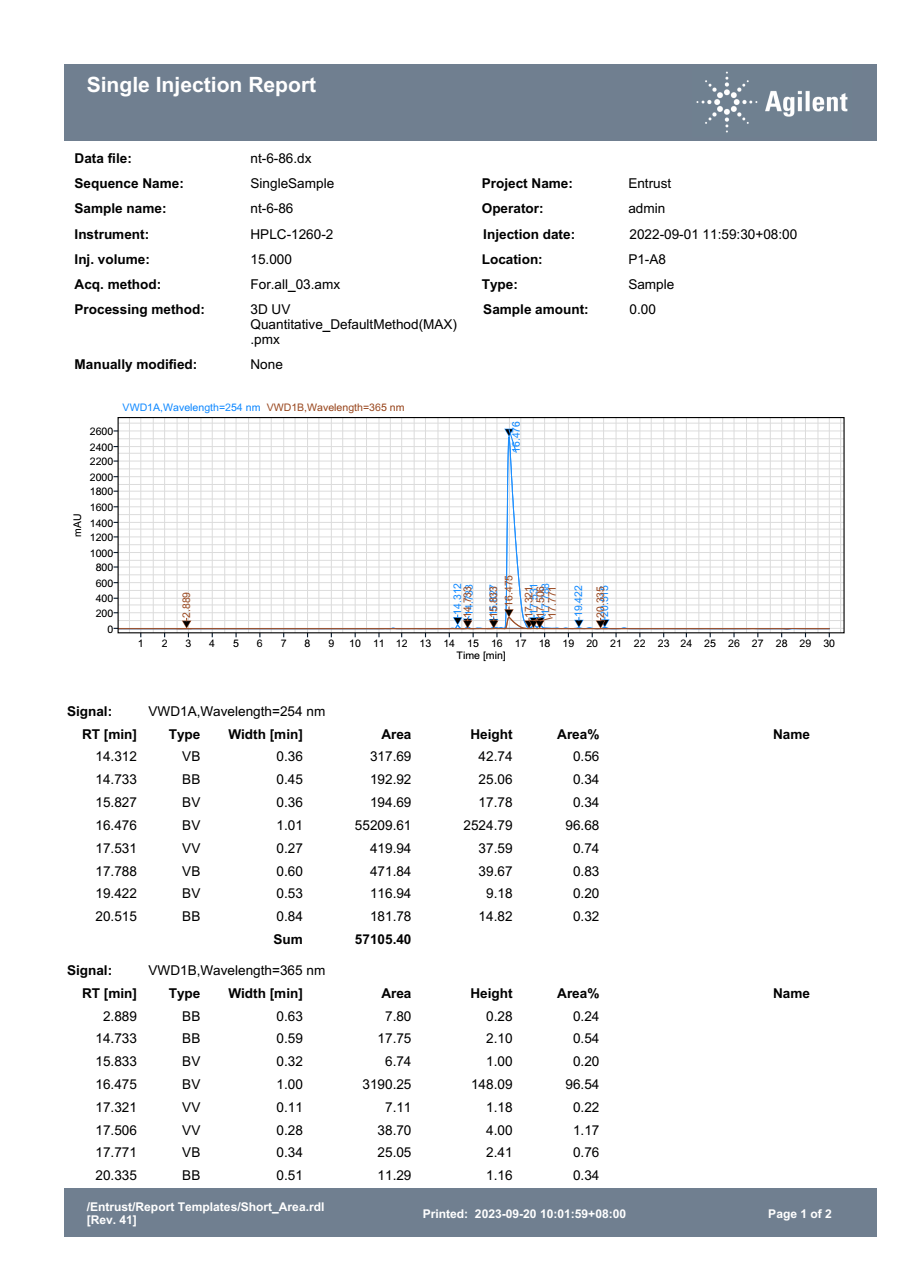


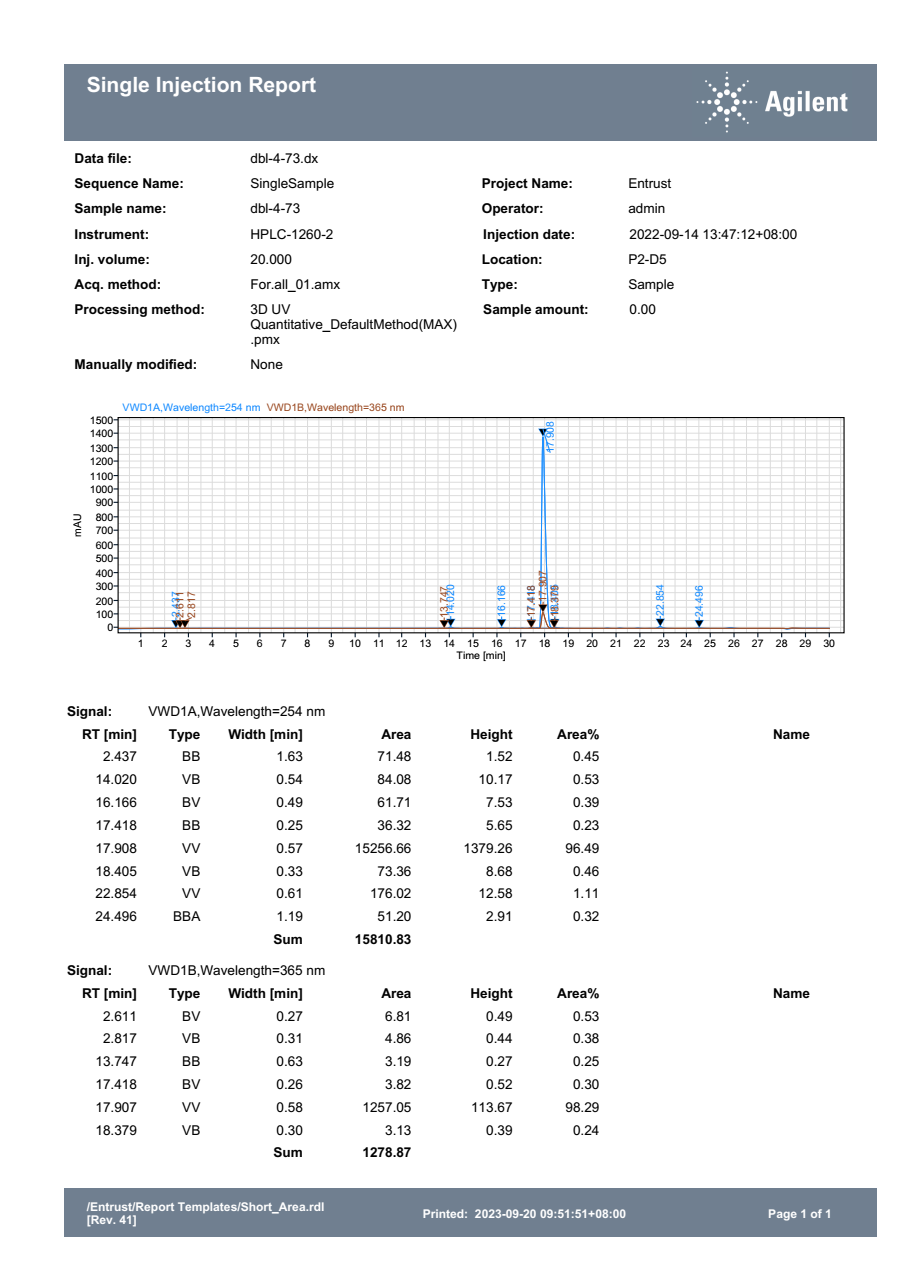


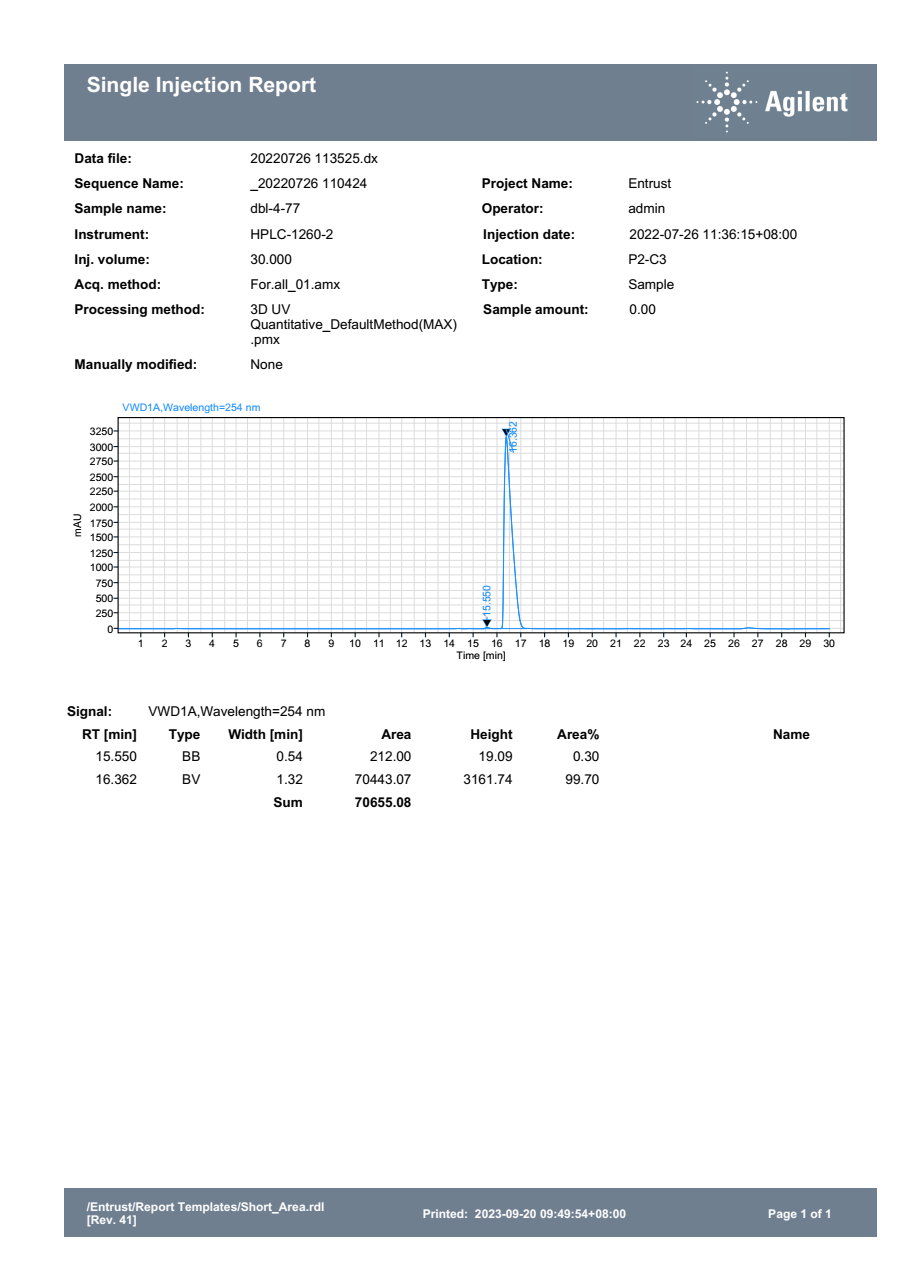


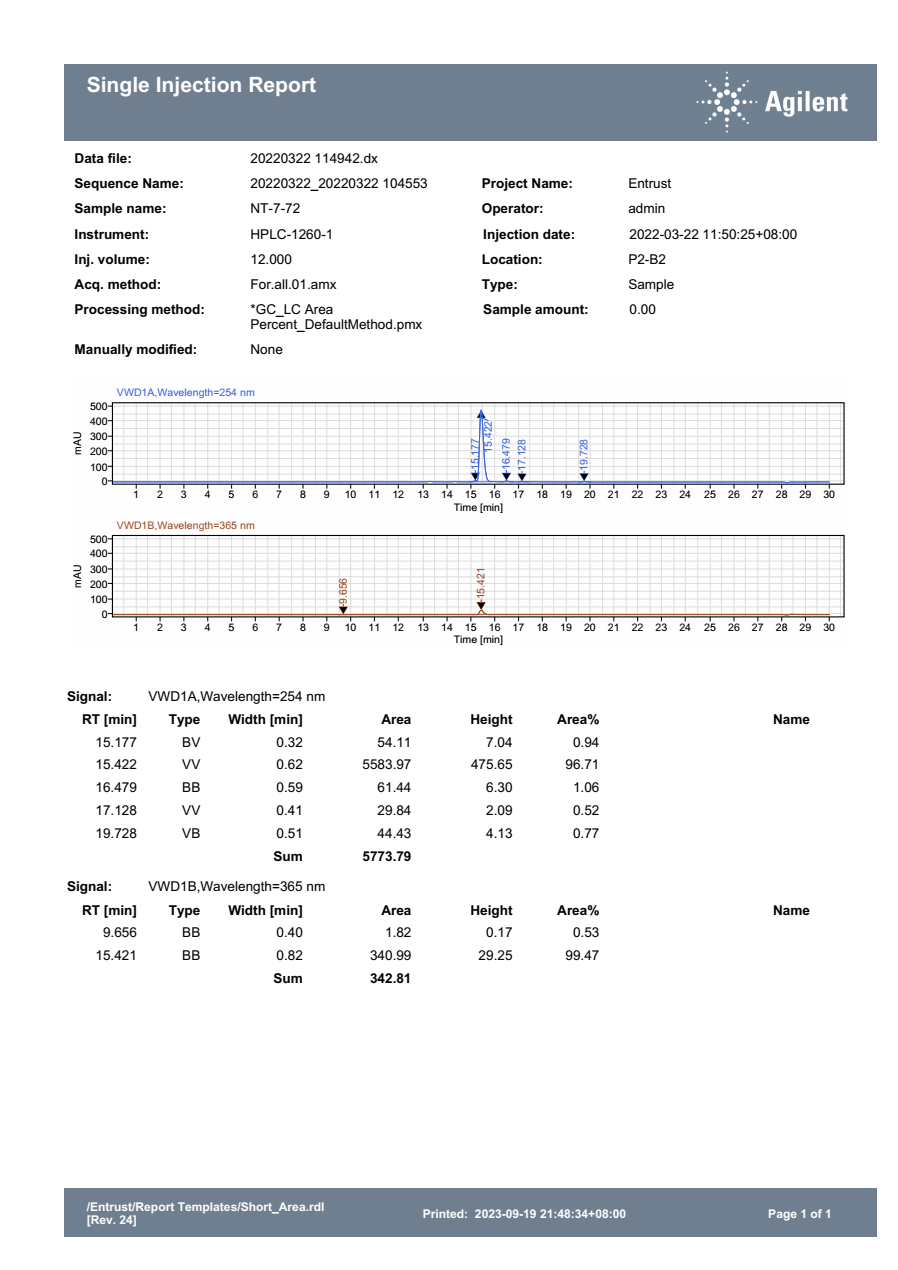


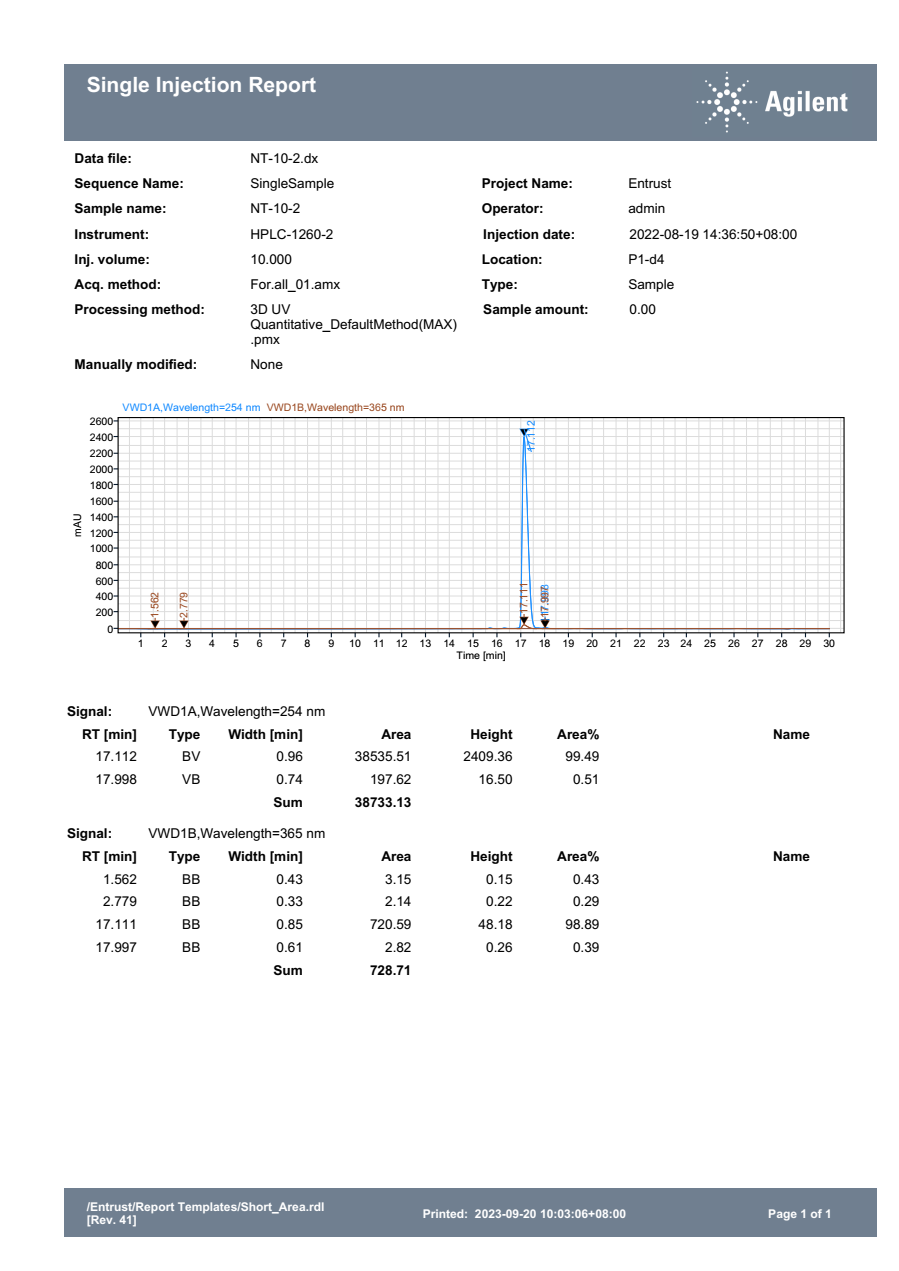


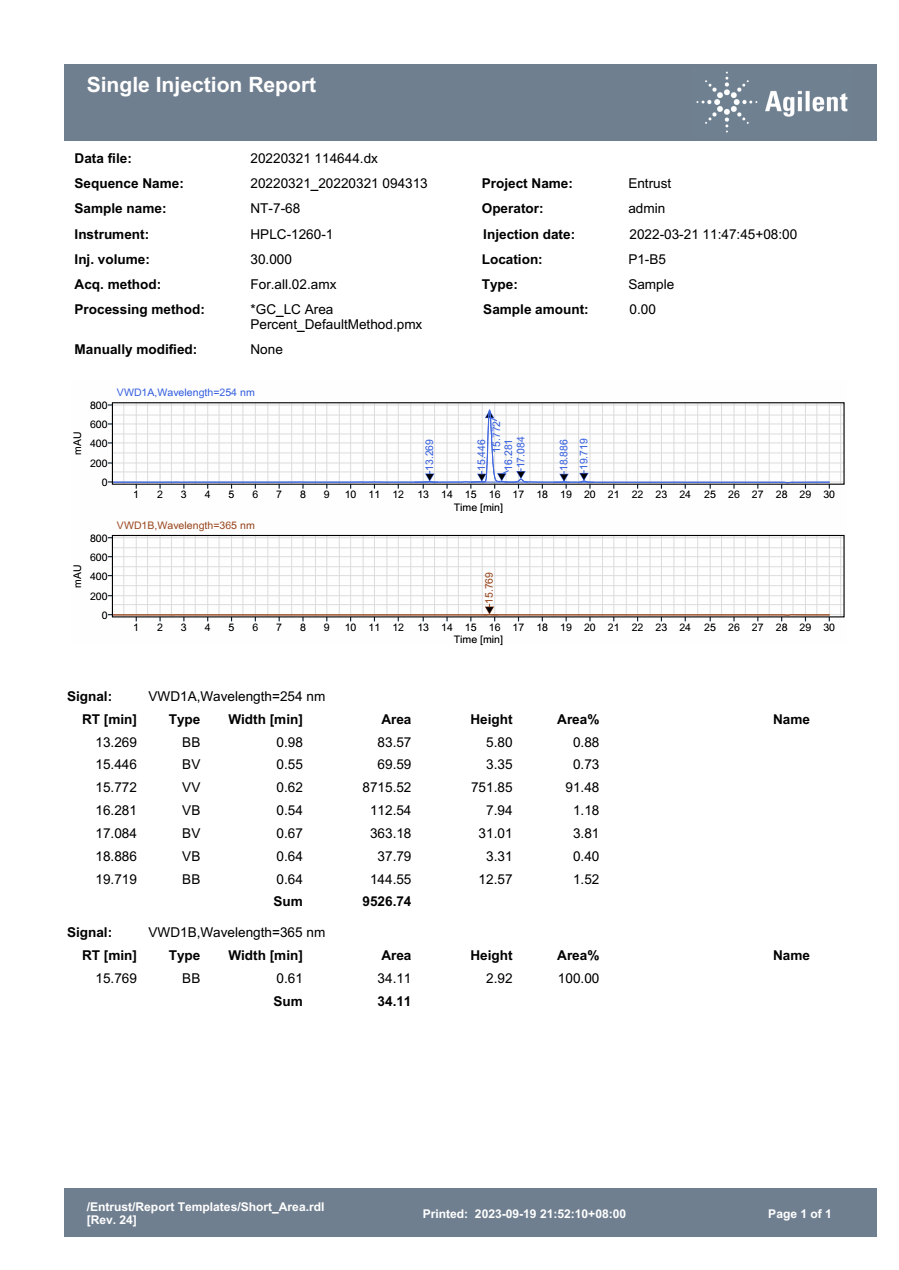


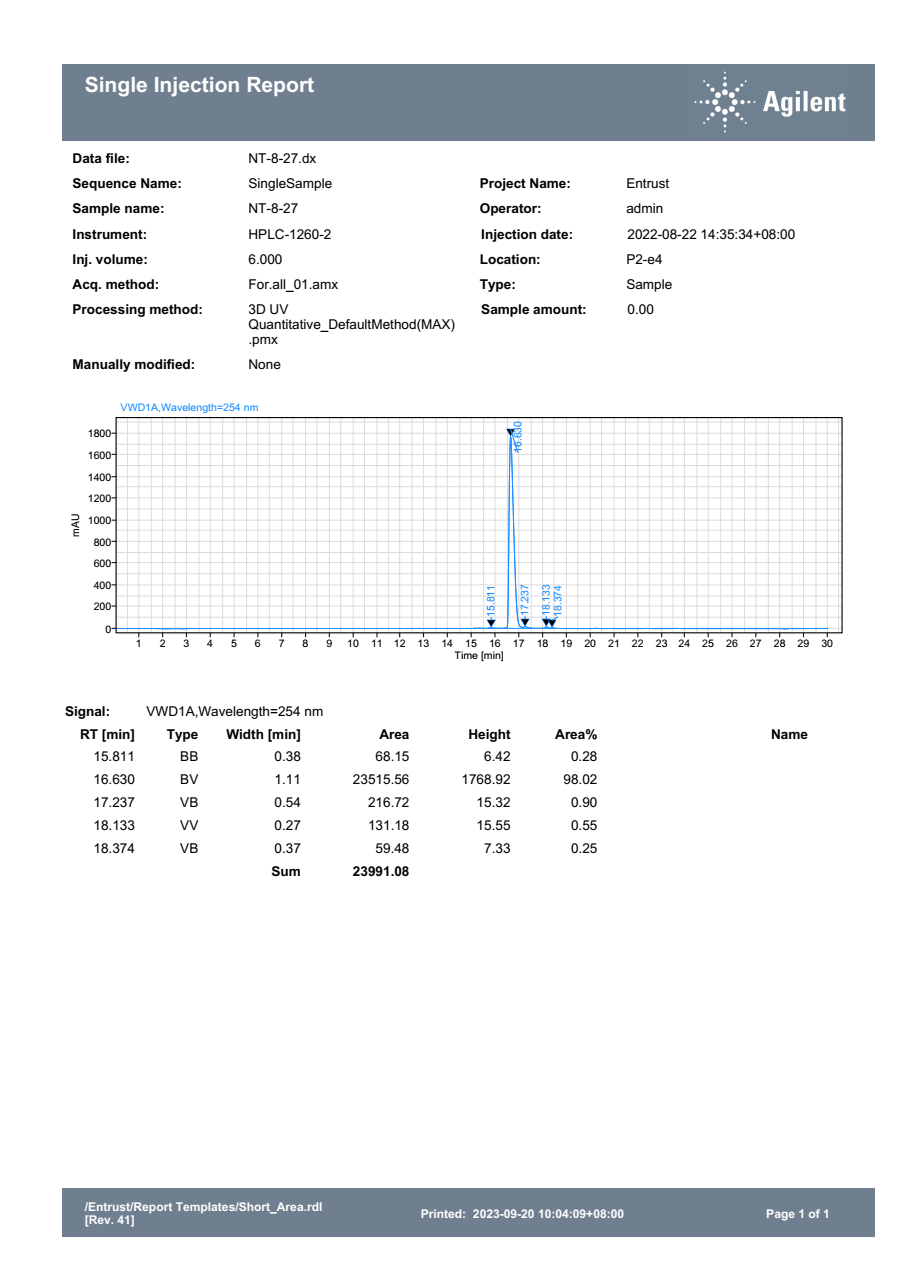


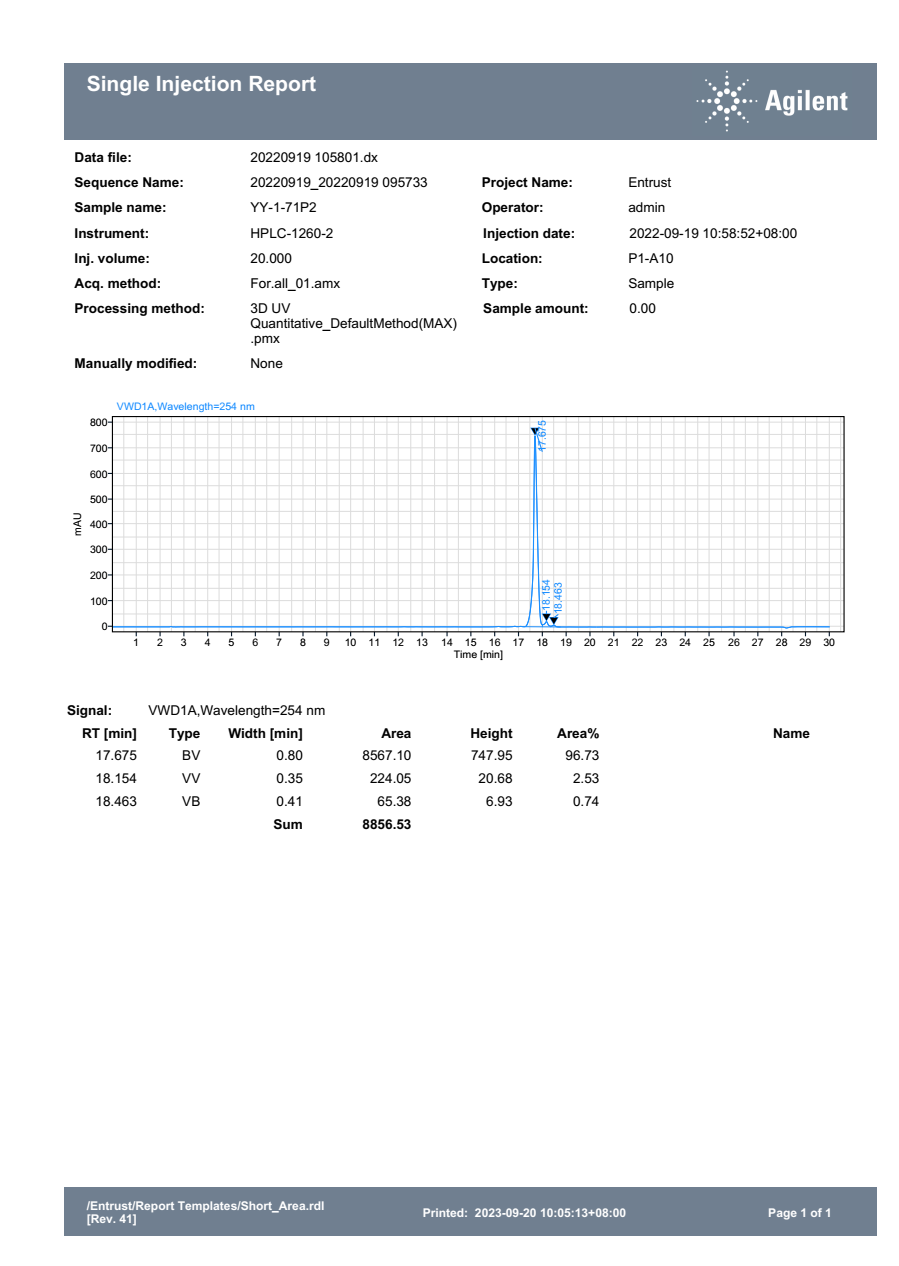


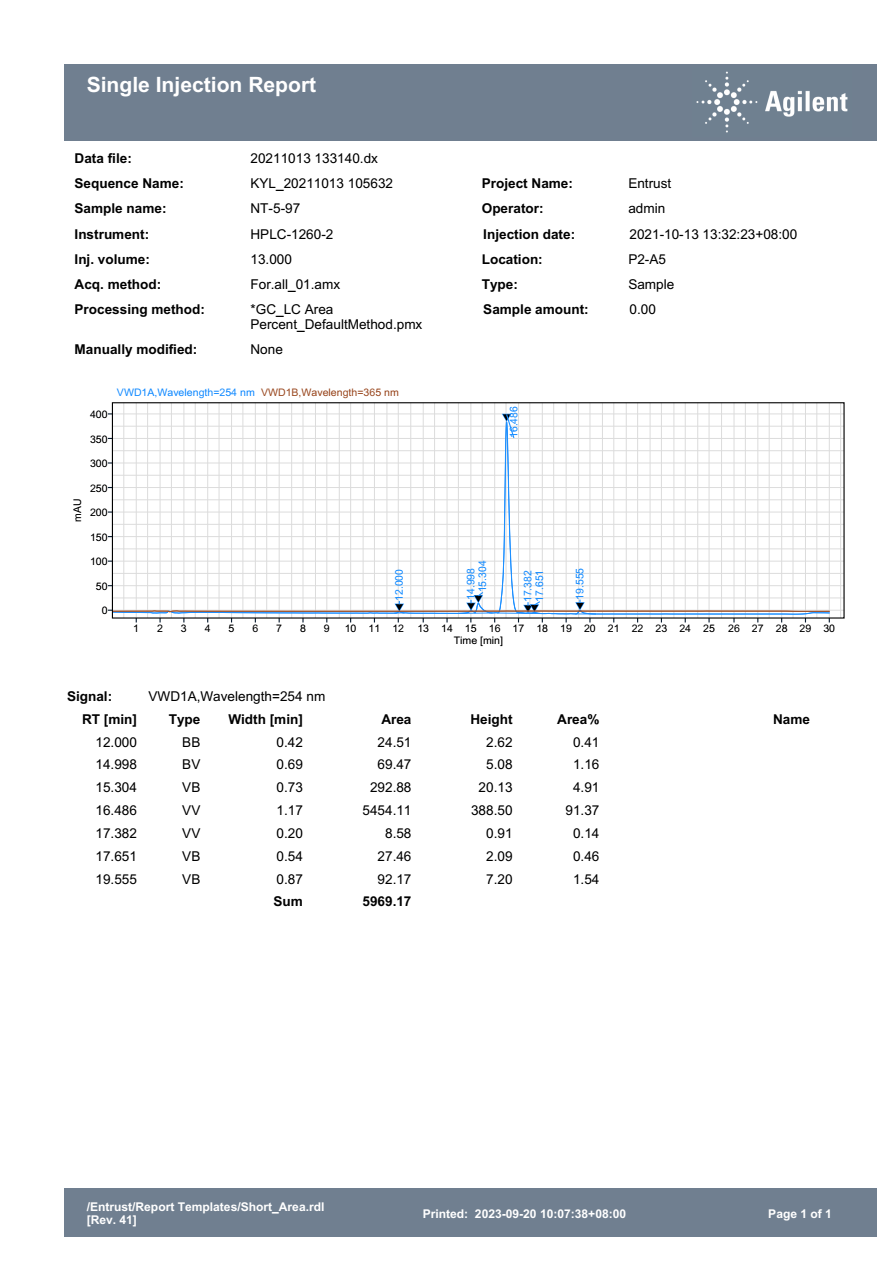


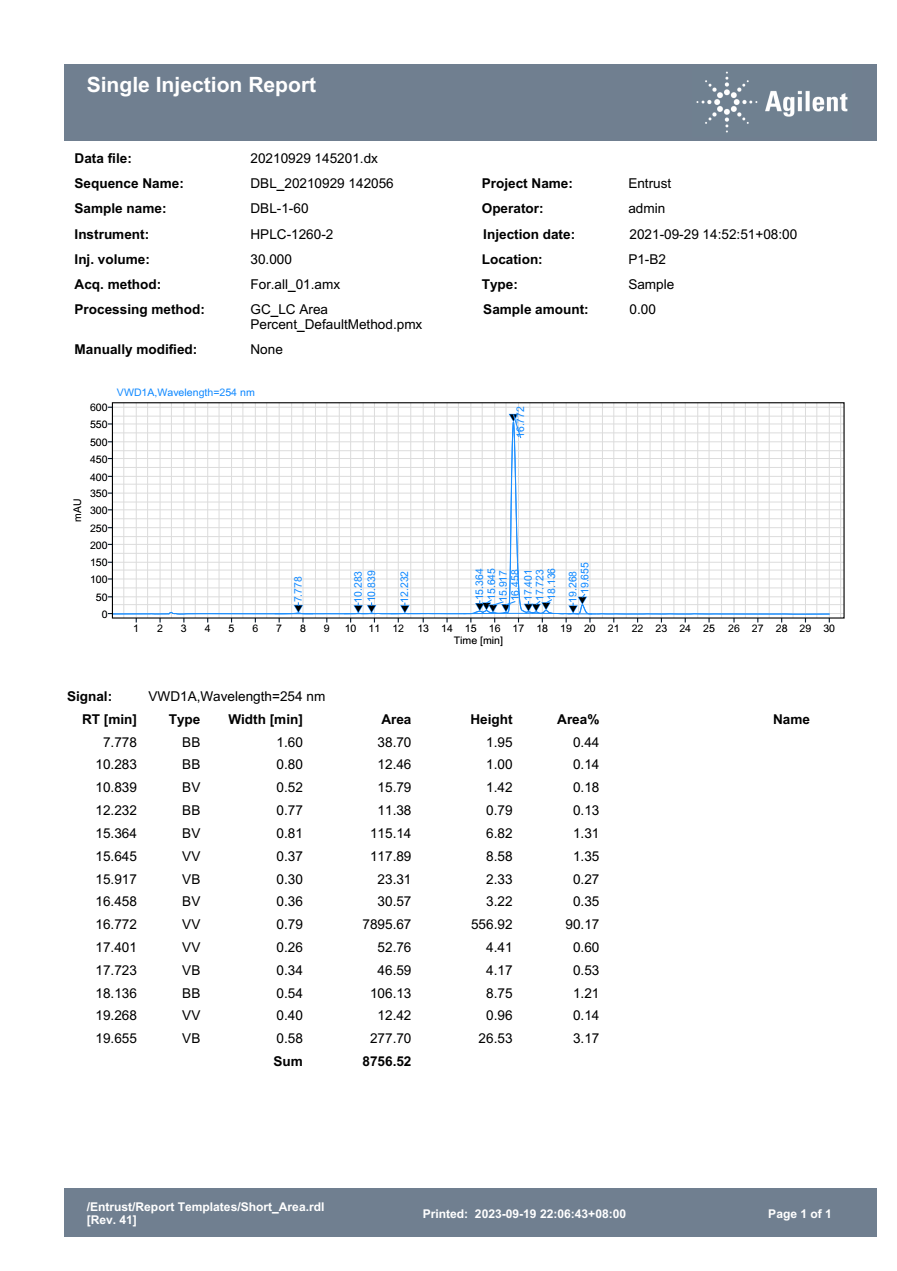


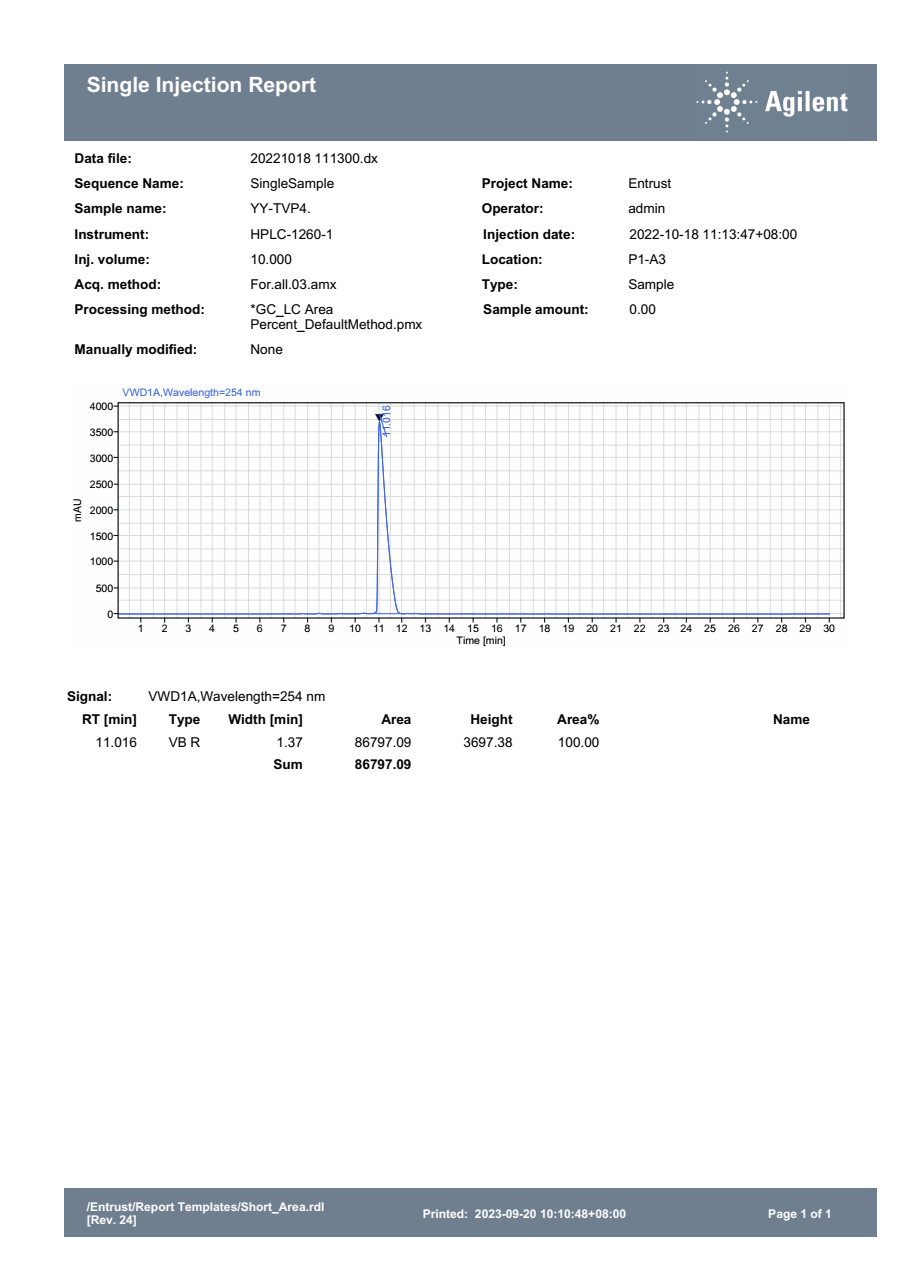


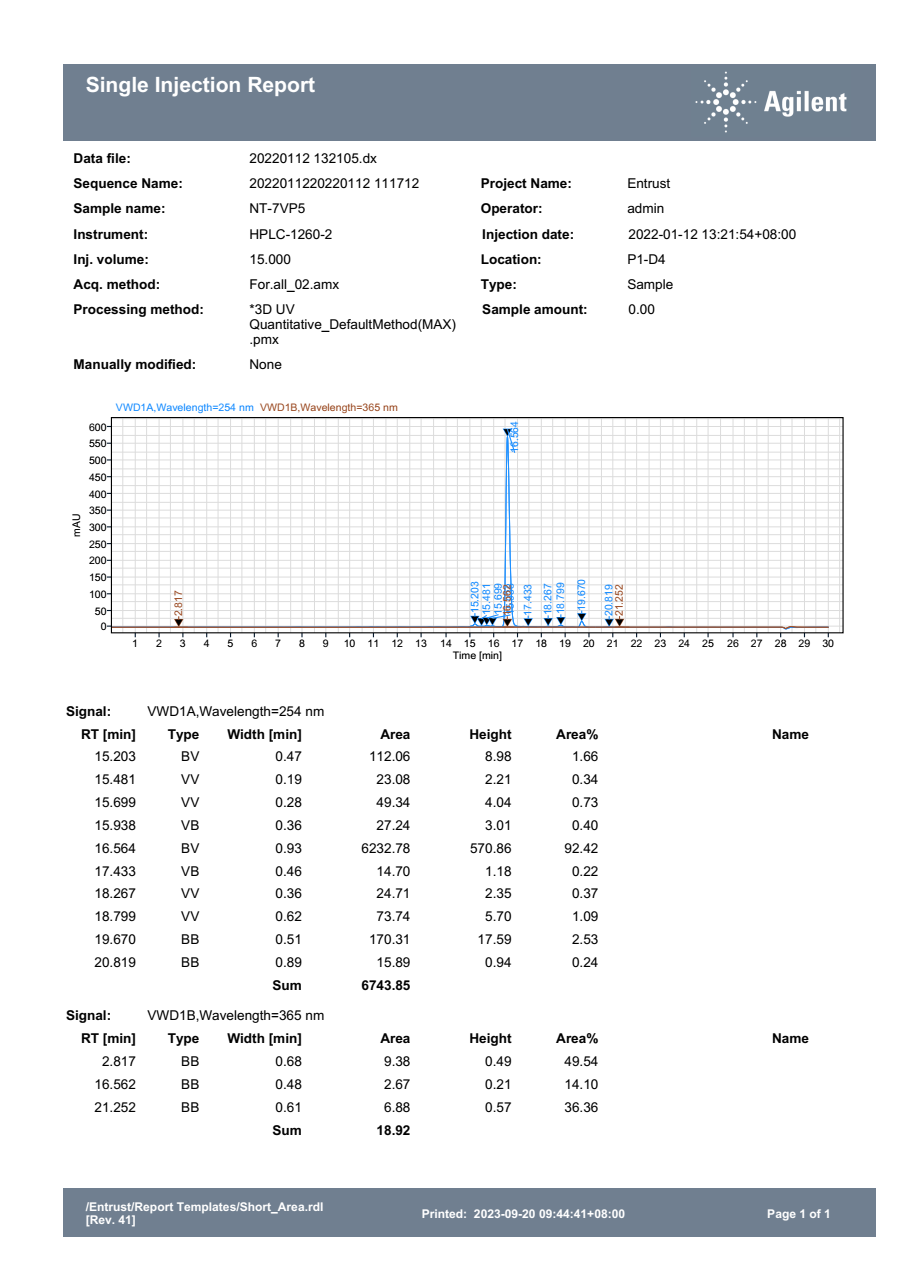


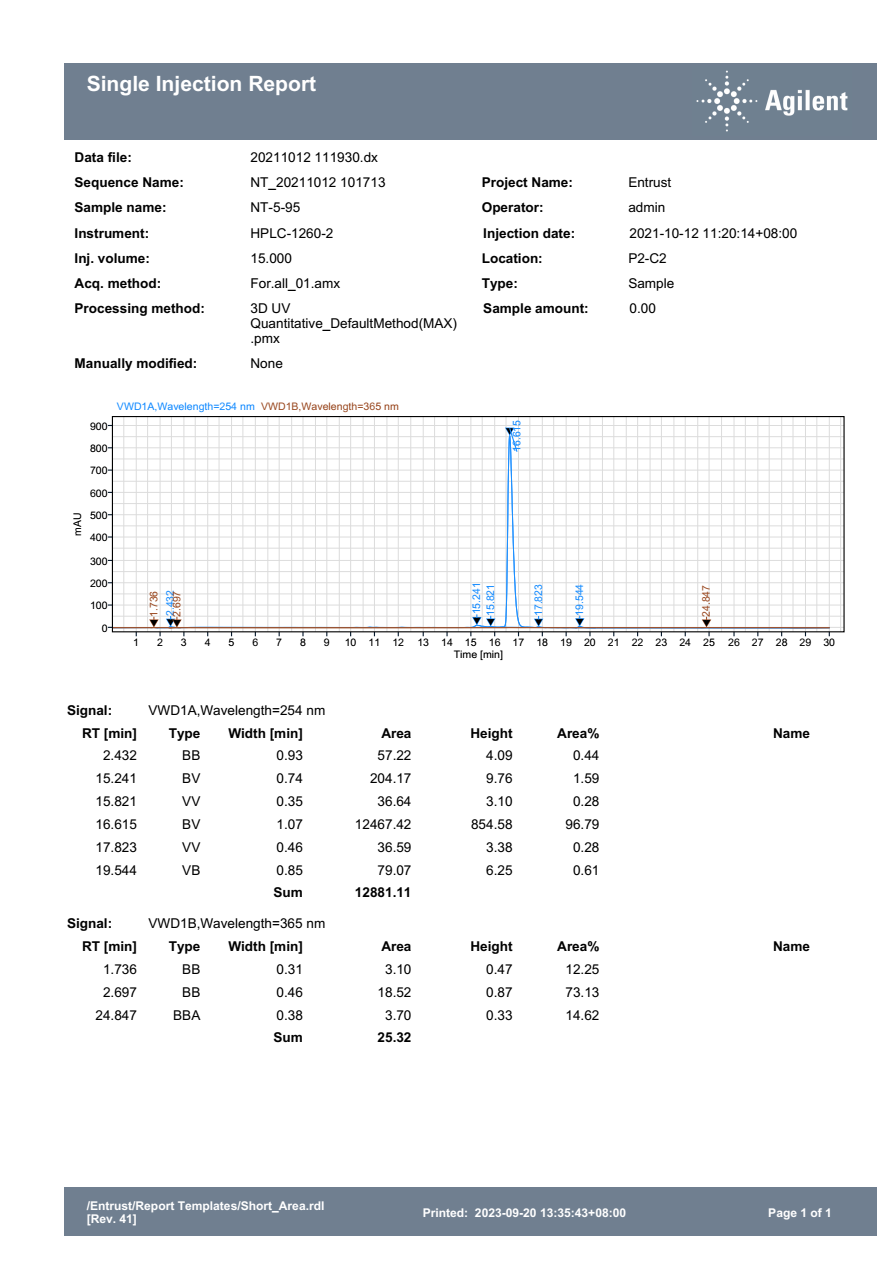


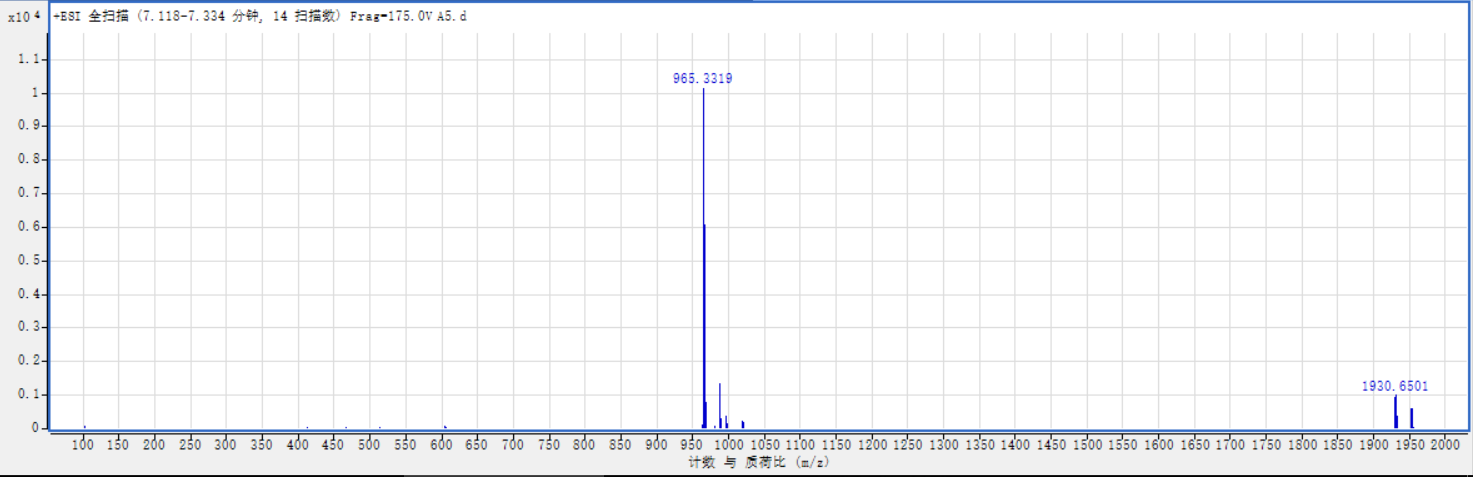


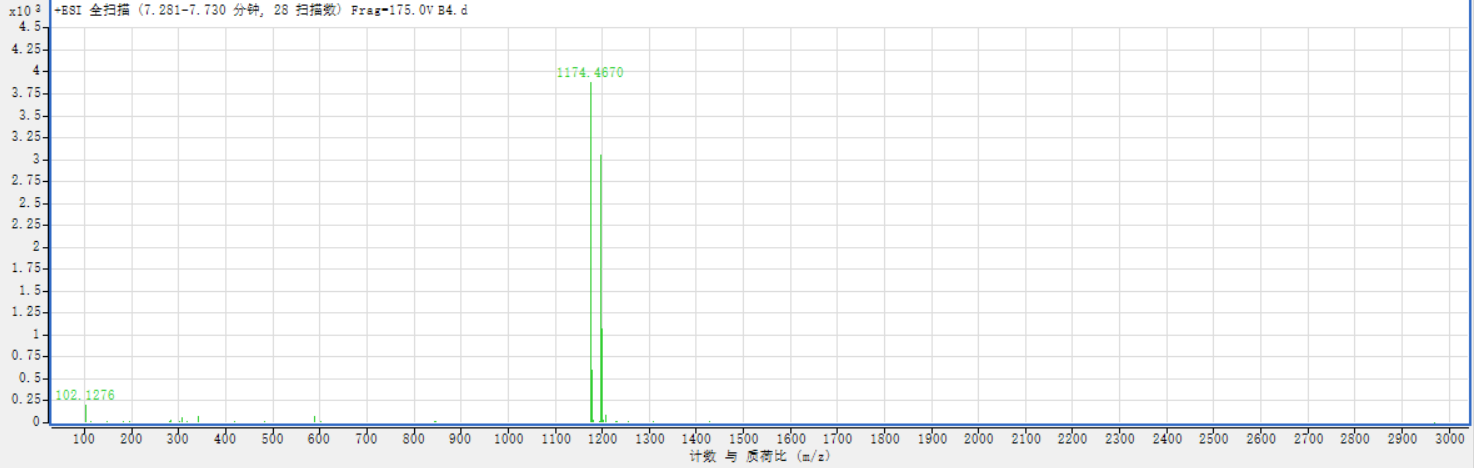


**G. References**

1. M. Lobera, K. P. Madauss, D. T. Pohlhaus, Q. G. Wright, M. Trocha, D. R. Schmidt, E. Baloglu, R. P. Trump, M. S. Head, G. A. Hofmann, M. Murray-Thompson, B. Schwartz, S. Chakravorty, Z. Wu, P. K. Mander, L. Kruidenier, R. A. Reid, W. Burkhart, B. J. Turunen, J. X. Rong, C. Wagner, M. B. Moyer, C. Wells, X. Hong, J. T. Moore, J. D. Williams, D. Soler, S. Ghosh, M. A. Nolan, *Nat. Chem. Biol*. **2013**, *9*, 319.
2. J. E. Bradner, N. West, M. L. Grachan, E. F. Greenberg, S. J. Haggarty, T. Warnow, R. Mazitschek, *Nat. Chem. Biol.* **2010**, *6*, 238.
3. Z. Rao, K. Li, J. Hong, D. Chen, B. Ding, L. Jiang, X. Qi, J. Hu, B. Yang, Q. He, X. Dong, J. Cao, C.-L. Zhu, *Eur. J. Med. Chem.* **2023**, *251*, 115248.
4. A. Bricelj, C. Steinebach, R. Kuchta, M. Gütschow, I. Sosič, *Front. Chem.* **2021**, *9*, 707317.
