# Supplementary material for: PROTAC‐Mediated HDAC7 Protein Degradation Unveils Its Deacetylase‐Independent Proinflammatory Function in Macrophages
Source: Adv Sci (Weinh). 2024 Jul 25;11(36):2309459. doi: 10.1002/advs.202309459 (PMC11423193; doi:10.1002/advs.202309459)
Supplement: Supplementary file 2 — Supplemental Tables 6‐7 [file ADVS-11-2309459-s002.docx]

**Supplemental Tables**

**PROTAC-mediated HDAC7 Protein Degradation Unveils Its Deacetylase-independent Proinflammatory Function in Macrophages**

Kailibinuer Kadier, Tian Niu, Baoli Ding, Boya Chen, Xuxin Qi, Danni Chen, Xirui Cheng, Yizheng Fang, Jiahao Zhou, Wenyi Zhao, Zeqi Liu, Yi Yuan, Zhan Zhou, Xiaowu Dong, Bo Yang, Qiaojun He, Ji Cao,* Li Jiang,* and Cheng-Liang Zhu*

**Table S6.** The quantitative mass spectrometry-based proteomes analyses for HDACs in RAW264.7 cells treated with **B4**

| **Protein**  **accession** | **Protein description** | **Gene**  **name** | **B4/DMSO Ratio** |
| --- | --- | --- | --- |
| Q501J7 | Phosphatase and actin regulator 4 OS=Mus musculus OX=10090 GN=Phactr4 PE=1 SV=2 | Phactr4 | 0.12 |
| Q5DTT3 | Protein TASOR 2 OS=Mus musculus OX=10090 GN=Tasor2 PE=1 SV=2 | Tasor2 | 0.12 |
| Q80US4 | Actin-related protein 5 OS=Mus musculus OX=10090 GN=Actr5 PE=1 SV=3 | Actr5 | 0.14 |
| Q8K2H1 | Periphilin-1 OS=Mus musculus OX=10090 GN=Pphln1 PE=1 SV=1 | Pphln1 | 0.18 |
| Q6P9P6 | Kinesin-like protein KIF11 OS=Mus musculus OX=10090 GN=Kif11 PE=1 SV=1 | Kif11 | 0.18 |
| Q921N8 | G protein pathway suppressor 2 OS=Mus musculus OX=10090 GN=Gps2 PE=1 SV=1 | Gps2 | 0.18 |
| Q9CXG9 | PHD finger protein 19 OS=Mus musculus OX=10090 GN=Phf19 PE=1 SV=1 | Phf19 | 0.21 |
| Q91VM3 | WD repeat domain phosphoinositide-interacting protein 4 OS=Mus musculus OX=10090 GN=Wdr45 PE=1 SV=1 | Wdr45 | 0.22 |
| Q8C6B9 | Active regulator of SIRT1 OS=Mus musculus OX=10090 GN=Rps19bp1 PE=1 SV=1 | Rps19bp1 | 0.22 |
| Q8R0K9 | Transcription factor E2F4 OS=Mus musculus OX=10090 GN=E2f4 PE=1 SV=1 | E2f4 | 0.23 |
| Q9CQU5 | ZW10 interactor OS=Mus musculus OX=10090 GN=Zwint PE=1 SV=1 | Zwint | 0.24 |
| Q7TPM1 | Protein PRRC2B OS=Mus musculus OX=10090 GN=Prrc2b PE=1 SV=1 | Prrc2b | 0.25 |
| Q9JLQ0 | CD2-associated protein OS=Mus musculus OX=10090 GN=Cd2ap PE=1 SV=3 | Cd2ap | 0.26 |
| Q8R2U2 | Nucleolus and neural progenitor protein OS=Mus musculus OX=10090 GN=Nepro PE=2 SV=1 | Nepro | 0.26 |
| Q08857 | Platelet glycoprotein 4 OS=Mus musculus OX=10090 GN=Cd36 PE=1 SV=2 | Cd36 | 0.26 |
| Q8C3X4 | Translation factor Guf1, mitochondrial OS=Mus musculus OX=10090 GN=Guf1 PE=1 SV=1 | Guf1 | 0.27 |
| Q9DBH0 | NEDD4-like E3 ubiquitin-protein ligase WWP2 OS=Mus musculus OX=10090 GN=Wwp2 PE=1 SV=1 | Wwp2 | 0.27 |
| Q99PU7 | Ubiquitin carboxyl-terminal hydrolase BAP1 OS=Mus musculus OX=10090 GN=Bap1 PE=1 SV=1 | Bap1 | 0.27 |
| Q9CR80 | Protein FAM32A OS=Mus musculus OX=10090 GN=Fam32a PE=2 SV=1 | Fam32a | 0.27 |
| A2AI05 | NADPH-dependent diflavin oxidoreductase 1 OS=Mus musculus OX=10090 GN=Ndor1 PE=2 SV=1 | Ndor1 | 0.28 |
| A2RSY1 | KAT8 regulatory NSL complex subunit 3 OS=Mus musculus OX=10090 GN=Kansl3 PE=1 SV=1 | Kansl3 | 0.28 |
| Q91VL8 | Telomeric repeat-binding factor 2-interacting protein 1 OS=Mus musculus OX=10090 GN=Terf2ip PE=1 SV=1 | Terf2ip | 0.28 |
| B1AZP2 | Disks large-associated protein 4 OS=Mus musculus OX=10090 GN=Dlgap4 PE=1 SV=1 | Dlgap4 | 0.30 |
| Q8R2R1 | Protein O-mannosyl-transferase 1 OS=Mus musculus OX=10090 GN=Pomt1 PE=1 SV=1 | Pomt1 | 0.30 |
| O08715 | A-kinase anchor protein 1, mitochondrial OS=Mus musculus OX=10090 GN=Akap1 PE=1 SV=4 | Akap1 | 0.31 |
| Q9CQA9 | Cancer-related nucleoside-triphosphatase homolog OS=Mus musculus OX=10090 GN=Ntpcr PE=1 SV=1 | Ntpcr | 0.34 |
| A2AJI0 | MAP7 domain-containing protein 1 OS=Mus musculus OX=10090 GN=Map7d1 PE=1 SV=1 | Map7d1 | 0.34 |
| Q91VN1 | Zinc finger protein 24 OS=Mus musculus OX=10090 GN=Znf24 PE=1 SV=1 | Znf24 | 0.35 |
| Q6PG16 | Holliday junction recognition protein OS=Mus musculus OX=10090 GN=Hjurp PE=1 SV=1 | Hjurp | 0.35 |
| Q8BP71 | RNA binding protein fox-1 homolog 2 OS=Mus musculus OX=10090 GN=Rbfox2 PE=1 SV=2 | Rbfox2 | 0.36 |
| Q9CS00 | Cactin OS=Mus musculus OX=10090 GN=Cactin PE=1 SV=2 | Cactin | 0.36 |
| Q62417 | Sorbin and SH3 domain-containing protein 1 OS=Mus musculus OX=10090 GN=Sorbs1 PE=1 SV=2 | Sorbs1 | 0.36 |
| Q9WTU0 | Lysine-specific demethylase PHF2 OS=Mus musculus OX=10090 GN=Phf2 PE=1 SV=2 | Phf2 | 0.38 |
| Q3UHB1 | 5'-nucleotidase domain-containing protein 3 OS=Mus musculus OX=10090 GN=Nt5dc3 PE=1 SV=1 | Nt5dc3 | 0.38 |
| Q9D7W5 | Mediator of RNA polymerase II transcription subunit 8 OS=Mus musculus OX=10090 GN=Med8 PE=1 SV=1 | Med8 | 0.38 |
| Q9CQU0 | Thioredoxin domain-containing protein 12 OS=Mus musculus OX=10090 GN=Txndc12 PE=1 SV=1 | Txndc12 | 0.38 |
| Q8BL48 | RING finger protein unkempt homolog OS=Mus musculus OX=10090 GN=Unk PE=1 SV=1 | Unk | 0.38 |
| Q9Z2E1 | Methyl-CpG-binding domain protein 2 OS=Mus musculus OX=10090 GN=Mbd2 PE=2 SV=2 | Mbd2 | 0.39 |
| Q8R1F9 | Ribonuclease P protein subunit p40 OS=Mus musculus OX=10090 GN=Rpp40 PE=2 SV=2 | Rpp40 | 0.39 |
| P60762 | Mortality factor 4-like protein 1 OS=Mus musculus OX=10090 GN=Morf4l1 PE=1 SV=2 | Morf4l1 | 0.39 |
| A6H619 | PHD and RING finger domain-containing protein 1 OS=Mus musculus OX=10090 GN=Phrf1 PE=1 SV=2 | Phrf1 | 0.40 |
| Q8K3Z9 | Nuclear envelope pore membrane protein POM 121 OS=Mus musculus OX=10090 GN=Pom121 PE=1 SV=2 | Pom121 | 0.41 |
| P59110 | Sentrin-specific protease 1 OS=Mus musculus OX=10090 GN=Senp1 PE=1 SV=1 | Senp1 | 0.42 |
| Q9JKP7 | DNA polymerase epsilon subunit 3 OS=Mus musculus OX=10090 GN=Pole3 PE=1 SV=1 | Pole3 | 0.43 |
| Q6KAQ7 | ZZ-type zinc finger-containing protein 3 OS=Mus musculus OX=10090 GN=Zzz3 PE=1 SV=2 | Zzz3 | 0.43 |
| Q9D084 | Centromere protein S OS=Mus musculus OX=10090 GN=Cenps PE=2 SV=1 | Cenps | 0.43 |
| Q3UD01 | Ataxin-7-like protein 3B OS=Mus musculus OX=10090 GN=Atxn7l3b PE=1 SV=1 | Atxn7l3b | 0.44 |
| Q9Z265 | Serine/threonine-protein kinase Chk2 OS=Mus musculus OX=10090 GN=Chek2 PE=1 SV=1 | Chek2 | 0.44 |
| Q9QUG2 | DNA polymerase kappa OS=Mus musculus OX=10090 GN=Polk PE=1 SV=1 | Polk | 0.45 |
| P47809 | Dual specificity mitogen-activated protein kinase kinase 4 OS=Mus musculus OX=10090 GN=Map2k4 PE=1 SV=2 | Map2k4 | 0.45 |
| Q8R3L8 | Cyclin-dependent kinase 8 OS=Mus musculus OX=10090 GN=Cdk8 PE=1 SV=3 | Cdk8 | 0.45 |
| Q8K400 | Syntaxin-binding protein 5 OS=Mus musculus OX=10090 GN=Stxbp5 PE=1 SV=3 | Stxbp5 | 0.45 |
| Q8CCN5 | BCAS3 microtubule associated cell migration factor OS=Mus musculus OX=10090 GN=Bcas3 PE=1 SV=2 | Bcas3 | 0.45 |
| Q0VBD2 | Protein MCM10 homolog OS=Mus musculus OX=10090 GN=Mcm10 PE=1 SV=1 | Mcm10 | 0.45 |
| Q78JW9 | Ubiquitin domain-containing protein UBFD1 OS=Mus musculus OX=10090 GN=Ubfd1 PE=1 SV=2 | Ubfd1 | 0.45 |
| Q9D7B1 | tRNA-dihydrouridine(20) synthase [NAD(P)+]-like OS=Mus musculus OX=10090 GN=Dus2 PE=1 SV=1 | Dus2 | 0.46 |
| Q3TUH1 | Phosphatidate cytidylyltransferase, mitochondrial OS=Mus musculus OX=10090 GN=Tamm41 PE=1 SV=2 | Tamm41 | 0.46 |
| Q3TV65 | MPN domain-containing protein OS=Mus musculus OX=10090 GN=Mpnd PE=2 SV=2 | Mpnd | 0.46 |
| Q8K2C8 | Glycerol-3-phosphate acyltransferase 4 OS=Mus musculus OX=10090 GN=Gpat4 PE=1 SV=1 | Gpat4 | 0.47 |
| Q91WT8 | RNA-binding protein 47 OS=Mus musculus OX=10090 GN=Rbm47 PE=1 SV=1 | Rbm47 | 0.47 |
| Q8CIB9 | N-acetyltransferase ESCO2 OS=Mus musculus OX=10090 GN=Esco2 PE=1 SV=3 | Esco2 | 0.47 |
| Q8JZS9 | 39S ribosomal protein L48, mitochondrial OS=Mus musculus OX=10090 GN=Mrpl48 PE=1 SV=1 | Mrpl48 | 0.47 |
| Q8BVN4 | Transcription termination factor 4, mitochondrial OS=Mus musculus OX=10090 GN=Mterf4 PE=2 SV=1 | Mterf4 | 0.47 |
| Q9WTP7 | GTP:AMP phosphotransferase AK3, mitochondrial OS=Mus musculus OX=10090 GN=Ak3 PE=1 SV=3 | Ak3 | 0.48 |
| A6PWY4 | WD repeat-containing protein 76 OS=Mus musculus OX=10090 GN=Wdr76 PE=1 SV=1 | Wdr76 | 0.48 |
| O35166 | Golgi SNAP receptor complex member 2 OS=Mus musculus OX=10090 GN=Gosr2 PE=1 SV=2 | Gosr2 | 0.48 |
| Q9D1P2 | Histone acetyltransferase KAT8 OS=Mus musculus OX=10090 GN=Kat8 PE=1 SV=1 | Kat8 | 0.48 |
| F8VPQ2 | AT-rich interactive domain-containing protein 4A OS=Mus musculus OX=10090 GN=Arid4a PE=1 SV=1 | Arid4a | 0.49 |
| Q9CR02 | Translation machinery-associated protein 16 OS=Mus musculus OX=10090 GN=Tma16 PE=1 SV=1 | Tma16 | 0.49 |
| Q3U0V2 | Tumor necrosis factor receptor type 1-associated DEATH domain protein OS=Mus musculus OX=10090 GN=Tradd PE=1 SV=1 | Tradd | 0.49 |
| Q9D4H9 | PHD finger protein 14 OS=Mus musculus OX=10090 GN=Phf14 PE=1 SV=1 | Phf14 | 0.49 |
| Q8VCD5 | Mediator of RNA polymerase II transcription subunit 17 OS=Mus musculus OX=10090 GN=Med17 PE=1 SV=1 | Med17 | 0.49 |
| Q8R3I3 | Conserved oligomeric Golgi complex subunit 6 OS=Mus musculus OX=10090 GN=Cog6 PE=1 SV=2 | Cog6 | 0.49 |
| Q91XB7 | Protein YIF1A OS=Mus musculus OX=10090 GN=Yif1a PE=1 SV=1 | Yif1a | 0.49 |
| Q9DCD6 | Gamma-aminobutyric acid receptor-associated protein OS=Mus musculus OX=10090 GN=Gabarap PE=1 SV=2 | Gabarap | 0.49 |
| Q9WU62 | Inner centromere protein OS=Mus musculus OX=10090 GN=Incenp PE=1 SV=2 | Incenp | 0.50 |
| Q8BZB2 | Phosphopantothenoylcysteine decarboxylase OS=Mus musculus OX=10090 GN=Ppcdc PE=1 SV=1 | Ppcdc | 0.50 |
| Q64705 | Upstream stimulatory factor 2 OS=Mus musculus OX=10090 GN=Usf2 PE=1 SV=1 | Usf2 | 0.50 |
| Q8BY89 | Choline transporter-like protein 2 OS=Mus musculus OX=10090 GN=Slc44a2 PE=1 SV=2 | Slc44a2 | 0.50 |
| Q14B71 | Cell division cycle-associated protein 2 OS=Mus musculus OX=10090 GN=Cdca2 PE=1 SV=2 | Cdca2 | 0.50 |
| Q8K221 | Arfaptin-2 OS=Mus musculus OX=10090 GN=Arfip2 PE=1 SV=2 | Arfip2 | 0.50 |
| Q8CI32 | BAG family molecular chaperone regulator 5 OS=Mus musculus OX=10090 GN=Bag5 PE=1 SV=1 | Bag5 | 0.51 |
| Q8BGB2 | Tetratricopeptide repeat protein 7A OS=Mus musculus OX=10090 GN=Ttc7a PE=1 SV=1 | Ttc7a | 0.51 |
| Q8CI08 | SLAIN motif-containing protein 2 OS=Mus musculus OX=10090 GN=Slain2 PE=1 SV=2 | Slain2 | 0.51 |
| Q8CI70 | Leucine-rich repeat-containing protein 20 OS=Mus musculus OX=10090 GN=Lrrc20 PE=1 SV=1 | Lrrc20 | 0.51 |
| Q61542 | StAR-related lipid transfer protein 3 OS=Mus musculus OX=10090 GN=Stard3 PE=1 SV=1 | Stard3 | 0.51 |
| Q3U5Q7 | UMP-CMP kinase 2, mitochondrial OS=Mus musculus OX=10090 GN=Cmpk2 PE=1 SV=2 | Cmpk2 | 0.51 |
| P48754 | Breast cancer type 1 susceptibility protein homolog OS=Mus musculus OX=10090 GN=Brca1 PE=1 SV=3 | Brca1 | 0.51 |
| P23336 | N-acetyllactosaminide alpha-1,3-galactosyltransferase OS=Mus musculus OX=10090 GN=Ggta1 PE=2 SV=1 | Ggta1 | 0.51 |
| Q9D2Y4 | Mixed lineage kinase domain-like protein OS=Mus musculus OX=10090 GN=Mlkl PE=1 SV=1 | Mlkl | 0.51 |
| Q5ND52 | rRNA methyltransferase 3, mitochondrial OS=Mus musculus OX=10090 GN=Mrm3 PE=2 SV=1 | Mrm3 | 0.52 |
| Q61712 | DnaJ homolog subfamily C member 1 OS=Mus musculus OX=10090 GN=Dnajc1 PE=1 SV=1 | Dnajc1 | 0.52 |
| Q9WTK3 | Glycosylphosphatidylinositol anchor attachment 1 protein OS=Mus musculus OX=10090 GN=Gpaa1 PE=1 SV=3 | Gpaa1 | 0.52 |
| P43277 | Histone H1.3 OS=Mus musculus OX=10090 GN=H1-3 PE=1 SV=2 | H1-3 | 0.53 |
| Q6ZWY3 | 40S ribosomal protein S27-like OS=Mus musculus OX=10090 GN=Rps27l PE=1 SV=3 | Rps27l | 0.53 |
| Q99LB0 | Deoxynucleotidyltransferase terminal-interacting protein 1 OS=Mus musculus OX=10090 GN=Dnttip1 PE=1 SV=1 | Dnttip1 | 0.53 |
| Q9D6K9 | Ceramide synthase 5 OS=Mus musculus OX=10090 GN=Cers5 PE=1 SV=1 | Cers5 | 0.53 |
| Q3TYA6 | M-phase phosphoprotein 8 OS=Mus musculus OX=10090 GN=Mphosph8 PE=1 SV=1 | Mphosph8 | 0.53 |
| Q9WVL6 | Exostosin-like 3 OS=Mus musculus OX=10090 GN=Extl3 PE=1 SV=2 | Extl3 | 0.53 |
| Q9CPX7 | 28S ribosomal protein S16, mitochondrial OS=Mus musculus OX=10090 GN=Mrps16 PE=1 SV=1 | Mrps16 | 0.53 |
| Q7TPH6 | E3 ubiquitin-protein ligase MYCBP2 OS=Mus musculus OX=10090 GN=Mycbp2 PE=1 SV=3 | Mycbp2 | 0.53 |
| Q9CQL6 | 39S ribosomal protein L35, mitochondrial OS=Mus musculus OX=10090 GN=Mrpl35 PE=2 SV=1 | Mrpl35 | 0.54 |
| Q9DCI3 | STARD3 N-terminal-like protein OS=Mus musculus OX=10090 GN=Stard3nl PE=1 SV=2 | Stard3nl | 0.54 |
| Q91XL9 | Oxysterol-binding protein-related protein 1 OS=Mus musculus OX=10090 GN=Osbpl1a PE=1 SV=2 | Osbpl1a | 0.54 |
| Q8CIC2 | Nucleoporin NUP42 OS=Mus musculus OX=10090 GN=Nup42 PE=2 SV=1 | Nup42 | 0.54 |
| A6H5Y3 | Methionine synthase OS=Mus musculus OX=10090 GN=Mtr PE=1 SV=1 | Mtr | 0.54 |
| Q8BGQ1 | Spermatogenesis-defective protein 39 homolog OS=Mus musculus OX=10090 GN=Vipas39 PE=1 SV=1 | Vipas39 | 0.54 |
| Q4VBD2 | Transmembrane anterior posterior transformation protein 1 OS=Mus musculus OX=10090 GN=Tapt1 PE=1 SV=2 | Tapt1 | 0.54 |
| Q3UHC2 | Leucine-rich repeat serine/threonine-protein kinase 1 OS=Mus musculus OX=10090 GN=Lrrk1 PE=1 SV=1 | Lrrk1 | 0.54 |
| Q791T5 | Mitochondrial carrier homolog 1 OS=Mus musculus OX=10090 GN=Mtch1 PE=1 SV=1 | Mtch1 | 0.54 |
| Q8BVI5 | Syntaxin-16 OS=Mus musculus OX=10090 GN=Stx16 PE=1 SV=3 | Stx16 | 0.54 |
| Q9R0G7 | Zinc finger E-box-binding homeobox 2 OS=Mus musculus OX=10090 GN=Zeb2 PE=1 SV=2 | Zeb2 | 0.54 |
| Q8R4H9 | Zinc transporter 5 OS=Mus musculus OX=10090 GN=Slc30a5 PE=1 SV=1 | Slc30a5 | 0.55 |
| Q8VCH6 | Delta(24)-sterol reductase OS=Mus musculus OX=10090 GN=Dhcr24 PE=1 SV=1 | Dhcr24 | 0.55 |
| O54786 | DNA fragmentation factor subunit alpha OS=Mus musculus OX=10090 GN=Dffa PE=1 SV=2 | Dffa | 0.55 |
| Q9DAV9 | Trimeric intracellular cation channel type B OS=Mus musculus OX=10090 GN=Tmem38b PE=1 SV=1 | Tmem38b | 0.55 |
| P84089 | Enhancer of rudimentary homolog OS=Mus musculus OX=10090 GN=Erh PE=1 SV=1 | Erh | 0.55 |
| Q3U6N9 | UPF0488 protein C8orf33 homolog OS=Mus musculus OX=10090 PE=2 SV=1 | -- | 0.55 |
| P62075 | Mitochondrial import inner membrane translocase subunit Tim13 OS=Mus musculus OX=10090 GN=Timm13 PE=1 SV=1 | Timm13 | 0.55 |
| P97808 | FXYD domain-containing ion transport regulator 5 OS=Mus musculus OX=10090 GN=Fxyd5 PE=1 SV=3 | Fxyd5 | 0.55 |
| Q6PFD6 | Kinesin-like protein KIF18B OS=Mus musculus OX=10090 GN=Kif18b PE=2 SV=2 | Kif18b | 0.55 |
| Q4VC33 | E3 ubiquitin-protein transferase MAEA OS=Mus musculus OX=10090 GN=Maea PE=1 SV=1 | Maea | 0.55 |
| Q8CIM5 | G-protein coupled receptor 84 OS=Mus musculus OX=10090 GN=Gpr84 PE=1 SV=1 | Gpr84 | 0.56 |
| Q8K3G5 | Inactive serine/threonine-protein kinase VRK3 OS=Mus musculus OX=10090 GN=Vrk3 PE=1 SV=2 | Vrk3 | 0.56 |
| Q9QZA0 | Carbonic anhydrase 5B, mitochondrial OS=Mus musculus OX=10090 GN=Ca5b PE=1 SV=2 | Ca5b | 0.56 |
| E9PY46 | Intraflagellar transport protein 140 homolog OS=Mus musculus OX=10090 GN=Ift140 PE=1 SV=1 | Ift140 | 0.56 |
| A2CG63 | AT-rich interactive domain-containing protein 4B OS=Mus musculus OX=10090 GN=Arid4b PE=1 SV=1 | Arid4b | 0.56 |
| P50171 | (3R)-3-hydroxyacyl-CoA dehydrogenase OS=Mus musculus OX=10090 GN=Hsd17b8 PE=1 SV=2 | Hsd17b8 | 0.56 |
| Q99LT0 | Protein dpy-30 homolog OS=Mus musculus OX=10090 GN=Dpy30 PE=1 SV=1 | Dpy30 | 0.56 |
| P04202 | Transforming growth factor beta-1 proprotein OS=Mus musculus OX=10090 GN=Tgfb1 PE=1 SV=1 | Tgfb1 | 0.56 |
| P62849 | 40S ribosomal protein S24 OS=Mus musculus OX=10090 GN=Rps24 PE=1 SV=1 | Rps24 | 0.57 |
| Q9DAM7 | Transmembrane protein 263 OS=Mus musculus OX=10090 GN=Tmem263 PE=1 SV=1 | Tmem263 | 0.57 |
| Q9D0S9 | Adenosine 5'-monophosphoramidase HINT2 OS=Mus musculus OX=10090 GN=Hint2 PE=1 SV=1 | Hint2 | 0.57 |
| Q61578 | NADPH:adrenodoxin oxidoreductase, mitochondrial OS=Mus musculus OX=10090 GN=Fdxr PE=1 SV=1 | Fdxr | 0.57 |
| O35969 | Guanidinoacetate N-methyltransferase OS=Mus musculus OX=10090 GN=Gamt PE=1 SV=1 | Gamt | 0.57 |
| P70257 | Nuclear factor 1 X-type OS=Mus musculus OX=10090 GN=Nfix PE=1 SV=2 | Nfix | 0.57 |
| Q9ESC8 | AF4/FMR2 family member 4 OS=Mus musculus OX=10090 GN=Aff4 PE=1 SV=1 | Aff4 | 0.57 |
| O08756 | 3-hydroxyacyl-CoA dehydrogenase type-2 OS=Mus musculus OX=10090 GN=Hsd17b10 PE=1 SV=4 | Hsd17b10 | 0.57 |
| Q9CXD6 | Mitochondrial calcium uniporter regulator 1 OS=Mus musculus OX=10090 GN=Mcur1 PE=1 SV=1 | Mcur1 | 0.57 |
| Q922Q2 | Serine/threonine-protein kinase RIO1 OS=Mus musculus OX=10090 GN=Riok1 PE=1 SV=2 | Riok1 | 0.58 |
| Q80VQ0 | Aldehyde dehydrogenase family 3 member B1 OS=Mus musculus OX=10090 GN=Aldh3b1 PE=1 SV=1 | Aldh3b1 | 0.58 |
| Q8VDK1 | Deaminated glutathione amidase OS=Mus musculus OX=10090 GN=Nit1 PE=1 SV=2 | Nit1 | 0.58 |
| B1AR13 | CDGSH iron-sulfur domain-containing protein 3, mitochondrial OS=Mus musculus OX=10090 GN=Cisd3 PE=1 SV=1 | Cisd3 | 0.58 |
| Q3TDQ1 | Dolichyl-diphosphooligosaccharide--protein glycosyltransferase subunit STT3B OS=Mus musculus OX=10090 GN=Stt3b PE=1 SV=2 | Stt3b | 0.58 |
| Q99LJ0 | CTTNBP2 N-terminal-like protein OS=Mus musculus OX=10090 GN=Cttnbp2nl PE=1 SV=1 | Cttnbp2nl | 0.58 |
| P43274 | Histone H1.4 OS=Mus musculus OX=10090 GN=H1-4 PE=1 SV=2 | H1-4 | 0.58 |
| Q9D2R6 | Cytochrome c oxidase assembly factor 3 homolog, mitochondrial OS=Mus musculus OX=10090 GN=Coa3 PE=1 SV=1 | Coa3 | 0.58 |
| P83093 | Stromal interaction molecule 2 OS=Mus musculus OX=10090 GN=Stim2 PE=1 SV=2 | Stim2 | 0.58 |
| O35144 | Telomeric repeat-binding factor 2 OS=Mus musculus OX=10090 GN=Terf2 PE=1 SV=3 | Terf2 | 0.58 |
| Q9WUU9 | Germinal-center associated nuclear protein OS=Mus musculus OX=10090 GN=Mcm3ap PE=1 SV=2 | Mcm3ap | 0.58 |
| Q9CQ49 | Nuclear cap-binding protein subunit 2 OS=Mus musculus OX=10090 GN=Ncbp2 PE=1 SV=1 | Ncbp2 | 0.58 |
| Q9JK48 | Endophilin-B1 OS=Mus musculus OX=10090 GN=Sh3glb1 PE=1 SV=1 | Sh3glb1 | 0.58 |
| Q9DBU6 | Serine/Arginine-related protein 53 OS=Mus musculus OX=10090 GN=Rsrc1 PE=1 SV=1 | Rsrc1 | 0.58 |
| Q91YR9 | Prostaglandin reductase 1 OS=Mus musculus OX=10090 GN=Ptgr1 PE=1 SV=2 | Ptgr1 | 0.58 |
| Q99N89 | 39S ribosomal protein L43, mitochondrial OS=Mus musculus OX=10090 GN=Mrpl43 PE=1 SV=1 | Mrpl43 | 0.59 |
| Q8BKI2 | Trinucleotide repeat-containing gene 6B protein OS=Mus musculus OX=10090 GN=Tnrc6b PE=1 SV=2 | Tnrc6b | 0.59 |
| Q8C7E9 | Cleavage stimulation factor subunit 2 tau variant OS=Mus musculus OX=10090 GN=Cstf2t PE=1 SV=2 | Cstf2t | 0.59 |
| Q60664 | Inositol 1,4,5-triphosphate receptor associated 2 OS=Mus musculus OX=10090 GN=Irag2 PE=1 SV=1 | Irag2 | 0.59 |
| Q91ZR2 | Sorting nexin-18 OS=Mus musculus OX=10090 GN=Snx18 PE=1 SV=1 | Snx18 | 0.59 |
| Q9ES56 | Trafficking protein particle complex subunit 4 OS=Mus musculus OX=10090 GN=Trappc4 PE=1 SV=1 | Trappc4 | 0.59 |
| Q9DD18 | D-aminoacyl-tRNA deacylase 1 OS=Mus musculus OX=10090 GN=Dtd1 PE=1 SV=2 | Dtd1 | 0.59 |
| P97315 | Cysteine and glycine-rich protein 1 OS=Mus musculus OX=10090 GN=Csrp1 PE=1 SV=3 | Csrp1 | 0.59 |
| Q9WVD5 | Mitochondrial ornithine transporter 1 OS=Mus musculus OX=10090 GN=Slc25a15 PE=1 SV=1 | Slc25a15 | 0.59 |
| Q6X893 | Choline transporter-like protein 1 OS=Mus musculus OX=10090 GN=Slc44a1 PE=1 SV=3 | Slc44a1 | 0.59 |
| Q9JKK1 | Syntaxin-6 OS=Mus musculus OX=10090 GN=Stx6 PE=1 SV=1 | Stx6 | 0.59 |
| F8VQB6 | Unconventional myosin-X OS=Mus musculus OX=10090 GN=Myo10 PE=1 SV=1 | Myo10 | 0.59 |
| Q3UY34 | Protein CUSTOS OS=Mus musculus OX=10090 GN=Custos PE=2 SV=1 | Custos | 0.59 |
| Q8VE97 | Serine/arginine-rich splicing factor 4 OS=Mus musculus OX=10090 GN=Srsf4 PE=2 SV=1 | Srsf4 | 0.59 |
| P11680 | Properdin OS=Mus musculus OX=10090 GN=Cfp PE=1 SV=2 | Cfp | 0.59 |
| Q9D7M8 | DNA-directed RNA polymerase II subunit RPB4 OS=Mus musculus OX=10090 GN=Polr2d PE=1 SV=2 | Polr2d | 0.60 |
| Q6A065 | Centrosomal protein of 170 kDa OS=Mus musculus OX=10090 GN=Cep170 PE=1 SV=2 | Cep170 | 0.60 |
| Q9Z2L6 | Multiple inositol polyphosphate phosphatase 1 OS=Mus musculus OX=10090 GN=Minpp1 PE=1 SV=3 | Minpp1 | 0.60 |
| Q3TVI8 | Pre-B-cell leukemia transcription factor-interacting protein 1 OS=Mus musculus OX=10090 GN=Pbxip1 PE=1 SV=2 | Pbxip1 | 0.60 |
| Q8VE38 | Oxidoreductase NAD-binding domain-containing protein 1 OS=Mus musculus OX=10090 GN=Oxnad1 PE=1 SV=2 | Oxnad1 | 0.60 |
| A2A8U2 | Transmembrane protein 201 OS=Mus musculus OX=10090 GN=Tmem201 PE=1 SV=1 | Tmem201 | 0.60 |
| Q9CQS2 | H/ACA ribonucleoprotein complex subunit 3 OS=Mus musculus OX=10090 GN=Nop10 PE=3 SV=1 | Nop10 | 0.60 |
| Q9DBT3 | Coiled-coil domain-containing protein 97 OS=Mus musculus OX=10090 GN=Ccdc97 PE=1 SV=1 | Ccdc97 | 0.60 |
| Q8BSZ2 | AP-3 complex subunit sigma-2 OS=Mus musculus OX=10090 GN=Ap3s2 PE=1 SV=1 | Ap3s2 | 0.60 |
| P39098 | Mannosyl-oligosaccharide 1,2-alpha-mannosidase IB OS=Mus musculus OX=10090 GN=Man1a2 PE=1 SV=1 | Man1a2 | 0.60 |
| P56379 | ATP synthase subunit ATP5MJ, mitochondrial OS=Mus musculus OX=10090 GN=Atp5mj PE=1 SV=1 | Atp5mj | 0.60 |
| Q9EP72 | ER membrane protein complex subunit 7 OS=Mus musculus OX=10090 GN=Emc7 PE=1 SV=1 | Emc7 | 0.60 |
| Q64430 | Copper-transporting ATPase 1 OS=Mus musculus OX=10090 GN=Atp7a PE=1 SV=3 | Atp7a | 0.60 |
| Q810A3 | Tetratricopeptide repeat protein 9C OS=Mus musculus OX=10090 GN=Ttc9c PE=1 SV=1 | Ttc9c | 0.60 |
| Q9D6J3 | Splicing factor YJU2 OS=Mus musculus OX=10090 GN=Yju2 PE=1 SV=1 | Yju2 | 0.60 |
| P62309 | Small nuclear ribonucleoprotein G OS=Mus musculus OX=10090 GN=Snrpg PE=1 SV=1 | Snrpg | 0.60 |
| Q8CFD4 | Sorting nexin-8 OS=Mus musculus OX=10090 GN=Snx8 PE=1 SV=1 | Snx8 | 0.61 |
| P61219 | DNA-directed RNA polymerases I, II, and III subunit RPABC2 OS=Mus musculus OX=10090 GN=Polr2f PE=2 SV=1 | Polr2f | 0.61 |
| Q8CHI8 | E1A-binding protein p400 OS=Mus musculus OX=10090 GN=Ep400 PE=1 SV=3 | Ep400 | 0.61 |
| Q4PJX1 | Protein odr-4 homolog OS=Mus musculus OX=10090 GN=Odr4 PE=1 SV=2 | Odr4 | 0.61 |
| P30415 | NK-tumor recognition protein OS=Mus musculus OX=10090 GN=Nktr PE=1 SV=4 | Nktr | 0.61 |
| G5E8P1 | Bromodomain-containing protein 1 OS=Mus musculus OX=10090 GN=Brd1 PE=1 SV=1 | Brd1 | 0.61 |
| Q9D9Z1 | Small kinetochore-associated protein OS=Mus musculus OX=10090 GN=Knstrn PE=2 SV=2 | Knstrn | 0.61 |
| O88531 | Palmitoyl-protein thioesterase 1 OS=Mus musculus OX=10090 GN=Ppt1 PE=1 SV=2 | Ppt1 | 0.61 |
| Q9DCC4 | Pyrroline-5-carboxylate reductase 3 OS=Mus musculus OX=10090 GN=Pycr3 PE=1 SV=2 | Pycr3 | 0.61 |
| O09005 | Sphingolipid delta(4)-desaturase DES1 OS=Mus musculus OX=10090 GN=Degs1 PE=1 SV=1 | Degs1 | 0.61 |
| P63166 | Small ubiquitin-related modifier 1 OS=Mus musculus OX=10090 GN=Sumo1 PE=1 SV=1 | Sumo1 | 0.61 |
| P35282 | Ras-related protein Rab-21 OS=Mus musculus OX=10090 GN=Rab21 PE=1 SV=4 | Rab21 | 0.61 |
| Q3UBX0 | Transmembrane protein 109 OS=Mus musculus OX=10090 GN=Tmem109 PE=1 SV=2 | Tmem109 | 0.61 |
| Q9CYD3 | Cartilage-associated protein OS=Mus musculus OX=10090 GN=Crtap PE=1 SV=3 | Crtap | 0.62 |
| Q497V5 | S1 RNA-binding domain-containing protein 1 OS=Mus musculus OX=10090 GN=Srbd1 PE=2 SV=3 | Srbd1 | 0.62 |
| Q4FK66 | Pre-mRNA-splicing factor 38A OS=Mus musculus OX=10090 GN=Prpf38a PE=1 SV=1 | Prpf38a | 0.62 |
| Q9CX30 | Protein YIF1B OS=Mus musculus OX=10090 GN=Yif1b PE=1 SV=2 | Yif1b | 0.62 |
| P63024 | Vesicle-associated membrane protein 3 OS=Mus musculus OX=10090 GN=Vamp3 PE=1 SV=1 | Vamp3 | 0.62 |
| Q9CQP3 | Coiled-coil-helix-coiled-coil-helix domain-containing protein 5 OS=Mus musculus OX=10090 GN=Chchd5 PE=1 SV=1 | Chchd5 | 0.62 |
| Q80T69 | Lysine-specific demethylase 9 OS=Mus musculus OX=10090 GN=Rsbn1 PE=1 SV=3 | Rsbn1 | 0.62 |
| Q9JIZ9 | Phospholipid scramblase 3 OS=Mus musculus OX=10090 GN=Plscr3 PE=1 SV=1 | Plscr3 | 0.62 |
| Q9CPY3 | Sororin OS=Mus musculus OX=10090 GN=Cdca5 PE=1 SV=1 | Cdca5 | 0.62 |
| Q5NBY9 | POZ (BTB) and AT hook-containing zinc finger 1 OS=Mus musculus OX=10090 GN=Patz1 PE=1 SV=1 | Patz1 | 0.62 |
| Q6PGG6 | Guanine nucleotide-binding protein-like 3-like protein OS=Mus musculus OX=10090 GN=Gnl3l PE=1 SV=1 | Gnl3l | 0.63 |
| Q3UHX9 | Putative methyltransferase C9orf114 homolog OS=Mus musculus OX=10090 GN=Spout1 PE=1 SV=1 | Spout1 | 0.63 |
| P58404 | Striatin-4 OS=Mus musculus OX=10090 GN=Strn4 PE=1 SV=2 | Strn4 | 0.63 |
| Q7TQK4 | Exosome complex component RRP40 OS=Mus musculus OX=10090 GN=Exosc3 PE=1 SV=3 | Exosc3 | 0.63 |
| O35680 | 28S ribosomal protein S12, mitochondrial OS=Mus musculus OX=10090 GN=Mrps12 PE=2 SV=1 | Mrps12 | 0.63 |
| P05132 | cAMP-dependent protein kinase catalytic subunit alpha OS=Mus musculus OX=10090 GN=Prkaca PE=1 SV=3 | Prkaca | 0.63 |
| Q571G4 | Protein lin-54 homolog OS=Mus musculus OX=10090 GN=Lin54 PE=2 SV=2 | Lin54 | 0.63 |
| Q62087 | Serum paraoxonase/lactonase 3 OS=Mus musculus OX=10090 GN=Pon3 PE=1 SV=2 | Pon3 | 0.63 |
| Q9CQ75 | NADH dehydrogenase [ubiquinone] 1 alpha subcomplex subunit 2 OS=Mus musculus OX=10090 GN=Ndufa2 PE=1 SV=3 | Ndufa2 | 0.63 |
| Q78WZ7 | DNA-directed RNA polymerase I subunit RPA43 OS=Mus musculus OX=10090 GN=Polr1f PE=1 SV=1 | Polr1f | 0.63 |
| Q8BLF1 | Neutral cholesterol ester hydrolase 1 OS=Mus musculus OX=10090 GN=Nceh1 PE=1 SV=1 | Nceh1 | 0.63 |
| P57746 | V-type proton ATPase subunit D OS=Mus musculus OX=10090 GN=Atp6v1d PE=1 SV=1 | Atp6v1d | 0.63 |
| Q76N33 | AMSH-like protease OS=Mus musculus OX=10090 GN=Stambpl1 PE=1 SV=1 | Stambpl1 | 0.63 |
| P47740 | Aldehyde dehydrogenase family 3 member A2 OS=Mus musculus OX=10090 GN=Aldh3a2 PE=1 SV=2 | Aldh3a2 | 0.63 |
| Q9Z1S0 | Mitotic checkpoint serine/threonine-protein kinase BUB1 beta OS=Mus musculus OX=10090 GN=Bub1b PE=1 SV=2 | Bub1b | 0.63 |
| Q64701 | Retinoblastoma-like protein 1 OS=Mus musculus OX=10090 GN=Rbl1 PE=1 SV=3 | Rbl1 | 0.63 |
| Q9Z2G6 | Protein sel-1 homolog 1 OS=Mus musculus OX=10090 GN=Sel1l PE=1 SV=2 | Sel1l | 0.64 |
| O55106 | Striatin OS=Mus musculus OX=10090 GN=Strn PE=1 SV=2 | Strn | 0.64 |
| Q8CBY0 | Glutamyl-tRNA(Gln) amidotransferase subunit C, mitochondrial OS=Mus musculus OX=10090 GN=Gatc PE=1 SV=1 | Gatc | 0.64 |
| Q9Z127 | Large neutral amino acids transporter small subunit 1 OS=Mus musculus OX=10090 GN=Slc7a5 PE=1 SV=2 | Slc7a5 | 0.64 |
| Q61136 | Serine/threonine-protein kinase PRP4 homolog OS=Mus musculus OX=10090 GN=Prpf4b PE=1 SV=3 | Prpf4b | 0.64 |
| Q9DAA6 | Exosome complex component CSL4 OS=Mus musculus OX=10090 GN=Exosc1 PE=1 SV=1 | Exosc1 | 0.64 |
| Q08122 | Transducin-like enhancer protein 3 OS=Mus musculus OX=10090 GN=Tle3 PE=1 SV=3 | Tle3 | 0.64 |
| Q61462 | Cytochrome b-245 light chain OS=Mus musculus OX=10090 GN=Cyba PE=1 SV=3 | Cyba | 0.64 |
| Q3V0C5 | Ubiquitin carboxyl-terminal hydrolase 48 OS=Mus musculus OX=10090 GN=Usp48 PE=1 SV=2 | Usp48 | 0.64 |
| Q5NCR9 | Nuclear speckle splicing regulatory protein 1 OS=Mus musculus OX=10090 GN=Nsrp1 PE=1 SV=1 | Nsrp1 | 0.64 |
| B1AUE5 | Peroxisome biogenesis factor 10 OS=Mus musculus OX=10090 GN=Pex10 PE=2 SV=1 | Pex10 | 0.64 |
| Q9D720 | Non-structural maintenance of chromosomes element 1 homolog OS=Mus musculus OX=10090 GN=Nsmce1 PE=1 SV=1 | Nsmce1 | 0.64 |
| Q8BHN5 | RNA-binding protein 45 OS=Mus musculus OX=10090 GN=Rbm45 PE=1 SV=1 | Rbm45 | 0.64 |
| Q5RJH6 | Protein SMG7 OS=Mus musculus OX=10090 GN=Smg7 PE=2 SV=1 | Smg7 | 0.65 |
| Q69ZN6 | N-acetylglucosamine-1-phosphotransferase subunits alpha/beta OS=Mus musculus OX=10090 GN=Gnptab PE=2 SV=2 | Gnptab | 0.65 |
| D3YZP9 | Coiled-coil domain-containing protein 6 OS=Mus musculus OX=10090 GN=Ccdc6 PE=1 SV=1 | Ccdc6 | 0.65 |
| P28574 | Protein max OS=Mus musculus OX=10090 GN=Max PE=1 SV=2 | Max | 0.65 |
| Q9CQH8 | Ribonuclease P protein subunit p14 OS=Mus musculus OX=10090 GN=Rpp14 PE=1 SV=1 | Rpp14 | 0.65 |
| Q61072 | Disintegrin and metalloproteinase domain-containing protein 9 OS=Mus musculus OX=10090 GN=Adam9 PE=1 SV=2 | Adam9 | 0.65 |
| P21279 | Guanine nucleotide-binding protein G(q) subunit alpha OS=Mus musculus OX=10090 GN=Gnaq PE=1 SV=4 | Gnaq | 0.65 |
| Q8BM55 | Transmembrane protein 214 OS=Mus musculus OX=10090 GN=Tmem214 PE=1 SV=1 | Tmem214 | 0.65 |
| Q8BUY9 | Geranylgeranyl transferase type-1 subunit beta OS=Mus musculus OX=10090 GN=Pggt1b PE=1 SV=1 | Pggt1b | 0.65 |
| Q3KNM2 | E3 ubiquitin-protein ligase MARCHF5 OS=Mus musculus OX=10090 GN=Marchf5 PE=1 SV=1 | Marchf5 | 0.65 |
| O08539 | Myc box-dependent-interacting protein 1 OS=Mus musculus OX=10090 GN=Bin1 PE=1 SV=1 | Bin1 | 0.65 |
| O70472 | Transmembrane protein 131 OS=Mus musculus OX=10090 GN=Tmem131 PE=2 SV=2 | Tmem131 | 0.65 |
| P97480 | Eyes absent homolog 3 OS=Mus musculus OX=10090 GN=Eya3 PE=1 SV=2 | Eya3 | 0.65 |
| Q91VN4 | MICOS complex subunit Mic25 OS=Mus musculus OX=10090 GN=Chchd6 PE=1 SV=2 | Chchd6 | 0.65 |
| Q8BT07 | Centrosomal protein of 55 kDa OS=Mus musculus OX=10090 GN=Cep55 PE=1 SV=2 | Cep55 | 0.65 |
| Q68ED3 | Terminal nucleotidyltransferase 4B OS=Mus musculus OX=10090 GN=Tent4b PE=1 SV=2 | Tent4b | 0.65 |
| Q8CHV6 | Transcriptional adapter 2-alpha OS=Mus musculus OX=10090 GN=Tada2a PE=1 SV=1 | Tada2a | 0.65 |
| Q32NY4 | Metal transporter CNNM3 OS=Mus musculus OX=10090 GN=Cnnm3 PE=1 SV=2 | Cnnm3 | 0.65 |
| Q80UX8 | Protein ABHD13 OS=Mus musculus OX=10090 GN=Abhd13 PE=2 SV=1 | Abhd13 | 0.65 |
| P23198 | Chromobox protein homolog 3 OS=Mus musculus OX=10090 GN=Cbx3 PE=1 SV=2 | Cbx3 | 0.65 |
| Q91WS0 | CDGSH iron-sulfur domain-containing protein 1 OS=Mus musculus OX=10090 GN=Cisd1 PE=1 SV=1 | Cisd1 | 0.65 |
| P59481 | VIP36-like protein OS=Mus musculus OX=10090 GN=Lman2l PE=1 SV=1 | Lman2l | 0.65 |
| Q8BTE0 | Succinate dehydrogenase assembly factor 4, mitochondrial OS=Mus musculus OX=10090 GN=Sdhaf4 PE=3 SV=2 | Sdhaf4 | 0.65 |
| O55100 | Synaptogyrin-1 OS=Mus musculus OX=10090 GN=Syngr1 PE=1 SV=2 | Syngr1 | 0.65 |
| Q60775 | ETS-related transcription factor Elf-1 OS=Mus musculus OX=10090 GN=Elf1 PE=1 SV=1 | Elf1 | 0.65 |
| Q80SZ7 | Guanine nucleotide-binding protein G(I)/G(S)/G(O) subunit gamma-5 OS=Mus musculus OX=10090 GN=Gng5 PE=1 SV=2 | Gng5 | 0.66 |
| Q6PDH4 | Ran-binding protein 3-like OS=Mus musculus OX=10090 GN=Ranbp3l PE=1 SV=1 | Ranbp3l | 0.66 |
| Q8BHC9 | Alpha-(1,3)-fucosyltransferase 11 OS=Mus musculus OX=10090 GN=Fut11 PE=1 SV=1 | Fut11 | 0.66 |
| Q920D3 | Mediator of RNA polymerase II transcription subunit 28 OS=Mus musculus OX=10090 GN=Med28 PE=1 SV=2 | Med28 | 0.66 |
| Q3THW5 | Histone H2A.V OS=Mus musculus OX=10090 GN=H2az2 PE=1 SV=3 | H2az2 | 0.66 |
| Q8K2X3 | CST complex subunit STN1 OS=Mus musculus OX=10090 GN=Stn1 PE=1 SV=2 | Stn1 | 0.66 |
| Q9WUD8 | Fas apoptotic inhibitory molecule 1 OS=Mus musculus OX=10090 GN=Faim PE=1 SV=1 | Faim | 0.66 |
| Q8K2Y0 | ORC ubiquitin ligase 1 OS=Mus musculus OX=10090 GN=Obi1 PE=1 SV=2 | Obi1 | 0.66 |
| Q8K2D6 | Deoxycytidylate deaminase OS=Mus musculus OX=10090 GN=Dctd PE=2 SV=1 | Dctd | 0.66 |
| Q60967 | Bifunctional 3'-phosphoadenosine 5'-phosphosulfate synthase 1 OS=Mus musculus OX=10090 GN=Papss1 PE=1 SV=1 | Papss1 | 0.66 |
| Q3ULF4 | Paraplegin OS=Mus musculus OX=10090 GN=Spg7 PE=1 SV=1 | Spg7 | 0.66 |
| Q9JJF9 | Signal peptide peptidase-like 2A OS=Mus musculus OX=10090 GN=Sppl2a PE=1 SV=2 | Sppl2a | 0.66 |
| Q8BHX1 | HAUS augmin-like complex subunit 1 OS=Mus musculus OX=10090 GN=Haus1 PE=1 SV=2 | Haus1 | 0.66 |
| Q9CQA6 | Coiled-coil-helix-coiled-coil-helix domain-containing protein 1 OS=Mus musculus OX=10090 GN=Chchd1 PE=1 SV=1 | Chchd1 | 0.66 |
| Q9D1B9 | 39S ribosomal protein L28, mitochondrial OS=Mus musculus OX=10090 GN=Mrpl28 PE=1 SV=3 | Mrpl28 | 0.66 |
| Q9Z0Y1 | Dynactin subunit 3 OS=Mus musculus OX=10090 GN=Dctn3 PE=1 SV=2 | Dctn3 | 0.66 |
| Q922Q4 | Pyrroline-5-carboxylate reductase 2 OS=Mus musculus OX=10090 GN=Pycr2 PE=1 SV=1 | Pycr2 | 0.66 |
| Q9D1H6 | NADH dehydrogenase [ubiquinone] 1 alpha subcomplex assembly factor 4 OS=Mus musculus OX=10090 GN=Ndufaf4 PE=1 SV=1 | Ndufaf4 | 0.66 |
| Q8BX90 | Fibronectin type-III domain-containing protein 3A OS=Mus musculus OX=10090 GN=Fndc3a PE=1 SV=3 | Fndc3a | 0.66 |
| Q8R4X3 | RNA-binding protein 12 OS=Mus musculus OX=10090 GN=Rbm12 PE=1 SV=3 | Rbm12 | 0.66 |
| Q9R0L7 | A-kinase anchor protein 8-like OS=Mus musculus OX=10090 GN=Akap8l PE=1 SV=1 | Akap8l | 0.66 |
| Q6ZPZ3 | Zinc finger CCCH domain-containing protein 4 OS=Mus musculus OX=10090 GN=Zc3h4 PE=1 SV=2 | Zc3h4 | 0.66 |
| P97384 | Annexin A11 OS=Mus musculus OX=10090 GN=Anxa11 PE=1 SV=2 | Anxa11 | 0.66 |
| Q8R0S2 | IQ motif and SEC7 domain-containing protein 1 OS=Mus musculus OX=10090 GN=Iqsec1 PE=1 SV=2 | Iqsec1 | 0.66 |
| Q9Z1B5 | Mitotic spindle assembly checkpoint protein MAD2A OS=Mus musculus OX=10090 GN=Mad2l1 PE=1 SV=2 | Mad2l1 | 0.66 |
| Q8BYU6 | Torsin-1A-interacting protein 2 OS=Mus musculus OX=10090 GN=Tor1aip2 PE=1 SV=1 | Tor1aip2 | 0.66 |
| Q80X85 | 28S ribosomal protein S7, mitochondrial OS=Mus musculus OX=10090 GN=Mrps7 PE=1 SV=1 | Mrps7 | 0.66 |
| P52623 | Uridine-cytidine kinase 1 OS=Mus musculus OX=10090 GN=Uck1 PE=1 SV=2 | Uck1 | 0.67 |
| P30276 | G2/mitotic-specific cyclin-B2 OS=Mus musculus OX=10090 GN=Ccnb2 PE=1 SV=2 | Ccnb2 | 0.67 |
| Q571H0 | Nucleolar pre-ribosomal-associated protein 1 OS=Mus musculus OX=10090 GN=Urb1 PE=1 SV=2 | Urb1 | 0.67 |
| Q9JL62 | Glycolipid transfer protein OS=Mus musculus OX=10090 GN=Gltp PE=1 SV=3 | Gltp | 0.67 |
| Q9Z0H4 | CUGBP Elav-like family member 2 OS=Mus musculus OX=10090 GN=Celf2 PE=1 SV=1 | Celf2 | 0.67 |
| Q8R404 | MICOS complex subunit MIC13 OS=Mus musculus OX=10090 GN=Micos13 PE=1 SV=1 | Micos13 | 0.67 |
| Q9CQ26 | STAM-binding protein OS=Mus musculus OX=10090 GN=Stambp PE=1 SV=1 | Stambp | 0.67 |
| Q9CZJ1 | Probable U3 small nucleolar RNA-associated protein 11 OS=Mus musculus OX=10090 GN=Utp11 PE=2 SV=1 | Utp11 | 0.67 |
| P03966 | N-myc proto-oncogene protein OS=Mus musculus OX=10090 GN=Mycn PE=2 SV=2 | Mycn | 0.67 |
| Q9WUZ9 | Ectonucleoside triphosphate diphosphohydrolase 5 OS=Mus musculus OX=10090 GN=Entpd5 PE=1 SV=1 | Entpd5 | 0.67 |
| Q61084 | Mitogen-activated protein kinase kinase kinase 3 OS=Mus musculus OX=10090 GN=Map3k3 PE=1 SV=1 | Map3k3 | 0.67 |
| Q6ZQA0 | Neurobeachin-like protein 2 OS=Mus musculus OX=10090 GN=Nbeal2 PE=1 SV=2 | Nbeal2 | 0.67 |
| Q6PR54 | Telomere-associated protein RIF1 OS=Mus musculus OX=10090 GN=Rif1 PE=1 SV=2 | Rif1 | 0.67 |
| P29595 | NEDD8 OS=Mus musculus OX=10090 GN=Nedd8 PE=1 SV=2 | Nedd8 | 0.67 |
| E9Q9D5 | Rab-like protein 2A OS=Mus musculus OX=10090 GN=Rabl2 PE=1 SV=1 | Rabl2 | 0.67 |
| Q8VCX5 | Calcium uptake protein 1, mitochondrial OS=Mus musculus OX=10090 GN=Micu1 PE=1 SV=1 | Micu1 | 0.67 |
| Q80WE4 | Kinesin-like protein KIF20B OS=Mus musculus OX=10090 GN=Kif20b PE=1 SV=3 | Kif20b | 0.67 |
| Q924Z6 | Exportin-6 OS=Mus musculus OX=10090 GN=Xpo6 PE=1 SV=2 | Xpo6 | 0.68 |
| Q5XG73 | Acyl-CoA-binding domain-containing protein 5 OS=Mus musculus OX=10090 GN=Acbd5 PE=1 SV=1 | Acbd5 | 0.68 |
| O70591 | Prefoldin subunit 2 OS=Mus musculus OX=10090 GN=Pfdn2 PE=1 SV=2 | Pfdn2 | 0.68 |
| Q5Y5T1 | Palmitoyltransferase ZDHHC20 OS=Mus musculus OX=10090 GN=Zdhhc20 PE=1 SV=1 | Zdhhc20 | 0.68 |
| Q8BKX6 | Serine/threonine-protein kinase SMG1 OS=Mus musculus OX=10090 GN=Smg1 PE=1 SV=3 | Smg1 | 0.68 |
| Q3UFY7 | 7-methylguanosine phosphate-specific 5'-nucleotidase OS=Mus musculus OX=10090 GN=Nt5c3b PE=1 SV=3 | Nt5c3b | 0.68 |
| P10711 | Transcription elongation factor A protein 1 OS=Mus musculus OX=10090 GN=Tcea1 PE=1 SV=2 | Tcea1 | 0.68 |
| P23708 | Nuclear transcription factor Y subunit alpha OS=Mus musculus OX=10090 GN=Nfya PE=1 SV=2 | Nfya | 0.68 |
| Q91WK1 | SPRY domain-containing protein 4 OS=Mus musculus OX=10090 GN=Spryd4 PE=1 SV=1 | Spryd4 | 0.68 |
| Q99KS2 | Neugrin OS=Mus musculus OX=10090 GN=Ngrn PE=2 SV=3 | Ngrn | 0.68 |
| P62245 | 40S ribosomal protein S15a OS=Mus musculus OX=10090 GN=Rps15a PE=1 SV=2 | Rps15a | 0.68 |
| Q6P3D0 | U8 snoRNA-decapping enzyme OS=Mus musculus OX=10090 GN=Nudt16 PE=1 SV=1 | Nudt16 | 0.68 |
| Q78PG9 | Coiled-coil domain-containing protein 25 OS=Mus musculus OX=10090 GN=Ccdc25 PE=1 SV=1 | Ccdc25 | 0.68 |
| Q9QZW0 | Phospholipid-transporting ATPase 11C OS=Mus musculus OX=10090 GN=Atp11c PE=1 SV=2 | Atp11c | 0.68 |
| Q8K4Z3 | NAD(P)H-hydrate epimerase OS=Mus musculus OX=10090 GN=Naxe PE=1 SV=1 | Naxe | 0.68 |
| Q5NC05 | Transcription termination factor 2 OS=Mus musculus OX=10090 GN=Ttf2 PE=1 SV=2 | Ttf2 | 0.68 |
| Q921L3 | Calcium load-activated calcium channel OS=Mus musculus OX=10090 GN=Tmco1 PE=1 SV=1 | Tmco1 | 0.68 |
| Q14BV6 | Carbohydrate deacetylase OS=Mus musculus OX=10090 GN=Ydjc PE=2 SV=2 | Ydjc | 0.68 |
| Q8K215 | LYR motif-containing protein 4 OS=Mus musculus OX=10090 GN=Lyrm4 PE=1 SV=1 | Lyrm4 | 0.68 |
| Q8R480 | Nuclear pore complex protein Nup85 OS=Mus musculus OX=10090 GN=Nup85 PE=1 SV=1 | Nup85 | 0.68 |
| Q8R0S1 | Cyclic AMP-dependent transcription factor ATF-7 OS=Mus musculus OX=10090 GN=Atf7 PE=1 SV=1 | Atf7 | 0.68 |
| Q924M7 | Mannose-6-phosphate isomerase OS=Mus musculus OX=10090 GN=Mpi PE=1 SV=1 | Mpi | 0.68 |
| Q91VM9 | Inorganic pyrophosphatase 2, mitochondrial OS=Mus musculus OX=10090 GN=Ppa2 PE=1 SV=1 | Ppa2 | 0.68 |
| O54916 | RalBP1-associated Eps domain-containing protein 1 OS=Mus musculus OX=10090 GN=Reps1 PE=1 SV=2 | Reps1 | 0.69 |
| Q99NF3 | Centrosomal protein of 41 kDa OS=Mus musculus OX=10090 GN=Cep41 PE=1 SV=1 | Cep41 | 0.69 |
| Q9CQB5 | CDGSH iron-sulfur domain-containing protein 2 OS=Mus musculus OX=10090 GN=Cisd2 PE=1 SV=1 | Cisd2 | 0.69 |
| P49025 | Citron Rho-interacting kinase OS=Mus musculus OX=10090 GN=Cit PE=1 SV=3 | Cit | 0.69 |
| P60824 | Cold-inducible RNA-binding protein OS=Mus musculus OX=10090 GN=Cirbp PE=1 SV=1 | Cirbp | 0.69 |
| Q5I012 | Putative sodium-coupled neutral amino acid transporter 10 OS=Mus musculus OX=10090 GN=Slc38a10 PE=1 SV=2 | Slc38a10 | 0.69 |
| Q64127 | Transcription intermediary factor 1-alpha OS=Mus musculus OX=10090 GN=Trim24 PE=1 SV=1 | Trim24 | 0.69 |
| Q8CHP8 | Glycerol-3-phosphate phosphatase OS=Mus musculus OX=10090 GN=Pgp PE=1 SV=1 | Pgp | 0.69 |
| Q8R2S9 | Actin-related protein 8 OS=Mus musculus OX=10090 GN=Actr8 PE=2 SV=1 | Actr8 | 0.69 |
| Q8BU11 | TOX high mobility group box family member 4 OS=Mus musculus OX=10090 GN=Tox4 PE=1 SV=3 | Tox4 | 0.69 |
| Q91YM4 | FAST kinase domain-containing protein 4 OS=Mus musculus OX=10090 GN=Tbrg4 PE=1 SV=1 | Tbrg4 | 0.69 |
| Q9Z222 | N-acetyllactosaminide beta-1,3-N-acetylglucosaminyltransferase 2 OS=Mus musculus OX=10090 GN=B3GNT2 PE=1 SV=3 | B3GNT2 | 0.69 |
| Q3UMB9 | WASH complex subunit 4 OS=Mus musculus OX=10090 GN=Washc4 PE=1 SV=2 | Washc4 | 0.69 |
| Q8BVL3 | Sorting nexin-17 OS=Mus musculus OX=10090 GN=Snx17 PE=1 SV=2 | Snx17 | 0.69 |
| Q9D2C6 | DNA-directed RNA polymerase III subunit RPC8 OS=Mus musculus OX=10090 GN=Polr3h PE=1 SV=2 | Polr3h | 0.69 |
| Q8CG46 | Structural maintenance of chromosomes protein 5 OS=Mus musculus OX=10090 GN=Smc5 PE=1 SV=1 | Smc5 | 0.69 |
| Q99JW4 | LIM and senescent cell antigen-like-containing domain protein 1 OS=Mus musculus OX=10090 GN=Lims1 PE=1 SV=3 | Lims1 | 0.69 |
| Q9R0P6 | Signal peptidase complex catalytic subunit SEC11A OS=Mus musculus OX=10090 GN=Sec11a PE=1 SV=1 | Sec11a | 0.69 |
| P18155 | Bifunctional methylenetetrahydrofolate dehydrogenase/cyclohydrolase, mitochondrial OS=Mus musculus OX=10090 GN=Mthfd2 PE=1 SV=1 | Mthfd2 | 0.69 |
| P63213 | Guanine nucleotide-binding protein G(I)/G(S)/G(O) subunit gamma-2 OS=Mus musculus OX=10090 GN=Gng2 PE=1 SV=2 | Gng2 | 0.69 |
| Q8C5L3 | CCR4-NOT transcription complex subunit 2 OS=Mus musculus OX=10090 GN=Cnot2 PE=1 SV=2 | Cnot2 | 0.69 |
| Q9CQ45 | Neudesin OS=Mus musculus OX=10090 GN=Nenf PE=1 SV=1 | Nenf | 0.69 |
| Q8R3N6 | THO complex subunit 1 OS=Mus musculus OX=10090 GN=Thoc1 PE=1 SV=1 | Thoc1 | 0.69 |
| Q9ESE1 | Lipopolysaccharide-responsive and beige-like anchor protein OS=Mus musculus OX=10090 GN=Lrba PE=1 SV=1 | Lrba | 0.69 |
| Q9CR08 | Ribonuclease P protein subunit p29 OS=Mus musculus OX=10090 GN=Pop4 PE=2 SV=1 | Pop4 | 0.69 |
| Q8BHE8 | m-AAA protease-interacting protein 1, mitochondrial OS=Mus musculus OX=10090 GN=Maip1 PE=1 SV=1 | Maip1 | 0.69 |
| Q8CCP0 | Nuclear export mediator factor Nemf OS=Mus musculus OX=10090 GN=Nemf PE=1 SV=2 | Nemf | 0.69 |
| P35991 | Tyrosine-protein kinase BTK OS=Mus musculus OX=10090 GN=Btk PE=1 SV=4 | Btk | 0.69 |
| Q9DB15 | 39S ribosomal protein L12, mitochondrial OS=Mus musculus OX=10090 GN=Mrpl12 PE=1 SV=2 | Mrpl12 | 0.69 |
| Q91YE7 | RNA-binding protein 5 OS=Mus musculus OX=10090 GN=Rbm5 PE=1 SV=1 | Rbm5 | 0.70 |
| Q8K057 | Intraflagellar transport protein 80 homolog OS=Mus musculus OX=10090 GN=Ift80 PE=1 SV=1 | Ift80 | 0.70 |
| Q80XC2 | tRNA (adenine(58)-N(1))-methyltransferase catalytic subunit TRMT61A OS=Mus musculus OX=10090 GN=Trmt61a PE=1 SV=1 | Trmt61a | 0.70 |
| E9Q137 | Testis-expressed protein 264 homolog OS=Mus musculus OX=10090 GN=Tex264 PE=2 SV=1 | Tex264 | 0.70 |
| Q9CY66 | H/ACA ribonucleoprotein complex subunit 1 OS=Mus musculus OX=10090 GN=Gar1 PE=1 SV=1 | Gar1 | 0.70 |
| Q5XJY4 | Presenilins-associated rhomboid-like protein, mitochondrial OS=Mus musculus OX=10090 GN=Parl PE=1 SV=1 | Parl | 0.70 |
| Q9CQV6 | Microtubule-associated proteins 1A/1B light chain 3B OS=Mus musculus OX=10090 GN=Map1lc3b PE=1 SV=3 | Map1lc3b | 0.70 |
| Q9Z1E3 | NF-kappa-B inhibitor alpha OS=Mus musculus OX=10090 GN=Nfkbia PE=1 SV=2 | Nfkbia | 0.70 |
| P0DMN7 | S-adenosylmethionine decarboxylase proenzyme 1 OS=Mus musculus OX=10090 GN=Amd1 PE=1 SV=1 | Amd1 | 0.70 |
| Q9JI44 | DNA methyltransferase 1-associated protein 1 OS=Mus musculus OX=10090 GN=Dmap1 PE=1 SV=1 | Dmap1 | 0.70 |
| Q3UJU9 | Regulator of microtubule dynamics protein 3 OS=Mus musculus OX=10090 GN=Rmdn3 PE=1 SV=2 | Rmdn3 | 0.70 |
| O70481 | E3 ubiquitin-protein ligase UBR1 OS=Mus musculus OX=10090 GN=Ubr1 PE=1 SV=2 | Ubr1 | 0.70 |
| Q9QXG4 | Acetyl-coenzyme A synthetase, cytoplasmic OS=Mus musculus OX=10090 GN=Acss2 PE=1 SV=2 | Acss2 | 0.70 |
| P11370 | Retrovirus-related Env polyprotein from Fv-4 locus OS=Mus musculus OX=10090 GN=Fv4 PE=1 SV=2 | Fv4 | 0.70 |
| Q9CTH6 | rRNA-processing protein FCF1 homolog OS=Mus musculus OX=10090 GN=Fcf1 PE=2 SV=2 | Fcf1 | 0.70 |
| Q9CXV1 | Succinate dehydrogenase [ubiquinone] cytochrome b small subunit, mitochondrial OS=Mus musculus OX=10090 GN=Sdhd PE=1 SV=2 | Sdhd | 0.70 |
| Q9D6M3 | Mitochondrial glutamate carrier 1 OS=Mus musculus OX=10090 GN=Slc25a22 PE=1 SV=1 | Slc25a22 | 0.70 |
| P47964 | 60S ribosomal protein L36 OS=Mus musculus OX=10090 GN=Rpl36 PE=1 SV=2 | Rpl36 | 0.70 |
| Q921V5 | Alpha-1,6-mannosyl-glycoprotein 2-beta-N-acetylglucosaminyltransferase OS=Mus musculus OX=10090 GN=Mgat2 PE=1 SV=1 | Mgat2 | 0.70 |
| Q9JJR8 | Transmembrane protein 9B OS=Mus musculus OX=10090 GN=Tmem9b PE=1 SV=1 | Tmem9b | 0.70 |
| Q8VIM9 | Immunity-related GTPase family Q protein OS=Mus musculus OX=10090 GN=Irgq PE=1 SV=1 | Irgq | 0.70 |
| Q91WD5 | NADH dehydrogenase [ubiquinone] iron-sulfur protein 2, mitochondrial OS=Mus musculus OX=10090 GN=Ndufs2 PE=1 SV=1 | Ndufs2 | 0.70 |
| Q9EQM6 | Microprocessor complex subunit DGCR8 OS=Mus musculus OX=10090 GN=Dgcr8 PE=1 SV=2 | Dgcr8 | 0.70 |
| Q8K385 | Ferric-chelate reductase 1 OS=Mus musculus OX=10090 GN=FRRS1 PE=1 SV=1 | FRRS1 | 0.70 |
| Q99KW9 | T-cell immunomodulatory protein OS=Mus musculus OX=10090 GN=Itfg1 PE=1 SV=2 | Itfg1 | 0.70 |
| Q80WR5 | UPF0688 protein C1orf174 homolog OS=Mus musculus OX=10090 PE=1 SV=1 | -- | 0.70 |
| Q8BGF3 | Dynein axonemal assembly factor 10 OS=Mus musculus OX=10090 GN=Dnaaf10 PE=1 SV=1 | Dnaaf10 | 0.71 |
| P28704 | Retinoic acid receptor RXR-beta OS=Mus musculus OX=10090 GN=Rxrb PE=1 SV=2 | Rxrb | 0.71 |
| O88520 | Leucine-rich repeat protein SHOC-2 OS=Mus musculus OX=10090 GN=Shoc2 PE=1 SV=2 | Shoc2 | 0.71 |
| Q8R1K1 | Ubiquitin-associated domain-containing protein 2 OS=Mus musculus OX=10090 GN=Ubac2 PE=1 SV=1 | Ubac2 | 0.71 |
| P51174 | Long-chain specific acyl-CoA dehydrogenase, mitochondrial OS=Mus musculus OX=10090 GN=Acadl PE=1 SV=2 | Acadl | 0.71 |
| O35604 | NPC intracellular cholesterol transporter 1 OS=Mus musculus OX=10090 GN=Npc1 PE=1 SV=2 | Npc1 | 0.71 |
| O54790 | Transcription factor MafG OS=Mus musculus OX=10090 GN=Mafg PE=1 SV=1 | Mafg | 0.71 |
| Q8BYP3 | Rho-related GTP-binding protein RhoF OS=Mus musculus OX=10090 GN=Rhof PE=1 SV=1 | Rhof | 0.71 |
| Q8VDZ4 | Palmitoyltransferase ZDHHC5 OS=Mus musculus OX=10090 GN=Zdhhc5 PE=1 SV=1 | Zdhhc5 | 0.71 |
| P84104 | Serine/arginine-rich splicing factor 3 OS=Mus musculus OX=10090 GN=Srsf3 PE=1 SV=1 | Srsf3 | 0.71 |
| Q7TSQ8 | Pyruvate dehydrogenase phosphatase regulatory subunit, mitochondrial OS=Mus musculus OX=10090 GN=Pdpr PE=1 SV=1 | Pdpr | 0.71 |
| Q9JIM1 | Equilibrative nucleoside transporter 1 OS=Mus musculus OX=10090 GN=Slc29a1 PE=1 SV=3 | Slc29a1 | 0.71 |
| Q9QY93 | dCTP pyrophosphatase 1 OS=Mus musculus OX=10090 GN=Dctpp1 PE=1 SV=1 | Dctpp1 | 0.71 |
| Q80V62 | Fanconi anemia group D2 protein homolog OS=Mus musculus OX=10090 GN=Fancd2 PE=1 SV=2 | Fancd2 | 0.71 |
| Q8BVA5 | Lipid droplet-associated hydrolase OS=Mus musculus OX=10090 GN=Ldah PE=1 SV=1 | Ldah | 0.71 |
| Q8BJS4 | SUN domain-containing protein 2 OS=Mus musculus OX=10090 GN=Sun2 PE=1 SV=3 | Sun2 | 0.71 |
| P62315 | Small nuclear ribonucleoprotein Sm D1 OS=Mus musculus OX=10090 GN=Snrpd1 PE=1 SV=1 | Snrpd1 | 0.71 |
| Q6KCD5 | Nipped-B-like protein OS=Mus musculus OX=10090 GN=Nipbl PE=1 SV=1 | Nipbl | 0.71 |
| P49442 | Inositol polyphosphate 1-phosphatase OS=Mus musculus OX=10090 GN=Inpp1 PE=1 SV=2 | Inpp1 | 0.71 |
| Q8BGA9 | Mitochondrial inner membrane protein OXA1L OS=Mus musculus OX=10090 GN=Oxa1l PE=1 SV=1 | Oxa1l | 0.71 |
| O70274 | Protein tyrosine phosphatase type IVA 2 OS=Mus musculus OX=10090 GN=Ptp4a2 PE=1 SV=1 | Ptp4a2 | 0.71 |
| Q91XA2 | Golgi membrane protein 1 OS=Mus musculus OX=10090 GN=Golm1 PE=1 SV=2 | Golm1 | 0.72 |
| Q9JKL4 | NADH dehydrogenase [ubiquinone] 1 alpha subcomplex assembly factor 3 OS=Mus musculus OX=10090 GN=Ndufaf3 PE=1 SV=1 | Ndufaf3 | 0.72 |
| Q9EQS3 | c-Myc-binding protein OS=Mus musculus OX=10090 GN=Mycbp PE=1 SV=5 | Mycbp | 0.72 |
| P43276 | Histone H1.5 OS=Mus musculus OX=10090 GN=H1-5 PE=1 SV=2 | H1-5 | 0.72 |
| Q8VC65 | Nurim OS=Mus musculus OX=10090 GN=Nrm PE=1 SV=1 | Nrm | 0.72 |
| P47757 | F-actin-capping protein subunit beta OS=Mus musculus OX=10090 GN=Capzb PE=1 SV=3 | Capzb | 0.72 |
| Q9R020 | Zinc finger Ran-binding domain-containing protein 2 OS=Mus musculus OX=10090 GN=Zranb2 PE=1 SV=2 | Zranb2 | 0.72 |
| Q9CZW4 | Fatty acid CoA ligase Acsl3 OS=Mus musculus OX=10090 GN=Acsl3 PE=1 SV=2 | Acsl3 | 0.72 |
| Q06185 | ATP synthase subunit e, mitochondrial OS=Mus musculus OX=10090 GN=Atp5me PE=1 SV=2 | Atp5me | 0.72 |
| P84102 | Small EDRK-rich factor 2 OS=Mus musculus OX=10090 GN=Serf2 PE=1 SV=1 | Serf2 | 0.72 |
| Q9D8L5 | Coiled-coil domain-containing protein 91 OS=Mus musculus OX=10090 GN=Ccdc91 PE=1 SV=2 | Ccdc91 | 0.72 |
| Q99KG3 | RNA-binding protein 10 OS=Mus musculus OX=10090 GN=Rbm10 PE=1 SV=1 | Rbm10 | 0.72 |
| Q8BYW1 | Rho GTPase-activating protein 25 OS=Mus musculus OX=10090 GN=Arhgap25 PE=1 SV=2 | Arhgap25 | 0.72 |
| Q9D4C5 | ELL-associated factor 1 OS=Mus musculus OX=10090 GN=Eaf1 PE=1 SV=2 | Eaf1 | 0.72 |
| Q6P9R1 | ATP-dependent RNA helicase DDX51 OS=Mus musculus OX=10090 GN=Ddx51 PE=1 SV=1 | Ddx51 | 0.72 |
| Q6PFD9 | Nuclear pore complex protein Nup98-Nup96 OS=Mus musculus OX=10090 GN=Nup98 PE=1 SV=2 | Nup98 | 0.72 |
| O88545 | COP9 signalosome complex subunit 6 OS=Mus musculus OX=10090 GN=Cops6 PE=1 SV=1 | Cops6 | 0.72 |
| Q9CQD4 | Charged multivesicular body protein 1b-2 OS=Mus musculus OX=10090 GN=Chmp1b2 PE=2 SV=2 | Chmp1b2 | 0.72 |
| P70182 | Phosphatidylinositol 4-phosphate 5-kinase type-1 alpha OS=Mus musculus OX=10090 GN=Pip5k1a PE=1 SV=2 | Pip5k1a | 0.72 |
| Q8BWW4 | La-related protein 4 OS=Mus musculus OX=10090 GN=Larp4 PE=1 SV=2 | Larp4 | 0.72 |
| P70196 | TNF receptor-associated factor 6 OS=Mus musculus OX=10090 GN=Traf6 PE=1 SV=2 | Traf6 | 0.72 |
| O35657 | Sialidase-1 OS=Mus musculus OX=10090 GN=Neu1 PE=1 SV=1 | Neu1 | 0.72 |
| O35326 | Serine/arginine-rich splicing factor 5 OS=Mus musculus OX=10090 GN=Srsf5 PE=1 SV=2 | Srsf5 | 0.72 |
| Q8VHH5 | Arf-GAP with GTPase, ANK repeat and PH domain-containing protein 3 OS=Mus musculus OX=10090 GN=Agap3 PE=1 SV=1 | Agap3 | 0.72 |
| Q922M5 | Cell division cycle-associated 7-like protein OS=Mus musculus OX=10090 GN=Cdca7l PE=1 SV=1 | Cdca7l | 0.72 |
| P97393 | Rho GTPase-activating protein 5 OS=Mus musculus OX=10090 GN=Arhgap5 PE=1 SV=2 | Arhgap5 | 0.72 |
| Q8C1Y8 | Vacuolar fusion protein CCZ1 homolog OS=Mus musculus OX=10090 GN=Ccz1 PE=1 SV=1 | Ccz1 | 0.72 |
| O35943 | Frataxin, mitochondrial OS=Mus musculus OX=10090 GN=Fxn PE=1 SV=1 | Fxn | 0.72 |
| O70496 | H(+)/Cl(-) exchange transporter 7 OS=Mus musculus OX=10090 GN=Clcn7 PE=1 SV=1 | Clcn7 | 0.72 |
| Q9DAU1 | Protein canopy homolog 3 OS=Mus musculus OX=10090 GN=Cnpy3 PE=1 SV=1 | Cnpy3 | 0.72 |
| Q9Z1J3 | Cysteine desulfurase, mitochondrial OS=Mus musculus OX=10090 GN=Nfs1 PE=1 SV=3 | Nfs1 | 0.72 |
| Q3TLD5 | Unconventional prefoldin RPB5 interactor OS=Mus musculus OX=10090 GN=Uri1 PE=1 SV=2 | Uri1 | 0.72 |
| Q9CPQ3 | Mitochondrial import receptor subunit TOM22 homolog OS=Mus musculus OX=10090 GN=Tomm22 PE=1 SV=3 | Tomm22 | 0.72 |
| Q91Z49 | UAP56-interacting factor OS=Mus musculus OX=10090 GN=Fyttd1 PE=1 SV=1 | Fyttd1 | 0.72 |
| Q01147 | Cyclic AMP-responsive element-binding protein 1 OS=Mus musculus OX=10090 GN=Creb1 PE=1 SV=2 | Creb1 | 0.72 |
| B1AQJ2 | Ubiquitin carboxyl-terminal hydrolase 36 OS=Mus musculus OX=10090 GN=Usp36 PE=1 SV=1 | Usp36 | 0.72 |
| Q8BGH2 | Sorting and assembly machinery component 50 homolog OS=Mus musculus OX=10090 GN=Samm50 PE=1 SV=1 | Samm50 | 0.72 |
| Q62511 | E3 ubiquitin-protein ligase ZFP91 OS=Mus musculus OX=10090 GN=Zfp91 PE=1 SV=3 | Zfp91 | 0.72 |
| Q9ER00 | Syntaxin-12 OS=Mus musculus OX=10090 GN=Stx12 PE=1 SV=1 | Stx12 | 0.72 |
| Q9JLV1 | BAG family molecular chaperone regulator 3 OS=Mus musculus OX=10090 GN=Bag3 PE=1 SV=2 | Bag3 | 0.72 |
| P48453 | Serine/threonine-protein phosphatase 2B catalytic subunit beta isoform OS=Mus musculus OX=10090 GN=Ppp3cb PE=1 SV=2 | Ppp3cb | 0.72 |
| Q9EQ28 | DNA polymerase delta subunit 3 OS=Mus musculus OX=10090 GN=Pold3 PE=1 SV=2 | Pold3 | 0.72 |
| Q8BNY6 | Neuronal calcium sensor 1 OS=Mus musculus OX=10090 GN=Ncs1 PE=1 SV=3 | Ncs1 | 0.73 |
| Q6R5N8 | Toll-like receptor 13 OS=Mus musculus OX=10090 GN=Tlr13 PE=1 SV=1 | Tlr13 | 0.73 |
| Q9CYN9 | Renin receptor OS=Mus musculus OX=10090 GN=Atp6ap2 PE=1 SV=2 | Atp6ap2 | 0.73 |
| Q9CXK9 | RNA-binding protein 33 OS=Mus musculus OX=10090 GN=Rbm33 PE=1 SV=2 | Rbm33 | 0.73 |
| Q8R2Q4 | Ribosome-releasing factor 2, mitochondrial OS=Mus musculus OX=10090 GN=Gfm2 PE=1 SV=2 | Gfm2 | 0.73 |
| Q8VCS6 | Mediator of RNA polymerase II transcription subunit 9 OS=Mus musculus OX=10090 GN=Med9 PE=1 SV=1 | Med9 | 0.73 |
| P97440 | Histone RNA hairpin-binding protein OS=Mus musculus OX=10090 GN=Slbp PE=1 SV=1 | Slbp | 0.73 |
| Q8QZS1 | 3-hydroxyisobutyryl-CoA hydrolase, mitochondrial OS=Mus musculus OX=10090 GN=Hibch PE=1 SV=1 | Hibch | 0.73 |
| P49443 | Protein phosphatase 1A OS=Mus musculus OX=10090 GN=Ppm1a PE=1 SV=1 | Ppm1a | 0.73 |
| Q91VC3 | Eukaryotic initiation factor 4A-III OS=Mus musculus OX=10090 GN=Eif4a3 PE=1 SV=3 | Eif4a3 | 0.73 |
| C0HKD8 | Microfibrillar-associated protein 1A OS=Mus musculus OX=10090 GN=Mfap1a PE=1 SV=1 | Mfap1a | 0.73 |
| Q9CZU4 | GTPase Era, mitochondrial OS=Mus musculus OX=10090 GN=Eral1 PE=2 SV=1 | Eral1 | 0.73 |
| O88271 | Craniofacial development protein 1 OS=Mus musculus OX=10090 GN=Cfdp1 PE=1 SV=1 | Cfdp1 | 0.73 |
| Q8BK30 | NADH dehydrogenase [ubiquinone] flavoprotein 3, mitochondrial OS=Mus musculus OX=10090 GN=Ndufv3 PE=1 SV=1 | Ndufv3 | 0.73 |
| Q9JIY2 | E3 ubiquitin-protein ligase Hakai OS=Mus musculus OX=10090 GN=Cbll1 PE=1 SV=1 | Cbll1 | 0.73 |
| P62852 | 40S ribosomal protein S25 OS=Mus musculus OX=10090 GN=Rps25 PE=1 SV=1 | Rps25 | 0.73 |
| Q03147 | Cyclin-dependent kinase 7 OS=Mus musculus OX=10090 GN=Cdk7 PE=1 SV=2 | Cdk7 | 0.73 |
| Q8K4P0 | pre-mRNA 3' end processing protein WDR33 OS=Mus musculus OX=10090 GN=Wdr33 PE=1 SV=1 | Wdr33 | 0.73 |
| Q9CQT7 | Desumoylating isopeptidase 1 OS=Mus musculus OX=10090 GN=Desi1 PE=1 SV=1 | Desi1 | 0.73 |
| Q3TRM4 | Patatin-like phospholipase domain-containing protein 6 OS=Mus musculus OX=10090 GN=Pnpla6 PE=1 SV=2 | Pnpla6 | 0.73 |
| Q8R3K3 | Pentatricopeptide repeat-containing protein 2, mitochondrial OS=Mus musculus OX=10090 GN=Ptcd2 PE=1 SV=1 | Ptcd2 | 0.73 |
| O88587 | Catechol O-methyltransferase OS=Mus musculus OX=10090 GN=Comt PE=1 SV=2 | Comt | 0.73 |
| Q9D6J5 | NADH dehydrogenase [ubiquinone] 1 beta subcomplex subunit 8, mitochondrial OS=Mus musculus OX=10090 GN=Ndufb8 PE=1 SV=1 | Ndufb8 | 0.73 |
| Q91WM1 | Spermatid perinuclear RNA-binding protein OS=Mus musculus OX=10090 GN=Strbp PE=1 SV=1 | Strbp | 0.73 |
| P10630 | Eukaryotic initiation factor 4A-II OS=Mus musculus OX=10090 GN=Eif4a2 PE=1 SV=2 | Eif4a2 | 0.73 |
| Q8BVU5 | ADP-ribose pyrophosphatase, mitochondrial OS=Mus musculus OX=10090 GN=Nudt9 PE=1 SV=1 | Nudt9 | 0.73 |
| Q9CRA8 | Exosome complex component RRP46 OS=Mus musculus OX=10090 GN=Exosc5 PE=1 SV=1 | Exosc5 | 0.73 |
| Q9EPR4 | Solute carrier family 23 member 2 OS=Mus musculus OX=10090 GN=Slc23a2 PE=1 SV=2 | Slc23a2 | 0.73 |
| Q9CQE6 | Histone chaperone ASF1A OS=Mus musculus OX=10090 GN=Asf1a PE=1 SV=1 | Asf1a | 0.73 |
| Q923B1 | Lariat debranching enzyme OS=Mus musculus OX=10090 GN=Dbr1 PE=1 SV=2 | Dbr1 | 0.73 |
| Q9D786 | HAUS augmin-like complex subunit 5 OS=Mus musculus OX=10090 GN=Haus5 PE=1 SV=1 | Haus5 | 0.73 |
| P54729 | NEDD8 ultimate buster 1 OS=Mus musculus OX=10090 GN=Nub1 PE=1 SV=2 | Nub1 | 0.73 |
| Q9ERR7 | Selenoprotein F OS=Mus musculus OX=10090 GN=Selenof PE=1 SV=3 | Selenof | 0.73 |
| Q99JV5 | StAR-related lipid transfer protein 4 OS=Mus musculus OX=10090 GN=Stard4 PE=1 SV=1 | Stard4 | 0.73 |
| Q5SUA5 | Unconventional myosin-Ig OS=Mus musculus OX=10090 GN=Myo1g PE=1 SV=1 | Myo1g | 0.73 |
| P61255 | 60S ribosomal protein L26 OS=Mus musculus OX=10090 GN=Rpl26 PE=1 SV=1 | Rpl26 | 0.73 |
| Q9DBR2 | Protein FAM13C OS=Mus musculus OX=10090 GN=Fam13c PE=1 SV=2 | Fam13c | 0.73 |
| Q9D968 | Host cell factor 2 OS=Mus musculus OX=10090 GN=Hcfc2 PE=1 SV=2 | Hcfc2 | 0.73 |
| Q99LI2 | Chloride channel CLIC-like protein 1 OS=Mus musculus OX=10090 GN=Clcc1 PE=1 SV=1 | Clcc1 | 0.73 |
| Q8VBT9 | Tether containing UBX domain for GLUT4 OS=Mus musculus OX=10090 GN=Aspscr1 PE=1 SV=1 | Aspscr1 | 0.73 |
| Q5PSV9 | Mediator of DNA damage checkpoint protein 1 OS=Mus musculus OX=10090 GN=Mdc1 PE=1 SV=1 | Mdc1 | 0.73 |
| Q9CY50 | Translocon-associated protein subunit alpha OS=Mus musculus OX=10090 GN=Ssr1 PE=1 SV=1 | Ssr1 | 0.73 |
| Q8CGZ0 | Calcium homeostasis endoplasmic reticulum protein OS=Mus musculus OX=10090 GN=Cherp PE=1 SV=1 | Cherp | 0.73 |
| Q62241 | U1 small nuclear ribonucleoprotein C OS=Mus musculus OX=10090 GN=Snrpc PE=1 SV=1 | Snrpc | 0.73 |
| P63030 | Mitochondrial pyruvate carrier 1 OS=Mus musculus OX=10090 GN=Mpc1 PE=1 SV=1 | Mpc1 | 0.73 |
| P39749 | Flap endonuclease 1 OS=Mus musculus OX=10090 GN=Fen1 PE=1 SV=1 | Fen1 | 0.73 |
| A2AQ19 | RNA polymerase-associated protein RTF1 homolog OS=Mus musculus OX=10090 GN=Rtf1 PE=1 SV=1 | Rtf1 | 0.74 |
| P70459 | ETS domain-containing transcription factor ERF OS=Mus musculus OX=10090 GN=Erf PE=1 SV=1 | Erf | 0.74 |
| Q9JHD2 | Histone acetyltransferase KAT2A OS=Mus musculus OX=10090 GN=Kat2a PE=1 SV=2 | Kat2a | 0.74 |
| Q8K2I4 | Beta-mannosidase OS=Mus musculus OX=10090 GN=Manba PE=1 SV=1 | Manba | 0.74 |
| Q9D024 | PAT complex subunit CCDC47 OS=Mus musculus OX=10090 GN=Ccdc47 PE=1 SV=2 | Ccdc47 | 0.74 |
| Q9DB73 | NADH-cytochrome b5 reductase 1 OS=Mus musculus OX=10090 GN=Cyb5r1 PE=1 SV=1 | Cyb5r1 | 0.74 |
| Q9D7B6 | Isobutyryl-CoA dehydrogenase, mitochondrial OS=Mus musculus OX=10090 GN=Acad8 PE=1 SV=2 | Acad8 | 0.74 |
| Q3UI43 | BRISC and BRCA1-A complex member 1 OS=Mus musculus OX=10090 GN=Babam1 PE=1 SV=1 | Babam1 | 0.74 |
| Q8VBZ0 | Dehydrogenase/reductase SDR family member on chromosome X homolog OS=Mus musculus OX=10090 GN=Dhrsx PE=1 SV=2 | Dhrsx | 0.74 |
| Q64518 | Sarcoplasmic/endoplasmic reticulum calcium ATPase 3 OS=Mus musculus OX=10090 GN=Atp2a3 PE=1 SV=4 | Atp2a3 | 0.74 |
| Q3U2P1 | Protein transport protein Sec24A OS=Mus musculus OX=10090 GN=Sec24a PE=1 SV=1 | Sec24a | 0.74 |
| P97465 | Docking protein 1 OS=Mus musculus OX=10090 GN=Dok1 PE=1 SV=2 | Dok1 | 0.74 |
| Q91VJ5 | Polyglutamine-binding protein 1 OS=Mus musculus OX=10090 GN=Pqbp1 PE=1 SV=1 | Pqbp1 | 0.74 |
| Q8BSF4 | Phosphatidylserine decarboxylase proenzyme, mitochondrial OS=Mus musculus OX=10090 GN=Pisd PE=2 SV=1 | Pisd | 0.74 |
| Q9R233 | Tapasin OS=Mus musculus OX=10090 GN=Tapbp PE=1 SV=2 | Tapbp | 0.74 |
| Q9QYJ3 | DnaJ homolog subfamily B member 1 OS=Mus musculus OX=10090 GN=Dnajb1 PE=1 SV=3 | Dnajb1 | 0.74 |
| Q60989 | E3 ubiquitin-protein ligase XIAP OS=Mus musculus OX=10090 GN=Xiap PE=1 SV=2 | Xiap | 0.74 |
| Q9Z2D1 | Myotubularin-related protein 2 OS=Mus musculus OX=10090 GN=Mtmr2 PE=1 SV=3 | Mtmr2 | 0.74 |
| P25911 | Tyrosine-protein kinase Lyn OS=Mus musculus OX=10090 GN=Lyn PE=1 SV=4 | Lyn | 0.74 |
| Q9JJF3 | Ribosomal oxygenase 1 OS=Mus musculus OX=10090 GN=Riox1 PE=1 SV=2 | Riox1 | 0.74 |
| O35368 | Interferon-activable protein 203 OS=Mus musculus OX=10090 GN=Ifi203 PE=1 SV=1 | Ifi203 | 0.74 |
| Q8CCJ3 | E3 UFM1-protein ligase 1 OS=Mus musculus OX=10090 GN=Ufl1 PE=1 SV=2 | Ufl1 | 0.74 |
| Q9CZ04 | COP9 signalosome complex subunit 7a OS=Mus musculus OX=10090 GN=Cops7a PE=1 SV=2 | Cops7a | 0.74 |
| Q9EQQ2 | Protein YIPF5 OS=Mus musculus OX=10090 GN=Yipf5 PE=1 SV=1 | Yipf5 | 0.74 |
| Q91WK0 | Leucine-rich repeat flightless-interacting protein 2 OS=Mus musculus OX=10090 GN=Lrrfip2 PE=1 SV=1 | Lrrfip2 | 0.74 |
| Q9CWG1 | Glioma pathogenesis-related protein 1 OS=Mus musculus OX=10090 GN=Glipr1 PE=2 SV=1 | Glipr1 | 0.74 |
| Q8K4F6 | 28S rRNA (cytosine-C(5))-methyltransferase OS=Mus musculus OX=10090 GN=Nsun5 PE=1 SV=2 | Nsun5 | 0.74 |
| O89090 | Transcription factor Sp1 OS=Mus musculus OX=10090 GN=Sp1 PE=1 SV=2 | Sp1 | 0.74 |
| P97479 | Unconventional myosin-VIIa OS=Mus musculus OX=10090 GN=Myo7a PE=1 SV=2 | Myo7a | 0.74 |
| P28798 | Progranulin OS=Mus musculus OX=10090 GN=Grn PE=1 SV=2 | Grn | 0.74 |
| Q3THK3 | General transcription factor IIF subunit 1 OS=Mus musculus OX=10090 GN=Gtf2f1 PE=1 SV=2 | Gtf2f1 | 0.74 |
| Q59J78 | NADH dehydrogenase [ubiquinone] 1 alpha subcomplex assembly factor 2 OS=Mus musculus OX=10090 GN=Ndufaf2 PE=1 SV=1 | Ndufaf2 | 0.74 |
| P21855 | B-cell differentiation antigen CD72 OS=Mus musculus OX=10090 GN=Cd72 PE=1 SV=2 | Cd72 | 0.74 |
| Q00547 | Hyaluronan mediated motility receptor OS=Mus musculus OX=10090 GN=Hmmr PE=1 SV=4 | Hmmr | 0.74 |
| Q8N9S3 | Activator of 90 kDa heat shock protein ATPase homolog 2 OS=Mus musculus OX=10090 GN=Ahsa2 PE=1 SV=2 | Ahsa2 | 0.75 |
| Q9Z2D8 | Methyl-CpG-binding domain protein 3 OS=Mus musculus OX=10090 GN=Mbd3 PE=1 SV=1 | Mbd3 | 0.75 |
| Q6P8H8 | Probable dolichyl pyrophosphate Glc1Man9GlcNAc2 alpha-1,3-glucosyltransferase OS=Mus musculus OX=10090 GN=Alg8 PE=2 SV=2 | Alg8 | 0.75 |
| Q9JHJ0 | Tropomodulin-3 OS=Mus musculus OX=10090 GN=Tmod3 PE=1 SV=1 | Tmod3 | 0.75 |
| Q99KB8 | Hydroxyacylglutathione hydrolase, mitochondrial OS=Mus musculus OX=10090 GN=Hagh PE=1 SV=2 | Hagh | 0.75 |
| B1AY10 | Transcriptional repressor NF-X1 OS=Mus musculus OX=10090 GN=Nfx1 PE=1 SV=1 | Nfx1 | 0.75 |
| O35551 | Rab GTPase-binding effector protein 1 OS=Mus musculus OX=10090 GN=Rabep1 PE=1 SV=2 | Rabep1 | 0.75 |
| Q8BKZ9 | Pyruvate dehydrogenase protein X component, mitochondrial OS=Mus musculus OX=10090 GN=Pdhx PE=1 SV=1 | Pdhx | 0.75 |
| Q922Y1 | UBX domain-containing protein 1 OS=Mus musculus OX=10090 GN=Ubxn1 PE=1 SV=1 | Ubxn1 | 0.75 |
| Q9JMD0 | BUB3-interacting and GLEBS motif-containing protein ZNF207 OS=Mus musculus OX=10090 GN=Znf207 PE=1 SV=1 | Znf207 | 0.75 |
| Q8K394 | Inactive phospholipase C-like protein 2 OS=Mus musculus OX=10090 GN=Plcl2 PE=1 SV=2 | Plcl2 | 0.75 |
| Q8VHL1 | Histone-lysine N-methyltransferase SETD7 OS=Mus musculus OX=10090 GN=Setd7 PE=1 SV=2 | Setd7 | 0.75 |
| Q9JIY5 | Serine protease HTRA2, mitochondrial OS=Mus musculus OX=10090 GN=Htra2 PE=1 SV=2 | Htra2 | 0.75 |
| Q9QXN3 | Activating signal cointegrator 1 OS=Mus musculus OX=10090 GN=Trip4 PE=1 SV=2 | Trip4 | 0.75 |
| Q91X78 | Erlin-1 OS=Mus musculus OX=10090 GN=Erlin1 PE=1 SV=2 | Erlin1 | 0.75 |
| Q5SS80 | Dehydrogenase/reductase SDR family member 13 OS=Mus musculus OX=10090 GN=Dhrs13 PE=1 SV=1 | Dhrs13 | 0.75 |
| P09671 | Superoxide dismutase [Mn], mitochondrial OS=Mus musculus OX=10090 GN=Sod2 PE=1 SV=3 | Sod2 | 0.75 |
| Q9EQI8 | 39S ribosomal protein L46, mitochondrial OS=Mus musculus OX=10090 GN=Mrpl46 PE=1 SV=1 | Mrpl46 | 0.75 |
| P97855 | Ras GTPase-activating protein-binding protein 1 OS=Mus musculus OX=10090 GN=G3bp1 PE=1 SV=1 | G3bp1 | 0.75 |
| Q91V41 | Ras-related protein Rab-14 OS=Mus musculus OX=10090 GN=Rab14 PE=1 SV=3 | Rab14 | 0.75 |
| Q8K327 | Chromosome alignment-maintaining phosphoprotein 1 OS=Mus musculus OX=10090 GN=Champ1 PE=1 SV=1 | Champ1 | 0.75 |
| Q08024 | Core-binding factor subunit beta OS=Mus musculus OX=10090 GN=Cbfb PE=1 SV=1 | Cbfb | 0.75 |
| O35231 | Kinesin-like protein KIFC3 OS=Mus musculus OX=10090 GN=Kifc3 PE=1 SV=4 | Kifc3 | 0.75 |
| Q9WV70 | Nucleolar complex protein 2 homolog OS=Mus musculus OX=10090 GN=Noc2l PE=1 SV=2 | Noc2l | 0.75 |
| Q8BHT6 | Beta-1,3-glucosyltransferase OS=Mus musculus OX=10090 GN=B3glct PE=1 SV=3 | B3glct | 0.75 |
| P12023 | Amyloid-beta precursor protein OS=Mus musculus OX=10090 GN=App PE=1 SV=3 | App | 0.75 |
| Q8BJ03 | Cytochrome c oxidase assembly protein COX15 homolog OS=Mus musculus OX=10090 GN=Cox15 PE=1 SV=1 | Cox15 | 0.75 |
| Q9EQK5 | Major vault protein OS=Mus musculus OX=10090 GN=Mvp PE=1 SV=4 | Mvp | 0.75 |
| Q8BGR2 | Volume-regulated anion channel subunit LRRC8D OS=Mus musculus OX=10090 GN=Lrrc8d PE=1 SV=1 | Lrrc8d | 0.75 |
| Q9D8Z1 | Activating signal cointegrator 1 complex subunit 1 OS=Mus musculus OX=10090 GN=Ascc1 PE=1 SV=1 | Ascc1 | 0.75 |
| Q9QXK2 | E3 ubiquitin-protein ligase RAD18 OS=Mus musculus OX=10090 GN=Rad18 PE=1 SV=2 | Rad18 | 0.75 |
| O08788 | Dynactin subunit 1 OS=Mus musculus OX=10090 GN=Dctn1 PE=1 SV=3 | Dctn1 | 0.75 |
| Q9D735 | Telomerase RNA component interacting RNase OS=Mus musculus OX=10090 GN=Trir PE=1 SV=1 | Trir | 0.75 |
| Q9CYH2 | Peroxiredoxin-like 2A OS=Mus musculus OX=10090 GN=Prxl2a PE=1 SV=2 | Prxl2a | 0.76 |
| P51660 | Peroxisomal multifunctional enzyme type 2 OS=Mus musculus OX=10090 GN=Hsd17b4 PE=1 SV=3 | Hsd17b4 | 0.76 |
| P70399 | TP53-binding protein 1 OS=Mus musculus OX=10090 GN=Tp53bp1 PE=1 SV=3 | Tp53bp1 | 0.76 |
| Q9CQL5 | 39S ribosomal protein L18, mitochondrial OS=Mus musculus OX=10090 GN=Mrpl18 PE=1 SV=1 | Mrpl18 | 0.76 |
| Q99KU1 | Dehydrodolichyl diphosphate synthase complex subunit Dhdds OS=Mus musculus OX=10090 GN=Dhdds PE=2 SV=1 | Dhdds | 0.76 |
| P49446 | Receptor-type tyrosine-protein phosphatase epsilon OS=Mus musculus OX=10090 GN=Ptpre PE=1 SV=3 | Ptpre | 0.76 |
| O70378 | ER membrane protein complex subunit 8 OS=Mus musculus OX=10090 GN=Emc8 PE=1 SV=1 | Emc8 | 0.76 |
| Q8BRG8 | Transmembrane protein 209 OS=Mus musculus OX=10090 GN=Tmem209 PE=2 SV=1 | Tmem209 | 0.76 |
| Q8BGI5 | Peroxisome assembly protein 26 OS=Mus musculus OX=10090 GN=Pex26 PE=1 SV=2 | Pex26 | 0.76 |
| Q78IK2 | ATP synthase membrane subunit K, mitochondrial OS=Mus musculus OX=10090 GN=Atp5mk PE=1 SV=1 | Atp5mk | 0.76 |
| O88286 | Protein Wiz OS=Mus musculus OX=10090 GN=Wiz PE=1 SV=2 | Wiz | 0.76 |
| A2ADY9 | Protein DDI1 homolog 2 OS=Mus musculus OX=10090 GN=Ddi2 PE=1 SV=1 | Ddi2 | 0.76 |
| Q3TYS2 | Cytochrome b-245 chaperone 1 OS=Mus musculus OX=10090 GN=Cybc1 PE=1 SV=2 | Cybc1 | 0.76 |
| Q91XI1 | tRNA-dihydrouridine(47) synthase [NAD(P)(+)]-like OS=Mus musculus OX=10090 GN=Dus3l PE=1 SV=1 | Dus3l | 0.76 |
| Q8K2H2 | Deubiquitinase OTUD6B OS=Mus musculus OX=10090 GN=Otud6b PE=1 SV=1 | Otud6b | 0.76 |
| Q8K1R7 | Serine/threonine-protein kinase Nek9 OS=Mus musculus OX=10090 GN=Nek9 PE=1 SV=2 | Nek9 | 0.76 |
| Q91ZR1 | Ras-related protein Rab-4B OS=Mus musculus OX=10090 GN=Rab4b PE=1 SV=2 | Rab4b | 0.76 |
| Q91X20 | Set1/Ash2 histone methyltransferase complex subunit ASH2 OS=Mus musculus OX=10090 GN=Ash2l PE=1 SV=1 | Ash2l | 0.76 |
| P70349 | Adenosine 5'-monophosphoramidase HINT1 OS=Mus musculus OX=10090 GN=Hint1 PE=1 SV=3 | Hint1 | 0.76 |
| P97477 | Aurora kinase A OS=Mus musculus OX=10090 GN=Aurka PE=1 SV=1 | Aurka | 0.76 |
| Q924H2 | Mediator of RNA polymerase II transcription subunit 15 OS=Mus musculus OX=10090 GN=Med15 PE=1 SV=3 | Med15 | 0.76 |
| Q5U430 | E3 ubiquitin-protein ligase UBR3 OS=Mus musculus OX=10090 GN=Ubr3 PE=1 SV=3 | Ubr3 | 0.76 |
| P53569 | CCAAT/enhancer-binding protein zeta OS=Mus musculus OX=10090 GN=Cebpz PE=1 SV=2 | Cebpz | 0.76 |
| Q64012 | RNA-binding protein Raly OS=Mus musculus OX=10090 GN=Raly PE=1 SV=3 | Raly | 0.76 |
| Q8CBE3 | WD repeat-containing protein 37 OS=Mus musculus OX=10090 GN=Wdr37 PE=1 SV=1 | Wdr37 | 0.76 |
| Q9QXT0 | Protein canopy homolog 2 OS=Mus musculus OX=10090 GN=Cnpy2 PE=1 SV=1 | Cnpy2 | 0.76 |
| P21619 | Lamin-B2 OS=Mus musculus OX=10090 GN=Lmnb2 PE=1 SV=2 | Lmnb2 | 0.76 |
| Q8K019 | Bcl-2-associated transcription factor 1 OS=Mus musculus OX=10090 GN=Bclaf1 PE=1 SV=2 | Bclaf1 | 0.76 |
| Q8R3G1 | Nuclear inhibitor of protein phosphatase 1 OS=Mus musculus OX=10090 GN=Ppp1r8 PE=1 SV=1 | Ppp1r8 | 0.76 |
| P97304 | DNA-directed RNA polymerases I and III subunit RPAC2 OS=Mus musculus OX=10090 GN=Polr1d PE=1 SV=1 | Polr1d | 0.76 |
| P10649 | Glutathione S-transferase Mu 1 OS=Mus musculus OX=10090 GN=Gstm1 PE=1 SV=2 | Gstm1 | 0.76 |
| Q9WTK5 | Nuclear factor NF-kappa-B p100 subunit OS=Mus musculus OX=10090 GN=Nfkb2 PE=1 SV=1 | Nfkb2 | 0.76 |
| P16879 | Tyrosine-protein kinase Fes/Fps OS=Mus musculus OX=10090 GN=Fes PE=1 SV=2 | Fes | 0.76 |
| Q04207 | Transcription factor p65 OS=Mus musculus OX=10090 GN=Rela PE=1 SV=1 | Rela | 0.76 |
| Q03265 | ATP synthase subunit alpha, mitochondrial OS=Mus musculus OX=10090 GN=Atp5f1a PE=1 SV=1 | Atp5f1a | 0.76 |
| P26231 | Catenin alpha-1 OS=Mus musculus OX=10090 GN=Ctnna1 PE=1 SV=1 | Ctnna1 | 0.76 |
| Q8BKX1 | Brain-specific angiogenesis inhibitor 1-associated protein 2 OS=Mus musculus OX=10090 GN=Baiap2 PE=1 SV=2 | Baiap2 | 0.76 |
| Q9ESZ8 | General transcription factor II-I OS=Mus musculus OX=10090 GN=Gtf2i PE=1 SV=3 | Gtf2i | 0.76 |
| Q99JI6 | Ras-related protein Rap-1b OS=Mus musculus OX=10090 GN=Rap1b PE=1 SV=2 | Rap1b | 0.76 |
| Q9D125 | 28S ribosomal protein S25, mitochondrial OS=Mus musculus OX=10090 GN=Mrps25 PE=1 SV=1 | Mrps25 | 0.76 |
| Q80X90 | Filamin-B OS=Mus musculus OX=10090 GN=Flnb PE=1 SV=3 | Flnb | 0.77 |
| Q7TNG5 | Echinoderm microtubule-associated protein-like 2 OS=Mus musculus OX=10090 GN=Eml2 PE=1 SV=1 | Eml2 | 0.77 |
| Q3V009 | Transmembrane emp24 domain-containing protein 1 OS=Mus musculus OX=10090 GN=Tmed1 PE=1 SV=1 | Tmed1 | 0.77 |
| O09000 | Nuclear receptor coactivator 3 OS=Mus musculus OX=10090 GN=Ncoa3 PE=1 SV=2 | Ncoa3 | 0.77 |
| P70444 | BH3-interacting domain death agonist OS=Mus musculus OX=10090 GN=Bid PE=1 SV=2 | Bid | 0.77 |
| Q9WUU8 | TNFAIP3-interacting protein 1 OS=Mus musculus OX=10090 GN=Tnip1 PE=1 SV=1 | Tnip1 | 0.77 |
| Q9CR27 | WASH complex subunit 3 OS=Mus musculus OX=10090 GN=Washc3 PE=1 SV=1 | Washc3 | 0.77 |
| Q810S1 | Calcium uniporter regulatory subunit MCUb, mitochondrial OS=Mus musculus OX=10090 GN=Mcub PE=1 SV=1 | Mcub | 0.77 |
| Q8BLH7 | HIRA-interacting protein 3 OS=Mus musculus OX=10090 GN=Hirip3 PE=1 SV=1 | Hirip3 | 0.77 |
| Q3TNH5 | Cotranscriptional regulator FAM172A OS=Mus musculus OX=10090 GN=Fam172a PE=1 SV=2 | Fam172a | 0.77 |
| Q7TT18 | Activating transcription factor 7-interacting protein 1 OS=Mus musculus OX=10090 GN=Atf7ip PE=1 SV=1 | Atf7ip | 0.77 |
| Q8VCB1 | Nucleoporin NDC1 OS=Mus musculus OX=10090 GN=Ndc1 PE=1 SV=1 | Ndc1 | 0.77 |
| Q9D8T7 | SRA stem-loop-interacting RNA-binding protein, mitochondrial OS=Mus musculus OX=10090 GN=Slirp PE=1 SV=2 | Slirp | 0.77 |
| Q80V86 | Integrator complex subunit 8 OS=Mus musculus OX=10090 GN=Ints8 PE=1 SV=1 | Ints8 | 0.77 |
| Q9QXV1 | Chromobox protein homolog 8 OS=Mus musculus OX=10090 GN=Cbx8 PE=1 SV=1 | Cbx8 | 0.77 |
| Q69Z99 | Zinc finger protein 512 OS=Mus musculus OX=10090 GN=Znf512 PE=2 SV=2 | Znf512 | 0.77 |
| P70248 | Unconventional myosin-If OS=Mus musculus OX=10090 GN=Myo1f PE=1 SV=1 | Myo1f | 0.77 |
| Q6A4J8 | Ubiquitin carboxyl-terminal hydrolase 7 OS=Mus musculus OX=10090 GN=Usp7 PE=1 SV=1 | Usp7 | 0.77 |
| Q9DBU0 | Transmembrane 9 superfamily member 1 OS=Mus musculus OX=10090 GN=Tm9sf1 PE=2 SV=2 | Tm9sf1 | 0.77 |
| P62996 | Transformer-2 protein homolog beta OS=Mus musculus OX=10090 GN=Tra2b PE=1 SV=1 | Tra2b | 0.77 |
| Q9R0E2 | Procollagen-lysine,2-oxoglutarate 5-dioxygenase 1 OS=Mus musculus OX=10090 GN=Plod1 PE=1 SV=1 | Plod1 | 0.77 |
| Q3TFQ1 | SPRY domain-containing protein 7 OS=Mus musculus OX=10090 GN=Spryd7 PE=1 SV=2 | Spryd7 | 0.77 |
| Q9WTP6 | Adenylate kinase 2, mitochondrial OS=Mus musculus OX=10090 GN=Ak2 PE=1 SV=5 | Ak2 | 0.77 |
| Q80SY5 | Pre-mRNA-splicing factor 38B OS=Mus musculus OX=10090 GN=Prpf38b PE=1 SV=1 | Prpf38b | 0.77 |
| Q9D2V7 | Coronin-7 OS=Mus musculus OX=10090 GN=Coro7 PE=1 SV=2 | Coro7 | 0.77 |
| Q8BIH0 | Histone deacetylase complex subunit SAP130 OS=Mus musculus OX=10090 GN=Sap130 PE=1 SV=2 | Sap130 | 0.77 |
| P80316 | T-complex protein 1 subunit epsilon OS=Mus musculus OX=10090 GN=Cct5 PE=1 SV=1 | Cct5 | 0.77 |
| Q91YK2 | Ribosomal RNA processing protein 1 homolog B OS=Mus musculus OX=10090 GN=Rrp1b PE=1 SV=2 | Rrp1b | 0.77 |
| P19973 | Lymphocyte-specific protein 1 OS=Mus musculus OX=10090 GN=Lsp1 PE=1 SV=2 | Lsp1 | 0.77 |
| Q8BTI7 | Serine/threonine-protein phosphatase 6 regulatory ankyrin repeat subunit C OS=Mus musculus OX=10090 GN=Ankrd52 PE=1 SV=1 | Ankrd52 | 0.77 |
| Q6DID5 | PWWP domain-containing DNA repair factor 3A OS=Mus musculus OX=10090 GN=Pwwp3a PE=1 SV=1 | Pwwp3a | 0.77 |
| Q9D903 | Probable rRNA-processing protein EBP2 OS=Mus musculus OX=10090 GN=Ebna1bp2 PE=2 SV=1 | Ebna1bp2 | 0.77 |
| P61079 | Ubiquitin-conjugating enzyme E2 D3 OS=Mus musculus OX=10090 GN=Ube2d3 PE=1 SV=1 | Ube2d3 | 0.77 |
| Q9Z148 | Histone-lysine N-methyltransferase EHMT2 OS=Mus musculus OX=10090 GN=Ehmt2 PE=1 SV=2 | Ehmt2 | 0.77 |
| Q3UNA4 | NTF2-related export protein 2 OS=Mus musculus OX=10090 GN=Nxt2 PE=2 SV=1 | Nxt2 | 0.77 |
| A2ASS6 | Titin OS=Mus musculus OX=10090 GN=Ttn PE=1 SV=1 | Ttn | 0.77 |
| Q8K389 | CDK5 regulatory subunit-associated protein 2 OS=Mus musculus OX=10090 GN=Cdk5rap2 PE=1 SV=3 | Cdk5rap2 | 0.77 |
| Q8BLX4 | GDP-fucose transporter 1 OS=Mus musculus OX=10090 GN=Slc35c1 PE=2 SV=1 | Slc35c1 | 0.77 |
| Q6Y7W8 | GRB10-interacting GYF protein 2 OS=Mus musculus OX=10090 GN=Gigyf2 PE=1 SV=2 | Gigyf2 | 0.77 |
| Q61387 | Cytochrome c oxidase subunit 7A-related protein, mitochondrial OS=Mus musculus OX=10090 GN=Cox7a2l PE=1 SV=1 | Cox7a2l | 0.77 |
| Q3UUQ7 | GPI inositol-deacylase OS=Mus musculus OX=10090 GN=Pgap1 PE=1 SV=3 | Pgap1 | 0.77 |
| P15920 | V-type proton ATPase 116 kDa subunit a2 OS=Mus musculus OX=10090 GN=Atp6v0a2 PE=1 SV=2 | Atp6v0a2 | 0.77 |
| Q8K1E0 | Syntaxin-5 OS=Mus musculus OX=10090 GN=Stx5 PE=1 SV=3 | Stx5 | 0.77 |
| Q91WE1 | Sorting nexin-15 OS=Mus musculus OX=10090 GN=Snx15 PE=1 SV=1 | Snx15 | 0.77 |
| Q8BH74 | Nuclear pore complex protein Nup107 OS=Mus musculus OX=10090 GN=Nup107 PE=1 SV=1 | Nup107 | 0.78 |
| P21126 | Ubiquitin-like protein 4A OS=Mus musculus OX=10090 GN=Ubl4a PE=1 SV=1 | Ubl4a | 0.78 |
| Q8BI72 | CDKN2A-interacting protein OS=Mus musculus OX=10090 GN=Cdkn2aip PE=1 SV=1 | Cdkn2aip | 0.78 |
| Q80TQ2 | Ubiquitin carboxyl-terminal hydrolase CYLD OS=Mus musculus OX=10090 GN=Cyld PE=1 SV=2 | Cyld | 0.78 |
| Q9Z1T6 | 1-phosphatidylinositol 3-phosphate 5-kinase OS=Mus musculus OX=10090 GN=Pikfyve PE=1 SV=3 | Pikfyve | 0.78 |
| Q8C3X8 | Lipase maturation factor 2 OS=Mus musculus OX=10090 GN=Lmf2 PE=1 SV=1 | Lmf2 | 0.78 |
| P70452 | Syntaxin-4 OS=Mus musculus OX=10090 GN=Stx4 PE=1 SV=1 | Stx4 | 0.78 |
| O70333 | Cysteine-rich PDZ-binding protein OS=Mus musculus OX=10090 GN=Cript PE=1 SV=1 | Cript | 0.78 |
| Q810V0 | U3 small nucleolar ribonucleoprotein protein MPP10 OS=Mus musculus OX=10090 GN=Mphosph10 PE=1 SV=2 | Mphosph10 | 0.78 |
| P35821 | Tyrosine-protein phosphatase non-receptor type 1 OS=Mus musculus OX=10090 GN=Ptpn1 PE=1 SV=2 | Ptpn1 | 0.78 |
| Q9CPQ5 | Centromere protein Q OS=Mus musculus OX=10090 GN=Cenpq PE=2 SV=2 | Cenpq | 0.78 |
| Q9D620 | Rab11 family-interacting protein 1 OS=Mus musculus OX=10090 GN=Rab11fip1 PE=1 SV=2 | Rab11fip1 | 0.78 |
| Q8VCT3 | Aminopeptidase B OS=Mus musculus OX=10090 GN=Rnpep PE=1 SV=2 | Rnpep | 0.78 |
| Q99LX5 | Multiple myeloma tumor-associated protein 2 homolog OS=Mus musculus OX=10090 GN=Mmtag2 PE=2 SV=1 | Mmtag2 | 0.78 |
| Q9WVM3 | Anaphase-promoting complex subunit 7 OS=Mus musculus OX=10090 GN=Anapc7 PE=1 SV=3 | Anapc7 | 0.78 |
| P25976 | Nucleolar transcription factor 1 OS=Mus musculus OX=10090 GN=Ubtf PE=1 SV=1 | Ubtf | 0.78 |
| Q8BU33 | 2-hydroxyacyl-CoA lyase 2 OS=Mus musculus OX=10090 GN=Ilvbl PE=1 SV=1 | Ilvbl | 0.78 |
| Q8VE19 | GATOR complex protein MIOS OS=Mus musculus OX=10090 GN=Mios PE=1 SV=2 | Mios | 0.78 |
| Q62193 | Replication protein A 32 kDa subunit OS=Mus musculus OX=10090 GN=Rpa2 PE=1 SV=1 | Rpa2 | 0.78 |
| O55126 | Protein NipSnap homolog 2 OS=Mus musculus OX=10090 GN=Nipsnap2 PE=1 SV=1 | Nipsnap2 | 0.78 |
| P48410 | ATP-binding cassette sub-family D member 1 OS=Mus musculus OX=10090 GN=Abcd1 PE=1 SV=1 | Abcd1 | 0.78 |
| Q9DBG5 | Perilipin-3 OS=Mus musculus OX=10090 GN=Plin3 PE=1 SV=1 | Plin3 | 0.78 |
| Q4FZC9 | Nesprin-3 OS=Mus musculus OX=10090 GN=Syne3 PE=1 SV=1 | Syne3 | 0.78 |
| Q62481 | Vacuolar protein sorting-associated protein 72 homolog OS=Mus musculus OX=10090 GN=Vps72 PE=2 SV=2 | Vps72 | 0.78 |
| Q9Z2V5 | Histone deacetylase 6 OS=Mus musculus OX=10090 GN=Hdac6 PE=1 SV=3 | Hdac6 | 0.78 |
| Q9JI46 | Diphosphoinositol polyphosphate phosphohydrolase 1 OS=Mus musculus OX=10090 GN=Nudt3 PE=1 SV=1 | Nudt3 | 0.78 |
| P30204 | Macrophage scavenger receptor types I and II OS=Mus musculus OX=10090 GN=Msr1 PE=1 SV=3 | Msr1 | 0.78 |
| Q69ZR9 | Protein TASOR OS=Mus musculus OX=10090 GN=Tasor PE=1 SV=2 | Tasor | 0.78 |
| Q3THS6 | S-adenosylmethionine synthase isoform type-2 OS=Mus musculus OX=10090 GN=Mat2a PE=1 SV=2 | Mat2a | 0.78 |
| Q8VBW5 | HMG box transcription factor BBX OS=Mus musculus OX=10090 GN=Bbx PE=1 SV=2 | Bbx | 0.78 |
| P23591 | GDP-L-fucose synthase OS=Mus musculus OX=10090 GN=Gfus PE=1 SV=3 | Gfus | 0.78 |
| Q8VDQ1 | Prostaglandin reductase 2 OS=Mus musculus OX=10090 GN=Ptgr2 PE=1 SV=2 | Ptgr2 | 0.78 |
| Q9Z129 | ATP-dependent DNA helicase Q1 OS=Mus musculus OX=10090 GN=Recql PE=1 SV=2 | Recql | 0.78 |
| Q9R0P4 | Small acidic protein OS=Mus musculus OX=10090 GN=Smap PE=1 SV=1 | Smap | 0.78 |
| Q7TSZ8 | Nucleus accumbens-associated protein 1 OS=Mus musculus OX=10090 GN=Nacc1 PE=1 SV=1 | Nacc1 | 0.78 |
| Q6QD59 | Vesicle transport protein SEC20 OS=Mus musculus OX=10090 GN=Bnip1 PE=1 SV=1 | Bnip1 | 0.78 |
| Q6P3A8 | 2-oxoisovalerate dehydrogenase subunit beta, mitochondrial OS=Mus musculus OX=10090 GN=Bckdhb PE=1 SV=2 | Bckdhb | 0.78 |
| Q61070 | Etoposide-induced protein 2.4 OS=Mus musculus OX=10090 GN=Ei24 PE=1 SV=3 | Ei24 | 0.78 |
| Q9Z0H1 | WD repeat-containing protein 46 OS=Mus musculus OX=10090 GN=Wdr46 PE=2 SV=1 | Wdr46 | 0.78 |
| P47915 | 60S ribosomal protein L29 OS=Mus musculus OX=10090 GN=Rpl29 PE=1 SV=2 | Rpl29 | 0.78 |
| P70261 | Paladin OS=Mus musculus OX=10090 GN=Pald1 PE=1 SV=1 | Pald1 | 0.78 |
| Q9DBH5 | Vesicular integral-membrane protein VIP36 OS=Mus musculus OX=10090 GN=Lman2 PE=1 SV=2 | Lman2 | 0.78 |
| A2AJ88 | Patatin-like phospholipase domain-containing protein 7 OS=Mus musculus OX=10090 GN=Pnpla7 PE=1 SV=1 | Pnpla7 | 0.79 |
| Q80V03 | Uncharacterized aarF domain-containing protein kinase 5 OS=Mus musculus OX=10090 GN=Adck5 PE=2 SV=2 | Adck5 | 0.79 |
| Q8BTY2 | Sodium bicarbonate cotransporter 3 OS=Mus musculus OX=10090 GN=Slc4a7 PE=1 SV=2 | Slc4a7 | 0.79 |
| Q8VEK0 | Cell cycle control protein 50A OS=Mus musculus OX=10090 GN=Tmem30a PE=1 SV=1 | Tmem30a | 0.79 |
| P22682 | E3 ubiquitin-protein ligase CBL OS=Mus musculus OX=10090 GN=Cbl PE=1 SV=3 | Cbl | 0.79 |
| P70122 | Ribosome maturation protein SBDS OS=Mus musculus OX=10090 GN=Sbds PE=1 SV=4 | Sbds | 0.79 |
| P54726 | UV excision repair protein RAD23 homolog A OS=Mus musculus OX=10090 GN=Rad23a PE=1 SV=2 | Rad23a | 0.79 |
| Q8K409 | DNA polymerase beta OS=Mus musculus OX=10090 GN=Polb PE=1 SV=3 | Polb | 0.79 |
| O88512 | AP-1 complex subunit gamma-like 2 OS=Mus musculus OX=10090 GN=Ap1g2 PE=1 SV=2 | Ap1g2 | 0.79 |
| P21460 | Cystatin-C OS=Mus musculus OX=10090 GN=Cst3 PE=1 SV=2 | Cst3 | 0.79 |
| Q9D0T1 | NHP2-like protein 1 OS=Mus musculus OX=10090 GN=Snu13 PE=1 SV=4 | Snu13 | 0.79 |
| Q9D8H7 | Metalloendopeptidase OMA1, mitochondrial OS=Mus musculus OX=10090 GN=Oma1 PE=1 SV=1 | Oma1 | 0.79 |
| Q9DBL7 | Bifunctional coenzyme A synthase OS=Mus musculus OX=10090 GN=Coasy PE=1 SV=2 | Coasy | 0.79 |
| Q08297 | DNA repair protein RAD51 homolog 1 OS=Mus musculus OX=10090 GN=Rad51 PE=1 SV=1 | Rad51 | 0.79 |
| Q99L43 | Phosphatidate cytidylyltransferase 2 OS=Mus musculus OX=10090 GN=Cds2 PE=1 SV=1 | Cds2 | 0.79 |
| Q8BW96 | Calcium/calmodulin-dependent protein kinase type 1D OS=Mus musculus OX=10090 GN=Camk1d PE=1 SV=2 | Camk1d | 0.79 |
| Q9D1R2 | Protein KTI12 homolog OS=Mus musculus OX=10090 GN=Kti12 PE=1 SV=1 | Kti12 | 0.79 |
| P70336 | Rho-associated protein kinase 2 OS=Mus musculus OX=10090 GN=Rock2 PE=1 SV=1 | Rock2 | 0.79 |
| Q9D7N9 | Adipocyte plasma membrane-associated protein OS=Mus musculus OX=10090 GN=Apmap PE=1 SV=1 | Apmap | 0.79 |
| Q8CFK2 | Transcription factor IIIB 90 kDa subunit OS=Mus musculus OX=10090 GN=Brf1 PE=1 SV=1 | Brf1 | 0.79 |
| Q8K310 | Matrin-3 OS=Mus musculus OX=10090 GN=Matr3 PE=1 SV=1 | Matr3 | 0.79 |
| Q91VW3 | SH3 domain-binding glutamic acid-rich-like protein 3 OS=Mus musculus OX=10090 GN=Sh3bgrl3 PE=1 SV=1 | Sh3bgrl3 | 0.79 |
| P58137 | Acyl-coenzyme A thioesterase 8 OS=Mus musculus OX=10090 GN=Acot8 PE=1 SV=1 | Acot8 | 0.79 |
| Q8C3P7 | N6-adenosine-methyltransferase subunit METTL3 OS=Mus musculus OX=10090 GN=Mettl3 PE=1 SV=2 | Mettl3 | 0.79 |
| P27601 | Guanine nucleotide-binding protein subunit alpha-13 OS=Mus musculus OX=10090 GN=Gna13 PE=1 SV=1 | Gna13 | 0.79 |
| P08030 | Adenine phosphoribosyltransferase OS=Mus musculus OX=10090 GN=Aprt PE=1 SV=2 | Aprt | 0.79 |
| Q6ZQB6 | Inositol hexakisphosphate and diphosphoinositol-pentakisphosphate kinase 2 OS=Mus musculus OX=10090 GN=Ppip5k2 PE=1 SV=3 | Ppip5k2 | 0.79 |
| P46467 | Vacuolar protein sorting-associated protein 4B OS=Mus musculus OX=10090 GN=Vps4b PE=1 SV=2 | Vps4b | 0.79 |
| O88665 | Bromodomain-containing protein 7 OS=Mus musculus OX=10090 GN=Brd7 PE=1 SV=1 | Brd7 | 0.79 |
| P56395 | Cytochrome b5 OS=Mus musculus OX=10090 GN=Cyb5a PE=1 SV=2 | Cyb5a | 0.79 |
| Q9WV30 | Nuclear factor of activated T-cells 5 OS=Mus musculus OX=10090 GN=Nfat5 PE=1 SV=2 | Nfat5 | 0.79 |
| Q8VBW6 | NEDD8-activating enzyme E1 regulatory subunit OS=Mus musculus OX=10090 GN=Nae1 PE=1 SV=1 | Nae1 | 0.79 |
| Q6PA06 | Atlastin-2 OS=Mus musculus OX=10090 GN=Atl2 PE=1 SV=1 | Atl2 | 0.79 |
| P10922 | Histone H1.0 OS=Mus musculus OX=10090 GN=H1-0 PE=2 SV=4 | H1-0 | 0.79 |
| Q6A0D4 | Raftlin OS=Mus musculus OX=10090 GN=Rftn1 PE=1 SV=4 | Rftn1 | 0.79 |
| Q8VCN9 | Tubulin-specific chaperone C OS=Mus musculus OX=10090 GN=Tbcc PE=1 SV=1 | Tbcc | 0.79 |
| Q8K3A0 | Iron-sulfur cluster co-chaperone protein HscB OS=Mus musculus OX=10090 GN=Hscb PE=1 SV=2 | Hscb | 0.79 |
| Q9DCI9 | 39S ribosomal protein L32, mitochondrial OS=Mus musculus OX=10090 GN=Mrpl32 PE=1 SV=1 | Mrpl32 | 0.79 |
| Q8CBY8 | Dynactin subunit 4 OS=Mus musculus OX=10090 GN=Dctn4 PE=1 SV=1 | Dctn4 | 0.79 |
| Q69ZS7 | HBS1-like protein OS=Mus musculus OX=10090 GN=Hbs1l PE=1 SV=2 | Hbs1l | 0.79 |
| Q8VEK6 | Inhibitor of growth protein 3 OS=Mus musculus OX=10090 GN=Ing3 PE=1 SV=2 | Ing3 | 0.79 |
| Q9CZ13 | Cytochrome b-c1 complex subunit 1, mitochondrial OS=Mus musculus OX=10090 GN=Uqcrc1 PE=1 SV=2 | Uqcrc1 | 0.79 |
| Q6GYP7 | Ral GTPase-activating protein subunit alpha-1 OS=Mus musculus OX=10090 GN=Ralgapa1 PE=1 SV=1 | Ralgapa1 | 0.79 |
| Q8R1U1 | Conserved oligomeric Golgi complex subunit 4 OS=Mus musculus OX=10090 GN=Cog4 PE=1 SV=1 | Cog4 | 0.79 |
| Q5U4D9 | THO complex subunit 6 homolog OS=Mus musculus OX=10090 GN=Thoc6 PE=1 SV=1 | Thoc6 | 0.79 |
| Q8CJF7 | Protein ELYS OS=Mus musculus OX=10090 GN=Ahctf1 PE=1 SV=1 | Ahctf1 | 0.79 |
| Q3UPP8 | Centrosomal protein of 63 kDa OS=Mus musculus OX=10090 GN=Cep63 PE=1 SV=2 | Cep63 | 0.79 |
| Q8VEJ9 | Vacuolar protein sorting-associated protein 4A OS=Mus musculus OX=10090 GN=Vps4a PE=1 SV=1 | Vps4a | 0.79 |
| Q64261 | Cyclin-dependent kinase 6 OS=Mus musculus OX=10090 GN=Cdk6 PE=1 SV=2 | Cdk6 | 0.79 |
| Q6RT24 | Centromere-associated protein E OS=Mus musculus OX=10090 GN=Cenpe PE=1 SV=1 | Cenpe | 0.80 |
| B1AY13 | Ubiquitin carboxyl-terminal hydrolase 24 OS=Mus musculus OX=10090 GN=Usp24 PE=1 SV=1 | Usp24 | 0.80 |
| O35280 | Serine/threonine-protein kinase Chk1 OS=Mus musculus OX=10090 GN=Chek1 PE=1 SV=2 | Chek1 | 0.80 |
| Q8BM72 | Heat shock 70 kDa protein 13 OS=Mus musculus OX=10090 GN=Hspa13 PE=1 SV=1 | Hspa13 | 0.80 |
| Q8VDD8 | WASH complex subunit 1 OS=Mus musculus OX=10090 GN=Washc1 PE=1 SV=1 | Washc1 | 0.80 |
| O54941 | SWI/SNF-related matrix-associated actin-dependent regulator of chromatin subfamily E member 1 OS=Mus musculus OX=10090 GN=Smarce1 PE=1 SV=1 | Smarce1 | 0.80 |
| P24860 | G2/mitotic-specific cyclin-B1 OS=Mus musculus OX=10090 GN=Ccnb1 PE=1 SV=3 | Ccnb1 | 0.80 |
| Q6P5G6 | UBX domain-containing protein 7 OS=Mus musculus OX=10090 GN=Ubxn7 PE=1 SV=2 | Ubxn7 | 0.80 |
| Q9WV96 | Mitochondrial import inner membrane translocase subunit Tim10 B OS=Mus musculus OX=10090 GN=Timm10b PE=1 SV=1 | Timm10b | 0.80 |
| P19536 | Cytochrome c oxidase subunit 5B, mitochondrial OS=Mus musculus OX=10090 GN=Cox5b PE=1 SV=1 | Cox5b | 0.80 |
| O35114 | Lysosome membrane protein 2 OS=Mus musculus OX=10090 GN=Scarb2 PE=1 SV=3 | Scarb2 | 0.80 |
| Q9CXL3 | Uncharacterized protein C7orf50 homolog OS=Mus musculus OX=10090 PE=1 SV=3 | -- | 0.80 |
| Q8R080 | G2 and S phase-expressed protein 1 OS=Mus musculus OX=10090 GN=Gtse1 PE=1 SV=2 | Gtse1 | 0.80 |
| P35922 | Synaptic functional regulator FMR1 OS=Mus musculus OX=10090 GN=Fmr1 PE=1 SV=1 | Fmr1 | 0.80 |
| Q921M4 | Golgin subfamily A member 2 OS=Mus musculus OX=10090 GN=Golga2 PE=1 SV=3 | Golga2 | 0.80 |
| Q9CZ09 | Histidine protein methyltransferase 1 homolog OS=Mus musculus OX=10090 GN=Mettl18 PE=2 SV=2 | Mettl18 | 0.80 |
| P14901 | Heme oxygenase 1 OS=Mus musculus OX=10090 GN=Hmox1 PE=1 SV=1 | Hmox1 | 0.80 |
| Q9D0G0 | 28S ribosomal protein S30, mitochondrial OS=Mus musculus OX=10090 GN=Mrps30 PE=1 SV=1 | Mrps30 | 0.80 |
| Q6VN19 | Ran-binding protein 10 OS=Mus musculus OX=10090 GN=Ranbp10 PE=1 SV=2 | Ranbp10 | 0.80 |
| Q8C570 | mRNA export factor OS=Mus musculus OX=10090 GN=Rae1 PE=1 SV=1 | Rae1 | 0.80 |
| P27046 | Alpha-mannosidase 2 OS=Mus musculus OX=10090 GN=Man2a1 PE=1 SV=2 | Man2a1 | 0.80 |
| Q8K202 | DNA-directed RNA polymerase I subunit RPA49 OS=Mus musculus OX=10090 GN=Polr1e PE=1 SV=2 | Polr1e | 0.80 |
| P0DPE0 | EEF1A lysine methyltransferase 4 OS=Mus musculus OX=10090 GN=Eef1akmt4 PE=2 SV=1 | Eef1akmt4 | 0.80 |
| Q8CAK3 | Shiftless antiviral inhibitor of ribosomal frameshifting protein homolog OS=Mus musculus OX=10090 GN=Shfl PE=2 SV=1 | Shfl | 0.80 |
| Q8R2U0 | Nucleoporin SEH1 OS=Mus musculus OX=10090 GN=Seh1l PE=2 SV=1 | Seh1l | 0.80 |
| Q8K2A7 | Integrator complex subunit 10 OS=Mus musculus OX=10090 GN=Ints10 PE=1 SV=3 | Ints10 | 0.80 |
| Q91WM3 | U3 small nucleolar RNA-interacting protein 2 OS=Mus musculus OX=10090 GN=Rrp9 PE=1 SV=1 | Rrp9 | 0.80 |
| Q07417 | Short-chain specific acyl-CoA dehydrogenase, mitochondrial OS=Mus musculus OX=10090 GN=Acads PE=1 SV=2 | Acads | 0.80 |
| Q920L1 | Acyl-CoA (8-3)-desaturase OS=Mus musculus OX=10090 GN=Fads1 PE=1 SV=1 | Fads1 | 0.80 |
| P05063 | Fructose-bisphosphate aldolase C OS=Mus musculus OX=10090 GN=Aldoc PE=1 SV=4 | Aldoc | 0.80 |
| B2RXR6 | Serine/threonine-protein phosphatase 6 regulatory ankyrin repeat subunit B OS=Mus musculus OX=10090 GN=Ankrd44 PE=1 SV=1 | Ankrd44 | 0.80 |
| P05531 | X-linked lymphocyte-regulated protein PM1 OS=Mus musculus OX=10090 GN=Xlr PE=2 SV=1 | Xlr | 0.80 |
| Q8BP56 | Protein-glucosylgalactosylhydroxylysine glucosidase OS=Mus musculus OX=10090 GN=Pgghg PE=1 SV=1 | Pgghg | 0.80 |
| P70441 | Na(+)/H(+) exchange regulatory cofactor NHE-RF1 OS=Mus musculus OX=10090 GN=Slc9a3r1 PE=1 SV=3 | Slc9a3r1 | 0.80 |
| Q9WTX5 | S-phase kinase-associated protein 1 OS=Mus musculus OX=10090 GN=Skp1 PE=1 SV=3 | Skp1 | 0.80 |
| Q8BFR5 | Elongation factor Tu, mitochondrial OS=Mus musculus OX=10090 GN=Tufm PE=1 SV=1 | Tufm | 0.80 |
| Q8VE95 | UPF0598 protein C8orf82 homolog OS=Mus musculus OX=10090 PE=1 SV=1 | -- | 0.80 |
| Q8R180 | ERO1-like protein alpha OS=Mus musculus OX=10090 GN=Ero1a PE=1 SV=2 | Ero1a | 0.80 |
| Q6DFW4 | Nucleolar protein 58 OS=Mus musculus OX=10090 GN=Nop58 PE=1 SV=1 | Nop58 | 0.80 |
| Q6QI06 | Rapamycin-insensitive companion of mTOR OS=Mus musculus OX=10090 GN=Rictor PE=1 SV=2 | Rictor | 0.80 |
| Q8JZV7 | N-acetylglucosamine-6-phosphate deacetylase OS=Mus musculus OX=10090 GN=Amdhd2 PE=1 SV=1 | Amdhd2 | 0.80 |
| Q06180 | Tyrosine-protein phosphatase non-receptor type 2 OS=Mus musculus OX=10090 GN=Ptpn2 PE=1 SV=2 | Ptpn2 | 0.80 |
| A2BH40 | AT-rich interactive domain-containing protein 1A OS=Mus musculus OX=10090 GN=Arid1a PE=1 SV=1 | Arid1a | 0.80 |
| Q8C2P3 | tRNA-dihydrouridine(16/17) synthase [NAD(P)(+)]-like OS=Mus musculus OX=10090 GN=Dus1l PE=2 SV=1 | Dus1l | 0.80 |
| Q6PB44 | Tyrosine-protein phosphatase non-receptor type 23 OS=Mus musculus OX=10090 GN=Ptpn23 PE=1 SV=2 | Ptpn23 | 0.80 |
| Q9R1P4 | Proteasome subunit alpha type-1 OS=Mus musculus OX=10090 GN=Psma1 PE=1 SV=1 | Psma1 | 0.80 |
| Q9CPP6 | NADH dehydrogenase [ubiquinone] 1 alpha subcomplex subunit 5 OS=Mus musculus OX=10090 GN=Ndufa5 PE=1 SV=3 | Ndufa5 | 0.80 |
| P52912 | Nucleolysin TIA-1 OS=Mus musculus OX=10090 GN=Tia1 PE=1 SV=1 | Tia1 | 0.80 |
| Q05CL8 | La-related protein 7 OS=Mus musculus OX=10090 GN=Larp7 PE=1 SV=2 | Larp7 | 0.80 |
| Q9Z1N2 | Origin recognition complex subunit 1 OS=Mus musculus OX=10090 GN=Orc1 PE=1 SV=2 | Orc1 | 0.80 |
| Q99JR8 | SWI/SNF-related matrix-associated actin-dependent regulator of chromatin subfamily D member 2 OS=Mus musculus OX=10090 GN=Smarcd2 PE=1 SV=2 | Smarcd2 | 0.80 |
| Q149F1 | RNA pseudouridylate synthase domain-containing protein 2 OS=Mus musculus OX=10090 GN=Rpusd2 PE=1 SV=2 | Rpusd2 | 0.80 |
| P61514 | 60S ribosomal protein L37a OS=Mus musculus OX=10090 GN=Rpl37a PE=1 SV=2 | Rpl37a | 0.80 |
| Q3UMR5 | Calcium uniporter protein, mitochondrial OS=Mus musculus OX=10090 GN=Mcu PE=1 SV=2 | Mcu | 0.81 |
| Q9CYA0 | Protein disulfide isomerase Creld2 OS=Mus musculus OX=10090 GN=Creld2 PE=1 SV=1 | Creld2 | 0.81 |
| P62878 | E3 ubiquitin-protein ligase RBX1 OS=Mus musculus OX=10090 GN=Rbx1 PE=1 SV=1 | Rbx1 | 0.81 |
| O55022 | Membrane-associated progesterone receptor component 1 OS=Mus musculus OX=10090 GN=Pgrmc1 PE=1 SV=4 | Pgrmc1 | 0.81 |
| O54864 | Histone-lysine N-methyltransferase SUV39H1 OS=Mus musculus OX=10090 GN=Suv39h1 PE=1 SV=1 | Suv39h1 | 0.81 |
| Q6P1H6 | Ankyrin repeat and LEM domain-containing protein 2 OS=Mus musculus OX=10090 GN=Ankle2 PE=1 SV=2 | Ankle2 | 0.81 |
| Q9D753 | Exosome complex component RRP43 OS=Mus musculus OX=10090 GN=Exosc8 PE=1 SV=1 | Exosc8 | 0.81 |
| Q91VR2 | ATP synthase subunit gamma, mitochondrial OS=Mus musculus OX=10090 GN=Atp5f1c PE=1 SV=1 | Atp5f1c | 0.81 |
| Q99KI0 | Aconitate hydratase, mitochondrial OS=Mus musculus OX=10090 GN=Aco2 PE=1 SV=1 | Aco2 | 0.81 |
| Q99PL5 | Ribosome-binding protein 1 OS=Mus musculus OX=10090 GN=Rrbp1 PE=1 SV=2 | Rrbp1 | 0.81 |
| Q9Z0H3 | SWI/SNF-related matrix-associated actin-dependent regulator of chromatin subfamily B member 1 OS=Mus musculus OX=10090 GN=Smarcb1 PE=1 SV=1 | Smarcb1 | 0.81 |
| A2AL36 | Centriolin OS=Mus musculus OX=10090 GN=Cntrl PE=1 SV=2 | Cntrl | 0.81 |
| Q8R4R6 | Nucleoporin NUP35 OS=Mus musculus OX=10090 GN=Nup35 PE=1 SV=2 | Nup35 | 0.81 |
| P33174 | Chromosome-associated kinesin KIF4 OS=Mus musculus OX=10090 GN=Kif4 PE=1 SV=3 | Kif4 | 0.81 |
| Q80WG5 | Volume-regulated anion channel subunit LRRC8A OS=Mus musculus OX=10090 GN=Lrrc8a PE=1 SV=1 | Lrrc8a | 0.81 |
| Q8BNW9 | Kelch repeat and BTB domain-containing protein 11 OS=Mus musculus OX=10090 GN=Kbtbd11 PE=1 SV=3 | Kbtbd11 | 0.81 |
| Q8VI84 | Nucleolar complex protein 3 homolog OS=Mus musculus OX=10090 GN=Noc3l PE=2 SV=2 | Noc3l | 0.81 |
| O35218 | Cleavage and polyadenylation specificity factor subunit 2 OS=Mus musculus OX=10090 GN=Cpsf2 PE=1 SV=1 | Cpsf2 | 0.81 |
| Q8K1R3 | Polyribonucleotide nucleotidyltransferase 1, mitochondrial OS=Mus musculus OX=10090 GN=Pnpt1 PE=1 SV=1 | Pnpt1 | 0.81 |
| Q9WU40 | Inner nuclear membrane protein Man1 OS=Mus musculus OX=10090 GN=Lemd3 PE=1 SV=2 | Lemd3 | 0.81 |
| Q05920 | Pyruvate carboxylase, mitochondrial OS=Mus musculus OX=10090 GN=Pc PE=1 SV=1 | Pc | 0.81 |
| P40630 | Transcription factor A, mitochondrial OS=Mus musculus OX=10090 GN=Tfam PE=1 SV=2 | Tfam | 0.81 |
| Q9EP52 | Twisted gastrulation protein homolog 1 OS=Mus musculus OX=10090 GN=Twsg1 PE=1 SV=1 | Twsg1 | 0.81 |
| Q9CY57 | Chromatin target of PRMT1 protein OS=Mus musculus OX=10090 GN=Chtop PE=1 SV=2 | Chtop | 0.81 |
| Q9JKY0 | CCR4-NOT transcription complex subunit 9 OS=Mus musculus OX=10090 GN=Cnot9 PE=1 SV=1 | Cnot9 | 0.81 |
| Q99KH8 | Serine/threonine-protein kinase 24 OS=Mus musculus OX=10090 GN=Stk24 PE=1 SV=1 | Stk24 | 0.81 |
| Q02248 | Catenin beta-1 OS=Mus musculus OX=10090 GN=Ctnnb1 PE=1 SV=1 | Ctnnb1 | 0.81 |
| Q3URS9 | Mitochondrial potassium channel OS=Mus musculus OX=10090 GN=Ccdc51 PE=1 SV=1 | Ccdc51 | 0.81 |
| Q9D1J3 | SAP domain-containing ribonucleoprotein OS=Mus musculus OX=10090 GN=Sarnp PE=1 SV=3 | Sarnp | 0.81 |
| Q99MR6 | Serrate RNA effector molecule homolog OS=Mus musculus OX=10090 GN=Srrt PE=1 SV=1 | Srrt | 0.81 |
| P08752 | Guanine nucleotide-binding protein G(i) subunit alpha-2 OS=Mus musculus OX=10090 GN=Gnai2 PE=1 SV=5 | Gnai2 | 0.81 |
| Q8C5P5 | 5'-nucleotidase domain-containing protein 1 OS=Mus musculus OX=10090 GN=Nt5dc1 PE=1 SV=2 | Nt5dc1 | 0.81 |
| Q9CR21 | Acyl carrier protein, mitochondrial OS=Mus musculus OX=10090 GN=Ndufab1 PE=1 SV=1 | Ndufab1 | 0.81 |
| Q9CZT4 | DNA-directed RNA polymerase III subunit RPC5 OS=Mus musculus OX=10090 GN=Polr3e PE=1 SV=2 | Polr3e | 0.81 |
| Q8CHP5 | Partner of Y14 and mago OS=Mus musculus OX=10090 GN=Pym1 PE=1 SV=2 | Pym1 | 0.81 |
| Q64310 | Surfeit locus protein 4 OS=Mus musculus OX=10090 GN=Surf4 PE=1 SV=1 | Surf4 | 0.81 |
| O35601 | FYN-binding protein 1 OS=Mus musculus OX=10090 GN=Fyb1 PE=1 SV=2 | Fyb1 | 0.81 |
| Q3TC93 | HCLS1-binding protein 3 OS=Mus musculus OX=10090 GN=Hs1bp3 PE=1 SV=2 | Hs1bp3 | 0.81 |
| Q8VDP4 | Cell cycle and apoptosis regulator protein 2 OS=Mus musculus OX=10090 GN=Ccar2 PE=1 SV=2 | Ccar2 | 0.81 |
| Q8VCY6 | U3 small nucleolar RNA-associated protein 6 homolog OS=Mus musculus OX=10090 GN=Utp6 PE=2 SV=1 | Utp6 | 0.81 |
| Q9D358 | Low molecular weight phosphotyrosine protein phosphatase OS=Mus musculus OX=10090 GN=Acp1 PE=1 SV=3 | Acp1 | 0.81 |
| Q8K212 | Phosphofurin acidic cluster sorting protein 1 OS=Mus musculus OX=10090 GN=Pacs1 PE=1 SV=2 | Pacs1 | 0.81 |
| P0C0A3 | Charged multivesicular body protein 6 OS=Mus musculus OX=10090 GN=Chmp6 PE=1 SV=2 | Chmp6 | 0.81 |
| P52332 | Tyrosine-protein kinase JAK1 OS=Mus musculus OX=10090 GN=Jak1 PE=1 SV=1 | Jak1 | 0.81 |
| Q9CQQ8 | U6 snRNA-associated Sm-like protein LSm7 OS=Mus musculus OX=10090 GN=Lsm7 PE=1 SV=1 | Lsm7 | 0.81 |
| Q9JM93 | ADP-ribosylation factor-like protein 6-interacting protein 4 OS=Mus musculus OX=10090 GN=Arl6ip4 PE=1 SV=1 | Arl6ip4 | 0.81 |
| Q9CQS8 | Protein transport protein Sec61 subunit beta OS=Mus musculus OX=10090 GN=Sec61b PE=1 SV=3 | Sec61b | 0.81 |
| Q3UL36 | Arginine and glutamate-rich protein 1 OS=Mus musculus OX=10090 GN=Arglu1 PE=1 SV=2 | Arglu1 | 0.81 |
| Q8R3F5 | Malonyl-CoA-acyl carrier protein transacylase, mitochondrial OS=Mus musculus OX=10090 GN=Mcat PE=1 SV=3 | Mcat | 0.81 |
| O08784 | Treacle protein OS=Mus musculus OX=10090 GN=Tcof1 PE=1 SV=1 | Tcof1 | 0.81 |
| Q4VBE8 | WD repeat-containing protein 18 OS=Mus musculus OX=10090 GN=Wdr18 PE=1 SV=1 | Wdr18 | 0.81 |
| Q9CQE8 | RNA transcription, translation and transport factor protein OS=Mus musculus OX=10090 GN=RTRAF PE=1 SV=1 | RTRAF | 0.81 |
| Q64213 | Splicing factor 1 OS=Mus musculus OX=10090 GN=Sf1 PE=1 SV=6 | Sf1 | 0.81 |
| P70699 | Lysosomal alpha-glucosidase OS=Mus musculus OX=10090 GN=Gaa PE=1 SV=2 | Gaa | 0.81 |
| Q8C5L7 | RNA-binding protein 34 OS=Mus musculus OX=10090 GN=Rbm34 PE=1 SV=2 | Rbm34 | 0.81 |
| Q0VEE6 | Zinc finger protein 800 OS=Mus musculus OX=10090 GN=Znf800 PE=1 SV=1 | Znf800 | 0.81 |
| Q9CXK8 | 60S ribosome subunit biogenesis protein NIP7 homolog OS=Mus musculus OX=10090 GN=Nip7 PE=1 SV=1 | Nip7 | 0.81 |
| Q9D855 | Cytochrome b-c1 complex subunit 7 OS=Mus musculus OX=10090 GN=Uqcrb PE=1 SV=3 | Uqcrb | 0.81 |
| Q8BKT7 | THO complex subunit 5 homolog OS=Mus musculus OX=10090 GN=Thoc5 PE=1 SV=2 | Thoc5 | 0.81 |
| Q8C0E2 | Vacuolar protein sorting-associated protein 26B OS=Mus musculus OX=10090 GN=Vps26b PE=1 SV=1 | Vps26b | 0.81 |
| Q8BI84 | Transport and Golgi organization protein 1 homolog OS=Mus musculus OX=10090 GN=Mia3 PE=1 SV=2 | Mia3 | 0.82 |
| Q8CFC7 | CLK4-associating serine/arginine rich protein OS=Mus musculus OX=10090 GN=Clasrp PE=1 SV=3 | Clasrp | 0.82 |
| Q9DB43 | Zinc finger protein-like 1 OS=Mus musculus OX=10090 GN=Zfpl1 PE=1 SV=1 | Zfpl1 | 0.82 |
| Q9QZM0 | Ubiquilin-2 OS=Mus musculus OX=10090 GN=Ubqln2 PE=1 SV=2 | Ubqln2 | 0.82 |
| Q9JKF7 | 39S ribosomal protein L39, mitochondrial OS=Mus musculus OX=10090 GN=Mrpl39 PE=1 SV=4 | Mrpl39 | 0.82 |
| Q9WUM3 | Coronin-1B OS=Mus musculus OX=10090 GN=Coro1b PE=1 SV=1 | Coro1b | 0.82 |
| P67984 | 60S ribosomal protein L22 OS=Mus musculus OX=10090 GN=Rpl22 PE=1 SV=2 | Rpl22 | 0.82 |
| Q9CZX5 | PIN2/TERF1-interacting telomerase inhibitor 1 OS=Mus musculus OX=10090 GN=Pinx1 PE=1 SV=2 | Pinx1 | 0.82 |
| Q04646 | Sodium/potassium-transporting ATPase subunit gamma OS=Mus musculus OX=10090 GN=Fxyd2 PE=1 SV=2 | Fxyd2 | 0.82 |
| Q61334 | B-cell receptor-associated protein 29 OS=Mus musculus OX=10090 GN=Bcap29 PE=1 SV=1 | Bcap29 | 0.82 |
| Q99K74 | Mediator of RNA polymerase II transcription subunit 24 OS=Mus musculus OX=10090 GN=Med24 PE=1 SV=1 | Med24 | 0.82 |
| Q9CT10 | Ran-binding protein 3 OS=Mus musculus OX=10090 GN=Ranbp3 PE=1 SV=2 | Ranbp3 | 0.82 |
| Q99KR3 | Endoribonuclease LACTB2 OS=Mus musculus OX=10090 GN=Lactb2 PE=1 SV=1 | Lactb2 | 0.82 |
| Q80XL6 | Acyl-CoA dehydrogenase family member 11 OS=Mus musculus OX=10090 GN=Acad11 PE=1 SV=2 | Acad11 | 0.82 |
| Q8VBT0 | Thioredoxin-related transmembrane protein 1 OS=Mus musculus OX=10090 GN=Tmx1 PE=1 SV=1 | Tmx1 | 0.82 |
| Q8K273 | ER membrane protein complex subunit 5 OS=Mus musculus OX=10090 GN=Mmgt1 PE=1 SV=1 | Mmgt1 | 0.82 |
| Q80U63 | Mitofusin-2 OS=Mus musculus OX=10090 GN=Mfn2 PE=1 SV=3 | Mfn2 | 0.82 |
| Q9D198 | Pre-mRNA-splicing factor SYF2 OS=Mus musculus OX=10090 GN=Syf2 PE=2 SV=1 | Syf2 | 0.82 |
| P39428 | TNF receptor-associated factor 1 OS=Mus musculus OX=10090 GN=Traf1 PE=1 SV=2 | Traf1 | 0.82 |
| Q80W47 | WD repeat domain phosphoinositide-interacting protein 2 OS=Mus musculus OX=10090 GN=Wipi2 PE=1 SV=1 | Wipi2 | 0.82 |
| Q3U3C9 | Genetic suppressor element 1 OS=Mus musculus OX=10090 GN=Gse1 PE=1 SV=2 | Gse1 | 0.82 |
| Q6PAM1 | Alpha-taxilin OS=Mus musculus OX=10090 GN=Txlna PE=1 SV=1 | Txlna | 0.82 |
| P50136 | 2-oxoisovalerate dehydrogenase subunit alpha, mitochondrial OS=Mus musculus OX=10090 GN=Bckdha PE=1 SV=1 | Bckdha | 0.82 |
| Q9QZD8 | Mitochondrial dicarboxylate carrier OS=Mus musculus OX=10090 GN=Slc25a10 PE=1 SV=2 | Slc25a10 | 0.82 |
| Q8K2H6 | Anaphase-promoting complex subunit 10 OS=Mus musculus OX=10090 GN=Anapc10 PE=1 SV=1 | Anapc10 | 0.82 |
| Q8VCF1 | Soluble calcium-activated nucleotidase 1 OS=Mus musculus OX=10090 GN=Cant1 PE=2 SV=1 | Cant1 | 0.82 |
| Q8BH60 | Golgi-associated PDZ and coiled-coil motif-containing protein OS=Mus musculus OX=10090 GN=Gopc PE=1 SV=1 | Gopc | 0.82 |
| P26618 | Platelet-derived growth factor receptor alpha OS=Mus musculus OX=10090 GN=Pdgfra PE=1 SV=3 | Pdgfra | 0.82 |
| Q8K1I7 | WAS/WASL-interacting protein family member 1 OS=Mus musculus OX=10090 GN=Wipf1 PE=1 SV=1 | Wipf1 | 0.82 |
| P31651 | Sodium- and chloride-dependent betaine transporter OS=Mus musculus OX=10090 GN=Slc6a12 PE=2 SV=1 | Slc6a12 | 0.82 |
| Q9EPK7 | Exportin-7 OS=Mus musculus OX=10090 GN=Xpo7 PE=1 SV=3 | Xpo7 | 0.82 |
| Q9CQ54 | NADH dehydrogenase [ubiquinone] 1 subunit C2 OS=Mus musculus OX=10090 GN=Ndufc2 PE=1 SV=1 | Ndufc2 | 0.82 |
| Q99K23 | Ufm1-specific protease 2 OS=Mus musculus OX=10090 GN=Ufsp2 PE=1 SV=1 | Ufsp2 | 0.82 |
| P63001 | Ras-related C3 botulinum toxin substrate 1 OS=Mus musculus OX=10090 GN=Rac1 PE=1 SV=1 | Rac1 | 0.82 |
| Q61458 | Cyclin-H OS=Mus musculus OX=10090 GN=Ccnh PE=1 SV=2 | Ccnh | 0.82 |
| Q91WK7 | Ankyrin repeat domain-containing protein 54 OS=Mus musculus OX=10090 GN=Ankrd54 PE=1 SV=1 | Ankrd54 | 0.82 |
| Q921W0 | Charged multivesicular body protein 1a OS=Mus musculus OX=10090 GN=Chmp1a PE=1 SV=1 | Chmp1a | 0.82 |
| Q8R1F5 | Putative hydroxypyruvate isomerase OS=Mus musculus OX=10090 GN=Hyi PE=1 SV=2 | Hyi | 0.82 |
| P70372 | ELAV-like protein 1 OS=Mus musculus OX=10090 GN=Elavl1 PE=1 SV=2 | Elavl1 | 0.82 |
| P21107 | Tropomyosin alpha-3 chain OS=Mus musculus OX=10090 GN=Tpm3 PE=1 SV=3 | Tpm3 | 0.82 |
| Q8BYM8 | Probable cysteine--tRNA ligase, mitochondrial OS=Mus musculus OX=10090 GN=Cars2 PE=1 SV=2 | Cars2 | 0.82 |
| Q6Q899 | Antiviral innate immune response receptor RIG-I OS=Mus musculus OX=10090 GN=Ddx58 PE=1 SV=2 | Ddx58 | 0.82 |
| Q8C4S8 | DENN domain-containing protein 2A OS=Mus musculus OX=10090 GN=Dennd2a PE=1 SV=1 | Dennd2a | 0.82 |
| Q60931 | Voltage-dependent anion-selective channel protein 3 OS=Mus musculus OX=10090 GN=Vdac3 PE=1 SV=1 | Vdac3 | 0.82 |
| Q3U186 | Probable arginine--tRNA ligase, mitochondrial OS=Mus musculus OX=10090 GN=Rars2 PE=1 SV=1 | Rars2 | 0.82 |
| Q9ER73 | Elongator complex protein 4 OS=Mus musculus OX=10090 GN=Elp4 PE=1 SV=2 | Elp4 | 0.82 |
| Q9DCW4 | Electron transfer flavoprotein subunit beta OS=Mus musculus OX=10090 GN=Etfb PE=1 SV=3 | Etfb | 0.82 |
| Q9ERN0 | Secretory carrier-associated membrane protein 2 OS=Mus musculus OX=10090 GN=Scamp2 PE=1 SV=1 | Scamp2 | 0.82 |
| Q64523 | Histone H2A type 2-C OS=Mus musculus OX=10090 GN=H2ac20 PE=1 SV=3 | H2ac20 | 0.82 |
| O35381 | Acidic leucine-rich nuclear phosphoprotein 32 family member A OS=Mus musculus OX=10090 GN=Anp32a PE=1 SV=1 | Anp32a | 0.82 |
| Q3TZZ7 | Extended synaptotagmin-2 OS=Mus musculus OX=10090 GN=Esyt2 PE=1 SV=1 | Esyt2 | 0.82 |
| Q8VE22 | 28S ribosomal protein S23, mitochondrial OS=Mus musculus OX=10090 GN=Mrps23 PE=1 SV=1 | Mrps23 | 0.82 |
| Q9CQJ2 | PIH1 domain-containing protein 1 OS=Mus musculus OX=10090 GN=Pih1d1 PE=1 SV=1 | Pih1d1 | 0.82 |
| F7BJB9 | MORC family CW-type zinc finger protein 3 OS=Mus musculus OX=10090 GN=Morc3 PE=1 SV=1 | Morc3 | 0.83 |
| P53994 | Ras-related protein Rab-2A OS=Mus musculus OX=10090 GN=Rab2a PE=1 SV=1 | Rab2a | 0.83 |
| Q9CXY6 | Interleukin enhancer-binding factor 2 OS=Mus musculus OX=10090 GN=Ilf2 PE=1 SV=1 | Ilf2 | 0.83 |
| Q7TNE3 | Sperm-associated antigen 7 OS=Mus musculus OX=10090 GN=Spag7 PE=1 SV=1 | Spag7 | 0.83 |
| Q8C863 | E3 ubiquitin-protein ligase Itchy OS=Mus musculus OX=10090 GN=Itch PE=1 SV=2 | Itch | 0.83 |
| O70422 | General transcription factor IIH subunit 4 OS=Mus musculus OX=10090 GN=Gtf2h4 PE=1 SV=1 | Gtf2h4 | 0.83 |
| Q7TMF2 | 3'-5' exoribonuclease 1 OS=Mus musculus OX=10090 GN=Eri1 PE=1 SV=2 | Eri1 | 0.83 |
| P22518 | Dual specificity protein kinase CLK1 OS=Mus musculus OX=10090 GN=Clk1 PE=1 SV=2 | Clk1 | 0.83 |
| Q80VY9 | ATP-dependent RNA helicase DHX33 OS=Mus musculus OX=10090 GN=Dhx33 PE=1 SV=1 | Dhx33 | 0.83 |
| D3Z6Q9 | Bridging integrator 2 OS=Mus musculus OX=10090 GN=Bin2 PE=1 SV=1 | Bin2 | 0.83 |
| P04441 | H-2 class II histocompatibility antigen gamma chain OS=Mus musculus OX=10090 GN=Cd74 PE=1 SV=3 | Cd74 | 0.83 |
| Q8BH57 | WD repeat-containing protein 48 OS=Mus musculus OX=10090 GN=Wdr48 PE=1 SV=1 | Wdr48 | 0.83 |
| Q61749 | Translation initiation factor eIF-2B subunit delta OS=Mus musculus OX=10090 GN=Eif2b4 PE=1 SV=2 | Eif2b4 | 0.83 |
| Q9ER38 | Torsin-3A OS=Mus musculus OX=10090 GN=Tor3a PE=1 SV=2 | Tor3a | 0.83 |
| Q9DB42 | Zinc finger protein 593 OS=Mus musculus OX=10090 GN=Znf593 PE=1 SV=2 | Znf593 | 0.83 |
| O55128 | Histone deacetylase complex subunit SAP18 OS=Mus musculus OX=10090 GN=Sap18 PE=1 SV=1 | Sap18 | 0.83 |
| Q8BHG9 | CGG triplet repeat-binding protein 1 OS=Mus musculus OX=10090 GN=Cggbp1 PE=1 SV=1 | Cggbp1 | 0.83 |
| P49138 | MAP kinase-activated protein kinase 2 OS=Mus musculus OX=10090 GN=Mapkapk2 PE=1 SV=2 | Mapkapk2 | 0.83 |
| Q9DCJ9 | N-acetylneuraminate lyase OS=Mus musculus OX=10090 GN=Npl PE=1 SV=1 | Npl | 0.83 |
| Q8VDD9 | PH-interacting protein OS=Mus musculus OX=10090 GN=Phip PE=1 SV=2 | Phip | 0.83 |
| Q9ER69 | Pre-mRNA-splicing regulator WTAP OS=Mus musculus OX=10090 GN=Wtap PE=1 SV=3 | Wtap | 0.83 |
| Q9DBZ5 | Eukaryotic translation initiation factor 3 subunit K OS=Mus musculus OX=10090 GN=Eif3k PE=1 SV=1 | Eif3k | 0.83 |
| Q8C0D0 | Probable tRNA pseudouridine synthase 1 OS=Mus musculus OX=10090 GN=Trub1 PE=1 SV=1 | Trub1 | 0.83 |
| O35309 | N-myc-interactor OS=Mus musculus OX=10090 GN=Nmi PE=1 SV=1 | Nmi | 0.83 |
| P20108 | Thioredoxin-dependent peroxide reductase, mitochondrial OS=Mus musculus OX=10090 GN=Prdx3 PE=1 SV=1 | Prdx3 | 0.83 |
| Q923G2 | DNA-directed RNA polymerases I, II, and III subunit RPABC3 OS=Mus musculus OX=10090 GN=Polr2h PE=1 SV=3 | Polr2h | 0.83 |
| Q8R0K4 | Coiled-coil domain-containing protein 137 OS=Mus musculus OX=10090 GN=Ccdc137 PE=2 SV=1 | Ccdc137 | 0.83 |
| Q9JKV7 | Exostosin-like 1 OS=Mus musculus OX=10090 GN=Extl1 PE=2 SV=2 | Extl1 | 0.83 |
| P55258 | Ras-related protein Rab-8A OS=Mus musculus OX=10090 GN=Rab8a PE=1 SV=2 | Rab8a | 0.83 |
| Q91V81 | RNA-binding protein 42 OS=Mus musculus OX=10090 GN=Rbm42 PE=1 SV=2 | Rbm42 | 0.83 |
| Q7TQH0 | Ataxin-2-like protein OS=Mus musculus OX=10090 GN=Atxn2l PE=1 SV=1 | Atxn2l | 0.83 |
| Q9JI75 | Ribosyldihydronicotinamide dehydrogenase [quinone] OS=Mus musculus OX=10090 GN=Nqo2 PE=1 SV=3 | Nqo2 | 0.83 |
| Q80ZV0 | Ribonuclease H2 subunit B OS=Mus musculus OX=10090 GN=Rnaseh2b PE=1 SV=2 | Rnaseh2b | 0.83 |
| P32233 | Developmentally-regulated GTP-binding protein 1 OS=Mus musculus OX=10090 GN=Drg1 PE=1 SV=1 | Drg1 | 0.83 |
| Q9QXY9 | Peroxisomal biogenesis factor 3 OS=Mus musculus OX=10090 GN=Pex3 PE=1 SV=1 | Pex3 | 0.83 |
| Q9JJA4 | Ribosome biogenesis protein WDR12 OS=Mus musculus OX=10090 GN=Wdr12 PE=1 SV=1 | Wdr12 | 0.83 |
| Q9CWX4 | Mitochondrial RNA pseudouridine synthase Rpusd4 OS=Mus musculus OX=10090 GN=Rpusd4 PE=2 SV=1 | Rpusd4 | 0.83 |
| Q9D6Z1 | Nucleolar protein 56 OS=Mus musculus OX=10090 GN=Nop56 PE=1 SV=2 | Nop56 | 0.83 |
| P13864 | DNA (cytosine-5)-methyltransferase 1 OS=Mus musculus OX=10090 GN=Dnmt1 PE=1 SV=5 | Dnmt1 | 0.83 |
| Q9CYU6 | Diphthine methyltransferase OS=Mus musculus OX=10090 GN=Dph7 PE=2 SV=1 | Dph7 | 0.83 |
| Q9CX66 | NOP protein chaperone 1 OS=Mus musculus OX=10090 GN=Nopchap1 PE=1 SV=1 | Nopchap1 | 0.83 |
| Q8CH77 | Neuron navigator 1 OS=Mus musculus OX=10090 GN=Nav1 PE=1 SV=2 | Nav1 | 0.83 |
| Q9D2V8 | Major facilitator superfamily domain-containing protein 10 OS=Mus musculus OX=10090 GN=Mfsd10 PE=1 SV=1 | Mfsd10 | 0.83 |
| Q8K0D5 | Elongation factor G, mitochondrial OS=Mus musculus OX=10090 GN=Gfm1 PE=1 SV=1 | Gfm1 | 0.83 |
| Q64343 | ATP-binding cassette sub-family G member 1 OS=Mus musculus OX=10090 GN=Abcg1 PE=1 SV=1 | Abcg1 | 0.83 |
| A2BE28 | Ribosomal biogenesis protein LAS1L OS=Mus musculus OX=10090 GN=Las1l PE=1 SV=1 | Las1l | 0.83 |
| Q9DC53 | Copine-8 OS=Mus musculus OX=10090 GN=Cpne8 PE=2 SV=3 | Cpne8 | 0.83 |
| P53395 | Lipoamide acyltransferase component of branched-chain alpha-keto acid dehydrogenase complex, mitochondrial OS=Mus musculus OX=10090 GN=Dbt PE=1 SV=2 | Dbt | 0.83 |
| Q8K021 | Secretory carrier-associated membrane protein 1 OS=Mus musculus OX=10090 GN=Scamp1 PE=1 SV=1 | Scamp1 | 0.83 |
| Q8K199 | COX assembly mitochondrial protein 2 homolog OS=Mus musculus OX=10090 GN=Cmc2 PE=3 SV=1 | Cmc2 | 0.83 |
| Q61029 | Lamina-associated polypeptide 2, isoforms beta/delta/epsilon/gamma OS=Mus musculus OX=10090 GN=Tmpo PE=1 SV=4 | Tmpo | 0.83 |
| Q9CRD2 | ER membrane protein complex subunit 2 OS=Mus musculus OX=10090 GN=Emc2 PE=1 SV=1 | Emc2 | 0.83 |
| Q8K078 | Solute carrier organic anion transporter family member 4A1 OS=Mus musculus OX=10090 GN=Slco4a1 PE=1 SV=2 | Slco4a1 | 0.83 |
| P62073 | Mitochondrial import inner membrane translocase subunit Tim10 OS=Mus musculus OX=10090 GN=Timm10 PE=1 SV=1 | Timm10 | 0.83 |
| Q99N94 | 39S ribosomal protein L9, mitochondrial OS=Mus musculus OX=10090 GN=Mrpl9 PE=1 SV=2 | Mrpl9 | 0.83 |
| F6ZDS4 | Nucleoprotein TPR OS=Mus musculus OX=10090 GN=Tpr PE=1 SV=1 | Tpr | 0.83 |
| Q8BGS0 | Protein MAK16 homolog OS=Mus musculus OX=10090 GN=Mak16 PE=1 SV=1 | Mak16 | 0.83 |
| Q9QYJ0 | DnaJ homolog subfamily A member 2 OS=Mus musculus OX=10090 GN=Dnaja2 PE=1 SV=1 | Dnaja2 | 0.83 |
| Q9D3E6 | Cohesin subunit SA-1 OS=Mus musculus OX=10090 GN=Stag1 PE=1 SV=3 | Stag1 | 0.83 |
| Q8CI95 | Oxysterol-binding protein-related protein 11 OS=Mus musculus OX=10090 GN=Osbpl11 PE=1 SV=2 | Osbpl11 | 0.83 |
| E9Q7G0 | Nuclear mitotic apparatus protein 1 OS=Mus musculus OX=10090 GN=Numa1 PE=1 SV=1 | Numa1 | 0.83 |
| O09159 | Lysosomal alpha-mannosidase OS=Mus musculus OX=10090 GN=Man2b1 PE=1 SV=4 | Man2b1 | 0.83 |
| P35293 | Ras-related protein Rab-18 OS=Mus musculus OX=10090 GN=Rab18 PE=1 SV=2 | Rab18 | 0.83 |
| Q9CXI0 | 2-methoxy-6-polyprenyl-1,4-benzoquinol methylase, mitochondrial OS=Mus musculus OX=10090 GN=Coq5 PE=1 SV=2 | Coq5 | 0.83 |
| Q8CD26 | Solute carrier family 35 member E1 OS=Mus musculus OX=10090 GN=Slc35e1 PE=1 SV=2 | Slc35e1 | 0.83 |
| P82343 | N-acylglucosamine 2-epimerase OS=Mus musculus OX=10090 GN=Renbp PE=1 SV=3 | Renbp | 0.83 |
| Q80WJ7 | Protein LYRIC OS=Mus musculus OX=10090 GN=Mtdh PE=1 SV=1 | Mtdh | 0.83 |
| Q9R0E1 | Multifunctional procollagen lysine hydroxylase and glycosyltransferase LH3 OS=Mus musculus OX=10090 GN=Plod3 PE=1 SV=1 | Plod3 | 0.83 |
| Q7TMV3 | FAST kinase domain-containing protein 5, mitochondrial OS=Mus musculus OX=10090 GN=Fastkd5 PE=2 SV=1 | Fastkd5 | 0.83 |
| Q505D7 | Optic atrophy 3 protein homolog OS=Mus musculus OX=10090 GN=Opa3 PE=1 SV=1 | Opa3 | 0.83 |
| Q8K2M0 | 39S ribosomal protein L38, mitochondrial OS=Mus musculus OX=10090 GN=Mrpl38 PE=1 SV=2 | Mrpl38 | 0.83 |
| Q6PAR5 | GTPase-activating protein and VPS9 domain-containing protein 1 OS=Mus musculus OX=10090 GN=Gapvd1 PE=1 SV=2 | Gapvd1 | 0.83 |
| Q07813 | Apoptosis regulator BAX OS=Mus musculus OX=10090 GN=Bax PE=1 SV=1 | Bax | 0.83 |
| Q80VL1 | Tudor and KH domain-containing protein OS=Mus musculus OX=10090 GN=Tdrkh PE=1 SV=1 | Tdrkh | 0.83 |
| Q6IRU5 | Clathrin light chain B OS=Mus musculus OX=10090 GN=Cltb PE=1 SV=1 | Cltb | 0.83 |
| Q91YR1 | Twinfilin-1 OS=Mus musculus OX=10090 GN=Twf1 PE=1 SV=2 | Twf1 | 0.83 |
| Q921T2 | Torsin-1A-interacting protein 1 OS=Mus musculus OX=10090 GN=Tor1aip1 PE=1 SV=3 | Tor1aip1 | 0.83 |
| Q91VT1 | E3 SUMO-protein ligase NSE2 OS=Mus musculus OX=10090 GN=Nsmce2 PE=2 SV=1 | Nsmce2 | 0.84 |
| Q9ER39 | Torsin-1A OS=Mus musculus OX=10090 GN=Tor1a PE=1 SV=1 | Tor1a | 0.84 |
| Q8BG81 | Polymerase delta-interacting protein 3 OS=Mus musculus OX=10090 GN=Poldip3 PE=1 SV=1 | Poldip3 | 0.84 |
| Q6ZQH8 | Nucleoporin NUP188 OS=Mus musculus OX=10090 GN=Nup188 PE=1 SV=2 | Nup188 | 0.84 |
| Q8BFZ3 | Beta-actin-like protein 2 OS=Mus musculus OX=10090 GN=Actbl2 PE=1 SV=1 | Actbl2 | 0.84 |
| P70403 | Protein CASP OS=Mus musculus OX=10090 GN=Cux1 PE=1 SV=2 | Cux1 | 0.84 |
| Q8BJ90 | Zinc finger protein 771 OS=Mus musculus OX=10090 GN=Znf771 PE=2 SV=1 | Znf771 | 0.84 |
| Q921G7 | Electron transfer flavoprotein-ubiquinone oxidoreductase, mitochondrial OS=Mus musculus OX=10090 GN=Etfdh PE=1 SV=1 | Etfdh | 0.84 |
| Q8CEC6 | Peptidylprolyl isomerase domain and WD repeat-containing protein 1 OS=Mus musculus OX=10090 GN=Ppwd1 PE=1 SV=2 | Ppwd1 | 0.84 |
| Q8BG05 | Heterogeneous nuclear ribonucleoprotein A3 OS=Mus musculus OX=10090 GN=Hnrnpa3 PE=1 SV=1 | Hnrnpa3 | 0.84 |
| Q9Z2U0 | Proteasome subunit alpha type-7 OS=Mus musculus OX=10090 GN=Psma7 PE=1 SV=1 | Psma7 | 0.84 |
| P70353 | Nuclear transcription factor Y subunit gamma OS=Mus musculus OX=10090 GN=Nfyc PE=1 SV=2 | Nfyc | 0.84 |
| Q9D4J7 | PHD finger protein 6 OS=Mus musculus OX=10090 GN=Phf6 PE=1 SV=1 | Phf6 | 0.84 |
| Q9QUJ7 | Long-chain-fatty-acid--CoA ligase 4 OS=Mus musculus OX=10090 GN=Acsl4 PE=1 SV=2 | Acsl4 | 0.84 |
| Q78IK4 | MICOS complex subunit Mic27 OS=Mus musculus OX=10090 GN=Apool PE=1 SV=1 | Apool | 0.84 |
| Q9R0A0 | Peroxisomal membrane protein PEX14 OS=Mus musculus OX=10090 GN=Pex14 PE=1 SV=1 | Pex14 | 0.84 |
| Q0VEJ0 | Centrosomal protein of 76 kDa OS=Mus musculus OX=10090 GN=Cep76 PE=1 SV=1 | Cep76 | 0.84 |
| E9Q5C9 | Nucleolar and coiled-body phosphoprotein 1 OS=Mus musculus OX=10090 GN=Nolc1 PE=1 SV=1 | Nolc1 | 0.84 |
| Q9DBY8 | Nuclear valosin-containing protein-like OS=Mus musculus OX=10090 GN=Nvl PE=1 SV=1 | Nvl | 0.84 |
| P10605 | Cathepsin B OS=Mus musculus OX=10090 GN=Ctsb PE=1 SV=2 | Ctsb | 0.84 |
| Q3UW53 | Protein Niban 1 OS=Mus musculus OX=10090 GN=Niban1 PE=1 SV=2 | Niban1 | 0.84 |
| Q9CQ10 | Charged multivesicular body protein 3 OS=Mus musculus OX=10090 GN=Chmp3 PE=1 SV=3 | Chmp3 | 0.84 |
| Q3TDX8 | Cytochrome b5 reductase 4 OS=Mus musculus OX=10090 GN=Cyb5r4 PE=2 SV=3 | Cyb5r4 | 0.84 |
| E9Q5F9 | Histone-lysine N-methyltransferase SETD2 OS=Mus musculus OX=10090 GN=Setd2 PE=1 SV=1 | Setd2 | 0.84 |
| Q501J2 | Adenine nucleotide translocase lysine N-methyltransferase OS=Mus musculus OX=10090 GN=Antkmt PE=1 SV=1 | Antkmt | 0.84 |
| Q9Z268 | RasGAP-activating-like protein 1 OS=Mus musculus OX=10090 GN=Rasal1 PE=1 SV=2 | Rasal1 | 0.84 |
| Q6DFV5 | Probable helicase with zinc finger domain OS=Mus musculus OX=10090 GN=Helz PE=1 SV=2 | Helz | 0.84 |
| P59017 | Bcl-2-like protein 13 OS=Mus musculus OX=10090 GN=Bcl2l13 PE=1 SV=2 | Bcl2l13 | 0.84 |
| Q80VD1 | Protein FAM98B OS=Mus musculus OX=10090 GN=Fam98b PE=1 SV=1 | Fam98b | 0.84 |
| Q9CXR1 | Dehydrogenase/reductase SDR family member 7 OS=Mus musculus OX=10090 GN=Dhrs7 PE=1 SV=2 | Dhrs7 | 0.84 |
| Q3TQB2 | FAD-dependent oxidoreductase domain-containing protein 1 OS=Mus musculus OX=10090 GN=Foxred1 PE=1 SV=1 | Foxred1 | 0.84 |
| Q9D7P6 | Iron-sulfur cluster assembly enzyme ISCU, mitochondrial OS=Mus musculus OX=10090 GN=Iscu PE=1 SV=1 | Iscu | 0.84 |
| Q3UZA1 | CapZ-interacting protein OS=Mus musculus OX=10090 GN=Rcsd1 PE=1 SV=1 | Rcsd1 | 0.84 |
| Q8C5K5 | Uncharacterized protein CXorf38 homolog OS=Mus musculus OX=10090 PE=1 SV=1 | -- | 0.84 |
| Q8K0C1 | Importin-13 OS=Mus musculus OX=10090 GN=Ipo13 PE=1 SV=1 | Ipo13 | 0.84 |
| P35601 | Replication factor C subunit 1 OS=Mus musculus OX=10090 GN=Rfc1 PE=1 SV=2 | Rfc1 | 0.84 |
| A2A6Q5 | Cell division cycle protein 27 homolog OS=Mus musculus OX=10090 GN=Cdc27 PE=1 SV=1 | Cdc27 | 0.84 |
| P48962 | ADP/ATP translocase 1 OS=Mus musculus OX=10090 GN=Slc25a4 PE=1 SV=4 | Slc25a4 | 0.84 |
| A2A791 | Zinc finger MYM-type protein 4 OS=Mus musculus OX=10090 GN=Zmym4 PE=1 SV=1 | Zmym4 | 0.84 |
| Q9QYF1 | Retinol dehydrogenase 11 OS=Mus musculus OX=10090 GN=Rdh11 PE=1 SV=2 | Rdh11 | 0.84 |
| Q8R2M2 | Deoxynucleotidyltransferase terminal-interacting protein 2 OS=Mus musculus OX=10090 GN=Dnttip2 PE=1 SV=1 | Dnttip2 | 0.84 |
| P97760 | DNA-directed RNA polymerase II subunit RPB3 OS=Mus musculus OX=10090 GN=Polr2c PE=1 SV=2 | Polr2c | 0.84 |
| Q9D864 | Actin-related protein 6 OS=Mus musculus OX=10090 GN=Actr6 PE=1 SV=2 | Actr6 | 0.84 |
| Q8WTY4 | Anamorsin OS=Mus musculus OX=10090 GN=Ciapin1 PE=1 SV=1 | Ciapin1 | 0.84 |
| Q52KE7 | Cyclin-L1 OS=Mus musculus OX=10090 GN=Ccnl1 PE=1 SV=1 | Ccnl1 | 0.84 |
| Q9D6J1 | Ceramide synthase 4 OS=Mus musculus OX=10090 GN=Cers4 PE=1 SV=1 | Cers4 | 0.84 |
| Q922E4 | Ethanolamine-phosphate cytidylyltransferase OS=Mus musculus OX=10090 GN=Pcyt2 PE=1 SV=1 | Pcyt2 | 0.84 |
| Q5SUF2 | Luc7-like protein 3 OS=Mus musculus OX=10090 GN=Luc7l3 PE=1 SV=1 | Luc7l3 | 0.84 |
| Q03267 | DNA-binding protein Ikaros OS=Mus musculus OX=10090 GN=Ikzf1 PE=1 SV=2 | Ikzf1 | 0.84 |
| E9Q394 | A-kinase anchor protein 13 OS=Mus musculus OX=10090 GN=Akap13 PE=1 SV=1 | Akap13 | 0.84 |
| Q9CY52 | Probable tRNA(His) guanylyltransferase OS=Mus musculus OX=10090 GN=Thg1l PE=1 SV=1 | Thg1l | 0.84 |
| Q9Z1M0 | P2X purinoceptor 7 OS=Mus musculus OX=10090 GN=P2rx7 PE=1 SV=2 | P2rx7 | 0.84 |
| Q8K2Z2 | Pre-mRNA-processing factor 39 OS=Mus musculus OX=10090 GN=Prpf39 PE=1 SV=3 | Prpf39 | 0.84 |
| O55028 | [3-methyl-2-oxobutanoate dehydrogenase [lipoamide]] kinase, mitochondrial OS=Mus musculus OX=10090 GN=Bckdk PE=1 SV=1 | Bckdk | 0.84 |
| Q80ZW2 | Protein THEM6 OS=Mus musculus OX=10090 GN=Them6 PE=1 SV=1 | Them6 | 0.84 |
| Q569Z6 | Thyroid hormone receptor-associated protein 3 OS=Mus musculus OX=10090 GN=Thrap3 PE=1 SV=1 | Thrap3 | 0.84 |
| Q9D3U0 | tRNA pseudouridine synthase Pus10 OS=Mus musculus OX=10090 GN=Pus10 PE=1 SV=1 | Pus10 | 0.84 |
| Q8CBW3 | Abl interactor 1 OS=Mus musculus OX=10090 GN=Abi1 PE=1 SV=3 | Abi1 | 0.84 |
| Q9QVP9 | Protein-tyrosine kinase 2-beta OS=Mus musculus OX=10090 GN=Ptk2b PE=1 SV=2 | Ptk2b | 0.84 |
| Q925J9 | Mediator of RNA polymerase II transcription subunit 1 OS=Mus musculus OX=10090 GN=Med1 PE=1 SV=2 | Med1 | 0.84 |
| Q91V09 | WD repeat-containing protein 13 OS=Mus musculus OX=10090 GN=Wdr13 PE=1 SV=1 | Wdr13 | 0.84 |
| Q9CQ69 | Cytochrome b-c1 complex subunit 8 OS=Mus musculus OX=10090 GN=Uqcrq PE=1 SV=3 | Uqcrq | 0.84 |
| Q99KJ8 | Dynactin subunit 2 OS=Mus musculus OX=10090 GN=Dctn2 PE=1 SV=3 | Dctn2 | 0.84 |
| Q6ZQM8 | UDP-glucuronosyltransferase 1A7 OS=Mus musculus OX=10090 GN=Ugt1a7 PE=1 SV=1 | Ugt1a7 | 0.84 |
| Q99J09 | Methylosome protein 50 OS=Mus musculus OX=10090 GN=Wdr77 PE=1 SV=1 | Wdr77 | 0.84 |
| Q924Z4 | Ceramide synthase 2 OS=Mus musculus OX=10090 GN=Cers2 PE=1 SV=1 | Cers2 | 0.84 |
| Q9D8S9 | BolA-like protein 1 OS=Mus musculus OX=10090 GN=Bola1 PE=1 SV=1 | Bola1 | 0.84 |
| O08810 | 116 kDa U5 small nuclear ribonucleoprotein component OS=Mus musculus OX=10090 GN=Eftud2 PE=1 SV=1 | Eftud2 | 0.84 |
| P47963 | 60S ribosomal protein L13 OS=Mus musculus OX=10090 GN=Rpl13 PE=1 SV=3 | Rpl13 | 0.84 |
| P97930 | Thymidylate kinase OS=Mus musculus OX=10090 GN=Dtymk PE=1 SV=2 | Dtymk | 0.84 |
| Q99MR3 | Solute carrier family 12 member 9 OS=Mus musculus OX=10090 GN=Slc12a9 PE=1 SV=2 | Slc12a9 | 0.84 |
| Q9D0M3 | Cytochrome c1, heme protein, mitochondrial OS=Mus musculus OX=10090 GN=Cyc1 PE=1 SV=1 | Cyc1 | 0.85 |
| Q9WUR9 | Adenylate kinase 4, mitochondrial OS=Mus musculus OX=10090 GN=Ak4 PE=1 SV=1 | Ak4 | 0.85 |
| Q9DCD0 | 6-phosphogluconate dehydrogenase, decarboxylating OS=Mus musculus OX=10090 GN=Pgd PE=1 SV=3 | Pgd | 0.85 |
| Q61609 | Sodium-dependent phosphate transporter 1 OS=Mus musculus OX=10090 GN=Slc20a1 PE=1 SV=1 | Slc20a1 | 0.85 |
| P68368 | Tubulin alpha-4A chain OS=Mus musculus OX=10090 GN=Tuba4a PE=1 SV=1 | Tuba4a | 0.85 |
| P35279 | Ras-related protein Rab-6A OS=Mus musculus OX=10090 GN=Rab6a PE=1 SV=4 | Rab6a | 0.85 |
| E9PVX6 | Proliferation marker protein Ki-67 OS=Mus musculus OX=10090 GN=Mki67 PE=1 SV=1 | Mki67 | 0.85 |
| Q9CY34 | NEDD8-conjugating enzyme UBE2F OS=Mus musculus OX=10090 GN=Ube2f PE=1 SV=1 | Ube2f | 0.85 |
| Q9DC51 | Guanine nucleotide-binding protein G(i) subunit alpha-3 OS=Mus musculus OX=10090 GN=Gnai3 PE=1 SV=3 | Gnai3 | 0.85 |
| Q8K3W0 | BRISC and BRCA1-A complex member 2 OS=Mus musculus OX=10090 GN=Babam2 PE=1 SV=2 | Babam2 | 0.85 |
| Q6PGH2 | Jupiter microtubule associated homolog 2 OS=Mus musculus OX=10090 GN=Jpt2 PE=1 SV=1 | Jpt2 | 0.85 |
| Q922Q8 | Leucine-rich repeat-containing protein 59 OS=Mus musculus OX=10090 GN=Lrrc59 PE=1 SV=1 | Lrrc59 | 0.85 |
| Q640N3 | Rho GTPase-activating protein 30 OS=Mus musculus OX=10090 GN=Arhgap30 PE=1 SV=3 | Arhgap30 | 0.85 |
| Q9JJZ4 | Ubiquitin-conjugating enzyme E2 J1 OS=Mus musculus OX=10090 GN=Ube2j1 PE=1 SV=2 | Ube2j1 | 0.85 |
| P63005 | Platelet-activating factor acetylhydrolase IB subunit beta OS=Mus musculus OX=10090 GN=Pafah1b1 PE=1 SV=2 | Pafah1b1 | 0.85 |
| Q8K387 | Ubiquitin carboxyl-terminal hydrolase 45 OS=Mus musculus OX=10090 GN=Usp45 PE=1 SV=1 | Usp45 | 0.85 |
| Q3UCQ1 | Forkhead box protein K2 OS=Mus musculus OX=10090 GN=Foxk2 PE=1 SV=3 | Foxk2 | 0.85 |
| O70475 | UDP-glucose 6-dehydrogenase OS=Mus musculus OX=10090 GN=Ugdh PE=1 SV=1 | Ugdh | 0.85 |
| Q99LC5 | Electron transfer flavoprotein subunit alpha, mitochondrial OS=Mus musculus OX=10090 GN=Etfa PE=1 SV=2 | Etfa | 0.85 |
| Q9D6N1 | Carbonic anhydrase 13 OS=Mus musculus OX=10090 GN=Ca13 PE=1 SV=1 | Ca13 | 0.85 |
| Q62084 | Protein phosphatase 1 regulatory subunit 14B OS=Mus musculus OX=10090 GN=Ppp1r14b PE=1 SV=2 | Ppp1r14b | 0.85 |
| Q9ET30 | Transmembrane 9 superfamily member 3 OS=Mus musculus OX=10090 GN=Tm9sf3 PE=1 SV=1 | Tm9sf3 | 0.85 |
| Q9JIX0 | Transcription and mRNA export factor ENY2 OS=Mus musculus OX=10090 GN=Eny2 PE=1 SV=1 | Eny2 | 0.85 |
| Q91YT0 | NADH dehydrogenase [ubiquinone] flavoprotein 1, mitochondrial OS=Mus musculus OX=10090 GN=Ndufv1 PE=1 SV=1 | Ndufv1 | 0.85 |
| Q3UJB9 | Enhancer of mRNA-decapping protein 4 OS=Mus musculus OX=10090 GN=Edc4 PE=1 SV=2 | Edc4 | 0.85 |
| Q8BX57 | PX domain-containing protein kinase-like protein OS=Mus musculus OX=10090 GN=Pxk PE=1 SV=2 | Pxk | 0.85 |
| Q9DBA9 | General transcription factor IIH subunit 1 OS=Mus musculus OX=10090 GN=Gtf2h1 PE=1 SV=2 | Gtf2h1 | 0.85 |
| O09131 | Glutathione S-transferase omega-1 OS=Mus musculus OX=10090 GN=Gsto1 PE=1 SV=2 | Gsto1 | 0.85 |
| Q9D0R4 | Probable ATP-dependent RNA helicase DDX56 OS=Mus musculus OX=10090 GN=Ddx56 PE=2 SV=1 | Ddx56 | 0.85 |
| Q811D0 | Disks large homolog 1 OS=Mus musculus OX=10090 GN=Dlg1 PE=1 SV=1 | Dlg1 | 0.85 |
| P97496 | SWI/SNF complex subunit SMARCC1 OS=Mus musculus OX=10090 GN=Smarcc1 PE=1 SV=2 | Smarcc1 | 0.85 |
| P57776 | Elongation factor 1-delta OS=Mus musculus OX=10090 GN=Eef1d PE=1 SV=3 | Eef1d | 0.85 |
| Q80Y20 | Alkylated DNA repair protein alkB homolog 8 OS=Mus musculus OX=10090 GN=Alkbh8 PE=1 SV=1 | Alkbh8 | 0.85 |
| Q9JJL8 | Serine--tRNA ligase, mitochondrial OS=Mus musculus OX=10090 GN=Sars2 PE=1 SV=2 | Sars2 | 0.85 |
| Q8K2Q7 | BRO1 domain-containing protein BROX OS=Mus musculus OX=10090 GN=Brox PE=1 SV=1 | Brox | 0.85 |
| P11835 | Integrin beta-2 OS=Mus musculus OX=10090 GN=Itgb2 PE=1 SV=2 | Itgb2 | 0.85 |
| Q8BFQ4 | WD repeat-containing protein 82 OS=Mus musculus OX=10090 GN=Wdr82 PE=1 SV=1 | Wdr82 | 0.85 |
| Q99LG0 | Ubiquitin carboxyl-terminal hydrolase 16 OS=Mus musculus OX=10090 GN=Usp16 PE=1 SV=2 | Usp16 | 0.85 |
| Q8R3Y5 | Uncharacterized protein C19orf47 homolog OS=Mus musculus OX=10090 PE=1 SV=2 | -- | 0.85 |
| Q8BXA5 | Cleft lip and palate transmembrane protein 1-like protein OS=Mus musculus OX=10090 GN=Clptm1l PE=1 SV=1 | Clptm1l | 0.85 |
| P54923 | [Protein ADP-ribosylarginine] hydrolase OS=Mus musculus OX=10090 GN=Adprh PE=1 SV=1 | Adprh | 0.85 |
| Q3U5F4 | YrdC domain-containing protein, mitochondrial OS=Mus musculus OX=10090 GN=Yrdc PE=1 SV=1 | Yrdc | 0.85 |
| D0QMC3 | Myeloid cell nuclear differentiation antigen-like protein OS=Mus musculus OX=10090 GN=Mndal PE=1 SV=1 | Mndal | 0.85 |
| P50396 | Rab GDP dissociation inhibitor alpha OS=Mus musculus OX=10090 GN=Gdi1 PE=1 SV=3 | Gdi1 | 0.85 |
| Q9DCL9 | Multifunctional protein ADE2 OS=Mus musculus OX=10090 GN=Paics PE=1 SV=4 | Paics | 0.85 |
| P70362 | Ubiquitin recognition factor in ER-associated degradation protein 1 OS=Mus musculus OX=10090 GN=Ufd1 PE=1 SV=2 | Ufd1 | 0.85 |
| P12367 | cAMP-dependent protein kinase type II-alpha regulatory subunit OS=Mus musculus OX=10090 GN=Prkar2a PE=1 SV=2 | Prkar2a | 0.85 |
| Q9WV85 | Nucleoside diphosphate kinase 3 OS=Mus musculus OX=10090 GN=Nme3 PE=1 SV=3 | Nme3 | 0.85 |
| Q8BMS9 | Ras association domain-containing protein 2 OS=Mus musculus OX=10090 GN=Rassf2 PE=1 SV=1 | Rassf2 | 0.85 |
| Q62095 | ATP-dependent RNA helicase DDX3Y OS=Mus musculus OX=10090 GN=Ddx3y PE=1 SV=2 | Ddx3y | 0.85 |
| P61924 | Coatomer subunit zeta-1 OS=Mus musculus OX=10090 GN=Copz1 PE=1 SV=1 | Copz1 | 0.85 |
| Q9CXW2 | 28S ribosomal protein S22, mitochondrial OS=Mus musculus OX=10090 GN=Mrps22 PE=1 SV=1 | Mrps22 | 0.85 |
| Q922D4 | Serine/threonine-protein phosphatase 6 regulatory subunit 3 OS=Mus musculus OX=10090 GN=Ppp6r3 PE=1 SV=1 | Ppp6r3 | 0.85 |
| Q9D8X2 | Coiled-coil domain-containing protein 124 OS=Mus musculus OX=10090 GN=Ccdc124 PE=1 SV=1 | Ccdc124 | 0.85 |
| Q9CRD0 | OCIA domain-containing protein 1 OS=Mus musculus OX=10090 GN=Ociad1 PE=1 SV=1 | Ociad1 | 0.85 |
| Q9CY73 | 39S ribosomal protein L44, mitochondrial OS=Mus musculus OX=10090 GN=Mrpl44 PE=1 SV=3 | Mrpl44 | 0.85 |
| Q8BTS4 | Nuclear pore complex protein Nup54 OS=Mus musculus OX=10090 GN=Nup54 PE=1 SV=1 | Nup54 | 0.85 |
| Q60952 | Centrosome-associated protein CEP250 OS=Mus musculus OX=10090 GN=Cep250 PE=1 SV=4 | Cep250 | 0.85 |
| Q91YN0 | Protein C12orf4 homolog OS=Mus musculus OX=10090 GN=D6Wsu163e PE=1 SV=1 | D6Wsu163e | 0.85 |
| Q9JJ80 | Ribosome production factor 2 homolog OS=Mus musculus OX=10090 GN=Rpf2 PE=2 SV=2 | Rpf2 | 0.85 |
| Q9D8V7 | Signal peptidase complex catalytic subunit SEC11C OS=Mus musculus OX=10090 GN=Sec11c PE=1 SV=3 | Sec11c | 0.86 |
| P57784 | U2 small nuclear ribonucleoprotein A' OS=Mus musculus OX=10090 GN=Snrpa1 PE=1 SV=2 | Snrpa1 | 0.86 |
| P59470 | DNA-directed RNA polymerase III subunit RPC2 OS=Mus musculus OX=10090 GN=Polr3b PE=1 SV=2 | Polr3b | 0.86 |
| Q8BYA0 | Tubulin-specific chaperone D OS=Mus musculus OX=10090 GN=Tbcd PE=1 SV=1 | Tbcd | 0.86 |
| Q62203 | Splicing factor 3A subunit 2 OS=Mus musculus OX=10090 GN=Sf3a2 PE=1 SV=2 | Sf3a2 | 0.86 |
| Q9D483 | DNA-directed RNA polymerase III subunit RPC3 OS=Mus musculus OX=10090 GN=Polr3c PE=1 SV=1 | Polr3c | 0.86 |
| Q569Z5 | Probable ATP-dependent RNA helicase DDX46 OS=Mus musculus OX=10090 GN=Ddx46 PE=1 SV=2 | Ddx46 | 0.86 |
| P55194 | SH3 domain-binding protein 1 OS=Mus musculus OX=10090 GN=Sh3bp1 PE=1 SV=3 | Sh3bp1 | 0.86 |
| Q9CQN1 | Heat shock protein 75 kDa, mitochondrial OS=Mus musculus OX=10090 GN=Trap1 PE=1 SV=1 | Trap1 | 0.86 |
| Q80U72 | Protein scribble homolog OS=Mus musculus OX=10090 GN=Scrib PE=1 SV=2 | Scrib | 0.86 |
| Q923D5 | WW domain-binding protein 11 OS=Mus musculus OX=10090 GN=Wbp11 PE=1 SV=2 | Wbp11 | 0.86 |
| Q3URD3 | Sarcolemmal membrane-associated protein OS=Mus musculus OX=10090 GN=Slmap PE=1 SV=2 | Slmap | 0.86 |
| Q9D823 | 60S ribosomal protein L37 OS=Mus musculus OX=10090 GN=Rpl37 PE=1 SV=3 | Rpl37 | 0.86 |
| P12265 | Beta-glucuronidase OS=Mus musculus OX=10090 GN=Gusb PE=1 SV=2 | Gusb | 0.86 |
| Q99LY9 | NADH dehydrogenase [ubiquinone] iron-sulfur protein 5 OS=Mus musculus OX=10090 GN=Ndufs5 PE=1 SV=3 | Ndufs5 | 0.86 |
| Q8K2K6 | Arf-GAP domain and FG repeat-containing protein 1 OS=Mus musculus OX=10090 GN=Agfg1 PE=1 SV=2 | Agfg1 | 0.86 |
| Q80TJ7 | Histone lysine demethylase PHF8 OS=Mus musculus OX=10090 GN=Phf8 PE=1 SV=2 | Phf8 | 0.86 |
| Q99J47 | Dehydrogenase/reductase SDR family member 7B OS=Mus musculus OX=10090 GN=Dhrs7b PE=1 SV=1 | Dhrs7b | 0.86 |
| Q91YY4 | ATP synthase mitochondrial F1 complex assembly factor 2 OS=Mus musculus OX=10090 GN=Atpaf2 PE=1 SV=1 | Atpaf2 | 0.86 |
| Q8VHR5 | Transcriptional repressor p66-beta OS=Mus musculus OX=10090 GN=Gatad2b PE=1 SV=1 | Gatad2b | 0.86 |
| Q91W98 | Solute carrier family 15 member 4 OS=Mus musculus OX=10090 GN=Slc15a4 PE=1 SV=1 | Slc15a4 | 0.86 |
| Q7TMW6 | Cytosolic iron-sulfur assembly component 3 OS=Mus musculus OX=10090 GN=Ciao3 PE=1 SV=2 | Ciao3 | 0.86 |
| Q8BV13 | COP9 signalosome complex subunit 7b OS=Mus musculus OX=10090 GN=Cops7b PE=1 SV=1 | Cops7b | 0.86 |
| Q8BHC4 | Dephospho-CoA kinase domain-containing protein OS=Mus musculus OX=10090 GN=Dcakd PE=1 SV=1 | Dcakd | 0.86 |
| Q8BVI4 | Dihydropteridine reductase OS=Mus musculus OX=10090 GN=Qdpr PE=1 SV=2 | Qdpr | 0.86 |
| Q3THE2 | Myosin regulatory light chain 12B OS=Mus musculus OX=10090 GN=Myl12b PE=1 SV=2 | Myl12b | 0.86 |
| Q9EST3 | Eukaryotic translation initiation factor 4E transporter OS=Mus musculus OX=10090 GN=Eif4enif1 PE=1 SV=2 | Eif4enif1 | 0.86 |
| O70201 | Baculoviral IAP repeat-containing protein 5 OS=Mus musculus OX=10090 GN=Birc5 PE=1 SV=1 | Birc5 | 0.86 |
| Q921I2 | Kelch domain-containing protein 4 OS=Mus musculus OX=10090 GN=Klhdc4 PE=2 SV=2 | Klhdc4 | 0.86 |
| Q9D0M1 | Phosphoribosyl pyrophosphate synthase-associated protein 1 OS=Mus musculus OX=10090 GN=Prpsap1 PE=1 SV=1 | Prpsap1 | 0.86 |
| P51855 | Glutathione synthetase OS=Mus musculus OX=10090 GN=Gss PE=1 SV=1 | Gss | 0.86 |
| Q8BMK4 | Cytoskeleton-associated protein 4 OS=Mus musculus OX=10090 GN=Ckap4 PE=1 SV=2 | Ckap4 | 0.86 |
| Q9DBX2 | Phosducin-like protein OS=Mus musculus OX=10090 GN=Pdcl PE=1 SV=1 | Pdcl | 0.86 |
| Q06649 | SH3 domain-binding protein 2 OS=Mus musculus OX=10090 GN=Sh3bp2 PE=1 SV=1 | Sh3bp2 | 0.86 |
| P61161 | Actin-related protein 2 OS=Mus musculus OX=10090 GN=Actr2 PE=1 SV=1 | Actr2 | 0.86 |
| Q9CR89 | Endoplasmic reticulum-Golgi intermediate compartment protein 2 OS=Mus musculus OX=10090 GN=Ergic2 PE=1 SV=1 | Ergic2 | 0.86 |
| P41105 | 60S ribosomal protein L28 OS=Mus musculus OX=10090 GN=Rpl28 PE=1 SV=2 | Rpl28 | 0.86 |
| Q9CQ65 | S-methyl-5'-thioadenosine phosphorylase OS=Mus musculus OX=10090 GN=Mtap PE=1 SV=1 | Mtap | 0.86 |
| Q6ZPJ3 | (E3-independent) E2 ubiquitin-conjugating enzyme UBE2O OS=Mus musculus OX=10090 GN=Ube2o PE=1 SV=3 | Ube2o | 0.86 |
| Q07113 | Cation-independent mannose-6-phosphate receptor OS=Mus musculus OX=10090 GN=Igf2r PE=1 SV=1 | Igf2r | 0.86 |
| Q9QZQ8 | Core histone macro-H2A.1 OS=Mus musculus OX=10090 GN=Macroh2a1 PE=1 SV=3 | Macroh2a1 | 0.86 |
| Q8VCC1 | 15-hydroxyprostaglandin dehydrogenase [NAD(+)] OS=Mus musculus OX=10090 GN=Hpgd PE=1 SV=1 | Hpgd | 0.86 |
| Q9DAT2 | MRG/MORF4L-binding protein OS=Mus musculus OX=10090 GN=Mrgbp PE=1 SV=2 | Mrgbp | 0.86 |
| Q61081 | Hsp90 co-chaperone Cdc37 OS=Mus musculus OX=10090 GN=Cdc37 PE=1 SV=1 | Cdc37 | 0.86 |
| Q8BK72 | 28S ribosomal protein S27, mitochondrial OS=Mus musculus OX=10090 GN=Mrps27 PE=1 SV=2 | Mrps27 | 0.86 |
| Q3U2U7 | Methyltransferase-like protein 17, mitochondrial OS=Mus musculus OX=10090 GN=Mettl17 PE=1 SV=2 | Mettl17 | 0.86 |
| O08528 | Hexokinase-2 OS=Mus musculus OX=10090 GN=Hk2 PE=1 SV=1 | Hk2 | 0.86 |
| Q8R574 | Phosphoribosyl pyrophosphate synthase-associated protein 2 OS=Mus musculus OX=10090 GN=Prpsap2 PE=1 SV=1 | Prpsap2 | 0.86 |
| O35841 | Apoptosis inhibitor 5 OS=Mus musculus OX=10090 GN=Api5 PE=1 SV=2 | Api5 | 0.86 |
| Q8R1I1 | Cytochrome b-c1 complex subunit 9 OS=Mus musculus OX=10090 GN=Uqcr10 PE=1 SV=1 | Uqcr10 | 0.86 |
| Q3TUU5 | Testis-expressed protein 30 OS=Mus musculus OX=10090 GN=Tex30 PE=1 SV=1 | Tex30 | 0.86 |
| Q8CE96 | tRNA (adenine(58)-N(1))-methyltransferase non-catalytic subunit TRM6 OS=Mus musculus OX=10090 GN=Trmt6 PE=1 SV=1 | Trmt6 | 0.86 |
| Q8K1L5 | E3 ubiquitin-protein ligase PPP1R11 OS=Mus musculus OX=10090 GN=Ppp1r11 PE=1 SV=1 | Ppp1r11 | 0.86 |
| Q6P8X1 | Sorting nexin-6 OS=Mus musculus OX=10090 GN=Snx6 PE=1 SV=2 | Snx6 | 0.86 |
| P62835 | Ras-related protein Rap-1A OS=Mus musculus OX=10090 GN=Rap1a PE=1 SV=1 | Rap1a | 0.86 |
| Q7TNV0 | Protein DEK OS=Mus musculus OX=10090 GN=Dek PE=1 SV=1 | Dek | 0.86 |
| Q99JF8 | PC4 and SFRS1-interacting protein OS=Mus musculus OX=10090 GN=Psip1 PE=1 SV=1 | Psip1 | 0.86 |
| Q8K0C9 | GDP-mannose 4,6 dehydratase OS=Mus musculus OX=10090 GN=Gmds PE=1 SV=1 | Gmds | 0.86 |
| Q91ZN5 | Adenosine 3'-phospho 5'-phosphosulfate transporter 1 OS=Mus musculus OX=10090 GN=Slc35b2 PE=1 SV=1 | Slc35b2 | 0.86 |
| Q9ESU6 | Bromodomain-containing protein 4 OS=Mus musculus OX=10090 GN=Brd4 PE=1 SV=2 | Brd4 | 0.86 |
| Q8BXN9 | Transmembrane protein 87A OS=Mus musculus OX=10090 GN=Tmem87a PE=1 SV=1 | Tmem87a | 0.86 |
| P97379 | Ras GTPase-activating protein-binding protein 2 OS=Mus musculus OX=10090 GN=G3bp2 PE=1 SV=2 | G3bp2 | 0.86 |
| Q9CQF3 | Cleavage and polyadenylation specificity factor subunit 5 OS=Mus musculus OX=10090 GN=Nudt21 PE=1 SV=1 | Nudt21 | 0.86 |
| Q8BH24 | Transmembrane 9 superfamily member 4 OS=Mus musculus OX=10090 GN=Tm9sf4 PE=1 SV=1 | Tm9sf4 | 0.86 |
| O08553 | Dihydropyrimidinase-related protein 2 OS=Mus musculus OX=10090 GN=Dpysl2 PE=1 SV=2 | Dpysl2 | 0.86 |
| P42859 | Huntingtin OS=Mus musculus OX=10090 GN=Htt PE=1 SV=2 | Htt | 0.86 |
| Q91YN9 | BAG family molecular chaperone regulator 2 OS=Mus musculus OX=10090 GN=Bag2 PE=1 SV=1 | Bag2 | 0.86 |
| Q8VDF2 | E3 ubiquitin-protein ligase UHRF1 OS=Mus musculus OX=10090 GN=Uhrf1 PE=1 SV=2 | Uhrf1 | 0.86 |
| Q8CI59 | Metalloreductase STEAP3 OS=Mus musculus OX=10090 GN=Steap3 PE=1 SV=1 | Steap3 | 0.86 |
| Q9D1N9 | 39S ribosomal protein L21, mitochondrial OS=Mus musculus OX=10090 GN=Mrpl21 PE=1 SV=1 | Mrpl21 | 0.86 |
| Q91W18 | Tudor domain-containing protein 3 OS=Mus musculus OX=10090 GN=Tdrd3 PE=1 SV=4 | Tdrd3 | 0.86 |
| Q9D1I6 | 39S ribosomal protein L14, mitochondrial OS=Mus musculus OX=10090 GN=Mrpl14 PE=1 SV=1 | Mrpl14 | 0.86 |
| Q9QWY8 | Arf-GAP with SH3 domain, ANK repeat and PH domain-containing protein 1 OS=Mus musculus OX=10090 GN=Asap1 PE=1 SV=2 | Asap1 | 0.86 |
| P15864 | Histone H1.2 OS=Mus musculus OX=10090 GN=H1-2 PE=1 SV=2 | H1-2 | 0.86 |
| Q9WVL3 | Solute carrier family 12 member 7 OS=Mus musculus OX=10090 GN=Slc12a7 PE=1 SV=1 | Slc12a7 | 0.86 |
| Q08288 | Cell growth-regulating nucleolar protein OS=Mus musculus OX=10090 GN=Lyar PE=1 SV=2 | Lyar | 0.86 |
| Q8BH73 | Glutaminyl-peptide cyclotransferase-like protein OS=Mus musculus OX=10090 GN=Qpctl PE=1 SV=1 | Qpctl | 0.86 |
| Q3TCJ1 | BRISC complex subunit Abraxas 2 OS=Mus musculus OX=10090 GN=Abraxas2 PE=1 SV=1 | Abraxas2 | 0.86 |
| Q9CU62 | Structural maintenance of chromosomes protein 1A OS=Mus musculus OX=10090 GN=Smc1a PE=1 SV=4 | Smc1a | 0.86 |
| Q922J9 | Fatty acyl-CoA reductase 1 OS=Mus musculus OX=10090 GN=Far1 PE=1 SV=1 | Far1 | 0.86 |
| P97329 | Kinesin-like protein KIF20A OS=Mus musculus OX=10090 GN=Kif20a PE=1 SV=1 | Kif20a | 0.86 |
| Q6PNC0 | DmX-like protein 1 OS=Mus musculus OX=10090 GN=Dmxl1 PE=1 SV=1 | Dmxl1 | 0.86 |
| Q9Z1X4 | Interleukin enhancer-binding factor 3 OS=Mus musculus OX=10090 GN=Ilf3 PE=1 SV=2 | Ilf3 | 0.86 |
| Q8C7V3 | U3 small nucleolar RNA-associated protein 15 homolog OS=Mus musculus OX=10090 GN=Utp15 PE=1 SV=1 | Utp15 | 0.86 |
| Q3UHX0 | Nucleolar protein 8 OS=Mus musculus OX=10090 GN=Nol8 PE=1 SV=2 | Nol8 | 0.86 |
| Q03958 | Prefoldin subunit 6 OS=Mus musculus OX=10090 GN=Pfdn6 PE=1 SV=1 | Pfdn6 | 0.86 |
| Q9D7Z3 | Nucleolar protein 7 OS=Mus musculus OX=10090 GN=Nol7 PE=1 SV=1 | Nol7 | 0.86 |
| P26043 | Radixin OS=Mus musculus OX=10090 GN=Rdx PE=1 SV=3 | Rdx | 0.86 |
| Q8BXV2 | BRI3-binding protein OS=Mus musculus OX=10090 GN=Bri3bp PE=1 SV=1 | Bri3bp | 0.87 |
| P97814 | Proline-serine-threonine phosphatase-interacting protein 1 OS=Mus musculus OX=10090 GN=Pstpip1 PE=1 SV=1 | Pstpip1 | 0.87 |
| Q9CZM2 | 60S ribosomal protein L15 OS=Mus musculus OX=10090 GN=Rpl15 PE=1 SV=4 | Rpl15 | 0.87 |
| Q61543 | Golgi apparatus protein 1 OS=Mus musculus OX=10090 GN=Glg1 PE=1 SV=1 | Glg1 | 0.87 |
| Q91XC8 | Death-associated protein 1 OS=Mus musculus OX=10090 GN=Dap PE=1 SV=3 | Dap | 0.87 |
| Q9CYX7 | RRP15-like protein OS=Mus musculus OX=10090 GN=Rrp15 PE=1 SV=2 | Rrp15 | 0.87 |
| Q9JKB1 | Ubiquitin carboxyl-terminal hydrolase isozyme L3 OS=Mus musculus OX=10090 GN=Uchl3 PE=1 SV=2 | Uchl3 | 0.87 |
| P36371 | Antigen peptide transporter 2 OS=Mus musculus OX=10090 GN=Tap2 PE=1 SV=1 | Tap2 | 0.87 |
| Q99JY0 | Trifunctional enzyme subunit beta, mitochondrial OS=Mus musculus OX=10090 GN=Hadhb PE=1 SV=1 | Hadhb | 0.87 |
| Q9QYA2 | Mitochondrial import receptor subunit TOM40 homolog OS=Mus musculus OX=10090 GN=Tomm40 PE=1 SV=3 | Tomm40 | 0.87 |
| P70195 | Proteasome subunit beta type-7 OS=Mus musculus OX=10090 GN=Psmb7 PE=1 SV=1 | Psmb7 | 0.87 |
| Q9DC63 | F-box only protein 3 OS=Mus musculus OX=10090 GN=Fbxo3 PE=1 SV=1 | Fbxo3 | 0.87 |
| Q76KJ5 | DNA-directed RNA polymerase I subunit RPA34 OS=Mus musculus OX=10090 GN=Polr1g PE=1 SV=2 | Polr1g | 0.87 |
| Q9EST4 | Proteasome assembly chaperone 2 OS=Mus musculus OX=10090 GN=Psmg2 PE=1 SV=1 | Psmg2 | 0.87 |
| Q99K48 | Non-POU domain-containing octamer-binding protein OS=Mus musculus OX=10090 GN=Nono PE=1 SV=3 | Nono | 0.87 |
| Q61194 | Phosphatidylinositol 4-phosphate 3-kinase C2 domain-containing subunit alpha OS=Mus musculus OX=10090 GN=Pik3c2a PE=1 SV=2 | Pik3c2a | 0.87 |
| O35892 | Nuclear autoantigen Sp-100 OS=Mus musculus OX=10090 GN=Sp100 PE=1 SV=2 | Sp100 | 0.87 |
| Q924K8 | Metastasis-associated protein MTA3 OS=Mus musculus OX=10090 GN=Mta3 PE=1 SV=1 | Mta3 | 0.87 |
| O88952 | Protein lin-7 homolog C OS=Mus musculus OX=10090 GN=Lin7c PE=1 SV=2 | Lin7c | 0.87 |
| P05202 | Aspartate aminotransferase, mitochondrial OS=Mus musculus OX=10090 GN=Got2 PE=1 SV=1 | Got2 | 0.87 |
| Q9JL15 | Galectin-8 OS=Mus musculus OX=10090 GN=Lgals8 PE=1 SV=1 | Lgals8 | 0.87 |
| Q6PF93 | Phosphatidylinositol 3-kinase catalytic subunit type 3 OS=Mus musculus OX=10090 GN=Pik3c3 PE=1 SV=1 | Pik3c3 | 0.87 |
| P56382 | ATP synthase subunit epsilon, mitochondrial OS=Mus musculus OX=10090 GN=Atp5f1e PE=1 SV=2 | Atp5f1e | 0.87 |
| P14148 | 60S ribosomal protein L7 OS=Mus musculus OX=10090 GN=Rpl7 PE=1 SV=2 | Rpl7 | 0.87 |
| P99026 | Proteasome subunit beta type-4 OS=Mus musculus OX=10090 GN=Psmb4 PE=1 SV=1 | Psmb4 | 0.87 |
| Q8BL95 | Cilia- and flagella-associated protein 298 OS=Mus musculus OX=10090 GN=Cfap298 PE=1 SV=1 | Cfap298 | 0.87 |
| Q99LS3 | Phosphoserine phosphatase OS=Mus musculus OX=10090 GN=Psph PE=1 SV=1 | Psph | 0.87 |
| Q8VHE0 | Translocation protein SEC63 homolog OS=Mus musculus OX=10090 GN=Sec63 PE=1 SV=4 | Sec63 | 0.87 |
| Q04750 | DNA topoisomerase 1 OS=Mus musculus OX=10090 GN=Top1 PE=1 SV=2 | Top1 | 0.87 |
| Q9D0F6 | Replication factor C subunit 5 OS=Mus musculus OX=10090 GN=Rfc5 PE=1 SV=1 | Rfc5 | 0.87 |
| P54103 | DnaJ homolog subfamily C member 2 OS=Mus musculus OX=10090 GN=Dnajc2 PE=1 SV=2 | Dnajc2 | 0.87 |
| Q8VD04 | GRIP1-associated protein 1 OS=Mus musculus OX=10090 GN=Gripap1 PE=1 SV=1 | Gripap1 | 0.87 |
| Q9CR39 | WD repeat domain phosphoinositide-interacting protein 3 OS=Mus musculus OX=10090 GN=Wdr45b PE=1 SV=2 | Wdr45b | 0.87 |
| Q9EPU0 | Regulator of nonsense transcripts 1 OS=Mus musculus OX=10090 GN=Upf1 PE=1 SV=2 | Upf1 | 0.87 |
| Q3UPF5 | Zinc finger CCCH-type antiviral protein 1 OS=Mus musculus OX=10090 GN=Zc3hav1 PE=1 SV=1 | Zc3hav1 | 0.87 |
| Q99KN2 | Probable cytosolic iron-sulfur protein assembly protein CIAO1 OS=Mus musculus OX=10090 GN=Ciao1 PE=1 SV=1 | Ciao1 | 0.87 |
| Q6ZPU9 | KIF-binding protein OS=Mus musculus OX=10090 GN=Kifbp PE=1 SV=2 | Kifbp | 0.87 |
| P52927 | High mobility group protein HMGI-C OS=Mus musculus OX=10090 GN=Hmga2 PE=1 SV=1 | Hmga2 | 0.87 |
| Q91ZW3 | SWI/SNF-related matrix-associated actin-dependent regulator of chromatin subfamily A member 5 OS=Mus musculus OX=10090 GN=Smarca5 PE=1 SV=1 | Smarca5 | 0.87 |
| Q8BWY9 | Protein CIP2A OS=Mus musculus OX=10090 GN=Cip2a PE=1 SV=3 | Cip2a | 0.87 |
| P28867 | Protein kinase C delta type OS=Mus musculus OX=10090 GN=Prkcd PE=1 SV=3 | Prkcd | 0.87 |
| Q9CR68 | Cytochrome b-c1 complex subunit Rieske, mitochondrial OS=Mus musculus OX=10090 GN=Uqcrfs1 PE=1 SV=1 | Uqcrfs1 | 0.87 |
| Q80UW8 | DNA-directed RNA polymerases I, II, and III subunit RPABC1 OS=Mus musculus OX=10090 GN=Polr2e PE=1 SV=1 | Polr2e | 0.87 |
| Q01965 | T-lymphocyte surface antigen Ly-9 OS=Mus musculus OX=10090 GN=Ly9 PE=1 SV=2 | Ly9 | 0.87 |
| Q4LDD4 | Arf-GAP with Rho-GAP domain, ANK repeat and PH domain-containing protein 1 OS=Mus musculus OX=10090 GN=Arap1 PE=1 SV=2 | Arap1 | 0.87 |
| Q8BMP6 | Golgi resident protein GCP60 OS=Mus musculus OX=10090 GN=Acbd3 PE=1 SV=3 | Acbd3 | 0.87 |
| Q62318 | Transcription intermediary factor 1-beta OS=Mus musculus OX=10090 GN=Trim28 PE=1 SV=3 | Trim28 | 0.87 |
| Q8BG48 | Serine/threonine-protein kinase 17B OS=Mus musculus OX=10090 GN=Stk17b PE=1 SV=1 | Stk17b | 0.87 |
| Q9CWM4 | Prefoldin subunit 1 OS=Mus musculus OX=10090 GN=Pfdn1 PE=1 SV=1 | Pfdn1 | 0.87 |
| P27612 | Phospholipase A-2-activating protein OS=Mus musculus OX=10090 GN=Plaa PE=1 SV=4 | Plaa | 0.87 |
| P97311 | DNA replication licensing factor MCM6 OS=Mus musculus OX=10090 GN=Mcm6 PE=1 SV=1 | Mcm6 | 0.87 |
| Q99N87 | 28S ribosomal protein S5, mitochondrial OS=Mus musculus OX=10090 GN=Mrps5 PE=1 SV=1 | Mrps5 | 0.87 |
| O70493 | Sorting nexin-12 OS=Mus musculus OX=10090 GN=Snx12 PE=1 SV=1 | Snx12 | 0.87 |
| Q80X71 | Transmembrane protein 106B OS=Mus musculus OX=10090 GN=Tmem106b PE=1 SV=1 | Tmem106b | 0.87 |
| Q8CI11 | Guanine nucleotide-binding protein-like 3 OS=Mus musculus OX=10090 GN=Gnl3 PE=1 SV=2 | Gnl3 | 0.87 |
| A2APB8 | Targeting protein for Xklp2 OS=Mus musculus OX=10090 GN=Tpx2 PE=1 SV=1 | Tpx2 | 0.87 |
| Q8C878 | NEDD8-activating enzyme E1 catalytic subunit OS=Mus musculus OX=10090 GN=Uba3 PE=1 SV=2 | Uba3 | 0.87 |
| Q3UMY5 | Echinoderm microtubule-associated protein-like 4 OS=Mus musculus OX=10090 GN=Eml4 PE=1 SV=1 | Eml4 | 0.87 |
| Q7TSG2 | RNA polymerase II subunit A C-terminal domain phosphatase OS=Mus musculus OX=10090 GN=Ctdp1 PE=1 SV=1 | Ctdp1 | 0.87 |
| P03888 | NADH-ubiquinone oxidoreductase chain 1 OS=Mus musculus OX=10090 GN=Mtnd1 PE=1 SV=3 | Mtnd1 | 0.87 |
| Q8QZT1 | Acetyl-CoA acetyltransferase, mitochondrial OS=Mus musculus OX=10090 GN=Acat1 PE=1 SV=1 | Acat1 | 0.87 |
| Q9QZB9 | Dynactin subunit 5 OS=Mus musculus OX=10090 GN=Dctn5 PE=1 SV=1 | Dctn5 | 0.87 |
| Q9Z0P5 | Twinfilin-2 OS=Mus musculus OX=10090 GN=Twf2 PE=1 SV=1 | Twf2 | 0.87 |
| Q9JL35 | High mobility group nucleosome-binding domain-containing protein 5 OS=Mus musculus OX=10090 GN=Hmgn5 PE=1 SV=2 | Hmgn5 | 0.87 |
| Q91WC9 | Diacylglycerol lipase-beta OS=Mus musculus OX=10090 GN=Daglb PE=1 SV=2 | Daglb | 0.87 |
| P62257 | Ubiquitin-conjugating enzyme E2 H OS=Mus musculus OX=10090 GN=Ube2h PE=1 SV=1 | Ube2h | 0.87 |
| Q9QX47 | Protein SON OS=Mus musculus OX=10090 GN=Son PE=1 SV=2 | Son | 0.87 |
| Q922P9 | Putative oxidoreductase GLYR1 OS=Mus musculus OX=10090 GN=Glyr1 PE=1 SV=1 | Glyr1 | 0.87 |
| Q8R2T8 | General transcription factor 3C polypeptide 5 OS=Mus musculus OX=10090 GN=Gtf3c5 PE=2 SV=2 | Gtf3c5 | 0.87 |
| Q8BG73 | SH3 domain-binding glutamic acid-rich-like protein 2 OS=Mus musculus OX=10090 GN=Sh3bgrl2 PE=1 SV=1 | Sh3bgrl2 | 0.87 |
| Q9JKR6 | Hypoxia up-regulated protein 1 OS=Mus musculus OX=10090 GN=Hyou1 PE=1 SV=1 | Hyou1 | 0.87 |
| B2RXS4 | Plexin-B2 OS=Mus musculus OX=10090 GN=Plxnb2 PE=1 SV=1 | Plxnb2 | 0.87 |
| Q9QYF9 | Protein NDRG3 OS=Mus musculus OX=10090 GN=Ndrg3 PE=1 SV=1 | Ndrg3 | 0.87 |
| P70333 | Heterogeneous nuclear ribonucleoprotein H2 OS=Mus musculus OX=10090 GN=Hnrnph2 PE=1 SV=1 | Hnrnph2 | 0.87 |
| Q8BWT1 | 3-ketoacyl-CoA thiolase, mitochondrial OS=Mus musculus OX=10090 GN=Acaa2 PE=1 SV=3 | Acaa2 | 0.87 |
| Q9ER72 | Cysteine--tRNA ligase, cytoplasmic OS=Mus musculus OX=10090 GN=Cars1 PE=1 SV=2 | Cars1 | 0.87 |
| Q9EQU5 | Protein SET OS=Mus musculus OX=10090 GN=Set PE=1 SV=1 | Set | 0.87 |
| Q8CG47 | Structural maintenance of chromosomes protein 4 OS=Mus musculus OX=10090 GN=Smc4 PE=1 SV=1 | Smc4 | 0.87 |
| P61082 | NEDD8-conjugating enzyme Ubc12 OS=Mus musculus OX=10090 GN=Ube2m PE=1 SV=1 | Ube2m | 0.87 |
| Q80YR5 | Scaffold attachment factor B2 OS=Mus musculus OX=10090 GN=Safb2 PE=1 SV=2 | Safb2 | 0.87 |
| Q64191 | N(4)-(beta-N-acetylglucosaminyl)-L-asparaginase OS=Mus musculus OX=10090 GN=Aga PE=1 SV=1 | Aga | 0.87 |
| P70218 | Mitogen-activated protein kinase kinase kinase kinase 1 OS=Mus musculus OX=10090 GN=Map4k1 PE=1 SV=1 | Map4k1 | 0.87 |
| Q9R062 | Glycogenin-1 OS=Mus musculus OX=10090 GN=Gyg1 PE=1 SV=3 | Gyg1 | 0.87 |
| Q9D5V6 | Synapse-associated protein 1 OS=Mus musculus OX=10090 GN=Syap1 PE=1 SV=1 | Syap1 | 0.88 |
| O08579 | Emerin OS=Mus musculus OX=10090 GN=Emd PE=1 SV=1 | Emd | 0.88 |
| P35285 | Ras-related protein Rab-22A OS=Mus musculus OX=10090 GN=Rab22a PE=1 SV=2 | Rab22a | 0.88 |
| Q3UYC0 | Protein phosphatase 1H OS=Mus musculus OX=10090 GN=Ppm1h PE=1 SV=1 | Ppm1h | 0.88 |
| Q91VM5 | RNA binding motif protein, X-linked-like-1 OS=Mus musculus OX=10090 GN=Rbmxl1 PE=2 SV=1 | Rbmxl1 | 0.88 |
| P67871 | Casein kinase II subunit beta OS=Mus musculus OX=10090 GN=Csnk2b PE=1 SV=1 | Csnk2b | 0.88 |
| G5E829 | Plasma membrane calcium-transporting ATPase 1 OS=Mus musculus OX=10090 GN=Atp2b1 PE=1 SV=1 | Atp2b1 | 0.88 |
| Q5SW19 | Clustered mitochondria protein homolog OS=Mus musculus OX=10090 GN=Cluh PE=1 SV=2 | Cluh | 0.88 |
| Q8BZ20 | Protein mono-ADP-ribosyltransferase PARP12 OS=Mus musculus OX=10090 GN=Parp12 PE=1 SV=3 | Parp12 | 0.88 |
| O88379 | Bromodomain adjacent to zinc finger domain protein 1A OS=Mus musculus OX=10090 GN=Baz1a PE=1 SV=3 | Baz1a | 0.88 |
| Q99LC2 | Cleavage stimulation factor subunit 1 OS=Mus musculus OX=10090 GN=Cstf1 PE=1 SV=1 | Cstf1 | 0.88 |
| Q8K4Q8 | Collectin-12 OS=Mus musculus OX=10090 GN=Colec12 PE=1 SV=1 | Colec12 | 0.88 |
| Q8VIJ6 | Splicing factor, proline- and glutamine-rich OS=Mus musculus OX=10090 GN=Sfpq PE=1 SV=1 | Sfpq | 0.88 |
| O88874 | Cyclin-K OS=Mus musculus OX=10090 GN=Ccnk PE=1 SV=3 | Ccnk | 0.88 |
| P03975 | IgE-binding protein OS=Mus musculus OX=10090 GN=Iap PE=2 SV=1 | Iap | 0.88 |
| P38647 | Stress-70 protein, mitochondrial OS=Mus musculus OX=10090 GN=Hspa9 PE=1 SV=3 | Hspa9 | 0.88 |
| Q5SRY7 | F-box/WD repeat-containing protein 11 OS=Mus musculus OX=10090 GN=Fbxw11 PE=1 SV=1 | Fbxw11 | 0.88 |
| P98078 | Disabled homolog 2 OS=Mus musculus OX=10090 GN=Dab2 PE=1 SV=2 | Dab2 | 0.88 |
| Q6PIP5 | NudC domain-containing protein 1 OS=Mus musculus OX=10090 GN=Nudcd1 PE=1 SV=2 | Nudcd1 | 0.88 |
| Q922K7 | Probable 28S rRNA (cytosine-C(5))-methyltransferase OS=Mus musculus OX=10090 GN=Nop2 PE=1 SV=1 | Nop2 | 0.88 |
| Q921W4 | Quinone oxidoreductase-like protein 1 OS=Mus musculus OX=10090 GN=Cryzl1 PE=1 SV=1 | Cryzl1 | 0.88 |
| Q9D6K8 | FUN14 domain-containing protein 2 OS=Mus musculus OX=10090 GN=Fundc2 PE=1 SV=1 | Fundc2 | 0.88 |
| Q8R081 | Heterogeneous nuclear ribonucleoprotein L OS=Mus musculus OX=10090 GN=Hnrnpl PE=1 SV=2 | Hnrnpl | 0.88 |
| G5E870 | E3 ubiquitin-protein ligase TRIP12 OS=Mus musculus OX=10090 GN=Trip12 PE=1 SV=1 | Trip12 | 0.88 |
| Q9Z0S1 | 3'(2'),5'-bisphosphate nucleotidase 1 OS=Mus musculus OX=10090 GN=Bpnt1 PE=1 SV=2 | Bpnt1 | 0.88 |
| Q9DC16 | Endoplasmic reticulum-Golgi intermediate compartment protein 1 OS=Mus musculus OX=10090 GN=Ergic1 PE=1 SV=1 | Ergic1 | 0.88 |
| Q9QXK7 | Cleavage and polyadenylation specificity factor subunit 3 OS=Mus musculus OX=10090 GN=Cpsf3 PE=1 SV=2 | Cpsf3 | 0.88 |
| P37040 | NADPH--cytochrome P450 reductase OS=Mus musculus OX=10090 GN=Por PE=1 SV=2 | Por | 0.88 |
| Q8BMS4 | Ubiquinone biosynthesis O-methyltransferase, mitochondrial OS=Mus musculus OX=10090 GN=Coq3 PE=1 SV=1 | Coq3 | 0.88 |
| Q8BJ05 | Zinc finger CCCH domain-containing protein 14 OS=Mus musculus OX=10090 GN=Zc3h14 PE=1 SV=1 | Zc3h14 | 0.88 |
| Q9Z1X9 | Cell division control protein 45 homolog OS=Mus musculus OX=10090 GN=Cdc45 PE=1 SV=2 | Cdc45 | 0.88 |
| P54227 | Stathmin OS=Mus musculus OX=10090 GN=Stmn1 PE=1 SV=2 | Stmn1 | 0.88 |
| Q9Z0W3 | Nuclear pore complex protein Nup160 OS=Mus musculus OX=10090 GN=Nup160 PE=1 SV=2 | Nup160 | 0.88 |
| P97370 | Sodium/potassium-transporting ATPase subunit beta-3 OS=Mus musculus OX=10090 GN=Atp1b3 PE=1 SV=1 | Atp1b3 | 0.88 |
| Q91WC0 | Actin-histidine N-methyltransferase OS=Mus musculus OX=10090 GN=Setd3 PE=1 SV=1 | Setd3 | 0.88 |
| Q9JLJ2 | 4-trimethylaminobutyraldehyde dehydrogenase OS=Mus musculus OX=10090 GN=Aldh9a1 PE=1 SV=1 | Aldh9a1 | 0.88 |
| Q9WUV0 | Origin recognition complex subunit 5 OS=Mus musculus OX=10090 GN=Orc5 PE=2 SV=1 | Orc5 | 0.88 |
| Q8VD58 | Protein EVI2B OS=Mus musculus OX=10090 GN=Evi2b PE=1 SV=1 | Evi2b | 0.88 |
| Q9CQJ6 | Density-regulated protein OS=Mus musculus OX=10090 GN=Denr PE=1 SV=1 | Denr | 0.88 |
| Q61112 | 45 kDa calcium-binding protein OS=Mus musculus OX=10090 GN=Sdf4 PE=1 SV=1 | Sdf4 | 0.88 |
| Q91YW3 | DnaJ homolog subfamily C member 3 OS=Mus musculus OX=10090 GN=Dnajc3 PE=1 SV=1 | Dnajc3 | 0.88 |
| Q0VBL3 | RNA-binding protein 15 OS=Mus musculus OX=10090 GN=Rbm15 PE=1 SV=1 | Rbm15 | 0.88 |
| Q9WV03 | Protein FAM50A OS=Mus musculus OX=10090 GN=Fam50a PE=1 SV=1 | Fam50a | 0.88 |
| Q9CZ15 | DNA replication complex GINS protein PSF1 OS=Mus musculus OX=10090 GN=Gins1 PE=1 SV=1 | Gins1 | 0.88 |
| P19783 | Cytochrome c oxidase subunit 4 isoform 1, mitochondrial OS=Mus musculus OX=10090 GN=Cox4i1 PE=1 SV=2 | Cox4i1 | 0.88 |
| Q60864 | Stress-induced-phosphoprotein 1 OS=Mus musculus OX=10090 GN=Stip1 PE=1 SV=1 | Stip1 | 0.88 |
| Q61425 | Hydroxyacyl-coenzyme A dehydrogenase, mitochondrial OS=Mus musculus OX=10090 GN=Hadh PE=1 SV=2 | Hadh | 0.88 |
| Q8K2T4 | Ubiquinol-cytochrome-c reductase complex assembly factor 3 OS=Mus musculus OX=10090 GN=Uqcc3 PE=1 SV=1 | Uqcc3 | 0.88 |
| Q9CQX8 | 28S ribosomal protein S36, mitochondrial OS=Mus musculus OX=10090 GN=Mrps36 PE=1 SV=1 | Mrps36 | 0.88 |
| Q7TMR0 | Lysosomal Pro-X carboxypeptidase OS=Mus musculus OX=10090 GN=Prcp PE=1 SV=2 | Prcp | 0.88 |
| Q8CGB3 | Uveal autoantigen with coiled-coil domains and ankyrin repeats OS=Mus musculus OX=10090 GN=Uaca PE=1 SV=2 | Uaca | 0.88 |
| A2AIV2 | Protein virilizer homolog OS=Mus musculus OX=10090 GN=Virma PE=1 SV=1 | Virma | 0.88 |
| P20664 | DNA primase small subunit OS=Mus musculus OX=10090 GN=Prim1 PE=1 SV=1 | Prim1 | 0.88 |
| P68040 | Receptor of activated protein C kinase 1 OS=Mus musculus OX=10090 GN=Rack1 PE=1 SV=3 | Rack1 | 0.88 |
| Q8BWR2 | PITH domain-containing protein 1 OS=Mus musculus OX=10090 GN=Pithd1 PE=1 SV=1 | Pithd1 | 0.88 |
| Q9CZX8 | 40S ribosomal protein S19 OS=Mus musculus OX=10090 GN=Rps19 PE=1 SV=3 | Rps19 | 0.88 |
| Q9JMA2 | Queuine tRNA-ribosyltransferase catalytic subunit 1 OS=Mus musculus OX=10090 GN=Qtrt1 PE=1 SV=2 | Qtrt1 | 0.88 |
| Q9CPW4 | Actin-related protein 2/3 complex subunit 5 OS=Mus musculus OX=10090 GN=Arpc5 PE=1 SV=3 | Arpc5 | 0.88 |
| Q8JZU2 | Tricarboxylate transport protein, mitochondrial OS=Mus musculus OX=10090 GN=Slc25a1 PE=1 SV=1 | Slc25a1 | 0.88 |
| O35226 | 26S proteasome non-ATPase regulatory subunit 4 OS=Mus musculus OX=10090 GN=Psmd4 PE=1 SV=1 | Psmd4 | 0.88 |
| Q8K301 | Probable ATP-dependent RNA helicase DDX52 OS=Mus musculus OX=10090 GN=Ddx52 PE=2 SV=2 | Ddx52 | 0.88 |
| P17433 | Transcription factor PU.1 OS=Mus musculus OX=10090 GN=Spi1 PE=1 SV=2 | Spi1 | 0.88 |
| Q9Z130 | Heterogeneous nuclear ribonucleoprotein D-like OS=Mus musculus OX=10090 GN=Hnrnpdl PE=1 SV=1 | Hnrnpdl | 0.88 |
| P28063 | Proteasome subunit beta type-8 OS=Mus musculus OX=10090 GN=Psmb8 PE=1 SV=2 | Psmb8 | 0.88 |
| Q68FL6 | Methionine--tRNA ligase, cytoplasmic OS=Mus musculus OX=10090 GN=Mars1 PE=1 SV=1 | Mars1 | 0.88 |
| Q8VDQ9 | Protein KRI1 homolog OS=Mus musculus OX=10090 GN=Kri1 PE=1 SV=3 | Kri1 | 0.88 |
| Q8VDN2 | Sodium/potassium-transporting ATPase subunit alpha-1 OS=Mus musculus OX=10090 GN=Atp1a1 PE=1 SV=1 | Atp1a1 | 0.88 |
| P62274 | 40S ribosomal protein S29 OS=Mus musculus OX=10090 GN=Rps29 PE=1 SV=2 | Rps29 | 0.88 |
| Q9CR62 | Mitochondrial 2-oxoglutarate/malate carrier protein OS=Mus musculus OX=10090 GN=Slc25a11 PE=1 SV=3 | Slc25a11 | 0.88 |
| P10639 | Thioredoxin OS=Mus musculus OX=10090 GN=Txn PE=1 SV=3 | Txn | 0.88 |
| Q9QZE7 | Translin-associated protein X OS=Mus musculus OX=10090 GN=Tsnax PE=1 SV=1 | Tsnax | 0.88 |
| Q6PGB6 | N-alpha-acetyltransferase 50 OS=Mus musculus OX=10090 GN=Naa50 PE=1 SV=1 | Naa50 | 0.88 |
| Q791V5 | Mitochondrial carrier homolog 2 OS=Mus musculus OX=10090 GN=Mtch2 PE=1 SV=1 | Mtch2 | 0.88 |
| Q8C1A5 | Thimet oligopeptidase OS=Mus musculus OX=10090 GN=Thop1 PE=1 SV=1 | Thop1 | 0.88 |
| Q9JHS9 | Spliceosome-associated protein CWC15 homolog OS=Mus musculus OX=10090 GN=Cwc15 PE=1 SV=1 | Cwc15 | 0.88 |
| P97300 | Neuroplastin OS=Mus musculus OX=10090 GN=Nptn PE=1 SV=3 | Nptn | 0.88 |
| Q5XJE5 | RNA polymerase-associated protein LEO1 OS=Mus musculus OX=10090 GN=Leo1 PE=1 SV=2 | Leo1 | 0.88 |
| Q9QUR7 | Peptidyl-prolyl cis-trans isomerase NIMA-interacting 1 OS=Mus musculus OX=10090 GN=Pin1 PE=1 SV=1 | Pin1 | 0.88 |
| P19426 | Negative elongation factor E OS=Mus musculus OX=10090 GN=Nelfe PE=1 SV=2 | Nelfe | 0.88 |
| Q99N84 | 28S ribosomal protein S18b, mitochondrial OS=Mus musculus OX=10090 GN=Mrps18b PE=1 SV=1 | Mrps18b | 0.88 |
| P60605 | Ubiquitin-conjugating enzyme E2 G2 OS=Mus musculus OX=10090 GN=Ube2g2 PE=1 SV=1 | Ube2g2 | 0.88 |
| Q640M1 | U3 small nucleolar RNA-associated protein 14 homolog A OS=Mus musculus OX=10090 GN=Utp14a PE=1 SV=1 | Utp14a | 0.88 |
| Q14CH7 | Alanine--tRNA ligase, mitochondrial OS=Mus musculus OX=10090 GN=Aars2 PE=1 SV=1 | Aars2 | 0.88 |
| Q7TQI3 | Ubiquitin thioesterase OTUB1 OS=Mus musculus OX=10090 GN=Otub1 PE=1 SV=2 | Otub1 | 0.88 |
| Q61792 | LIM and SH3 domain protein 1 OS=Mus musculus OX=10090 GN=Lasp1 PE=1 SV=1 | Lasp1 | 0.88 |
| P97287 | Induced myeloid leukemia cell differentiation protein Mcl-1 homolog OS=Mus musculus OX=10090 GN=Mcl1 PE=1 SV=3 | Mcl1 | 0.88 |
| Q9CZE3 | Ras-related protein Rab-32 OS=Mus musculus OX=10090 GN=Rab32 PE=1 SV=3 | Rab32 | 0.89 |
| Q9EQ61 | Pescadillo homolog OS=Mus musculus OX=10090 GN=Pes1 PE=1 SV=1 | Pes1 | 0.89 |
| Q9CW46 | Ribonucleoprotein PTB-binding 1 OS=Mus musculus OX=10090 GN=Raver1 PE=1 SV=2 | Raver1 | 0.89 |
| Q8BYI6 | Lysophosphatidylcholine acyltransferase 2 OS=Mus musculus OX=10090 GN=Lpcat2 PE=1 SV=1 | Lpcat2 | 0.89 |
| Q8K1A6 | Coiled-coil and C2 domain-containing protein 1A OS=Mus musculus OX=10090 GN=Cc2d1a PE=1 SV=2 | Cc2d1a | 0.89 |
| Q8JZQ2 | AFG3-like protein 2 OS=Mus musculus OX=10090 GN=Afg3l2 PE=1 SV=1 | Afg3l2 | 0.89 |
| Q8CHT0 | Delta-1-pyrroline-5-carboxylate dehydrogenase, mitochondrial OS=Mus musculus OX=10090 GN=Aldh4a1 PE=1 SV=3 | Aldh4a1 | 0.89 |
| Q99020 | Heterogeneous nuclear ribonucleoprotein A/B OS=Mus musculus OX=10090 GN=Hnrnpab PE=1 SV=1 | Hnrnpab | 0.89 |
| O88597 | Beclin-1 OS=Mus musculus OX=10090 GN=Becn1 PE=1 SV=3 | Becn1 | 0.89 |
| O88696 | ATP-dependent Clp protease proteolytic subunit, mitochondrial OS=Mus musculus OX=10090 GN=Clpp PE=1 SV=1 | Clpp | 0.89 |
| Q91VD9 | NADH-ubiquinone oxidoreductase 75 kDa subunit, mitochondrial OS=Mus musculus OX=10090 GN=Ndufs1 PE=1 SV=2 | Ndufs1 | 0.89 |
| Q6P4S8 | Integrator complex subunit 1 OS=Mus musculus OX=10090 GN=Ints1 PE=1 SV=2 | Ints1 | 0.89 |
| Q9CQE1 | Protein NipSnap homolog 3B OS=Mus musculus OX=10090 GN=Nipsnap3b PE=1 SV=1 | Nipsnap3b | 0.89 |
| Q8R3C0 | Mini-chromosome maintenance complex-binding protein OS=Mus musculus OX=10090 GN=Mcmbp PE=1 SV=1 | Mcmbp | 0.89 |
| P63038 | 60 kDa heat shock protein, mitochondrial OS=Mus musculus OX=10090 GN=Hspd1 PE=1 SV=1 | Hspd1 | 0.89 |
| Q9WTX8 | Mitotic spindle assembly checkpoint protein MAD1 OS=Mus musculus OX=10090 GN=Mad1l1 PE=1 SV=1 | Mad1l1 | 0.89 |
| Q9CWK8 | Sorting nexin-2 OS=Mus musculus OX=10090 GN=Snx2 PE=1 SV=2 | Snx2 | 0.89 |
| P61358 | 60S ribosomal protein L27 OS=Mus musculus OX=10090 GN=Rpl27 PE=1 SV=2 | Rpl27 | 0.89 |
| Q8CDM8 | FHF complex subunit HOOK interacting protein 2A OS=Mus musculus OX=10090 GN=Fhip2a PE=1 SV=2 | Fhip2a | 0.89 |
| O70325 | Phospholipid hydroperoxide glutathione peroxidase OS=Mus musculus OX=10090 GN=Gpx4 PE=1 SV=4 | Gpx4 | 0.89 |
| Q7TPV4 | Myb-binding protein 1A OS=Mus musculus OX=10090 GN=Mybbp1a PE=1 SV=2 | Mybbp1a | 0.89 |
| Q9DCZ4 | MICOS complex subunit Mic26 OS=Mus musculus OX=10090 GN=Apoo PE=1 SV=2 | Apoo | 0.89 |
| Q9QY76 | Vesicle-associated membrane protein-associated protein B OS=Mus musculus OX=10090 GN=Vapb PE=1 SV=3 | Vapb | 0.89 |
| P41216 | Long-chain-fatty-acid--CoA ligase 1 OS=Mus musculus OX=10090 GN=Acsl1 PE=1 SV=2 | Acsl1 | 0.89 |
| Q6NV83 | U2 snRNP-associated SURP motif-containing protein OS=Mus musculus OX=10090 GN=U2surp PE=1 SV=3 | U2surp | 0.89 |
| Q99KE1 | NAD-dependent malic enzyme, mitochondrial OS=Mus musculus OX=10090 GN=Me2 PE=1 SV=1 | Me2 | 0.89 |
| Q3UEB3 | Poly(U)-binding-splicing factor PUF60 OS=Mus musculus OX=10090 GN=Puf60 PE=1 SV=2 | Puf60 | 0.89 |
| Q8VEG6 | CCR4-NOT transcription complex subunit 6-like OS=Mus musculus OX=10090 GN=Cnot6l PE=1 SV=2 | Cnot6l | 0.89 |
| P20152 | Vimentin OS=Mus musculus OX=10090 GN=Vim PE=1 SV=3 | Vim | 0.89 |
| Q99MD9 | Nuclear autoantigenic sperm protein OS=Mus musculus OX=10090 GN=Nasp PE=1 SV=2 | Nasp | 0.89 |
| D3Z7P3 | Glutaminase kidney isoform, mitochondrial OS=Mus musculus OX=10090 GN=Gls PE=1 SV=1 | Gls | 0.89 |
| Q80Y98 | Phospholipase DDHD2 OS=Mus musculus OX=10090 GN=Ddhd2 PE=1 SV=3 | Ddhd2 | 0.89 |
| Q9ERA6 | Tuftelin-interacting protein 11 OS=Mus musculus OX=10090 GN=Tfip11 PE=1 SV=1 | Tfip11 | 0.89 |
| Q9QWV9 | Cyclin-T1 OS=Mus musculus OX=10090 GN=Ccnt1 PE=1 SV=3 | Ccnt1 | 0.89 |
| Q9CPT4 | Myeloid-derived growth factor OS=Mus musculus OX=10090 GN=Mydgf PE=1 SV=1 | Mydgf | 0.89 |
| Q8VBT6 | Apolipoprotein B receptor OS=Mus musculus OX=10090 GN=Apobr PE=1 SV=1 | Apobr | 0.89 |
| Q9CXE7 | Transmembrane emp24 domain-containing protein 5 OS=Mus musculus OX=10090 GN=Tmed5 PE=1 SV=1 | Tmed5 | 0.89 |
| Q8BG26 | AP-4 complex accessory subunit RUSC1 OS=Mus musculus OX=10090 GN=Rusc1 PE=1 SV=2 | Rusc1 | 0.89 |
| Q9D1Q4 | Dolichol-phosphate mannosyltransferase subunit 3 OS=Mus musculus OX=10090 GN=Dpm3 PE=1 SV=1 | Dpm3 | 0.89 |
| Q9CY58 | Plasminogen activator inhibitor 1 RNA-binding protein OS=Mus musculus OX=10090 GN=Serbp1 PE=1 SV=2 | Serbp1 | 0.89 |
| Q921K9 | B-cell CLL/lymphoma 7 protein family member B OS=Mus musculus OX=10090 GN=Bcl7b PE=1 SV=1 | Bcl7b | 0.89 |
| Q91VJ1 | Interferon-inducible protein AIM2 OS=Mus musculus OX=10090 GN=Aim2 PE=1 SV=2 | Aim2 | 0.89 |
| Q8C163 | Nuclease EXOG, mitochondrial OS=Mus musculus OX=10090 GN=Exog PE=1 SV=1 | Exog | 0.89 |
| Q7TMF3 | NADH dehydrogenase [ubiquinone] 1 alpha subcomplex subunit 12 OS=Mus musculus OX=10090 GN=Ndufa12 PE=1 SV=2 | Ndufa12 | 0.89 |
| P63158 | High mobility group protein B1 OS=Mus musculus OX=10090 GN=Hmgb1 PE=1 SV=2 | Hmgb1 | 0.89 |
| P56959 | RNA-binding protein FUS OS=Mus musculus OX=10090 GN=Fus PE=1 SV=1 | Fus | 0.89 |
| P62869 | Elongin-B OS=Mus musculus OX=10090 GN=Elob PE=1 SV=1 | Elob | 0.89 |
| P83882 | 60S ribosomal protein L36a OS=Mus musculus OX=10090 GN=Rpl36a PE=3 SV=2 | Rpl36a | 0.89 |
| Q60787 | Lymphocyte cytosolic protein 2 OS=Mus musculus OX=10090 GN=Lcp2 PE=1 SV=2 | Lcp2 | 0.89 |
| Q8JZM7 | Parafibromin OS=Mus musculus OX=10090 GN=Cdc73 PE=1 SV=1 | Cdc73 | 0.89 |
| Q9CTG6 | Polyamine-transporting ATPase 13A2 OS=Mus musculus OX=10090 GN=Atp13a2 PE=2 SV=3 | Atp13a2 | 0.89 |
| P62983 | Ubiquitin-40S ribosomal protein S27a OS=Mus musculus OX=10090 GN=Rps27a PE=1 SV=2 | Rps27a | 0.89 |
| Q99M28 | RNA-binding protein with serine-rich domain 1 OS=Mus musculus OX=10090 GN=Rnps1 PE=1 SV=1 | Rnps1 | 0.89 |
| Q9QXS1 | Plectin OS=Mus musculus OX=10090 GN=Plec PE=1 SV=3 | Plec | 0.89 |
| Q9JJG9 | Nitric oxide-associated protein 1 OS=Mus musculus OX=10090 GN=Noa1 PE=1 SV=1 | Noa1 | 0.89 |
| Q8C7X2 | ER membrane protein complex subunit 1 OS=Mus musculus OX=10090 GN=Emc1 PE=1 SV=1 | Emc1 | 0.89 |
| Q9CQF9 | Prenylcysteine oxidase OS=Mus musculus OX=10090 GN=Pcyox1 PE=1 SV=1 | Pcyox1 | 0.89 |
| Q921H8 | 3-ketoacyl-CoA thiolase A, peroxisomal OS=Mus musculus OX=10090 GN=Acaa1a PE=1 SV=1 | Acaa1a | 0.89 |
| Q60992 | Guanine nucleotide exchange factor VAV2 OS=Mus musculus OX=10090 GN=Vav2 PE=1 SV=1 | Vav2 | 0.89 |
| Q9QX60 | Deoxyguanosine kinase, mitochondrial OS=Mus musculus OX=10090 GN=Dguok PE=1 SV=3 | Dguok | 0.89 |
| Q99LP6 | GrpE protein homolog 1, mitochondrial OS=Mus musculus OX=10090 GN=Grpel1 PE=1 SV=1 | Grpel1 | 0.89 |
| Q8VEA4 | Mitochondrial intermembrane space import and assembly protein 40 OS=Mus musculus OX=10090 GN=Chchd4 PE=1 SV=1 | Chchd4 | 0.89 |
| Q8R146 | Acylamino-acid-releasing enzyme OS=Mus musculus OX=10090 GN=Apeh PE=1 SV=3 | Apeh | 0.89 |
| Q9Z315 | U4/U6.U5 tri-snRNP-associated protein 1 OS=Mus musculus OX=10090 GN=Sart1 PE=1 SV=1 | Sart1 | 0.89 |
| Q9CPT5 | Nucleolar protein 16 OS=Mus musculus OX=10090 GN=Nop16 PE=1 SV=1 | Nop16 | 0.89 |
| Q80TP3 | E3 ubiquitin-protein ligase UBR5 OS=Mus musculus OX=10090 GN=Ubr5 PE=1 SV=2 | Ubr5 | 0.89 |
| P43346 | Deoxycytidine kinase OS=Mus musculus OX=10090 GN=Dck PE=1 SV=1 | Dck | 0.89 |
| P62806 | Histone H4 OS=Mus musculus OX=10090 GN=H4c1 PE=1 SV=2 | H4c1 | 0.89 |
| Q61074 | Protein phosphatase 1G OS=Mus musculus OX=10090 GN=Ppm1g PE=1 SV=3 | Ppm1g | 0.89 |
| O35130 | Ribosomal RNA small subunit methyltransferase NEP1 OS=Mus musculus OX=10090 GN=Emg1 PE=1 SV=1 | Emg1 | 0.89 |
| Q9D172 | Glutamine amidotransferase-like class 1 domain-containing protein 3, mitochondrial OS=Mus musculus OX=10090 GN=Gatd3 PE=1 SV=1 | Gatd3 | 0.89 |
| Q91VX2 | Ubiquitin-associated protein 2 OS=Mus musculus OX=10090 GN=Ubap2 PE=1 SV=1 | Ubap2 | 0.89 |
| Q61687 | Transcriptional regulator ATRX OS=Mus musculus OX=10090 GN=Atrx PE=1 SV=3 | Atrx | 0.89 |
| Q91VU7 | Pseudouridylate synthase 7 homolog OS=Mus musculus OX=10090 GN=Pus7 PE=2 SV=2 | Pus7 | 0.89 |
| O70404 | Vesicle-associated membrane protein 8 OS=Mus musculus OX=10090 GN=Vamp8 PE=1 SV=1 | Vamp8 | 0.89 |
| Q8K2T1 | NmrA-like family domain-containing protein 1 OS=Mus musculus OX=10090 GN=Nmral1 PE=1 SV=1 | Nmral1 | 0.89 |
| Q99J36 | THUMP domain-containing protein 1 OS=Mus musculus OX=10090 GN=Thumpd1 PE=1 SV=1 | Thumpd1 | 0.89 |
| Q8C4Y3 | Negative elongation factor B OS=Mus musculus OX=10090 GN=Nelfb PE=1 SV=2 | Nelfb | 0.89 |
| Q5SVQ0 | Histone acetyltransferase KAT7 OS=Mus musculus OX=10090 GN=Kat7 PE=1 SV=1 | Kat7 | 0.89 |
| Q8BXQ2 | GPI transamidase component PIG-T OS=Mus musculus OX=10090 GN=Pigt PE=1 SV=2 | Pigt | 0.89 |
| P61222 | ATP-binding cassette sub-family E member 1 OS=Mus musculus OX=10090 GN=Abce1 PE=1 SV=1 | Abce1 | 0.89 |
| O70469 | Docking protein 2 OS=Mus musculus OX=10090 GN=Dok2 PE=1 SV=1 | Dok2 | 0.89 |
| P41230 | Lysine-specific demethylase 5C OS=Mus musculus OX=10090 GN=Kdm5c PE=1 SV=4 | Kdm5c | 0.89 |
| P01897 | H-2 class I histocompatibility antigen, L-D alpha chain OS=Mus musculus OX=10090 GN=H2-L PE=1 SV=2 | H2-L | 0.89 |
| Q9CYZ2 | Tumor protein D54 OS=Mus musculus OX=10090 GN=Tpd52l2 PE=1 SV=1 | Tpd52l2 | 0.89 |
| Q03249 | Galactose-1-phosphate uridylyltransferase OS=Mus musculus OX=10090 GN=Galt PE=1 SV=3 | Galt | 0.89 |
| E9Q7E2 | AT-rich interactive domain-containing protein 2 OS=Mus musculus OX=10090 GN=Arid2 PE=1 SV=1 | Arid2 | 0.89 |
| Q62376 | U1 small nuclear ribonucleoprotein 70 kDa OS=Mus musculus OX=10090 GN=Snrnp70 PE=1 SV=2 | Snrnp70 | 0.89 |
| Q6ZQF0 | DNA topoisomerase 2-binding protein 1 OS=Mus musculus OX=10090 GN=Topbp1 PE=1 SV=2 | Topbp1 | 0.89 |
| Q9WUN2 | Serine/threonine-protein kinase TBK1 OS=Mus musculus OX=10090 GN=Tbk1 PE=1 SV=1 | Tbk1 | 0.89 |
| Q9D1K2 | V-type proton ATPase subunit F OS=Mus musculus OX=10090 GN=Atp6v1f PE=1 SV=2 | Atp6v1f | 0.89 |
| Q8BH43 | Wiskott-Aldrich syndrome protein family member 2 OS=Mus musculus OX=10090 GN=Wasf2 PE=1 SV=1 | Wasf2 | 0.89 |
| Q9CZW5 | Mitochondrial import receptor subunit TOM70 OS=Mus musculus OX=10090 GN=Tomm70 PE=1 SV=2 | Tomm70 | 0.89 |
| Q61024 | Asparagine synthetase [glutamine-hydrolyzing] OS=Mus musculus OX=10090 GN=Asns PE=1 SV=3 | Asns | 0.89 |
| Q8VI33 | Transcription initiation factor TFIID subunit 9 OS=Mus musculus OX=10090 GN=Taf9 PE=1 SV=1 | Taf9 | 0.90 |
| P97765 | WW domain-binding protein 2 OS=Mus musculus OX=10090 GN=Wbp2 PE=1 SV=1 | Wbp2 | 0.90 |
| Q8R0G9 | Nuclear pore complex protein Nup133 OS=Mus musculus OX=10090 GN=Nup133 PE=1 SV=2 | Nup133 | 0.90 |
| Q8CFY5 | Protoheme IX farnesyltransferase, mitochondrial OS=Mus musculus OX=10090 GN=Cox10 PE=2 SV=1 | Cox10 | 0.90 |
| P22315 | Ferrochelatase, mitochondrial OS=Mus musculus OX=10090 GN=Fech PE=1 SV=2 | Fech | 0.90 |
| B1ARD6 | Schlafen family member 9 OS=Mus musculus OX=10090 GN=Slfn9 PE=2 SV=1 | Slfn9 | 0.90 |
| Q7TT37 | Elongator complex protein 1 OS=Mus musculus OX=10090 GN=Elp1 PE=1 SV=2 | Elp1 | 0.90 |
| Q9R190 | Metastasis-associated protein MTA2 OS=Mus musculus OX=10090 GN=Mta2 PE=1 SV=1 | Mta2 | 0.90 |
| O88554 | Poly [ADP-ribose] polymerase 2 OS=Mus musculus OX=10090 GN=Parp2 PE=1 SV=3 | Parp2 | 0.90 |
| Q64008 | Ras-related protein Rab-34 OS=Mus musculus OX=10090 GN=Rab34 PE=1 SV=2 | Rab34 | 0.90 |
| P58064 | 28S ribosomal protein S6, mitochondrial OS=Mus musculus OX=10090 GN=Mrps6 PE=1 SV=3 | Mrps6 | 0.90 |
| Q61102 | Iron-sulfur clusters transporter ABCB7, mitochondrial OS=Mus musculus OX=10090 GN=Abcb7 PE=1 SV=3 | Abcb7 | 0.90 |
| Q3UMU9 | Hepatoma-derived growth factor-related protein 2 OS=Mus musculus OX=10090 GN=Hdgfl2 PE=1 SV=1 | Hdgfl2 | 0.90 |
| P48678 | Prelamin-A/C OS=Mus musculus OX=10090 GN=Lmna PE=1 SV=2 | Lmna | 0.90 |
| Q08943 | FACT complex subunit SSRP1 OS=Mus musculus OX=10090 GN=Ssrp1 PE=1 SV=2 | Ssrp1 | 0.90 |
| P55937 | Golgin subfamily A member 3 OS=Mus musculus OX=10090 GN=Golga3 PE=1 SV=3 | Golga3 | 0.90 |
| Q3U4G3 | Xyloside xylosyltransferase 1 OS=Mus musculus OX=10090 GN=Xxylt1 PE=1 SV=2 | Xxylt1 | 0.90 |
| Q9CW03 | Structural maintenance of chromosomes protein 3 OS=Mus musculus OX=10090 GN=Smc3 PE=1 SV=2 | Smc3 | 0.90 |
| Q5SUQ9 | CST complex subunit CTC1 OS=Mus musculus OX=10090 GN=Ctc1 PE=1 SV=2 | Ctc1 | 0.90 |
| Q8BS35 | Alkylglycerol monooxygenase OS=Mus musculus OX=10090 GN=Agmo PE=1 SV=1 | Agmo | 0.90 |
| Q5BLK4 | Terminal uridylyltransferase 7 OS=Mus musculus OX=10090 GN=Tut7 PE=1 SV=3 | Tut7 | 0.90 |
| Q8BMG7 | Rab3 GTPase-activating protein non-catalytic subunit OS=Mus musculus OX=10090 GN=Rab3gap2 PE=1 SV=2 | Rab3gap2 | 0.90 |
| Q91W39 | Nuclear receptor coactivator 5 OS=Mus musculus OX=10090 GN=Ncoa5 PE=1 SV=1 | Ncoa5 | 0.90 |
| P32020 | Sterol carrier protein 2 OS=Mus musculus OX=10090 GN=Scp2 PE=1 SV=3 | Scp2 | 0.90 |
| Q9D6N5 | Dr1-associated corepressor OS=Mus musculus OX=10090 GN=Drap1 PE=1 SV=3 | Drap1 | 0.90 |
| Q8K284 | General transcription factor 3C polypeptide 1 OS=Mus musculus OX=10090 GN=Gtf3c1 PE=1 SV=2 | Gtf3c1 | 0.90 |
| Q8R3F9 | Speckle targeted PIP5K1A-regulated poly(A) polymerase OS=Mus musculus OX=10090 GN=Tut1 PE=1 SV=1 | Tut1 | 0.90 |
| Q9DCA2 | 28S ribosomal protein S11, mitochondrial OS=Mus musculus OX=10090 GN=Mrps11 PE=1 SV=2 | Mrps11 | 0.90 |
| P14685 | 26S proteasome non-ATPase regulatory subunit 3 OS=Mus musculus OX=10090 GN=Psmd3 PE=1 SV=3 | Psmd3 | 0.90 |
| Q8C2Q3 | RNA-binding protein 14 OS=Mus musculus OX=10090 GN=Rbm14 PE=1 SV=1 | Rbm14 | 0.90 |
| Q99J99 | 3-mercaptopyruvate sulfurtransferase OS=Mus musculus OX=10090 GN=Mpst PE=1 SV=4 | Mpst | 0.90 |
| Q9CX56 | 26S proteasome non-ATPase regulatory subunit 8 OS=Mus musculus OX=10090 GN=Psmd8 PE=1 SV=2 | Psmd8 | 0.90 |
| Q9D110 | 5-formyltetrahydrofolate cyclo-ligase OS=Mus musculus OX=10090 GN=Mthfs PE=2 SV=2 | Mthfs | 0.90 |
| Q9WU84 | Copper chaperone for superoxide dismutase OS=Mus musculus OX=10090 GN=Ccs PE=1 SV=1 | Ccs | 0.90 |
| Q80YA7 | Dipeptidyl peptidase 8 OS=Mus musculus OX=10090 GN=Dpp8 PE=1 SV=1 | Dpp8 | 0.90 |
| Q69Z38 | Inactive tyrosine-protein kinase PEAK1 OS=Mus musculus OX=10090 GN=Peak1 PE=1 SV=4 | Peak1 | 0.90 |
| Q99LI9 | Polyribonucleotide 5'-hydroxyl-kinase Clp1 OS=Mus musculus OX=10090 GN=Clp1 PE=1 SV=1 | Clp1 | 0.90 |
| Q9CZX7 | Type 2 phosphatidylinositol 4,5-bisphosphate 4-phosphatase OS=Mus musculus OX=10090 GN=Pip4p2 PE=1 SV=1 | Pip4p2 | 0.90 |
| P49452 | Centromere protein C OS=Mus musculus OX=10090 GN=Cenpc PE=1 SV=2 | Cenpc | 0.90 |
| Q8C9B9 | Death-inducer obliterator 1 OS=Mus musculus OX=10090 GN=Dido1 PE=1 SV=4 | Dido1 | 0.90 |
| Q9EQ32 | Phosphoinositide 3-kinase adapter protein 1 OS=Mus musculus OX=10090 GN=Pik3ap1 PE=1 SV=1 | Pik3ap1 | 0.90 |
| Q8BJW6 | Eukaryotic translation initiation factor 2A OS=Mus musculus OX=10090 GN=Eif2a PE=1 SV=2 | Eif2a | 0.90 |
| Q01320 | DNA topoisomerase 2-alpha OS=Mus musculus OX=10090 GN=Top2a PE=1 SV=2 | Top2a | 0.90 |
| Q9R099 | Transducin beta-like protein 2 OS=Mus musculus OX=10090 GN=Tbl2 PE=1 SV=2 | Tbl2 | 0.90 |
| Q99LL3 | Carbohydrate sulfotransferase 12 OS=Mus musculus OX=10090 GN=Chst12 PE=2 SV=2 | Chst12 | 0.90 |
| Q6P8I4 | PEST proteolytic signal-containing nuclear protein OS=Mus musculus OX=10090 GN=Pcnp PE=1 SV=1 | Pcnp | 0.90 |
| Q8R2U6 | Diphosphoinositol polyphosphate phosphohydrolase 2 OS=Mus musculus OX=10090 GN=Nudt4 PE=1 SV=1 | Nudt4 | 0.90 |
| Q9Z2A5 | Arginyl-tRNA--protein transferase 1 OS=Mus musculus OX=10090 GN=Ate1 PE=1 SV=2 | Ate1 | 0.90 |
| Q3TX08 | tRNA (guanine(26)-N(2))-dimethyltransferase OS=Mus musculus OX=10090 GN=Trmt1 PE=1 SV=2 | Trmt1 | 0.90 |
| Q9JJ94 | Sjoegren syndrome nuclear autoantigen 1 homolog OS=Mus musculus OX=10090 GN=Ssna1 PE=1 SV=1 | Ssna1 | 0.90 |
| P56542 | Deoxyribonuclease-2-alpha OS=Mus musculus OX=10090 GN=Dnase2 PE=1 SV=1 | Dnase2 | 0.90 |
| Q99P69 | Kinetochore protein Nuf2 OS=Mus musculus OX=10090 GN=Nuf2 PE=1 SV=2 | Nuf2 | 0.90 |
| P97822 | Acidic leucine-rich nuclear phosphoprotein 32 family member E OS=Mus musculus OX=10090 GN=Anp32e PE=1 SV=2 | Anp32e | 0.90 |
| Q8BXC6 | COMM domain-containing protein 2 OS=Mus musculus OX=10090 GN=Commd2 PE=1 SV=1 | Commd2 | 0.90 |
| P53612 | Geranylgeranyl transferase type-2 subunit beta OS=Mus musculus OX=10090 GN=Rabggtb PE=1 SV=2 | Rabggtb | 0.90 |
| Q9CR67 | Transmembrane protein 33 OS=Mus musculus OX=10090 GN=Tmem33 PE=1 SV=1 | Tmem33 | 0.90 |
| Q8K363 | ATP-dependent RNA helicase DDX18 OS=Mus musculus OX=10090 GN=Ddx18 PE=1 SV=1 | Ddx18 | 0.90 |
| O54879 | High mobility group protein B3 OS=Mus musculus OX=10090 GN=Hmgb3 PE=1 SV=3 | Hmgb3 | 0.90 |
| Q9JK81 | MYG1 exonuclease OS=Mus musculus OX=10090 GN=Myg1 PE=1 SV=1 | Myg1 | 0.90 |
| Q9JIX8 | Apoptotic chromatin condensation inducer in the nucleus OS=Mus musculus OX=10090 GN=Acin1 PE=1 SV=3 | Acin1 | 0.90 |
| Q9D0E1 | Heterogeneous nuclear ribonucleoprotein M OS=Mus musculus OX=10090 GN=Hnrnpm PE=1 SV=3 | Hnrnpm | 0.90 |
| Q5RJG1 | Nucleolar protein 10 OS=Mus musculus OX=10090 GN=Nol10 PE=2 SV=1 | Nol10 | 0.90 |
| Q9QUM9 | Proteasome subunit alpha type-6 OS=Mus musculus OX=10090 GN=Psma6 PE=1 SV=1 | Psma6 | 0.90 |
| Q9R060 | Cytosolic Fe-S cluster assembly factor NUBP1 OS=Mus musculus OX=10090 GN=Nubp1 PE=1 SV=1 | Nubp1 | 0.90 |
| Q99MS7 | EH domain-binding protein 1-like protein 1 OS=Mus musculus OX=10090 GN=Ehbp1l1 PE=1 SV=1 | Ehbp1l1 | 0.90 |
| Q922U1 | U4/U6 small nuclear ribonucleoprotein Prp3 OS=Mus musculus OX=10090 GN=Prpf3 PE=1 SV=1 | Prpf3 | 0.90 |
| Q61881 | DNA replication licensing factor MCM7 OS=Mus musculus OX=10090 GN=Mcm7 PE=1 SV=1 | Mcm7 | 0.90 |
| Q9DB05 | Alpha-soluble NSF attachment protein OS=Mus musculus OX=10090 GN=Napa PE=1 SV=1 | Napa | 0.90 |
| Q9CX34 | Protein SGT1 homolog OS=Mus musculus OX=10090 GN=Sugt1 PE=1 SV=3 | Sugt1 | 0.90 |
| Q9CQ91 | NADH dehydrogenase [ubiquinone] 1 alpha subcomplex subunit 3 OS=Mus musculus OX=10090 GN=Ndufa3 PE=1 SV=1 | Ndufa3 | 0.90 |
| A2AAY5 | SH3 and PX domain-containing protein 2B OS=Mus musculus OX=10090 GN=Sh3pxd2b PE=1 SV=1 | Sh3pxd2b | 0.90 |
| Q9D0B0 | Serine/arginine-rich splicing factor 9 OS=Mus musculus OX=10090 GN=Srsf9 PE=1 SV=1 | Srsf9 | 0.90 |
| Q9WUK2 | Eukaryotic translation initiation factor 4H OS=Mus musculus OX=10090 GN=Eif4h PE=1 SV=3 | Eif4h | 0.90 |
| Q9JII5 | DAZ-associated protein 1 OS=Mus musculus OX=10090 GN=Dazap1 PE=1 SV=2 | Dazap1 | 0.90 |
| Q8BIQ5 | Cleavage stimulation factor subunit 2 OS=Mus musculus OX=10090 GN=Cstf2 PE=1 SV=2 | Cstf2 | 0.90 |
| Q8R3L2 | Transcription factor 25 OS=Mus musculus OX=10090 GN=Tcf25 PE=1 SV=2 | Tcf25 | 0.90 |
| Q91VA6 | Polymerase delta-interacting protein 2 OS=Mus musculus OX=10090 GN=Poldip2 PE=1 SV=1 | Poldip2 | 0.91 |
| Q6P9L6 | Kinesin-like protein KIF15 OS=Mus musculus OX=10090 GN=Kif15 PE=1 SV=1 | Kif15 | 0.91 |
| P62196 | 26S proteasome regulatory subunit 8 OS=Mus musculus OX=10090 GN=Psmc5 PE=1 SV=1 | Psmc5 | 0.91 |
| Q8C156 | Condensin complex subunit 2 OS=Mus musculus OX=10090 GN=Ncaph PE=1 SV=1 | Ncaph | 0.91 |
| P26040 | Ezrin OS=Mus musculus OX=10090 GN=Ezr PE=1 SV=3 | Ezr | 0.91 |
| Q99LI7 | Cleavage stimulation factor subunit 3 OS=Mus musculus OX=10090 GN=Cstf3 PE=1 SV=1 | Cstf3 | 0.91 |
| P49615 | Cyclin-dependent-like kinase 5 OS=Mus musculus OX=10090 GN=Cdk5 PE=1 SV=1 | Cdk5 | 0.91 |
| Q9ERI5 | Bifunctional arginine demethylase and lysyl-hydroxylase JMJD6 OS=Mus musculus OX=10090 GN=Jmjd6 PE=1 SV=2 | Jmjd6 | 0.91 |
| Q9WVJ2 | 26S proteasome non-ATPase regulatory subunit 13 OS=Mus musculus OX=10090 GN=Psmd13 PE=1 SV=1 | Psmd13 | 0.91 |
| Q99MV1 | Tudor domain-containing protein 1 OS=Mus musculus OX=10090 GN=Tdrd1 PE=1 SV=2 | Tdrd1 | 0.91 |
| Q62433 | Protein NDRG1 OS=Mus musculus OX=10090 GN=Ndrg1 PE=1 SV=1 | Ndrg1 | 0.91 |
| P31996 | Macrosialin OS=Mus musculus OX=10090 GN=Cd68 PE=1 SV=1 | Cd68 | 0.91 |
| Q80VJ3 | 2'-deoxynucleoside 5'-phosphate N-hydrolase 1 OS=Mus musculus OX=10090 GN=Dnph1 PE=1 SV=2 | Dnph1 | 0.91 |
| Q6PD03 | Serine/threonine-protein phosphatase 2A 56 kDa regulatory subunit alpha isoform OS=Mus musculus OX=10090 GN=Ppp2r5a PE=1 SV=1 | Ppp2r5a | 0.91 |
| Q9DCT6 | Chromatin complexes subunit BAP18 OS=Mus musculus OX=10090 GN=Bap18 PE=1 SV=1 | Bap18 | 0.91 |
| Q3UJD6 | Ubiquitin carboxyl-terminal hydrolase 19 OS=Mus musculus OX=10090 GN=Usp19 PE=1 SV=1 | Usp19 | 0.91 |
| O89051 | Integral membrane protein 2B OS=Mus musculus OX=10090 GN=Itm2b PE=1 SV=1 | Itm2b | 0.91 |
| Q9CQM9 | Glutaredoxin-3 OS=Mus musculus OX=10090 GN=Glrx3 PE=1 SV=1 | Glrx3 | 0.91 |
| Q9D1H8 | 39S ribosomal protein L53, mitochondrial OS=Mus musculus OX=10090 GN=Mrpl53 PE=1 SV=1 | Mrpl53 | 0.91 |
| Q8VE37 | Regulator of chromosome condensation OS=Mus musculus OX=10090 GN=Rcc1 PE=1 SV=1 | Rcc1 | 0.91 |
| P47810 | Wee1-like protein kinase OS=Mus musculus OX=10090 GN=Wee1 PE=1 SV=2 | Wee1 | 0.91 |
| Q9D6L8 | Peptidyl-prolyl cis-trans isomerase-like 3 OS=Mus musculus OX=10090 GN=Ppil3 PE=1 SV=1 | Ppil3 | 0.91 |
| Q8R4B8 | NACHT, LRR and PYD domains-containing protein 3 OS=Mus musculus OX=10090 GN=Nlrp3 PE=1 SV=1 | Nlrp3 | 0.91 |
| Q8R1V4 | Transmembrane emp24 domain-containing protein 4 OS=Mus musculus OX=10090 GN=Tmed4 PE=1 SV=1 | Tmed4 | 0.91 |
| Q9DBE9 | pre-rRNA 2'-O-ribose RNA methyltransferase FTSJ3 OS=Mus musculus OX=10090 GN=Ftsj3 PE=1 SV=1 | Ftsj3 | 0.91 |
| Q8VEA8 | Ras-related protein Rab-7b OS=Mus musculus OX=10090 GN=Rab7b PE=1 SV=1 | Rab7b | 0.91 |
| Q9D083 | Kinetochore protein Spc24 OS=Mus musculus OX=10090 GN=Spc24 PE=1 SV=1 | Spc24 | 0.91 |
| Q8K4L0 | ATP-dependent RNA helicase DDX54 OS=Mus musculus OX=10090 GN=Ddx54 PE=1 SV=1 | Ddx54 | 0.91 |
| Q9Z2M7 | Phosphomannomutase 2 OS=Mus musculus OX=10090 GN=Pmm2 PE=1 SV=1 | Pmm2 | 0.91 |
| Q3TCH7 | Cullin-4A OS=Mus musculus OX=10090 GN=Cul4a PE=1 SV=1 | Cul4a | 0.91 |
| O08795 | Glucosidase 2 subunit beta OS=Mus musculus OX=10090 GN=Prkcsh PE=1 SV=1 | Prkcsh | 0.91 |
| Q9CSN1 | SNW domain-containing protein 1 OS=Mus musculus OX=10090 GN=Snw1 PE=1 SV=3 | Snw1 | 0.91 |
| Q9ERG2 | Striatin-3 OS=Mus musculus OX=10090 GN=Strn3 PE=1 SV=1 | Strn3 | 0.91 |
| Q9D2X5 | MAU2 chromatid cohesion factor homolog OS=Mus musculus OX=10090 GN=Mau2 PE=1 SV=3 | Mau2 | 0.91 |
| Q8CH25 | SAFB-like transcription modulator OS=Mus musculus OX=10090 GN=Sltm PE=1 SV=1 | Sltm | 0.91 |
| P62320 | Small nuclear ribonucleoprotein Sm D3 OS=Mus musculus OX=10090 GN=Snrpd3 PE=1 SV=1 | Snrpd3 | 0.91 |
| Q8BX17 | Gem-associated protein 5 OS=Mus musculus OX=10090 GN=Gemin5 PE=1 SV=2 | Gemin5 | 0.91 |
| O55023 | Inositol monophosphatase 1 OS=Mus musculus OX=10090 GN=Impa1 PE=1 SV=1 | Impa1 | 0.91 |
| Q9CYC5 | Kinetochore-associated protein DSN1 homolog OS=Mus musculus OX=10090 GN=Dsn1 PE=1 SV=1 | Dsn1 | 0.91 |
| Q9DCT5 | Stromal cell-derived factor 2 OS=Mus musculus OX=10090 GN=Sdf2 PE=1 SV=1 | Sdf2 | 0.91 |
| P62313 | U6 snRNA-associated Sm-like protein LSm6 OS=Mus musculus OX=10090 GN=Lsm6 PE=1 SV=1 | Lsm6 | 0.91 |
| Q8BSQ9 | Protein polybromo-1 OS=Mus musculus OX=10090 GN=Pbrm1 PE=1 SV=4 | Pbrm1 | 0.91 |
| Q9CZP5 | Mitochondrial chaperone BCS1 OS=Mus musculus OX=10090 GN=Bcs1l PE=1 SV=1 | Bcs1l | 0.91 |
| Q9D6K5 | Synaptojanin-2-binding protein OS=Mus musculus OX=10090 GN=Synj2bp PE=1 SV=1 | Synj2bp | 0.91 |
| P59016 | Vacuolar protein sorting-associated protein 33B OS=Mus musculus OX=10090 GN=Vps33b PE=1 SV=1 | Vps33b | 0.91 |
| Q9CRB9 | MICOS complex subunit Mic19 OS=Mus musculus OX=10090 GN=Chchd3 PE=1 SV=1 | Chchd3 | 0.91 |
| P58006 | Sestrin-1 OS=Mus musculus OX=10090 GN=Sesn1 PE=1 SV=3 | Sesn1 | 0.91 |
| Q3UQ84 | Threonine--tRNA ligase, mitochondrial OS=Mus musculus OX=10090 GN=Tars2 PE=1 SV=1 | Tars2 | 0.91 |
| Q5DW34 | Histone-lysine N-methyltransferase EHMT1 OS=Mus musculus OX=10090 GN=Ehmt1 PE=1 SV=2 | Ehmt1 | 0.91 |
| Q8BTI8 | Serine/arginine repetitive matrix protein 2 OS=Mus musculus OX=10090 GN=Srrm2 PE=1 SV=3 | Srrm2 | 0.91 |
| Q9CZR8 | Elongation factor Ts, mitochondrial OS=Mus musculus OX=10090 GN=Tsfm PE=1 SV=1 | Tsfm | 0.91 |
| Q9DC23 | DnaJ homolog subfamily C member 10 OS=Mus musculus OX=10090 GN=Dnajc10 PE=1 SV=2 | Dnajc10 | 0.91 |
| P16254 | Signal recognition particle 14 kDa protein OS=Mus musculus OX=10090 GN=Srp14 PE=1 SV=1 | Srp14 | 0.91 |
| O35685 | Nuclear migration protein nudC OS=Mus musculus OX=10090 GN=Nudc PE=1 SV=1 | Nudc | 0.91 |
| Q9DCG9 | Multifunctional methyltransferase subunit TRM112-like protein OS=Mus musculus OX=10090 GN=Trmt112 PE=1 SV=1 | Trmt112 | 0.91 |
| Q61595 | Kinectin OS=Mus musculus OX=10090 GN=Ktn1 PE=1 SV=1 | Ktn1 | 0.91 |
| Q99JX7 | Nuclear RNA export factor 1 OS=Mus musculus OX=10090 GN=Nxf1 PE=1 SV=3 | Nxf1 | 0.91 |
| P62488 | DNA-directed RNA polymerase II subunit RPB7 OS=Mus musculus OX=10090 GN=Polr2g PE=1 SV=1 | Polr2g | 0.91 |
| Q8K0H5 | Transcription initiation factor TFIID subunit 10 OS=Mus musculus OX=10090 GN=Taf10 PE=1 SV=1 | Taf10 | 0.91 |
| Q78YY6 | DnaJ homolog subfamily C member 15 OS=Mus musculus OX=10090 GN=Dnajc15 PE=1 SV=1 | Dnajc15 | 0.91 |
| O54885 | TYRO protein tyrosine kinase-binding protein OS=Mus musculus OX=10090 GN=Tyrobp PE=1 SV=1 | Tyrobp | 0.91 |
| Q9CR26 | Vacuolar protein sorting-associated protein VTA1 homolog OS=Mus musculus OX=10090 GN=Vta1 PE=1 SV=1 | Vta1 | 0.91 |
| Q61189 | Methylosome subunit pICln OS=Mus musculus OX=10090 GN=Clns1a PE=1 SV=1 | Clns1a | 0.91 |
| Q9EPB5 | Serine hydrolase-like protein OS=Mus musculus OX=10090 GN=Serhl PE=1 SV=1 | Serhl | 0.91 |
| Q80UM3 | N-alpha-acetyltransferase 15, NatA auxiliary subunit OS=Mus musculus OX=10090 GN=Naa15 PE=1 SV=1 | Naa15 | 0.91 |
| B1AZA5 | Transmembrane protein 245 OS=Mus musculus OX=10090 GN=Tmem245 PE=1 SV=1 | Tmem245 | 0.91 |
| P56671 | Myc-associated zinc finger protein OS=Mus musculus OX=10090 GN=Maz PE=1 SV=1 | Maz | 0.91 |
| Q8BIJ6 | Isoleucine--tRNA ligase, mitochondrial OS=Mus musculus OX=10090 GN=Iars2 PE=1 SV=1 | Iars2 | 0.91 |
| P58742 | Aladin OS=Mus musculus OX=10090 GN=Aaas PE=1 SV=1 | Aaas | 0.91 |
| Q99PT3 | INO80 complex subunit B OS=Mus musculus OX=10090 GN=Ino80b PE=1 SV=2 | Ino80b | 0.91 |
| Q9R0M6 | Ras-related protein Rab-9A OS=Mus musculus OX=10090 GN=Rab9a PE=1 SV=1 | Rab9a | 0.91 |
| Q3UMT1 | Protein phosphatase 1 regulatory subunit 12C OS=Mus musculus OX=10090 GN=Ppp1r12c PE=1 SV=1 | Ppp1r12c | 0.91 |
| Q8BG15 | CTD small phosphatase-like protein 2 OS=Mus musculus OX=10090 GN=Ctdspl2 PE=1 SV=1 | Ctdspl2 | 0.91 |
| Q99K28 | ADP-ribosylation factor GTPase-activating protein 2 OS=Mus musculus OX=10090 GN=Arfgap2 PE=1 SV=1 | Arfgap2 | 0.91 |
| Q8BWW9 | Serine/threonine-protein kinase N2 OS=Mus musculus OX=10090 GN=Pkn2 PE=1 SV=3 | Pkn2 | 0.91 |
| Q9D168 | Integrator complex subunit 12 OS=Mus musculus OX=10090 GN=Ints12 PE=1 SV=1 | Ints12 | 0.91 |
| Q3U7R1 | Extended synaptotagmin-1 OS=Mus musculus OX=10090 GN=Esyt1 PE=1 SV=2 | Esyt1 | 0.91 |
| Q61733 | 28S ribosomal protein S31, mitochondrial OS=Mus musculus OX=10090 GN=Mrps31 PE=1 SV=1 | Mrps31 | 0.91 |
| Q8C5N3 | Pre-mRNA-splicing factor CWC22 homolog OS=Mus musculus OX=10090 GN=Cwc22 PE=1 SV=1 | Cwc22 | 0.91 |
| O70435 | Proteasome subunit alpha type-3 OS=Mus musculus OX=10090 GN=Psma3 PE=1 SV=3 | Psma3 | 0.91 |
| O89017 | Legumain OS=Mus musculus OX=10090 GN=Lgmn PE=1 SV=1 | Lgmn | 0.91 |
| Q8K4B0 | Metastasis-associated protein MTA1 OS=Mus musculus OX=10090 GN=Mta1 PE=1 SV=1 | Mta1 | 0.91 |
| P51150 | Ras-related protein Rab-7a OS=Mus musculus OX=10090 GN=Rab7a PE=1 SV=2 | Rab7a | 0.91 |
| Q8BJQ2 | Ubiquitin carboxyl-terminal hydrolase 1 OS=Mus musculus OX=10090 GN=Usp1 PE=1 SV=1 | Usp1 | 0.91 |
| Q9DB96 | Neuroguidin OS=Mus musculus OX=10090 GN=Ngdn PE=1 SV=1 | Ngdn | 0.91 |
| O55201 | Transcription elongation factor SPT5 OS=Mus musculus OX=10090 GN=Supt5h PE=1 SV=1 | Supt5h | 0.91 |
| O35382 | Exocyst complex component 4 OS=Mus musculus OX=10090 GN=Exoc4 PE=1 SV=2 | Exoc4 | 0.91 |
| P62307 | Small nuclear ribonucleoprotein F OS=Mus musculus OX=10090 GN=Snrpf PE=1 SV=1 | Snrpf | 0.91 |
| P42932 | T-complex protein 1 subunit theta OS=Mus musculus OX=10090 GN=Cct8 PE=1 SV=3 | Cct8 | 0.91 |
| Q8R033 | LYR motif-containing protein 2 OS=Mus musculus OX=10090 GN=Lyrm2 PE=3 SV=1 | Lyrm2 | 0.91 |
| Q3U9N9 | Monocarboxylate transporter 10 OS=Mus musculus OX=10090 GN=Slc16a10 PE=1 SV=1 | Slc16a10 | 0.91 |
| Q91W82 | Ubiquitin-conjugating enzyme E2 E2 OS=Mus musculus OX=10090 GN=Ube2e2 PE=2 SV=1 | Ube2e2 | 0.91 |
| Q9CRC8 | Leucine-rich repeat-containing protein 40 OS=Mus musculus OX=10090 GN=Lrrc40 PE=1 SV=2 | Lrrc40 | 0.91 |
| B8ZXI1 | Queuine tRNA-ribosyltransferase accessory subunit 2 OS=Mus musculus OX=10090 GN=Qtrt2 PE=1 SV=2 | Qtrt2 | 0.91 |
| Q62093 | Serine/arginine-rich splicing factor 2 OS=Mus musculus OX=10090 GN=Srsf2 PE=1 SV=4 | Srsf2 | 0.91 |
| P68373 | Tubulin alpha-1C chain OS=Mus musculus OX=10090 GN=Tuba1c PE=1 SV=1 | Tuba1c | 0.91 |
| Q5KU39 | Vacuolar protein sorting-associated protein 41 homolog OS=Mus musculus OX=10090 GN=Vps41 PE=1 SV=1 | Vps41 | 0.91 |
| Q8BGD9 | Eukaryotic translation initiation factor 4B OS=Mus musculus OX=10090 GN=Eif4b PE=1 SV=1 | Eif4b | 0.91 |
| Q8CGC6 | RNA-binding protein 28 OS=Mus musculus OX=10090 GN=Rbm28 PE=1 SV=4 | Rbm28 | 0.91 |
| P97868 | E3 ubiquitin-protein ligase RBBP6 OS=Mus musculus OX=10090 GN=Rbbp6 PE=1 SV=5 | Rbbp6 | 0.91 |
| P00158 | Cytochrome b OS=Mus musculus OX=10090 GN=Mt-Cyb PE=1 SV=1 | Mt-Cyb | 0.91 |
| Q99JR1 | Sideroflexin-1 OS=Mus musculus OX=10090 GN=Sfxn1 PE=1 SV=3 | Sfxn1 | 0.91 |
| Q99PV0 | Pre-mRNA-processing-splicing factor 8 OS=Mus musculus OX=10090 GN=Prpf8 PE=1 SV=2 | Prpf8 | 0.91 |
| Q9EQ80 | NIF3-like protein 1 OS=Mus musculus OX=10090 GN=Nif3l1 PE=1 SV=4 | Nif3l1 | 0.91 |
| Q921H9 | Cytochrome c oxidase assembly factor 7 OS=Mus musculus OX=10090 GN=Coa7 PE=1 SV=1 | Coa7 | 0.91 |
| Q99MJ9 | ATP-dependent RNA helicase DDX50 OS=Mus musculus OX=10090 GN=Ddx50 PE=2 SV=1 | Ddx50 | 0.91 |
| Q64727 | Vinculin OS=Mus musculus OX=10090 GN=Vcl PE=1 SV=4 | Vcl | 0.91 |
| Q9Z1M8 | Protein Red OS=Mus musculus OX=10090 GN=Ik PE=1 SV=2 | Ik | 0.91 |
| Q3U821 | WD repeat-containing protein 75 OS=Mus musculus OX=10090 GN=Wdr75 PE=1 SV=1 | Wdr75 | 0.91 |
| Q8BSP2 | Condensin-2 complex subunit H2 OS=Mus musculus OX=10090 GN=Ncaph2 PE=1 SV=1 | Ncaph2 | 0.92 |
| P55302 | Alpha-2-macroglobulin receptor-associated protein OS=Mus musculus OX=10090 GN=Lrpap1 PE=1 SV=1 | Lrpap1 | 0.92 |
| Q8C1Q6 | Small integral membrane protein 4 OS=Mus musculus OX=10090 GN=Smim4 PE=1 SV=2 | Smim4 | 0.92 |
| Q9Z120 | tRNA (guanine-N(7)-)-methyltransferase OS=Mus musculus OX=10090 GN=Mettl1 PE=1 SV=1 | Mettl1 | 0.92 |
| P12970 | 60S ribosomal protein L7a OS=Mus musculus OX=10090 GN=Rpl7a PE=1 SV=2 | Rpl7a | 0.92 |
| Q9WVS7 | Dual specificity mitogen-activated protein kinase kinase 5 OS=Mus musculus OX=10090 GN=Map2k5 PE=1 SV=1 | Map2k5 | 0.92 |
| Q3U9G9 | Delta(14)-sterol reductase LBR OS=Mus musculus OX=10090 GN=Lbr PE=1 SV=2 | Lbr | 0.92 |
| Q9QYC0 | Alpha-adducin OS=Mus musculus OX=10090 GN=Add1 PE=1 SV=2 | Add1 | 0.92 |
| Q9QZL0 | Receptor-interacting serine/threonine-protein kinase 3 OS=Mus musculus OX=10090 GN=Ripk3 PE=1 SV=2 | Ripk3 | 0.92 |
| Q9JLT4 | Thioredoxin reductase 2, mitochondrial OS=Mus musculus OX=10090 GN=Txnrd2 PE=1 SV=4 | Txnrd2 | 0.92 |
| Q91VH2 | Sorting nexin-9 OS=Mus musculus OX=10090 GN=Snx9 PE=1 SV=1 | Snx9 | 0.92 |
| Q9CZN8 | Glutamyl-tRNA(Gln) amidotransferase subunit A, mitochondrial OS=Mus musculus OX=10090 GN=Qrsl1 PE=1 SV=1 | Qrsl1 | 0.92 |
| Q9DCM0 | Persulfide dioxygenase ETHE1, mitochondrial OS=Mus musculus OX=10090 GN=Ethe1 PE=1 SV=2 | Ethe1 | 0.92 |
| Q80UK7 | Spindle assembly abnormal protein 6 homolog OS=Mus musculus OX=10090 GN=Sass6 PE=2 SV=2 | Sass6 | 0.92 |
| Q3UQA7 | Selenoprotein H OS=Mus musculus OX=10090 GN=Selenoh PE=1 SV=2 | Selenoh | 0.92 |
| Q9CPR5 | 39S ribosomal protein L15, mitochondrial OS=Mus musculus OX=10090 GN=Mrpl15 PE=1 SV=1 | Mrpl15 | 0.92 |
| P39429 | TNF receptor-associated factor 2 OS=Mus musculus OX=10090 GN=Traf2 PE=1 SV=1 | Traf2 | 0.92 |
| P27546 | Microtubule-associated protein 4 OS=Mus musculus OX=10090 GN=Map4 PE=1 SV=3 | Map4 | 0.92 |
| Q60854 | Serpin B6 OS=Mus musculus OX=10090 GN=Serpinb6 PE=1 SV=1 | Serpinb6 | 0.92 |
| Q9CWT6 | Probable ATP-dependent RNA helicase DDX28 OS=Mus musculus OX=10090 GN=Ddx28 PE=2 SV=2 | Ddx28 | 0.92 |
| Q61191 | Host cell factor 1 OS=Mus musculus OX=10090 GN=Hcfc1 PE=1 SV=2 | Hcfc1 | 0.92 |
| Q60520 | Paired amphipathic helix protein Sin3a OS=Mus musculus OX=10090 GN=Sin3a PE=1 SV=3 | Sin3a | 0.92 |
| P05555 | Integrin alpha-M OS=Mus musculus OX=10090 GN=Itgam PE=1 SV=2 | Itgam | 0.92 |
| Q8BGC4 | Prostaglandin reductase-3 OS=Mus musculus OX=10090 GN=Zadh2 PE=1 SV=1 | Zadh2 | 0.92 |
| Q8CFI2 | Ubiquitin-conjugating enzyme E2 R1 OS=Mus musculus OX=10090 GN=Cdc34 PE=1 SV=1 | Cdc34 | 0.92 |
| P62281 | 40S ribosomal protein S11 OS=Mus musculus OX=10090 GN=Rps11 PE=1 SV=3 | Rps11 | 0.92 |
| Q9CR98 | Protein FAM136A OS=Mus musculus OX=10090 GN=Fam136a PE=1 SV=1 | Fam136a | 0.92 |
| P97429 | Annexin A4 OS=Mus musculus OX=10090 GN=Anxa4 PE=1 SV=4 | Anxa4 | 0.92 |
| O55234 | Proteasome subunit beta type-5 OS=Mus musculus OX=10090 GN=Psmb5 PE=1 SV=3 | Psmb5 | 0.92 |
| Q7TND5 | Ribosome production factor 1 OS=Mus musculus OX=10090 GN=Rpf1 PE=2 SV=2 | Rpf1 | 0.92 |
| P97352 | Protein S100-A13 OS=Mus musculus OX=10090 GN=S100a13 PE=1 SV=1 | S100a13 | 0.92 |
| Q80X41 | Serine/threonine-protein kinase VRK1 OS=Mus musculus OX=10090 GN=Vrk1 PE=1 SV=2 | Vrk1 | 0.92 |
| Q9WV54 | Acid ceramidase OS=Mus musculus OX=10090 GN=Asah1 PE=1 SV=1 | Asah1 | 0.92 |
| P63328 | Serine/threonine-protein phosphatase 2B catalytic subunit alpha isoform OS=Mus musculus OX=10090 GN=Ppp3ca PE=1 SV=1 | Ppp3ca | 0.92 |
| Q922D8 | C-1-tetrahydrofolate synthase, cytoplasmic OS=Mus musculus OX=10090 GN=Mthfd1 PE=1 SV=4 | Mthfd1 | 0.92 |
| P53986 | Monocarboxylate transporter 1 OS=Mus musculus OX=10090 GN=Slc16a1 PE=1 SV=1 | Slc16a1 | 0.92 |
| P58281 | Dynamin-like 120 kDa protein, mitochondrial OS=Mus musculus OX=10090 GN=Opa1 PE=1 SV=1 | Opa1 | 0.92 |
| Q8CAQ8 | MICOS complex subunit Mic60 OS=Mus musculus OX=10090 GN=Immt PE=1 SV=1 | Immt | 0.92 |
| Q9CQV8 | 14-3-3 protein beta/alpha OS=Mus musculus OX=10090 GN=Ywhab PE=1 SV=3 | Ywhab | 0.92 |
| Q9EPV8 | Ubiquitin-like protein 5 OS=Mus musculus OX=10090 GN=Ubl5 PE=1 SV=1 | Ubl5 | 0.92 |
| P80315 | T-complex protein 1 subunit delta OS=Mus musculus OX=10090 GN=Cct4 PE=1 SV=3 | Cct4 | 0.92 |
| Q8C078 | Calcium/calmodulin-dependent protein kinase kinase 2 OS=Mus musculus OX=10090 GN=Camkk2 PE=1 SV=2 | Camkk2 | 0.92 |
| Q91WK2 | Eukaryotic translation initiation factor 3 subunit H OS=Mus musculus OX=10090 GN=Eif3h PE=1 SV=1 | Eif3h | 0.92 |
| Q9DC37 | Major facilitator superfamily domain-containing protein 1 OS=Mus musculus OX=10090 GN=Mfsd1 PE=1 SV=1 | Mfsd1 | 0.92 |
| P62748 | Hippocalcin-like protein 1 OS=Mus musculus OX=10090 GN=Hpcal1 PE=1 SV=2 | Hpcal1 | 0.92 |
| Q68FF6 | ARF GTPase-activating protein GIT1 OS=Mus musculus OX=10090 GN=Git1 PE=1 SV=1 | Git1 | 0.92 |
| O88487 | Cytoplasmic dynein 1 intermediate chain 2 OS=Mus musculus OX=10090 GN=Dync1i2 PE=1 SV=1 | Dync1i2 | 0.92 |
| P0CG15 | Chromosome transmission fidelity protein 8 homolog OS=Mus musculus OX=10090 GN=Chtf8 PE=3 SV=1 | Chtf8 | 0.92 |
| Q6NVF9 | Cleavage and polyadenylation specificity factor subunit 6 OS=Mus musculus OX=10090 GN=Cpsf6 PE=1 SV=1 | Cpsf6 | 0.92 |
| Q921Y2 | U3 small nucleolar ribonucleoprotein protein IMP3 OS=Mus musculus OX=10090 GN=Imp3 PE=2 SV=1 | Imp3 | 0.92 |
| Q8BJY1 | 26S proteasome non-ATPase regulatory subunit 5 OS=Mus musculus OX=10090 GN=Psmd5 PE=1 SV=4 | Psmd5 | 0.92 |
| Q8C052 | Microtubule-associated protein 1S OS=Mus musculus OX=10090 GN=Map1s PE=1 SV=2 | Map1s | 0.92 |
| O88425 | Nucleoside diphosphate kinase 6 OS=Mus musculus OX=10090 GN=Nme6 PE=1 SV=1 | Nme6 | 0.92 |
| Q66GT5 | Phosphatidylglycerophosphatase and protein-tyrosine phosphatase 1 OS=Mus musculus OX=10090 GN=Ptpmt1 PE=1 SV=1 | Ptpmt1 | 0.92 |
| Q8BSK8 | Ribosomal protein S6 kinase beta-1 OS=Mus musculus OX=10090 GN=Rps6kb1 PE=1 SV=2 | Rps6kb1 | 0.92 |
| Q61210 | Rho guanine nucleotide exchange factor 1 OS=Mus musculus OX=10090 GN=Arhgef1 PE=1 SV=2 | Arhgef1 | 0.92 |
| Q9JIF7 | Coatomer subunit beta OS=Mus musculus OX=10090 GN=Copb1 PE=1 SV=1 | Copb1 | 0.92 |
| Q8BMA6 | Signal recognition particle subunit SRP68 OS=Mus musculus OX=10090 GN=Srp68 PE=1 SV=2 | Srp68 | 0.92 |
| Q91VW5 | Golgin subfamily A member 4 OS=Mus musculus OX=10090 GN=Golga4 PE=1 SV=2 | Golga4 | 0.92 |
| Q3UMB5 | Guanine nucleotide exchange protein SMCR8 OS=Mus musculus OX=10090 GN=Smcr8 PE=1 SV=2 | Smcr8 | 0.92 |
| P62301 | 40S ribosomal protein S13 OS=Mus musculus OX=10090 GN=Rps13 PE=1 SV=2 | Rps13 | 0.92 |
| P31266 | Recombining binding protein suppressor of hairless OS=Mus musculus OX=10090 GN=Rbpj PE=1 SV=1 | Rbpj | 0.92 |
| Q9R1P1 | Proteasome subunit beta type-3 OS=Mus musculus OX=10090 GN=Psmb3 PE=1 SV=1 | Psmb3 | 0.92 |
| Q9WVE8 | Protein kinase C and casein kinase substrate in neurons protein 2 OS=Mus musculus OX=10090 GN=Pacsin2 PE=1 SV=1 | Pacsin2 | 0.92 |
| P30416 | Peptidyl-prolyl cis-trans isomerase FKBP4 OS=Mus musculus OX=10090 GN=Fkbp4 PE=1 SV=5 | Fkbp4 | 0.92 |
| Q9D1R9 | 60S ribosomal protein L34 OS=Mus musculus OX=10090 GN=Rpl34 PE=1 SV=2 | Rpl34 | 0.92 |
| Q60749 | KH domain-containing, RNA-binding, signal transduction-associated protein 1 OS=Mus musculus OX=10090 GN=Khdrbs1 PE=1 SV=2 | Khdrbs1 | 0.92 |
| Q8CHH9 | Septin-8 OS=Mus musculus OX=10090 GN=Septin8 PE=1 SV=4 | Septin8 | 0.92 |
| Q9DCN2 | NADH-cytochrome b5 reductase 3 OS=Mus musculus OX=10090 GN=Cyb5r3 PE=1 SV=3 | Cyb5r3 | 0.92 |
| P08003 | Protein disulfide-isomerase A4 OS=Mus musculus OX=10090 GN=Pdia4 PE=1 SV=3 | Pdia4 | 0.92 |
| O55143 | Sarcoplasmic/endoplasmic reticulum calcium ATPase 2 OS=Mus musculus OX=10090 GN=Atp2a2 PE=1 SV=2 | Atp2a2 | 0.92 |
| P18760 | Cofilin-1 OS=Mus musculus OX=10090 GN=Cfl1 PE=1 SV=3 | Cfl1 | 0.92 |
| Q80W54 | CAAX prenyl protease 1 homolog OS=Mus musculus OX=10090 GN=Zmpste24 PE=1 SV=2 | Zmpste24 | 0.92 |
| Q9D2G2 | Dihydrolipoyllysine-residue succinyltransferase component of 2-oxoglutarate dehydrogenase complex, mitochondrial OS=Mus musculus OX=10090 GN=Dlst PE=1 SV=1 | Dlst | 0.92 |
| Q05910 | Disintegrin and metalloproteinase domain-containing protein 8 OS=Mus musculus OX=10090 GN=Adam8 PE=1 SV=3 | Adam8 | 0.92 |
| P62751 | 60S ribosomal protein L23a OS=Mus musculus OX=10090 GN=Rpl23a PE=1 SV=1 | Rpl23a | 0.92 |
| Q00651 | Integrin alpha-4 OS=Mus musculus OX=10090 GN=Itga4 PE=1 SV=1 | Itga4 | 0.92 |
| Q91W50 | Cold shock domain-containing protein E1 OS=Mus musculus OX=10090 GN=Csde1 PE=1 SV=1 | Csde1 | 0.92 |
| Q91XD6 | Vacuolar protein-sorting-associated protein 36 OS=Mus musculus OX=10090 GN=Vps36 PE=1 SV=1 | Vps36 | 0.92 |
| O35691 | Pinin OS=Mus musculus OX=10090 GN=Pnn PE=1 SV=4 | Pnn | 0.92 |
| Q8CDG3 | Deubiquitinating protein VCPIP1 OS=Mus musculus OX=10090 GN=Vcpip1 PE=1 SV=1 | Vcpip1 | 0.92 |
| Q9CYH6 | Ribosome biogenesis regulatory protein homolog OS=Mus musculus OX=10090 GN=Rrs1 PE=1 SV=1 | Rrs1 | 0.92 |
| Q9CWZ7 | Gamma-soluble NSF attachment protein OS=Mus musculus OX=10090 GN=Napg PE=1 SV=1 | Napg | 0.92 |
| Q922V4 | Pleiotropic regulator 1 OS=Mus musculus OX=10090 GN=Plrg1 PE=1 SV=1 | Plrg1 | 0.92 |
| P17809 | Solute carrier family 2, facilitated glucose transporter member 1 OS=Mus musculus OX=10090 GN=Slc2a1 PE=1 SV=4 | Slc2a1 | 0.92 |
| Q9CX97 | WD repeat-containing protein 55 OS=Mus musculus OX=10090 GN=Wdr55 PE=1 SV=2 | Wdr55 | 0.92 |
| Q9D1E6 | Tubulin-folding cofactor B OS=Mus musculus OX=10090 GN=Tbcb PE=1 SV=2 | Tbcb | 0.92 |
| P35278 | Ras-related protein Rab-5C OS=Mus musculus OX=10090 GN=Rab5c PE=1 SV=2 | Rab5c | 0.92 |
| Q9QZE5 | Coatomer subunit gamma-1 OS=Mus musculus OX=10090 GN=Copg1 PE=1 SV=1 | Copg1 | 0.92 |
| P40201 | Chromodomain-helicase-DNA-binding protein 1 OS=Mus musculus OX=10090 GN=Chd1 PE=1 SV=3 | Chd1 | 0.92 |
| O88829 | Lactosylceramide alpha-2,3-sialyltransferase OS=Mus musculus OX=10090 GN=St3gal5 PE=1 SV=2 | St3gal5 | 0.92 |
| Q9DC71 | 28S ribosomal protein S15, mitochondrial OS=Mus musculus OX=10090 GN=Mrps15 PE=1 SV=2 | Mrps15 | 0.92 |
| P17095 | High mobility group protein HMG-I/HMG-Y OS=Mus musculus OX=10090 GN=Hmga1 PE=1 SV=4 | Hmga1 | 0.92 |
| Q9JMH6 | Thioredoxin reductase 1, cytoplasmic OS=Mus musculus OX=10090 GN=Txnrd1 PE=1 SV=3 | Txnrd1 | 0.92 |
| Q8VDM6 | Heterogeneous nuclear ribonucleoprotein U-like protein 1 OS=Mus musculus OX=10090 GN=Hnrnpul1 PE=1 SV=1 | Hnrnpul1 | 0.92 |
| Q8BH79 | Anoctamin-10 OS=Mus musculus OX=10090 GN=Ano10 PE=1 SV=1 | Ano10 | 0.92 |
| Q9DBU3 | Serine/threonine-protein kinase RIO3 OS=Mus musculus OX=10090 GN=Riok3 PE=1 SV=3 | Riok3 | 0.92 |
| O70152 | Dolichol-phosphate mannosyltransferase subunit 1 OS=Mus musculus OX=10090 GN=Dpm1 PE=1 SV=1 | Dpm1 | 0.92 |
| P39688 | Tyrosine-protein kinase Fyn OS=Mus musculus OX=10090 GN=Fyn PE=1 SV=4 | Fyn | 0.92 |
| Q61171 | Peroxiredoxin-2 OS=Mus musculus OX=10090 GN=Prdx2 PE=1 SV=3 | Prdx2 | 0.92 |
| Q9DBJ1 | Phosphoglycerate mutase 1 OS=Mus musculus OX=10090 GN=Pgam1 PE=1 SV=3 | Pgam1 | 0.92 |
| Q8BG79 | CWF19-like protein 2 OS=Mus musculus OX=10090 GN=Cwf19l2 PE=1 SV=1 | Cwf19l2 | 0.92 |
| Q9CWW7 | CXXC-type zinc finger protein 1 OS=Mus musculus OX=10090 GN=Cxxc1 PE=1 SV=1 | Cxxc1 | 0.92 |
| Q8BJW5 | Nucleolar protein 11 OS=Mus musculus OX=10090 GN=Nol11 PE=2 SV=1 | Nol11 | 0.92 |
| Q9JLZ3 | Methylglutaconyl-CoA hydratase, mitochondrial OS=Mus musculus OX=10090 GN=Auh PE=1 SV=1 | Auh | 0.92 |
| Q8C436 | Protein N-lysine methyltransferase METTL21D OS=Mus musculus OX=10090 GN=Vcpkmt PE=1 SV=2 | Vcpkmt | 0.92 |
| A2APY7 | Arginine-hydroxylase NDUFAF5, mitochondrial OS=Mus musculus OX=10090 GN=Ndufaf5 PE=1 SV=1 | Ndufaf5 | 0.92 |
| Q08874 | Microphthalmia-associated transcription factor OS=Mus musculus OX=10090 GN=Mitf PE=1 SV=4 | Mitf | 0.92 |
| Q3TXS7 | 26S proteasome non-ATPase regulatory subunit 1 OS=Mus musculus OX=10090 GN=Psmd1 PE=1 SV=1 | Psmd1 | 0.92 |
| P10126 | Elongation factor 1-alpha 1 OS=Mus musculus OX=10090 GN=Eef1a1 PE=1 SV=3 | Eef1a1 | 0.92 |
| Q9CWU9 | Nucleoporin Nup37 OS=Mus musculus OX=10090 GN=Nup37 PE=1 SV=2 | Nup37 | 0.92 |
| Q8BIZ6 | Smad nuclear-interacting protein 1 OS=Mus musculus OX=10090 GN=Snip1 PE=1 SV=1 | Snip1 | 0.92 |
| Q9D7A8 | Armadillo repeat-containing protein 1 OS=Mus musculus OX=10090 GN=Armc1 PE=1 SV=1 | Armc1 | 0.92 |
| O88574 | Histone deacetylase complex subunit SAP30 OS=Mus musculus OX=10090 GN=Sap30 PE=1 SV=1 | Sap30 | 0.92 |
| Q9ERU9 | E3 SUMO-protein ligase RanBP2 OS=Mus musculus OX=10090 GN=Ranbp2 PE=1 SV=2 | Ranbp2 | 0.92 |
| Q9D6R2 | Isocitrate dehydrogenase [NAD] subunit alpha, mitochondrial OS=Mus musculus OX=10090 GN=Idh3a PE=1 SV=1 | Idh3a | 0.92 |
| Q69ZR2 | E3 ubiquitin-protein ligase HECTD1 OS=Mus musculus OX=10090 GN=Hectd1 PE=1 SV=2 | Hectd1 | 0.92 |
| Q93092 | Transaldolase OS=Mus musculus OX=10090 GN=Taldo1 PE=1 SV=2 | Taldo1 | 0.92 |
| Q64324 | Syntaxin-binding protein 2 OS=Mus musculus OX=10090 GN=Stxbp2 PE=1 SV=1 | Stxbp2 | 0.92 |
| Q6PAC3 | DDB1- and CUL4-associated factor 13 OS=Mus musculus OX=10090 GN=Dcaf13 PE=2 SV=2 | Dcaf13 | 0.93 |
| Q91ZX7 | Prolow-density lipoprotein receptor-related protein 1 OS=Mus musculus OX=10090 GN=Lrp1 PE=1 SV=1 | Lrp1 | 0.93 |
| Q9JHI5 | Isovaleryl-CoA dehydrogenase, mitochondrial OS=Mus musculus OX=10090 GN=Ivd PE=1 SV=1 | Ivd | 0.93 |
| P97821 | Dipeptidyl peptidase 1 OS=Mus musculus OX=10090 GN=Ctsc PE=1 SV=1 | Ctsc | 0.93 |
| P17918 | Proliferating cell nuclear antigen OS=Mus musculus OX=10090 GN=Pcna PE=1 SV=2 | Pcna | 0.93 |
| Q61941 | NAD(P) transhydrogenase, mitochondrial OS=Mus musculus OX=10090 GN=Nnt PE=1 SV=2 | Nnt | 0.93 |
| P61025 | Cyclin-dependent kinases regulatory subunit 1 OS=Mus musculus OX=10090 GN=Cks1b PE=3 SV=1 | Cks1b | 0.93 |
| Q63943 | Myocyte-specific enhancer factor 2D OS=Mus musculus OX=10090 GN=Mef2d PE=1 SV=2 | Mef2d | 0.93 |
| P12815 | Programmed cell death protein 6 OS=Mus musculus OX=10090 GN=Pdcd6 PE=1 SV=2 | Pdcd6 | 0.93 |
| Q9CQ22 | Ragulator complex protein LAMTOR1 OS=Mus musculus OX=10090 GN=Lamtor1 PE=1 SV=1 | Lamtor1 | 0.93 |
| Q9DB77 | Cytochrome b-c1 complex subunit 2, mitochondrial OS=Mus musculus OX=10090 GN=Uqcrc2 PE=1 SV=1 | Uqcrc2 | 0.93 |
| Q8BKE6 | Cytochrome P450 20A1 OS=Mus musculus OX=10090 GN=Cyp20a1 PE=1 SV=1 | Cyp20a1 | 0.93 |
| Q922S8 | Kinesin-like protein KIF2C OS=Mus musculus OX=10090 GN=Kif2c PE=1 SV=1 | Kif2c | 0.93 |
| Q8BVY0 | Ribosomal L1 domain-containing protein 1 OS=Mus musculus OX=10090 GN=Rsl1d1 PE=1 SV=1 | Rsl1d1 | 0.93 |
| Q9ERV1 | Probable E3 ubiquitin-protein ligase makorin-2 OS=Mus musculus OX=10090 GN=Mkrn2 PE=1 SV=2 | Mkrn2 | 0.93 |
| Q8CH18 | Cell division cycle and apoptosis regulator protein 1 OS=Mus musculus OX=10090 GN=Ccar1 PE=1 SV=1 | Ccar1 | 0.93 |
| Q61033 | Lamina-associated polypeptide 2, isoforms alpha/zeta OS=Mus musculus OX=10090 GN=Tmpo PE=1 SV=4 | Tmpo | 0.93 |
| Q3B7Z2 | Oxysterol-binding protein 1 OS=Mus musculus OX=10090 GN=Osbp PE=1 SV=3 | Osbp | 0.93 |
| Q9D2L9 | Serine protease FAM111A OS=Mus musculus OX=10090 GN=Fam111a PE=2 SV=1 | Fam111a | 0.93 |
| P56480 | ATP synthase subunit beta, mitochondrial OS=Mus musculus OX=10090 GN=Atp5f1b PE=1 SV=2 | Atp5f1b | 0.93 |
| Q9JHS4 | ATP-dependent Clp protease ATP-binding subunit clpX-like, mitochondrial OS=Mus musculus OX=10090 GN=Clpx PE=1 SV=2 | Clpx | 0.93 |
| Q9D7V9 | N-acylethanolamine-hydrolyzing acid amidase OS=Mus musculus OX=10090 GN=Naaa PE=1 SV=2 | Naaa | 0.93 |
| O88513 | Geminin OS=Mus musculus OX=10090 GN=Gmnn PE=1 SV=1 | Gmnn | 0.93 |
| P17182 | Alpha-enolase OS=Mus musculus OX=10090 GN=Eno1 PE=1 SV=3 | Eno1 | 0.93 |
| O54692 | Centromere/kinetochore protein zw10 homolog OS=Mus musculus OX=10090 GN=Zw10 PE=1 SV=3 | Zw10 | 0.93 |
| Q99K85 | Phosphoserine aminotransferase OS=Mus musculus OX=10090 GN=Psat1 PE=1 SV=1 | Psat1 | 0.93 |
| P08113 | Endoplasmin OS=Mus musculus OX=10090 GN=Hsp90b1 PE=1 SV=2 | Hsp90b1 | 0.93 |
| Q91W90 | Thioredoxin domain-containing protein 5 OS=Mus musculus OX=10090 GN=Txndc5 PE=1 SV=2 | Txndc5 | 0.93 |
| O88796 | Ribonuclease P protein subunit p30 OS=Mus musculus OX=10090 GN=Rpp30 PE=1 SV=1 | Rpp30 | 0.93 |
| Q9QZB7 | Actin-related protein 10 OS=Mus musculus OX=10090 GN=Actr10 PE=1 SV=2 | Actr10 | 0.93 |
| O08734 | Bcl-2 homologous antagonist/killer OS=Mus musculus OX=10090 GN=Bak1 PE=1 SV=3 | Bak1 | 0.93 |
| P56391 | Cytochrome c oxidase subunit 6B1 OS=Mus musculus OX=10090 GN=Cox6b1 PE=1 SV=2 | Cox6b1 | 0.93 |
| Q9D1P4 | Cysteine and histidine-rich domain-containing protein 1 OS=Mus musculus OX=10090 GN=Chordc1 PE=1 SV=1 | Chordc1 | 0.93 |
| Q8BU03 | Periodic tryptophan protein 2 homolog OS=Mus musculus OX=10090 GN=Pwp2 PE=1 SV=1 | Pwp2 | 0.93 |
| Q99LC9 | Peroxisome assembly factor 2 OS=Mus musculus OX=10090 GN=Pex6 PE=1 SV=1 | Pex6 | 0.93 |
| P70404 | Isocitrate dehydrogenase [NAD] subunit gamma 1, mitochondrial OS=Mus musculus OX=10090 GN=Idh3g PE=1 SV=1 | Idh3g | 0.93 |
| Q99M15 | Proline-serine-threonine phosphatase-interacting protein 2 OS=Mus musculus OX=10090 GN=Pstpip2 PE=1 SV=4 | Pstpip2 | 0.93 |
| P61087 | Ubiquitin-conjugating enzyme E2 K OS=Mus musculus OX=10090 GN=Ube2k PE=1 SV=3 | Ube2k | 0.93 |
| Q924C1 | Exportin-5 OS=Mus musculus OX=10090 GN=Xpo5 PE=1 SV=1 | Xpo5 | 0.93 |
| Q8BHS3 | Pre-mRNA-splicing factor RBM22 OS=Mus musculus OX=10090 GN=Rbm22 PE=1 SV=1 | Rbm22 | 0.93 |
| Q9JHF7 | Hematopoietic prostaglandin D synthase OS=Mus musculus OX=10090 GN=Hpgds PE=1 SV=3 | Hpgds | 0.93 |
| Q9Z103 | Activity-dependent neuroprotector homeobox protein OS=Mus musculus OX=10090 GN=Adnp PE=1 SV=2 | Adnp | 0.93 |
| Q5SXJ3 | Fanconi anemia group J protein homolog OS=Mus musculus OX=10090 GN=Brip1 PE=2 SV=1 | Brip1 | 0.93 |
| Q8BH15 | CCR4-NOT transcription complex subunit 10 OS=Mus musculus OX=10090 GN=Cnot10 PE=1 SV=1 | Cnot10 | 0.93 |
| Q6NSR8 | Probable aminopeptidase NPEPL1 OS=Mus musculus OX=10090 GN=Npepl1 PE=1 SV=1 | Npepl1 | 0.93 |
| Q9D5V5 | Cullin-5 OS=Mus musculus OX=10090 GN=Cul5 PE=1 SV=3 | Cul5 | 0.93 |
| Q9JIK5 | Nucleolar RNA helicase 2 OS=Mus musculus OX=10090 GN=Ddx21 PE=1 SV=3 | Ddx21 | 0.93 |
| P04184 | Thymidine kinase, cytosolic OS=Mus musculus OX=10090 GN=Tk1 PE=1 SV=3 | Tk1 | 0.93 |
| Q6ZQ58 | La-related protein 1 OS=Mus musculus OX=10090 GN=Larp1 PE=1 SV=3 | Larp1 | 0.93 |
| Q80YR7 | Claspin OS=Mus musculus OX=10090 GN=Clspn PE=1 SV=2 | Clspn | 0.93 |
| P97797 | Tyrosine-protein phosphatase non-receptor type substrate 1 OS=Mus musculus OX=10090 GN=Sirpa PE=1 SV=2 | Sirpa | 0.93 |
| Q9DCX2 | ATP synthase subunit d, mitochondrial OS=Mus musculus OX=10090 GN=Atp5pd PE=1 SV=3 | Atp5pd | 0.93 |
| Q9DCT1 | 1,5-anhydro-D-fructose reductase OS=Mus musculus OX=10090 GN=Akr1e2 PE=1 SV=1 | Akr1e2 | 0.93 |
| Q9DB85 | Ribosomal RNA-processing protein 8 OS=Mus musculus OX=10090 GN=Rrp8 PE=1 SV=1 | Rrp8 | 0.93 |
| Q9CZD3 | Glycine--tRNA ligase OS=Mus musculus OX=10090 GN=Gars1 PE=1 SV=1 | Gars1 | 0.93 |
| P67778 | Prohibitin OS=Mus musculus OX=10090 GN=Phb PE=1 SV=1 | Phb | 0.93 |
| P97807 | Fumarate hydratase, mitochondrial OS=Mus musculus OX=10090 GN=Fh PE=1 SV=3 | Fh | 0.93 |
| P62317 | Small nuclear ribonucleoprotein Sm D2 OS=Mus musculus OX=10090 GN=Snrpd2 PE=1 SV=1 | Snrpd2 | 0.93 |
| Q3TDN2 | FAS-associated factor 2 OS=Mus musculus OX=10090 GN=Faf2 PE=1 SV=2 | Faf2 | 0.93 |
| Q921J2 | GTP-binding protein Rheb OS=Mus musculus OX=10090 GN=Rheb PE=1 SV=1 | Rheb | 0.93 |
| Q9EP97 | Sentrin-specific protease 3 OS=Mus musculus OX=10090 GN=Senp3 PE=1 SV=1 | Senp3 | 0.93 |
| Q7TQK1 | Integrator complex subunit 7 OS=Mus musculus OX=10090 GN=Ints7 PE=1 SV=1 | Ints7 | 0.93 |
| P21550 | Beta-enolase OS=Mus musculus OX=10090 GN=Eno3 PE=1 SV=3 | Eno3 | 0.93 |
| P24788 | Cyclin-dependent kinase 11B OS=Mus musculus OX=10090 GN=Cdk11b PE=1 SV=2 | Cdk11b | 0.93 |
| Q6ZQL4 | WD repeat-containing protein 43 OS=Mus musculus OX=10090 GN=Wdr43 PE=1 SV=2 | Wdr43 | 0.93 |
| Q6PCN7 | Helicase-like transcription factor OS=Mus musculus OX=10090 GN=Hltf PE=1 SV=1 | Hltf | 0.93 |
| P68510 | 14-3-3 protein eta OS=Mus musculus OX=10090 GN=Ywhah PE=1 SV=2 | Ywhah | 0.93 |
| Q9CZL5 | Pterin-4-alpha-carbinolamine dehydratase 2 OS=Mus musculus OX=10090 GN=Pcbd2 PE=1 SV=2 | Pcbd2 | 0.93 |
| Q9JJY4 | Probable ATP-dependent RNA helicase DDX20 OS=Mus musculus OX=10090 GN=Ddx20 PE=1 SV=2 | Ddx20 | 0.93 |
| Q9D0I8 | mRNA turnover protein 4 homolog OS=Mus musculus OX=10090 GN=Mrto4 PE=1 SV=1 | Mrto4 | 0.93 |
| P18052 | Receptor-type tyrosine-protein phosphatase alpha OS=Mus musculus OX=10090 GN=Ptpra PE=1 SV=3 | Ptpra | 0.93 |
| O35598 | Disintegrin and metalloproteinase domain-containing protein 10 OS=Mus musculus OX=10090 GN=Adam10 PE=1 SV=2 | Adam10 | 0.93 |
| Q9JJT9 | Phosphorylated adapter RNA export protein OS=Mus musculus OX=10090 GN=Phax PE=1 SV=1 | Phax | 0.93 |
| O09106 | Histone deacetylase 1 OS=Mus musculus OX=10090 GN=Hdac1 PE=1 SV=1 | Hdac1 | 0.93 |
| Q61207 | Prosaposin OS=Mus musculus OX=10090 GN=Psap PE=1 SV=2 | Psap | 0.93 |
| Q9EQP2 | EH domain-containing protein 4 OS=Mus musculus OX=10090 GN=Ehd4 PE=1 SV=1 | Ehd4 | 0.93 |
| Q9WV32 | Actin-related protein 2/3 complex subunit 1B OS=Mus musculus OX=10090 GN=Arpc1b PE=1 SV=4 | Arpc1b | 0.93 |
| Q9CQA3 | Succinate dehydrogenase [ubiquinone] iron-sulfur subunit, mitochondrial OS=Mus musculus OX=10090 GN=Sdhb PE=1 SV=1 | Sdhb | 0.93 |
| Q8R4N0 | Citramalyl-CoA lyase, mitochondrial OS=Mus musculus OX=10090 GN=Clybl PE=1 SV=2 | Clybl | 0.93 |
| O09117 | Synaptophysin-like protein 1 OS=Mus musculus OX=10090 GN=Sypl1 PE=1 SV=2 | Sypl1 | 0.93 |
| P97376 | Protein FRG1 OS=Mus musculus OX=10090 GN=Frg1 PE=1 SV=2 | Frg1 | 0.93 |
| P30355 | Arachidonate 5-lipoxygenase-activating protein OS=Mus musculus OX=10090 GN=Alox5ap PE=1 SV=2 | Alox5ap | 0.93 |
| P70227 | Inositol 1,4,5-trisphosphate receptor type 3 OS=Mus musculus OX=10090 GN=Itpr3 PE=1 SV=3 | Itpr3 | 0.93 |
| Q9JHU9 | Inositol-3-phosphate synthase 1 OS=Mus musculus OX=10090 GN=Isyna1 PE=1 SV=1 | Isyna1 | 0.93 |
| Q9DB34 | Charged multivesicular body protein 2a OS=Mus musculus OX=10090 GN=Chmp2a PE=1 SV=1 | Chmp2a | 0.93 |
| P24547 | Inosine-5'-monophosphate dehydrogenase 2 OS=Mus musculus OX=10090 GN=Impdh2 PE=1 SV=2 | Impdh2 | 0.93 |
| Q9D818 | Suppressor APC domain-containing protein 2 OS=Mus musculus OX=10090 GN=Sapcd2 PE=1 SV=1 | Sapcd2 | 0.93 |
| Q9R0Q4 | Mortality factor 4-like protein 2 OS=Mus musculus OX=10090 GN=Morf4l2 PE=1 SV=1 | Morf4l2 | 0.93 |
| Q9CWX2 | Complex I intermediate-associated protein 30, mitochondrial OS=Mus musculus OX=10090 GN=Ndufaf1 PE=1 SV=2 | Ndufaf1 | 0.93 |
| Q9WU42 | Nuclear receptor corepressor 2 OS=Mus musculus OX=10090 GN=Ncor2 PE=1 SV=3 | Ncor2 | 0.93 |
| Q8CD92 | Tetratricopeptide repeat protein 27 OS=Mus musculus OX=10090 GN=Ttc27 PE=1 SV=2 | Ttc27 | 0.93 |
| Q8CFE3 | REST corepressor 1 OS=Mus musculus OX=10090 GN=Rcor1 PE=1 SV=3 | Rcor1 | 0.93 |
| P58021 | Transmembrane 9 superfamily member 2 OS=Mus musculus OX=10090 GN=Tm9sf2 PE=1 SV=1 | Tm9sf2 | 0.93 |
| Q61656 | Probable ATP-dependent RNA helicase DDX5 OS=Mus musculus OX=10090 GN=Ddx5 PE=1 SV=2 | Ddx5 | 0.93 |
| Q9EP69 | Phosphatidylinositol-3-phosphatase SAC1 OS=Mus musculus OX=10090 GN=Sacm1l PE=1 SV=1 | Sacm1l | 0.93 |
| Q00PI9 | Heterogeneous nuclear ribonucleoprotein U-like protein 2 OS=Mus musculus OX=10090 GN=Hnrnpul2 PE=1 SV=2 | Hnrnpul2 | 0.93 |
| Q9CXW4 | 60S ribosomal protein L11 OS=Mus musculus OX=10090 GN=Rpl11 PE=1 SV=4 | Rpl11 | 0.93 |
| B2RX14 | Terminal uridylyltransferase 4 OS=Mus musculus OX=10090 GN=Tut4 PE=1 SV=2 | Tut4 | 0.93 |
| Q9DB20 | ATP synthase subunit O, mitochondrial OS=Mus musculus OX=10090 GN=Atp5po PE=1 SV=1 | Atp5po | 0.93 |
| Q61036 | Serine/threonine-protein kinase PAK 3 OS=Mus musculus OX=10090 GN=Pak3 PE=1 SV=2 | Pak3 | 0.94 |
| Q8K4Q6 | Endonuclease 8-like 1 OS=Mus musculus OX=10090 GN=Neil1 PE=2 SV=3 | Neil1 | 0.94 |
| Q91X21 | Uncharacterized protein KIAA2013 OS=Mus musculus OX=10090 GN=Kiaa2013 PE=1 SV=1 | Kiaa2013 | 0.94 |
| Q9JLQ2 | ARF GTPase-activating protein GIT2 OS=Mus musculus OX=10090 GN=Git2 PE=1 SV=2 | Git2 | 0.94 |
| Q8R2K1 | Fucose mutarotase OS=Mus musculus OX=10090 GN=Fuom PE=1 SV=1 | Fuom | 0.94 |
| Q8C5H8 | NAD kinase 2, mitochondrial OS=Mus musculus OX=10090 GN=Nadk2 PE=1 SV=2 | Nadk2 | 0.94 |
| P07742 | Ribonucleoside-diphosphate reductase large subunit OS=Mus musculus OX=10090 GN=Rrm1 PE=1 SV=2 | Rrm1 | 0.94 |
| Q99LN9 | Deoxyhypusine hydroxylase OS=Mus musculus OX=10090 GN=Dohh PE=1 SV=2 | Dohh | 0.94 |
| Q6PAL0 | BEN domain-containing protein 3 OS=Mus musculus OX=10090 GN=Bend3 PE=1 SV=1 | Bend3 | 0.94 |
| P63085 | Mitogen-activated protein kinase 1 OS=Mus musculus OX=10090 GN=Mapk1 PE=1 SV=3 | Mapk1 | 0.94 |
| Q8BHB4 | WD repeat-containing protein 3 OS=Mus musculus OX=10090 GN=Wdr3 PE=1 SV=1 | Wdr3 | 0.94 |
| Q99P72 | Reticulon-4 OS=Mus musculus OX=10090 GN=Rtn4 PE=1 SV=2 | Rtn4 | 0.94 |
| Q8BLN5 | Lanosterol synthase OS=Mus musculus OX=10090 GN=Lss PE=1 SV=2 | Lss | 0.94 |
| Q8CGF7 | Transcription elongation regulator 1 OS=Mus musculus OX=10090 GN=Tcerg1 PE=1 SV=2 | Tcerg1 | 0.94 |
| P35550 | rRNA 2'-O-methyltransferase fibrillarin OS=Mus musculus OX=10090 GN=Fbl PE=1 SV=2 | Fbl | 0.94 |
| Q9QY06 | Unconventional myosin-IXb OS=Mus musculus OX=10090 GN=Myo9b PE=1 SV=2 | Myo9b | 0.94 |
| O70252 | Heme oxygenase 2 OS=Mus musculus OX=10090 GN=Hmox2 PE=1 SV=1 | Hmox2 | 0.94 |
| Q8R317 | Ubiquilin-1 OS=Mus musculus OX=10090 GN=Ubqln1 PE=1 SV=1 | Ubqln1 | 0.94 |
| Q8CHC4 | Synaptojanin-1 OS=Mus musculus OX=10090 GN=Synj1 PE=1 SV=3 | Synj1 | 0.94 |
| Q8R5K2 | Ubiquitin carboxyl-terminal hydrolase 33 OS=Mus musculus OX=10090 GN=Usp33 PE=1 SV=2 | Usp33 | 0.94 |
| Q5EBH1 | Ras association domain-containing protein 5 OS=Mus musculus OX=10090 GN=Rassf5 PE=1 SV=1 | Rassf5 | 0.94 |
| Q99K01 | Pyridoxal-dependent decarboxylase domain-containing protein 1 OS=Mus musculus OX=10090 GN=Pdxdc1 PE=1 SV=2 | Pdxdc1 | 0.94 |
| P01900 | H-2 class I histocompatibility antigen, D-D alpha chain OS=Mus musculus OX=10090 GN=H2-D1 PE=1 SV=1 | H2-D1 | 0.94 |
| Q04899 | Cyclin-dependent kinase 18 OS=Mus musculus OX=10090 GN=Cdk18 PE=1 SV=1 | Cdk18 | 0.94 |
| Q99KN9 | Clathrin interactor 1 OS=Mus musculus OX=10090 GN=Clint1 PE=1 SV=2 | Clint1 | 0.94 |
| Q8K2B3 | Succinate dehydrogenase [ubiquinone] flavoprotein subunit, mitochondrial OS=Mus musculus OX=10090 GN=Sdha PE=1 SV=1 | Sdha | 0.94 |
| Q6ZQJ5 | DNA replication ATP-dependent helicase/nuclease DNA2 OS=Mus musculus OX=10090 GN=Dna2 PE=1 SV=2 | Dna2 | 0.94 |
| Q9CQZ5 | NADH dehydrogenase [ubiquinone] 1 alpha subcomplex subunit 6 OS=Mus musculus OX=10090 GN=Ndufa6 PE=1 SV=1 | Ndufa6 | 0.94 |
| P97371 | Proteasome activator complex subunit 1 OS=Mus musculus OX=10090 GN=Psme1 PE=1 SV=2 | Psme1 | 0.94 |
| Q9DBD5 | Proline-, glutamic acid- and leucine-rich protein 1 OS=Mus musculus OX=10090 GN=Pelp1 PE=1 SV=2 | Pelp1 | 0.94 |
| Q8C176 | Transcription initiation factor TFIID subunit 2 OS=Mus musculus OX=10090 GN=Taf2 PE=2 SV=2 | Taf2 | 0.94 |
| P09581 | Macrophage colony-stimulating factor 1 receptor OS=Mus musculus OX=10090 GN=Csf1r PE=1 SV=3 | Csf1r | 0.94 |
| Q9D0K2 | Succinyl-CoA:3-ketoacid coenzyme A transferase 1, mitochondrial OS=Mus musculus OX=10090 GN=Oxct1 PE=1 SV=1 | Oxct1 | 0.94 |
| P83870 | PHD finger-like domain-containing protein 5A OS=Mus musculus OX=10090 GN=Phf5a PE=1 SV=1 | Phf5a | 0.94 |
| O35239 | Tyrosine-protein phosphatase non-receptor type 9 OS=Mus musculus OX=10090 GN=Ptpn9 PE=1 SV=2 | Ptpn9 | 0.94 |
| Q9DB27 | Malignant T-cell-amplified sequence 1 OS=Mus musculus OX=10090 GN=Mcts1 PE=1 SV=1 | Mcts1 | 0.94 |
| P08249 | Malate dehydrogenase, mitochondrial OS=Mus musculus OX=10090 GN=Mdh2 PE=1 SV=3 | Mdh2 | 0.94 |
| P62264 | 40S ribosomal protein S14 OS=Mus musculus OX=10090 GN=Rps14 PE=1 SV=3 | Rps14 | 0.94 |
| O35316 | Sodium- and chloride-dependent taurine transporter OS=Mus musculus OX=10090 GN=Slc6a6 PE=1 SV=2 | Slc6a6 | 0.94 |
| O88653 | Ragulator complex protein LAMTOR3 OS=Mus musculus OX=10090 GN=Lamtor3 PE=1 SV=1 | Lamtor3 | 0.94 |
| Q9CVI2 | Protein FAM133B OS=Mus musculus OX=10090 GN=Fam133b PE=1 SV=3 | Fam133b | 0.94 |
| Q8BMJ3 | Eukaryotic translation initiation factor 1A, X-chromosomal OS=Mus musculus OX=10090 GN=Eif1ax PE=2 SV=3 | Eif1ax | 0.94 |
| Q80X73 | Protein pelota homolog OS=Mus musculus OX=10090 GN=Pelo PE=1 SV=3 | Pelo | 0.94 |
| Q925I1 | ATPase family AAA domain-containing protein 3 OS=Mus musculus OX=10090 GN=Atad3 PE=1 SV=1 | Atad3 | 0.94 |
| Q9CQJ4 | E3 ubiquitin-protein ligase RING2 OS=Mus musculus OX=10090 GN=Rnf2 PE=1 SV=1 | Rnf2 | 0.94 |
| Q3V300 | Kinesin-like protein KIF22 OS=Mus musculus OX=10090 GN=Kif22 PE=2 SV=2 | Kif22 | 0.94 |
| Q80Y81 | Zinc phosphodiesterase ELAC protein 2 OS=Mus musculus OX=10090 GN=Elac2 PE=1 SV=1 | Elac2 | 0.94 |
| Q9R007 | C-type lectin domain family 5 member A OS=Mus musculus OX=10090 GN=Clec5a PE=1 SV=2 | Clec5a | 0.94 |
| P61804 | Dolichyl-diphosphooligosaccharide--protein glycosyltransferase subunit DAD1 OS=Mus musculus OX=10090 GN=Dad1 PE=1 SV=3 | Dad1 | 0.94 |
| Q9D5T0 | Outer mitochondrial transmembrane helix translocase OS=Mus musculus OX=10090 GN=Atad1 PE=1 SV=1 | Atad1 | 0.94 |
| Q9R061 | Cytosolic Fe-S cluster assembly factor NUBP2 OS=Mus musculus OX=10090 GN=Nubp2 PE=1 SV=1 | Nubp2 | 0.94 |
| P09925 | Surfeit locus protein 1 OS=Mus musculus OX=10090 GN=Surf1 PE=1 SV=3 | Surf1 | 0.94 |
| Q80UU1 | Ankyrin repeat and zinc finger domain-containing protein 1 OS=Mus musculus OX=10090 GN=Ankzf1 PE=1 SV=2 | Ankzf1 | 0.94 |
| Q8R3V5 | Endophilin-B2 OS=Mus musculus OX=10090 GN=Sh3glb2 PE=1 SV=2 | Sh3glb2 | 0.94 |
| Q9JLI8 | Squamous cell carcinoma antigen recognized by T-cells 3 OS=Mus musculus OX=10090 GN=Sart3 PE=1 SV=1 | Sart3 | 0.94 |
| Q6P5B0 | RRP12-like protein OS=Mus musculus OX=10090 GN=Rrp12 PE=1 SV=1 | Rrp12 | 0.94 |
| P51881 | ADP/ATP translocase 2 OS=Mus musculus OX=10090 GN=Slc25a5 PE=1 SV=3 | Slc25a5 | 0.94 |
| B1AUR6 | Protein MMS22-like OS=Mus musculus OX=10090 GN=Mms22l PE=1 SV=1 | Mms22l | 0.94 |
| P59999 | Actin-related protein 2/3 complex subunit 4 OS=Mus musculus OX=10090 GN=Arpc4 PE=1 SV=3 | Arpc4 | 0.94 |
| Q5SSI6 | U3 small nucleolar RNA-associated protein 18 homolog OS=Mus musculus OX=10090 GN=Utp18 PE=1 SV=1 | Utp18 | 0.94 |
| E9Q4Z2 | Acetyl-CoA carboxylase 2 OS=Mus musculus OX=10090 GN=Acacb PE=1 SV=1 | Acacb | 0.94 |
| Q61753 | D-3-phosphoglycerate dehydrogenase OS=Mus musculus OX=10090 GN=Phgdh PE=1 SV=3 | Phgdh | 0.94 |
| Q922H4 | Mannose-1-phosphate guanyltransferase alpha OS=Mus musculus OX=10090 GN=Gmppa PE=1 SV=1 | Gmppa | 0.94 |
| P70280 | Vesicle-associated membrane protein 7 OS=Mus musculus OX=10090 GN=Vamp7 PE=1 SV=1 | Vamp7 | 0.94 |
| O08789 | Max-binding protein MNT OS=Mus musculus OX=10090 GN=Mnt PE=2 SV=2 | Mnt | 0.94 |
| P62889 | 60S ribosomal protein L30 OS=Mus musculus OX=10090 GN=Rpl30 PE=1 SV=2 | Rpl30 | 0.94 |
| P62962 | Profilin-1 OS=Mus musculus OX=10090 GN=Pfn1 PE=1 SV=2 | Pfn1 | 0.94 |
| Q6ZWN5 | 40S ribosomal protein S9 OS=Mus musculus OX=10090 GN=Rps9 PE=1 SV=3 | Rps9 | 0.94 |
| Q9D554 | Splicing factor 3A subunit 3 OS=Mus musculus OX=10090 GN=Sf3a3 PE=1 SV=2 | Sf3a3 | 0.94 |
| Q8K224 | RNA cytidine acetyltransferase OS=Mus musculus OX=10090 GN=Nat10 PE=1 SV=1 | Nat10 | 0.94 |
| Q922H9 | Zinc finger protein 330 OS=Mus musculus OX=10090 GN=Znf330 PE=1 SV=1 | Znf330 | 0.94 |
| Q921X9 | Protein disulfide-isomerase A5 OS=Mus musculus OX=10090 GN=Pdia5 PE=1 SV=1 | Pdia5 | 0.94 |
| Q7TSC1 | Protein PRRC2A OS=Mus musculus OX=10090 GN=Prrc2a PE=1 SV=1 | Prrc2a | 0.94 |
| Q03347 | Runt-related transcription factor 1 OS=Mus musculus OX=10090 GN=Runx1 PE=1 SV=1 | Runx1 | 0.94 |
| Q8VD62 | UPF0696 protein C11orf68 homolog OS=Mus musculus OX=10090 GN=Bles03 PE=1 SV=2 | Bles03 | 0.94 |
| P70697 | Uroporphyrinogen decarboxylase OS=Mus musculus OX=10090 GN=Urod PE=1 SV=2 | Urod | 0.94 |
| Q9Z0M5 | Lysosomal acid lipase/cholesteryl ester hydrolase OS=Mus musculus OX=10090 GN=Lipa PE=1 SV=2 | Lipa | 0.94 |
| P63242 | Eukaryotic translation initiation factor 5A-1 OS=Mus musculus OX=10090 GN=Eif5a PE=1 SV=2 | Eif5a | 0.94 |
| Q9ESP1 | Stromal cell-derived factor 2-like protein 1 OS=Mus musculus OX=10090 GN=Sdf2l1 PE=1 SV=2 | Sdf2l1 | 0.94 |
| Q3TKT4 | Transcription activator BRG1 OS=Mus musculus OX=10090 GN=Smarca4 PE=1 SV=1 | Smarca4 | 0.94 |
| Q7TME2 | Sperm-associated antigen 5 OS=Mus musculus OX=10090 GN=Spag5 PE=1 SV=1 | Spag5 | 0.94 |
| Q9EPU4 | Cleavage and polyadenylation specificity factor subunit 1 OS=Mus musculus OX=10090 GN=Cpsf1 PE=1 SV=1 | Cpsf1 | 0.94 |
| P06795 | ATP-dependent translocase ABCB1 OS=Mus musculus OX=10090 GN=Abcb1b PE=1 SV=1 | Abcb1b | 0.94 |
| P97377 | Cyclin-dependent kinase 2 OS=Mus musculus OX=10090 GN=Cdk2 PE=1 SV=2 | Cdk2 | 0.94 |
| B2RY56 | RNA-binding protein 25 OS=Mus musculus OX=10090 GN=Rbm25 PE=1 SV=2 | Rbm25 | 0.94 |
| Q8BU88 | 39S ribosomal protein L22, mitochondrial OS=Mus musculus OX=10090 GN=Mrpl22 PE=1 SV=1 | Mrpl22 | 0.94 |
| Q9QY81 | Nuclear pore membrane glycoprotein 210 OS=Mus musculus OX=10090 GN=Nup210 PE=1 SV=2 | Nup210 | 0.94 |
| Q8BUK6 | Protein Hook homolog 3 OS=Mus musculus OX=10090 GN=Hook3 PE=1 SV=2 | Hook3 | 0.94 |
| P97492 | Regulator of G-protein signaling 14 OS=Mus musculus OX=10090 GN=Rgs14 PE=1 SV=2 | Rgs14 | 0.94 |
| Q62383 | Transcription elongation factor SPT6 OS=Mus musculus OX=10090 GN=Supt6h PE=1 SV=2 | Supt6h | 0.94 |
| Q9ERY9 | Ergosterol biosynthetic protein 28 homolog OS=Mus musculus OX=10090 GN=Erg28 PE=2 SV=1 | Erg28 | 0.94 |
| P07356 | Annexin A2 OS=Mus musculus OX=10090 GN=Anxa2 PE=1 SV=2 | Anxa2 | 0.94 |
| Q6ZPR5 | Sphingomyelin phosphodiesterase 4 OS=Mus musculus OX=10090 GN=Smpd4 PE=1 SV=2 | Smpd4 | 0.94 |
| Q9Z2Z6 | Mitochondrial carnitine/acylcarnitine carrier protein OS=Mus musculus OX=10090 GN=Slc25a20 PE=1 SV=1 | Slc25a20 | 0.94 |
| P43275 | Histone H1.1 OS=Mus musculus OX=10090 GN=H1-1 PE=1 SV=2 | H1-1 | 0.94 |
| Q91YR7 | Pre-mRNA-processing factor 6 OS=Mus musculus OX=10090 GN=Prpf6 PE=1 SV=1 | Prpf6 | 0.94 |
| Q9D1Q1 | M-phase phosphoprotein 6 OS=Mus musculus OX=10090 GN=Mphosph6 PE=1 SV=1 | Mphosph6 | 0.94 |
| Q9R1J0 | Sterol-4-alpha-carboxylate 3-dehydrogenase, decarboxylating OS=Mus musculus OX=10090 GN=Nsdhl PE=1 SV=1 | Nsdhl | 0.94 |
| Q921N6 | Probable ATP-dependent RNA helicase DDX27 OS=Mus musculus OX=10090 GN=Ddx27 PE=1 SV=3 | Ddx27 | 0.94 |
| Q3TCX3 | KH homology domain-containing protein 4 OS=Mus musculus OX=10090 GN=Khdc4 PE=1 SV=1 | Khdc4 | 0.94 |
| Q8BYH7 | TBC1 domain family member 17 OS=Mus musculus OX=10090 GN=Tbc1d17 PE=1 SV=2 | Tbc1d17 | 0.94 |
| Q9CWZ3 | RNA-binding protein 8A OS=Mus musculus OX=10090 GN=Rbm8a PE=1 SV=4 | Rbm8a | 0.94 |
| Q61164 | Transcriptional repressor CTCF OS=Mus musculus OX=10090 GN=Ctcf PE=1 SV=2 | Ctcf | 0.94 |
| P21958 | Antigen peptide transporter 1 OS=Mus musculus OX=10090 GN=Tap1 PE=1 SV=3 | Tap1 | 0.94 |
| Q8R3N1 | Nucleolar protein 14 OS=Mus musculus OX=10090 GN=Nop14 PE=1 SV=2 | Nop14 | 0.94 |
| G5E8V9 | Arfaptin-1 OS=Mus musculus OX=10090 GN=Arfip1 PE=1 SV=1 | Arfip1 | 0.94 |
| Q69ZN7 | Myoferlin OS=Mus musculus OX=10090 GN=Myof PE=1 SV=2 | Myof | 0.94 |
| O35704 | Serine palmitoyltransferase 1 OS=Mus musculus OX=10090 GN=Sptlc1 PE=1 SV=2 | Sptlc1 | 0.94 |
| Q05860 | Formin-1 OS=Mus musculus OX=10090 GN=Fmn1 PE=1 SV=2 | Fmn1 | 0.94 |
| Q8C0D5 | Elongation factor-like GTPase 1 OS=Mus musculus OX=10090 GN=Efl1 PE=1 SV=1 | Efl1 | 0.94 |
| Q9CPP0 | Nucleoplasmin-3 OS=Mus musculus OX=10090 GN=Npm3 PE=1 SV=3 | Npm3 | 0.94 |
| Q921C5 | Protein bicaudal D homolog 2 OS=Mus musculus OX=10090 GN=Bicd2 PE=1 SV=1 | Bicd2 | 0.94 |
| O35047 | Homologous-pairing protein 2 homolog OS=Mus musculus OX=10090 GN=Psmc3ip PE=1 SV=1 | Psmc3ip | 0.94 |
| Q3TL44 | NLR family member X1 OS=Mus musculus OX=10090 GN=Nlrx1 PE=1 SV=1 | Nlrx1 | 0.94 |
| Q9D287 | Pre-mRNA-splicing factor SPF27 OS=Mus musculus OX=10090 GN=Bcas2 PE=1 SV=1 | Bcas2 | 0.94 |
| Q8C3J5 | Dedicator of cytokinesis protein 2 OS=Mus musculus OX=10090 GN=Dock2 PE=1 SV=3 | Dock2 | 0.94 |
| Q8C4J7 | Transducin beta-like protein 3 OS=Mus musculus OX=10090 GN=Tbl3 PE=2 SV=1 | Tbl3 | 0.94 |
| Q5SUR0 | Phosphoribosylformylglycinamidine synthase OS=Mus musculus OX=10090 GN=Pfas PE=1 SV=1 | Pfas | 0.94 |
| Q80U58 | Pumilio homolog 2 OS=Mus musculus OX=10090 GN=Pum2 PE=1 SV=2 | Pum2 | 0.94 |
| Q6PAV2 | Probable E3 ubiquitin-protein ligase HERC4 OS=Mus musculus OX=10090 GN=Herc4 PE=1 SV=2 | Herc4 | 0.94 |
| P07091 | Protein S100-A4 OS=Mus musculus OX=10090 GN=S100a4 PE=1 SV=1 | S100a4 | 0.94 |
| Q9CR16 | Peptidyl-prolyl cis-trans isomerase D OS=Mus musculus OX=10090 GN=Ppid PE=1 SV=3 | Ppid | 0.95 |
| Q9WUR2 | Enoyl-CoA delta isomerase 2 OS=Mus musculus OX=10090 GN=Eci2 PE=1 SV=2 | Eci2 | 0.95 |
| Q9JJT0 | RNA 3'-terminal phosphate cyclase-like protein OS=Mus musculus OX=10090 GN=Rcl1 PE=2 SV=1 | Rcl1 | 0.95 |
| Q8C147 | Dedicator of cytokinesis protein 8 OS=Mus musculus OX=10090 GN=Dock8 PE=1 SV=4 | Dock8 | 0.95 |
| Q8BJ71 | Nuclear pore complex protein Nup93 OS=Mus musculus OX=10090 GN=Nup93 PE=1 SV=1 | Nup93 | 0.95 |
| Q99PT1 | Rho GDP-dissociation inhibitor 1 OS=Mus musculus OX=10090 GN=Arhgdia PE=1 SV=3 | Arhgdia | 0.95 |
| Q8BM39 | Pre-mRNA-splicing factor 18 OS=Mus musculus OX=10090 GN=Prpf18 PE=1 SV=1 | Prpf18 | 0.95 |
| Q9Z0D9 | CX3C chemokine receptor 1 OS=Mus musculus OX=10090 GN=Cx3cr1 PE=1 SV=1 | Cx3cr1 | 0.95 |
| Q9QXM1 | Junction-mediating and -regulatory protein OS=Mus musculus OX=10090 GN=Jmy PE=1 SV=1 | Jmy | 0.95 |
| Q9BCZ4 | Selenoprotein S OS=Mus musculus OX=10090 GN=Selenos PE=1 SV=3 | Selenos | 0.95 |
| Q7TSH6 | SR-related and CTD-associated factor 4 OS=Mus musculus OX=10090 GN=Scaf4 PE=1 SV=1 | Scaf4 | 0.95 |
| Q9CYZ6 | Required for excision 1-B domain-containing protein OS=Mus musculus OX=10090 GN=Rex1bd PE=1 SV=1 | Rex1bd | 0.95 |
| P13020 | Gelsolin OS=Mus musculus OX=10090 GN=Gsn PE=1 SV=3 | Gsn | 0.95 |
| Q8VDJ3 | Vigilin OS=Mus musculus OX=10090 GN=Hdlbp PE=1 SV=1 | Hdlbp | 0.95 |
| P45952 | Medium-chain specific acyl-CoA dehydrogenase, mitochondrial OS=Mus musculus OX=10090 GN=Acadm PE=1 SV=1 | Acadm | 0.95 |
| Q9CQR6 | Serine/threonine-protein phosphatase 6 catalytic subunit OS=Mus musculus OX=10090 GN=Ppp6c PE=1 SV=1 | Ppp6c | 0.95 |
| P11499 | Heat shock protein HSP 90-beta OS=Mus musculus OX=10090 GN=Hsp90ab1 PE=1 SV=3 | Hsp90ab1 | 0.95 |
| O70480 | Vesicle-associated membrane protein 4 OS=Mus musculus OX=10090 GN=Vamp4 PE=1 SV=1 | Vamp4 | 0.95 |
| P70460 | Vasodilator-stimulated phosphoprotein OS=Mus musculus OX=10090 GN=Vasp PE=1 SV=4 | Vasp | 0.95 |
| Q80TY0 | Formin-binding protein 1 OS=Mus musculus OX=10090 GN=Fnbp1 PE=1 SV=2 | Fnbp1 | 0.95 |
| Q61093 | Cytochrome b-245 heavy chain OS=Mus musculus OX=10090 GN=Cybb PE=1 SV=1 | Cybb | 0.95 |
| Q8BYH8 | Chromodomain-helicase-DNA-binding protein 9 OS=Mus musculus OX=10090 GN=Chd9 PE=1 SV=2 | Chd9 | 0.95 |
| P49717 | DNA replication licensing factor MCM4 OS=Mus musculus OX=10090 GN=Mcm4 PE=1 SV=1 | Mcm4 | 0.95 |
| P23475 | X-ray repair cross-complementing protein 6 OS=Mus musculus OX=10090 GN=Xrcc6 PE=1 SV=5 | Xrcc6 | 0.95 |
| Q5HZJ0 | Ribonuclease 3 OS=Mus musculus OX=10090 GN=Drosha PE=1 SV=1 | Drosha | 0.95 |
| Q9R1X4 | Protein timeless homolog OS=Mus musculus OX=10090 GN=Timeless PE=1 SV=3 | Timeless | 0.95 |
| Q8CFE2 | Histone PARylation factor 1 OS=Mus musculus OX=10090 GN=Hpf1 PE=1 SV=1 | Hpf1 | 0.95 |
| Q9JKX6 | ADP-sugar pyrophosphatase OS=Mus musculus OX=10090 GN=Nudt5 PE=1 SV=1 | Nudt5 | 0.95 |
| P70168 | Importin subunit beta-1 OS=Mus musculus OX=10090 GN=Kpnb1 PE=1 SV=2 | Kpnb1 | 0.95 |
| Q62261 | Spectrin beta chain, non-erythrocytic 1 OS=Mus musculus OX=10090 GN=Sptbn1 PE=1 SV=2 | Sptbn1 | 0.95 |
| P70677 | Caspase-3 OS=Mus musculus OX=10090 GN=Casp3 PE=1 SV=1 | Casp3 | 0.95 |
| Q80V26 | Golgi-resident adenosine 3',5'-bisphosphate 3'-phosphatase OS=Mus musculus OX=10090 GN=Bpnt2 PE=1 SV=1 | Bpnt2 | 0.95 |
| Q6ZPR6 | Inhibitor of Bruton tyrosine kinase OS=Mus musculus OX=10090 GN=Ibtk PE=1 SV=3 | Ibtk | 0.95 |
| P24063 | Integrin alpha-L OS=Mus musculus OX=10090 GN=Itgal PE=1 SV=2 | Itgal | 0.95 |
| Q8K3D3 | DNA repair protein SWI5 homolog OS=Mus musculus OX=10090 GN=Swi5 PE=1 SV=1 | Swi5 | 0.95 |
| Q62018 | RNA polymerase-associated protein CTR9 homolog OS=Mus musculus OX=10090 GN=Ctr9 PE=1 SV=2 | Ctr9 | 0.95 |
| Q8K072 | Receptor expression-enhancing protein 4 OS=Mus musculus OX=10090 GN=Reep4 PE=1 SV=1 | Reep4 | 0.95 |
| O88532 | Zinc finger RNA-binding protein OS=Mus musculus OX=10090 GN=Zfr PE=1 SV=2 | Zfr | 0.95 |
| Q9QYG0 | Protein NDRG2 OS=Mus musculus OX=10090 GN=Ndrg2 PE=1 SV=1 | Ndrg2 | 0.95 |
| E9Q9A9 | 2'-5'-oligoadenylate synthase 2 OS=Mus musculus OX=10090 GN=Oas2 PE=1 SV=1 | Oas2 | 0.95 |
| Q62192 | CD180 antigen OS=Mus musculus OX=10090 GN=Cd180 PE=1 SV=2 | Cd180 | 0.95 |
| Q9R059 | Four and a half LIM domains protein 3 OS=Mus musculus OX=10090 GN=Fhl3 PE=1 SV=2 | Fhl3 | 0.95 |
| P70288 | Histone deacetylase 2 OS=Mus musculus OX=10090 GN=Hdac2 PE=1 SV=1 | Hdac2 | 0.95 |
| P46638 | Ras-related protein Rab-11B OS=Mus musculus OX=10090 GN=Rab11b PE=1 SV=3 | Rab11b | 0.95 |
| P70388 | DNA repair protein RAD50 OS=Mus musculus OX=10090 GN=Rad50 PE=1 SV=1 | Rad50 | 0.95 |
| Q5SSZ5 | Tensin-3 OS=Mus musculus OX=10090 GN=Tns3 PE=1 SV=1 | Tns3 | 0.95 |
| Q8BIF9 | Zinc finger protein 787 OS=Mus musculus OX=10090 GN=Znf787 PE=2 SV=3 | Znf787 | 0.95 |
| Q924L1 | LETM1 domain-containing protein 1 OS=Mus musculus OX=10090 GN=Letmd1 PE=1 SV=1 | Letmd1 | 0.95 |
| Q78ZA7 | Nucleosome assembly protein 1-like 4 OS=Mus musculus OX=10090 GN=Nap1l4 PE=1 SV=1 | Nap1l4 | 0.95 |
| P58252 | Elongation factor 2 OS=Mus musculus OX=10090 GN=Eef2 PE=1 SV=2 | Eef2 | 0.95 |
| Q9JII6 | Aldo-keto reductase family 1 member A1 OS=Mus musculus OX=10090 GN=Akr1a1 PE=1 SV=3 | Akr1a1 | 0.95 |
| P81069 | GA-binding protein subunit beta-2 OS=Mus musculus OX=10090 GN=Gabpb2 PE=1 SV=2 | Gabpb2 | 0.95 |
| Q99KP6 | Pre-mRNA-processing factor 19 OS=Mus musculus OX=10090 GN=Prpf19 PE=1 SV=1 | Prpf19 | 0.95 |
| P47962 | 60S ribosomal protein L5 OS=Mus musculus OX=10090 GN=Rpl5 PE=1 SV=3 | Rpl5 | 0.95 |
| Q8CG72 | ADP-ribose glycohydrolase ARH3 OS=Mus musculus OX=10090 GN=Adprs PE=1 SV=1 | Adprs | 0.95 |
| Q9WVL2 | Signal transducer and activator of transcription 2 OS=Mus musculus OX=10090 GN=Stat2 PE=1 SV=1 | Stat2 | 0.95 |
| Q9CZN7 | Serine hydroxymethyltransferase, mitochondrial OS=Mus musculus OX=10090 GN=Shmt2 PE=1 SV=1 | Shmt2 | 0.95 |
| Q9CXI5 | Mesencephalic astrocyte-derived neurotrophic factor OS=Mus musculus OX=10090 GN=Manf PE=1 SV=1 | Manf | 0.95 |
| P62827 | GTP-binding nuclear protein Ran OS=Mus musculus OX=10090 GN=Ran PE=1 SV=3 | Ran | 0.95 |
| O88967 | ATP-dependent zinc metalloprotease YME1L1 OS=Mus musculus OX=10090 GN=Yme1l1 PE=1 SV=1 | Yme1l1 | 0.95 |
| Q8CFV9 | Riboflavin kinase OS=Mus musculus OX=10090 GN=Rfk PE=1 SV=2 | Rfk | 0.95 |
| Q9Z0V8 | Mitochondrial import inner membrane translocase subunit Tim17-A OS=Mus musculus OX=10090 GN=Timm17a PE=1 SV=1 | Timm17a | 0.95 |
| P16332 | Methylmalonyl-CoA mutase, mitochondrial OS=Mus musculus OX=10090 GN=Mmut PE=1 SV=2 | Mmut | 0.95 |
| P23780 | Beta-galactosidase OS=Mus musculus OX=10090 GN=Glb1 PE=1 SV=1 | Glb1 | 0.95 |
| Q3UE37 | Ubiquitin-conjugating enzyme E2 Z OS=Mus musculus OX=10090 GN=Ube2z PE=1 SV=2 | Ube2z | 0.95 |
| Q62446 | Peptidyl-prolyl cis-trans isomerase FKBP3 OS=Mus musculus OX=10090 GN=Fkbp3 PE=1 SV=2 | Fkbp3 | 0.95 |
| G3X9K3 | Brefeldin A-inhibited guanine nucleotide-exchange protein 1 OS=Mus musculus OX=10090 GN=Arfgef1 PE=1 SV=1 | Arfgef1 | 0.95 |
| P46978 | Dolichyl-diphosphooligosaccharide--protein glycosyltransferase subunit STT3A OS=Mus musculus OX=10090 GN=Stt3a PE=1 SV=1 | Stt3a | 0.95 |
| Q9D0B6 | Protein PBDC1 OS=Mus musculus OX=10090 GN=Pbdc1 PE=1 SV=1 | Pbdc1 | 0.95 |
| Q9CQV7 | Mitochondrial import inner membrane translocase subunit TIM14 OS=Mus musculus OX=10090 GN=Dnajc19 PE=1 SV=3 | Dnajc19 | 0.95 |
| Q9EQG9 | Ceramide transfer protein OS=Mus musculus OX=10090 GN=Cert1 PE=1 SV=1 | Cert1 | 0.95 |
| P70290 | 55 kDa erythrocyte membrane protein OS=Mus musculus OX=10090 GN=Mpp1 PE=1 SV=1 | Mpp1 | 0.95 |
| Q8BGT7 | Survival of motor neuron-related-splicing factor 30 OS=Mus musculus OX=10090 GN=Smndc1 PE=1 SV=1 | Smndc1 | 0.95 |
| Q99LB6 | Methionine adenosyltransferase 2 subunit beta OS=Mus musculus OX=10090 GN=Mat2b PE=1 SV=1 | Mat2b | 0.95 |
| Q66JV4 | RNA-binding protein 12B-B OS=Mus musculus OX=10090 GN=Rbm12b2 PE=2 SV=2 | Rbm12b2 | 0.95 |
| O08856 | RNA polymerase II elongation factor ELL OS=Mus musculus OX=10090 GN=Ell PE=2 SV=2 | Ell | 0.95 |
| Q8CG76 | Aflatoxin B1 aldehyde reductase member 2 OS=Mus musculus OX=10090 GN=Akr7a2 PE=1 SV=3 | Akr7a2 | 0.95 |
| P27048 | Small nuclear ribonucleoprotein-associated protein B OS=Mus musculus OX=10090 GN=Snrpb PE=1 SV=1 | Snrpb | 0.95 |
| Q99KF1 | Transmembrane emp24 domain-containing protein 9 OS=Mus musculus OX=10090 GN=Tmed9 PE=1 SV=2 | Tmed9 | 0.95 |
| Q9CRB2 | H/ACA ribonucleoprotein complex subunit 2 OS=Mus musculus OX=10090 GN=Nhp2 PE=1 SV=1 | Nhp2 | 0.95 |
| Q9D115 | Zinc finger protein 706 OS=Mus musculus OX=10090 GN=Znf706 PE=1 SV=1 | Znf706 | 0.95 |
| Q9QUN3 | B-cell linker protein OS=Mus musculus OX=10090 GN=Blnk PE=1 SV=1 | Blnk | 0.95 |
| P97386 | DNA ligase 3 OS=Mus musculus OX=10090 GN=Lig3 PE=1 SV=2 | Lig3 | 0.95 |
| Q9CPV4 | Glyoxalase domain-containing protein 4 OS=Mus musculus OX=10090 GN=Glod4 PE=1 SV=1 | Glod4 | 0.95 |
| Q99JY4 | TraB domain-containing protein OS=Mus musculus OX=10090 GN=Trabd PE=1 SV=1 | Trabd | 0.95 |
| Q8BIW1 | Exopolyphosphatase PRUNE1 OS=Mus musculus OX=10090 GN=Prune1 PE=1 SV=1 | Prune1 | 0.95 |
| Q8R1T1 | Charged multivesicular body protein 7 OS=Mus musculus OX=10090 GN=Chmp7 PE=1 SV=1 | Chmp7 | 0.95 |
| Q8BZH4 | Pogo transposable element with ZNF domain OS=Mus musculus OX=10090 GN=Pogz PE=1 SV=2 | Pogz | 0.95 |
| Q9D6H2 | Intraflagellar transport protein 25 homolog OS=Mus musculus OX=10090 GN=Hspb11 PE=1 SV=2 | Hspb11 | 0.95 |
| Q01853 | Transitional endoplasmic reticulum ATPase OS=Mus musculus OX=10090 GN=Vcp PE=1 SV=4 | Vcp | 0.95 |
| Q7TN29 | Stromal membrane-associated protein 2 OS=Mus musculus OX=10090 GN=Smap2 PE=1 SV=1 | Smap2 | 0.95 |
| Q60780 | Growth arrest-specific protein 7 OS=Mus musculus OX=10090 GN=Gas7 PE=1 SV=1 | Gas7 | 0.95 |
| Q8BGA5 | KRR1 small subunit processome component homolog OS=Mus musculus OX=10090 GN=Krr1 PE=2 SV=1 | Krr1 | 0.95 |
| Q8BP92 | Reticulocalbin-2 OS=Mus musculus OX=10090 GN=Rcn2 PE=1 SV=1 | Rcn2 | 0.95 |
| O70477 | Homeobox protein PKNOX1 OS=Mus musculus OX=10090 GN=Pknox1 PE=1 SV=3 | Pknox1 | 0.95 |
| Q91Z67 | SLIT-ROBO Rho GTPase-activating protein 2 OS=Mus musculus OX=10090 GN=Srgap2 PE=1 SV=2 | Srgap2 | 0.95 |
| Q8BHY2 | Nucleolar complex protein 4 homolog OS=Mus musculus OX=10090 GN=Noc4l PE=2 SV=1 | Noc4l | 0.95 |
| O88685 | 26S proteasome regulatory subunit 6A OS=Mus musculus OX=10090 GN=Psmc3 PE=1 SV=2 | Psmc3 | 0.95 |
| Q1HFZ0 | RNA cytosine C(5)-methyltransferase NSUN2 OS=Mus musculus OX=10090 GN=Nsun2 PE=1 SV=2 | Nsun2 | 0.95 |
| O35143 | ATPase inhibitor, mitochondrial OS=Mus musculus OX=10090 GN=Atp5if1 PE=1 SV=2 | Atp5if1 | 0.95 |
| Q9QZD4 | DNA repair endonuclease XPF OS=Mus musculus OX=10090 GN=Ercc4 PE=1 SV=3 | Ercc4 | 0.95 |
| P97364 | Selenide, water dikinase 2 OS=Mus musculus OX=10090 GN=Sephs2 PE=1 SV=3 | Sephs2 | 0.95 |
| Q8BHN3 | Neutral alpha-glucosidase AB OS=Mus musculus OX=10090 GN=Ganab PE=1 SV=1 | Ganab | 0.95 |
| P14069 | Protein S100-A6 OS=Mus musculus OX=10090 GN=S100a6 PE=1 SV=3 | S100a6 | 0.95 |
| O55135 | Eukaryotic translation initiation factor 6 OS=Mus musculus OX=10090 GN=Eif6 PE=1 SV=2 | Eif6 | 0.95 |
| Q9JHW4 | Selenocysteine-specific elongation factor OS=Mus musculus OX=10090 GN=Eefsec PE=1 SV=2 | Eefsec | 0.95 |
| Q9WUM4 | Coronin-1C OS=Mus musculus OX=10090 GN=Coro1c PE=1 SV=2 | Coro1c | 0.95 |
| Q8BT14 | CCR4-NOT transcription complex subunit 4 OS=Mus musculus OX=10090 GN=Cnot4 PE=1 SV=2 | Cnot4 | 0.95 |
| Q8BHD7 | Polypyrimidine tract-binding protein 3 OS=Mus musculus OX=10090 GN=Ptbp3 PE=1 SV=1 | Ptbp3 | 0.95 |
| Q6NZN0 | RNA-binding protein 26 OS=Mus musculus OX=10090 GN=Rbm26 PE=1 SV=2 | Rbm26 | 0.95 |
| Q99KK2 | N-acylneuraminate cytidylyltransferase OS=Mus musculus OX=10090 GN=Cmas PE=1 SV=2 | Cmas | 0.96 |
| Q9R0I7 | YLP motif-containing protein 1 OS=Mus musculus OX=10090 GN=Ylpm1 PE=2 SV=2 | Ylpm1 | 0.96 |
| O09061 | Proteasome subunit beta type-1 OS=Mus musculus OX=10090 GN=Psmb1 PE=1 SV=1 | Psmb1 | 0.96 |
| P61971 | Nuclear transport factor 2 OS=Mus musculus OX=10090 GN=Nutf2 PE=1 SV=1 | Nutf2 | 0.96 |
| Q9WV60 | Glycogen synthase kinase-3 beta OS=Mus musculus OX=10090 GN=Gsk3b PE=1 SV=2 | Gsk3b | 0.96 |
| Q9R117 | Non-receptor tyrosine-protein kinase TYK2 OS=Mus musculus OX=10090 GN=Tyk2 PE=1 SV=3 | Tyk2 | 0.96 |
| Q99LE6 | ATP-binding cassette sub-family F member 2 OS=Mus musculus OX=10090 GN=Abcf2 PE=1 SV=1 | Abcf2 | 0.96 |
| Q6NS46 | Protein RRP5 homolog OS=Mus musculus OX=10090 GN=Pdcd11 PE=1 SV=2 | Pdcd11 | 0.96 |
| P61620 | Protein transport protein Sec61 subunit alpha isoform 1 OS=Mus musculus OX=10090 GN=Sec61a1 PE=1 SV=2 | Sec61a1 | 0.96 |
| Q8K3X4 | Probable E3 ubiquitin-protein ligase IRF2BPL OS=Mus musculus OX=10090 GN=Irf2bpl PE=1 SV=1 | Irf2bpl | 0.96 |
| P17897 | Lysozyme C-1 OS=Mus musculus OX=10090 GN=Lyz1 PE=1 SV=1 | Lyz1 | 0.96 |
| Q9QWT9 | Kinesin-like protein KIFC1 OS=Mus musculus OX=10090 GN=Kifc1 PE=1 SV=2 | Kifc1 | 0.96 |
| Q61550 | Double-strand-break repair protein rad21 homolog OS=Mus musculus OX=10090 GN=Rad21 PE=1 SV=3 | Rad21 | 0.96 |
| Q9D8S4 | Oligoribonuclease, mitochondrial OS=Mus musculus OX=10090 GN=Rexo2 PE=1 SV=2 | Rexo2 | 0.96 |
| A2A6A1 | G patch domain-containing protein 8 OS=Mus musculus OX=10090 GN=Gpatch8 PE=1 SV=1 | Gpatch8 | 0.96 |
| Q8VE62 | Polyadenylate-binding protein-interacting protein 1 OS=Mus musculus OX=10090 GN=Paip1 PE=1 SV=1 | Paip1 | 0.96 |
| P38060 | Hydroxymethylglutaryl-CoA lyase, mitochondrial OS=Mus musculus OX=10090 GN=Hmgcl PE=1 SV=2 | Hmgcl | 0.96 |
| P61290 | Proteasome activator complex subunit 3 OS=Mus musculus OX=10090 GN=Psme3 PE=1 SV=1 | Psme3 | 0.96 |
| Q9D8T2 | Gasdermin-D OS=Mus musculus OX=10090 GN=Gsdmd PE=1 SV=1 | Gsdmd | 0.96 |
| Q9JLV5 | Cullin-3 OS=Mus musculus OX=10090 GN=Cul3 PE=1 SV=1 | Cul3 | 0.96 |
| Q8BHL8 | Proteasome inhibitor PI31 subunit OS=Mus musculus OX=10090 GN=Psmf1 PE=1 SV=1 | Psmf1 | 0.96 |
| O88466 | Zinc finger protein 106 OS=Mus musculus OX=10090 GN=Znf106 PE=1 SV=3 | Znf106 | 0.96 |
| Q8BTM8 | Filamin-A OS=Mus musculus OX=10090 GN=Flna PE=1 SV=5 | Flna | 0.96 |
| Q3V1T4 | Prolyl 3-hydroxylase 1 OS=Mus musculus OX=10090 GN=P3h1 PE=1 SV=2 | P3h1 | 0.96 |
| Q8VCG3 | WD repeat-containing protein 74 OS=Mus musculus OX=10090 GN=Wdr74 PE=2 SV=1 | Wdr74 | 0.96 |
| Q9EQH3 | Vacuolar protein sorting-associated protein 35 OS=Mus musculus OX=10090 GN=Vps35 PE=1 SV=1 | Vps35 | 0.96 |
| P26443 | Glutamate dehydrogenase 1, mitochondrial OS=Mus musculus OX=10090 GN=Glud1 PE=1 SV=1 | Glud1 | 0.96 |
| Q8R323 | Replication factor C subunit 3 OS=Mus musculus OX=10090 GN=Rfc3 PE=1 SV=1 | Rfc3 | 0.96 |
| P14733 | Lamin-B1 OS=Mus musculus OX=10090 GN=Lmnb1 PE=1 SV=3 | Lmnb1 | 0.96 |
| Q8CIN4 | Serine/threonine-protein kinase PAK 2 OS=Mus musculus OX=10090 GN=Pak2 PE=1 SV=1 | Pak2 | 0.96 |
| Q8BY71 | Histone acetyltransferase type B catalytic subunit OS=Mus musculus OX=10090 GN=Hat1 PE=1 SV=1 | Hat1 | 0.96 |
| Q91YH5 | Atlastin-3 OS=Mus musculus OX=10090 GN=Atl3 PE=1 SV=1 | Atl3 | 0.96 |
| Q8C8U0 | Liprin-beta-1 OS=Mus musculus OX=10090 GN=Ppfibp1 PE=1 SV=3 | Ppfibp1 | 0.96 |
| Q921M3 | Splicing factor 3B subunit 3 OS=Mus musculus OX=10090 GN=Sf3b3 PE=1 SV=1 | Sf3b3 | 0.96 |
| Q8BP67 | 60S ribosomal protein L24 OS=Mus musculus OX=10090 GN=Rpl24 PE=1 SV=2 | Rpl24 | 0.96 |
| Q3UJP5 | Cilia- and flagella-associated protein 418 OS=Mus musculus OX=10090 GN=Cfap418 PE=1 SV=1 | Cfap418 | 0.96 |
| Q8C854 | Myelin expression factor 2 OS=Mus musculus OX=10090 GN=Myef2 PE=1 SV=1 | Myef2 | 0.96 |
| Q3UFM5 | Nucleolar MIF4G domain-containing protein 1 OS=Mus musculus OX=10090 GN=Nom1 PE=1 SV=2 | Nom1 | 0.96 |
| Q80W00 | Serine/threonine-protein phosphatase 1 regulatory subunit 10 OS=Mus musculus OX=10090 GN=Ppp1r10 PE=1 SV=1 | Ppp1r10 | 0.96 |
| Q91WG2 | Rab GTPase-binding effector protein 2 OS=Mus musculus OX=10090 GN=Rabep2 PE=1 SV=3 | Rabep2 | 0.96 |
| P47753 | F-actin-capping protein subunit alpha-1 OS=Mus musculus OX=10090 GN=Capza1 PE=1 SV=4 | Capza1 | 0.96 |
| P14211 | Calreticulin OS=Mus musculus OX=10090 GN=Calr PE=1 SV=1 | Calr | 0.96 |
| Q9D517 | 1-acyl-sn-glycerol-3-phosphate acyltransferase gamma OS=Mus musculus OX=10090 GN=Agpat3 PE=1 SV=2 | Agpat3 | 0.96 |
| Q9CQN7 | 39S ribosomal protein L41, mitochondrial OS=Mus musculus OX=10090 GN=Mrpl41 PE=1 SV=1 | Mrpl41 | 0.96 |
| Q91VU0 | Protein FAM3C OS=Mus musculus OX=10090 GN=Fam3c PE=1 SV=1 | Fam3c | 0.96 |
| P51829 | Adenylate cyclase type 7 OS=Mus musculus OX=10090 GN=Adcy7 PE=1 SV=2 | Adcy7 | 0.96 |
| Q62280 | Protein SSXT OS=Mus musculus OX=10090 GN=Ss18 PE=1 SV=2 | Ss18 | 0.96 |
| Q9JJK2 | LanC-like protein 2 OS=Mus musculus OX=10090 GN=Lancl2 PE=1 SV=1 | Lancl2 | 0.96 |
| Q91VE6 | MKI67 FHA domain-interacting nucleolar phosphoprotein OS=Mus musculus OX=10090 GN=Nifk PE=1 SV=1 | Nifk | 0.96 |
| Q62418 | Drebrin-like protein OS=Mus musculus OX=10090 GN=Dbnl PE=1 SV=2 | Dbnl | 0.96 |
| P50544 | Very long-chain specific acyl-CoA dehydrogenase, mitochondrial OS=Mus musculus OX=10090 GN=Acadvl PE=1 SV=3 | Acadvl | 0.96 |
| P98195 | Probable phospholipid-transporting ATPase IIB OS=Mus musculus OX=10090 GN=Atp9b PE=1 SV=4 | Atp9b | 0.96 |
| Q9CQE5 | Regulator of G-protein signaling 10 OS=Mus musculus OX=10090 GN=Rgs10 PE=1 SV=1 | Rgs10 | 0.96 |
| Q9DC04 | Regulator of G-protein signaling 3 OS=Mus musculus OX=10090 GN=Rgs3 PE=1 SV=2 | Rgs3 | 0.96 |
| O54782 | Epididymis-specific alpha-mannosidase OS=Mus musculus OX=10090 GN=Man2b2 PE=1 SV=2 | Man2b2 | 0.96 |
| Q8R409 | Protein HEXIM1 OS=Mus musculus OX=10090 GN=Hexim1 PE=1 SV=1 | Hexim1 | 0.96 |
| P42227 | Signal transducer and activator of transcription 3 OS=Mus musculus OX=10090 GN=Stat3 PE=1 SV=2 | Stat3 | 0.96 |
| Q3UDR8 | Protein YIPF3 OS=Mus musculus OX=10090 GN=Yipf3 PE=1 SV=1 | Yipf3 | 0.96 |
| Q9WUQ2 | Prolactin regulatory element-binding protein OS=Mus musculus OX=10090 GN=Preb PE=1 SV=1 | Preb | 0.96 |
| Q9JIK9 | 28S ribosomal protein S34, mitochondrial OS=Mus musculus OX=10090 GN=Mrps34 PE=1 SV=1 | Mrps34 | 0.96 |
| Q9D2D7 | Zinc finger protein 687 OS=Mus musculus OX=10090 GN=Znf687 PE=1 SV=1 | Znf687 | 0.96 |
| Q9CR47 | Ribosome biogenesis protein NSA2 homolog OS=Mus musculus OX=10090 GN=Nsa2 PE=2 SV=1 | Nsa2 | 0.96 |
| Q80X80 | Phospholipid transfer protein C2CD2L OS=Mus musculus OX=10090 GN=C2cd2l PE=1 SV=3 | C2cd2l | 0.96 |
| Q9QY36 | N-alpha-acetyltransferase 10 OS=Mus musculus OX=10090 GN=Naa10 PE=1 SV=1 | Naa10 | 0.96 |
| Q9D1P0 | 39S ribosomal protein L13, mitochondrial OS=Mus musculus OX=10090 GN=Mrpl13 PE=1 SV=1 | Mrpl13 | 0.96 |
| P06151 | L-lactate dehydrogenase A chain OS=Mus musculus OX=10090 GN=Ldha PE=1 SV=3 | Ldha | 0.96 |
| Q9WTX6 | Cullin-1 OS=Mus musculus OX=10090 GN=Cul1 PE=1 SV=1 | Cul1 | 0.96 |
| Q7TSV4 | Phosphoglucomutase-2 OS=Mus musculus OX=10090 GN=Pgm2 PE=1 SV=1 | Pgm2 | 0.96 |
| O35638 | Cohesin subunit SA-2 OS=Mus musculus OX=10090 GN=Stag2 PE=1 SV=3 | Stag2 | 0.96 |
| P09103 | Protein disulfide-isomerase OS=Mus musculus OX=10090 GN=P4hb PE=1 SV=2 | P4hb | 0.96 |
| O55142 | 60S ribosomal protein L35a OS=Mus musculus OX=10090 GN=Rpl35a PE=1 SV=2 | Rpl35a | 0.96 |
| O35855 | Branched-chain-amino-acid aminotransferase, mitochondrial OS=Mus musculus OX=10090 GN=Bcat2 PE=1 SV=2 | Bcat2 | 0.96 |
| Q9CQZ6 | NADH dehydrogenase [ubiquinone] 1 beta subcomplex subunit 3 OS=Mus musculus OX=10090 GN=Ndufb3 PE=1 SV=1 | Ndufb3 | 0.96 |
| Q99NB8 | Ubiquilin-4 OS=Mus musculus OX=10090 GN=Ubqln4 PE=1 SV=1 | Ubqln4 | 0.96 |
| Q9R1B9 | Slit homolog 2 protein OS=Mus musculus OX=10090 GN=Slit2 PE=2 SV=2 | Slit2 | 0.96 |
| Q9JK23 | Proteasome assembly chaperone 1 OS=Mus musculus OX=10090 GN=Psmg1 PE=1 SV=1 | Psmg1 | 0.96 |
| Q4KWH5 | 1-phosphatidylinositol 4,5-bisphosphate phosphodiesterase eta-1 OS=Mus musculus OX=10090 GN=Plch1 PE=2 SV=1 | Plch1 | 0.96 |
| Q9CU65 | Zinc finger MYM-type protein 2 OS=Mus musculus OX=10090 GN=Zmym2 PE=1 SV=3 | Zmym2 | 0.96 |
| P22907 | Porphobilinogen deaminase OS=Mus musculus OX=10090 GN=Hmbs PE=1 SV=2 | Hmbs | 0.96 |
| Q80TL7 | Protein MON2 homolog OS=Mus musculus OX=10090 GN=Mon2 PE=1 SV=2 | Mon2 | 0.96 |
| P51859 | Hepatoma-derived growth factor OS=Mus musculus OX=10090 GN=Hdgf PE=1 SV=2 | Hdgf | 0.96 |
| P59759 | Myocardin-related transcription factor B OS=Mus musculus OX=10090 GN=Mrtfb PE=1 SV=1 | Mrtfb | 0.96 |
| Q6P069 | Sorcin OS=Mus musculus OX=10090 GN=Sri PE=1 SV=1 | Sri | 0.96 |
| Q9CXW3 | Calcyclin-binding protein OS=Mus musculus OX=10090 GN=Cacybp PE=1 SV=1 | Cacybp | 0.96 |
| Q9CQC6 | Basic leucine zipper and W2 domain-containing protein 1 OS=Mus musculus OX=10090 GN=Bzw1 PE=1 SV=1 | Bzw1 | 0.96 |
| Q9Z210 | Peroxisomal membrane protein 11B OS=Mus musculus OX=10090 GN=Pex11b PE=1 SV=1 | Pex11b | 0.96 |
| Q8BX10 | Serine/threonine-protein phosphatase PGAM5, mitochondrial OS=Mus musculus OX=10090 GN=Pgam5 PE=1 SV=1 | Pgam5 | 0.96 |
| Q5XG71 | Small subunit processome component 20 homolog OS=Mus musculus OX=10090 GN=Utp20 PE=1 SV=2 | Utp20 | 0.96 |
| O88342 | WD repeat-containing protein 1 OS=Mus musculus OX=10090 GN=Wdr1 PE=1 SV=3 | Wdr1 | 0.96 |
| Q3UBG2 | PTB-containing, cubilin and LRP1-interacting protein OS=Mus musculus OX=10090 GN=Pid1 PE=1 SV=2 | Pid1 | 0.96 |
| Q9D289 | Trafficking protein particle complex subunit 6B OS=Mus musculus OX=10090 GN=Trappc6b PE=1 SV=1 | Trappc6b | 0.96 |
| Q9D773 | 39S ribosomal protein L2, mitochondrial OS=Mus musculus OX=10090 GN=Mrpl2 PE=1 SV=1 | Mrpl2 | 0.96 |
| P16110 | Galectin-3 OS=Mus musculus OX=10090 GN=Lgals3 PE=1 SV=3 | Lgals3 | 0.96 |
| A2AN08 | E3 ubiquitin-protein ligase UBR4 OS=Mus musculus OX=10090 GN=Ubr4 PE=1 SV=1 | Ubr4 | 0.96 |
| Q91UZ5 | Inositol monophosphatase 2 OS=Mus musculus OX=10090 GN=Impa2 PE=1 SV=1 | Impa2 | 0.96 |
| Q91VY9 | Zinc finger protein 622 OS=Mus musculus OX=10090 GN=Znf622 PE=1 SV=1 | Znf622 | 0.96 |
| P11983 | T-complex protein 1 subunit alpha OS=Mus musculus OX=10090 GN=Tcp1 PE=1 SV=3 | Tcp1 | 0.96 |
| Q505F5 | Leucine-rich repeat-containing protein 47 OS=Mus musculus OX=10090 GN=Lrrc47 PE=1 SV=1 | Lrrc47 | 0.96 |
| Q8K124 | Pleckstrin homology domain-containing family O member 2 OS=Mus musculus OX=10090 GN=Plekho2 PE=1 SV=1 | Plekho2 | 0.96 |
| Q8BPU7 | Engulfment and cell motility protein 1 OS=Mus musculus OX=10090 GN=Elmo1 PE=1 SV=2 | Elmo1 | 0.96 |
| Q9WUU7 | Cathepsin Z OS=Mus musculus OX=10090 GN=Ctsz PE=1 SV=1 | Ctsz | 0.96 |
| Q5U5Q9 | BRCA1-A complex subunit RAP80 OS=Mus musculus OX=10090 GN=Uimc1 PE=1 SV=2 | Uimc1 | 0.96 |
| Q922E6 | FAST kinase domain-containing protein 2, mitochondrial OS=Mus musculus OX=10090 GN=Fastkd2 PE=2 SV=2 | Fastkd2 | 0.96 |
| Q7TN31 | Angiogenic factor with G patch and FHA domains 1 OS=Mus musculus OX=10090 GN=Aggf1 PE=2 SV=1 | Aggf1 | 0.96 |
| P39054 | Dynamin-2 OS=Mus musculus OX=10090 GN=Dnm2 PE=1 SV=2 | Dnm2 | 0.96 |
| P80317 | T-complex protein 1 subunit zeta OS=Mus musculus OX=10090 GN=Cct6a PE=1 SV=3 | Cct6a | 0.96 |
| Q99LH1 | Nucleolar GTP-binding protein 2 OS=Mus musculus OX=10090 GN=Gnl2 PE=1 SV=2 | Gnl2 | 0.96 |
| Q9CQ92 | Mitochondrial fission 1 protein OS=Mus musculus OX=10090 GN=Fis1 PE=1 SV=1 | Fis1 | 0.96 |
| Q9DBR0 | A-kinase anchor protein 8 OS=Mus musculus OX=10090 GN=Akap8 PE=1 SV=1 | Akap8 | 0.96 |
| Q9R1C7 | Pre-mRNA-processing factor 40 homolog A OS=Mus musculus OX=10090 GN=Prpf40a PE=1 SV=1 | Prpf40a | 0.96 |
| Q6PGF3 | Mediator of RNA polymerase II transcription subunit 16 OS=Mus musculus OX=10090 GN=Med16 PE=1 SV=2 | Med16 | 0.96 |
| Q9Z2X1 | Heterogeneous nuclear ribonucleoprotein F OS=Mus musculus OX=10090 GN=Hnrnpf PE=1 SV=3 | Hnrnpf | 0.96 |
| Q8BL97 | Serine/arginine-rich splicing factor 7 OS=Mus musculus OX=10090 GN=Srsf7 PE=1 SV=1 | Srsf7 | 0.96 |
| O55091 | Protein IMPACT OS=Mus musculus OX=10090 GN=Impact PE=1 SV=2 | Impact | 0.96 |
| Q9Z2Y8 | Pyridoxal phosphate homeostasis protein OS=Mus musculus OX=10090 GN=Plpbp PE=1 SV=1 | Plpbp | 0.96 |
| Q8CD15 | Ribosomal oxygenase 2 OS=Mus musculus OX=10090 GN=Riox2 PE=1 SV=2 | Riox2 | 0.96 |
| Q6PDQ2 | Chromodomain-helicase-DNA-binding protein 4 OS=Mus musculus OX=10090 GN=Chd4 PE=1 SV=1 | Chd4 | 0.96 |
| Q9D8B4 | NADH dehydrogenase [ubiquinone] 1 alpha subcomplex subunit 11 OS=Mus musculus OX=10090 GN=Ndufa11 PE=1 SV=2 | Ndufa11 | 0.96 |
| Q9CQT5 | Proteasome maturation protein OS=Mus musculus OX=10090 GN=Pomp PE=1 SV=1 | Pomp | 0.96 |
| O88712 | C-terminal-binding protein 1 OS=Mus musculus OX=10090 GN=Ctbp1 PE=1 SV=2 | Ctbp1 | 0.96 |
| Q61838 | Pregnancy zone protein OS=Mus musculus OX=10090 GN=Pzp PE=1 SV=3 | Pzp | 0.96 |
| Q8VHK9 | ATP-dependent DNA/RNA helicase DHX36 OS=Mus musculus OX=10090 GN=Dhx36 PE=1 SV=2 | Dhx36 | 0.96 |
| P58462 | Forkhead box protein P1 OS=Mus musculus OX=10090 GN=Foxp1 PE=1 SV=1 | Foxp1 | 0.96 |
| O08807 | Peroxiredoxin-4 OS=Mus musculus OX=10090 GN=Prdx4 PE=1 SV=1 | Prdx4 | 0.96 |
| Q69ZX6 | ATPase MORC2A OS=Mus musculus OX=10090 GN=Morc2a PE=1 SV=2 | Morc2a | 0.96 |
| Q99ME9 | GTP-binding protein 4 OS=Mus musculus OX=10090 GN=Gtpbp4 PE=1 SV=3 | Gtpbp4 | 0.96 |
| Q99LG2 | Transportin-2 OS=Mus musculus OX=10090 GN=Tnpo2 PE=1 SV=1 | Tnpo2 | 0.96 |
| P35979 | 60S ribosomal protein L12 OS=Mus musculus OX=10090 GN=Rpl12 PE=1 SV=2 | Rpl12 | 0.96 |
| Q60739 | BAG family molecular chaperone regulator 1 OS=Mus musculus OX=10090 GN=Bag1 PE=1 SV=3 | Bag1 | 0.96 |
| Q8CFQ3 | RNA helicase aquarius OS=Mus musculus OX=10090 GN=Aqr PE=1 SV=2 | Aqr | 0.97 |
| Q9EPE9 | Endoplasmic reticulum transmembrane helix translocase OS=Mus musculus OX=10090 GN=Atp13a1 PE=1 SV=2 | Atp13a1 | 0.97 |
| Q60692 | Proteasome subunit beta type-6 OS=Mus musculus OX=10090 GN=Psmb6 PE=1 SV=3 | Psmb6 | 0.97 |
| P54823 | Probable ATP-dependent RNA helicase DDX6 OS=Mus musculus OX=10090 GN=Ddx6 PE=1 SV=1 | Ddx6 | 0.97 |
| Q61510 | E3 ubiquitin/ISG15 ligase TRIM25 OS=Mus musculus OX=10090 GN=Trim25 PE=1 SV=2 | Trim25 | 0.97 |
| Q76MZ3 | Serine/threonine-protein phosphatase 2A 65 kDa regulatory subunit A alpha isoform OS=Mus musculus OX=10090 GN=Ppp2r1a PE=1 SV=3 | Ppp2r1a | 0.97 |
| Q5SFM8 | RNA-binding protein 27 OS=Mus musculus OX=10090 GN=Rbm27 PE=1 SV=3 | Rbm27 | 0.97 |
| Q8BTY8 | Sec1 family domain-containing protein 2 OS=Mus musculus OX=10090 GN=Scfd2 PE=1 SV=1 | Scfd2 | 0.97 |
| Q8CFI7 | DNA-directed RNA polymerase II subunit RPB2 OS=Mus musculus OX=10090 GN=Polr2b PE=1 SV=2 | Polr2b | 0.97 |
| Q8BJU0 | Small glutamine-rich tetratricopeptide repeat-containing protein alpha OS=Mus musculus OX=10090 GN=Sgta PE=1 SV=2 | Sgta | 0.97 |
| Q8BMC4 | Nucleolar protein 9 OS=Mus musculus OX=10090 GN=Nop9 PE=1 SV=1 | Nop9 | 0.97 |
| P62911 | 60S ribosomal protein L32 OS=Mus musculus OX=10090 GN=Rpl32 PE=1 SV=2 | Rpl32 | 0.97 |
| Q62426 | Cystatin-B OS=Mus musculus OX=10090 GN=Cstb PE=1 SV=1 | Cstb | 0.97 |
| Q5SU73 | Coilin OS=Mus musculus OX=10090 GN=Coil PE=1 SV=1 | Coil | 0.97 |
| P80313 | T-complex protein 1 subunit eta OS=Mus musculus OX=10090 GN=Cct7 PE=1 SV=1 | Cct7 | 0.97 |
| Q7TPR4 | Alpha-actinin-1 OS=Mus musculus OX=10090 GN=Actn1 PE=1 SV=1 | Actn1 | 0.97 |
| Q8R0J7 | Vacuolar protein sorting-associated protein 37B OS=Mus musculus OX=10090 GN=Vps37b PE=1 SV=1 | Vps37b | 0.97 |
| Q9R1Q9 | V-type proton ATPase subunit S1 OS=Mus musculus OX=10090 GN=Atp6ap1 PE=1 SV=1 | Atp6ap1 | 0.97 |
| Q6P8M1 | Putative deoxyribonuclease TATDN1 OS=Mus musculus OX=10090 GN=Tatdn1 PE=1 SV=1 | Tatdn1 | 0.97 |
| P62814 | V-type proton ATPase subunit B, brain isoform OS=Mus musculus OX=10090 GN=Atp6v1b2 PE=1 SV=1 | Atp6v1b2 | 0.97 |
| O35344 | Importin subunit alpha-4 OS=Mus musculus OX=10090 GN=Kpna3 PE=1 SV=1 | Kpna3 | 0.97 |
| Q91Z38 | Tetratricopeptide repeat protein 1 OS=Mus musculus OX=10090 GN=Ttc1 PE=1 SV=1 | Ttc1 | 0.97 |
| Q62086 | Serum paraoxonase/arylesterase 2 OS=Mus musculus OX=10090 GN=Pon2 PE=1 SV=2 | Pon2 | 0.97 |
| Q8BKC8 | Phosphatidylinositol 4-kinase beta OS=Mus musculus OX=10090 GN=Pi4kb PE=1 SV=2 | Pi4kb | 0.97 |
| P58468 | Ribosome biogenesis protein SLX9 homolog OS=Mus musculus OX=10090 GN=Slx9 PE=1 SV=1 | Slx9 | 0.97 |
| Q3U0M1 | Trafficking protein particle complex subunit 9 OS=Mus musculus OX=10090 GN=Trappc9 PE=1 SV=2 | Trappc9 | 0.97 |
| Q8BFY6 | Peflin OS=Mus musculus OX=10090 GN=Pef1 PE=1 SV=1 | Pef1 | 0.97 |
| Q9ERH4 | Nucleolar and spindle-associated protein 1 OS=Mus musculus OX=10090 GN=Nusap1 PE=1 SV=1 | Nusap1 | 0.97 |
| Q9R0Q3 | Transmembrane emp24 domain-containing protein 2 OS=Mus musculus OX=10090 GN=Tmed2 PE=1 SV=1 | Tmed2 | 0.97 |
| Q80UK8 | Integrator complex subunit 2 OS=Mus musculus OX=10090 GN=Ints2 PE=1 SV=2 | Ints2 | 0.97 |
| Q60960 | Importin subunit alpha-5 OS=Mus musculus OX=10090 GN=Kpna1 PE=1 SV=2 | Kpna1 | 0.97 |
| Q80TN5 | Palmitoyltransferase ZDHHC17 OS=Mus musculus OX=10090 GN=Zdhhc17 PE=1 SV=2 | Zdhhc17 | 0.97 |
| P50518 | V-type proton ATPase subunit E 1 OS=Mus musculus OX=10090 GN=Atp6v1e1 PE=1 SV=2 | Atp6v1e1 | 0.97 |
| P59808 | SAM and SH3 domain-containing protein 1 OS=Mus musculus OX=10090 GN=Sash1 PE=1 SV=1 | Sash1 | 0.97 |
| Q9D7E3 | Esterase OVCA2 OS=Mus musculus OX=10090 GN=Ovca2 PE=1 SV=1 | Ovca2 | 0.97 |
| Q61183 | Poly(A) polymerase alpha OS=Mus musculus OX=10090 GN=Papola PE=1 SV=4 | Papola | 0.97 |
| O88845 | A-kinase anchor protein 10, mitochondrial OS=Mus musculus OX=10090 GN=Akap10 PE=1 SV=3 | Akap10 | 0.97 |
| Q05512 | Serine/threonine-protein kinase MARK2 OS=Mus musculus OX=10090 GN=Mark2 PE=1 SV=3 | Mark2 | 0.97 |
| Q9CQX4 | PCNA-associated factor OS=Mus musculus OX=10090 GN=Pclaf PE=1 SV=1 | Pclaf | 0.97 |
| Q8BG32 | 26S proteasome non-ATPase regulatory subunit 11 OS=Mus musculus OX=10090 GN=Psmd11 PE=1 SV=3 | Psmd11 | 0.97 |
| Q6ZWZ2 | Ubiquitin-conjugating enzyme E2 R2 OS=Mus musculus OX=10090 GN=Ube2r2 PE=1 SV=1 | Ube2r2 | 0.97 |
| Q8BG17 | Nucleolar protein 12 OS=Mus musculus OX=10090 GN=Nol12 PE=1 SV=1 | Nol12 | 0.97 |
| Q8BTU1 | Cilia- and flagella-associated protein 20 OS=Mus musculus OX=10090 GN=Cfap20 PE=1 SV=1 | Cfap20 | 0.97 |
| P19253 | 60S ribosomal protein L13a OS=Mus musculus OX=10090 GN=Rpl13a PE=1 SV=4 | Rpl13a | 0.97 |
| Q8CI94 | Glycogen phosphorylase, brain form OS=Mus musculus OX=10090 GN=Pygb PE=1 SV=3 | Pygb | 0.97 |
| Q61166 | Microtubule-associated protein RP/EB family member 1 OS=Mus musculus OX=10090 GN=Mapre1 PE=1 SV=3 | Mapre1 | 0.97 |
| Q8VDP6 | CDP-diacylglycerol--inositol 3-phosphatidyltransferase OS=Mus musculus OX=10090 GN=Cdipt PE=1 SV=1 | Cdipt | 0.97 |
| Q8K2Z4 | Condensin complex subunit 1 OS=Mus musculus OX=10090 GN=Ncapd2 PE=1 SV=2 | Ncapd2 | 0.97 |
| Q80YW0 | Cytohesin-4 OS=Mus musculus OX=10090 GN=Cyth4 PE=1 SV=1 | Cyth4 | 0.97 |
| Q8K411 | Presequence protease, mitochondrial OS=Mus musculus OX=10090 GN=Pitrm1 PE=1 SV=1 | Pitrm1 | 0.97 |
| P60122 | RuvB-like 1 OS=Mus musculus OX=10090 GN=Ruvbl1 PE=1 SV=1 | Ruvbl1 | 0.97 |
| Q8JZQ9 | Eukaryotic translation initiation factor 3 subunit B OS=Mus musculus OX=10090 GN=Eif3b PE=1 SV=1 | Eif3b | 0.97 |
| Q9D4H8 | Cullin-2 OS=Mus musculus OX=10090 GN=Cul2 PE=1 SV=2 | Cul2 | 0.97 |
| P11031 | Activated RNA polymerase II transcriptional coactivator p15 OS=Mus musculus OX=10090 GN=Sub1 PE=1 SV=3 | Sub1 | 0.97 |
| Q9Z1F9 | SUMO-activating enzyme subunit 2 OS=Mus musculus OX=10090 GN=Uba2 PE=1 SV=1 | Uba2 | 0.97 |
| Q66JQ7 | Kinetochore scaffold 1 OS=Mus musculus OX=10090 GN=Knl1 PE=1 SV=3 | Knl1 | 0.97 |
| P47802 | Metaxin-1 OS=Mus musculus OX=10090 GN=Mtx1 PE=1 SV=1 | Mtx1 | 0.97 |
| Q8BTW3 | Exosome complex component MTR3 OS=Mus musculus OX=10090 GN=Exosc6 PE=1 SV=1 | Exosc6 | 0.97 |
| Q8BMS1 | Trifunctional enzyme subunit alpha, mitochondrial OS=Mus musculus OX=10090 GN=Hadha PE=1 SV=1 | Hadha | 0.97 |
| Q8BUB4 | WD repeat and FYVE domain-containing protein 2 OS=Mus musculus OX=10090 GN=Wdfy2 PE=1 SV=2 | Wdfy2 | 0.97 |
| O08663 | Methionine aminopeptidase 2 OS=Mus musculus OX=10090 GN=Metap2 PE=1 SV=1 | Metap2 | 0.97 |
| Q8R1N0 | Zinc finger protein 830 OS=Mus musculus OX=10090 GN=Znf830 PE=1 SV=1 | Znf830 | 0.97 |
| Q60974 | Nuclear receptor corepressor 1 OS=Mus musculus OX=10090 GN=Ncor1 PE=1 SV=1 | Ncor1 | 0.97 |
| P83917 | Chromobox protein homolog 1 OS=Mus musculus OX=10090 GN=Cbx1 PE=1 SV=1 | Cbx1 | 0.97 |
| P84099 | 60S ribosomal protein L19 OS=Mus musculus OX=10090 GN=Rpl19 PE=1 SV=1 | Rpl19 | 0.97 |
| P48025 | Tyrosine-protein kinase SYK OS=Mus musculus OX=10090 GN=Syk PE=1 SV=2 | Syk | 0.97 |
| Q8BK67 | Protein RCC2 OS=Mus musculus OX=10090 GN=Rcc2 PE=1 SV=1 | Rcc2 | 0.97 |
| O08759 | Ubiquitin-protein ligase E3A OS=Mus musculus OX=10090 GN=Ube3a PE=1 SV=2 | Ube3a | 0.97 |
| Q91X58 | AN1-type zinc finger protein 2B OS=Mus musculus OX=10090 GN=Zfand2b PE=1 SV=1 | Zfand2b | 0.97 |
| Q9D8V0 | Minor histocompatibility antigen H13 OS=Mus musculus OX=10090 GN=Hm13 PE=1 SV=1 | Hm13 | 0.97 |
| Q61990 | Poly(rC)-binding protein 2 OS=Mus musculus OX=10090 GN=Pcbp2 PE=1 SV=1 | Pcbp2 | 0.97 |
| Q8BRF7 | Sec1 family domain-containing protein 1 OS=Mus musculus OX=10090 GN=Scfd1 PE=1 SV=1 | Scfd1 | 0.97 |
| Q924T7 | E3 ubiquitin-protein ligase RNF31 OS=Mus musculus OX=10090 GN=Rnf31 PE=1 SV=2 | Rnf31 | 0.97 |
| P45376 | Aldo-keto reductase family 1 member B1 OS=Mus musculus OX=10090 GN=Akr1b1 PE=1 SV=3 | Akr1b1 | 0.97 |
| Q8R1F1 | Protein Niban 2 OS=Mus musculus OX=10090 GN=Niban2 PE=1 SV=2 | Niban2 | 0.97 |
| Q8VDL4 | ADP-dependent glucokinase OS=Mus musculus OX=10090 GN=Adpgk PE=1 SV=2 | Adpgk | 0.97 |
| Q9JI11 | Serine/threonine-protein kinase 4 OS=Mus musculus OX=10090 GN=Stk4 PE=1 SV=1 | Stk4 | 0.97 |
| Q80Y44 | Probable ATP-dependent RNA helicase DDX10 OS=Mus musculus OX=10090 GN=Ddx10 PE=1 SV=2 | Ddx10 | 0.97 |
| P52825 | Carnitine O-palmitoyltransferase 2, mitochondrial OS=Mus musculus OX=10090 GN=Cpt2 PE=1 SV=2 | Cpt2 | 0.97 |
| O88842 | FYVE, RhoGEF and PH domain-containing protein 3 OS=Mus musculus OX=10090 GN=Fgd3 PE=1 SV=1 | Fgd3 | 0.97 |
| P83940 | Elongin-C OS=Mus musculus OX=10090 GN=Eloc PE=1 SV=1 | Eloc | 0.97 |
| Q6Q477 | Plasma membrane calcium-transporting ATPase 4 OS=Mus musculus OX=10090 GN=Atp2b4 PE=1 SV=1 | Atp2b4 | 0.97 |
| Q5PRF0 | HEAT repeat-containing protein 5A OS=Mus musculus OX=10090 GN=Heatr5a PE=1 SV=2 | Heatr5a | 0.97 |
| Q8BL66 | Early endosome antigen 1 OS=Mus musculus OX=10090 GN=Eea1 PE=1 SV=2 | Eea1 | 0.97 |
| Q9WU78 | Programmed cell death 6-interacting protein OS=Mus musculus OX=10090 GN=Pdcd6ip PE=1 SV=3 | Pdcd6ip | 0.97 |
| Q60870 | Receptor expression-enhancing protein 5 OS=Mus musculus OX=10090 GN=Reep5 PE=1 SV=1 | Reep5 | 0.97 |
| Q9Z0H7 | B-cell lymphoma/leukemia 10 OS=Mus musculus OX=10090 GN=Bcl10 PE=1 SV=1 | Bcl10 | 0.97 |
| Q9WVM1 | Rac GTPase-activating protein 1 OS=Mus musculus OX=10090 GN=Racgap1 PE=1 SV=1 | Racgap1 | 0.97 |
| B2RR83 | 3'-5' RNA helicase YTHDC2 OS=Mus musculus OX=10090 GN=Ythdc2 PE=1 SV=1 | Ythdc2 | 0.97 |
| Q9Z2G9 | Oxidoreductase HTATIP2 OS=Mus musculus OX=10090 GN=Htatip2 PE=1 SV=3 | Htatip2 | 0.97 |
| Q8BTT6 | U3 small nucleolar RNA-associated protein 25 homolog OS=Mus musculus OX=10090 GN=Utp25 PE=1 SV=2 | Utp25 | 0.97 |
| Q9D1C9 | Ribosomal RNA-processing protein 7 homolog A OS=Mus musculus OX=10090 GN=Rrp7a PE=2 SV=1 | Rrp7a | 0.97 |
| Q61464 | Zinc finger protein 638 OS=Mus musculus OX=10090 GN=Znf638 PE=1 SV=2 | Znf638 | 0.97 |
| Q9DC33 | High mobility group protein 20A OS=Mus musculus OX=10090 GN=Hmg20a PE=1 SV=1 | Hmg20a | 0.97 |
| E9Q6J5 | Biorientation of chromosomes in cell division protein 1-like 1 OS=Mus musculus OX=10090 GN=Bod1l PE=1 SV=1 | Bod1l | 0.97 |
| Q6PHN9 | Ras-related protein Rab-35 OS=Mus musculus OX=10090 GN=Rab35 PE=1 SV=1 | Rab35 | 0.97 |
| P26039 | Talin-1 OS=Mus musculus OX=10090 GN=Tln1 PE=1 SV=2 | Tln1 | 0.97 |
| P08207 | Protein S100-A10 OS=Mus musculus OX=10090 GN=S100a10 PE=1 SV=2 | S100a10 | 0.98 |
| P53810 | Phosphatidylinositol transfer protein alpha isoform OS=Mus musculus OX=10090 GN=Pitpna PE=1 SV=2 | Pitpna | 0.98 |
| Q8BK08 | Transmembrane protein 11, mitochondrial OS=Mus musculus OX=10090 GN=Tmem11 PE=1 SV=1 | Tmem11 | 0.98 |
| O35955 | Proteasome subunit beta type-10 OS=Mus musculus OX=10090 GN=Psmb10 PE=1 SV=1 | Psmb10 | 0.98 |
| Q3U1J4 | DNA damage-binding protein 1 OS=Mus musculus OX=10090 GN=Ddb1 PE=1 SV=2 | Ddb1 | 0.98 |
| Q99KV1 | DnaJ homolog subfamily B member 11 OS=Mus musculus OX=10090 GN=Dnajb11 PE=1 SV=1 | Dnajb11 | 0.98 |
| Q9DAK9 | 14 kDa phosphohistidine phosphatase OS=Mus musculus OX=10090 GN=Phpt1 PE=1 SV=1 | Phpt1 | 0.98 |
| Q9DC61 | Mitochondrial-processing peptidase subunit alpha OS=Mus musculus OX=10090 GN=Pmpca PE=1 SV=1 | Pmpca | 0.98 |
| Q9CQE7 | Endoplasmic reticulum-Golgi intermediate compartment protein 3 OS=Mus musculus OX=10090 GN=Ergic3 PE=1 SV=1 | Ergic3 | 0.98 |
| Q64475 | Histone H2B type 1-B OS=Mus musculus OX=10090 GN=H2bc3 PE=1 SV=3 | H2bc3 | 0.98 |
| Q8BS90 | Protein aurora borealis OS=Mus musculus OX=10090 GN=Bora PE=1 SV=1 | Bora | 0.98 |
| Q8C8M1 | SIN3-HDAC complex-associated factor OS=Mus musculus OX=10090 GN=Sinhcaf PE=1 SV=1 | Sinhcaf | 0.98 |
| Q8BSY0 | Aspartyl/asparaginyl beta-hydroxylase OS=Mus musculus OX=10090 GN=Asph PE=1 SV=1 | Asph | 0.98 |
| Q9D2Z4 | Sentrin-specific protease 8 OS=Mus musculus OX=10090 GN=Senp8 PE=1 SV=2 | Senp8 | 0.98 |
| Q07076 | Annexin A7 OS=Mus musculus OX=10090 GN=Anxa7 PE=1 SV=2 | Anxa7 | 0.98 |
| Q9Z277 | Tyrosine-protein kinase BAZ1B OS=Mus musculus OX=10090 GN=Baz1b PE=1 SV=2 | Baz1b | 0.98 |
| Q6PDM2 | Serine/arginine-rich splicing factor 1 OS=Mus musculus OX=10090 GN=Srsf1 PE=1 SV=3 | Srsf1 | 0.98 |
| Q9D945 | Protein LLP homolog OS=Mus musculus OX=10090 GN=Llph PE=1 SV=1 | Llph | 0.98 |
| A2A8Z1 | Oxysterol-binding protein-related protein 9 OS=Mus musculus OX=10090 GN=Osbpl9 PE=1 SV=1 | Osbpl9 | 0.98 |
| Q920Q8 | Influenza virus NS1A-binding protein homolog OS=Mus musculus OX=10090 GN=Ivns1abp PE=1 SV=2 | Ivns1abp | 0.98 |
| Q3U1G5 | Interferon-stimulated 20 kDa exonuclease-like 2 OS=Mus musculus OX=10090 GN=Isg20l2 PE=1 SV=2 | Isg20l2 | 0.98 |
| Q8K2Q9 | Shootin-1 OS=Mus musculus OX=10090 GN=Shtn1 PE=1 SV=1 | Shtn1 | 0.98 |
| Q99J27 | Acetyl-coenzyme A transporter 1 OS=Mus musculus OX=10090 GN=Slc33a1 PE=1 SV=1 | Slc33a1 | 0.98 |
| P24638 | Lysosomal acid phosphatase OS=Mus musculus OX=10090 GN=Acp2 PE=1 SV=2 | Acp2 | 0.98 |
| P70279 | Surfeit locus protein 6 OS=Mus musculus OX=10090 GN=Surf6 PE=1 SV=1 | Surf6 | 0.98 |
| Q2VPQ9 | Chromatin modification-related protein MEAF6 OS=Mus musculus OX=10090 GN=Meaf6 PE=1 SV=1 | Meaf6 | 0.98 |
| Q9DCH6 | AN1-type zinc finger protein 6 OS=Mus musculus OX=10090 GN=Zfand6 PE=1 SV=1 | Zfand6 | 0.98 |
| Q9CPV5 | Polyamine-modulated factor 1 OS=Mus musculus OX=10090 GN=Pmf1 PE=1 SV=1 | Pmf1 | 0.98 |
| P83887 | Tubulin gamma-1 chain OS=Mus musculus OX=10090 GN=Tubg1 PE=1 SV=1 | Tubg1 | 0.98 |
| P11438 | Lysosome-associated membrane glycoprotein 1 OS=Mus musculus OX=10090 GN=Lamp1 PE=1 SV=2 | Lamp1 | 0.98 |
| Q8K3A9 | 7SK snRNA methylphosphate capping enzyme OS=Mus musculus OX=10090 GN=Mepce PE=1 SV=2 | Mepce | 0.98 |
| P00920 | Carbonic anhydrase 2 OS=Mus musculus OX=10090 GN=Ca2 PE=1 SV=4 | Ca2 | 0.98 |
| Q3UFS0 | Protein zyg-11 homolog B OS=Mus musculus OX=10090 GN=Zyg11b PE=1 SV=2 | Zyg11b | 0.98 |
| Q8C181 | Muscleblind-like protein 2 OS=Mus musculus OX=10090 GN=Mbnl2 PE=2 SV=2 | Mbnl2 | 0.98 |
| Q8VCL2 | Protein SCO2 homolog, mitochondrial OS=Mus musculus OX=10090 GN=Sco2 PE=1 SV=1 | Sco2 | 0.98 |
| Q80XU3 | Nuclear ubiquitous casein and cyclin-dependent kinase substrate 1 OS=Mus musculus OX=10090 GN=Nucks1 PE=1 SV=1 | Nucks1 | 0.98 |
| O35134 | DNA-directed RNA polymerase I subunit RPA1 OS=Mus musculus OX=10090 GN=Polr1a PE=1 SV=2 | Polr1a | 0.98 |
| Q5SXY1 | Cytospin-B OS=Mus musculus OX=10090 GN=Specc1 PE=1 SV=2 | Specc1 | 0.98 |
| Q8R0F6 | Integrin-linked kinase-associated serine/threonine phosphatase 2C OS=Mus musculus OX=10090 GN=Ilkap PE=1 SV=1 | Ilkap | 0.98 |
| Q9ES28 | Rho guanine nucleotide exchange factor 7 OS=Mus musculus OX=10090 GN=Arhgef7 PE=1 SV=2 | Arhgef7 | 0.98 |
| Q8BZA9 | Fructose-2,6-bisphosphatase TIGAR OS=Mus musculus OX=10090 GN=Tigar PE=1 SV=1 | Tigar | 0.98 |
| Q921S7 | 39S ribosomal protein L37, mitochondrial OS=Mus musculus OX=10090 GN=Mrpl37 PE=1 SV=1 | Mrpl37 | 0.98 |
| Q9JHE7 | Protein TSSC4 OS=Mus musculus OX=10090 GN=Tssc4 PE=1 SV=1 | Tssc4 | 0.98 |
| Q9EPQ8 | Transcription factor 20 OS=Mus musculus OX=10090 GN=Tcf20 PE=1 SV=3 | Tcf20 | 0.98 |
| P97471 | Mothers against decapentaplegic homolog 4 OS=Mus musculus OX=10090 GN=Smad4 PE=1 SV=2 | Smad4 | 0.98 |
| Q91YN5 | UDP-N-acetylhexosamine pyrophosphorylase OS=Mus musculus OX=10090 GN=Uap1 PE=1 SV=1 | Uap1 | 0.98 |
| Q9Z0R6 | Intersectin-2 OS=Mus musculus OX=10090 GN=Itsn2 PE=1 SV=2 | Itsn2 | 0.98 |
| P08775 | DNA-directed RNA polymerase II subunit RPB1 OS=Mus musculus OX=10090 GN=Polr2a PE=1 SV=3 | Polr2a | 0.98 |
| Q61160 | FAS-associated death domain protein OS=Mus musculus OX=10090 GN=Fadd PE=1 SV=1 | Fadd | 0.98 |
| Q3UHD6 | Sorting nexin-27 OS=Mus musculus OX=10090 GN=Snx27 PE=1 SV=2 | Snx27 | 0.98 |
| P47968 | Ribose-5-phosphate isomerase OS=Mus musculus OX=10090 GN=Rpia PE=1 SV=2 | Rpia | 0.98 |
| Q3TIU4 | 2',5'-phosphodiesterase 12 OS=Mus musculus OX=10090 GN=Pde12 PE=1 SV=2 | Pde12 | 0.98 |
| Q9DB90 | Protein SMG9 OS=Mus musculus OX=10090 GN=Smg9 PE=1 SV=1 | Smg9 | 0.98 |
| Q8CGY8 | UDP-N-acetylglucosamine--peptide N-acetylglucosaminyltransferase 110 kDa subunit OS=Mus musculus OX=10090 GN=Ogt PE=1 SV=2 | Ogt | 0.98 |
| P81269 | Cyclic AMP-dependent transcription factor ATF-1 OS=Mus musculus OX=10090 GN=Atf1 PE=1 SV=1 | Atf1 | 0.98 |
| Q6P9Q4 | FH1/FH2 domain-containing protein 1 OS=Mus musculus OX=10090 GN=Fhod1 PE=1 SV=3 | Fhod1 | 0.98 |
| Q3UVL4 | Vacuolar protein sorting-associated protein 51 homolog OS=Mus musculus OX=10090 GN=Vps51 PE=1 SV=2 | Vps51 | 0.98 |
| Q3UCV8 | Ubiquitin thioesterase otulin OS=Mus musculus OX=10090 GN=Otulin PE=1 SV=1 | Otulin | 0.98 |
| Q9ERB0 | Synaptosomal-associated protein 29 OS=Mus musculus OX=10090 GN=Snap29 PE=1 SV=1 | Snap29 | 0.98 |
| P47856 | Glutamine--fructose-6-phosphate aminotransferase [isomerizing] 1 OS=Mus musculus OX=10090 GN=Gfpt1 PE=1 SV=3 | Gfpt1 | 0.98 |
| P45377 | Aldose reductase-related protein 2 OS=Mus musculus OX=10090 GN=Akr1b8 PE=1 SV=2 | Akr1b8 | 0.98 |
| Q63810 | Calcineurin subunit B type 1 OS=Mus musculus OX=10090 GN=Ppp3r1 PE=1 SV=3 | Ppp3r1 | 0.98 |
| P37913 | DNA ligase 1 OS=Mus musculus OX=10090 GN=Lig1 PE=1 SV=2 | Lig1 | 0.98 |
| Q9D0W5 | Peptidyl-prolyl cis-trans isomerase-like 1 OS=Mus musculus OX=10090 GN=Ppil1 PE=1 SV=1 | Ppil1 | 0.98 |
| Q91ZV0 | Melanoma inhibitory activity protein 2 OS=Mus musculus OX=10090 GN=Mia2 PE=1 SV=3 | Mia2 | 0.98 |
| P47713 | Cytosolic phospholipase A2 OS=Mus musculus OX=10090 GN=Pla2g4a PE=1 SV=1 | Pla2g4a | 0.98 |
| O70251 | Elongation factor 1-beta OS=Mus musculus OX=10090 GN=Eef1b PE=1 SV=5 | Eef1b | 0.98 |
| Q80UZ2 | Protein SDA1 homolog OS=Mus musculus OX=10090 GN=Sdad1 PE=1 SV=1 | Sdad1 | 0.98 |
| Q8C3X2 | Coiled-coil domain-containing protein 90B, mitochondrial OS=Mus musculus OX=10090 GN=Ccdc90b PE=1 SV=1 | Ccdc90b | 0.98 |
| Q00262 | Syntaxin-2 OS=Mus musculus OX=10090 GN=Stx2 PE=1 SV=1 | Stx2 | 0.98 |
| O35286 | Pre-mRNA-splicing factor ATP-dependent RNA helicase DHX15 OS=Mus musculus OX=10090 GN=Dhx15 PE=1 SV=2 | Dhx15 | 0.98 |
| Q8BKS9 | Pumilio homolog 3 OS=Mus musculus OX=10090 GN=Pum3 PE=1 SV=2 | Pum3 | 0.98 |
| P51863 | V-type proton ATPase subunit d 1 OS=Mus musculus OX=10090 GN=Atp6v0d1 PE=1 SV=2 | Atp6v0d1 | 0.98 |
| Q8K368 | Fanconi anemia group I protein homolog OS=Mus musculus OX=10090 GN=Fanci PE=1 SV=2 | Fanci | 0.98 |
| Q6ZQ08 | CCR4-NOT transcription complex subunit 1 OS=Mus musculus OX=10090 GN=Cnot1 PE=1 SV=2 | Cnot1 | 0.98 |
| Q922H2 | [Pyruvate dehydrogenase (acetyl-transferring)] kinase isozyme 3, mitochondrial OS=Mus musculus OX=10090 GN=Pdk3 PE=1 SV=1 | Pdk3 | 0.98 |
| Q9QZ08 | N-acetyl-D-glucosamine kinase OS=Mus musculus OX=10090 GN=Nagk PE=1 SV=3 | Nagk | 0.98 |
| P60710 | Actin, cytoplasmic 1 OS=Mus musculus OX=10090 GN=Actb PE=1 SV=1 | Actb | 0.98 |
| P60840 | Alpha-endosulfine OS=Mus musculus OX=10090 GN=Ensa PE=1 SV=1 | Ensa | 0.98 |
| P48758 | Carbonyl reductase [NADPH] 1 OS=Mus musculus OX=10090 GN=Cbr1 PE=1 SV=3 | Cbr1 | 0.98 |
| Q6Y685 | Transforming acidic coiled-coil-containing protein 1 OS=Mus musculus OX=10090 GN=Tacc1 PE=1 SV=1 | Tacc1 | 0.98 |
| Q8VDM4 | 26S proteasome non-ATPase regulatory subunit 2 OS=Mus musculus OX=10090 GN=Psmd2 PE=1 SV=1 | Psmd2 | 0.98 |
| Q9CY64 | Biliverdin reductase A OS=Mus musculus OX=10090 GN=Blvra PE=1 SV=1 | Blvra | 0.98 |
| Q99KD5 | Protein unc-45 homolog A OS=Mus musculus OX=10090 GN=Unc45a PE=1 SV=2 | Unc45a | 0.98 |
| P17710 | Hexokinase-1 OS=Mus musculus OX=10090 GN=Hk1 PE=1 SV=3 | Hk1 | 0.98 |
| Q91VS8 | FERM, ARHGEF and pleckstrin domain-containing protein 2 OS=Mus musculus OX=10090 GN=Farp2 PE=1 SV=2 | Farp2 | 0.98 |
| Q6ZQI3 | Malectin OS=Mus musculus OX=10090 GN=Mlec PE=1 SV=2 | Mlec | 0.98 |
| Q9D8Z2 | TP53-regulated inhibitor of apoptosis 1 OS=Mus musculus OX=10090 GN=Triap1 PE=1 SV=1 | Triap1 | 0.98 |
| Q9CRA5 | Golgi phosphoprotein 3 OS=Mus musculus OX=10090 GN=Golph3 PE=1 SV=1 | Golph3 | 0.98 |
| Q91V04 | Translocating chain-associated membrane protein 1 OS=Mus musculus OX=10090 GN=Tram1 PE=1 SV=3 | Tram1 | 0.98 |
| P53702 | Holocytochrome c-type synthase OS=Mus musculus OX=10090 GN=Hccs PE=1 SV=2 | Hccs | 0.98 |
| Q9CQJ8 | NADH dehydrogenase [ubiquinone] 1 beta subcomplex subunit 9 OS=Mus musculus OX=10090 GN=Ndufb9 PE=1 SV=3 | Ndufb9 | 0.98 |
| Q9ER80 | Receptor-transporting protein 4 OS=Mus musculus OX=10090 GN=Rtp4 PE=1 SV=1 | Rtp4 | 0.98 |
| P53564 | Homeobox protein cut-like 1 OS=Mus musculus OX=10090 GN=Cux1 PE=1 SV=3 | Cux1 | 0.98 |
| Q8K2Q0 | COMM domain-containing protein 9 OS=Mus musculus OX=10090 GN=Commd9 PE=1 SV=3 | Commd9 | 0.98 |
| Q3UPL0 | Protein transport protein Sec31A OS=Mus musculus OX=10090 GN=Sec31a PE=1 SV=2 | Sec31a | 0.98 |
| Q99LD4 | COP9 signalosome complex subunit 1 OS=Mus musculus OX=10090 GN=Gps1 PE=1 SV=1 | Gps1 | 0.98 |
| Q9D0M2 | Cell division cycle-associated protein 7 OS=Mus musculus OX=10090 GN=Cdca7 PE=1 SV=1 | Cdca7 | 0.98 |
| Q3UKJ7 | WD40 repeat-containing protein SMU1 OS=Mus musculus OX=10090 GN=Smu1 PE=2 SV=2 | Smu1 | 0.98 |
| Q3TGW2 | Endonuclease/exonuclease/phosphatase family domain-containing protein 1 OS=Mus musculus OX=10090 GN=Eepd1 PE=1 SV=1 | Eepd1 | 0.98 |
| Q921F2 | TAR DNA-binding protein 43 OS=Mus musculus OX=10090 GN=Tardbp PE=1 SV=1 | Tardbp | 0.98 |
| Q0P678 | Zinc finger CCCH domain-containing protein 18 OS=Mus musculus OX=10090 GN=Zc3h18 PE=1 SV=1 | Zc3h18 | 0.98 |
| Q8VE99 | Coiled-coil domain-containing protein 115 OS=Mus musculus OX=10090 GN=Ccdc115 PE=1 SV=1 | Ccdc115 | 0.98 |
| P25444 | 40S ribosomal protein S2 OS=Mus musculus OX=10090 GN=Rps2 PE=1 SV=3 | Rps2 | 0.98 |
| Q8R149 | BUD13 homolog OS=Mus musculus OX=10090 GN=Bud13 PE=1 SV=1 | Bud13 | 0.98 |
| Q91YU8 | Suppressor of SWI4 1 homolog OS=Mus musculus OX=10090 GN=Ppan PE=1 SV=2 | Ppan | 0.98 |
| Q61655 | ATP-dependent RNA helicase DDX19A OS=Mus musculus OX=10090 GN=Ddx19a PE=1 SV=2 | Ddx19a | 0.98 |
| Q4QY64 | ATPase family AAA domain-containing protein 5 OS=Mus musculus OX=10090 GN=Atad5 PE=1 SV=1 | Atad5 | 0.98 |
| P70700 | DNA-directed RNA polymerase I subunit RPA2 OS=Mus musculus OX=10090 GN=Polr1b PE=1 SV=2 | Polr1b | 0.98 |
| P56394 | Cytochrome c oxidase copper chaperone OS=Mus musculus OX=10090 GN=Cox17 PE=1 SV=2 | Cox17 | 0.98 |
| P30681 | High mobility group protein B2 OS=Mus musculus OX=10090 GN=Hmgb2 PE=1 SV=3 | Hmgb2 | 0.98 |
| P62334 | 26S proteasome regulatory subunit 10B OS=Mus musculus OX=10090 GN=Psmc6 PE=1 SV=1 | Psmc6 | 0.98 |
| Q99LL5 | Periodic tryptophan protein 1 homolog OS=Mus musculus OX=10090 GN=Pwp1 PE=1 SV=1 | Pwp1 | 0.98 |
| Q6NZJ6 | Eukaryotic translation initiation factor 4 gamma 1 OS=Mus musculus OX=10090 GN=Eif4g1 PE=1 SV=1 | Eif4g1 | 0.98 |
| P62141 | Serine/threonine-protein phosphatase PP1-beta catalytic subunit OS=Mus musculus OX=10090 GN=Ppp1cb PE=1 SV=3 | Ppp1cb | 0.98 |
| Q810D6 | Glutamate-rich WD repeat-containing protein 1 OS=Mus musculus OX=10090 GN=Grwd1 PE=1 SV=2 | Grwd1 | 0.98 |
| P39689 | Cyclin-dependent kinase inhibitor 1 OS=Mus musculus OX=10090 GN=Cdkn1a PE=1 SV=4 | Cdkn1a | 0.98 |
| Q9DCE5 | p21-activated protein kinase-interacting protein 1 OS=Mus musculus OX=10090 GN=Pak1ip1 PE=1 SV=2 | Pak1ip1 | 0.98 |
| Q9R0Q7 | Prostaglandin E synthase 3 OS=Mus musculus OX=10090 GN=Ptges3 PE=1 SV=1 | Ptges3 | 0.98 |
| Q9DBC7 | cAMP-dependent protein kinase type I-alpha regulatory subunit OS=Mus musculus OX=10090 GN=Prkar1a PE=1 SV=3 | Prkar1a | 0.98 |
| Q9WU81 | Glucose-6-phosphate exchanger SLC37A2 OS=Mus musculus OX=10090 GN=Slc37a2 PE=1 SV=1 | Slc37a2 | 0.98 |
| Q9JKW0 | ADP-ribosylation factor-like protein 6-interacting protein 1 OS=Mus musculus OX=10090 GN=Arl6ip1 PE=1 SV=1 | Arl6ip1 | 0.98 |
| Q9JJA7 | Cyclin-L2 OS=Mus musculus OX=10090 GN=Ccnl2 PE=1 SV=1 | Ccnl2 | 0.98 |
| Q91WF7 | Polyphosphoinositide phosphatase OS=Mus musculus OX=10090 GN=Fig4 PE=1 SV=1 | Fig4 | 0.98 |
| P13597 | Intercellular adhesion molecule 1 OS=Mus musculus OX=10090 GN=Icam1 PE=1 SV=1 | Icam1 | 0.98 |
| P63276 | 40S ribosomal protein S17 OS=Mus musculus OX=10090 GN=Rps17 PE=1 SV=2 | Rps17 | 0.98 |
| P24369 | Peptidyl-prolyl cis-trans isomerase B OS=Mus musculus OX=10090 GN=Ppib PE=1 SV=2 | Ppib | 0.98 |
| P11627 | Neural cell adhesion molecule L1 OS=Mus musculus OX=10090 GN=L1cam PE=1 SV=1 | L1cam | 0.98 |
| Q9DBS1 | Transmembrane protein 43 OS=Mus musculus OX=10090 GN=Tmem43 PE=1 SV=1 | Tmem43 | 0.98 |
| Q3UFY8 | tRNA methyltransferase 10 homolog C OS=Mus musculus OX=10090 GN=Trmt10c PE=1 SV=2 | Trmt10c | 0.98 |
| Q3TEA8 | Heterochromatin protein 1-binding protein 3 OS=Mus musculus OX=10090 GN=Hp1bp3 PE=1 SV=1 | Hp1bp3 | 0.98 |
| Q99MR8 | Methylcrotonoyl-CoA carboxylase subunit alpha, mitochondrial OS=Mus musculus OX=10090 GN=Mccc1 PE=1 SV=2 | Mccc1 | 0.98 |
| P46460 | Vesicle-fusing ATPase OS=Mus musculus OX=10090 GN=Nsf PE=1 SV=2 | Nsf | 0.98 |
| P14869 | 60S acidic ribosomal protein P0 OS=Mus musculus OX=10090 GN=Rplp0 PE=1 SV=3 | Rplp0 | 0.98 |
| Q8BVW3 | Tripartite motif-containing protein 14 OS=Mus musculus OX=10090 GN=Trim14 PE=1 SV=2 | Trim14 | 0.98 |
| P97372 | Proteasome activator complex subunit 2 OS=Mus musculus OX=10090 GN=Psme2 PE=1 SV=4 | Psme2 | 0.98 |
| P54276 | DNA mismatch repair protein Msh6 OS=Mus musculus OX=10090 GN=Msh6 PE=1 SV=3 | Msh6 | 0.98 |
| Q58NB6 | Dehydrogenase/reductase SDR family member 9 OS=Mus musculus OX=10090 GN=Dhrs9 PE=2 SV=2 | Dhrs9 | 0.99 |
| P55264 | Adenosine kinase OS=Mus musculus OX=10090 GN=Adk PE=1 SV=2 | Adk | 0.99 |
| O70318 | Band 4.1-like protein 2 OS=Mus musculus OX=10090 GN=Epb41l2 PE=1 SV=2 | Epb41l2 | 0.99 |
| Q8CH02 | SURP and G-patch domain-containing protein 1 OS=Mus musculus OX=10090 GN=Sugp1 PE=1 SV=1 | Sugp1 | 0.99 |
| O35495 | Cyclin-dependent kinase 14 OS=Mus musculus OX=10090 GN=Cdk14 PE=1 SV=2 | Cdk14 | 0.99 |
| P62897 | Cytochrome c, somatic OS=Mus musculus OX=10090 GN=Cycs PE=1 SV=2 | Cycs | 0.99 |
| A2BDX3 | Adenylyltransferase and sulfurtransferase MOCS3 OS=Mus musculus OX=10090 GN=Mocs3 PE=1 SV=1 | Mocs3 | 0.99 |
| P05064 | Fructose-bisphosphate aldolase A OS=Mus musculus OX=10090 GN=Aldoa PE=1 SV=2 | Aldoa | 0.99 |
| O35864 | COP9 signalosome complex subunit 5 OS=Mus musculus OX=10090 GN=Cops5 PE=1 SV=3 | Cops5 | 0.99 |
| Q9R0Q9 | Mannose-P-dolichol utilization defect 1 protein OS=Mus musculus OX=10090 GN=Mpdu1 PE=1 SV=1 | Mpdu1 | 0.99 |
| E9Q236 | ATP-binding cassette sub-family C member 4 OS=Mus musculus OX=10090 GN=Abcc4 PE=1 SV=1 | Abcc4 | 0.99 |
| Q8K5B2 | Multiple coagulation factor deficiency protein 2 homolog OS=Mus musculus OX=10090 GN=Mcfd2 PE=1 SV=1 | Mcfd2 | 0.99 |
| Q9CYR6 | Phosphoacetylglucosamine mutase OS=Mus musculus OX=10090 GN=Pgm3 PE=1 SV=1 | Pgm3 | 0.99 |
| Q9CYA6 | Zinc finger CCHC domain-containing protein 8 OS=Mus musculus OX=10090 GN=Zcchc8 PE=1 SV=3 | Zcchc8 | 0.99 |
| Q9EP89 | Serine beta-lactamase-like protein LACTB, mitochondrial OS=Mus musculus OX=10090 GN=Lactb PE=1 SV=1 | Lactb | 0.99 |
| Q7M6Y3 | Phosphatidylinositol-binding clathrin assembly protein OS=Mus musculus OX=10090 GN=Picalm PE=1 SV=1 | Picalm | 0.99 |
| Q9CXX9 | CUE domain-containing protein 2 OS=Mus musculus OX=10090 GN=Cuedc2 PE=1 SV=1 | Cuedc2 | 0.99 |
| Q6PD19 | Armadillo-like helical domain-containing protein 3 OS=Mus musculus OX=10090 GN=Armh3 PE=1 SV=2 | Armh3 | 0.99 |
| Q9CZT6 | Protein CMSS1 OS=Mus musculus OX=10090 GN=Cmss1 PE=2 SV=1 | Cmss1 | 0.99 |
| Q00899 | Transcriptional repressor protein YY1 OS=Mus musculus OX=10090 GN=Yy1 PE=1 SV=1 | Yy1 | 0.99 |
| Q9WUB4 | Dynactin subunit 6 OS=Mus musculus OX=10090 GN=Dctn6 PE=1 SV=1 | Dctn6 | 0.99 |
| Q99LG4 | Tetratricopeptide repeat protein 5 OS=Mus musculus OX=10090 GN=Ttc5 PE=1 SV=2 | Ttc5 | 0.99 |
| Q8K114 | Integrator complex subunit 9 OS=Mus musculus OX=10090 GN=Ints9 PE=1 SV=1 | Ints9 | 0.99 |
| P24270 | Catalase OS=Mus musculus OX=10090 GN=Cat PE=1 SV=4 | Cat | 0.99 |
| Q99LF4 | RNA-splicing ligase RtcB homolog OS=Mus musculus OX=10090 GN=Rtcb PE=1 SV=1 | Rtcb | 0.99 |
| Q8CG48 | Structural maintenance of chromosomes protein 2 OS=Mus musculus OX=10090 GN=Smc2 PE=1 SV=2 | Smc2 | 0.99 |
| Q9D771 | Proton-activated chloride channel OS=Mus musculus OX=10090 GN=Pacc1 PE=1 SV=1 | Pacc1 | 0.99 |
| Q923D4 | Splicing factor 3B subunit 5 OS=Mus musculus OX=10090 GN=Sf3b5 PE=1 SV=1 | Sf3b5 | 0.99 |
| Q8CGC7 | Bifunctional glutamate/proline--tRNA ligase OS=Mus musculus OX=10090 GN=Eprs1 PE=1 SV=4 | Eprs1 | 0.99 |
| P52503 | NADH dehydrogenase [ubiquinone] iron-sulfur protein 6, mitochondrial OS=Mus musculus OX=10090 GN=Ndufs6 PE=1 SV=2 | Ndufs6 | 0.99 |
| Q8VI75 | Importin-4 OS=Mus musculus OX=10090 GN=Ipo4 PE=1 SV=1 | Ipo4 | 0.99 |
| Q9DCU6 | 39S ribosomal protein L4, mitochondrial OS=Mus musculus OX=10090 GN=Mrpl4 PE=1 SV=1 | Mrpl4 | 0.99 |
| Q9D3P8 | Plasminogen receptor (KT) OS=Mus musculus OX=10090 GN=Plgrkt PE=1 SV=1 | Plgrkt | 0.99 |
| Q3UMQ8 | H/ACA ribonucleoprotein complex non-core subunit NAF1 OS=Mus musculus OX=10090 GN=Naf1 PE=1 SV=2 | Naf1 | 0.99 |
| Q99LD9 | Translation initiation factor eIF-2B subunit beta OS=Mus musculus OX=10090 GN=Eif2b2 PE=1 SV=1 | Eif2b2 | 0.99 |
| P58242 | Acid sphingomyelinase-like phosphodiesterase 3b OS=Mus musculus OX=10090 GN=Smpdl3b PE=1 SV=1 | Smpdl3b | 0.99 |
| Q9QXA5 | U6 snRNA-associated Sm-like protein LSm4 OS=Mus musculus OX=10090 GN=Lsm4 PE=1 SV=1 | Lsm4 | 0.99 |
| Q8BHJ9 | Pre-mRNA-splicing factor SLU7 OS=Mus musculus OX=10090 GN=Slu7 PE=1 SV=1 | Slu7 | 0.99 |
| Q09200 | Beta-1,4 N-acetylgalactosaminyltransferase 1 OS=Mus musculus OX=10090 GN=B4galnt1 PE=1 SV=1 | B4galnt1 | 0.99 |
| O55029 | Coatomer subunit beta' OS=Mus musculus OX=10090 GN=Copb2 PE=1 SV=2 | Copb2 | 0.99 |
| Q64339 | Ubiquitin-like protein ISG15 OS=Mus musculus OX=10090 GN=Isg15 PE=1 SV=4 | Isg15 | 0.99 |
| Q9QXX4 | Calcium-binding mitochondrial carrier protein Aralar2 OS=Mus musculus OX=10090 GN=Slc25a13 PE=1 SV=1 | Slc25a13 | 0.99 |
| Q3THG9 | Alanyl-tRNA editing protein Aarsd1 OS=Mus musculus OX=10090 GN=Aarsd1 PE=1 SV=2 | Aarsd1 | 0.99 |
| Q8VEE4 | Replication protein A 70 kDa DNA-binding subunit OS=Mus musculus OX=10090 GN=Rpa1 PE=1 SV=1 | Rpa1 | 0.99 |
| O08599 | Syntaxin-binding protein 1 OS=Mus musculus OX=10090 GN=Stxbp1 PE=1 SV=2 | Stxbp1 | 0.99 |
| Q3THJ3 | Probable RNA-binding protein EIF1AD OS=Mus musculus OX=10090 GN=Eif1ad PE=1 SV=2 | Eif1ad | 0.99 |
| Q61127 | NGFI-A-binding protein 2 OS=Mus musculus OX=10090 GN=Nab2 PE=1 SV=2 | Nab2 | 0.99 |
| Q8BXK4 | S-adenosylmethionine sensor upstream of mTORC1 OS=Mus musculus OX=10090 GN=Bmt2 PE=2 SV=2 | Bmt2 | 0.99 |
| Q9JIH2 | Nuclear pore complex protein Nup50 OS=Mus musculus OX=10090 GN=Nup50 PE=1 SV=3 | Nup50 | 0.99 |
| Q8R010 | Aminoacyl tRNA synthase complex-interacting multifunctional protein 2 OS=Mus musculus OX=10090 GN=Aimp2 PE=1 SV=2 | Aimp2 | 0.99 |
| Q9Z1D1 | Eukaryotic translation initiation factor 3 subunit G OS=Mus musculus OX=10090 GN=Eif3g PE=1 SV=2 | Eif3g | 0.99 |
| P23116 | Eukaryotic translation initiation factor 3 subunit A OS=Mus musculus OX=10090 GN=Eif3a PE=1 SV=5 | Eif3a | 0.99 |
| Q9JLV6 | Bifunctional polynucleotide phosphatase/kinase OS=Mus musculus OX=10090 GN=Pnkp PE=1 SV=2 | Pnkp | 0.99 |
| Q8VEJ4 | Notchless protein homolog 1 OS=Mus musculus OX=10090 GN=Nle1 PE=1 SV=4 | Nle1 | 0.99 |
| Q61937 | Nucleophosmin OS=Mus musculus OX=10090 GN=Npm1 PE=1 SV=1 | Npm1 | 0.99 |
| O88351 | Inhibitor of nuclear factor kappa-B kinase subunit beta OS=Mus musculus OX=10090 GN=Ikbkb PE=1 SV=1 | Ikbkb | 0.99 |
| Q99J62 | Replication factor C subunit 4 OS=Mus musculus OX=10090 GN=Rfc4 PE=1 SV=1 | Rfc4 | 0.99 |
| Q9CX11 | rRNA-processing protein UTP23 homolog OS=Mus musculus OX=10090 GN=Utp23 PE=2 SV=1 | Utp23 | 0.99 |
| P27659 | 60S ribosomal protein L3 OS=Mus musculus OX=10090 GN=Rpl3 PE=1 SV=3 | Rpl3 | 0.99 |
| Q9DC28 | Casein kinase I isoform delta OS=Mus musculus OX=10090 GN=Csnk1d PE=1 SV=2 | Csnk1d | 0.99 |
| Q9CQT1 | Methylthioribose-1-phosphate isomerase OS=Mus musculus OX=10090 GN=Mri1 PE=1 SV=1 | Mri1 | 0.99 |
| P70670 | Nascent polypeptide-associated complex subunit alpha, muscle-specific form OS=Mus musculus OX=10090 GN=Naca PE=1 SV=2 | Naca | 0.99 |
| P27773 | Protein disulfide-isomerase A3 OS=Mus musculus OX=10090 GN=Pdia3 PE=1 SV=2 | Pdia3 | 0.99 |
| Q60953 | Protein PML OS=Mus musculus OX=10090 GN=Pml PE=1 SV=3 | Pml | 0.99 |
| Q9CZ57 | 5-methylcytosine rRNA methyltransferase NSUN4 OS=Mus musculus OX=10090 GN=Nsun4 PE=1 SV=1 | Nsun4 | 0.99 |
| Q99KX1 | Myeloid leukemia factor 2 OS=Mus musculus OX=10090 GN=Mlf2 PE=1 SV=1 | Mlf2 | 0.99 |
| Q8R1X6 | Spartin OS=Mus musculus OX=10090 GN=Spart PE=1 SV=1 | Spart | 0.99 |
| Q91VC9 | Growth hormone-inducible transmembrane protein OS=Mus musculus OX=10090 GN=Ghitm PE=1 SV=1 | Ghitm | 0.99 |
| Q91YL2 | E3 ubiquitin-protein ligase RNF126 OS=Mus musculus OX=10090 GN=Rnf126 PE=1 SV=1 | Rnf126 | 0.99 |
| Q8R2N2 | U3 small nucleolar RNA-associated protein 4 homolog OS=Mus musculus OX=10090 GN=Utp4 PE=2 SV=3 | Utp4 | 0.99 |
| Q62384 | Zinc finger protein ZPR1 OS=Mus musculus OX=10090 GN=Zpr1 PE=1 SV=1 | Zpr1 | 0.99 |
| Q99JI4 | 26S proteasome non-ATPase regulatory subunit 6 OS=Mus musculus OX=10090 GN=Psmd6 PE=1 SV=1 | Psmd6 | 0.99 |
| Q62348 | Translin OS=Mus musculus OX=10090 GN=Tsn PE=1 SV=1 | Tsn | 0.99 |
| P23906 | Interferon regulatory factor 2 OS=Mus musculus OX=10090 GN=Irf2 PE=1 SV=1 | Irf2 | 0.99 |
| O35609 | Secretory carrier-associated membrane protein 3 OS=Mus musculus OX=10090 GN=Scamp3 PE=1 SV=3 | Scamp3 | 0.99 |
| P24452 | Macrophage-capping protein OS=Mus musculus OX=10090 GN=Capg PE=1 SV=2 | Capg | 0.99 |
| Q9CQ40 | 39S ribosomal protein L49, mitochondrial OS=Mus musculus OX=10090 GN=Mrpl49 PE=1 SV=1 | Mrpl49 | 0.99 |
| Q64133 | Amine oxidase [flavin-containing] A OS=Mus musculus OX=10090 GN=Maoa PE=1 SV=3 | Maoa | 0.99 |
| Q8VHI3 | GDP-fucose protein O-fucosyltransferase 2 OS=Mus musculus OX=10090 GN=Pofut2 PE=1 SV=1 | Pofut2 | 0.99 |
| Q9QXD8 | LIM domain-containing protein 1 OS=Mus musculus OX=10090 GN=Limd1 PE=1 SV=2 | Limd1 | 0.99 |
| Q9D0C4 | tRNA (guanine(37)-N1)-methyltransferase OS=Mus musculus OX=10090 GN=Trmt5 PE=1 SV=1 | Trmt5 | 0.99 |
| P51949 | CDK-activating kinase assembly factor MAT1 OS=Mus musculus OX=10090 GN=Mnat1 PE=1 SV=2 | Mnat1 | 0.99 |
| P15532 | Nucleoside diphosphate kinase A OS=Mus musculus OX=10090 GN=Nme1 PE=1 SV=1 | Nme1 | 0.99 |
| Q9DBE0 | Cysteine sulfinic acid decarboxylase OS=Mus musculus OX=10090 GN=Csad PE=1 SV=1 | Csad | 0.99 |
| P49710 | Hematopoietic lineage cell-specific protein OS=Mus musculus OX=10090 GN=Hcls1 PE=1 SV=2 | Hcls1 | 0.99 |
| P26151 | High affinity immunoglobulin gamma Fc receptor I OS=Mus musculus OX=10090 GN=Fcgr1 PE=1 SV=1 | Fcgr1 | 0.99 |
| Q91ZJ5 | UTP--glucose-1-phosphate uridylyltransferase OS=Mus musculus OX=10090 GN=Ugp2 PE=1 SV=3 | Ugp2 | 0.99 |
| O09044 | Synaptosomal-associated protein 23 OS=Mus musculus OX=10090 GN=Snap23 PE=1 SV=1 | Snap23 | 0.99 |
| Q8VCH8 | UBX domain-containing protein 4 OS=Mus musculus OX=10090 GN=Ubxn4 PE=1 SV=1 | Ubxn4 | 0.99 |
| Q8R0X7 | Sphingosine-1-phosphate lyase 1 OS=Mus musculus OX=10090 GN=Sgpl1 PE=1 SV=1 | Sgpl1 | 0.99 |
| Q8VC30 | Triokinase/FMN cyclase OS=Mus musculus OX=10090 GN=Tkfc PE=1 SV=1 | Tkfc | 0.99 |
| Q80WQ2 | Protein VAC14 homolog OS=Mus musculus OX=10090 GN=Vac14 PE=1 SV=1 | Vac14 | 0.99 |
| Q9D4V0 | Ethanolamine kinase 1 OS=Mus musculus OX=10090 GN=Etnk1 PE=1 SV=2 | Etnk1 | 0.99 |
| P42225 | Signal transducer and activator of transcription 1 OS=Mus musculus OX=10090 GN=Stat1 PE=1 SV=1 | Stat1 | 0.99 |
| P63323 | 40S ribosomal protein S12 OS=Mus musculus OX=10090 GN=Rps12 PE=1 SV=2 | Rps12 | 0.99 |
| O88942 | Nuclear factor of activated T-cells, cytoplasmic 1 OS=Mus musculus OX=10090 GN=Nfatc1 PE=1 SV=1 | Nfatc1 | 0.99 |
| Q9D9V3 | Ethylmalonyl-CoA decarboxylase OS=Mus musculus OX=10090 GN=Echdc1 PE=1 SV=2 | Echdc1 | 0.99 |
| P63073 | Eukaryotic translation initiation factor 4E OS=Mus musculus OX=10090 GN=Eif4e PE=1 SV=1 | Eif4e | 0.99 |
| O35386 | Phytanoyl-CoA dioxygenase, peroxisomal OS=Mus musculus OX=10090 GN=Phyh PE=1 SV=1 | Phyh | 0.99 |
| Q8VEK3 | Heterogeneous nuclear ribonucleoprotein U OS=Mus musculus OX=10090 GN=Hnrnpu PE=1 SV=1 | Hnrnpu | 0.99 |
| P40142 | Transketolase OS=Mus musculus OX=10090 GN=Tkt PE=1 SV=1 | Tkt | 0.99 |
| E9QAT4 | Protein transport protein Sec16A OS=Mus musculus OX=10090 GN=Sec16a PE=1 SV=1 | Sec16a | 0.99 |
| Q60680 | Inhibitor of nuclear factor kappa-B kinase subunit alpha OS=Mus musculus OX=10090 GN=Chuk PE=1 SV=1 | Chuk | 0.99 |
| Q80XR2 | Calcium-transporting ATPase type 2C member 1 OS=Mus musculus OX=10090 GN=Atp2c1 PE=1 SV=2 | Atp2c1 | 0.99 |
| Q6ZPY2 | Protein SMG5 OS=Mus musculus OX=10090 GN=Smg5 PE=1 SV=2 | Smg5 | 0.99 |
| Q9CR11 | YEATS domain-containing protein 4 OS=Mus musculus OX=10090 GN=Yeats4 PE=2 SV=1 | Yeats4 | 0.99 |
| Q9JLB0 | Protein PALS2 OS=Mus musculus OX=10090 GN=Pals2 PE=1 SV=1 | Pals2 | 0.99 |
| P25206 | DNA replication licensing factor MCM3 OS=Mus musculus OX=10090 GN=Mcm3 PE=1 SV=2 | Mcm3 | 0.99 |
| Q8BWZ3 | N-alpha-acetyltransferase 25, NatB auxiliary subunit OS=Mus musculus OX=10090 GN=Naa25 PE=1 SV=1 | Naa25 | 0.99 |
| Q9D3D9 | ATP synthase subunit delta, mitochondrial OS=Mus musculus OX=10090 GN=Atp5f1d PE=1 SV=1 | Atp5f1d | 0.99 |
| Q9CQA1 | Trafficking protein particle complex subunit 5 OS=Mus musculus OX=10090 GN=Trappc5 PE=1 SV=1 | Trappc5 | 0.99 |
| Q9CY97 | RNA polymerase II subunit A C-terminal domain phosphatase SSU72 OS=Mus musculus OX=10090 GN=Ssu72 PE=1 SV=1 | Ssu72 | 0.99 |
| P16675 | Lysosomal protective protein OS=Mus musculus OX=10090 GN=Ctsa PE=1 SV=1 | Ctsa | 0.99 |
| Q8K296 | Myotubularin-related protein 3 OS=Mus musculus OX=10090 GN=Mtmr3 PE=1 SV=2 | Mtmr3 | 0.99 |
| P52480 | Pyruvate kinase PKM OS=Mus musculus OX=10090 GN=Pkm PE=1 SV=4 | Pkm | 0.99 |
| Q99L04 | Dehydrogenase/reductase SDR family member 1 OS=Mus musculus OX=10090 GN=Dhrs1 PE=1 SV=1 | Dhrs1 | 0.99 |
| Q61686 | Chromobox protein homolog 5 OS=Mus musculus OX=10090 GN=Cbx5 PE=1 SV=1 | Cbx5 | 0.99 |
| Q9DBR1 | 5'-3' exoribonuclease 2 OS=Mus musculus OX=10090 GN=Xrn2 PE=1 SV=1 | Xrn2 | 0.99 |
| Q61335 | B-cell receptor-associated protein 31 OS=Mus musculus OX=10090 GN=Bcap31 PE=1 SV=4 | Bcap31 | 0.99 |
| Q9D061 | Acyl-CoA-binding domain-containing protein 6 OS=Mus musculus OX=10090 GN=Acbd6 PE=1 SV=2 | Acbd6 | 0.99 |
| P59941 | NAD-dependent protein deacetylase sirtuin-6 OS=Mus musculus OX=10090 GN=Sirt6 PE=1 SV=1 | Sirt6 | 0.99 |
| O35643 | AP-1 complex subunit beta-1 OS=Mus musculus OX=10090 GN=Ap1b1 PE=1 SV=2 | Ap1b1 | 0.99 |
| Q8K274 | Ketosamine-3-kinase OS=Mus musculus OX=10090 GN=Fn3krp PE=1 SV=2 | Fn3krp | 0.99 |
| Q99P21 | Adenine DNA glycosylase OS=Mus musculus OX=10090 GN=Mutyh PE=1 SV=2 | Mutyh | 1.00 |
| Q8JZR0 | Long-chain-fatty-acid--CoA ligase 5 OS=Mus musculus OX=10090 GN=Acsl5 PE=1 SV=1 | Acsl5 | 1.00 |
| Q9D0F9 | Phosphoglucomutase-1 OS=Mus musculus OX=10090 GN=Pgm1 PE=1 SV=4 | Pgm1 | 1.00 |
| O35375 | Neuropilin-2 OS=Mus musculus OX=10090 GN=Nrp2 PE=1 SV=2 | Nrp2 | 1.00 |
| Q9JL60 | Glucocorticoid modulatory element-binding protein 1 OS=Mus musculus OX=10090 GN=Gmeb1 PE=1 SV=2 | Gmeb1 | 1.00 |
| Q8CEE7 | Retinol dehydrogenase 13 OS=Mus musculus OX=10090 GN=Rdh13 PE=1 SV=1 | Rdh13 | 1.00 |
| Q8BHG1 | Nardilysin OS=Mus musculus OX=10090 GN=Nrdc PE=1 SV=1 | Nrdc | 1.00 |
| Q9D1Q6 | Endoplasmic reticulum resident protein 44 OS=Mus musculus OX=10090 GN=Erp44 PE=1 SV=1 | Erp44 | 1.00 |
| Q66JS6 | Eukaryotic translation initiation factor 3 subunit J-B OS=Mus musculus OX=10090 GN=Eif3j2 PE=1 SV=1 | Eif3j2 | 1.00 |
| Q9D4H2 | GRIP and coiled-coil domain-containing protein 1 OS=Mus musculus OX=10090 GN=Gcc1 PE=1 SV=2 | Gcc1 | 1.00 |
| P47811 | Mitogen-activated protein kinase 14 OS=Mus musculus OX=10090 GN=Mapk14 PE=1 SV=3 | Mapk14 | 1.00 |
| Q9Z1T1 | AP-3 complex subunit beta-1 OS=Mus musculus OX=10090 GN=Ap3b1 PE=1 SV=2 | Ap3b1 | 1.00 |
| Q9CQY5 | Magnesium transporter protein 1 OS=Mus musculus OX=10090 GN=Magt1 PE=1 SV=1 | Magt1 | 1.00 |
| Q91ZU1 | Ankyrin repeat and SOCS box protein 6 OS=Mus musculus OX=10090 GN=Asb6 PE=1 SV=1 | Asb6 | 1.00 |
| Q920Q6 | RNA-binding protein Musashi homolog 2 OS=Mus musculus OX=10090 GN=Msi2 PE=1 SV=1 | Msi2 | 1.00 |
| Q9D880 | Mitochondrial import inner membrane translocase subunit TIM50 OS=Mus musculus OX=10090 GN=Timm50 PE=1 SV=1 | Timm50 | 1.00 |
| P57716 | Nicastrin OS=Mus musculus OX=10090 GN=Ncstn PE=1 SV=3 | Ncstn | 1.00 |
| Q9ES46 | Beta-parvin OS=Mus musculus OX=10090 GN=Parvb PE=1 SV=1 | Parvb | 1.00 |
| O88291 | DBIRD complex subunit ZNF326 OS=Mus musculus OX=10090 GN=Znf326 PE=1 SV=1 | Znf326 | 1.00 |
| P97358 | TATA box-binding protein-associated factor RNA polymerase I subunit B OS=Mus musculus OX=10090 GN=Taf1b PE=1 SV=2 | Taf1b | 1.00 |
| Q9CSH3 | Exosome complex exonuclease RRP44 OS=Mus musculus OX=10090 GN=Dis3 PE=1 SV=4 | Dis3 | 1.00 |
| Q9D2N9 | Vacuolar protein sorting-associated protein 33A OS=Mus musculus OX=10090 GN=Vps33a PE=1 SV=2 | Vps33a | 1.00 |
| Q9JHU4 | Cytoplasmic dynein 1 heavy chain 1 OS=Mus musculus OX=10090 GN=Dync1h1 PE=1 SV=2 | Dync1h1 | 1.00 |
| O88879 | Apoptotic protease-activating factor 1 OS=Mus musculus OX=10090 GN=Apaf1 PE=1 SV=3 | Apaf1 | 1.00 |
| Q9D964 | Glycine amidinotransferase, mitochondrial OS=Mus musculus OX=10090 GN=Gatm PE=1 SV=1 | Gatm | 1.00 |
| P22892 | AP-1 complex subunit gamma-1 OS=Mus musculus OX=10090 GN=Ap1g1 PE=1 SV=3 | Ap1g1 | 1.00 |
| Q7TT50 | Serine/threonine-protein kinase MRCK beta OS=Mus musculus OX=10090 GN=Cdc42bpb PE=1 SV=2 | Cdc42bpb | 1.00 |
| Q8BIG4 | F-box only protein 28 OS=Mus musculus OX=10090 GN=Fbxo28 PE=1 SV=1 | Fbxo28 | 1.00 |
| Q9D7G0 | Ribose-phosphate pyrophosphokinase 1 OS=Mus musculus OX=10090 GN=Prps1 PE=1 SV=4 | Prps1 | 1.00 |
| E9Q5K9 | YTH domain-containing protein 1 OS=Mus musculus OX=10090 GN=Ythdc1 PE=1 SV=2 | Ythdc1 | 1.00 |
| Q6NVE8 | WD repeat-containing protein 44 OS=Mus musculus OX=10090 GN=Wdr44 PE=1 SV=1 | Wdr44 | 1.00 |
| Q3TLH4 | Protein PRRC2C OS=Mus musculus OX=10090 GN=Prrc2c PE=1 SV=3 | Prrc2c | 1.00 |
| Q923D2 | Flavin reductase (NADPH) OS=Mus musculus OX=10090 GN=Blvrb PE=1 SV=3 | Blvrb | 1.00 |
| Q9D820 | Prolyl-tRNA synthetase associated domain-containing protein 1 OS=Mus musculus OX=10090 GN=Prorsd1 PE=1 SV=1 | Prorsd1 | 1.00 |
| Q8R151 | NFX1-type zinc finger-containing protein 1 OS=Mus musculus OX=10090 GN=Znfx1 PE=1 SV=3 | Znfx1 | 1.00 |
| Q9QXZ0 | Microtubule-actin cross-linking factor 1 OS=Mus musculus OX=10090 GN=Macf1 PE=1 SV=2 | Macf1 | 1.00 |
| Q6DFX2 | Anthrax toxin receptor 2 OS=Mus musculus OX=10090 GN=Antxr2 PE=1 SV=1 | Antxr2 | 1.00 |
| E9PVA8 | eIF-2-alpha kinase activator GCN1 OS=Mus musculus OX=10090 GN=Gcn1 PE=1 SV=1 | Gcn1 | 1.00 |
| Q60930 | Voltage-dependent anion-selective channel protein 2 OS=Mus musculus OX=10090 GN=Vdac2 PE=1 SV=2 | Vdac2 | 1.00 |
| Q9D906 | Ubiquitin-like modifier-activating enzyme ATG7 OS=Mus musculus OX=10090 GN=Atg7 PE=1 SV=1 | Atg7 | 1.00 |
| Q9D883 | Splicing factor U2AF 35 kDa subunit OS=Mus musculus OX=10090 GN=U2af1 PE=1 SV=4 | U2af1 | 1.00 |
| Q9CPQ1 | Cytochrome c oxidase subunit 6C OS=Mus musculus OX=10090 GN=Cox6c PE=1 SV=3 | Cox6c | 1.00 |
| O88544 | COP9 signalosome complex subunit 4 OS=Mus musculus OX=10090 GN=Cops4 PE=1 SV=1 | Cops4 | 1.00 |
| Q8CCF0 | U4/U6 small nuclear ribonucleoprotein Prp31 OS=Mus musculus OX=10090 GN=Prpf31 PE=1 SV=3 | Prpf31 | 1.00 |
| O70566 | Protein diaphanous homolog 2 OS=Mus musculus OX=10090 GN=Diaph2 PE=1 SV=2 | Diaph2 | 1.00 |
| Q6ZWV3 | 60S ribosomal protein L10 OS=Mus musculus OX=10090 GN=Rpl10 PE=1 SV=3 | Rpl10 | 1.00 |
| Q6GQT9 | Nodal modulator 1 OS=Mus musculus OX=10090 GN=Nomo1 PE=1 SV=1 | Nomo1 | 1.00 |
| Q9Z2D6 | Methyl-CpG-binding protein 2 OS=Mus musculus OX=10090 GN=Mecp2 PE=1 SV=1 | Mecp2 | 1.00 |
| Q8K4Z5 | Splicing factor 3A subunit 1 OS=Mus musculus OX=10090 GN=Sf3a1 PE=1 SV=1 | Sf3a1 | 1.00 |
| Q9Z110 | Delta-1-pyrroline-5-carboxylate synthase OS=Mus musculus OX=10090 GN=Aldh18a1 PE=1 SV=2 | Aldh18a1 | 1.00 |
| Q9DD02 | Protein Hikeshi OS=Mus musculus OX=10090 GN=Hikeshi PE=1 SV=1 | Hikeshi | 1.00 |
| P62743 | AP-2 complex subunit sigma OS=Mus musculus OX=10090 GN=Ap2s1 PE=1 SV=1 | Ap2s1 | 1.00 |
| Q99MI1 | ELKS/Rab6-interacting/CAST family member 1 OS=Mus musculus OX=10090 GN=Erc1 PE=1 SV=1 | Erc1 | 1.00 |
| Q9CWV6 | PRKR-interacting protein 1 OS=Mus musculus OX=10090 GN=Prkrip1 PE=1 SV=2 | Prkrip1 | 1.00 |
| Q99N69 | Leupaxin OS=Mus musculus OX=10090 GN=Lpxn PE=1 SV=2 | Lpxn | 1.00 |
| P68037 | Ubiquitin-conjugating enzyme E2 L3 OS=Mus musculus OX=10090 GN=Ube2l3 PE=1 SV=1 | Ube2l3 | 1.00 |
| Q9D8N0 | Elongation factor 1-gamma OS=Mus musculus OX=10090 GN=Eef1g PE=1 SV=3 | Eef1g | 1.00 |
| Q9QXB9 | Developmentally-regulated GTP-binding protein 2 OS=Mus musculus OX=10090 GN=Drg2 PE=1 SV=1 | Drg2 | 1.00 |
| P54071 | Isocitrate dehydrogenase [NADP], mitochondrial OS=Mus musculus OX=10090 GN=Idh2 PE=1 SV=3 | Idh2 | 1.00 |
| P47911 | 60S ribosomal protein L6 OS=Mus musculus OX=10090 GN=Rpl6 PE=1 SV=3 | Rpl6 | 1.00 |
| O35654 | DNA polymerase delta subunit 2 OS=Mus musculus OX=10090 GN=Pold2 PE=1 SV=2 | Pold2 | 1.00 |
| Q8BG60 | Thioredoxin-interacting protein OS=Mus musculus OX=10090 GN=Txnip PE=1 SV=1 | Txnip | 1.00 |
| Q8VCM8 | Nicalin OS=Mus musculus OX=10090 GN=Ncln PE=1 SV=2 | Ncln | 1.00 |
| Q9JLM4 | Zinc finger MYM-type protein 3 OS=Mus musculus OX=10090 GN=Zmym3 PE=1 SV=1 | Zmym3 | 1.00 |
| Q8C092 | Transcription initiation factor TFIID subunit 5 OS=Mus musculus OX=10090 GN=Taf5 PE=1 SV=1 | Taf5 | 1.00 |
| Q9Z1E4 | Glycogen [starch] synthase, muscle OS=Mus musculus OX=10090 GN=Gys1 PE=1 SV=2 | Gys1 | 1.00 |
| Q8BYK6 | YTH domain-containing family protein 3 OS=Mus musculus OX=10090 GN=Ythdf3 PE=1 SV=2 | Ythdf3 | 1.00 |
| Q9CQX2 | Cytochrome b5 type B OS=Mus musculus OX=10090 GN=Cyb5b PE=1 SV=1 | Cyb5b | 1.00 |
| Q9CQW0 | ER membrane protein complex subunit 6 OS=Mus musculus OX=10090 GN=Emc6 PE=1 SV=1 | Emc6 | 1.00 |
| Q6A009 | E3 ubiquitin-protein ligase listerin OS=Mus musculus OX=10090 GN=Ltn1 PE=1 SV=3 | Ltn1 | 1.00 |
| Q8BG30 | Negative elongation factor A OS=Mus musculus OX=10090 GN=Nelfa PE=1 SV=1 | Nelfa | 1.00 |
| Q91XD7 | Protein disulfide isomerase Creld1 OS=Mus musculus OX=10090 GN=Creld1 PE=1 SV=1 | Creld1 | 1.00 |
| Q8CDM1 | ATPase family AAA domain-containing protein 2 OS=Mus musculus OX=10090 GN=Atad2 PE=1 SV=1 | Atad2 | 1.00 |
| P28033 | CCAAT/enhancer-binding protein beta OS=Mus musculus OX=10090 GN=Cebpb PE=1 SV=1 | Cebpb | 1.00 |
| Q2TBE6 | Phosphatidylinositol 4-kinase type 2-alpha OS=Mus musculus OX=10090 GN=Pi4k2a PE=1 SV=1 | Pi4k2a | 1.00 |
| P26450 | Phosphatidylinositol 3-kinase regulatory subunit alpha OS=Mus musculus OX=10090 GN=Pik3r1 PE=1 SV=2 | Pik3r1 | 1.00 |
| Q05816 | Fatty acid-binding protein 5 OS=Mus musculus OX=10090 GN=Fabp5 PE=1 SV=3 | Fabp5 | 1.00 |
| Q99PM9 | Uridine-cytidine kinase 2 OS=Mus musculus OX=10090 GN=Uck2 PE=1 SV=1 | Uck2 | 1.00 |
| Q9JL56 | Glycerophosphodiester phosphodiesterase 1 OS=Mus musculus OX=10090 GN=Gde1 PE=1 SV=1 | Gde1 | 1.00 |
| P62342 | Thioredoxin reductase-like selenoprotein T OS=Mus musculus OX=10090 GN=Selenot PE=1 SV=2 | Selenot | 1.00 |
| Q9CWX9 | Probable ATP-dependent RNA helicase DDX47 OS=Mus musculus OX=10090 GN=Ddx47 PE=2 SV=2 | Ddx47 | 1.00 |
| P08228 | Superoxide dismutase [Cu-Zn] OS=Mus musculus OX=10090 GN=Sod1 PE=1 SV=2 | Sod1 | 1.00 |
| Q9CQG2 | RNA N6-adenosine-methyltransferase METTL16 OS=Mus musculus OX=10090 GN=Mettl16 PE=1 SV=1 | Mettl16 | 1.00 |
| P59328 | WD repeat and HMG-box DNA-binding protein 1 OS=Mus musculus OX=10090 GN=Wdhd1 PE=1 SV=2 | Wdhd1 | 1.00 |
| Q3ULD5 | Methylcrotonoyl-CoA carboxylase beta chain, mitochondrial OS=Mus musculus OX=10090 GN=Mccc2 PE=1 SV=1 | Mccc2 | 1.00 |
| Q91V61 | Sideroflexin-3 OS=Mus musculus OX=10090 GN=Sfxn3 PE=1 SV=1 | Sfxn3 | 1.00 |
| B9EJR8 | Dynein axonemal assembly factor 5 OS=Mus musculus OX=10090 GN=Dnaaf5 PE=1 SV=1 | Dnaaf5 | 1.00 |
| P47226 | Testin OS=Mus musculus OX=10090 GN=Tes PE=1 SV=1 | Tes | 1.00 |
| Q80UP5 | Ankyrin repeat domain-containing protein 13A OS=Mus musculus OX=10090 GN=Ankrd13a PE=1 SV=2 | Ankrd13a | 1.00 |
| A0A338P6K9 | Glutamine and serine-rich protein 1 OS=Mus musculus OX=10090 GN=Qser1 PE=2 SV=1 | Qser1 | 1.00 |
| Q8K2C7 | Protein OS-9 OS=Mus musculus OX=10090 GN=Os9 PE=1 SV=2 | Os9 | 1.00 |
| Q99M31 | Heat shock 70 kDa protein 14 OS=Mus musculus OX=10090 GN=Hspa14 PE=1 SV=2 | Hspa14 | 1.00 |
| Q80UJ7 | Rab3 GTPase-activating protein catalytic subunit OS=Mus musculus OX=10090 GN=Rab3gap1 PE=1 SV=4 | Rab3gap1 | 1.00 |
| E9PYL2 | Proline-rich protein 12 OS=Mus musculus OX=10090 GN=Prr12 PE=1 SV=1 | Prr12 | 1.00 |
| Q80UP3 | Diacylglycerol kinase zeta OS=Mus musculus OX=10090 GN=Dgkz PE=1 SV=2 | Dgkz | 1.00 |
| Q62425 | Cytochrome c oxidase subunit NDUFA4 OS=Mus musculus OX=10090 GN=Ndufa4 PE=1 SV=2 | Ndufa4 | 1.00 |
| P48193 | Protein 4.1 OS=Mus musculus OX=10090 GN=Epb41 PE=1 SV=2 | Epb41 | 1.00 |
| O08585 | Clathrin light chain A OS=Mus musculus OX=10090 GN=Clta PE=1 SV=2 | Clta | 1.00 |
| Q91YJ3 | Thymocyte nuclear protein 1 OS=Mus musculus OX=10090 GN=Thyn1 PE=1 SV=1 | Thyn1 | 1.00 |
| Q3V3R1 | Monofunctional C1-tetrahydrofolate synthase, mitochondrial OS=Mus musculus OX=10090 GN=Mthfd1l PE=1 SV=2 | Mthfd1l | 1.00 |
| O35857 | Mitochondrial import inner membrane translocase subunit TIM44 OS=Mus musculus OX=10090 GN=Timm44 PE=1 SV=2 | Timm44 | 1.00 |
| Q91VN6 | Probable ATP-dependent RNA helicase DDX41 OS=Mus musculus OX=10090 GN=Ddx41 PE=1 SV=2 | Ddx41 | 1.00 |
| Q6ZQ38 | Cullin-associated NEDD8-dissociated protein 1 OS=Mus musculus OX=10090 GN=Cand1 PE=1 SV=2 | Cand1 | 1.00 |
| Q8BP48 | Methionine aminopeptidase 1 OS=Mus musculus OX=10090 GN=Metap1 PE=1 SV=1 | Metap1 | 1.00 |
| Q8BG51 | Mitochondrial Rho GTPase 1 OS=Mus musculus OX=10090 GN=Rhot1 PE=1 SV=1 | Rhot1 | 1.00 |
| Q9CQZ0 | ORM1-like protein 2 OS=Mus musculus OX=10090 GN=Ormdl2 PE=1 SV=1 | Ormdl2 | 1.00 |
| Q6NZC7 | SEC23-interacting protein OS=Mus musculus OX=10090 GN=Sec23ip PE=1 SV=2 | Sec23ip | 1.00 |
| O35737 | Heterogeneous nuclear ribonucleoprotein H OS=Mus musculus OX=10090 GN=Hnrnph1 PE=1 SV=3 | Hnrnph1 | 1.00 |
| Q9DCD2 | Pre-mRNA-splicing factor SYF1 OS=Mus musculus OX=10090 GN=Xab2 PE=1 SV=1 | Xab2 | 1.00 |
| Q920B9 | FACT complex subunit SPT16 OS=Mus musculus OX=10090 GN=Supt16h PE=1 SV=2 | Supt16h | 1.00 |
| P35980 | 60S ribosomal protein L18 OS=Mus musculus OX=10090 GN=Rpl18 PE=1 SV=3 | Rpl18 | 1.00 |
| Q6P2B1 | Transportin-3 OS=Mus musculus OX=10090 GN=Tnpo3 PE=1 SV=1 | Tnpo3 | 1.01 |
| Q9D0F3 | Protein ERGIC-53 OS=Mus musculus OX=10090 GN=Lman1 PE=1 SV=1 | Lman1 | 1.01 |
| Q8BK63 | Casein kinase I isoform alpha OS=Mus musculus OX=10090 GN=Csnk1a1 PE=1 SV=2 | Csnk1a1 | 1.01 |
| Q8BRN9 | Coiled-coil and C2 domain-containing protein 1B OS=Mus musculus OX=10090 GN=Cc2d1b PE=1 SV=1 | Cc2d1b | 1.01 |
| Q9D8M4 | 60S ribosomal protein L7-like 1 OS=Mus musculus OX=10090 GN=Rpl7l1 PE=1 SV=1 | Rpl7l1 | 1.01 |
| Q8R5M8 | Cell adhesion molecule 1 OS=Mus musculus OX=10090 GN=Cadm1 PE=1 SV=2 | Cadm1 | 1.01 |
| Q3TZX8 | Polynucleotide 5'-hydroxyl-kinase NOL9 OS=Mus musculus OX=10090 GN=Nol9 PE=1 SV=1 | Nol9 | 1.01 |
| P26323 | Friend leukemia integration 1 transcription factor OS=Mus musculus OX=10090 GN=Fli1 PE=1 SV=1 | Fli1 | 1.01 |
| O55222 | Integrin-linked protein kinase OS=Mus musculus OX=10090 GN=Ilk PE=1 SV=2 | Ilk | 1.01 |
| Q8R2R6 | Mitochondrial ribosome-associated GTPase 1 OS=Mus musculus OX=10090 GN=Mtg1 PE=1 SV=2 | Mtg1 | 1.01 |
| Q61699 | Heat shock protein 105 kDa OS=Mus musculus OX=10090 GN=Hsph1 PE=1 SV=2 | Hsph1 | 1.01 |
| Q9D7X3 | Dual specificity protein phosphatase 3 OS=Mus musculus OX=10090 GN=Dusp3 PE=1 SV=1 | Dusp3 | 1.01 |
| E9Q634 | Unconventional myosin-Ie OS=Mus musculus OX=10090 GN=Myo1e PE=1 SV=1 | Myo1e | 1.01 |
| Q8BUV3 | Gephyrin OS=Mus musculus OX=10090 GN=Gphn PE=1 SV=2 | Gphn | 1.01 |
| Q8K297 | Procollagen galactosyltransferase 1 OS=Mus musculus OX=10090 GN=Colgalt1 PE=1 SV=2 | Colgalt1 | 1.01 |
| Q9Z1R2 | Large proline-rich protein BAG6 OS=Mus musculus OX=10090 GN=Bag6 PE=1 SV=1 | Bag6 | 1.01 |
| Q9DBC3 | Cap-specific mRNA (nucleoside-2'-O-)-methyltransferase 1 OS=Mus musculus OX=10090 GN=Cmtr1 PE=1 SV=1 | Cmtr1 | 1.01 |
| Q6WKZ8 | E3 ubiquitin-protein ligase UBR2 OS=Mus musculus OX=10090 GN=Ubr2 PE=1 SV=2 | Ubr2 | 1.01 |
| P09405 | Nucleolin OS=Mus musculus OX=10090 GN=Ncl PE=1 SV=2 | Ncl | 1.01 |
| Q8CFE6 | Sodium-coupled neutral amino acid transporter 2 OS=Mus musculus OX=10090 GN=Slc38a2 PE=1 SV=1 | Slc38a2 | 1.01 |
| A2RSX7 | tRNA wybutosine-synthesizing protein 5 OS=Mus musculus OX=10090 GN=Tyw5 PE=2 SV=2 | Tyw5 | 1.01 |
| Q8K298 | Anillin OS=Mus musculus OX=10090 GN=Anln PE=1 SV=2 | Anln | 1.01 |
| Q8VCH5 | Rab9 effector protein with kelch motifs OS=Mus musculus OX=10090 GN=Rabepk PE=1 SV=2 | Rabepk | 1.01 |
| Q9JLV2 | Short transient receptor potential channel 4-associated protein OS=Mus musculus OX=10090 GN=Trpc4ap PE=1 SV=2 | Trpc4ap | 1.01 |
| Q9CQE3 | 28S ribosomal protein S17, mitochondrial OS=Mus musculus OX=10090 GN=Mrps17 PE=1 SV=1 | Mrps17 | 1.01 |
| P26350 | Prothymosin alpha OS=Mus musculus OX=10090 GN=Ptma PE=1 SV=2 | Ptma | 1.01 |
| Q9Z179 | SHC SH2 domain-binding protein 1 OS=Mus musculus OX=10090 GN=Shcbp1 PE=1 SV=1 | Shcbp1 | 1.01 |
| P63087 | Serine/threonine-protein phosphatase PP1-gamma catalytic subunit OS=Mus musculus OX=10090 GN=Ppp1cc PE=1 SV=1 | Ppp1cc | 1.01 |
| Q91WJ8 | Far upstream element-binding protein 1 OS=Mus musculus OX=10090 GN=Fubp1 PE=1 SV=1 | Fubp1 | 1.01 |
| P70398 | Probable ubiquitin carboxyl-terminal hydrolase FAF-X OS=Mus musculus OX=10090 GN=Usp9x PE=1 SV=2 | Usp9x | 1.01 |
| Q99N95 | 39S ribosomal protein L3, mitochondrial OS=Mus musculus OX=10090 GN=Mrpl3 PE=1 SV=1 | Mrpl3 | 1.01 |
| O54946 | DnaJ homolog subfamily B member 6 OS=Mus musculus OX=10090 GN=Dnajb6 PE=1 SV=4 | Dnajb6 | 1.01 |
| Q8C407 | Protein YIPF4 OS=Mus musculus OX=10090 GN=Yipf4 PE=1 SV=1 | Yipf4 | 1.01 |
| Q3TBD2 | Rho GTPase-activating protein 45 OS=Mus musculus OX=10090 GN=Arhgap45 PE=1 SV=2 | Arhgap45 | 1.01 |
| P50580 | Proliferation-associated protein 2G4 OS=Mus musculus OX=10090 GN=Pa2g4 PE=1 SV=3 | Pa2g4 | 1.01 |
| Q99PN3 | Tripartite motif-containing protein 26 OS=Mus musculus OX=10090 GN=Trim26 PE=2 SV=3 | Trim26 | 1.01 |
| P35486 | Pyruvate dehydrogenase E1 component subunit alpha, somatic form, mitochondrial OS=Mus musculus OX=10090 GN=Pdha1 PE=1 SV=1 | Pdha1 | 1.01 |
| Q91WG4 | Elongator complex protein 2 OS=Mus musculus OX=10090 GN=Elp2 PE=1 SV=1 | Elp2 | 1.01 |
| P30999 | Catenin delta-1 OS=Mus musculus OX=10090 GN=Ctnnd1 PE=1 SV=2 | Ctnnd1 | 1.01 |
| Q9CWE0 | Mitochondrial fission regulator 1-like OS=Mus musculus OX=10090 GN=Mtfr1l PE=1 SV=1 | Mtfr1l | 1.01 |
| Q9CQV5 | 28S ribosomal protein S24, mitochondrial OS=Mus musculus OX=10090 GN=Mrps24 PE=1 SV=1 | Mrps24 | 1.01 |
| Q91W96 | Anaphase-promoting complex subunit 4 OS=Mus musculus OX=10090 GN=Anapc4 PE=1 SV=1 | Anapc4 | 1.01 |
| Q7TQ95 | Endoplasmic reticulum junction formation protein lunapark OS=Mus musculus OX=10090 GN=Lnpk PE=1 SV=1 | Lnpk | 1.01 |
| P62855 | 40S ribosomal protein S26 OS=Mus musculus OX=10090 GN=Rps26 PE=1 SV=3 | Rps26 | 1.01 |
| Q8VCF0 | Mitochondrial antiviral-signaling protein OS=Mus musculus OX=10090 GN=Mavs PE=1 SV=1 | Mavs | 1.01 |
| Q3TPE9 | Ankyrin repeat and MYND domain-containing protein 2 OS=Mus musculus OX=10090 GN=Ankmy2 PE=1 SV=1 | Ankmy2 | 1.01 |
| O89001 | Carboxypeptidase D OS=Mus musculus OX=10090 GN=Cpd PE=1 SV=2 | Cpd | 1.01 |
| P20029 | Endoplasmic reticulum chaperone BiP OS=Mus musculus OX=10090 GN=Hspa5 PE=1 SV=3 | Hspa5 | 1.01 |
| Q9DB40 | Mediator of RNA polymerase II transcription subunit 27 OS=Mus musculus OX=10090 GN=Med27 PE=1 SV=2 | Med27 | 1.01 |
| Q5SWD9 | Pre-rRNA-processing protein TSR1 homolog OS=Mus musculus OX=10090 GN=Tsr1 PE=1 SV=1 | Tsr1 | 1.01 |
| P07901 | Heat shock protein HSP 90-alpha OS=Mus musculus OX=10090 GN=Hsp90aa1 PE=1 SV=4 | Hsp90aa1 | 1.01 |
| Q91YR5 | eEF1A lysine and N-terminal methyltransferase OS=Mus musculus OX=10090 GN=Mettl13 PE=1 SV=1 | Mettl13 | 1.01 |
| Q9WTY1 | Programmed cell death protein 7 OS=Mus musculus OX=10090 GN=Pdcd7 PE=1 SV=1 | Pdcd7 | 1.01 |
| O09172 | Glutamate--cysteine ligase regulatory subunit OS=Mus musculus OX=10090 GN=Gclm PE=1 SV=1 | Gclm | 1.01 |
| Q9D4H1 | Exocyst complex component 2 OS=Mus musculus OX=10090 GN=Exoc2 PE=1 SV=1 | Exoc2 | 1.01 |
| Q9CR61 | NADH dehydrogenase [ubiquinone] 1 beta subcomplex subunit 7 OS=Mus musculus OX=10090 GN=Ndufb7 PE=1 SV=3 | Ndufb7 | 1.01 |
| Q8CHY3 | Dymeclin OS=Mus musculus OX=10090 GN=Dym PE=1 SV=1 | Dym | 1.01 |
| Q8QZY1 | Eukaryotic translation initiation factor 3 subunit L OS=Mus musculus OX=10090 GN=Eif3l PE=1 SV=1 | Eif3l | 1.01 |
| Q6P5E4 | UDP-glucose:glycoprotein glucosyltransferase 1 OS=Mus musculus OX=10090 GN=Uggt1 PE=1 SV=4 | Uggt1 | 1.01 |
| Q9CY27 | Very-long-chain enoyl-CoA reductase OS=Mus musculus OX=10090 GN=Tecr PE=1 SV=1 | Tecr | 1.01 |
| Q61316 | Heat shock 70 kDa protein 4 OS=Mus musculus OX=10090 GN=Hspa4 PE=1 SV=1 | Hspa4 | 1.01 |
| Q61584 | Fragile X mental retardation syndrome-related protein 1 OS=Mus musculus OX=10090 GN=Fxr1 PE=1 SV=2 | Fxr1 | 1.01 |
| P63037 | DnaJ homolog subfamily A member 1 OS=Mus musculus OX=10090 GN=Dnaja1 PE=1 SV=1 | Dnaja1 | 1.01 |
| O55098 | Serine/threonine-protein kinase 10 OS=Mus musculus OX=10090 GN=Stk10 PE=1 SV=2 | Stk10 | 1.01 |
| Q9Z0N1 | Eukaryotic translation initiation factor 2 subunit 3, X-linked OS=Mus musculus OX=10090 GN=Eif2s3x PE=1 SV=2 | Eif2s3x | 1.01 |
| Q60676 | Serine/threonine-protein phosphatase 5 OS=Mus musculus OX=10090 GN=Ppp5c PE=1 SV=3 | Ppp5c | 1.01 |
| Q9Z2X2 | 26S proteasome non-ATPase regulatory subunit 10 OS=Mus musculus OX=10090 GN=Psmd10 PE=1 SV=3 | Psmd10 | 1.01 |
| P99027 | 60S acidic ribosomal protein P2 OS=Mus musculus OX=10090 GN=Rplp2 PE=1 SV=3 | Rplp2 | 1.01 |
| Q8VHZ7 | U3 small nucleolar ribonucleoprotein protein IMP4 OS=Mus musculus OX=10090 GN=Imp4 PE=2 SV=1 | Imp4 | 1.01 |
| P19096 | Fatty acid synthase OS=Mus musculus OX=10090 GN=Fasn PE=1 SV=2 | Fasn | 1.01 |
| Q9WTM5 | RuvB-like 2 OS=Mus musculus OX=10090 GN=Ruvbl2 PE=1 SV=3 | Ruvbl2 | 1.01 |
| Q8R344 | Coiled-coil domain-containing protein 12 OS=Mus musculus OX=10090 GN=Ccdc12 PE=1 SV=2 | Ccdc12 | 1.01 |
| Q9CYR0 | Single-stranded DNA-binding protein, mitochondrial OS=Mus musculus OX=10090 GN=Ssbp1 PE=1 SV=1 | Ssbp1 | 1.01 |
| Q8CHT3 | Integrator complex subunit 5 OS=Mus musculus OX=10090 GN=Ints5 PE=2 SV=1 | Ints5 | 1.01 |
| Q923T9 | Calcium/calmodulin-dependent protein kinase type II subunit gamma OS=Mus musculus OX=10090 GN=Camk2g PE=1 SV=1 | Camk2g | 1.01 |
| Q9CWU6 | Ubiquinol-cytochrome-c reductase complex assembly factor 1 OS=Mus musculus OX=10090 GN=Uqcc1 PE=1 SV=1 | Uqcc1 | 1.01 |
| Q8CCM6 | Mitochondrial import inner membrane translocase subunit Tim21 OS=Mus musculus OX=10090 GN=Timm21 PE=1 SV=2 | Timm21 | 1.01 |
| P54775 | 26S proteasome regulatory subunit 6B OS=Mus musculus OX=10090 GN=Psmc4 PE=1 SV=2 | Psmc4 | 1.01 |
| Q6PAQ4 | RNA exonuclease 4 OS=Mus musculus OX=10090 GN=Rexo4 PE=2 SV=2 | Rexo4 | 1.01 |
| Q99PU8 | ATP-dependent RNA helicase DHX30 OS=Mus musculus OX=10090 GN=Dhx30 PE=1 SV=1 | Dhx30 | 1.01 |
| Q9QYI3 | DnaJ homolog subfamily C member 7 OS=Mus musculus OX=10090 GN=Dnajc7 PE=1 SV=2 | Dnajc7 | 1.01 |
| Q8CHY6 | Transcriptional repressor p66 alpha OS=Mus musculus OX=10090 GN=Gatad2a PE=1 SV=2 | Gatad2a | 1.01 |
| Q3TW96 | UDP-N-acetylhexosamine pyrophosphorylase-like protein 1 OS=Mus musculus OX=10090 GN=Uap1l1 PE=1 SV=1 | Uap1l1 | 1.01 |
| Q6A0A2 | La-related protein 4B OS=Mus musculus OX=10090 GN=Larp4b PE=1 SV=2 | Larp4b | 1.01 |
| Q80SW1 | S-adenosylhomocysteine hydrolase-like protein 1 OS=Mus musculus OX=10090 GN=Ahcyl1 PE=1 SV=1 | Ahcyl1 | 1.01 |
| Q60875 | Rho guanine nucleotide exchange factor 2 OS=Mus musculus OX=10090 GN=Arhgef2 PE=1 SV=4 | Arhgef2 | 1.01 |
| P61089 | Ubiquitin-conjugating enzyme E2 N OS=Mus musculus OX=10090 GN=Ube2n PE=1 SV=1 | Ube2n | 1.01 |
| P35564 | Calnexin OS=Mus musculus OX=10090 GN=Canx PE=1 SV=1 | Canx | 1.01 |
| Q8R035 | Peptidyl-tRNA hydrolase ICT1, mitochondrial OS=Mus musculus OX=10090 GN=Mrpl58 PE=1 SV=1 | Mrpl58 | 1.01 |
| P62960 | Y-box-binding protein 1 OS=Mus musculus OX=10090 GN=Ybx1 PE=1 SV=3 | Ybx1 | 1.01 |
| Q3TJZ6 | Protein FAM98A OS=Mus musculus OX=10090 GN=Fam98a PE=1 SV=1 | Fam98a | 1.01 |
| P97369 | Neutrophil cytosol factor 4 OS=Mus musculus OX=10090 GN=Ncf4 PE=1 SV=2 | Ncf4 | 1.01 |
| Q62314 | Trans-Golgi network integral membrane protein 2 OS=Mus musculus OX=10090 GN=Tgoln2 PE=1 SV=1 | Tgoln2 | 1.01 |
| Q9JMA1 | Ubiquitin carboxyl-terminal hydrolase 14 OS=Mus musculus OX=10090 GN=Usp14 PE=1 SV=3 | Usp14 | 1.01 |
| Q921J4 | Ubiquitin-conjugating enzyme E2 S OS=Mus musculus OX=10090 GN=Ube2s PE=1 SV=1 | Ube2s | 1.01 |
| A2AIV8 | Caspase recruitment domain-containing protein 9 OS=Mus musculus OX=10090 GN=Card9 PE=1 SV=1 | Card9 | 1.01 |
| Q8VDG3 | Poly(A)-specific ribonuclease PARN OS=Mus musculus OX=10090 GN=Parn PE=1 SV=1 | Parn | 1.01 |
| P80314 | T-complex protein 1 subunit beta OS=Mus musculus OX=10090 GN=Cct2 PE=1 SV=4 | Cct2 | 1.01 |
| Q69ZK6 | Probable JmjC domain-containing histone demethylation protein 2C OS=Mus musculus OX=10090 GN=Jmjd1c PE=1 SV=3 | Jmjd1c | 1.01 |
| O35435 | Dihydroorotate dehydrogenase (quinone), mitochondrial OS=Mus musculus OX=10090 GN=Dhodh PE=1 SV=2 | Dhodh | 1.01 |
| P29341 | Polyadenylate-binding protein 1 OS=Mus musculus OX=10090 GN=Pabpc1 PE=1 SV=2 | Pabpc1 | 1.01 |
| Q80VA0 | N-acetylgalactosaminyltransferase 7 OS=Mus musculus OX=10090 GN=Galnt7 PE=1 SV=2 | Galnt7 | 1.01 |
| Q9CYG7 | Mitochondrial import receptor subunit TOM34 OS=Mus musculus OX=10090 GN=Tomm34 PE=1 SV=1 | Tomm34 | 1.01 |
| Q9R008 | Mevalonate kinase OS=Mus musculus OX=10090 GN=Mvk PE=1 SV=1 | Mvk | 1.01 |
| Q61490 | CD166 antigen OS=Mus musculus OX=10090 GN=Alcam PE=1 SV=3 | Alcam | 1.01 |
| O70551 | SRSF protein kinase 1 OS=Mus musculus OX=10090 GN=Srpk1 PE=1 SV=2 | Srpk1 | 1.01 |
| Q9Z1G3 | V-type proton ATPase subunit C 1 OS=Mus musculus OX=10090 GN=Atp6v1c1 PE=1 SV=4 | Atp6v1c1 | 1.01 |
| Q8BXZ1 | Protein disulfide-isomerase TMX3 OS=Mus musculus OX=10090 GN=Tmx3 PE=1 SV=2 | Tmx3 | 1.01 |
| Q91YQ5 | Dolichyl-diphosphooligosaccharide--protein glycosyltransferase subunit 1 OS=Mus musculus OX=10090 GN=Rpn1 PE=1 SV=1 | Rpn1 | 1.01 |
| Q9Z1N5 | Spliceosome RNA helicase Ddx39b OS=Mus musculus OX=10090 GN=Ddx39b PE=1 SV=1 | Ddx39b | 1.01 |
| O88643 | Serine/threonine-protein kinase PAK 1 OS=Mus musculus OX=10090 GN=Pak1 PE=1 SV=1 | Pak1 | 1.01 |
| Q3TWF6 | WD repeat-containing protein 70 OS=Mus musculus OX=10090 GN=Wdr70 PE=1 SV=1 | Wdr70 | 1.01 |
| P61759 | Prefoldin subunit 3 OS=Mus musculus OX=10090 GN=Vbp1 PE=1 SV=2 | Vbp1 | 1.01 |
| Q7TSS2 | Ubiquitin-conjugating enzyme E2 Q1 OS=Mus musculus OX=10090 GN=Ube2q1 PE=1 SV=2 | Ube2q1 | 1.02 |
| Q8K1M6 | Dynamin-1-like protein OS=Mus musculus OX=10090 GN=Dnm1l PE=1 SV=2 | Dnm1l | 1.02 |
| Q9Z2X8 | Kelch-like ECH-associated protein 1 OS=Mus musculus OX=10090 GN=Keap1 PE=1 SV=1 | Keap1 | 1.02 |
| Q8BJL0 | SWI/SNF-related matrix-associated actin-dependent regulator of chromatin subfamily A-like protein 1 OS=Mus musculus OX=10090 GN=Smarcal1 PE=1 SV=1 | Smarcal1 | 1.02 |
| Q6ZPY7 | Lysine-specific demethylase 3B OS=Mus musculus OX=10090 GN=Kdm3b PE=1 SV=3 | Kdm3b | 1.02 |
| O88559 | Menin OS=Mus musculus OX=10090 GN=Men1 PE=1 SV=2 | Men1 | 1.02 |
| Q99MU3 | Double-stranded RNA-specific adenosine deaminase OS=Mus musculus OX=10090 GN=Adar PE=1 SV=2 | Adar | 1.02 |
| P97351 | 40S ribosomal protein S3a OS=Mus musculus OX=10090 GN=Rps3a PE=1 SV=3 | Rps3a | 1.02 |
| O70370 | Cathepsin S OS=Mus musculus OX=10090 GN=Ctss PE=1 SV=2 | Ctss | 1.02 |
| Q9WVL0 | Maleylacetoacetate isomerase OS=Mus musculus OX=10090 GN=Gstz1 PE=1 SV=1 | Gstz1 | 1.02 |
| Q99P88 | Nuclear pore complex protein Nup155 OS=Mus musculus OX=10090 GN=Nup155 PE=1 SV=1 | Nup155 | 1.02 |
| O08529 | Calpain-2 catalytic subunit OS=Mus musculus OX=10090 GN=Capn2 PE=1 SV=4 | Capn2 | 1.02 |
| Q9D8S3 | ADP-ribosylation factor GTPase-activating protein 3 OS=Mus musculus OX=10090 GN=Arfgap3 PE=1 SV=2 | Arfgap3 | 1.02 |
| P34022 | Ran-specific GTPase-activating protein OS=Mus musculus OX=10090 GN=Ranbp1 PE=1 SV=2 | Ranbp1 | 1.02 |
| Q01768 | Nucleoside diphosphate kinase B OS=Mus musculus OX=10090 GN=Nme2 PE=1 SV=1 | Nme2 | 1.02 |
| P55012 | Solute carrier family 12 member 2 OS=Mus musculus OX=10090 GN=Slc12a2 PE=1 SV=2 | Slc12a2 | 1.02 |
| A3KGB4 | TBC1 domain family member 8B OS=Mus musculus OX=10090 GN=Tbc1d8b PE=1 SV=1 | Tbc1d8b | 1.02 |
| P53026 | 60S ribosomal protein L10a OS=Mus musculus OX=10090 GN=Rpl10a PE=1 SV=3 | Rpl10a | 1.02 |
| P23506 | Protein-L-isoaspartate(D-aspartate) O-methyltransferase OS=Mus musculus OX=10090 GN=Pcmt1 PE=1 SV=3 | Pcmt1 | 1.02 |
| Q9R0U0 | Serine/arginine-rich splicing factor 10 OS=Mus musculus OX=10090 GN=Srsf10 PE=1 SV=2 | Srsf10 | 1.02 |
| Q6PDG5 | SWI/SNF complex subunit SMARCC2 OS=Mus musculus OX=10090 GN=Smarcc2 PE=1 SV=2 | Smarcc2 | 1.02 |
| P05201 | Aspartate aminotransferase, cytoplasmic OS=Mus musculus OX=10090 GN=Got1 PE=1 SV=3 | Got1 | 1.02 |
| Q9Z204 | Heterogeneous nuclear ribonucleoproteins C1/C2 OS=Mus musculus OX=10090 GN=Hnrnpc PE=1 SV=1 | Hnrnpc | 1.02 |
| Q6P4T2 | U5 small nuclear ribonucleoprotein 200 kDa helicase OS=Mus musculus OX=10090 GN=Snrnp200 PE=1 SV=1 | Snrnp200 | 1.02 |
| P42867 | UDP-N-acetylglucosamine--dolichyl-phosphate N-acetylglucosaminephosphotransferase OS=Mus musculus OX=10090 GN=Dpagt1 PE=1 SV=2 | Dpagt1 | 1.02 |
| P42337 | Phosphatidylinositol 4,5-bisphosphate 3-kinase catalytic subunit alpha isoform OS=Mus musculus OX=10090 GN=Pik3ca PE=1 SV=2 | Pik3ca | 1.02 |
| Q0GNC1 | Inverted formin-2 OS=Mus musculus OX=10090 GN=Inf2 PE=1 SV=1 | Inf2 | 1.02 |
| Q09143 | High affinity cationic amino acid transporter 1 OS=Mus musculus OX=10090 GN=Slc7a1 PE=1 SV=1 | Slc7a1 | 1.02 |
| E9Q8T7 | Dynein axonemal heavy chain 1 OS=Mus musculus OX=10090 GN=Dnah1 PE=1 SV=1 | Dnah1 | 1.02 |
| Q6NZF1 | Zinc finger CCCH domain-containing protein 11A OS=Mus musculus OX=10090 GN=Zc3h11a PE=1 SV=1 | Zc3h11a | 1.02 |
| Q64511 | DNA topoisomerase 2-beta OS=Mus musculus OX=10090 GN=Top2b PE=1 SV=2 | Top2b | 1.02 |
| Q921Y4 | Molybdate-anion transporter OS=Mus musculus OX=10090 GN=Mfsd5 PE=2 SV=1 | Mfsd5 | 1.02 |
| Q8K2Y7 | 39S ribosomal protein L47, mitochondrial OS=Mus musculus OX=10090 GN=Mrpl47 PE=1 SV=2 | Mrpl47 | 1.02 |
| P20060 | Beta-hexosaminidase subunit beta OS=Mus musculus OX=10090 GN=Hexb PE=1 SV=2 | Hexb | 1.02 |
| Q9JJ89 | Coiled-coil domain-containing protein 86 OS=Mus musculus OX=10090 GN=Ccdc86 PE=1 SV=2 | Ccdc86 | 1.02 |
| Q8R3D1 | TBC1 domain family member 13 OS=Mus musculus OX=10090 GN=Tbc1d13 PE=1 SV=1 | Tbc1d13 | 1.02 |
| Q61249 | Immunoglobulin-binding protein 1 OS=Mus musculus OX=10090 GN=Igbp1 PE=1 SV=1 | Igbp1 | 1.02 |
| P18242 | Cathepsin D OS=Mus musculus OX=10090 GN=Ctsd PE=1 SV=1 | Ctsd | 1.02 |
| P60335 | Poly(rC)-binding protein 1 OS=Mus musculus OX=10090 GN=Pcbp1 PE=1 SV=1 | Pcbp1 | 1.02 |
| P62918 | 60S ribosomal protein L8 OS=Mus musculus OX=10090 GN=Rpl8 PE=1 SV=2 | Rpl8 | 1.02 |
| Q9D0F1 | Kinetochore protein NDC80 homolog OS=Mus musculus OX=10090 GN=Ndc80 PE=1 SV=1 | Ndc80 | 1.02 |
| P70429 | Ena/VASP-like protein OS=Mus musculus OX=10090 GN=Evl PE=1 SV=2 | Evl | 1.02 |
| P61022 | Calcineurin B homologous protein 1 OS=Mus musculus OX=10090 GN=Chp1 PE=1 SV=2 | Chp1 | 1.02 |
| Q6PCP5 | Mitochondrial fission factor OS=Mus musculus OX=10090 GN=Mff PE=1 SV=1 | Mff | 1.02 |
| Q6PGH1 | Protein BUD31 homolog OS=Mus musculus OX=10090 GN=Bud31 PE=1 SV=2 | Bud31 | 1.02 |
| Q9DCV4 | Regulator of microtubule dynamics protein 1 OS=Mus musculus OX=10090 GN=Rmdn1 PE=1 SV=2 | Rmdn1 | 1.02 |
| Q8VDD5 | Myosin-9 OS=Mus musculus OX=10090 GN=Myh9 PE=1 SV=4 | Myh9 | 1.02 |
| Q78PY7 | Staphylococcal nuclease domain-containing protein 1 OS=Mus musculus OX=10090 GN=Snd1 PE=1 SV=1 | Snd1 | 1.02 |
| Q07832 | Serine/threonine-protein kinase PLK1 OS=Mus musculus OX=10090 GN=Plk1 PE=1 SV=2 | Plk1 | 1.02 |
| Q9D2R0 | Acetoacetyl-CoA synthetase OS=Mus musculus OX=10090 GN=Aacs PE=1 SV=1 | Aacs | 1.02 |
| P62311 | U6 snRNA-associated Sm-like protein LSm3 OS=Mus musculus OX=10090 GN=Lsm3 PE=1 SV=2 | Lsm3 | 1.02 |
| P63094 | Guanine nucleotide-binding protein G(s) subunit alpha isoforms short OS=Mus musculus OX=10090 GN=Gnas PE=1 SV=1 | Gnas | 1.02 |
| Q9D8W5 | 26S proteasome non-ATPase regulatory subunit 12 OS=Mus musculus OX=10090 GN=Psmd12 PE=1 SV=4 | Psmd12 | 1.02 |
| Q6ZQ93 | Ubiquitin carboxyl-terminal hydrolase 34 OS=Mus musculus OX=10090 GN=Usp34 PE=1 SV=3 | Usp34 | 1.02 |
| Q8BFQ8 | Glutamine amidotransferase-like class 1 domain-containing protein 1 OS=Mus musculus OX=10090 GN=Gatd1 PE=1 SV=1 | Gatd1 | 1.02 |
| P31938 | Dual specificity mitogen-activated protein kinase kinase 1 OS=Mus musculus OX=10090 GN=Map2k1 PE=1 SV=2 | Map2k1 | 1.02 |
| Q6A0A9 | Constitutive coactivator of PPAR-gamma-like protein 1 OS=Mus musculus OX=10090 GN=FAM120A PE=1 SV=2 | FAM120A | 1.02 |
| P62915 | Transcription initiation factor IIB OS=Mus musculus OX=10090 GN=Gtf2b PE=1 SV=1 | Gtf2b | 1.02 |
| Q9D937 | Uncharacterized protein C11orf98 homolog OS=Mus musculus OX=10090 PE=1 SV=1 | -- | 1.02 |
| P97493 | Thioredoxin, mitochondrial OS=Mus musculus OX=10090 GN=Txn2 PE=1 SV=1 | Txn2 | 1.02 |
| P42128 | Forkhead box protein K1 OS=Mus musculus OX=10090 GN=Foxk1 PE=1 SV=2 | Foxk1 | 1.02 |
| O08605 | MAP kinase-interacting serine/threonine-protein kinase 1 OS=Mus musculus OX=10090 GN=Mknk1 PE=1 SV=2 | Mknk1 | 1.02 |
| Q6PB66 | Leucine-rich PPR motif-containing protein, mitochondrial OS=Mus musculus OX=10090 GN=Lrpprc PE=1 SV=2 | Lrpprc | 1.02 |
| O55125 | Protein NipSnap homolog 1 OS=Mus musculus OX=10090 GN=Nipsnap1 PE=1 SV=1 | Nipsnap1 | 1.02 |
| Q8VBV3 | Exosome complex component RRP4 OS=Mus musculus OX=10090 GN=Exosc2 PE=1 SV=1 | Exosc2 | 1.02 |
| Q7TSI3 | Serine/threonine-protein phosphatase 6 regulatory subunit 1 OS=Mus musculus OX=10090 GN=Ppp6r1 PE=1 SV=1 | Ppp6r1 | 1.02 |
| P84091 | AP-2 complex subunit mu OS=Mus musculus OX=10090 GN=Ap2m1 PE=1 SV=1 | Ap2m1 | 1.02 |
| A2RSY6 | TRMT1-like protein OS=Mus musculus OX=10090 GN=Trmt1l PE=1 SV=1 | Trmt1l | 1.02 |
| Q8BY35 | FYVE, RhoGEF and PH domain-containing protein 2 OS=Mus musculus OX=10090 GN=Fgd2 PE=1 SV=1 | Fgd2 | 1.02 |
| O88851 | Putative hydrolase RBBP9 OS=Mus musculus OX=10090 GN=Rbbp9 PE=1 SV=2 | Rbbp9 | 1.02 |
| Q8R242 | Di-N-acetylchitobiase OS=Mus musculus OX=10090 GN=Ctbs PE=1 SV=2 | Ctbs | 1.02 |
| Q810A7 | ATP-dependent RNA helicase DDX42 OS=Mus musculus OX=10090 GN=Ddx42 PE=1 SV=3 | Ddx42 | 1.02 |
| Q9DCZ1 | GMP reductase 1 OS=Mus musculus OX=10090 GN=Gmpr PE=1 SV=1 | Gmpr | 1.02 |
| Q3U308 | Cytoplasmic tRNA 2-thiolation protein 2 OS=Mus musculus OX=10090 GN=Ctu2 PE=1 SV=1 | Ctu2 | 1.02 |
| Q9Z1Q9 | Valine--tRNA ligase OS=Mus musculus OX=10090 GN=Vars1 PE=1 SV=1 | Vars1 | 1.02 |
| P00493 | Hypoxanthine-guanine phosphoribosyltransferase OS=Mus musculus OX=10090 GN=Hprt1 PE=1 SV=3 | Hprt1 | 1.02 |
| Q9CYI4 | Putative RNA-binding protein Luc7-like 1 OS=Mus musculus OX=10090 GN=Luc7l PE=1 SV=2 | Luc7l | 1.02 |
| P58059 | 28S ribosomal protein S21, mitochondrial OS=Mus musculus OX=10090 GN=Mrps21 PE=1 SV=1 | Mrps21 | 1.02 |
| Q9D1M4 | Eukaryotic translation elongation factor 1 epsilon-1 OS=Mus musculus OX=10090 GN=Eef1e1 PE=1 SV=1 | Eef1e1 | 1.02 |
| P24288 | Branched-chain-amino-acid aminotransferase, cytosolic OS=Mus musculus OX=10090 GN=Bcat1 PE=1 SV=2 | Bcat1 | 1.02 |
| Q9R0H0 | Peroxisomal acyl-coenzyme A oxidase 1 OS=Mus musculus OX=10090 GN=Acox1 PE=1 SV=5 | Acox1 | 1.02 |
| Q8VEL2 | Myotubularin-related protein 14 OS=Mus musculus OX=10090 GN=Mtmr14 PE=1 SV=2 | Mtmr14 | 1.02 |
| Q8QZY9 | Splicing factor 3B subunit 4 OS=Mus musculus OX=10090 GN=Sf3b4 PE=1 SV=1 | Sf3b4 | 1.02 |
| Q8VEM8 | Phosphate carrier protein, mitochondrial OS=Mus musculus OX=10090 GN=Slc25a3 PE=1 SV=1 | Slc25a3 | 1.02 |
| Q9Z0N2 | Eukaryotic translation initiation factor 2 subunit 3, Y-linked OS=Mus musculus OX=10090 GN=Eif2s3y PE=1 SV=2 | Eif2s3y | 1.02 |
| Q9JLJ5 | Elongation of very long chain fatty acids protein 1 OS=Mus musculus OX=10090 GN=Elovl1 PE=1 SV=1 | Elovl1 | 1.02 |
| Q99ME2 | WD repeat-containing protein 6 OS=Mus musculus OX=10090 GN=Wdr6 PE=1 SV=1 | Wdr6 | 1.02 |
| P42208 | Septin-2 OS=Mus musculus OX=10090 GN=Septin2 PE=1 SV=2 | Septin2 | 1.02 |
| Q3UYV9 | Nuclear cap-binding protein subunit 1 OS=Mus musculus OX=10090 GN=Ncbp1 PE=1 SV=2 | Ncbp1 | 1.02 |
| Q6ZQ03 | Formin-binding protein 4 OS=Mus musculus OX=10090 GN=Fnbp4 PE=1 SV=2 | Fnbp4 | 1.02 |
| Q91WQ3 | Tyrosine--tRNA ligase, cytoplasmic OS=Mus musculus OX=10090 GN=Yars1 PE=1 SV=3 | Yars1 | 1.02 |
| Q91YI0 | Argininosuccinate lyase OS=Mus musculus OX=10090 GN=Asl PE=1 SV=1 | Asl | 1.02 |
| O35215 | D-dopachrome decarboxylase OS=Mus musculus OX=10090 GN=Ddt PE=1 SV=3 | Ddt | 1.02 |
| Q00422 | GA-binding protein alpha chain OS=Mus musculus OX=10090 GN=Gabpa PE=1 SV=2 | Gabpa | 1.02 |
| Q9DCL8 | Protein phosphatase inhibitor 2 OS=Mus musculus OX=10090 GN=Ppp1r2 PE=1 SV=3 | Ppp1r2 | 1.02 |
| Q61216 | Double-strand break repair protein MRE11 OS=Mus musculus OX=10090 GN=Mre11 PE=1 SV=1 | Mre11 | 1.02 |
| Q8BX70 | Vacuolar protein sorting-associated protein 13C OS=Mus musculus OX=10090 GN=Vps13c PE=1 SV=2 | Vps13c | 1.02 |
| P46471 | 26S proteasome regulatory subunit 7 OS=Mus musculus OX=10090 GN=Psmc2 PE=1 SV=5 | Psmc2 | 1.02 |
| Q8BFV2 | PCI domain-containing protein 2 OS=Mus musculus OX=10090 GN=Pcid2 PE=1 SV=1 | Pcid2 | 1.02 |
| Q9WV55 | Vesicle-associated membrane protein-associated protein A OS=Mus musculus OX=10090 GN=Vapa PE=1 SV=2 | Vapa | 1.02 |
| Q8CFJ7 | Solute carrier family 25 member 45 OS=Mus musculus OX=10090 GN=Slc25a45 PE=1 SV=1 | Slc25a45 | 1.02 |
| Q9D023 | Mitochondrial pyruvate carrier 2 OS=Mus musculus OX=10090 GN=Mpc2 PE=1 SV=1 | Mpc2 | 1.02 |
| P10107 | Annexin A1 OS=Mus musculus OX=10090 GN=Anxa1 PE=1 SV=2 | Anxa1 | 1.02 |
| Q8BG07 | 5'-3' exonuclease PLD4 OS=Mus musculus OX=10090 GN=Pld4 PE=1 SV=1 | Pld4 | 1.02 |
| Q9CQU3 | Protein RER1 OS=Mus musculus OX=10090 GN=Rer1 PE=1 SV=1 | Rer1 | 1.02 |
| Q80U78 | Pumilio homolog 1 OS=Mus musculus OX=10090 GN=Pum1 PE=1 SV=2 | Pum1 | 1.02 |
| Q8C3Y4 | Kinetochore-associated protein 1 OS=Mus musculus OX=10090 GN=Kntc1 PE=1 SV=2 | Kntc1 | 1.02 |
| Q8BGY7 | Protein FAM210A OS=Mus musculus OX=10090 GN=Fam210a PE=1 SV=1 | Fam210a | 1.02 |
| Q3UDE2 | Tubulin--tyrosine ligase-like protein 12 OS=Mus musculus OX=10090 GN=Ttll12 PE=1 SV=1 | Ttll12 | 1.02 |
| Q56A08 | G-patch domain and KOW motifs-containing protein OS=Mus musculus OX=10090 GN=Gpkow PE=1 SV=2 | Gpkow | 1.03 |
| Q9WUA2 | Phenylalanine--tRNA ligase beta subunit OS=Mus musculus OX=10090 GN=Farsb PE=1 SV=2 | Farsb | 1.03 |
| B2RQC6 | CAD protein OS=Mus musculus OX=10090 GN=Cad PE=1 SV=1 | Cad | 1.03 |
| Q8K1X4 | Nck-associated protein 1-like OS=Mus musculus OX=10090 GN=Nckap1l PE=1 SV=1 | Nckap1l | 1.03 |
| P18653 | Ribosomal protein S6 kinase alpha-1 OS=Mus musculus OX=10090 GN=Rps6ka1 PE=1 SV=1 | Rps6ka1 | 1.03 |
| P60843 | Eukaryotic initiation factor 4A-I OS=Mus musculus OX=10090 GN=Eif4a1 PE=1 SV=1 | Eif4a1 | 1.03 |
| P09528 | Ferritin heavy chain OS=Mus musculus OX=10090 GN=Fth1 PE=1 SV=2 | Fth1 | 1.03 |
| Q9QYE6 | Golgin subfamily A member 5 OS=Mus musculus OX=10090 GN=Golga5 PE=1 SV=2 | Golga5 | 1.03 |
| Q99KQ4 | Nicotinamide phosphoribosyltransferase OS=Mus musculus OX=10090 GN=Nampt PE=1 SV=1 | Nampt | 1.03 |
| O88441 | Metaxin-2 OS=Mus musculus OX=10090 GN=Mtx2 PE=1 SV=1 | Mtx2 | 1.03 |
| Q922H1 | Protein arginine N-methyltransferase 3 OS=Mus musculus OX=10090 GN=Prmt3 PE=1 SV=2 | Prmt3 | 1.03 |
| Q924S8 | Sprouty-related, EVH1 domain-containing protein 1 OS=Mus musculus OX=10090 GN=Spred1 PE=1 SV=1 | Spred1 | 1.03 |
| O09110 | Dual specificity mitogen-activated protein kinase kinase 3 OS=Mus musculus OX=10090 GN=Map2k3 PE=1 SV=2 | Map2k3 | 1.03 |
| Q68FL4 | Putative adenosylhomocysteinase 3 OS=Mus musculus OX=10090 GN=Ahcyl2 PE=1 SV=1 | Ahcyl2 | 1.03 |
| P25322 | G1/S-specific cyclin-D1 OS=Mus musculus OX=10090 GN=Ccnd1 PE=1 SV=1 | Ccnd1 | 1.03 |
| Q91V12 | Cytosolic acyl coenzyme A thioester hydrolase OS=Mus musculus OX=10090 GN=Acot7 PE=1 SV=2 | Acot7 | 1.03 |
| Q8K1J6 | CCA tRNA nucleotidyltransferase 1, mitochondrial OS=Mus musculus OX=10090 GN=Trnt1 PE=1 SV=1 | Trnt1 | 1.03 |
| O88939 | Zinc finger and BTB domain-containing protein 7A OS=Mus musculus OX=10090 GN=Zbtb7a PE=1 SV=2 | Zbtb7a | 1.03 |
| P50431 | Serine hydroxymethyltransferase, cytosolic OS=Mus musculus OX=10090 GN=Shmt1 PE=1 SV=3 | Shmt1 | 1.03 |
| Q99K51 | Plastin-3 OS=Mus musculus OX=10090 GN=Pls3 PE=1 SV=3 | Pls3 | 1.03 |
| Q9CZ82 | Mediator of RNA polymerase II transcription subunit 18 OS=Mus musculus OX=10090 GN=Med18 PE=1 SV=1 | Med18 | 1.03 |
| Q9CQI3 | Glia maturation factor beta OS=Mus musculus OX=10090 GN=Gmfb PE=1 SV=3 | Gmfb | 1.03 |
| Q91WG8 | Bifunctional UDP-N-acetylglucosamine 2-epimerase/N-acetylmannosamine kinase OS=Mus musculus OX=10090 GN=Gne PE=1 SV=1 | Gne | 1.03 |
| Q6PGC1 | ATP-dependent RNA helicase DHX29 OS=Mus musculus OX=10090 GN=Dhx29 PE=1 SV=1 | Dhx29 | 1.03 |
| Q8BFY9 | Transportin-1 OS=Mus musculus OX=10090 GN=Tnpo1 PE=1 SV=2 | Tnpo1 | 1.03 |
| Q61768 | Kinesin-1 heavy chain OS=Mus musculus OX=10090 GN=Kif5b PE=1 SV=3 | Kif5b | 1.03 |
| Q9D8Y8 | Inhibitor of growth protein 5 OS=Mus musculus OX=10090 GN=Ing5 PE=1 SV=1 | Ing5 | 1.03 |
| Q9CPZ8 | COX assembly mitochondrial protein homolog OS=Mus musculus OX=10090 GN=Cmc1 PE=1 SV=1 | Cmc1 | 1.03 |
| O70133 | ATP-dependent RNA helicase A OS=Mus musculus OX=10090 GN=Dhx9 PE=1 SV=2 | Dhx9 | 1.03 |
| Q3U1Y4 | DENN domain-containing protein 4B OS=Mus musculus OX=10090 GN=Dennd4b PE=1 SV=2 | Dennd4b | 1.03 |
| P18654 | Ribosomal protein S6 kinase alpha-3 OS=Mus musculus OX=10090 GN=Rps6ka3 PE=1 SV=2 | Rps6ka3 | 1.03 |
| Q8C079 | Striatin-interacting protein 1 OS=Mus musculus OX=10090 GN=Strip1 PE=1 SV=2 | Strip1 | 1.03 |
| E9Q4N7 | AT-rich interactive domain-containing protein 1B OS=Mus musculus OX=10090 GN=Arid1b PE=1 SV=1 | Arid1b | 1.03 |
| Q9QYL7 | Activator of basal transcription 1 OS=Mus musculus OX=10090 GN=Abt1 PE=2 SV=1 | Abt1 | 1.03 |
| Q99LC3 | NADH dehydrogenase [ubiquinone] 1 alpha subcomplex subunit 10, mitochondrial OS=Mus musculus OX=10090 GN=Ndufa10 PE=1 SV=1 | Ndufa10 | 1.03 |
| Q9CQQ7 | ATP synthase F(0) complex subunit B1, mitochondrial OS=Mus musculus OX=10090 GN=Atp5pb PE=1 SV=1 | Atp5pb | 1.03 |
| A2AR02 | Peptidyl-prolyl cis-trans isomerase G OS=Mus musculus OX=10090 GN=Ppig PE=1 SV=1 | Ppig | 1.03 |
| Q6A068 | Cell division cycle 5-like protein OS=Mus musculus OX=10090 GN=Cdc5l PE=1 SV=2 | Cdc5l | 1.03 |
| Q60597 | 2-oxoglutarate dehydrogenase, mitochondrial OS=Mus musculus OX=10090 GN=Ogdh PE=1 SV=3 | Ogdh | 1.03 |
| Q8CIE6 | Coatomer subunit alpha OS=Mus musculus OX=10090 GN=Copa PE=1 SV=2 | Copa | 1.03 |
| P17439 | Lysosomal acid glucosylceramidase OS=Mus musculus OX=10090 GN=Gba PE=1 SV=1 | Gba | 1.03 |
| Q91V01 | Lysophospholipid acyltransferase 5 OS=Mus musculus OX=10090 GN=Lpcat3 PE=1 SV=1 | Lpcat3 | 1.03 |
| Q8K183 | Pyridoxal kinase OS=Mus musculus OX=10090 GN=Pdxk PE=1 SV=1 | Pdxk | 1.03 |
| Q91YP2 | Neurolysin, mitochondrial OS=Mus musculus OX=10090 GN=Nln PE=1 SV=1 | Nln | 1.03 |
| Q9JM90 | Signal-transducing adaptor protein 1 OS=Mus musculus OX=10090 GN=Stap1 PE=1 SV=1 | Stap1 | 1.03 |
| Q9Z2G0 | Protein fem-1 homolog B OS=Mus musculus OX=10090 GN=Fem1b PE=1 SV=1 | Fem1b | 1.03 |
| Q9D0R2 | Threonine--tRNA ligase 1, cytoplasmic OS=Mus musculus OX=10090 GN=Tars1 PE=1 SV=2 | Tars1 | 1.03 |
| P99029 | Peroxiredoxin-5, mitochondrial OS=Mus musculus OX=10090 GN=Prdx5 PE=1 SV=2 | Prdx5 | 1.03 |
| Q9WUB0 | RanBP-type and C3HC4-type zinc finger-containing protein 1 OS=Mus musculus OX=10090 GN=Rbck1 PE=1 SV=2 | Rbck1 | 1.03 |
| Q9JKF1 | Ras GTPase-activating-like protein IQGAP1 OS=Mus musculus OX=10090 GN=Iqgap1 PE=1 SV=2 | Iqgap1 | 1.03 |
| Q6PE01 | U5 small nuclear ribonucleoprotein 40 kDa protein OS=Mus musculus OX=10090 GN=Snrnp40 PE=1 SV=1 | Snrnp40 | 1.03 |
| Q60972 | Histone-binding protein RBBP4 OS=Mus musculus OX=10090 GN=Rbbp4 PE=1 SV=5 | Rbbp4 | 1.03 |
| Q9R0N0 | Galactokinase OS=Mus musculus OX=10090 GN=Galk1 PE=1 SV=2 | Galk1 | 1.03 |
| Q8R3Q6 | Protein MIX23 OS=Mus musculus OX=10090 GN=Mix23 PE=1 SV=1 | Mix23 | 1.03 |
| Q6P9Q6 | FK506-binding protein 15 OS=Mus musculus OX=10090 GN=Fkbp15 PE=1 SV=2 | Fkbp15 | 1.03 |
| P51569 | Alpha-galactosidase A OS=Mus musculus OX=10090 GN=Gla PE=1 SV=1 | Gla | 1.03 |
| Q3TUA9 | Protein O-mannose kinase OS=Mus musculus OX=10090 GN=Pomk PE=1 SV=2 | Pomk | 1.03 |
| Q9JIS8 | Solute carrier family 12 member 4 OS=Mus musculus OX=10090 GN=Slc12a4 PE=1 SV=2 | Slc12a4 | 1.03 |
| Q7TNP2 | Serine/threonine-protein phosphatase 2A 65 kDa regulatory subunit A beta isoform OS=Mus musculus OX=10090 GN=Ppp2r1b PE=1 SV=2 | Ppp2r1b | 1.03 |
| Q61187 | Tumor susceptibility gene 101 protein OS=Mus musculus OX=10090 GN=Tsg101 PE=1 SV=2 | Tsg101 | 1.03 |
| O70338 | Ribonuclease H1 OS=Mus musculus OX=10090 GN=Rnaseh1 PE=2 SV=1 | Rnaseh1 | 1.03 |
| Q8BYY4 | Tetratricopeptide repeat protein 39B OS=Mus musculus OX=10090 GN=Ttc39b PE=1 SV=1 | Ttc39b | 1.03 |
| Q9CWW6 | Peptidyl-prolyl cis-trans isomerase NIMA-interacting 4 OS=Mus musculus OX=10090 GN=Pin4 PE=1 SV=1 | Pin4 | 1.03 |
| Q9DBS2 | Tumor protein p63-regulated gene 1-like protein OS=Mus musculus OX=10090 GN=Tprg1l PE=1 SV=1 | Tprg1l | 1.03 |
| O54940 | BCL2/adenovirus E1B 19 kDa protein-interacting protein 2 OS=Mus musculus OX=10090 GN=Bnip2 PE=1 SV=2 | Bnip2 | 1.03 |
| Q03141 | MAP/microtubule affinity-regulating kinase 3 OS=Mus musculus OX=10090 GN=Mark3 PE=1 SV=2 | Mark3 | 1.03 |
| P26638 | Serine--tRNA ligase, cytoplasmic OS=Mus musculus OX=10090 GN=Sars1 PE=1 SV=3 | Sars1 | 1.03 |
| P56183 | Ribosomal RNA processing protein 1 homolog A OS=Mus musculus OX=10090 GN=Rrp1 PE=1 SV=2 | Rrp1 | 1.03 |
| Q8BXA1 | Golgi integral membrane protein 4 OS=Mus musculus OX=10090 GN=Golim4 PE=1 SV=1 | Golim4 | 1.03 |
| P59708 | Splicing factor 3B subunit 6 OS=Mus musculus OX=10090 GN=Sf3b6 PE=1 SV=1 | Sf3b6 | 1.03 |
| P70318 | Nucleolysin TIAR OS=Mus musculus OX=10090 GN=Tial1 PE=1 SV=1 | Tial1 | 1.03 |
| Q3TRM8 | Hexokinase-3 OS=Mus musculus OX=10090 GN=Hk3 PE=1 SV=2 | Hk3 | 1.03 |
| Q64FW2 | All-trans-retinol 13,14-reductase OS=Mus musculus OX=10090 GN=Retsat PE=1 SV=3 | Retsat | 1.03 |
| Q9D3D0 | Alpha-tocopherol transfer protein-like OS=Mus musculus OX=10090 GN=Ttpal PE=1 SV=3 | Ttpal | 1.03 |
| Q63850 | Nuclear pore glycoprotein p62 OS=Mus musculus OX=10090 GN=Nup62 PE=1 SV=2 | Nup62 | 1.03 |
| P50247 | Adenosylhomocysteinase OS=Mus musculus OX=10090 GN=Ahcy PE=1 SV=3 | Ahcy | 1.03 |
| Q8BGC0 | HIV Tat-specific factor 1 homolog OS=Mus musculus OX=10090 GN=Htatsf1 PE=1 SV=1 | Htatsf1 | 1.03 |
| Q8K3C3 | Protein LZIC OS=Mus musculus OX=10090 GN=Lzic PE=1 SV=1 | Lzic | 1.03 |
| Q9CQ28 | Diphthine--ammonia ligase OS=Mus musculus OX=10090 GN=Dph6 PE=1 SV=1 | Dph6 | 1.03 |
| Q78JE5 | F-box only protein 22 OS=Mus musculus OX=10090 GN=Fbxo22 PE=1 SV=2 | Fbxo22 | 1.03 |
| Q9CXF4 | TBC1 domain family member 15 OS=Mus musculus OX=10090 GN=Tbc1d15 PE=1 SV=1 | Tbc1d15 | 1.03 |
| Q8BGD6 | Sodium-coupled neutral amino acid transporter 9 OS=Mus musculus OX=10090 GN=Slc38a9 PE=1 SV=1 | Slc38a9 | 1.03 |
| Q9CQF6 | L-aminoadipate-semialdehyde dehydrogenase-phosphopantetheinyl transferase OS=Mus musculus OX=10090 GN=Aasdhppt PE=1 SV=1 | Aasdhppt | 1.03 |
| Q9EPK2 | Protein XRP2 OS=Mus musculus OX=10090 GN=Rp2 PE=1 SV=3 | Rp2 | 1.03 |
| Q6P3Y5 | Zinc finger protein 280C OS=Mus musculus OX=10090 GN=Znf280c PE=1 SV=1 | Znf280c | 1.03 |
| Q8BVE8 | Histone-lysine N-methyltransferase NSD2 OS=Mus musculus OX=10090 GN=Nsd2 PE=1 SV=2 | Nsd2 | 1.03 |
| O08740 | DNA-directed RNA polymerase II subunit RPB11 OS=Mus musculus OX=10090 GN=Polr2j PE=1 SV=1 | Polr2j | 1.03 |
| Q9Z2V6 | Histone deacetylase 5 OS=Mus musculus OX=10090 GN=Hdac5 PE=1 SV=2 | Hdac5 | 1.03 |
| Q2L4X1 | Basic leucine zipper and W2 domain-containing protein 2 OS=Mus musculus molossinus OX=57486 GN=Bzw2 PE=2 SV=1 | Bzw2 | 1.03 |
| Q8BUM3 | Tyrosine-protein phosphatase non-receptor type 7 OS=Mus musculus OX=10090 GN=Ptpn7 PE=1 SV=1 | Ptpn7 | 1.03 |
| Q65Z40 | Wings apart-like protein homolog OS=Mus musculus OX=10090 GN=Wapl PE=1 SV=2 | Wapl | 1.03 |
| Q922Q1 | Mitochondrial amidoxime reducing component 2 OS=Mus musculus OX=10090 GN=Mtarc2 PE=1 SV=1 | Mtarc2 | 1.03 |
| Q9CR57 | 60S ribosomal protein L14 OS=Mus musculus OX=10090 GN=Rpl14 PE=1 SV=3 | Rpl14 | 1.03 |
| Q9WTL7 | Acyl-protein thioesterase 2 OS=Mus musculus OX=10090 GN=Lypla2 PE=1 SV=1 | Lypla2 | 1.03 |
| Q3UIR3 | E3 ubiquitin-protein ligase DTX3L OS=Mus musculus OX=10090 GN=Dtx3l PE=1 SV=1 | Dtx3l | 1.03 |
| Q922B2 | Aspartate--tRNA ligase, cytoplasmic OS=Mus musculus OX=10090 GN=Dars1 PE=1 SV=2 | Dars1 | 1.03 |
| P15307 | Proto-oncogene c-Rel OS=Mus musculus OX=10090 GN=Rel PE=1 SV=2 | Rel | 1.03 |
| Q8BNV1 | tRNA (uracil-5-)-methyltransferase homolog A OS=Mus musculus OX=10090 GN=Trmt2a PE=1 SV=1 | Trmt2a | 1.03 |
| Q09014 | Neutrophil cytosol factor 1 OS=Mus musculus OX=10090 GN=Ncf1 PE=1 SV=3 | Ncf1 | 1.03 |
| P62821 | Ras-related protein Rab-1A OS=Mus musculus OX=10090 GN=Rab1A PE=1 SV=3 | Rab1A | 1.03 |
| P57080 | Ubiquitin carboxyl-terminal hydrolase 25 OS=Mus musculus OX=10090 GN=Usp25 PE=1 SV=2 | Usp25 | 1.03 |
| Q9DC69 | NADH dehydrogenase [ubiquinone] 1 alpha subcomplex subunit 9, mitochondrial OS=Mus musculus OX=10090 GN=Ndufa9 PE=1 SV=2 | Ndufa9 | 1.03 |
| Q9WUP7 | Ubiquitin carboxyl-terminal hydrolase isozyme L5 OS=Mus musculus OX=10090 GN=Uchl5 PE=1 SV=2 | Uchl5 | 1.03 |
| Q8K009 | Mitochondrial 10-formyltetrahydrofolate dehydrogenase OS=Mus musculus OX=10090 GN=Aldh1l2 PE=1 SV=2 | Aldh1l2 | 1.03 |
| Q6AXC6 | ATP-dependent DNA helicase DDX11 OS=Mus musculus OX=10090 GN=Ddx11 PE=1 SV=2 | Ddx11 | 1.03 |
| Q99J45 | Nuclear receptor-binding protein OS=Mus musculus OX=10090 GN=Nrbp1 PE=1 SV=1 | Nrbp1 | 1.03 |
| Q9CVB6 | Actin-related protein 2/3 complex subunit 2 OS=Mus musculus OX=10090 GN=Arpc2 PE=1 SV=3 | Arpc2 | 1.03 |
| Q8BY02 | NF-kappa-B-repressing factor OS=Mus musculus OX=10090 GN=Nkrf PE=2 SV=3 | Nkrf | 1.03 |
| Q9JJ78 | Lymphokine-activated killer T-cell-originated protein kinase OS=Mus musculus OX=10090 GN=Pbk PE=1 SV=1 | Pbk | 1.03 |
| Q9EQC5 | N-terminal kinase-like protein OS=Mus musculus OX=10090 GN=Scyl1 PE=1 SV=1 | Scyl1 | 1.03 |
| E9Q4P1 | WD repeat and FYVE domain-containing protein 1 OS=Mus musculus OX=10090 GN=Wdfy1 PE=1 SV=1 | Wdfy1 | 1.03 |
| Q99JT9 | 1,2-dihydroxy-3-keto-5-methylthiopentene dioxygenase OS=Mus musculus OX=10090 GN=Adi1 PE=1 SV=1 | Adi1 | 1.03 |
| O89110 | Caspase-8 OS=Mus musculus OX=10090 GN=Casp8 PE=1 SV=1 | Casp8 | 1.03 |
| Q9WUH1 | Transmembrane protein 115 OS=Mus musculus OX=10090 GN=Tmem115 PE=1 SV=1 | Tmem115 | 1.03 |
| Q00612 | Glucose-6-phosphate 1-dehydrogenase X OS=Mus musculus OX=10090 GN=G6pdx PE=1 SV=3 | G6pdx | 1.03 |
| Q8BN21 | Serine/threonine-protein kinase VRK2 OS=Mus musculus OX=10090 GN=Vrk2 PE=1 SV=2 | Vrk2 | 1.03 |
| Q9CQ71 | Replication protein A 14 kDa subunit OS=Mus musculus OX=10090 GN=Rpa3 PE=1 SV=1 | Rpa3 | 1.03 |
| Q9CPR1 | RWD domain-containing protein 4 OS=Mus musculus OX=10090 GN=Rwdd4 PE=2 SV=1 | Rwdd4 | 1.04 |
| Q60605 | Myosin light polypeptide 6 OS=Mus musculus OX=10090 GN=Myl6 PE=1 SV=3 | Myl6 | 1.04 |
| Q8CIV2 | Membralin OS=Mus musculus OX=10090 GN=Tmem259 PE=1 SV=2 | Tmem259 | 1.04 |
| Q01730 | Ras suppressor protein 1 OS=Mus musculus OX=10090 GN=Rsu1 PE=1 SV=3 | Rsu1 | 1.04 |
| Q8R050 | Eukaryotic peptide chain release factor GTP-binding subunit ERF3A OS=Mus musculus OX=10090 GN=Gspt1 PE=1 SV=2 | Gspt1 | 1.04 |
| P42125 | Enoyl-CoA delta isomerase 1, mitochondrial OS=Mus musculus OX=10090 GN=Eci1 PE=1 SV=2 | Eci1 | 1.04 |
| Q99N96 | 39S ribosomal protein L1, mitochondrial OS=Mus musculus OX=10090 GN=Mrpl1 PE=1 SV=2 | Mrpl1 | 1.04 |
| P10518 | Delta-aminolevulinic acid dehydratase OS=Mus musculus OX=10090 GN=Alad PE=1 SV=1 | Alad | 1.04 |
| Q8JZM0 | Dimethyladenosine transferase 1, mitochondrial OS=Mus musculus OX=10090 GN=Tfb1m PE=1 SV=1 | Tfb1m | 1.04 |
| Q9ET26 | E3 ubiquitin-protein ligase RNF114 OS=Mus musculus OX=10090 GN=Rnf114 PE=1 SV=2 | Rnf114 | 1.04 |
| Q60770 | Syntaxin-binding protein 3 OS=Mus musculus OX=10090 GN=Stxbp3 PE=1 SV=1 | Stxbp3 | 1.04 |
| Q8R0F3 | Formylglycine-generating enzyme OS=Mus musculus OX=10090 GN=Sumf1 PE=1 SV=2 | Sumf1 | 1.04 |
| B9EJ86 | Oxysterol-binding protein-related protein 8 OS=Mus musculus OX=10090 GN=Osbpl8 PE=1 SV=1 | Osbpl8 | 1.04 |
| P54310 | Hormone-sensitive lipase OS=Mus musculus OX=10090 GN=Lipe PE=1 SV=2 | Lipe | 1.04 |
| Q8VCA8 | Secernin-2 OS=Mus musculus OX=10090 GN=Scrn2 PE=1 SV=1 | Scrn2 | 1.04 |
| Q9JKC8 | AP-3 complex subunit mu-1 OS=Mus musculus OX=10090 GN=Ap3m1 PE=1 SV=1 | Ap3m1 | 1.04 |
| Q7TNC4 | Putative RNA-binding protein Luc7-like 2 OS=Mus musculus OX=10090 GN=Luc7l2 PE=1 SV=1 | Luc7l2 | 1.04 |
| Q31125 | Zinc transporter SLC39A7 OS=Mus musculus OX=10090 GN=Slc39a7 PE=1 SV=2 | Slc39a7 | 1.04 |
| Q99MN1 | Lysine--tRNA ligase OS=Mus musculus OX=10090 GN=Kars1 PE=1 SV=1 | Kars1 | 1.04 |
| P16045 | Galectin-1 OS=Mus musculus OX=10090 GN=Lgals1 PE=1 SV=3 | Lgals1 | 1.04 |
| P61028 | Ras-related protein Rab-8B OS=Mus musculus OX=10090 GN=Rab8b PE=1 SV=1 | Rab8b | 1.04 |
| Q8VE18 | Protein SMG8 OS=Mus musculus OX=10090 GN=Smg8 PE=1 SV=1 | Smg8 | 1.04 |
| Q9Z1K5 | E3 ubiquitin-protein ligase ARIH1 OS=Mus musculus OX=10090 GN=Arih1 PE=1 SV=3 | Arih1 | 1.04 |
| Q61103 | Zinc finger protein ubi-d4 OS=Mus musculus OX=10090 GN=Dpf2 PE=1 SV=1 | Dpf2 | 1.04 |
| Q8VDG5 | Phosphopantothenate--cysteine ligase OS=Mus musculus OX=10090 GN=Ppcs PE=1 SV=1 | Ppcs | 1.04 |
| Q9QYI4 | DnaJ homolog subfamily B member 12 OS=Mus musculus OX=10090 GN=Dnajb12 PE=1 SV=2 | Dnajb12 | 1.04 |
| Q8K194 | U4/U6.U5 small nuclear ribonucleoprotein 27 kDa protein OS=Mus musculus OX=10090 GN=Snrnp27 PE=1 SV=1 | Snrnp27 | 1.04 |
| Q9JIB4 | General transcription factor IIH subunit 2 OS=Mus musculus OX=10090 GN=Gtf2h2 PE=1 SV=1 | Gtf2h2 | 1.04 |
| B1AZI6 | THO complex subunit 2 OS=Mus musculus OX=10090 GN=Thoc2 PE=1 SV=1 | Thoc2 | 1.04 |
| O70145 | Neutrophil cytosol factor 2 OS=Mus musculus OX=10090 GN=Ncf2 PE=1 SV=1 | Ncf2 | 1.04 |
| Q9EQH2 | Endoplasmic reticulum aminopeptidase 1 OS=Mus musculus OX=10090 GN=Erap1 PE=1 SV=2 | Erap1 | 1.04 |
| P48725 | Pericentrin OS=Mus musculus OX=10090 GN=Pcnt PE=1 SV=2 | Pcnt | 1.04 |
| Q7TMY8 | E3 ubiquitin-protein ligase HUWE1 OS=Mus musculus OX=10090 GN=Huwe1 PE=1 SV=5 | Huwe1 | 1.04 |
| Q9CQ80 | Vacuolar protein-sorting-associated protein 25 OS=Mus musculus OX=10090 GN=Vps25 PE=1 SV=1 | Vps25 | 1.04 |
| Q8C0L8 | Conserved oligomeric Golgi complex subunit 5 OS=Mus musculus OX=10090 GN=Cog5 PE=1 SV=3 | Cog5 | 1.04 |
| Q3UHA3 | Spatacsin OS=Mus musculus OX=10090 GN=Spg11 PE=1 SV=3 | Spg11 | 1.04 |
| O88848 | ADP-ribosylation factor-like protein 6 OS=Mus musculus OX=10090 GN=Arl6 PE=1 SV=1 | Arl6 | 1.04 |
| Q3UND0 | Src kinase-associated phosphoprotein 2 OS=Mus musculus OX=10090 GN=Skap2 PE=1 SV=2 | Skap2 | 1.04 |
| Q99KC8 | von Willebrand factor A domain-containing protein 5A OS=Mus musculus OX=10090 GN=Vwa5a PE=1 SV=2 | Vwa5a | 1.04 |
| Q8C6E0 | Cilia- and flagella-associated protein 36 OS=Mus musculus OX=10090 GN=Cfap36 PE=1 SV=1 | Cfap36 | 1.04 |
| Q8BIP0 | Aspartate--tRNA ligase, mitochondrial OS=Mus musculus OX=10090 GN=Dars2 PE=1 SV=1 | Dars2 | 1.04 |
| Q8C2K1 | Differentially expressed in FDCP 6 OS=Mus musculus OX=10090 GN=Def6 PE=1 SV=1 | Def6 | 1.04 |
| Q504P2 | C-type lectin domain family 12 member A OS=Mus musculus OX=10090 GN=Clec12a PE=2 SV=1 | Clec12a | 1.04 |
| Q9Z0X1 | Apoptosis-inducing factor 1, mitochondrial OS=Mus musculus OX=10090 GN=Aifm1 PE=1 SV=1 | Aifm1 | 1.04 |
| O54950 | 5'-AMP-activated protein kinase subunit gamma-1 OS=Mus musculus OX=10090 GN=Prkag1 PE=1 SV=2 | Prkag1 | 1.04 |
| Q6PDL0 | Cytoplasmic dynein 1 light intermediate chain 2 OS=Mus musculus OX=10090 GN=Dync1li2 PE=1 SV=2 | Dync1li2 | 1.04 |
| Q91YJ5 | Translation initiation factor IF-2, mitochondrial OS=Mus musculus OX=10090 GN=Mtif2 PE=1 SV=2 | Mtif2 | 1.04 |
| O09111 | NADH dehydrogenase [ubiquinone] 1 beta subcomplex subunit 11, mitochondrial OS=Mus musculus OX=10090 GN=Ndufb11 PE=1 SV=2 | Ndufb11 | 1.04 |
| Q61337 | Bcl2-associated agonist of cell death OS=Mus musculus OX=10090 GN=Bad PE=1 SV=1 | Bad | 1.04 |
| Q64516 | Glycerol kinase OS=Mus musculus OX=10090 GN=Gk PE=1 SV=2 | Gk | 1.04 |
| Q9CQF4 | Mitochondrial transcription rescue factor 1 OS=Mus musculus OX=10090 GN=Mtres1 PE=1 SV=1 | Mtres1 | 1.04 |
| Q61286 | Transcription factor 12 OS=Mus musculus OX=10090 GN=Tcf12 PE=1 SV=2 | Tcf12 | 1.04 |
| Q99N85 | 28S ribosomal protein S18a, mitochondrial OS=Mus musculus OX=10090 GN=Mrps18a PE=2 SV=1 | Mrps18a | 1.04 |
| P62862 | 40S ribosomal protein S30 OS=Mus musculus OX=10090 GN=Fau PE=1 SV=1 | Fau | 1.04 |
| Q9D8E6 | 60S ribosomal protein L4 OS=Mus musculus OX=10090 GN=Rpl4 PE=1 SV=3 | Rpl4 | 1.04 |
| P62627 | Dynein light chain roadblock-type 1 OS=Mus musculus OX=10090 GN=Dynlrb1 PE=1 SV=3 | Dynlrb1 | 1.04 |
| Q8CGA0 | Protein phosphatase 1F OS=Mus musculus OX=10090 GN=Ppm1f PE=1 SV=1 | Ppm1f | 1.04 |
| Q8C547 | HEAT repeat-containing protein 5B OS=Mus musculus OX=10090 GN=Heatr5b PE=1 SV=3 | Heatr5b | 1.04 |
| Q8BH59 | Calcium-binding mitochondrial carrier protein Aralar1 OS=Mus musculus OX=10090 GN=Slc25a12 PE=1 SV=1 | Slc25a12 | 1.04 |
| Q9ERE7 | LRP chaperone MESD OS=Mus musculus OX=10090 GN=Mesd PE=1 SV=1 | Mesd | 1.04 |
| Q9D0J8 | Parathymosin OS=Mus musculus OX=10090 GN=Ptms PE=1 SV=3 | Ptms | 1.04 |
| P11688 | Integrin alpha-5 OS=Mus musculus OX=10090 GN=Itga5 PE=1 SV=3 | Itga5 | 1.04 |
| Q61035 | Histidine--tRNA ligase, cytoplasmic OS=Mus musculus OX=10090 GN=Hars1 PE=1 SV=2 | Hars1 | 1.04 |
| Q8CIE4 | Protein mono-ADP-ribosyltransferase PARP10 OS=Mus musculus OX=10090 GN=Parp10 PE=2 SV=1 | Parp10 | 1.04 |
| Q4VA53 | Sister chromatid cohesion protein PDS5 homolog B OS=Mus musculus OX=10090 GN=Pds5b PE=1 SV=1 | Pds5b | 1.04 |
| Q00420 | GA-binding protein subunit beta-1 OS=Mus musculus OX=10090 GN=Gabpb1 PE=1 SV=2 | Gabpb1 | 1.04 |
| Q9QUR6 | Prolyl endopeptidase OS=Mus musculus OX=10090 GN=Prep PE=1 SV=1 | Prep | 1.04 |
| Q9WVQ5 | Methylthioribulose-1-phosphate dehydratase OS=Mus musculus OX=10090 GN=Apip PE=1 SV=1 | Apip | 1.04 |
| Q80UV9 | Transcription initiation factor TFIID subunit 1 OS=Mus musculus OX=10090 GN=Taf1 PE=1 SV=2 | Taf1 | 1.04 |
| Q99KR7 | Peptidyl-prolyl cis-trans isomerase F, mitochondrial OS=Mus musculus OX=10090 GN=Ppif PE=1 SV=1 | Ppif | 1.04 |
| Q3V1L4 | Cytosolic purine 5'-nucleotidase OS=Mus musculus OX=10090 GN=Nt5c2 PE=1 SV=2 | Nt5c2 | 1.04 |
| Q9CYF5 | RCC1-like G exchanging factor-like protein OS=Mus musculus OX=10090 GN=Rcc1l PE=1 SV=1 | Rcc1l | 1.04 |
| Q68FD5 | Clathrin heavy chain 1 OS=Mus musculus OX=10090 GN=Cltc PE=1 SV=3 | Cltc | 1.04 |
| Q60996 | Serine/threonine-protein phosphatase 2A 56 kDa regulatory subunit gamma isoform OS=Mus musculus OX=10090 GN=Ppp2r5c PE=1 SV=2 | Ppp2r5c | 1.04 |
| P60867 | 40S ribosomal protein S20 OS=Mus musculus OX=10090 GN=Rps20 PE=1 SV=1 | Rps20 | 1.04 |
| Q9JM62 | Receptor expression-enhancing protein 6 OS=Mus musculus OX=10090 GN=Reep6 PE=1 SV=1 | Reep6 | 1.04 |
| Q99M01 | Phenylalanine--tRNA ligase, mitochondrial OS=Mus musculus OX=10090 GN=Fars2 PE=1 SV=1 | Fars2 | 1.04 |
| Q9Z1Z2 | Serine-threonine kinase receptor-associated protein OS=Mus musculus OX=10090 GN=Strap PE=1 SV=2 | Strap | 1.04 |
| Q91WV0 | Protein Dr1 OS=Mus musculus OX=10090 GN=Dr1 PE=1 SV=1 | Dr1 | 1.04 |
| O55131 | Septin-7 OS=Mus musculus OX=10090 GN=Septin7 PE=1 SV=1 | Septin7 | 1.04 |
| Q3UM29 | Conserved oligomeric Golgi complex subunit 7 OS=Mus musculus OX=10090 GN=Cog7 PE=1 SV=1 | Cog7 | 1.04 |
| P60766 | Cell division control protein 42 homolog OS=Mus musculus OX=10090 GN=Cdc42 PE=1 SV=2 | Cdc42 | 1.04 |
| Q9CQF0 | 39S ribosomal protein L11, mitochondrial OS=Mus musculus OX=10090 GN=Mrpl11 PE=1 SV=1 | Mrpl11 | 1.04 |
| Q8K337 | Type II inositol 1,4,5-trisphosphate 5-phosphatase OS=Mus musculus OX=10090 GN=Inpp5b PE=1 SV=1 | Inpp5b | 1.04 |
| Q8CI51 | PDZ and LIM domain protein 5 OS=Mus musculus OX=10090 GN=Pdlim5 PE=1 SV=4 | Pdlim5 | 1.04 |
| Q9CQ86 | Migration and invasion enhancer 1 OS=Mus musculus OX=10090 GN=Mien1 PE=1 SV=1 | Mien1 | 1.04 |
| P47754 | F-actin-capping protein subunit alpha-2 OS=Mus musculus OX=10090 GN=Capza2 PE=1 SV=3 | Capza2 | 1.04 |
| O35887 | Calumenin OS=Mus musculus OX=10090 GN=Calu PE=1 SV=1 | Calu | 1.04 |
| Q99JY9 | Actin-related protein 3 OS=Mus musculus OX=10090 GN=Actr3 PE=1 SV=3 | Actr3 | 1.04 |
| Q60865 | Caprin-1 OS=Mus musculus OX=10090 GN=Caprin1 PE=1 SV=2 | Caprin1 | 1.04 |
| Q8C0S1 | DIS3-like exonuclease 1 OS=Mus musculus OX=10090 GN=Dis3l PE=1 SV=2 | Dis3l | 1.04 |
| Q3TIX9 | U4/U6.U5 tri-snRNP-associated protein 2 OS=Mus musculus OX=10090 GN=Usp39 PE=1 SV=2 | Usp39 | 1.04 |
| Q9CPW7 | Zinc finger matrin-type protein 2 OS=Mus musculus OX=10090 GN=Zmat2 PE=2 SV=1 | Zmat2 | 1.04 |
| Q8R3P6 | Integrator complex subunit 14 OS=Mus musculus OX=10090 GN=Ints14 PE=1 SV=1 | Ints14 | 1.04 |
| Q61470 | Leukocyte antigen CD37 OS=Mus musculus OX=10090 GN=Cd37 PE=1 SV=1 | Cd37 | 1.04 |
| Q3V1V3 | ESF1 homolog OS=Mus musculus OX=10090 GN=Esf1 PE=1 SV=1 | Esf1 | 1.04 |
| Q9D0D4 | Probable dimethyladenosine transferase OS=Mus musculus OX=10090 GN=Dimt1 PE=2 SV=1 | Dimt1 | 1.04 |
| Q64735 | Complement component receptor 1-like protein OS=Mus musculus OX=10090 GN=Cr1l PE=1 SV=1 | Cr1l | 1.04 |
| Q5DTM8 | E3 ubiquitin-protein ligase BRE1A OS=Mus musculus OX=10090 GN=Rnf20 PE=1 SV=2 | Rnf20 | 1.04 |
| O09167 | 60S ribosomal protein L21 OS=Mus musculus OX=10090 GN=Rpl21 PE=1 SV=3 | Rpl21 | 1.04 |
| Q8R2Y0 | Monoacylglycerol lipase ABHD6 OS=Mus musculus OX=10090 GN=Abhd6 PE=1 SV=1 | Abhd6 | 1.04 |
| Q8BVE3 | V-type proton ATPase subunit H OS=Mus musculus OX=10090 GN=Atp6v1h PE=1 SV=1 | Atp6v1h | 1.04 |
| Q501J6 | Probable ATP-dependent RNA helicase DDX17 OS=Mus musculus OX=10090 GN=Ddx17 PE=1 SV=1 | Ddx17 | 1.04 |
| Q8R5C5 | Beta-centractin OS=Mus musculus OX=10090 GN=Actr1b PE=1 SV=1 | Actr1b | 1.04 |
| P61164 | Alpha-centractin OS=Mus musculus OX=10090 GN=Actr1a PE=1 SV=1 | Actr1a | 1.04 |
| P97478 | 5-demethoxyubiquinone hydroxylase, mitochondrial OS=Mus musculus OX=10090 GN=Coq7 PE=1 SV=3 | Coq7 | 1.04 |
| Q9CWJ9 | Bifunctional purine biosynthesis protein ATIC OS=Mus musculus OX=10090 GN=Atic PE=1 SV=2 | Atic | 1.04 |
| P59325 | Eukaryotic translation initiation factor 5 OS=Mus musculus OX=10090 GN=Eif5 PE=1 SV=1 | Eif5 | 1.04 |
| P42567 | Epidermal growth factor receptor substrate 15 OS=Mus musculus OX=10090 GN=Eps15 PE=1 SV=1 | Eps15 | 1.04 |
| P49135 | General transcription and DNA repair factor IIH helicase subunit XPB OS=Mus musculus OX=10090 GN=Ercc3 PE=2 SV=1 | Ercc3 | 1.05 |
| Q9DBL1 | Short/branched chain specific acyl-CoA dehydrogenase, mitochondrial OS=Mus musculus OX=10090 GN=Acadsb PE=1 SV=1 | Acadsb | 1.05 |
| Q8C0P7 | E3 SUMO-protein ligase ZNF451 OS=Mus musculus OX=10090 GN=Znf451 PE=1 SV=1 | Znf451 | 1.05 |
| Q9CZU3 | Exosome RNA helicase MTR4 OS=Mus musculus OX=10090 GN=Mtrex PE=1 SV=1 | Mtrex | 1.05 |
| Q68FH4 | N-acetylgalactosamine kinase OS=Mus musculus OX=10090 GN=Galk2 PE=1 SV=1 | Galk2 | 1.05 |
| E9Q912 | Rap1 GTPase-GDP dissociation stimulator 1 OS=Mus musculus OX=10090 GN=Rap1gds1 PE=2 SV=1 | Rap1gds1 | 1.05 |
| P84096 | Rho-related GTP-binding protein RhoG OS=Mus musculus OX=10090 GN=Rhog PE=1 SV=1 | Rhog | 1.05 |
| P51912 | Neutral amino acid transporter B(0) OS=Mus musculus OX=10090 GN=Slc1a5 PE=1 SV=2 | Slc1a5 | 1.05 |
| Q60973 | Histone-binding protein RBBP7 OS=Mus musculus OX=10090 GN=Rbbp7 PE=1 SV=1 | Rbbp7 | 1.05 |
| Q9DBT5 | AMP deaminase 2 OS=Mus musculus OX=10090 GN=Ampd2 PE=1 SV=1 | Ampd2 | 1.05 |
| O08997 | Copper transport protein ATOX1 OS=Mus musculus OX=10090 GN=Atox1 PE=1 SV=1 | Atox1 | 1.05 |
| O35129 | Prohibitin-2 OS=Mus musculus OX=10090 GN=Phb2 PE=1 SV=1 | Phb2 | 1.05 |
| P09055 | Integrin beta-1 OS=Mus musculus OX=10090 GN=Itgb1 PE=1 SV=1 | Itgb1 | 1.05 |
| Q9ESX5 | H/ACA ribonucleoprotein complex subunit DKC1 OS=Mus musculus OX=10090 GN=Dkc1 PE=1 SV=4 | Dkc1 | 1.05 |
| P30993 | C5a anaphylatoxin chemotactic receptor 1 OS=Mus musculus OX=10090 GN=C5ar1 PE=1 SV=2 | C5ar1 | 1.05 |
| P59235 | Nucleoporin Nup43 OS=Mus musculus OX=10090 GN=Nup43 PE=1 SV=2 | Nup43 | 1.05 |
| Q8BWY3 | Eukaryotic peptide chain release factor subunit 1 OS=Mus musculus OX=10090 GN=Etf1 PE=1 SV=4 | Etf1 | 1.05 |
| P46656 | Adrenodoxin, mitochondrial OS=Mus musculus OX=10090 GN=Fdx1 PE=1 SV=1 | Fdx1 | 1.05 |
| Q9CS74 | Protein ecdysoneless homolog OS=Mus musculus OX=10090 GN=Ecd PE=1 SV=2 | Ecd | 1.05 |
| Q7M753 | Pantothenate kinase 2, mitochondrial OS=Mus musculus OX=10090 GN=Pank2 PE=1 SV=1 | Pank2 | 1.05 |
| O54784 | Death-associated protein kinase 3 OS=Mus musculus OX=10090 GN=Dapk3 PE=1 SV=1 | Dapk3 | 1.05 |
| O35250 | Exocyst complex component 7 OS=Mus musculus OX=10090 GN=Exoc7 PE=1 SV=2 | Exoc7 | 1.05 |
| Q8CIM8 | Integrator complex subunit 4 OS=Mus musculus OX=10090 GN=Ints4 PE=1 SV=1 | Ints4 | 1.05 |
| Q62083 | PRKCA-binding protein OS=Mus musculus OX=10090 GN=Pick1 PE=1 SV=2 | Pick1 | 1.05 |
| Q61151 | Serine/threonine-protein phosphatase 2A 56 kDa regulatory subunit epsilon isoform OS=Mus musculus OX=10090 GN=Ppp2r5e PE=1 SV=3 | Ppp2r5e | 1.05 |
| Q9DAW6 | U4/U6 small nuclear ribonucleoprotein Prp4 OS=Mus musculus OX=10090 GN=Prpf4 PE=1 SV=1 | Prpf4 | 1.05 |
| P61982 | 14-3-3 protein gamma OS=Mus musculus OX=10090 GN=Ywhag PE=1 SV=2 | Ywhag | 1.05 |
| Q9WVA3 | Mitotic checkpoint protein BUB3 OS=Mus musculus OX=10090 GN=Bub3 PE=1 SV=2 | Bub3 | 1.05 |
| Q9DCN1 | NAD-capped RNA hydrolase NUDT12 OS=Mus musculus OX=10090 GN=Nudt12 PE=1 SV=1 | Nudt12 | 1.05 |
| Q9JHR7 | Insulin-degrading enzyme OS=Mus musculus OX=10090 GN=Ide PE=1 SV=1 | Ide | 1.05 |
| Q9D6K7 | Tetratricopeptide repeat protein 33 OS=Mus musculus OX=10090 GN=Ttc33 PE=1 SV=1 | Ttc33 | 1.05 |
| Q70FJ1 | A-kinase anchor protein 9 OS=Mus musculus OX=10090 GN=Akap9 PE=1 SV=2 | Akap9 | 1.05 |
| Q80Y19 | Rho GTPase-activating protein 11A OS=Mus musculus OX=10090 GN=Arhgap11a PE=1 SV=2 | Arhgap11a | 1.05 |
| Q8R0H9 | ADP-ribosylation factor-binding protein GGA1 OS=Mus musculus OX=10090 GN=Gga1 PE=1 SV=1 | Gga1 | 1.05 |
| Q8BG67 | Protein EFR3 homolog A OS=Mus musculus OX=10090 GN=Efr3a PE=1 SV=1 | Efr3a | 1.05 |
| P61967 | AP-1 complex subunit sigma-1A OS=Mus musculus OX=10090 GN=Ap1s1 PE=1 SV=1 | Ap1s1 | 1.05 |
| Q60823 | RAC-beta serine/threonine-protein kinase OS=Mus musculus OX=10090 GN=Akt2 PE=1 SV=1 | Akt2 | 1.05 |
| O88622 | Poly(ADP-ribose) glycohydrolase OS=Mus musculus OX=10090 GN=Parg PE=1 SV=2 | Parg | 1.05 |
| Q921X6 | DNA-directed RNA polymerase III subunit RPC6 OS=Mus musculus OX=10090 GN=Polr3f PE=1 SV=1 | Polr3f | 1.05 |
| P63101 | 14-3-3 protein zeta/delta OS=Mus musculus OX=10090 GN=Ywhaz PE=1 SV=1 | Ywhaz | 1.05 |
| Q64521 | Glycerol-3-phosphate dehydrogenase, mitochondrial OS=Mus musculus OX=10090 GN=Gpd2 PE=1 SV=2 | Gpd2 | 1.05 |
| P0C7N9 | Proteasome assembly chaperone 4 OS=Mus musculus OX=10090 GN=Psmg4 PE=1 SV=1 | Psmg4 | 1.05 |
| Q8C754 | Vacuolar protein sorting-associated protein 52 homolog OS=Mus musculus OX=10090 GN=Vps52 PE=1 SV=1 | Vps52 | 1.05 |
| Q9WTX2 | Interferon-inducible double-stranded RNA-dependent protein kinase activator A OS=Mus musculus OX=10090 GN=Prkra PE=1 SV=1 | Prkra | 1.05 |
| Q9WVA4 | Transgelin-2 OS=Mus musculus OX=10090 GN=Tagln2 PE=1 SV=4 | Tagln2 | 1.05 |
| Q8VCI5 | Peroxisomal biogenesis factor 19 OS=Mus musculus OX=10090 GN=Pex19 PE=1 SV=1 | Pex19 | 1.05 |
| Q8VDQ8 | NAD-dependent protein deacetylase sirtuin-2 OS=Mus musculus OX=10090 GN=Sirt2 PE=1 SV=2 | Sirt2 | 1.05 |
| Q69ZK0 | Phosphatidylinositol 3,4,5-trisphosphate-dependent Rac exchanger 1 protein OS=Mus musculus OX=10090 GN=Prex1 PE=1 SV=2 | Prex1 | 1.05 |
| P28352 | DNA-(apurinic or apyrimidinic site) endonuclease OS=Mus musculus OX=10090 GN=Apex1 PE=1 SV=2 | Apex1 | 1.05 |
| Q9Z2W0 | Aspartyl aminopeptidase OS=Mus musculus OX=10090 GN=Dnpep PE=1 SV=2 | Dnpep | 1.05 |
| O08749 | Dihydrolipoyl dehydrogenase, mitochondrial OS=Mus musculus OX=10090 GN=Dld PE=1 SV=2 | Dld | 1.05 |
| Q8R123 | FAD synthase OS=Mus musculus OX=10090 GN=Flad1 PE=1 SV=1 | Flad1 | 1.05 |
| Q8BL74 | General transcription factor 3C polypeptide 2 OS=Mus musculus OX=10090 GN=Gtf3c2 PE=2 SV=2 | Gtf3c2 | 1.05 |
| Q8CAY6 | Acetyl-CoA acetyltransferase, cytosolic OS=Mus musculus OX=10090 GN=Acat2 PE=1 SV=2 | Acat2 | 1.05 |
| Q9CY94 | DNA replication complex GINS protein PSF3 OS=Mus musculus OX=10090 GN=Gins3 PE=2 SV=1 | Gins3 | 1.05 |
| Q8BP40 | Lysophosphatidic acid phosphatase type 6 OS=Mus musculus OX=10090 GN=Acp6 PE=1 SV=1 | Acp6 | 1.05 |
| Q9CQW2 | ADP-ribosylation factor-like protein 8B OS=Mus musculus OX=10090 GN=Arl8b PE=1 SV=1 | Arl8b | 1.05 |
| Q9D051 | Pyruvate dehydrogenase E1 component subunit beta, mitochondrial OS=Mus musculus OX=10090 GN=Pdhb PE=1 SV=1 | Pdhb | 1.05 |
| Q8BMB3 | Eukaryotic translation initiation factor 4E type 2 OS=Mus musculus OX=10090 GN=Eif4e2 PE=1 SV=1 | Eif4e2 | 1.05 |
| P60898 | DNA-directed RNA polymerase II subunit RPB9 OS=Mus musculus OX=10090 GN=Polr2i PE=1 SV=1 | Polr2i | 1.05 |
| Q8R5H1 | Ubiquitin carboxyl-terminal hydrolase 15 OS=Mus musculus OX=10090 GN=Usp15 PE=1 SV=1 | Usp15 | 1.05 |
| Q8BHZ4 | Zinc finger protein 592 OS=Mus musculus OX=10090 GN=Znf592 PE=1 SV=3 | Znf592 | 1.05 |
| Q9DBG7 | Signal recognition particle receptor subunit alpha OS=Mus musculus OX=10090 GN=Srpra PE=1 SV=1 | Srpra | 1.05 |
| Q8BWU5 | Probable tRNA N6-adenosine threonylcarbamoyltransferase OS=Mus musculus OX=10090 GN=Osgep PE=1 SV=2 | Osgep | 1.05 |
| Q8R0A7 | Uncharacterized protein KIAA0513 OS=Mus musculus OX=10090 GN=Kiaa0513 PE=1 SV=1 | Kiaa0513 | 1.05 |
| Q8C3Q9 | Caspase-9 OS=Mus musculus OX=10090 GN=Casp9 PE=1 SV=1 | Casp9 | 1.05 |
| P31750 | RAC-alpha serine/threonine-protein kinase OS=Mus musculus OX=10090 GN=Akt1 PE=1 SV=2 | Akt1 | 1.05 |
| Q8K135 | Dyslexia-associated protein KIAA0319-like protein OS=Mus musculus OX=10090 GN=Kiaa0319l PE=1 SV=1 | Kiaa0319l | 1.05 |
| Q9QUK3 | Protein CLN8 OS=Mus musculus OX=10090 GN=Cln8 PE=1 SV=1 | Cln8 | 1.05 |
| Q921F4 | Heterogeneous nuclear ribonucleoprotein L-like OS=Mus musculus OX=10090 GN=Hnrnpll PE=1 SV=3 | Hnrnpll | 1.05 |
| B2RWS6 | Histone acetyltransferase p300 OS=Mus musculus OX=10090 GN=Ep300 PE=1 SV=2 | Ep300 | 1.05 |
| Q9CQW9 | Interferon-induced transmembrane protein 3 OS=Mus musculus OX=10090 GN=Ifitm3 PE=1 SV=1 | Ifitm3 | 1.05 |
| O88455 | 7-dehydrocholesterol reductase OS=Mus musculus OX=10090 GN=Dhcr7 PE=1 SV=1 | Dhcr7 | 1.05 |
| Q6P4S6 | Serine/threonine-protein kinase SIK3 OS=Mus musculus OX=10090 GN=Sik3 PE=1 SV=3 | Sik3 | 1.05 |
| P47758 | Signal recognition particle receptor subunit beta OS=Mus musculus OX=10090 GN=Srprb PE=1 SV=1 | Srprb | 1.05 |
| Q6A026 | Sister chromatid cohesion protein PDS5 homolog A OS=Mus musculus OX=10090 GN=Pds5a PE=1 SV=3 | Pds5a | 1.05 |
| Q60902 | Epidermal growth factor receptor substrate 15-like 1 OS=Mus musculus OX=10090 GN=Eps15l1 PE=1 SV=3 | Eps15l1 | 1.05 |
| Q5SV80 | Unconventional myosin-XIX OS=Mus musculus OX=10090 GN=Myo19 PE=1 SV=1 | Myo19 | 1.05 |
| O08800 | Serpin B8 OS=Mus musculus OX=10090 GN=Serpinb8 PE=1 SV=2 | Serpinb8 | 1.05 |
| Q6P5F6 | Zinc transporter ZIP10 OS=Mus musculus OX=10090 GN=Slc39a10 PE=1 SV=1 | Slc39a10 | 1.05 |
| Q8K1Y2 | Serine/threonine-protein kinase D3 OS=Mus musculus OX=10090 GN=Prkd3 PE=1 SV=1 | Prkd3 | 1.05 |
| P47738 | Aldehyde dehydrogenase, mitochondrial OS=Mus musculus OX=10090 GN=Aldh2 PE=1 SV=1 | Aldh2 | 1.05 |
| Q11011 | Puromycin-sensitive aminopeptidase OS=Mus musculus OX=10090 GN=Npepps PE=1 SV=2 | Npepps | 1.05 |
| P46061 | Ran GTPase-activating protein 1 OS=Mus musculus OX=10090 GN=Rangap1 PE=1 SV=2 | Rangap1 | 1.05 |
| Q9CRT8 | Exportin-T OS=Mus musculus OX=10090 GN=Xpot PE=1 SV=3 | Xpot | 1.05 |
| Q80XQ2 | TBC1 domain family member 5 OS=Mus musculus OX=10090 GN=Tbc1d5 PE=1 SV=2 | Tbc1d5 | 1.05 |
| D3YXK2 | Scaffold attachment factor B1 OS=Mus musculus OX=10090 GN=Safb PE=1 SV=2 | Safb | 1.05 |
| P28659 | CUGBP Elav-like family member 1 OS=Mus musculus OX=10090 GN=Celf1 PE=1 SV=2 | Celf1 | 1.05 |
| Q8CHW4 | Translation initiation factor eIF-2B subunit epsilon OS=Mus musculus OX=10090 GN=Eif2b5 PE=1 SV=1 | Eif2b5 | 1.05 |
| Q3UDW8 | Heparan-alpha-glucosaminide N-acetyltransferase OS=Mus musculus OX=10090 GN=Hgsnat PE=1 SV=2 | Hgsnat | 1.05 |
| Q8BZN6 | Dedicator of cytokinesis protein 10 OS=Mus musculus OX=10090 GN=Dock10 PE=1 SV=3 | Dock10 | 1.05 |
| P32921 | Tryptophan--tRNA ligase, cytoplasmic OS=Mus musculus OX=10090 GN=Wars1 PE=1 SV=2 | Wars1 | 1.05 |
| Q923Z3 | Protein MTO1 homolog, mitochondrial OS=Mus musculus OX=10090 GN=Mto1 PE=2 SV=1 | Mto1 | 1.05 |
| Q9DCA5 | Ribosome biogenesis protein BRX1 homolog OS=Mus musculus OX=10090 GN=Brix1 PE=1 SV=3 | Brix1 | 1.05 |
| Q61263 | Sterol O-acyltransferase 1 OS=Mus musculus OX=10090 GN=Soat1 PE=1 SV=2 | Soat1 | 1.05 |
| Q8CJG0 | Protein argonaute-2 OS=Mus musculus OX=10090 GN=Ago2 PE=1 SV=3 | Ago2 | 1.05 |
| O55047 | Serine/threonine-protein kinase tousled-like 2 OS=Mus musculus OX=10090 GN=Tlk2 PE=1 SV=2 | Tlk2 | 1.05 |
| Q60631 | Growth factor receptor-bound protein 2 OS=Mus musculus OX=10090 GN=Grb2 PE=1 SV=1 | Grb2 | 1.05 |
| A2A4P0 | ATP-dependent RNA helicase DHX8 OS=Mus musculus OX=10090 GN=Dhx8 PE=2 SV=1 | Dhx8 | 1.05 |
| P61961 | Ubiquitin-fold modifier 1 OS=Mus musculus OX=10090 GN=Ufm1 PE=1 SV=1 | Ufm1 | 1.05 |
| P53995 | Anaphase-promoting complex subunit 1 OS=Mus musculus OX=10090 GN=Anapc1 PE=1 SV=2 | Anapc1 | 1.05 |
| Q91WN1 | DnaJ homolog subfamily C member 9 OS=Mus musculus OX=10090 GN=Dnajc9 PE=1 SV=2 | Dnajc9 | 1.05 |
| Q8K3J1 | NADH dehydrogenase [ubiquinone] iron-sulfur protein 8, mitochondrial OS=Mus musculus OX=10090 GN=Ndufs8 PE=1 SV=1 | Ndufs8 | 1.05 |
| Q9QYY8 | Spastin OS=Mus musculus OX=10090 GN=Spast PE=1 SV=3 | Spast | 1.05 |
| Q9CQR2 | 40S ribosomal protein S21 OS=Mus musculus OX=10090 GN=Rps21 PE=1 SV=1 | Rps21 | 1.05 |
| Q7TMK9 | Heterogeneous nuclear ribonucleoprotein Q OS=Mus musculus OX=10090 GN=Syncrip PE=1 SV=2 | Syncrip | 1.05 |
| Q8CH72 | E3 ubiquitin-protein ligase TRIM32 OS=Mus musculus OX=10090 GN=Trim32 PE=1 SV=2 | Trim32 | 1.05 |
| Q8CI71 | Syndetin OS=Mus musculus OX=10090 GN=Vps50 PE=1 SV=2 | Vps50 | 1.05 |
| Q9D8P4 | 39S ribosomal protein L17, mitochondrial OS=Mus musculus OX=10090 GN=Mrpl17 PE=1 SV=1 | Mrpl17 | 1.05 |
| P81117 | Nucleobindin-2 OS=Mus musculus OX=10090 GN=Nucb2 PE=1 SV=2 | Nucb2 | 1.05 |
| Q99104 | Unconventional myosin-Va OS=Mus musculus OX=10090 GN=Myo5a PE=1 SV=2 | Myo5a | 1.05 |
| Q91VR5 | ATP-dependent RNA helicase DDX1 OS=Mus musculus OX=10090 GN=Ddx1 PE=1 SV=1 | Ddx1 | 1.05 |
| Q9D819 | Inorganic pyrophosphatase OS=Mus musculus OX=10090 GN=Ppa1 PE=1 SV=1 | Ppa1 | 1.05 |
| Q6NSU3 | Glycosyltransferase 8 domain-containing protein 1 OS=Mus musculus OX=10090 GN=Glt8d1 PE=1 SV=1 | Glt8d1 | 1.05 |
| Q9D020 | Cytosolic 5'-nucleotidase 3A OS=Mus musculus OX=10090 GN=Nt5c3a PE=1 SV=4 | Nt5c3a | 1.05 |
| Q8C7K6 | Prenylcysteine oxidase-like OS=Mus musculus OX=10090 GN=Pcyox1l PE=1 SV=1 | Pcyox1l | 1.05 |
| Q91VJ4 | Serine/threonine-protein kinase 38 OS=Mus musculus OX=10090 GN=Stk38 PE=1 SV=1 | Stk38 | 1.06 |
| Q922J3 | CAP-Gly domain-containing linker protein 1 OS=Mus musculus OX=10090 GN=Clip1 PE=1 SV=1 | Clip1 | 1.06 |
| P57780 | Alpha-actinin-4 OS=Mus musculus OX=10090 GN=Actn4 PE=1 SV=1 | Actn4 | 1.06 |
| Q8BW10 | RNA-binding protein NOB1 OS=Mus musculus OX=10090 GN=Nob1 PE=1 SV=1 | Nob1 | 1.06 |
| P97825 | Jupiter microtubule associated homolog 1 OS=Mus musculus OX=10090 GN=Jpt1 PE=1 SV=3 | Jpt1 | 1.06 |
| P62702 | 40S ribosomal protein S4, X isoform OS=Mus musculus OX=10090 GN=Rps4x PE=1 SV=2 | Rps4x | 1.06 |
| Q8K012 | Formin-binding protein 1-like OS=Mus musculus OX=10090 GN=Fnbp1l PE=1 SV=2 | Fnbp1l | 1.06 |
| Q3TVP5 | Inactive ubiquitin thioesterase OTULINL OS=Mus musculus OX=10090 GN=Otulinl PE=1 SV=1 | Otulinl | 1.06 |
| Q8BMF4 | Dihydrolipoyllysine-residue acetyltransferase component of pyruvate dehydrogenase complex, mitochondrial OS=Mus musculus OX=10090 GN=Dlat PE=1 SV=2 | Dlat | 1.06 |
| Q921E6 | Polycomb protein EED OS=Mus musculus OX=10090 GN=Eed PE=1 SV=1 | Eed | 1.06 |
| Q8K2F8 | Protein LSM14 homolog A OS=Mus musculus OX=10090 GN=Lsm14a PE=1 SV=1 | Lsm14a | 1.06 |
| Q9Z0E0 | Neurochondrin OS=Mus musculus OX=10090 GN=Ncdn PE=1 SV=1 | Ncdn | 1.06 |
| P49312 | Heterogeneous nuclear ribonucleoprotein A1 OS=Mus musculus OX=10090 GN=Hnrnpa1 PE=1 SV=2 | Hnrnpa1 | 1.06 |
| Q3UHX2 | 28 kDa heat- and acid-stable phosphoprotein OS=Mus musculus OX=10090 GN=Pdap1 PE=1 SV=1 | Pdap1 | 1.06 |
| Q80UM7 | Mannosyl-oligosaccharide glucosidase OS=Mus musculus OX=10090 GN=Mogs PE=1 SV=1 | Mogs | 1.06 |
| Q60862 | Origin recognition complex subunit 2 OS=Mus musculus OX=10090 GN=Orc2 PE=1 SV=1 | Orc2 | 1.06 |
| Q9CZI9 | Apoptosis-enhancing nuclease OS=Mus musculus OX=10090 GN=Aen PE=2 SV=1 | Aen | 1.06 |
| P61021 | Ras-related protein Rab-5B OS=Mus musculus OX=10090 GN=Rab5b PE=1 SV=1 | Rab5b | 1.06 |
| Q9R0M5 | Thiamin pyrophosphokinase 1 OS=Mus musculus OX=10090 GN=Tpk1 PE=1 SV=1 | Tpk1 | 1.06 |
| Q6PD26 | GPI transamidase component PIG-S OS=Mus musculus OX=10090 GN=Pigs PE=1 SV=3 | Pigs | 1.06 |
| Q9Z321 | DNA topoisomerase 3-beta-1 OS=Mus musculus OX=10090 GN=Top3b PE=1 SV=1 | Top3b | 1.06 |
| Q921M7 | CYFIP-related Rac1 interactor B OS=Mus musculus OX=10090 GN=Cyrib PE=1 SV=1 | Cyrib | 1.06 |
| O08811 | General transcription and DNA repair factor IIH helicase subunit XPD OS=Mus musculus OX=10090 GN=Ercc2 PE=1 SV=2 | Ercc2 | 1.06 |
| Q9CXY9 | GPI-anchor transamidase OS=Mus musculus OX=10090 GN=Pigk PE=1 SV=2 | Pigk | 1.06 |
| Q3U0V1 | Far upstream element-binding protein 2 OS=Mus musculus OX=10090 GN=Khsrp PE=1 SV=2 | Khsrp | 1.06 |
| Q8BH95 | Enoyl-CoA hydratase, mitochondrial OS=Mus musculus OX=10090 GN=Echs1 PE=1 SV=1 | Echs1 | 1.06 |
| Q8BFR4 | N-acetylglucosamine-6-sulfatase OS=Mus musculus OX=10090 GN=Gns PE=1 SV=1 | Gns | 1.06 |
| Q9ESV0 | ATP-dependent RNA helicase DDX24 OS=Mus musculus OX=10090 GN=Ddx24 PE=1 SV=2 | Ddx24 | 1.06 |
| Q9CR59 | Growth arrest and DNA damage-inducible proteins-interacting protein 1 OS=Mus musculus OX=10090 GN=Gadd45gip1 PE=1 SV=1 | Gadd45gip1 | 1.06 |
| Q5U458 | DnaJ homolog subfamily C member 11 OS=Mus musculus OX=10090 GN=Dnajc11 PE=1 SV=2 | Dnajc11 | 1.06 |
| Q8BMJ2 | Leucine--tRNA ligase, cytoplasmic OS=Mus musculus OX=10090 GN=Lars1 PE=1 SV=2 | Lars1 | 1.06 |
| Q924T2 | 28S ribosomal protein S2, mitochondrial OS=Mus musculus OX=10090 GN=Mrps2 PE=1 SV=1 | Mrps2 | 1.06 |
| Q8C7R4 | Ubiquitin-like modifier-activating enzyme 6 OS=Mus musculus OX=10090 GN=Uba6 PE=1 SV=1 | Uba6 | 1.06 |
| Q9D902 | General transcription factor IIE subunit 2 OS=Mus musculus OX=10090 GN=Gtf2e2 PE=1 SV=2 | Gtf2e2 | 1.06 |
| O08992 | Syntenin-1 OS=Mus musculus OX=10090 GN=Sdcbp PE=1 SV=1 | Sdcbp | 1.06 |
| Q61233 | Plastin-2 OS=Mus musculus OX=10090 GN=Lcp1 PE=1 SV=4 | Lcp1 | 1.06 |
| Q8BGQ7 | Alanine--tRNA ligase, cytoplasmic OS=Mus musculus OX=10090 GN=Aars1 PE=1 SV=1 | Aars1 | 1.06 |
| Q58A65 | C-Jun-amino-terminal kinase-interacting protein 4 OS=Mus musculus OX=10090 GN=Spag9 PE=1 SV=2 | Spag9 | 1.06 |
| P18572 | Basigin OS=Mus musculus OX=10090 GN=Bsg PE=1 SV=2 | Bsg | 1.06 |
| Q9D0L8 | mRNA cap guanine-N7 methyltransferase OS=Mus musculus OX=10090 GN=Rnmt PE=1 SV=1 | Rnmt | 1.06 |
| P61965 | WD repeat-containing protein 5 OS=Mus musculus OX=10090 GN=Wdr5 PE=1 SV=1 | Wdr5 | 1.06 |
| P58681 | Toll-like receptor 7 OS=Mus musculus OX=10090 GN=Tlr7 PE=1 SV=1 | Tlr7 | 1.06 |
| O88448 | Kinesin light chain 2 OS=Mus musculus OX=10090 GN=Klc2 PE=1 SV=1 | Klc2 | 1.06 |
| Q8C5N5 | Programmed cell death protein 2-like OS=Mus musculus OX=10090 GN=Pdcd2l PE=1 SV=1 | Pdcd2l | 1.06 |
| Q3UM18 | Large subunit GTPase 1 homolog OS=Mus musculus OX=10090 GN=Lsg1 PE=1 SV=2 | Lsg1 | 1.06 |
| Q9WV80 | Sorting nexin-1 OS=Mus musculus OX=10090 GN=Snx1 PE=1 SV=1 | Snx1 | 1.06 |
| P58501 | PAX3- and PAX7-binding protein 1 OS=Mus musculus OX=10090 GN=Paxbp1 PE=1 SV=3 | Paxbp1 | 1.06 |
| Q8BGR9 | Ubiquitin-like domain-containing CTD phosphatase 1 OS=Mus musculus OX=10090 GN=Ublcp1 PE=1 SV=1 | Ublcp1 | 1.06 |
| Q3UMW8 | Ceroid-lipofuscinosis neuronal protein 5 homolog OS=Mus musculus OX=10090 GN=Cln5 PE=1 SV=1 | Cln5 | 1.06 |
| Q9CR09 | Ubiquitin-fold modifier-conjugating enzyme 1 OS=Mus musculus OX=10090 GN=Ufc1 PE=1 SV=1 | Ufc1 | 1.06 |
| Q99J95 | Cyclin-dependent kinase 9 OS=Mus musculus OX=10090 GN=Cdk9 PE=1 SV=1 | Cdk9 | 1.06 |
| Q9R049 | E3 ubiquitin-protein ligase AMFR OS=Mus musculus OX=10090 GN=Amfr PE=1 SV=2 | Amfr | 1.06 |
| Q8BUE4 | Ferroptosis suppressor protein 1 OS=Mus musculus OX=10090 GN=Aifm2 PE=1 SV=1 | Aifm2 | 1.06 |
| O89023 | Tripeptidyl-peptidase 1 OS=Mus musculus OX=10090 GN=Tpp1 PE=1 SV=2 | Tpp1 | 1.06 |
| Q3UVK0 | Endoplasmic reticulum metallopeptidase 1 OS=Mus musculus OX=10090 GN=Ermp1 PE=1 SV=2 | Ermp1 | 1.06 |
| Q8VBZ3 | Cleft lip and palate transmembrane protein 1 homolog OS=Mus musculus OX=10090 GN=Clptm1 PE=1 SV=1 | Clptm1 | 1.06 |
| Q6ZWX6 | Eukaryotic translation initiation factor 2 subunit 1 OS=Mus musculus OX=10090 GN=Eif2s1 PE=1 SV=3 | Eif2s1 | 1.06 |
| P52293 | Importin subunit alpha-1 OS=Mus musculus OX=10090 GN=Kpna2 PE=1 SV=2 | Kpna2 | 1.06 |
| Q2YDW2 | Protein misato homolog 1 OS=Mus musculus OX=10090 GN=Msto1 PE=1 SV=1 | Msto1 | 1.06 |
| Q8VH51 | RNA-binding protein 39 OS=Mus musculus OX=10090 GN=Rbm39 PE=1 SV=2 | Rbm39 | 1.06 |
| Q9JKN1 | Zinc transporter 7 OS=Mus musculus OX=10090 GN=Slc30a7 PE=1 SV=1 | Slc30a7 | 1.06 |
| Q99LC8 | Translation initiation factor eIF-2B subunit alpha OS=Mus musculus OX=10090 GN=Eif2b1 PE=1 SV=1 | Eif2b1 | 1.06 |
| A2AGT5 | Cytoskeleton-associated protein 5 OS=Mus musculus OX=10090 GN=Ckap5 PE=1 SV=1 | Ckap5 | 1.06 |
| Q9CXF7 | Chromodomain-helicase-DNA-binding protein 1-like OS=Mus musculus OX=10090 GN=Chd1l PE=1 SV=1 | Chd1l | 1.06 |
| Q9CS42 | Ribose-phosphate pyrophosphokinase 2 OS=Mus musculus OX=10090 GN=Prps2 PE=1 SV=4 | Prps2 | 1.06 |
| Q9D8U8 | Sorting nexin-5 OS=Mus musculus OX=10090 GN=Snx5 PE=1 SV=1 | Snx5 | 1.06 |
| Q8R420 | Phospholipid-transporting ATPase ABCA3 OS=Mus musculus OX=10090 GN=Abca3 PE=1 SV=3 | Abca3 | 1.06 |
| Q6ZWU9 | 40S ribosomal protein S27 OS=Mus musculus OX=10090 GN=Rps27 PE=1 SV=3 | Rps27 | 1.06 |
| Q9D2P4 | Ubiquitin-related modifier 1 OS=Mus musculus OX=10090 GN=Urm1 PE=1 SV=1 | Urm1 | 1.06 |
| Q91WU5 | Arsenite methyltransferase OS=Mus musculus OX=10090 GN=As3mt PE=1 SV=2 | As3mt | 1.06 |
| Q9ES52 | Phosphatidylinositol 3,4,5-trisphosphate 5-phosphatase 1 OS=Mus musculus OX=10090 GN=Inpp5d PE=1 SV=2 | Inpp5d | 1.06 |
| P57787 | Monocarboxylate transporter 4 OS=Mus musculus OX=10090 GN=Slc16a3 PE=1 SV=1 | Slc16a3 | 1.06 |
| P14206 | 40S ribosomal protein SA OS=Mus musculus OX=10090 GN=Rpsa PE=1 SV=4 | Rpsa | 1.06 |
| P26369 | Splicing factor U2AF 65 kDa subunit OS=Mus musculus OX=10090 GN=U2af2 PE=1 SV=3 | U2af2 | 1.06 |
| Q8CI04 | Conserved oligomeric Golgi complex subunit 3 OS=Mus musculus OX=10090 GN=Cog3 PE=1 SV=3 | Cog3 | 1.06 |
| E9Q555 | E3 ubiquitin-protein ligase RNF213 OS=Mus musculus OX=10090 GN=Rnf213 PE=1 SV=3 | Rnf213 | 1.06 |
| P50516 | V-type proton ATPase catalytic subunit A OS=Mus musculus OX=10090 GN=Atp6v1a PE=1 SV=2 | Atp6v1a | 1.06 |
| P29351 | Tyrosine-protein phosphatase non-receptor type 6 OS=Mus musculus OX=10090 GN=Ptpn6 PE=1 SV=2 | Ptpn6 | 1.06 |
| P01902 | H-2 class I histocompatibility antigen, K-D alpha chain OS=Mus musculus OX=10090 GN=H2-K1 PE=1 SV=1 | H2-K1 | 1.06 |
| Q8BGS2 | BolA-like protein 2 OS=Mus musculus OX=10090 GN=Bola2 PE=1 SV=1 | Bola2 | 1.06 |
| P14115 | 60S ribosomal protein L27a OS=Mus musculus OX=10090 GN=Rpl27a PE=1 SV=5 | Rpl27a | 1.06 |
| Q8VE91 | Reticulophagy regulator 1 OS=Mus musculus OX=10090 GN=Retreg1 PE=1 SV=2 | Retreg1 | 1.06 |
| Q80U93 | Nuclear pore complex protein Nup214 OS=Mus musculus OX=10090 GN=Nup214 PE=1 SV=2 | Nup214 | 1.06 |
| O35900 | U6 snRNA-associated Sm-like protein LSm2 OS=Mus musculus OX=10090 GN=Lsm2 PE=1 SV=1 | Lsm2 | 1.06 |
| Q8C129 | Leucyl-cystinyl aminopeptidase OS=Mus musculus OX=10090 GN=Lnpep PE=1 SV=1 | Lnpep | 1.06 |
| Q3ULJ0 | Glycerol-3-phosphate dehydrogenase 1-like protein OS=Mus musculus OX=10090 GN=Gpd1l PE=1 SV=2 | Gpd1l | 1.06 |
| Q64455 | Receptor-type tyrosine-protein phosphatase eta OS=Mus musculus OX=10090 GN=Ptprj PE=1 SV=2 | Ptprj | 1.06 |
| Q33DR2 | All trans-polyprenyl-diphosphate synthase PDSS1 OS=Mus musculus OX=10090 GN=Pdss1 PE=1 SV=1 | Pdss1 | 1.06 |
| Q9WVK4 | EH domain-containing protein 1 OS=Mus musculus OX=10090 GN=Ehd1 PE=1 SV=1 | Ehd1 | 1.06 |
| P11881 | Inositol 1,4,5-trisphosphate receptor type 1 OS=Mus musculus OX=10090 GN=Itpr1 PE=1 SV=2 | Itpr1 | 1.06 |
| P00375 | Dihydrofolate reductase OS=Mus musculus OX=10090 GN=Dhfr PE=1 SV=3 | Dhfr | 1.06 |
| P09411 | Phosphoglycerate kinase 1 OS=Mus musculus OX=10090 GN=Pgk1 PE=1 SV=4 | Pgk1 | 1.06 |
| Q9WVG6 | Histone-arginine methyltransferase CARM1 OS=Mus musculus OX=10090 GN=Carm1 PE=1 SV=2 | Carm1 | 1.06 |
| Q8CIG8 | Protein arginine N-methyltransferase 5 OS=Mus musculus OX=10090 GN=Prmt5 PE=1 SV=3 | Prmt5 | 1.06 |
| P49586 | Choline-phosphate cytidylyltransferase A OS=Mus musculus OX=10090 GN=Pcyt1a PE=1 SV=1 | Pcyt1a | 1.06 |
| Q6P1G0 | HEAT repeat-containing protein 6 OS=Mus musculus OX=10090 GN=Heatr6 PE=1 SV=1 | Heatr6 | 1.06 |
| Q91YT7 | YTH domain-containing family protein 2 OS=Mus musculus OX=10090 GN=Ythdf2 PE=1 SV=1 | Ythdf2 | 1.06 |
| Q6PHQ8 | N-alpha-acetyltransferase 35, NatC auxiliary subunit OS=Mus musculus OX=10090 GN=Naa35 PE=1 SV=1 | Naa35 | 1.06 |
| Q5SYD0 | Unconventional myosin-Id OS=Mus musculus OX=10090 GN=Myo1d PE=1 SV=1 | Myo1d | 1.06 |
| O35379 | Multidrug resistance-associated protein 1 OS=Mus musculus OX=10090 GN=Abcc1 PE=1 SV=1 | Abcc1 | 1.06 |
| Q60648 | Ganglioside GM2 activator OS=Mus musculus OX=10090 GN=Gm2a PE=1 SV=2 | Gm2a | 1.06 |
| Q6P549 | Phosphatidylinositol 3,4,5-trisphosphate 5-phosphatase 2 OS=Mus musculus OX=10090 GN=Inppl1 PE=1 SV=1 | Inppl1 | 1.06 |
| Q8CB96 | Ras association domain-containing protein 4 OS=Mus musculus OX=10090 GN=Rassf4 PE=1 SV=1 | Rassf4 | 1.06 |
| Q6PDI5 | Proteasome adapter and scaffold protein ECM29 OS=Mus musculus OX=10090 GN=Ecpas PE=1 SV=3 | Ecpas | 1.06 |
| Q7JJ13 | Bromodomain-containing protein 2 OS=Mus musculus OX=10090 GN=Brd2 PE=1 SV=1 | Brd2 | 1.06 |
| Q05D44 | Eukaryotic translation initiation factor 5B OS=Mus musculus OX=10090 GN=Eif5b PE=1 SV=2 | Eif5b | 1.06 |
| Q8JZU0 | NAD(P)H pyrophosphatase NUDT13, mitochondrial OS=Mus musculus OX=10090 GN=Nudt13 PE=1 SV=2 | Nudt13 | 1.06 |
| P61027 | Ras-related protein Rab-10 OS=Mus musculus OX=10090 GN=Rab10 PE=1 SV=1 | Rab10 | 1.06 |
| Q60932 | Voltage-dependent anion-selective channel protein 1 OS=Mus musculus OX=10090 GN=Vdac1 PE=1 SV=3 | Vdac1 | 1.06 |
| Q8CAS9 | Protein mono-ADP-ribosyltransferase PARP9 OS=Mus musculus OX=10090 GN=Parp9 PE=1 SV=2 | Parp9 | 1.06 |
| O70126 | Aurora kinase B OS=Mus musculus OX=10090 GN=Aurkb PE=1 SV=2 | Aurkb | 1.06 |
| Q9DAR7 | m7GpppX diphosphatase OS=Mus musculus OX=10090 GN=Dcps PE=1 SV=1 | Dcps | 1.06 |
| P97310 | DNA replication licensing factor MCM2 OS=Mus musculus OX=10090 GN=Mcm2 PE=1 SV=3 | Mcm2 | 1.06 |
| P46062 | Signal-induced proliferation-associated protein 1 OS=Mus musculus OX=10090 GN=Sipa1 PE=1 SV=2 | Sipa1 | 1.06 |
| Q8CC88 | von Willebrand factor A domain-containing protein 8 OS=Mus musculus OX=10090 GN=Vwa8 PE=1 SV=2 | Vwa8 | 1.06 |
| Q8BIG7 | Catechol O-methyltransferase domain-containing protein 1 OS=Mus musculus OX=10090 GN=Comtd1 PE=1 SV=1 | Comtd1 | 1.06 |
| A2A432 | Cullin-4B OS=Mus musculus OX=10090 GN=Cul4b PE=1 SV=1 | Cul4b | 1.06 |
| P56477 | Interferon regulatory factor 5 OS=Mus musculus OX=10090 GN=Irf5 PE=1 SV=1 | Irf5 | 1.06 |
| Q9CZU6 | Citrate synthase, mitochondrial OS=Mus musculus OX=10090 GN=Cs PE=1 SV=1 | Cs | 1.06 |
| P63280 | SUMO-conjugating enzyme UBC9 OS=Mus musculus OX=10090 GN=Ube2i PE=1 SV=1 | Ube2i | 1.06 |
| Q9CXZ1 | NADH dehydrogenase [ubiquinone] iron-sulfur protein 4, mitochondrial OS=Mus musculus OX=10090 GN=Ndufs4 PE=1 SV=3 | Ndufs4 | 1.06 |
| Q9D2E2 | Target of EGR1 protein 1 OS=Mus musculus OX=10090 GN=Toe1 PE=1 SV=1 | Toe1 | 1.06 |
| Q8C1D8 | Protein IWS1 homolog OS=Mus musculus OX=10090 GN=Iws1 PE=1 SV=1 | Iws1 | 1.06 |
| Q9JKF6 | Nectin-1 OS=Mus musculus OX=10090 GN=Nectin1 PE=1 SV=3 | Nectin1 | 1.06 |
| P25799 | Nuclear factor NF-kappa-B p105 subunit OS=Mus musculus OX=10090 GN=Nfkb1 PE=1 SV=2 | Nfkb1 | 1.06 |
| P53798 | Squalene synthase OS=Mus musculus OX=10090 GN=Fdft1 PE=1 SV=2 | Fdft1 | 1.07 |
| Q9D824 | Pre-mRNA 3'-end-processing factor FIP1 OS=Mus musculus OX=10090 GN=Fip1l1 PE=1 SV=1 | Fip1l1 | 1.07 |
| P45878 | Peptidyl-prolyl cis-trans isomerase FKBP2 OS=Mus musculus OX=10090 GN=Fkbp2 PE=1 SV=1 | Fkbp2 | 1.07 |
| Q9Z2U1 | Proteasome subunit alpha type-5 OS=Mus musculus OX=10090 GN=Psma5 PE=1 SV=1 | Psma5 | 1.07 |
| O35405 | 5'-3' exonuclease PLD3 OS=Mus musculus OX=10090 GN=Pld3 PE=1 SV=1 | Pld3 | 1.07 |
| P97762 | Retinitis pigmentosa 9 protein homolog OS=Mus musculus OX=10090 GN=rp9 PE=1 SV=1 | rp9 | 1.07 |
| Q8K339 | DNA/RNA-binding protein KIN17 OS=Mus musculus OX=10090 GN=Kin PE=1 SV=1 | Kin | 1.07 |
| Q8BGW1 | Alpha-ketoglutarate-dependent dioxygenase FTO OS=Mus musculus OX=10090 GN=Fto PE=1 SV=1 | Fto | 1.07 |
| Q7TMB8 | Cytoplasmic FMR1-interacting protein 1 OS=Mus musculus OX=10090 GN=Cyfip1 PE=1 SV=1 | Cyfip1 | 1.07 |
| Q63932 | Dual specificity mitogen-activated protein kinase kinase 2 OS=Mus musculus OX=10090 GN=Map2k2 PE=1 SV=2 | Map2k2 | 1.07 |
| Q6TEK5 | Vitamin K epoxide reductase complex subunit 1-like protein 1 OS=Mus musculus OX=10090 GN=Vkorc1l1 PE=1 SV=1 | Vkorc1l1 | 1.07 |
| Q8BY87 | Ubiquitin carboxyl-terminal hydrolase 47 OS=Mus musculus OX=10090 GN=Usp47 PE=1 SV=2 | Usp47 | 1.07 |
| Q8JZX4 | Splicing factor 45 OS=Mus musculus OX=10090 GN=Rbm17 PE=1 SV=1 | Rbm17 | 1.07 |
| Q9JKZ2 | Sodium/myo-inositol cotransporter OS=Mus musculus OX=10090 GN=Slc5a3 PE=1 SV=2 | Slc5a3 | 1.07 |
| Q9DCH4 | Eukaryotic translation initiation factor 3 subunit F OS=Mus musculus OX=10090 GN=Eif3f PE=1 SV=2 | Eif3f | 1.07 |
| P27641 | X-ray repair cross-complementing protein 5 OS=Mus musculus OX=10090 GN=Xrcc5 PE=1 SV=4 | Xrcc5 | 1.07 |
| Q9CPW2 | Ferredoxin-2, mitochondrial OS=Mus musculus OX=10090 GN=Fdx2 PE=1 SV=1 | Fdx2 | 1.07 |
| Q62167 | ATP-dependent RNA helicase DDX3X OS=Mus musculus OX=10090 GN=Ddx3x PE=1 SV=3 | Ddx3x | 1.07 |
| P49962 | Signal recognition particle 9 kDa protein OS=Mus musculus OX=10090 GN=Srp9 PE=1 SV=2 | Srp9 | 1.07 |
| P09926 | Surfeit locus protein 2 OS=Mus musculus OX=10090 GN=Surf2 PE=1 SV=1 | Surf2 | 1.07 |
| Q8C3I8 | Protein HGH1 homolog OS=Mus musculus OX=10090 GN=Hgh1 PE=1 SV=1 | Hgh1 | 1.07 |
| Q8R3C6 | Probable RNA-binding protein 19 OS=Mus musculus OX=10090 GN=Rbm19 PE=1 SV=1 | Rbm19 | 1.07 |
| Q6IRU2 | Tropomyosin alpha-4 chain OS=Mus musculus OX=10090 GN=Tpm4 PE=1 SV=3 | Tpm4 | 1.07 |
| Q9D8Y1 | Transmembrane protein 126A OS=Mus musculus OX=10090 GN=Tmem126a PE=1 SV=1 | Tmem126a | 1.07 |
| P34884 | Macrophage migration inhibitory factor OS=Mus musculus OX=10090 GN=Mif PE=1 SV=2 | Mif | 1.07 |
| Q9Z2D0 | Myotubularin-related protein 9 OS=Mus musculus OX=10090 GN=Mtmr9 PE=1 SV=2 | Mtmr9 | 1.07 |
| Q60790 | Ras GTPase-activating protein 3 OS=Mus musculus OX=10090 GN=Rasa3 PE=1 SV=2 | Rasa3 | 1.07 |
| Q9Z2L7 | Cytokine receptor-like factor 3 OS=Mus musculus OX=10090 GN=Crlf3 PE=1 SV=1 | Crlf3 | 1.07 |
| Q0VGB7 | Serine/threonine-protein phosphatase 4 regulatory subunit 2 OS=Mus musculus OX=10090 GN=Ppp4r2 PE=1 SV=1 | Ppp4r2 | 1.07 |
| Q52KI8 | Serine/arginine repetitive matrix protein 1 OS=Mus musculus OX=10090 GN=Srrm1 PE=1 SV=2 | Srrm1 | 1.07 |
| Q9DAU9 | Zinc finger protein 654 OS=Mus musculus OX=10090 GN=Znf654 PE=1 SV=3 | Znf654 | 1.07 |
| P97450 | ATP synthase-coupling factor 6, mitochondrial OS=Mus musculus OX=10090 GN=Atp5pf PE=1 SV=1 | Atp5pf | 1.07 |
| Q3USH5 | Splicing factor, suppressor of white-apricot homolog OS=Mus musculus OX=10090 GN=Sfswap PE=1 SV=2 | Sfswap | 1.07 |
| O70492 | Sorting nexin-3 OS=Mus musculus OX=10090 GN=Snx3 PE=1 SV=3 | Snx3 | 1.07 |
| Q9D8Z6 | Autophagy-related protein 101 OS=Mus musculus OX=10090 GN=Atg101 PE=1 SV=1 | Atg101 | 1.07 |
| Q8K2D3 | Enhancer of mRNA-decapping protein 3 OS=Mus musculus OX=10090 GN=Edc3 PE=1 SV=1 | Edc3 | 1.07 |
| Q6PDY2 | 2-aminoethanethiol dioxygenase OS=Mus musculus OX=10090 GN=Ado PE=1 SV=2 | Ado | 1.07 |
| P54731 | FAS-associated factor 1 OS=Mus musculus OX=10090 GN=Faf1 PE=1 SV=2 | Faf1 | 1.07 |
| P0DN34 | NADH dehydrogenase [ubiquinone] 1 beta subcomplex subunit 1 OS=Mus musculus OX=10090 GN=Ndufb1 PE=1 SV=1 | Ndufb1 | 1.07 |
| P54728 | UV excision repair protein RAD23 homolog B OS=Mus musculus OX=10090 GN=Rad23b PE=1 SV=2 | Rad23b | 1.07 |
| Q99LZ3 | DNA replication complex GINS protein SLD5 OS=Mus musculus OX=10090 GN=Gins4 PE=1 SV=1 | Gins4 | 1.07 |
| Q60848 | Lymphocyte-specific helicase OS=Mus musculus OX=10090 GN=Hells PE=1 SV=2 | Hells | 1.07 |
| E9PYH6 | Histone-lysine N-methyltransferase SETD1A OS=Mus musculus OX=10090 GN=Setd1a PE=1 SV=1 | Setd1a | 1.07 |
| Q62073 | Mitogen-activated protein kinase kinase kinase 7 OS=Mus musculus OX=10090 GN=Map3k7 PE=1 SV=1 | Map3k7 | 1.07 |
| O35648 | Centrin-3 OS=Mus musculus OX=10090 GN=Cetn3 PE=1 SV=1 | Cetn3 | 1.07 |
| Q61211 | Eukaryotic translation initiation factor 2D OS=Mus musculus OX=10090 GN=Eif2d PE=1 SV=3 | Eif2d | 1.07 |
| Q8BJZ4 | 28S ribosomal protein S35, mitochondrial OS=Mus musculus OX=10090 GN=Mrps35 PE=1 SV=2 | Mrps35 | 1.07 |
| Q60855 | Receptor-interacting serine/threonine-protein kinase 1 OS=Mus musculus OX=10090 GN=Ripk1 PE=1 SV=1 | Ripk1 | 1.07 |
| Q99LQ7 | Plasmanylethanolamine desaturase OS=Mus musculus OX=10090 GN=PEDS1 PE=1 SV=1 | PEDS1 | 1.07 |
| P97461 | 40S ribosomal protein S5 OS=Mus musculus OX=10090 GN=Rps5 PE=1 SV=3 | Rps5 | 1.07 |
| Q8K3H0 | DCC-interacting protein 13-alpha OS=Mus musculus OX=10090 GN=Appl1 PE=1 SV=1 | Appl1 | 1.07 |
| Q6DIC0 | Probable global transcription activator SNF2L2 OS=Mus musculus OX=10090 GN=Smarca2 PE=1 SV=1 | Smarca2 | 1.07 |
| Q9WUK4 | Replication factor C subunit 2 OS=Mus musculus OX=10090 GN=Rfc2 PE=1 SV=1 | Rfc2 | 1.07 |
| Q99KK7 | Dipeptidyl peptidase 3 OS=Mus musculus OX=10090 GN=Dpp3 PE=1 SV=2 | Dpp3 | 1.07 |
| Q3TYX3 | Histone-lysine N-trimethyltransferase SMYD5 OS=Mus musculus OX=10090 GN=Smyd5 PE=1 SV=2 | Smyd5 | 1.07 |
| Q9JI10 | Serine/threonine-protein kinase 3 OS=Mus musculus OX=10090 GN=Stk3 PE=1 SV=1 | Stk3 | 1.07 |
| Q9EP82 | tRNA (guanine-N(7)-)-methyltransferase non-catalytic subunit WDR4 OS=Mus musculus OX=10090 GN=Wdr4 PE=1 SV=2 | Wdr4 | 1.07 |
| P53811 | Phosphatidylinositol transfer protein beta isoform OS=Mus musculus OX=10090 GN=Pitpnb PE=1 SV=2 | Pitpnb | 1.07 |
| Q9CQ06 | 39S ribosomal protein L24, mitochondrial OS=Mus musculus OX=10090 GN=Mrpl24 PE=1 SV=1 | Mrpl24 | 1.07 |
| Q99NB9 | Splicing factor 3B subunit 1 OS=Mus musculus OX=10090 GN=Sf3b1 PE=1 SV=1 | Sf3b1 | 1.07 |
| O88738 | Baculoviral IAP repeat-containing protein 6 OS=Mus musculus OX=10090 GN=Birc6 PE=1 SV=2 | Birc6 | 1.07 |
| Q8BZR9 | Nuclear cap-binding protein subunit 3 OS=Mus musculus OX=10090 GN=Ncbp3 PE=1 SV=1 | Ncbp3 | 1.07 |
| Q9JIG7 | Coiled-coil domain-containing protein 22 OS=Mus musculus OX=10090 GN=Ccdc22 PE=1 SV=1 | Ccdc22 | 1.07 |
| P59997 | Lysine-specific demethylase 2A OS=Mus musculus OX=10090 GN=Kdm2a PE=1 SV=2 | Kdm2a | 1.07 |
| P56213 | FAD-linked sulfhydryl oxidase ALR OS=Mus musculus OX=10090 GN=Gfer PE=1 SV=2 | Gfer | 1.07 |
| Q9Z2I8 | Succinate--CoA ligase [GDP-forming] subunit beta, mitochondrial OS=Mus musculus OX=10090 GN=Suclg2 PE=1 SV=3 | Suclg2 | 1.07 |
| Q8CHG7 | Rap guanine nucleotide exchange factor 2 OS=Mus musculus OX=10090 GN=Rapgef2 PE=1 SV=2 | Rapgef2 | 1.07 |
| P97452 | Ribosome biogenesis protein BOP1 OS=Mus musculus OX=10090 GN=Bop1 PE=1 SV=1 | Bop1 | 1.07 |
| P62874 | Guanine nucleotide-binding protein G(I)/G(S)/G(T) subunit beta-1 OS=Mus musculus OX=10090 GN=Gnb1 PE=1 SV=3 | Gnb1 | 1.07 |
| Q8R307 | Vacuolar protein sorting-associated protein 18 homolog OS=Mus musculus OX=10090 GN=Vps18 PE=1 SV=2 | Vps18 | 1.07 |
| O88895 | Histone deacetylase 3 OS=Mus musculus OX=10090 GN=Hdac3 PE=1 SV=1 | Hdac3 | 1.07 |
| O88986 | 2-amino-3-ketobutyrate coenzyme A ligase, mitochondrial OS=Mus musculus OX=10090 GN=Gcat PE=1 SV=2 | Gcat | 1.07 |
| P17751 | Triosephosphate isomerase OS=Mus musculus OX=10090 GN=Tpi1 PE=1 SV=5 | Tpi1 | 1.07 |
| Q91YX5 | Acyl-CoA:lysophosphatidylglycerol acyltransferase 1 OS=Mus musculus OX=10090 GN=Lpgat1 PE=1 SV=1 | Lpgat1 | 1.07 |
| Q8R1S0 | Ubiquinone biosynthesis monooxygenase COQ6, mitochondrial OS=Mus musculus OX=10090 GN=Coq6 PE=1 SV=3 | Coq6 | 1.07 |
| Q8CEE6 | PAS domain-containing serine/threonine-protein kinase OS=Mus musculus OX=10090 GN=Pask PE=1 SV=3 | Pask | 1.07 |
| Q8CFX1 | GDH/6PGL endoplasmic bifunctional protein OS=Mus musculus OX=10090 GN=H6pd PE=1 SV=2 | H6pd | 1.07 |
| Q62315 | Protein Jumonji OS=Mus musculus OX=10090 GN=Jarid2 PE=1 SV=1 | Jarid2 | 1.07 |
| Q9D187 | Cytosolic iron-sulfur assembly component 2B OS=Mus musculus OX=10090 GN=Ciao2b PE=1 SV=1 | Ciao2b | 1.07 |
| Q8R0G7 | Protein spinster homolog 1 OS=Mus musculus OX=10090 GN=Spns1 PE=2 SV=1 | Spns1 | 1.07 |
| Q8VCS3 | Glycosaminoglycan xylosylkinase OS=Mus musculus OX=10090 GN=Fam20b PE=1 SV=1 | Fam20b | 1.07 |
| O35345 | Importin subunit alpha-7 OS=Mus musculus OX=10090 GN=Kpna6 PE=1 SV=2 | Kpna6 | 1.07 |
| Q8CIH5 | 1-phosphatidylinositol 4,5-bisphosphate phosphodiesterase gamma-2 OS=Mus musculus OX=10090 GN=Plcg2 PE=1 SV=1 | Plcg2 | 1.07 |
| Q9D7K5 | Distal membrane-arm assembly complex protein 2 OS=Mus musculus OX=10090 GN=Dmac2 PE=1 SV=1 | Dmac2 | 1.07 |
| P53996 | CCHC-type zinc finger nucleic acid binding protein OS=Mus musculus OX=10090 GN=Cnbp PE=1 SV=2 | Cnbp | 1.07 |
| Q62158 | Zinc finger protein RFP OS=Mus musculus OX=10090 GN=Trim27 PE=1 SV=2 | Trim27 | 1.07 |
| Q9DCR2 | AP-3 complex subunit sigma-1 OS=Mus musculus OX=10090 GN=Ap3s1 PE=1 SV=2 | Ap3s1 | 1.07 |
| Q99L13 | 3-hydroxyisobutyrate dehydrogenase, mitochondrial OS=Mus musculus OX=10090 GN=Hibadh PE=1 SV=1 | Hibadh | 1.07 |
| Q8JZK9 | Hydroxymethylglutaryl-CoA synthase, cytoplasmic OS=Mus musculus OX=10090 GN=Hmgcs1 PE=1 SV=1 | Hmgcs1 | 1.07 |
| Q9CQ36 | DNA polymerase epsilon subunit 4 OS=Mus musculus OX=10090 GN=Pole4 PE=1 SV=1 | Pole4 | 1.07 |
| P61600 | N-alpha-acetyltransferase 20 OS=Mus musculus OX=10090 GN=Naa20 PE=1 SV=1 | Naa20 | 1.07 |
| Q8N7N5 | DDB1- and CUL4-associated factor 8 OS=Mus musculus OX=10090 GN=Dcaf8 PE=1 SV=1 | Dcaf8 | 1.07 |
| O08808 | Protein diaphanous homolog 1 OS=Mus musculus OX=10090 GN=Diaph1 PE=1 SV=1 | Diaph1 | 1.07 |
| Q62448 | Eukaryotic translation initiation factor 4 gamma 2 OS=Mus musculus OX=10090 GN=Eif4g2 PE=1 SV=2 | Eif4g2 | 1.07 |
| Q8C6G8 | WD repeat-containing protein 26 OS=Mus musculus OX=10090 GN=Wdr26 PE=1 SV=3 | Wdr26 | 1.07 |
| O08582 | GTP-binding protein 1 OS=Mus musculus OX=10090 GN=Gtpbp1 PE=1 SV=2 | Gtpbp1 | 1.07 |
| O08583 | THO complex subunit 4 OS=Mus musculus OX=10090 GN=Alyref PE=1 SV=3 | Alyref | 1.07 |
| Q9CPS7 | RNA-binding protein PNO1 OS=Mus musculus OX=10090 GN=Pno1 PE=1 SV=1 | Pno1 | 1.07 |
| Q80TV8 | CLIP-associating protein 1 OS=Mus musculus OX=10090 GN=Clasp1 PE=1 SV=2 | Clasp1 | 1.08 |
| Q9JIF0 | Protein arginine N-methyltransferase 1 OS=Mus musculus OX=10090 GN=Prmt1 PE=1 SV=1 | Prmt1 | 1.08 |
| O54825 | Bystin OS=Mus musculus OX=10090 GN=Bysl PE=1 SV=3 | Bysl | 1.08 |
| P49718 | DNA replication licensing factor MCM5 OS=Mus musculus OX=10090 GN=Mcm5 PE=1 SV=2 | Mcm5 | 1.08 |
| O55236 | mRNA-capping enzyme OS=Mus musculus OX=10090 GN=Rngtt PE=1 SV=1 | Rngtt | 1.08 |
| P62876 | DNA-directed RNA polymerases I, II, and III subunit RPABC5 OS=Mus musculus OX=10090 GN=Polr2l PE=3 SV=1 | Polr2l | 1.08 |
| Q8BH58 | TIP41-like protein OS=Mus musculus OX=10090 GN=Tiprl PE=1 SV=1 | Tiprl | 1.08 |
| P14131 | 40S ribosomal protein S16 OS=Mus musculus OX=10090 GN=Rps16 PE=1 SV=4 | Rps16 | 1.08 |
| Q8CDC7 | Zinc finger and BTB domain-containing protein 9 OS=Mus musculus OX=10090 GN=Zbtb9 PE=2 SV=1 | Zbtb9 | 1.08 |
| Q3URQ0 | Testis-expressed protein 10 OS=Mus musculus OX=10090 GN=Tex10 PE=1 SV=1 | Tex10 | 1.08 |
| Q5F2E8 | Serine/threonine-protein kinase TAO1 OS=Mus musculus OX=10090 GN=Taok1 PE=1 SV=1 | Taok1 | 1.08 |
| Q80UG5 | Septin-9 OS=Mus musculus OX=10090 GN=Septin9 PE=1 SV=1 | Septin9 | 1.08 |
| Q8BFU2 | Histone H2A type 3 OS=Mus musculus OX=10090 GN=H2aw PE=1 SV=3 | H2aw | 1.08 |
| Q02819 | Nucleobindin-1 OS=Mus musculus OX=10090 GN=Nucb1 PE=1 SV=2 | Nucb1 | 1.08 |
| E9Q784 | Zinc finger CCCH domain-containing protein 13 OS=Mus musculus OX=10090 GN=Zc3h13 PE=1 SV=1 | Zc3h13 | 1.08 |
| Q6P542 | ATP-binding cassette sub-family F member 1 OS=Mus musculus OX=10090 GN=Abcf1 PE=1 SV=1 | Abcf1 | 1.08 |
| P11157 | Ribonucleoside-diphosphate reductase subunit M2 OS=Mus musculus OX=10090 GN=Rrm2 PE=1 SV=1 | Rrm2 | 1.08 |
| Q8BRH0 | Protein O-mannosyl-transferase TMTC3 OS=Mus musculus OX=10090 GN=Tmtc3 PE=1 SV=2 | Tmtc3 | 1.08 |
| Q6ZPF4 | Formin-like protein 3 OS=Mus musculus OX=10090 GN=Fmnl3 PE=1 SV=2 | Fmnl3 | 1.08 |
| Q8CA72 | Gigaxonin OS=Mus musculus OX=10090 GN=Gan PE=1 SV=2 | Gan | 1.08 |
| Q922X9 | Protein arginine N-methyltransferase 7 OS=Mus musculus OX=10090 GN=Prmt7 PE=1 SV=1 | Prmt7 | 1.08 |
| Q9CQC9 | GTP-binding protein SAR1b OS=Mus musculus OX=10090 GN=Sar1b PE=1 SV=1 | Sar1b | 1.08 |
| P70315 | Wiskott-Aldrich syndrome protein homolog OS=Mus musculus OX=10090 GN=Was PE=1 SV=1 | Was | 1.08 |
| Q8BVG4 | Dipeptidyl peptidase 9 OS=Mus musculus OX=10090 GN=Dpp9 PE=1 SV=2 | Dpp9 | 1.08 |
| Q3UA06 | Pachytene checkpoint protein 2 homolog OS=Mus musculus OX=10090 GN=Trip13 PE=1 SV=1 | Trip13 | 1.08 |
| Q9CXT8 | Mitochondrial-processing peptidase subunit beta OS=Mus musculus OX=10090 GN=Pmpcb PE=1 SV=1 | Pmpcb | 1.08 |
| Q91ZW2 | GDP-fucose protein O-fucosyltransferase 1 OS=Mus musculus OX=10090 GN=Pofut1 PE=1 SV=1 | Pofut1 | 1.08 |
| Q8R3S6 | Exocyst complex component 1 OS=Mus musculus OX=10090 GN=Exoc1 PE=1 SV=4 | Exoc1 | 1.08 |
| Q7TQC5 | Aprataxin OS=Mus musculus OX=10090 GN=Aptx PE=1 SV=2 | Aptx | 1.08 |
| P40694 | DNA-binding protein SMUBP-2 OS=Mus musculus OX=10090 GN=Ighmbp2 PE=1 SV=1 | Ighmbp2 | 1.08 |
| Q6P5D8 | Structural maintenance of chromosomes flexible hinge domain-containing protein 1 OS=Mus musculus OX=10090 GN=Smchd1 PE=1 SV=2 | Smchd1 | 1.08 |
| Q8BZQ7 | Anaphase-promoting complex subunit 2 OS=Mus musculus OX=10090 GN=Anapc2 PE=1 SV=2 | Anapc2 | 1.08 |
| Q91YE6 | Importin-9 OS=Mus musculus OX=10090 GN=Ipo9 PE=1 SV=3 | Ipo9 | 1.08 |
| O35658 | Complement component 1 Q subcomponent-binding protein, mitochondrial OS=Mus musculus OX=10090 GN=C1qbp PE=1 SV=1 | C1qbp | 1.08 |
| Q8BM85 | TBC domain-containing protein kinase-like protein OS=Mus musculus OX=10090 GN=Tbck PE=1 SV=1 | Tbck | 1.08 |
| Q921G6 | Leucine-rich repeat and calponin homology domain-containing protein 4 OS=Mus musculus OX=10090 GN=Lrch4 PE=1 SV=1 | Lrch4 | 1.08 |
| Q9Z1Q2 | Phosphatidylserine lipase ABHD16A OS=Mus musculus OX=10090 GN=Abhd16a PE=1 SV=3 | Abhd16a | 1.08 |
| Q60710 | Deoxynucleoside triphosphate triphosphohydrolase SAMHD1 OS=Mus musculus OX=10090 GN=Samhd1 PE=1 SV=3 | Samhd1 | 1.08 |
| Q8C522 | Endonuclease domain-containing 1 protein OS=Mus musculus OX=10090 GN=Endod1 PE=1 SV=2 | Endod1 | 1.08 |
| Q9ES74 | Serine/threonine-protein kinase Nek7 OS=Mus musculus OX=10090 GN=Nek7 PE=1 SV=1 | Nek7 | 1.08 |
| Q5SNZ0 | Girdin OS=Mus musculus OX=10090 GN=Ccdc88a PE=1 SV=2 | Ccdc88a | 1.08 |
| Q9D1R1 | Complex I assembly factor TMEM126B, mitochondrial OS=Mus musculus OX=10090 GN=Tmem126b PE=1 SV=1 | Tmem126b | 1.08 |
| P70302 | Stromal interaction molecule 1 OS=Mus musculus OX=10090 GN=Stim1 PE=1 SV=2 | Stim1 | 1.08 |
| Q7TMX5 | Protein SHQ1 homolog OS=Mus musculus OX=10090 GN=Shq1 PE=1 SV=2 | Shq1 | 1.08 |
| Q61009 | Scavenger receptor class B member 1 OS=Mus musculus OX=10090 GN=Scarb1 PE=1 SV=1 | Scarb1 | 1.08 |
| Q9QYB5 | Gamma-adducin OS=Mus musculus OX=10090 GN=Add3 PE=1 SV=2 | Add3 | 1.08 |
| O88569 | Heterogeneous nuclear ribonucleoproteins A2/B1 OS=Mus musculus OX=10090 GN=Hnrnpa2b1 PE=1 SV=2 | Hnrnpa2b1 | 1.08 |
| Q78IS1 | Transmembrane emp24 domain-containing protein 3 OS=Mus musculus OX=10090 GN=Tmed3 PE=1 SV=1 | Tmed3 | 1.08 |
| P62242 | 40S ribosomal protein S8 OS=Mus musculus OX=10090 GN=Rps8 PE=1 SV=2 | Rps8 | 1.08 |
| Q8R349 | Cell division cycle protein 16 homolog OS=Mus musculus OX=10090 GN=Cdc16 PE=1 SV=1 | Cdc16 | 1.08 |
| Q6P2L6 | Histone-lysine N-methyltransferase NSD3 OS=Mus musculus OX=10090 GN=Nsd3 PE=1 SV=2 | Nsd3 | 1.08 |
| O35387 | HCLS1-associated protein X-1 OS=Mus musculus OX=10090 GN=Hax1 PE=1 SV=1 | Hax1 | 1.08 |
| Q8K003 | Translation machinery-associated protein 7 OS=Mus musculus OX=10090 GN=Tma7 PE=3 SV=1 | Tma7 | 1.08 |
| P23492 | Purine nucleoside phosphorylase OS=Mus musculus OX=10090 GN=Pnp PE=1 SV=2 | Pnp | 1.08 |
| Q91XB0 | Three-prime repair exonuclease 1 OS=Mus musculus OX=10090 GN=Trex1 PE=1 SV=2 | Trex1 | 1.08 |
| P60521 | Gamma-aminobutyric acid receptor-associated protein-like 2 OS=Mus musculus OX=10090 GN=Gabarapl2 PE=1 SV=1 | Gabarapl2 | 1.08 |
| Q9DBG6 | Dolichyl-diphosphooligosaccharide--protein glycosyltransferase subunit 2 OS=Mus musculus OX=10090 GN=Rpn2 PE=1 SV=1 | Rpn2 | 1.08 |
| P70268 | Serine/threonine-protein kinase N1 OS=Mus musculus OX=10090 GN=Pkn1 PE=1 SV=3 | Pkn1 | 1.08 |
| Q99L27 | GMP reductase 2 OS=Mus musculus OX=10090 GN=Gmpr2 PE=1 SV=2 | Gmpr2 | 1.08 |
| Q9D600 | DNA replication complex GINS protein PSF2 OS=Mus musculus OX=10090 GN=Gins2 PE=1 SV=1 | Gins2 | 1.08 |
| Q9CWL8 | Beta-catenin-like protein 1 OS=Mus musculus OX=10090 GN=Ctnnbl1 PE=1 SV=1 | Ctnnbl1 | 1.08 |
| O08709 | Peroxiredoxin-6 OS=Mus musculus OX=10090 GN=Prdx6 PE=1 SV=3 | Prdx6 | 1.08 |
| Q06138 | Calcium-binding protein 39 OS=Mus musculus OX=10090 GN=Cab39 PE=1 SV=2 | Cab39 | 1.08 |
| P63034 | Cytohesin-2 OS=Mus musculus OX=10090 GN=Cyth2 PE=1 SV=2 | Cyth2 | 1.08 |
| Q8C779 | RPA-related protein RADX OS=Mus musculus OX=10090 GN=Radx PE=2 SV=1 | Radx | 1.08 |
| Q921D4 | Mediator of RNA polymerase II transcription subunit 6 OS=Mus musculus OX=10090 GN=Med6 PE=1 SV=2 | Med6 | 1.08 |
| A6X919 | Probable C-mannosyltransferase DPY19L1 OS=Mus musculus OX=10090 GN=Dpy19l1 PE=1 SV=1 | Dpy19l1 | 1.08 |
| Q9D1M7 | Peptidyl-prolyl cis-trans isomerase FKBP11 OS=Mus musculus OX=10090 GN=Fkbp11 PE=1 SV=1 | Fkbp11 | 1.08 |
| Q6ZWV7 | 60S ribosomal protein L35 OS=Mus musculus OX=10090 GN=Rpl35 PE=1 SV=1 | Rpl35 | 1.08 |
| B2RY04 | Dedicator of cytokinesis protein 5 OS=Mus musculus OX=10090 GN=Dock5 PE=1 SV=2 | Dock5 | 1.08 |
| Q9JL26 | Formin-like protein 1 OS=Mus musculus OX=10090 GN=Fmnl1 PE=1 SV=1 | Fmnl1 | 1.08 |
| P29594 | Caspase-2 OS=Mus musculus OX=10090 GN=Casp2 PE=1 SV=5 | Casp2 | 1.08 |
| Q8BU31 | Ras-related protein Rap-2c OS=Mus musculus OX=10090 GN=Rap2c PE=1 SV=1 | Rap2c | 1.08 |
| Q99J87 | Probable ATP-dependent RNA helicase DHX58 OS=Mus musculus OX=10090 GN=Dhx58 PE=1 SV=2 | Dhx58 | 1.08 |
| Q9JMG1 | Endothelial differentiation-related factor 1 OS=Mus musculus OX=10090 GN=Edf1 PE=1 SV=1 | Edf1 | 1.08 |
| Q3UMC0 | ATPase family protein 2 homolog OS=Mus musculus OX=10090 GN=Spata5 PE=1 SV=2 | Spata5 | 1.08 |
| P43406 | Integrin alpha-V OS=Mus musculus OX=10090 GN=Itgav PE=1 SV=2 | Itgav | 1.08 |
| Q99N93 | 39S ribosomal protein L16, mitochondrial OS=Mus musculus OX=10090 GN=Mrpl16 PE=1 SV=1 | Mrpl16 | 1.08 |
| O08547 | Vesicle-trafficking protein SEC22b OS=Mus musculus OX=10090 GN=Sec22b PE=1 SV=3 | Sec22b | 1.08 |
| Q8CDN6 | Thioredoxin-like protein 1 OS=Mus musculus OX=10090 GN=Txnl1 PE=1 SV=3 | Txnl1 | 1.08 |
| Q921I9 | Exosome complex component RRP41 OS=Mus musculus OX=10090 GN=Exosc4 PE=1 SV=3 | Exosc4 | 1.08 |
| Q8JZN5 | Complex I assembly factor ACAD9, mitochondrial OS=Mus musculus OX=10090 GN=Acad9 PE=1 SV=2 | Acad9 | 1.08 |
| Q8R001 | Microtubule-associated protein RP/EB family member 2 OS=Mus musculus OX=10090 GN=Mapre2 PE=1 SV=1 | Mapre2 | 1.08 |
| Q6A028 | Switch-associated protein 70 OS=Mus musculus OX=10090 GN=Swap70 PE=1 SV=2 | Swap70 | 1.08 |
| Q60715 | Prolyl 4-hydroxylase subunit alpha-1 OS=Mus musculus OX=10090 GN=P4ha1 PE=1 SV=2 | P4ha1 | 1.09 |
| O88447 | Kinesin light chain 1 OS=Mus musculus OX=10090 GN=Klc1 PE=1 SV=3 | Klc1 | 1.09 |
| Q8R4U7 | Leucine zipper protein 1 OS=Mus musculus OX=10090 GN=Luzp1 PE=1 SV=2 | Luzp1 | 1.09 |
| P60229 | Eukaryotic translation initiation factor 3 subunit E OS=Mus musculus OX=10090 GN=Eif3e PE=1 SV=1 | Eif3e | 1.09 |
| Q9BDB7 | Interferon-induced protein 44-like OS=Mus musculus OX=10090 GN=Ifi44l PE=2 SV=2 | Ifi44l | 1.09 |
| Q9CQD1 | Ras-related protein Rab-5A OS=Mus musculus OX=10090 GN=Rab5a PE=1 SV=1 | Rab5a | 1.09 |
| Q9Z0G0 | PDZ domain-containing protein GIPC1 OS=Mus musculus OX=10090 GN=Gipc1 PE=1 SV=1 | Gipc1 | 1.09 |
| Q9ER88 | 28S ribosomal protein S29, mitochondrial OS=Mus musculus OX=10090 GN=Dap3 PE=1 SV=1 | Dap3 | 1.09 |
| Q8BQ30 | Phostensin OS=Mus musculus OX=10090 GN=Ppp1r18 PE=1 SV=1 | Ppp1r18 | 1.09 |
| P26041 | Moesin OS=Mus musculus OX=10090 GN=Msn PE=1 SV=3 | Msn | 1.09 |
| Q6P5F9 | Exportin-1 OS=Mus musculus OX=10090 GN=Xpo1 PE=1 SV=1 | Xpo1 | 1.09 |
| G3X9G7 | Zinc finger protein 809 OS=Mus musculus OX=10090 GN=Zfp809 PE=2 SV=1 | Zfp809 | 1.09 |
| Q8CIG9 | F-box/LRR-repeat protein 8 OS=Mus musculus OX=10090 GN=Fbxl8 PE=1 SV=1 | Fbxl8 | 1.09 |
| Q8K358 | Phosphatidylinositol glycan anchor biosynthesis class U protein OS=Mus musculus OX=10090 GN=Pigu PE=1 SV=4 | Pigu | 1.09 |
| Q8BFP9 | [Pyruvate dehydrogenase (acetyl-transferring)] kinase isozyme 1, mitochondrial OS=Mus musculus OX=10090 GN=Pdk1 PE=1 SV=2 | Pdk1 | 1.09 |
| O70503 | Very-long-chain 3-oxoacyl-CoA reductase OS=Mus musculus OX=10090 GN=Hsd17b12 PE=1 SV=1 | Hsd17b12 | 1.09 |
| Q9QZD9 | Eukaryotic translation initiation factor 3 subunit I OS=Mus musculus OX=10090 GN=Eif3i PE=1 SV=1 | Eif3i | 1.09 |
| Q8BUI3 | Leucine-rich repeat and WD repeat-containing protein 1 OS=Mus musculus OX=10090 GN=LRWD1 PE=2 SV=1 | LRWD1 | 1.09 |
| E9Q2M9 | WD repeat- and FYVE domain-containing protein 4 OS=Mus musculus OX=10090 GN=Wdfy4 PE=1 SV=2 | Wdfy4 | 1.09 |
| Q9QXE7 | F-box-like/WD repeat-containing protein TBL1X OS=Mus musculus OX=10090 GN=Tbl1x PE=1 SV=2 | Tbl1x | 1.09 |
| Q91YP3 | Deoxyribose-phosphate aldolase OS=Mus musculus OX=10090 GN=Dera PE=1 SV=1 | Dera | 1.09 |
| Q8R5K4 | Nucleolar protein 6 OS=Mus musculus OX=10090 GN=Nol6 PE=2 SV=2 | Nol6 | 1.09 |
| Q3TL26 | Dimethyladenosine transferase 2, mitochondrial OS=Mus musculus OX=10090 GN=Tfb2m PE=2 SV=1 | Tfb2m | 1.09 |
| Q62351 | Transferrin receptor protein 1 OS=Mus musculus OX=10090 GN=Tfrc PE=1 SV=1 | Tfrc | 1.09 |
| Q9DCH2 | Ribonuclease P protein subunit p20 OS=Mus musculus OX=10090 GN=Pop7 PE=2 SV=1 | Pop7 | 1.09 |
| P50429 | Arylsulfatase B OS=Mus musculus OX=10090 GN=Arsb PE=1 SV=3 | Arsb | 1.09 |
| Q8VE88 | Protein FAM114A2 OS=Mus musculus OX=10090 GN=Fam114a2 PE=1 SV=2 | Fam114a2 | 1.09 |
| Q9D6J6 | NADH dehydrogenase [ubiquinone] flavoprotein 2, mitochondrial OS=Mus musculus OX=10090 GN=Ndufv2 PE=1 SV=2 | Ndufv2 | 1.09 |
| P30285 | Cyclin-dependent kinase 4 OS=Mus musculus OX=10090 GN=Cdk4 PE=1 SV=1 | Cdk4 | 1.09 |
| Q9CYK1 | Tryptophan--tRNA ligase, mitochondrial OS=Mus musculus OX=10090 GN=Wars2 PE=1 SV=2 | Wars2 | 1.09 |
| O70310 | Glycylpeptide N-tetradecanoyltransferase 1 OS=Mus musculus OX=10090 GN=Nmt1 PE=1 SV=1 | Nmt1 | 1.09 |
| Q91V76 | Ester hydrolase C11orf54 homolog OS=Mus musculus OX=10090 PE=1 SV=1 | -- | 1.09 |
| Q8VIB3 | Type 2 lactosamine alpha-2,3-sialyltransferase OS=Mus musculus OX=10090 GN=St3gal6 PE=2 SV=3 | St3gal6 | 1.09 |
| Q920E5 | Farnesyl pyrophosphate synthase OS=Mus musculus OX=10090 GN=Fdps PE=1 SV=1 | Fdps | 1.09 |
| Q8BWM0 | Prostaglandin E synthase 2 OS=Mus musculus OX=10090 GN=Ptges2 PE=1 SV=3 | Ptges2 | 1.09 |
| Q9CWS4 | Integrator complex subunit 11 OS=Mus musculus OX=10090 GN=Ints11 PE=1 SV=1 | Ints11 | 1.09 |
| P11928 | 2'-5'-oligoadenylate synthase 1A OS=Mus musculus OX=10090 GN=Oas1a PE=1 SV=2 | Oas1a | 1.09 |
| Q9CWN7 | CCR4-NOT transcription complex subunit 11 OS=Mus musculus OX=10090 GN=Cnot11 PE=1 SV=1 | Cnot11 | 1.09 |
| O70311 | Glycylpeptide N-tetradecanoyltransferase 2 OS=Mus musculus OX=10090 GN=Nmt2 PE=1 SV=1 | Nmt2 | 1.09 |
| Q3THK7 | GMP synthase [glutamine-hydrolyzing] OS=Mus musculus OX=10090 GN=Gmps PE=1 SV=2 | Gmps | 1.09 |
| Q8BHX3 | Borealin OS=Mus musculus OX=10090 GN=Cdca8 PE=1 SV=2 | Cdca8 | 1.09 |
| B1AVY7 | Kinesin-like protein KIF16B OS=Mus musculus OX=10090 GN=Kif16b PE=1 SV=1 | Kif16b | 1.09 |
| Q9CR64 | Protein kish-A OS=Mus musculus OX=10090 GN=Tmem167a PE=1 SV=1 | Tmem167a | 1.09 |
| Q9JM76 | Actin-related protein 2/3 complex subunit 3 OS=Mus musculus OX=10090 GN=Arpc3 PE=1 SV=3 | Arpc3 | 1.09 |
| Q08509 | Epidermal growth factor receptor kinase substrate 8 OS=Mus musculus OX=10090 GN=Eps8 PE=1 SV=2 | Eps8 | 1.09 |
| O89086 | RNA-binding protein 3 OS=Mus musculus OX=10090 GN=Rbm3 PE=1 SV=1 | Rbm3 | 1.09 |
| P63017 | Heat shock cognate 71 kDa protein OS=Mus musculus OX=10090 GN=Hspa8 PE=1 SV=1 | Hspa8 | 1.09 |
| Q9CPU0 | Lactoylglutathione lyase OS=Mus musculus OX=10090 GN=Glo1 PE=1 SV=3 | Glo1 | 1.09 |
| P57759 | Endoplasmic reticulum resident protein 29 OS=Mus musculus OX=10090 GN=Erp29 PE=1 SV=2 | Erp29 | 1.09 |
| Q9DCJ5 | NADH dehydrogenase [ubiquinone] 1 alpha subcomplex subunit 8 OS=Mus musculus OX=10090 GN=Ndufa8 PE=1 SV=3 | Ndufa8 | 1.09 |
| Q9JLN9 | Serine/threonine-protein kinase mTOR OS=Mus musculus OX=10090 GN=Mtor PE=1 SV=2 | Mtor | 1.09 |
| O88630 | Golgi SNAP receptor complex member 1 OS=Mus musculus OX=10090 GN=Gosr1 PE=1 SV=2 | Gosr1 | 1.09 |
| Q6NZQ4 | PAX-interacting protein 1 OS=Mus musculus OX=10090 GN=Paxip1 PE=1 SV=1 | Paxip1 | 1.09 |
| Q8BGB8 | Ubiquinone biosynthesis protein COQ4 homolog, mitochondrial OS=Mus musculus OX=10090 GN=Coq4 PE=1 SV=1 | Coq4 | 1.09 |
| Q6GQT6 | Sterol regulatory element-binding protein cleavage-activating protein OS=Mus musculus OX=10090 GN=Scap PE=1 SV=1 | Scap | 1.09 |
| Q80XP8 | Protein FAM76B OS=Mus musculus OX=10090 GN=Fam76b PE=1 SV=1 | Fam76b | 1.09 |
| Q9ES00 | Ubiquitin conjugation factor E4 B OS=Mus musculus OX=10090 GN=Ube4b PE=1 SV=3 | Ube4b | 1.09 |
| Q8R1Q8 | Cytoplasmic dynein 1 light intermediate chain 1 OS=Mus musculus OX=10090 GN=Dync1li1 PE=1 SV=1 | Dync1li1 | 1.09 |
| Q8BVU0 | DISP complex protein LRCH3 OS=Mus musculus OX=10090 GN=Lrch3 PE=1 SV=3 | Lrch3 | 1.09 |
| Q7TQK5 | Coiled-coil domain-containing protein 93 OS=Mus musculus OX=10090 GN=Ccdc93 PE=1 SV=1 | Ccdc93 | 1.09 |
| Q9CWI3 | BRCA2 and CDKN1A-interacting protein OS=Mus musculus OX=10090 GN=Bccip PE=1 SV=1 | Bccip | 1.09 |
| Q8CE90 | Dual specificity mitogen-activated protein kinase kinase 7 OS=Mus musculus OX=10090 GN=Map2k7 PE=1 SV=1 | Map2k7 | 1.09 |
| Q91X96 | Guanine nucleotide exchange factor MSS4 OS=Mus musculus OX=10090 GN=Rabif PE=1 SV=1 | Rabif | 1.09 |
| Q8BZW8 | NHL repeat-containing protein 2 OS=Mus musculus OX=10090 GN=Nhlrc2 PE=1 SV=1 | Nhlrc2 | 1.09 |
| Q9CYN2 | Signal peptidase complex subunit 2 OS=Mus musculus OX=10090 GN=Spcs2 PE=1 SV=1 | Spcs2 | 1.09 |
| P97434 | Myosin phosphatase Rho-interacting protein OS=Mus musculus OX=10090 GN=Mprip PE=1 SV=2 | Mprip | 1.09 |
| Q80XK6 | Autophagy-related protein 2 homolog B OS=Mus musculus OX=10090 GN=Atg2b PE=1 SV=3 | Atg2b | 1.09 |
| Q8CI33 | CWF19-like protein 1 OS=Mus musculus OX=10090 GN=Cwf19l1 PE=1 SV=2 | Cwf19l1 | 1.09 |
| A2AB59 | Rho GTPase-activating protein 27 OS=Mus musculus OX=10090 GN=Arhgap27 PE=1 SV=1 | Arhgap27 | 1.09 |
| P62908 | 40S ribosomal protein S3 OS=Mus musculus OX=10090 GN=Rps3 PE=1 SV=1 | Rps3 | 1.09 |
| Q8BU30 | Isoleucine--tRNA ligase, cytoplasmic OS=Mus musculus OX=10090 GN=Iars1 PE=1 SV=2 | Iars1 | 1.09 |
| Q91XL3 | UDP-glucuronic acid decarboxylase 1 OS=Mus musculus OX=10090 GN=Uxs1 PE=1 SV=1 | Uxs1 | 1.09 |
| Q8BX09 | Retinoblastoma-binding protein 5 OS=Mus musculus OX=10090 GN=Rbbp5 PE=1 SV=2 | Rbbp5 | 1.09 |
| Q4VAA2 | Protein CDV3 OS=Mus musculus OX=10090 GN=Cdv3 PE=1 SV=2 | Cdv3 | 1.09 |
| Q9CPY7 | Cytosol aminopeptidase OS=Mus musculus OX=10090 GN=Lap3 PE=1 SV=3 | Lap3 | 1.09 |
| Q8BVF2 | Phosducin-like protein 3 OS=Mus musculus OX=10090 GN=Pdcl3 PE=1 SV=1 | Pdcl3 | 1.09 |
| P35700 | Peroxiredoxin-1 OS=Mus musculus OX=10090 GN=Prdx1 PE=1 SV=1 | Prdx1 | 1.09 |
| Q8CHK3 | Lysophospholipid acyltransferase 7 OS=Mus musculus OX=10090 GN=Mboat7 PE=1 SV=1 | Mboat7 | 1.09 |
| P40124 | Adenylyl cyclase-associated protein 1 OS=Mus musculus OX=10090 GN=Cap1 PE=1 SV=4 | Cap1 | 1.09 |
| E9Q1P8 | Interferon regulatory factor 2-binding protein 2 OS=Mus musculus OX=10090 GN=Irf2bp2 PE=1 SV=1 | Irf2bp2 | 1.09 |
| P35290 | Ras-related protein Rab-24 OS=Mus musculus OX=10090 GN=Rab24 PE=1 SV=2 | Rab24 | 1.09 |
| Q5XF89 | Polyamine-transporting ATPase 13A3 OS=Mus musculus OX=10090 GN=Atp13a3 PE=1 SV=1 | Atp13a3 | 1.09 |
| O35343 | Importin subunit alpha-3 OS=Mus musculus OX=10090 GN=Kpna4 PE=1 SV=1 | Kpna4 | 1.09 |
| Q8BQZ4 | Ral GTPase-activating protein subunit beta OS=Mus musculus OX=10090 GN=Ralgapb PE=1 SV=2 | Ralgapb | 1.09 |
| Q9CY18 | Sorting nexin-7 OS=Mus musculus OX=10090 GN=Snx7 PE=1 SV=1 | Snx7 | 1.09 |
| Q91ZF0 | DnaJ homolog subfamily C member 24 OS=Mus musculus OX=10090 GN=Dnajc24 PE=1 SV=4 | Dnajc24 | 1.09 |
| Q8K0V4 | CCR4-NOT transcription complex subunit 3 OS=Mus musculus OX=10090 GN=Cnot3 PE=1 SV=1 | Cnot3 | 1.09 |
| Q62186 | Translocon-associated protein subunit delta OS=Mus musculus OX=10090 GN=Ssr4 PE=1 SV=1 | Ssr4 | 1.09 |
| Q9D0M0 | Exosome complex exonuclease RRP42 OS=Mus musculus OX=10090 GN=Exosc7 PE=1 SV=2 | Exosc7 | 1.10 |
| Q91V92 | ATP-citrate synthase OS=Mus musculus OX=10090 GN=Acly PE=1 SV=1 | Acly | 1.10 |
| P62270 | 40S ribosomal protein S18 OS=Mus musculus OX=10090 GN=Rps18 PE=1 SV=3 | Rps18 | 1.10 |
| Q9R0P3 | S-formylglutathione hydrolase OS=Mus musculus OX=10090 GN=Esd PE=1 SV=1 | Esd | 1.10 |
| P41241 | Tyrosine-protein kinase CSK OS=Mus musculus OX=10090 GN=Csk PE=1 SV=2 | Csk | 1.10 |
| Q5FWK3 | Rho GTPase-activating protein 1 OS=Mus musculus OX=10090 GN=Arhgap1 PE=1 SV=1 | Arhgap1 | 1.10 |
| Q9D6Y9 | 1,4-alpha-glucan-branching enzyme OS=Mus musculus OX=10090 GN=Gbe1 PE=1 SV=1 | Gbe1 | 1.10 |
| Q64337 | Sequestosome-1 OS=Mus musculus OX=10090 GN=Sqstm1 PE=1 SV=1 | Sqstm1 | 1.10 |
| P10810 | Monocyte differentiation antigen CD14 OS=Mus musculus OX=10090 GN=Cd14 PE=1 SV=1 | Cd14 | 1.10 |
| P80318 | T-complex protein 1 subunit gamma OS=Mus musculus OX=10090 GN=Cct3 PE=1 SV=1 | Cct3 | 1.10 |
| Q91VR8 | Protein BRICK1 OS=Mus musculus OX=10090 GN=Brk1 PE=1 SV=1 | Brk1 | 1.10 |
| Q9JK91 | DNA mismatch repair protein Mlh1 OS=Mus musculus OX=10090 GN=Mlh1 PE=1 SV=2 | Mlh1 | 1.10 |
| Q8C142 | Low density lipoprotein receptor adapter protein 1 OS=Mus musculus OX=10090 GN=Ldlrap1 PE=1 SV=3 | Ldlrap1 | 1.10 |
| Q8K0C4 | Lanosterol 14-alpha demethylase OS=Mus musculus OX=10090 GN=Cyp51a1 PE=1 SV=1 | Cyp51a1 | 1.10 |
| O35522 | Proteasome subunit beta type-9 OS=Mus musculus bactrianus OX=35531 GN=Psmb9 PE=1 SV=1 | Psmb9 | 1.10 |
| Q8VC03 | Echinoderm microtubule-associated protein-like 3 OS=Mus musculus OX=10090 GN=Eml3 PE=1 SV=1 | Eml3 | 1.10 |
| O88543 | COP9 signalosome complex subunit 3 OS=Mus musculus OX=10090 GN=Cops3 PE=1 SV=3 | Cops3 | 1.10 |
| Q8C1B7 | Septin-11 OS=Mus musculus OX=10090 GN=Septin11 PE=1 SV=4 | Septin11 | 1.10 |
| O88983 | Syntaxin-8 OS=Mus musculus OX=10090 GN=Stx8 PE=1 SV=1 | Stx8 | 1.10 |
| Q8R1B4 | Eukaryotic translation initiation factor 3 subunit C OS=Mus musculus OX=10090 GN=Eif3c PE=1 SV=1 | Eif3c | 1.10 |
| P14152 | Malate dehydrogenase, cytoplasmic OS=Mus musculus OX=10090 GN=Mdh1 PE=1 SV=3 | Mdh1 | 1.10 |
| P14824 | Annexin A6 OS=Mus musculus OX=10090 GN=Anxa6 PE=1 SV=3 | Anxa6 | 1.10 |
| Q9EST5 | Acidic leucine-rich nuclear phosphoprotein 32 family member B OS=Mus musculus OX=10090 GN=Anp32b PE=1 SV=1 | Anp32b | 1.10 |
| Q9WUM5 | Succinate--CoA ligase [ADP/GDP-forming] subunit alpha, mitochondrial OS=Mus musculus OX=10090 GN=Suclg1 PE=1 SV=4 | Suclg1 | 1.10 |
| Q8K4R9 | Disks large-associated protein 5 OS=Mus musculus OX=10090 GN=Dlgap5 PE=1 SV=2 | Dlgap5 | 1.10 |
| Q8BMI0 | F-box only protein 38 OS=Mus musculus OX=10090 GN=Fbxo38 PE=1 SV=1 | Fbxo38 | 1.10 |
| Q8K2C9 | Very-long-chain (3R)-3-hydroxyacyl-CoA dehydratase 3 OS=Mus musculus OX=10090 GN=Hacd3 PE=1 SV=2 | Hacd3 | 1.10 |
| Q9CSU0 | Regulation of nuclear pre-mRNA domain-containing protein 1B OS=Mus musculus OX=10090 GN=Rprd1b PE=1 SV=2 | Rprd1b | 1.10 |
| Q80U87 | Ubiquitin carboxyl-terminal hydrolase 8 OS=Mus musculus OX=10090 GN=Usp8 PE=1 SV=2 | Usp8 | 1.10 |
| Q8C2E4 | Pentatricopeptide repeat-containing protein 1, mitochondrial OS=Mus musculus OX=10090 GN=Ptcd1 PE=2 SV=2 | Ptcd1 | 1.10 |
| Q9WTI7 | Unconventional myosin-Ic OS=Mus musculus OX=10090 GN=Myo1c PE=1 SV=2 | Myo1c | 1.10 |
| P11440 | Cyclin-dependent kinase 1 OS=Mus musculus OX=10090 GN=Cdk1 PE=1 SV=3 | Cdk1 | 1.10 |
| Q8BH04 | Phosphoenolpyruvate carboxykinase [GTP], mitochondrial OS=Mus musculus OX=10090 GN=Pck2 PE=1 SV=1 | Pck2 | 1.10 |
| Q2EMV9 | Protein mono-ADP-ribosyltransferase PARP14 OS=Mus musculus OX=10090 GN=Parp14 PE=1 SV=3 | Parp14 | 1.10 |
| P58389 | Serine/threonine-protein phosphatase 2A activator OS=Mus musculus OX=10090 GN=Ptpa PE=1 SV=1 | Ptpa | 1.10 |
| Q9QWH1 | Polyhomeotic-like protein 2 OS=Mus musculus OX=10090 GN=Phc2 PE=1 SV=1 | Phc2 | 1.10 |
| Q8BH86 | D-glutamate cyclase, mitochondrial OS=Mus musculus OX=10090 GN=Dglucy PE=1 SV=1 | Dglucy | 1.10 |
| Q6ZPV2 | Chromatin-remodeling ATPase INO80 OS=Mus musculus OX=10090 GN=Ino80 PE=1 SV=2 | Ino80 | 1.10 |
| Q80ZK0 | 28S ribosomal protein S10, mitochondrial OS=Mus musculus OX=10090 GN=Mrps10 PE=1 SV=1 | Mrps10 | 1.10 |
| Q8BMT4 | Transforming growth factor beta activator LRRC33 OS=Mus musculus OX=10090 GN=Nrros PE=1 SV=2 | Nrros | 1.10 |
| Q8C7V8 | Coiled-coil domain-containing protein 134 OS=Mus musculus OX=10090 GN=Ccdc134 PE=1 SV=1 | Ccdc134 | 1.10 |
| Q9JHQ5 | Leucine zipper transcription factor-like protein 1 OS=Mus musculus OX=10090 GN=Lztfl1 PE=1 SV=1 | Lztfl1 | 1.10 |
| P31230 | Aminoacyl tRNA synthase complex-interacting multifunctional protein 1 OS=Mus musculus OX=10090 GN=Aimp1 PE=1 SV=2 | Aimp1 | 1.10 |
| Q7TSE6 | Serine/threonine-protein kinase 38-like OS=Mus musculus OX=10090 GN=Stk38l PE=1 SV=2 | Stk38l | 1.10 |
| P62900 | 60S ribosomal protein L31 OS=Mus musculus OX=10090 GN=Rpl31 PE=1 SV=1 | Rpl31 | 1.10 |
| Q8BRT1 | CLIP-associating protein 2 OS=Mus musculus OX=10090 GN=Clasp2 PE=1 SV=1 | Clasp2 | 1.10 |
| Q9D1G1 | Ras-related protein Rab-1B OS=Mus musculus OX=10090 GN=Rab1b PE=1 SV=1 | Rab1b | 1.10 |
| Q921G8 | Gamma-tubulin complex component 2 OS=Mus musculus OX=10090 GN=Tubgcp2 PE=1 SV=2 | Tubgcp2 | 1.10 |
| Q14AX6 | Cyclin-dependent kinase 12 OS=Mus musculus OX=10090 GN=Cdk12 PE=1 SV=2 | Cdk12 | 1.10 |
[truncated: 754,266 more chars]
